# Supplementary material for: Identification of ecogeographical gaps in the Spanish Aegilops collections with potential tolerance to drought and salinity
Source: PeerJ. 2017 Jul 27;5:e3494. doi: 10.7717/peerj.3494 (PMC5534164; doi:10.7717/peerj.3494)
Supplement: Table S1 [file peerj-05-3494-s001.docx]

Ecogeographical variables considered in the characterization of the occurrence sites of the targeted *Aegilops* species in Spain

| **Ecogeographical component** | **Description** | **Unit** | **Source** |
| --- | --- | --- | --- |
| Bioclimatic | Monthly precipitation (January to June) | mm | Hijmans et al. (2005) |
|  | Monthly mean temperature (January to June) | °C | Hijmans et al. (2005) |
|  | Monthly minimum temperature (January to June) | °C | Hijmans et al. (2005) |
|  | Monthly maximum temperature  (January to June) | °C | Hijmans et al. (2005) |
|  | Annual mean temperatura | °C | Hijmans et al. (2005) |
|  | Mean daily temperature range (Mean monthly (max temp - min temp)) | °C | Hijmans et al. (2005) |
|  | Isothermality (bio_2/bio_7) (* 100) | - | Hijmans et al. (2005) |
|  | Temperature seasonality (standard deviation *100) | - | Hijmans et al. (2005) |
|  | Minimum temperature of coldest month | °C | Hijmans et al. (2005) |
|  | Annual temperature range (BIO5-BIO6) | °C | Hijmans et al. (2005) |
|  | Mean temperature of wettest quarter | °C | Hijmans et al. (2005) |
|  | Mean temperature of coldest quarter | °C | Hijmans et al. (2005) |
|  | Annual precipitation | mm | Hijmans et al. (2005) |
|  | Precipitation of wettest month | mm | Hijmans et al. (2005) |
|  | Precipitation seasonality (Coefficient of variation) |  | Hijmans et al. (2005) |
|  | Precipitation of wettest quarter | mm | Hijmans et al. (2005) |
|  | Precipitation of coldest quarter | mm | Hijmans et al. (2005) |
| Geophysic | Elevation | m | Hijmans et al. (2005) |
|  | Slope | ° | Jarvis et al. (2008) |
|  | Northness | ° | Jarvis et al. (2008) |
|  | Eastness | ° | Jarvis et al. (2008) |
| Edaphic | Reference depth of the soil unit | m | HWS Database (2012) |
|  | Topsoil gravel content | % vol. | HWS Database (2012) |
|  | Topsoil sand fraction | % weight | HWS Database (2012) |
|  | Topsoil silt fraction | % weight | HWS Database (2012) |
|  | Topsoil clay fraction | % weight | HWS Database (2012) |
|  | Topsoil reference bulk density | % weight | HWS Database (2012) |
|  | Topsoil organic carbon | % weight | HWS Database (2012) |
|  | Topsoil pH (H_2_O) | -log(H+) | HWS Database (2012) |
|  | Topsoil CEC due to clay fraction | cmol/kg | HWS Database (2012) |
|  | Topsoil CEC (soil) | cmol/kg | HWS Database (2012) |
|  | Topsoil base saturation | % | HWS Database (2012) |
|  | Topsoil total exchangeable bases | cmol/kg | HWS Database (2012) |
|  | Topsoil calcium carbonate content | % weight | HWS Database (2012) |
|  | Topsoil calcium sulphate (gypsum) content | % weight | HWS Database (2012) |
|  | Topsoil sodicity | % | HWS Database (2012) |
|  | Topsoil salinity | dS/m | HWS Database (2012) |

References

- Hijmans RJ, Cameron SE, Parra JL, Jones PG, Jarvis A. 2005. Very high resolution interpolated climate surfaces for global land areas. International Journal of Climatology 25:1965–1978 DOI 10.1002/joc.1276.
- HWS Database 2012. Harmonized world soil (HWS) Database (version 12). Rome, Italy, Laxenburg, Austria: FAO, IIASA. Available at http://webarchive.iiasa.ac.at/ Research/ LUC/ External-World-soil-database/HTML/ (accessed on 01 April 2013).
- Jarvis A, Reuter HI, Nelson A, Guevara E. 2008. Digital Elevation Models (DEM) of the Shuttle Radar Topography Mission (SRTM) Hole-filled SRTM for the globe Version 4. Available at http:// srtm.csi.cgiar.org/ (accessed on 07 January 2016).
