# Supplementary material for: Identification of ecogeographical gaps in the Spanish Aegilops collections with potential tolerance to drought and salinity
Source: PeerJ. 2017 Jul 27;5:e3494. doi: 10.7717/peerj.3494 (PMC5534164; doi:10.7717/peerj.3494)
Supplement: Table S2 [file peerj-05-3494-s002.docx]

Environmental characteristics (average values) of the different categories of the ELC map for each *Aegilops* species in Spain

|  | *Ae. biuncialis* | | | | | | | |
| --- | --- | --- | --- | --- | --- | --- | --- | --- |
|  | Bioclimatic component | | | Geophysic component | | Edaphic component | | |
| ELC category | Isothermality | Precipitation in the wettest month | Minimum temperature in January | Eastness | Northness | Topsoil reference bulk density | Topsoil salinity | Topsoil base saturation |
| 1 | 37 | 61 | -0.1 | 0.6 | 0.6 | 1.6 | 0.1 | 92 |
| 2 | 37 | 83 | -1.5 | 0.5 | 0.5 | 1.4 | 0.1 | 35 |
| 3 | 37 | 65 | -0.3 | 0.5 | 0.5 | 1.4 | 0.3 | 99 |
| 4 | 37 | 62 | 0.1 | -0.5 | -0.5 | 1.6 | 0.1 | 91 |
| 5 | 37 | 79 | -0.9 | -0.6 | -0.6 | 1.4 | 0.1 | 43 |
| 6 | 37 | 65 | -0.2 | -0.6 | -0.6 | 1.4 | 0.3 | 99 |
| 7 | 37 | 76 | 0.9 | 0.4 | 0.4 | 1.6 | 0.1 | 89 |
| 8 | 37 | 67 | 1.4 | 0.4 | 0.4 | 1.4 | 0.1 | 73 |
| 9 | 38 | 68 | 0.4 | 0.4 | 0.4 | 1.4 | 0.2 | 98 |
| 10 | 40 | 133 | 1.9 | 0.5 | 0.5 | 1.7 | 0.1 | 10 |
| 11 | 40 | 149 | 2.5 | 0.5 | 0.5 | 1.4 | 0.1 | 30 |
| 12 | 39 | 125 | 1.9 | 0.5 | 0.5 | 1.4 | 0.1 | 96 |
| 13 | 40 | 134 | 1.8 | -0.6 | -0.5 | 1.7 | 0.1 | 10 |
| 14 | 40 | 150 | 2.6 | -0.6 | -0.6 | 1.4 | 0.1 | 30 |
| 15 | 39 | 124 | 2.4 | -0.6 | -0.6 | 1.4 | 0.2 | 97 |
| 16 | 39 | 137 | 6.2 | 0.3 | 0.3 | 1.4 | 0.1 | 25 |
| 17 | 40 | 120 | 5.5 | 0.3 | 0.3 | 1.3 | 0.2 | 98 |
| 18 | 35 | 70 | 3.7 | 0.6 | 0.6 | 1.6 | 0.1 | 92 |
| 19 | 35 | 89 | 3.9 | 0.6 | 0.6 | 1.4 | 0.1 | 35 |
| 20 | 37 | 75 | 4.5 | 0.6 | 0.5 | 1.4 | 0.3 | 100 |
| 21 | 37 | 73 | 4.1 | -0.6 | -0.6 | 1.6 | 0.1 | 87 |
| 22 | 37 | 80 | 3.8 | -0.6 | -0.6 | 1.4 | 0.1 | 52 |
| 23 | 38 | 74 | 4.5 | -0.6 | -0.6 | 1.3 | 0.3 | 99 |
| 24 | 38 | 76 | 4.5 | 0.4 | 0.4 | 1.6 | 0.1 | 85 |
| 25 | 38 | 79 | 4.2 | 0.4 | 0.4 | 1.4 | 0.1 | 55 |
| 26 | 39 | 74 | 5.8 | 0.3 | 0.3 | 1.3 | 0.3 | 98 |
|  | | | | | | | | |
|  | *Ae. geniculata* | | | | | | | |
|  | Bioclimatic component | | | Geophysic component | | Edaphic component | | |
| ELC category | Isothermality | Minimum temperature in the coldest month | Annual temperature range | Altitude | Slope | Topsoil sand fraction | Topsoil organic carbon | Topsoil gravel content |
| 1 | 40 | 2.3 | 21.3 | 585 | 11.4 | 45 | 2.6 | 7 |
| 2 | 40 | 2.5 | 21.8 | 569 | 13.6 | 39 | 1.9 | 18 |
| 3 | 39 | 3.2 | 21.9 | 564 | 12.9 | 40 | 0.9 | 7 |
| 4 | 40 | 3.5 | 20.3 | 437 | 3.2 | 44 | 3.0 | 7 |
| 5 | 39 | 4.6 | 23.1 | 277 | 2.8 | 39 | 1.4 | 18 |
| 6 | 38 | 4.9 | 21.9 | 240 | 2.3 | 34 | 0.8 | 6 |
| 7 | 39 | 6.6 | 19.6 | 181 | 3.2 | 53 | 2.4 | 6 |
| 8 | 40 | 6.2 | 24.5 | 221 | 1.6 | 44 | 1.0 | 21 |
| 9 | 40 | 6.9 | 23.0 | 245 | 2.6 | 32 | 0.8 | 6 |
| 10 | 37 | -2.7 | 25.5 | 1384 | 11.0 | 44 | 2.7 | 7 |
| 11 | 37 | -2.5 | 27.0 | 1352 | 10.5 | 45 | 1.7 | 19 |
| 12 | 38 | -1.8 | 27.9 | 1233 | 9.5 | 33 | 0.8 | 7 |
| 13 | 38 | -0.7 | 27.3 | 940 | 1.8 | 53 | 2.1 | 6 |
| 14 | 38 | -0.8 | 28.4 | 936 | 1.8 | 45 | 1.2 | 18 |
| 15 | 38 | -0.5 | 28.5 | 830 | 1.5 | 29 | 0.7 | 6 |
| 16 | 39 | 2.2 | 26.6 | 687 | 2.4 | 41 | 2.7 | 8 |
| 17 | 39 | 1.5 | 27.1 | 1176 | 4.4 | 38 | 2.0 | 18 |
| 18 | 39 | 1.2 | 29.0 | 1051 | 3.5 | 30 | 0.8 | 6 |
| 19 | 35 | 1.1 | 27.3 | 798 | 11.3 | 43 | 2.7 | 8 |
| 20 | 35 | 1.2 | 27.8 | 816 | 10.9 | 48 | 1.5 | 20 |
| 21 | 37 | 1.7 | 28.8 | 851 | 10.9 | 29 | 0.8 | 6 |
| 22 | 35 | 1.4 | 29.3 | 632 | 1.3 | 64 | 1.3 | 5 |
| 23 | 35 | 1.8 | 28.2 | 591 | 2.4 | 48 | 1.1 | 18 |
| 24 | 36 | 1.6 | 28.7 | 546 | 1.4 | 31 | 0.7 | 6 |
| 25 | 37 | 3.6 | 30.4 | 323 | 0.9 | 73 | 1.5 | 4 |
| 26 | 37 | 3.2 | 30.9 | 450 | 1.6 | 51 | 1.0 | 21 |
| 27 | 38 | 3.7 | 29.7 | 475 | 1.9 | 28 | 0.8 | 5 |
|  | | | | | | | | |
|  | *Ae. neglecta* | | | | | | | |
|  | Bioclimatic component | | | Geophysic component | | Edaphic component | | |
| ELC category | Isothermality | Precipitation in March | Maximum temperature  in May | Altitude | Northness | Topsoil total exchangeable bases | Reference depth of the soil unit | Topsoil salinity |
| 1 | 39 | 89 | 16.2 | 818 | -0.7 | 7 | 100 | 0.1 |
| 2 | 38 | 86 | 15.6 | 1043 | -0.6 | 13 | 26 | 0.1 |
| 3 | 38 | 75 | 16.9 | 991 | -0.6 | 34 | 100 | 0.4 |
| 4 | 40 | 113 | 19.1 | 394 | 0.0 | 7 | 100 | 0.1 |
| 5 | 39 | 118 | 18.6 | 417 | 0.0 | 10 | 28 | 0.1 |
| 6 | 39 | 110 | 21.2 | 414 | 0.0 | 39 | 100 | 0.2 |
| 7 | 39 | 89 | 16.2 | 818 | 0.7 | 7 | 100 | 0.1 |
| 8 | 38 | 86 | 15.5 | 1046 | 0.6 | 13 | 26 | 0.1 |
| 9 | 38 | 74 | 16.9 | 987 | 0.6 | 34 | 100 | 0.4 |
| 10 | 37 | 44 | 19.1 | 854 | -0.6 | 7 | 100 | 0.1 |
| 11 | 37 | 50 | 18.6 | 899 | -0.6 | 18 | 16 | 0.1 |
| 12 | 37 | 42 | 19.9 | 713 | -0.6 | 33 | 100 | 0.4 |
| 13 | 38 | 39 | 20.5 | 710 | 0.0 | 10 | 100 | 0.1 |
| 14 | 38 | 39 | 19.8 | 901 | 0.0 | 8 | 10 | 0.1 |
| 15 | 37 | 30 | 22.0 | 334 | 0.0 | 29 | 100 | 0.4 |
| 16 | 37 | 44 | 19.1 | 851 | 0.6 | 7 | 100 | 0.1 |
| 17 | 37 | 50 | 18.6 | 904 | 0.6 | 18 | 16 | 0.1 |
| 18 | 37 | 42 | 19.8 | 715 | 0.6 | 32 | 100 | 0.4 |
| 19 | 37 | 62 | 22.5 | 619 | -0.8 | 10 | 100 | 0.1 |
| 20 | 38 | 49 | 22.3 | 544 | -0.7 | 16 | 13 | 0.1 |
| 21 | 38 | 45 | 22.5 | 532 | -0.7 | 32 | 100 | 0.4 |
| 22 | 38 | 65 | 23.4 | 360 | 0.0 | 8 | 100 | 0.1 |
| 23 | 38 | 61 | 23.1 | 421 | 0.0 | 14 | 13 | 0.1 |
| 24 | 38 | 67 | 23.8 | 275 | 0.0 | 33 | 100 | 0.3 |
| 25 | 37 | 62 | 22.4 | 638 | 0.8 | 11 | 100 | 0.1 |
| 26 | 39 | 49 | 22.2 | 542 | 0.7 | 16 | 13 | 0.1 |
| 27 | 38 | 45 | 22.4 | 541 | 0.7 | 31 | 100 | 0.4 |
|  | | | | | | | | |
|  | *Ae. triuncialis* | | | | | | | |
|  | Bioclimatic component | | | Geophysic component | | Edaphic component | | |
| ELC category | Isothermality | Precipitation in April | Minimum temperature  in April | Altitude | Eastness | Topsoil clay fraction | Topsoil organic carbon | Topsoil base saturation |
| 1 | 35 | 56 | 8.3 | 334 | 0.8 | 21 | 1.9 | 26 |
| 2 | 37 | 45 | 8.0 | 392 | 0.7 | 20 | 0.8 | 97 |
| 3 | 37 | 46 | 8.3 | 378 | 0.7 | 49 | 0.9 | 100 |
| 4 | 36 | 56 | 8.3 | 339 | -0.8 | 21 | 2.0 | 26 |
| 5 | 37 | 45 | 8.0 | 393 | -0.7 | 20 | 0.8 | 96 |
| 6 | 37 | 47 | 8.3 | 381 | -0.7 | 49 | 0.9 | 100 |
| 7 | 38 | 54 | 8.9 | 362 | 0.0 | 21 | 1.8 | 24 |
| 8 | 39 | 48 | 9.4 | 342 | 0.0 | 17 | 0.9 | 90 |
| 9 | 38 | 53 | 9.9 | 282 | 0.0 | 52 | 0.9 | 99 |
| 10 | 37 | 54 | 3.5 | 992 | 0.6 | 22 | 2.3 | 25 |
| 11 | 37 | 50 | 4.6 | 876 | 0.6 | 20 | 0.9 | 96 |
| 12 | 38 | 49 | 4.7 | 827 | 0.6 | 49 | 0.9 | 100 |
| 13 | 37 | 54 | 3.5 | 988 | -0.6 | 22 | 2.3 | 25 |
| 14 | 37 | 50 | 4.6 | 877 | -0.6 | 20 | 0.9 | 96 |
| 15 | 38 | 49 | 4.7 | 827 | -0.6 | 49 | 0.9 | 100 |
| 16 | 37 | 59 | 6.5 | 689 | 0.0 | 21 | 1.6 | 24 |
| 17 | 37 | 53 | 6.1 | 1035 | 0.0 | 19 | 1.0 | 90 |
| 18 | 37 | 62 | 6.4 | 915 | 0.0 | 49 | 0.9 | 100 |
| 19 | 39 | 89 | 4.2 | 826 | 0.7 | 22 | 2.8 | 29 |
| 20 | 38 | 86 | 3.0 | 1044 | 0.6 | 20 | 1.5 | 97 |
| 21 | 38 | 74 | 3.6 | 883 | 0.6 | 49 | 0.9 | 100 |
| 22 | 39 | 89 | 4.2 | 826 | -0.7 | 22 | 2.8 | 29 |
| 23 | 38 | 86 | 3.1 | 1039 | -0.6 | 20 | 1.5 | 97 |
| 24 | 38 | 74 | 3.6 | 888 | -0.6 | 49 | 0.9 | 100 |
| 25 | 39 | 98 | 9.0 | 174 | 0.0 | 22 | 2.8 | 29 |
| 26 | 39 | 70 | 6.0 | 1033 | 0.0 | 20 | 1.7 | 99 |
| 27 | 39 | 70 | 6.1 | 1013 | 0.0 | 49 | 0.9 | 100 |
|  | | | | | | | | |
|  | *Ae. ventricosa* | | | | | | | |
|  | Bioclimatic component | | | Geophysic component | | Edaphic component | | |
| ELC category | Temperature seasonality | Precipitation in April | Mean temperature of the coldest quarter | Altitude | Slope | Topsoil reference bulk density | Topsoil base saturation | Topsoil sodicity |
| 1 | 5979 | 52 | 3.8 | 923 | 1.9 | 1.4 | 25 | 1.2 |
| 2 | 6193 | 47 | 4.9 | 787 | 1.7 | 1.4 | 95 | 2.8 |
| 3 | 6206 | 48 | 4.8 | 787 | 1.3 | 1.3 | 100 | 1.0 |
| 4 | 6304 | 52 | 7.9 | 426 | 1.4 | 1.4 | 24 | 1.0 |
| 5 | 6620 | 54 | 7.7 | 476 | 1.6 | 1.5 | 86 | 1.6 |
| 6 | 6457 | 57 | 8.2 | 552 | 2.0 | 1.3 | 100 | 1.0 |
| 7 | 6030 | 59 | 2.5 | 1243 | 9.9 | 1.4 | 26 | 1.3 |
| 8 | 6231 | 60 | 3.5 | 1181 | 9.2 | 1.4 | 95 | 2.1 |
| 9 | 6163 | 59 | 4.4 | 1065 | 9.5 | 1.3 | 100 | 1.0 |
| 10 | 5376 | 57 | 8.5 | 206 | 2.8 | 1.4 | 26 | 1.2 |
| 11 | 5384 | 44 | 9.4 | 241 | 2.6 | 1.4 | 99 | 2.8 |
| 12 | 5327 | 44 | 9.6 | 207 | 1.7 | 1.3 | 100 | 1.0 |
| 13 | 5162 | 55 | 10.1 | 310 | 2.7 | 1.4 | 24 | 1.1 |
| 14 | 5089 | 39 | 11.1 | 241 | 2.0 | 1.5 | 89 | 2.0 |
| 15 | 5288 | 48 | 10.6 | 256 | 1.9 | 1.3 | 99 | 1.0 |
| 16 | 4772 | 54 | 8.9 | 463 | 14.9 | 1.4 | 24 | 1.4 |
| 17 | 5407 | 50 | 8.6 | 543 | 12.4 | 1.4 | 98 | 2.6 |
| 18 | 5373 | 47 | 8.6 | 678 | 13.5 | 1.3 | 99 | 1.0 |
| 19 | 4427 | 89 | 6.6 | 558 | 3.2 | 1.4 | 28 | 2.0 |
| 20 | 4748 | 85 | 5.7 | 600 | 3.4 | 1.4 | 96 | 2.1 |
| 21 | 5246 | 75 | 4.3 | 745 | 2.7 | 1.3 | 96 | 1.2 |
| 22 | 3773 | 94 | 9.4 | 170 | 3.6 | 1.4 | 29 | 2.1 |
| 23 | 5714 | 75 | 6.1 | 1110 | 3.4 | 1.4 | 99 | 2.4 |
| 24 | 3703 | 81 | 8.8 | 409 | 2.0 | 1.3 | 67 | 1.9 |
| 25 | 5083 | 89 | 2.5 | 1207 | 11.5 | 1.4 | 30 | 2.3 |
| 26 | 5253 | 89 | 2.0 | 1273 | 12.5 | 1.4 | 97 | 2.1 |
| 27 | 5635 | 79 | 1.8 | 1229 | 10.0 | 1.3 | 99 | 1.1 |
