# Supplementary figures and images for: Identification of ecogeographical gaps in the Spanish Aegilops collections with potential tolerance to drought and salinity
Source: PeerJ. 2017 Jul 27;5:e3494. doi: 10.7717/peerj.3494 (PMC5534164; doi:10.7717/peerj.3494)

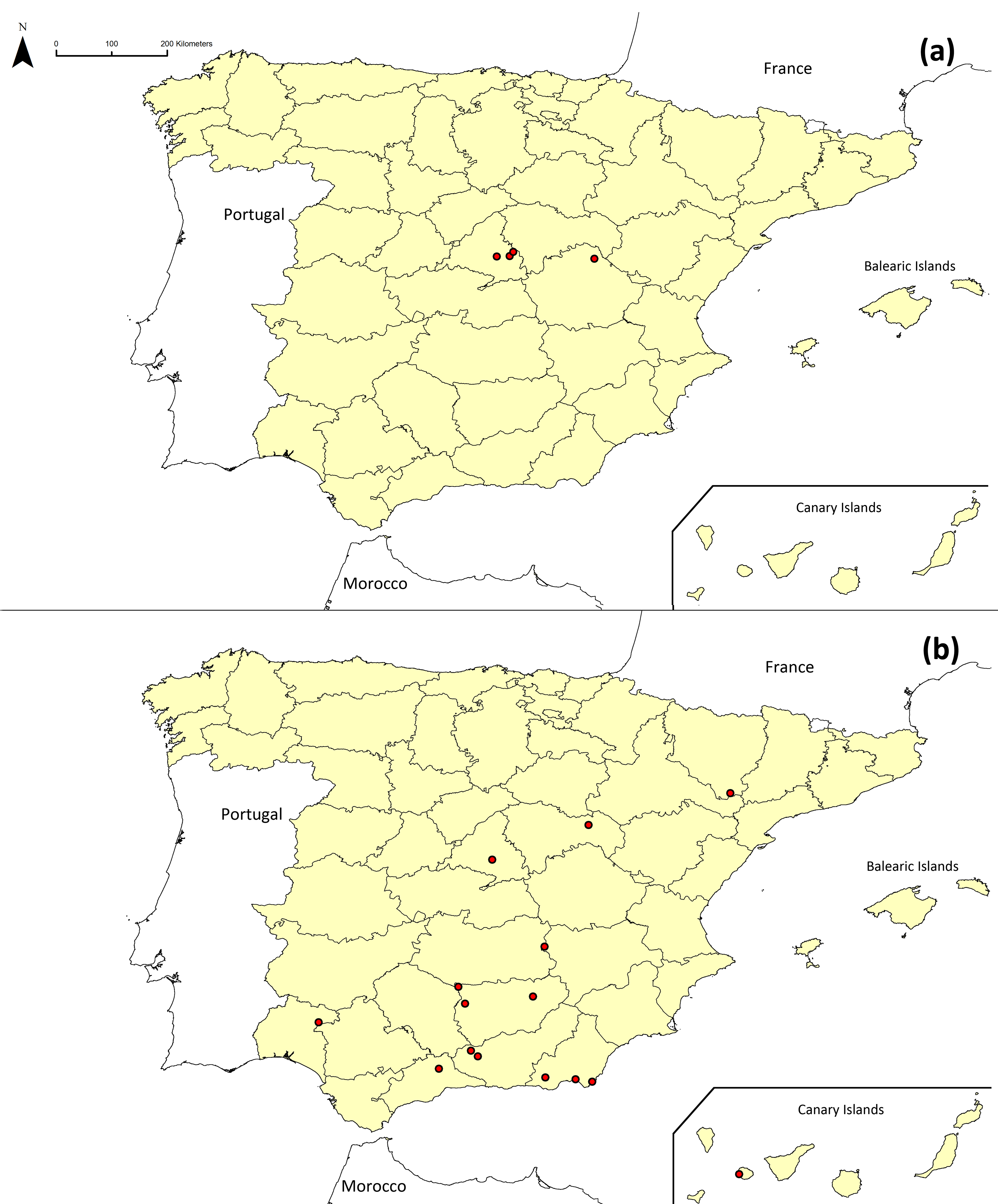

Supplement: Figure S1 [file peerj-05-3494-s003.png]

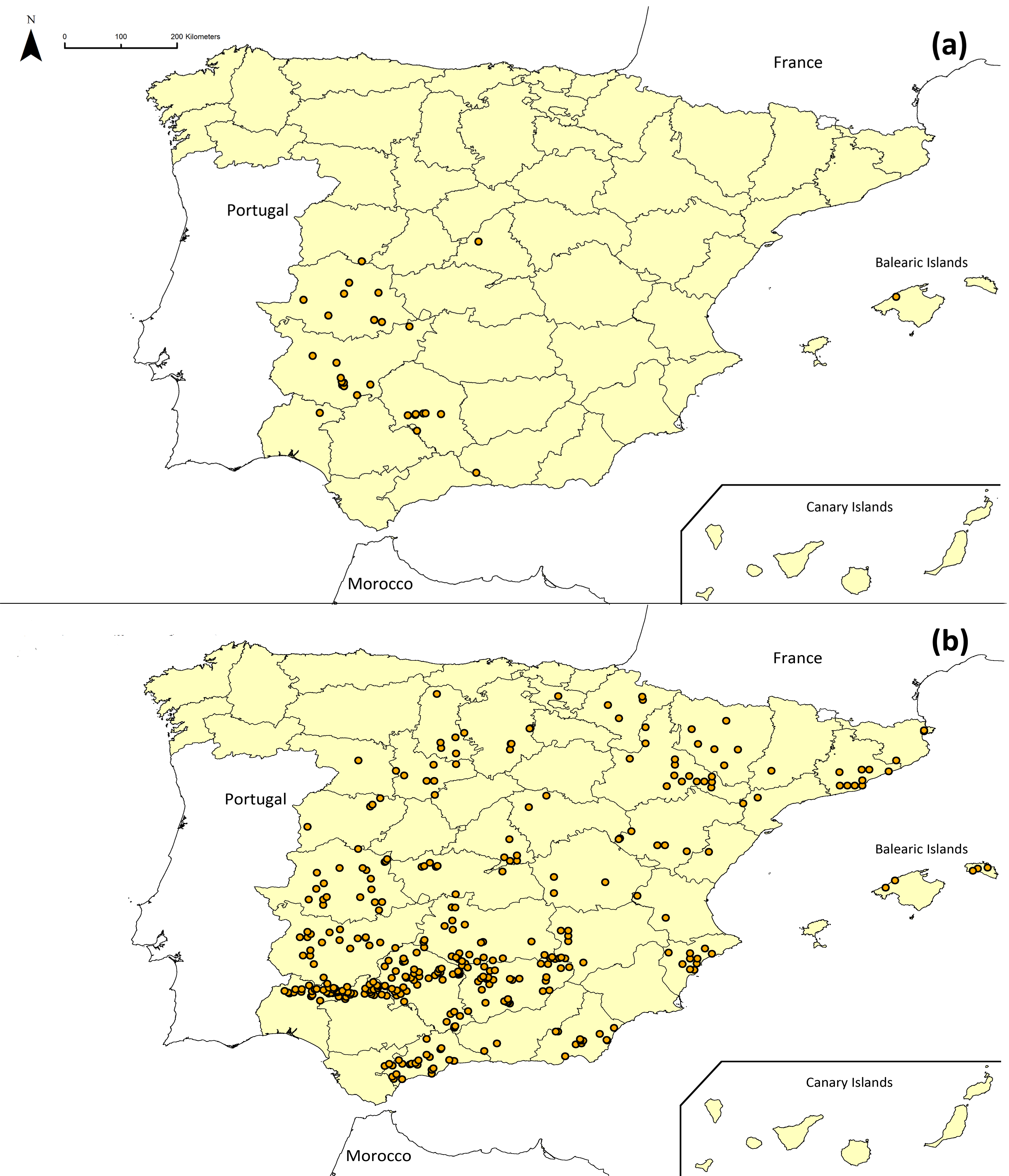

Supplement: Figure S2 [file peerj-05-3494-s004.png]

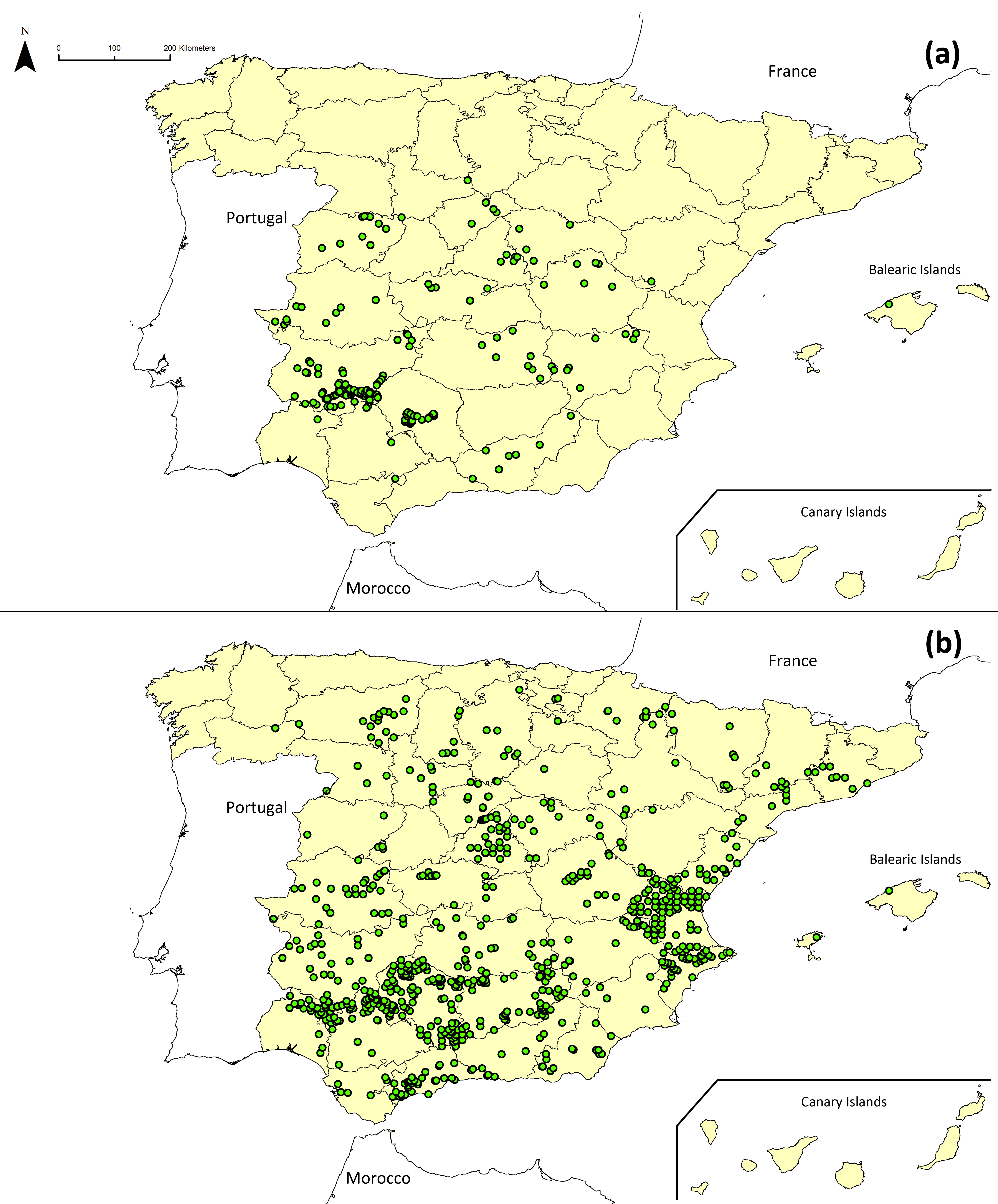

Supplement: Figure S3 [file peerj-05-3494-s005.png]

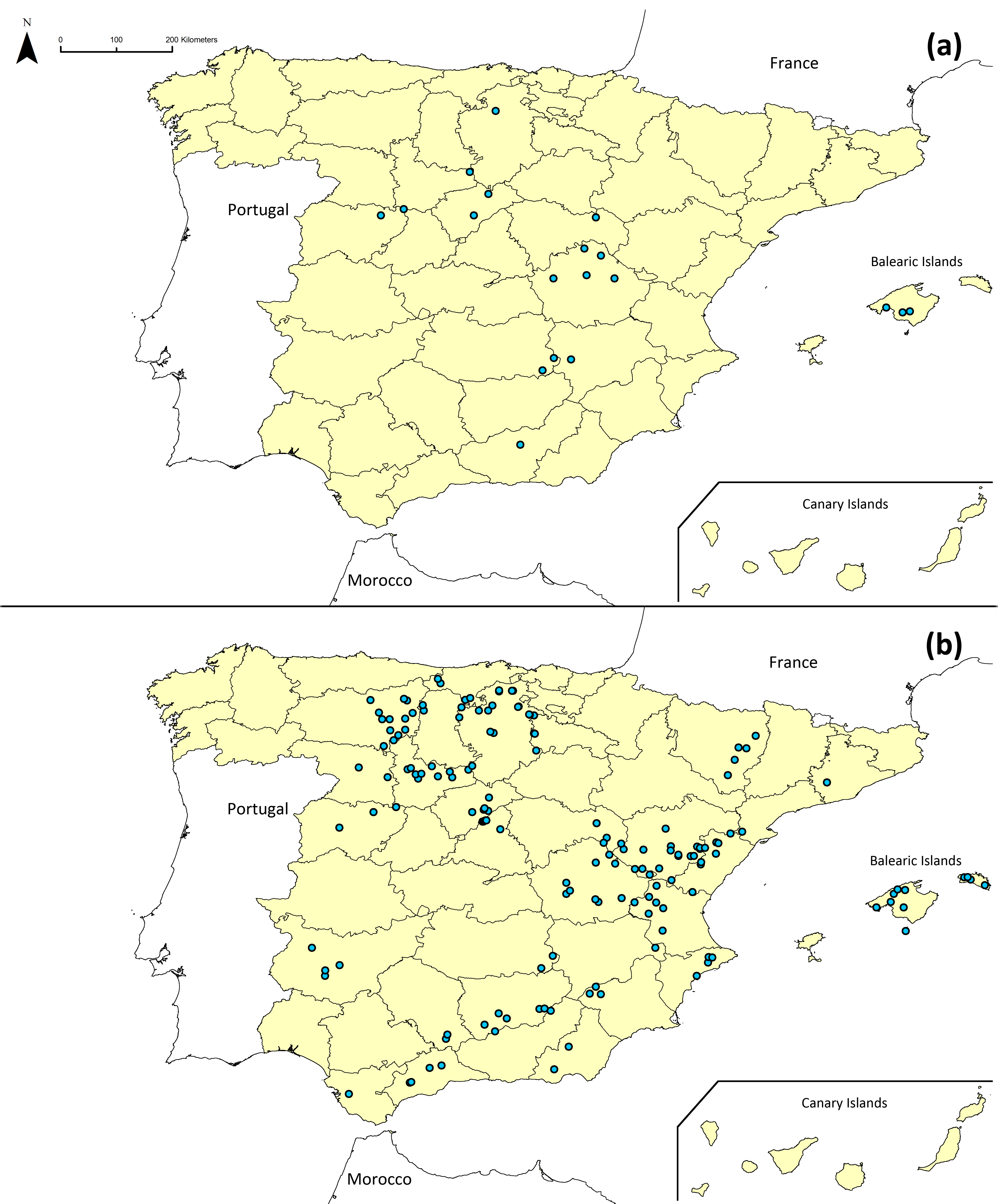

Supplement: Figure S4 [file peerj-05-3494-s006.png]

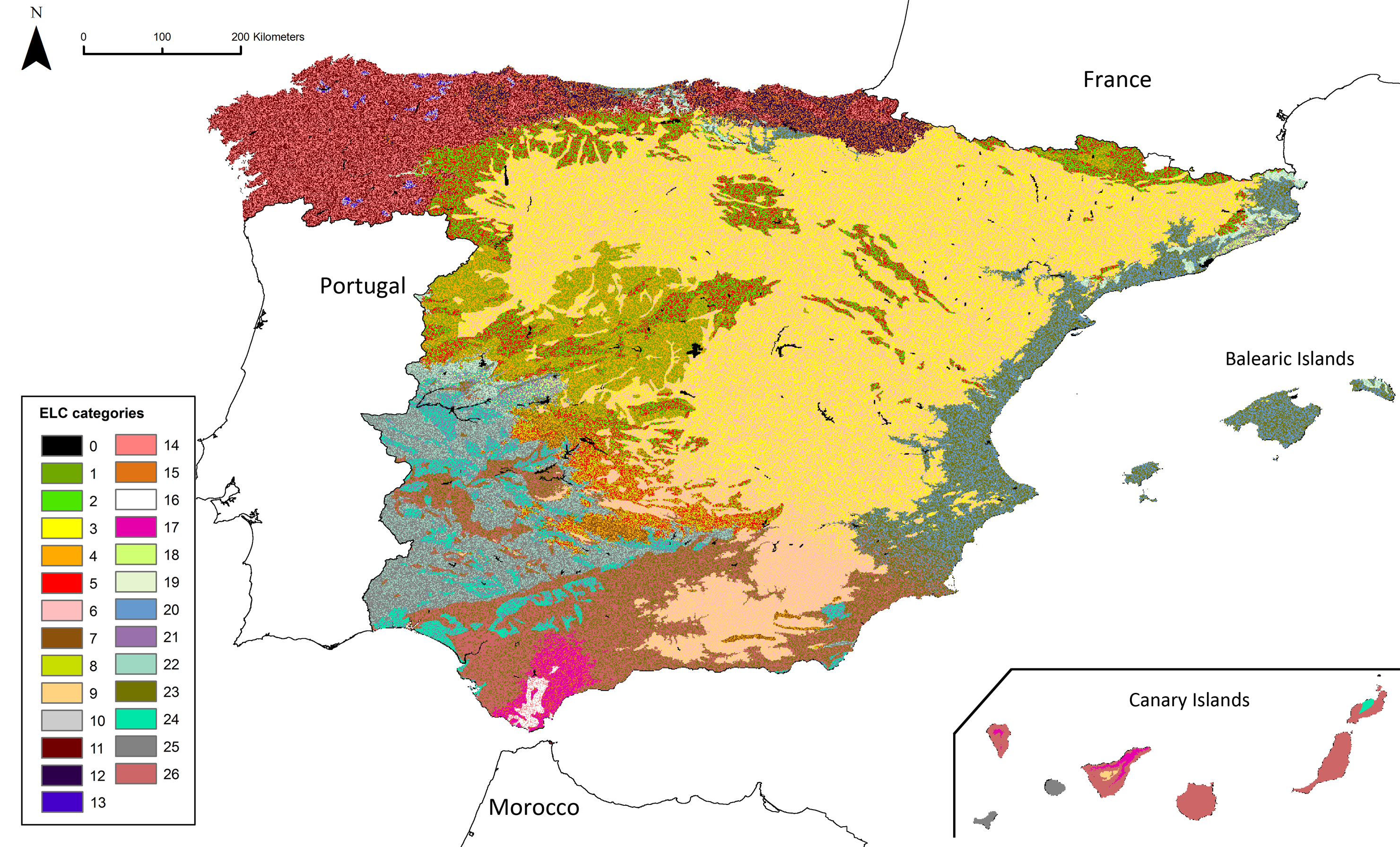

Supplement: Figure S5 — The environmental characteristics of the different categories are described in Table S10. [file peerj-05-3494-s007.png]

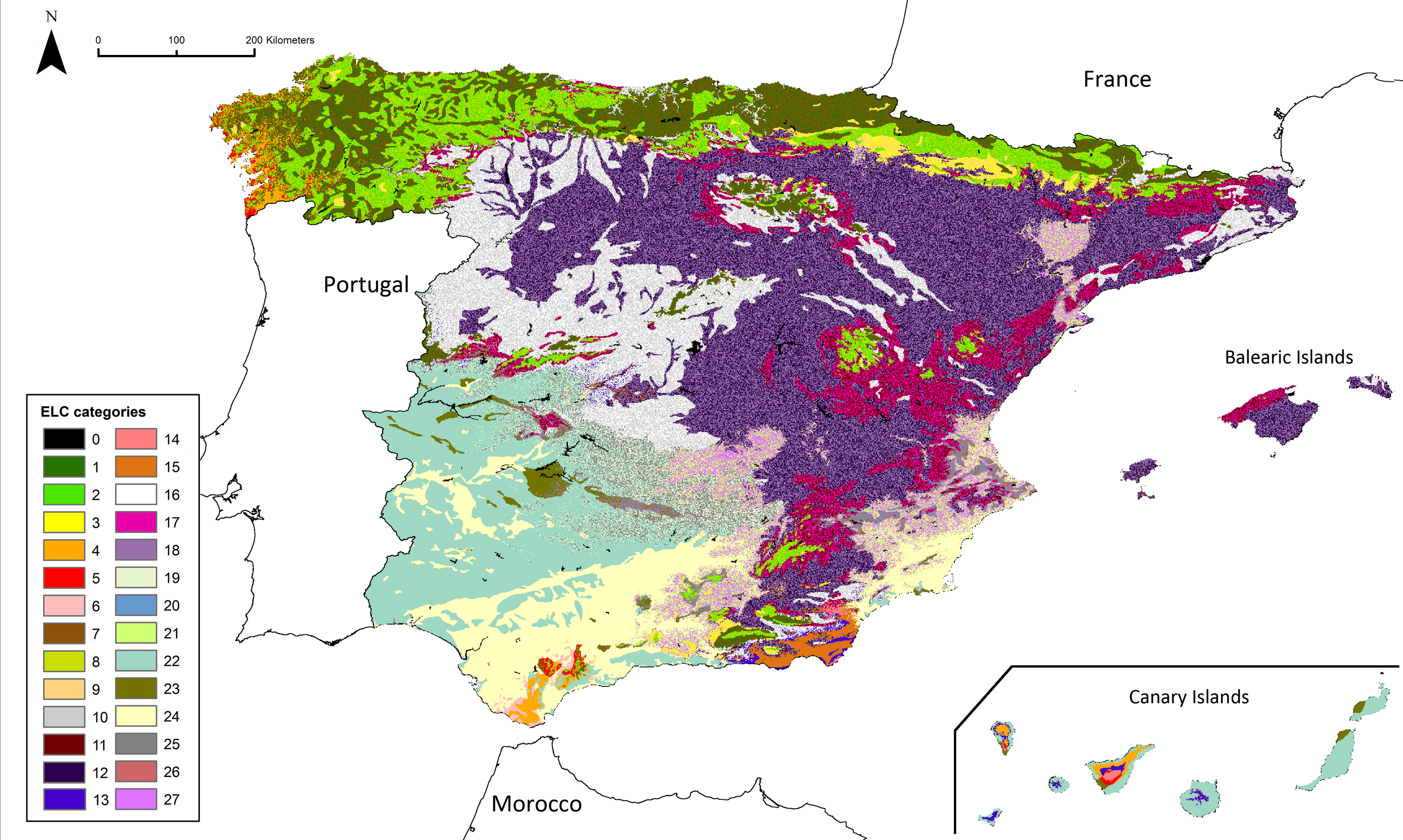

Supplement: Figure S6 — The environmental characteristics of the different categories are described in Table S10. [file peerj-05-3494-s008.png]

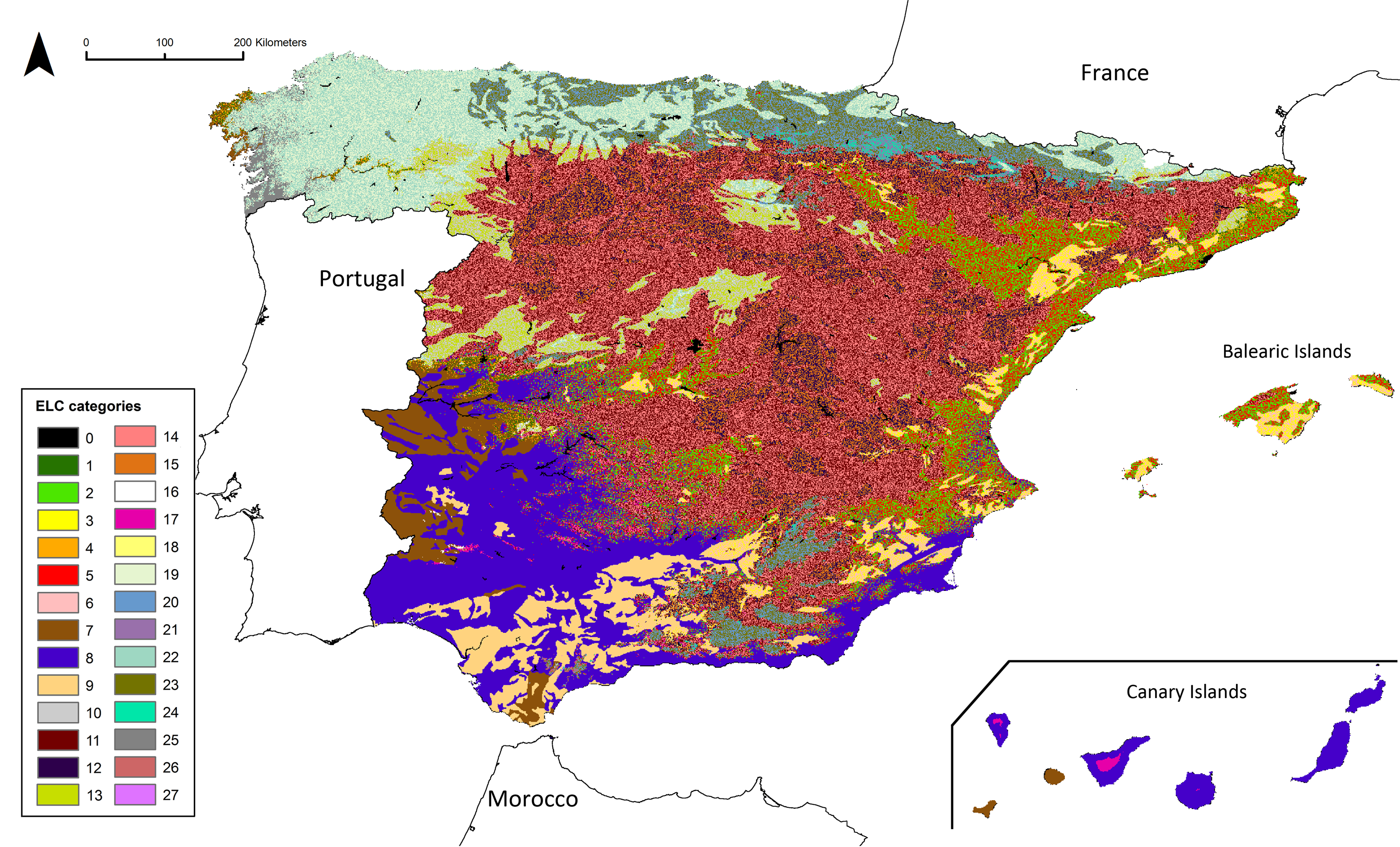

Supplement: Figure S7 — The environmental characteristics of the different categories are described in Table S10. [file peerj-05-3494-s009.png]

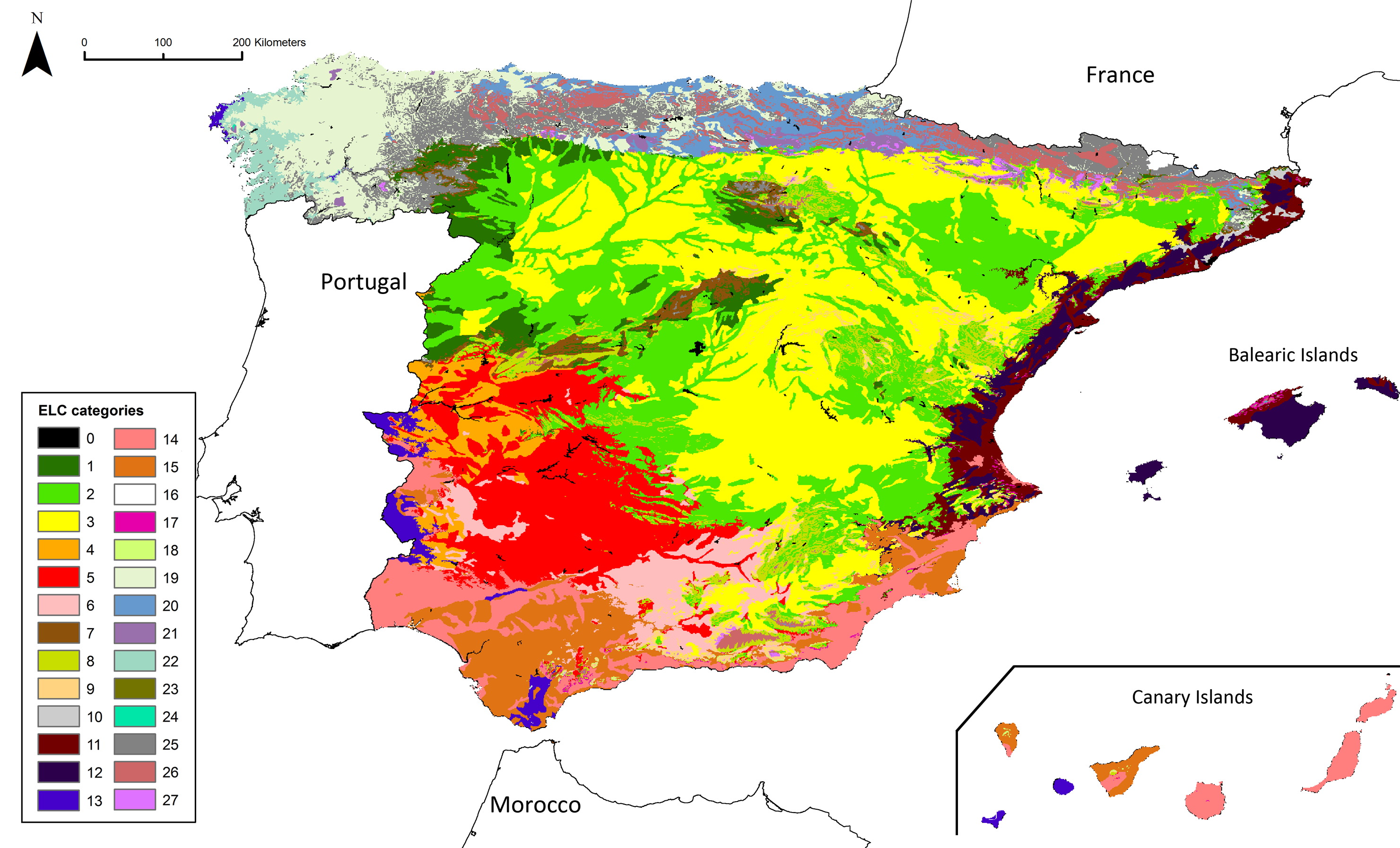

Supplement: Figure S8 — The environmental characteristics of the different categories are described in Table S10. [file peerj-05-3494-s010.png]

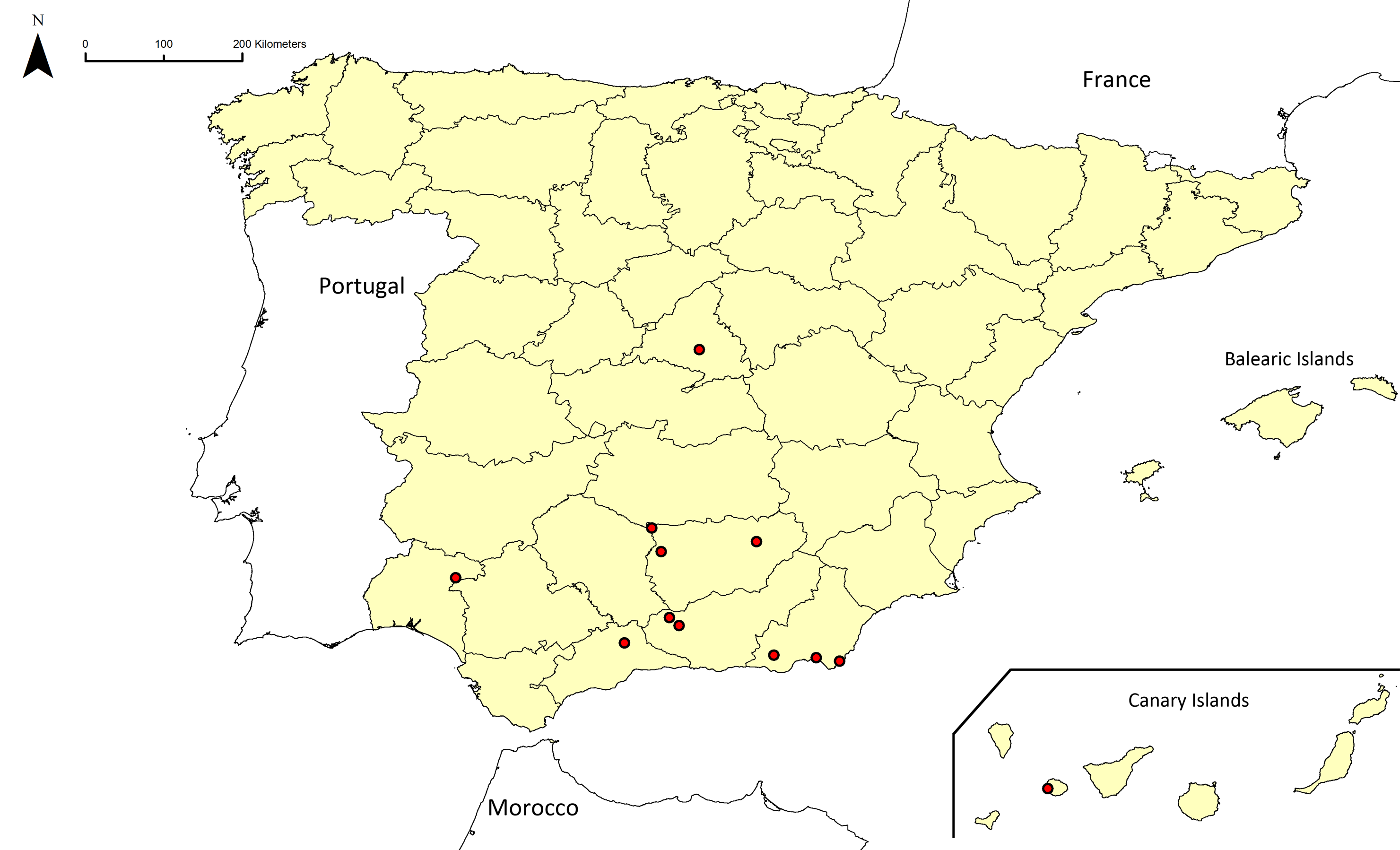

Supplement: Figure S9 [file peerj-05-3494-s011.png]

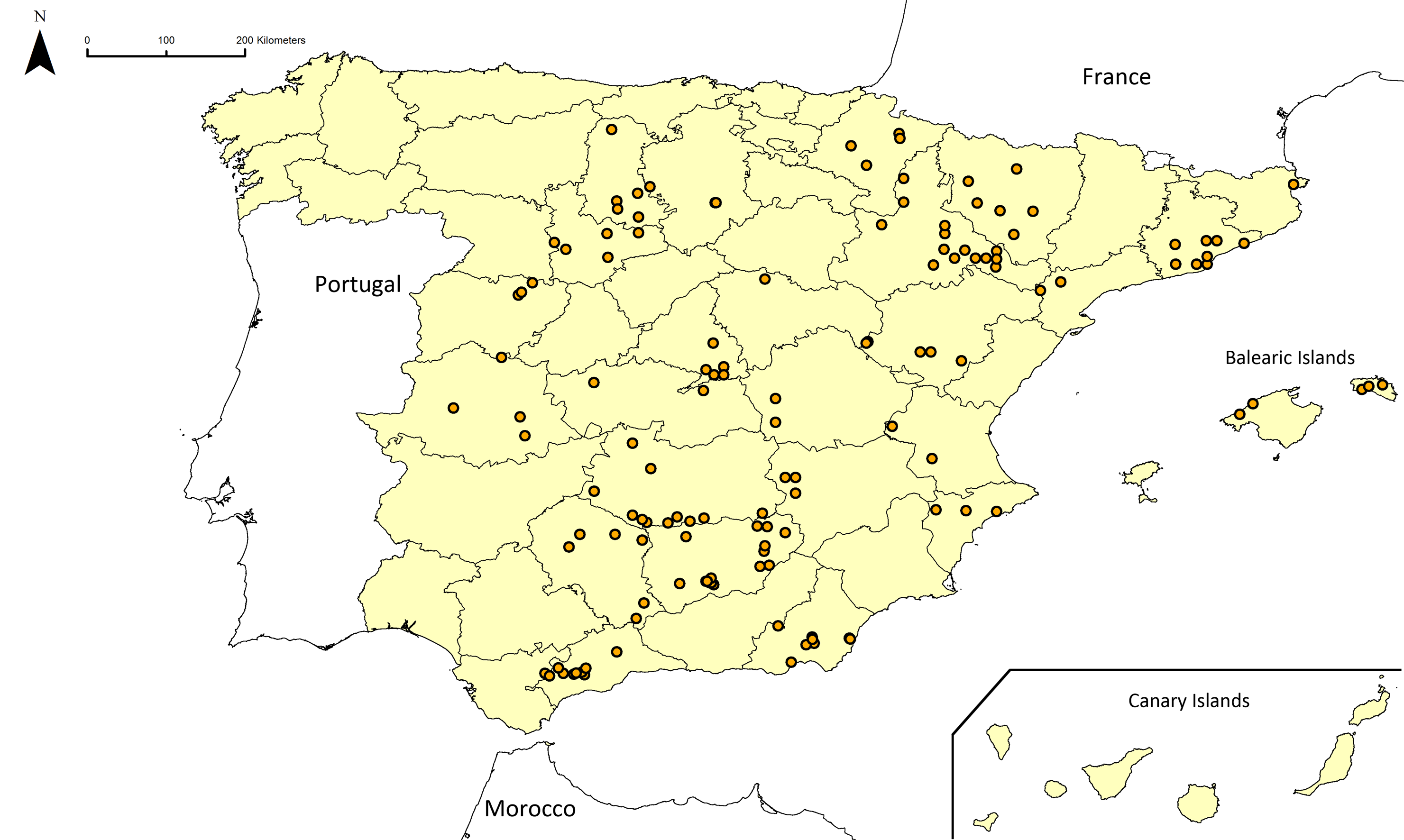

Supplement: Figure S10 [file peerj-05-3494-s012.png]

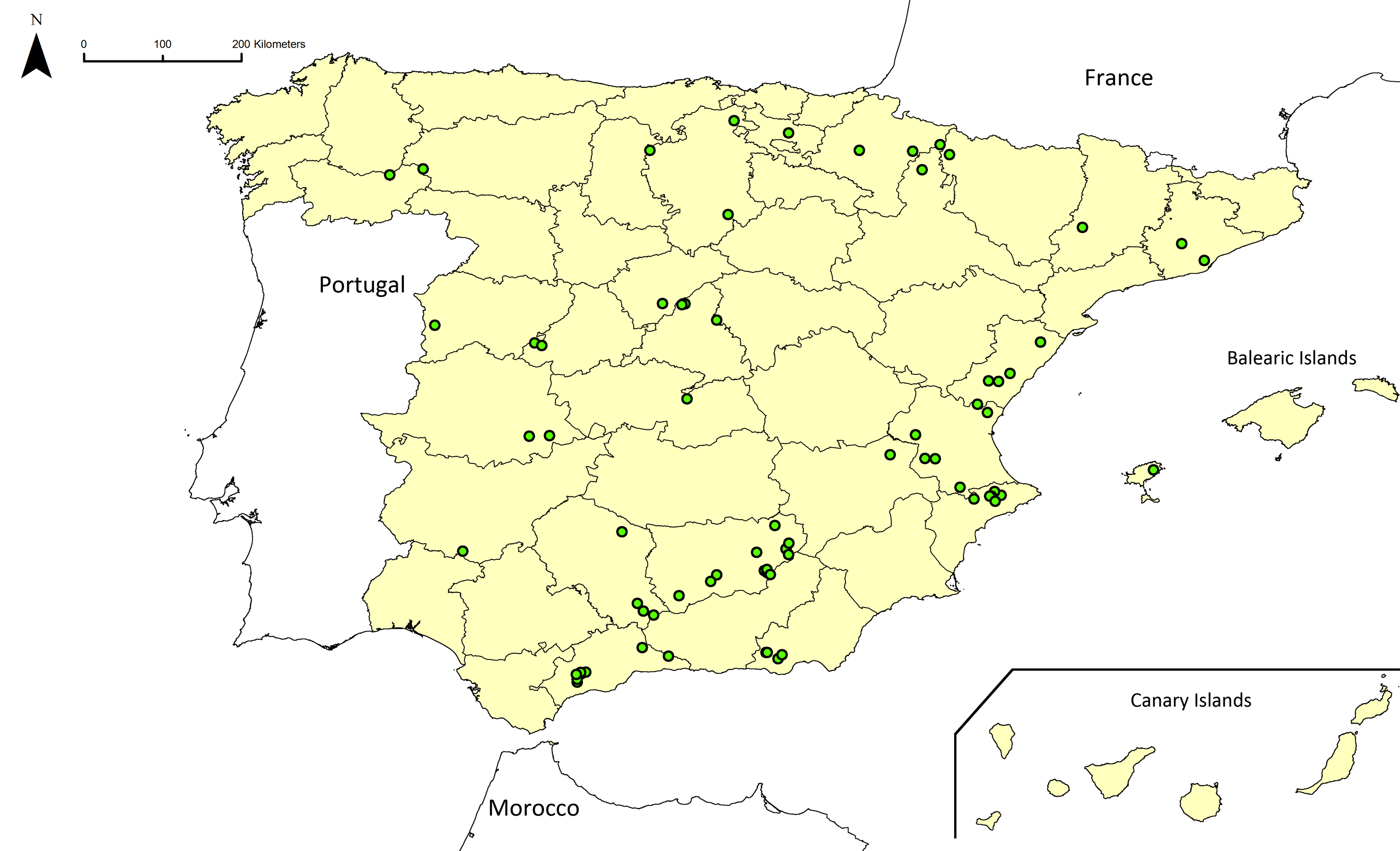

Supplement: Figure S11 [file peerj-05-3494-s013.png]

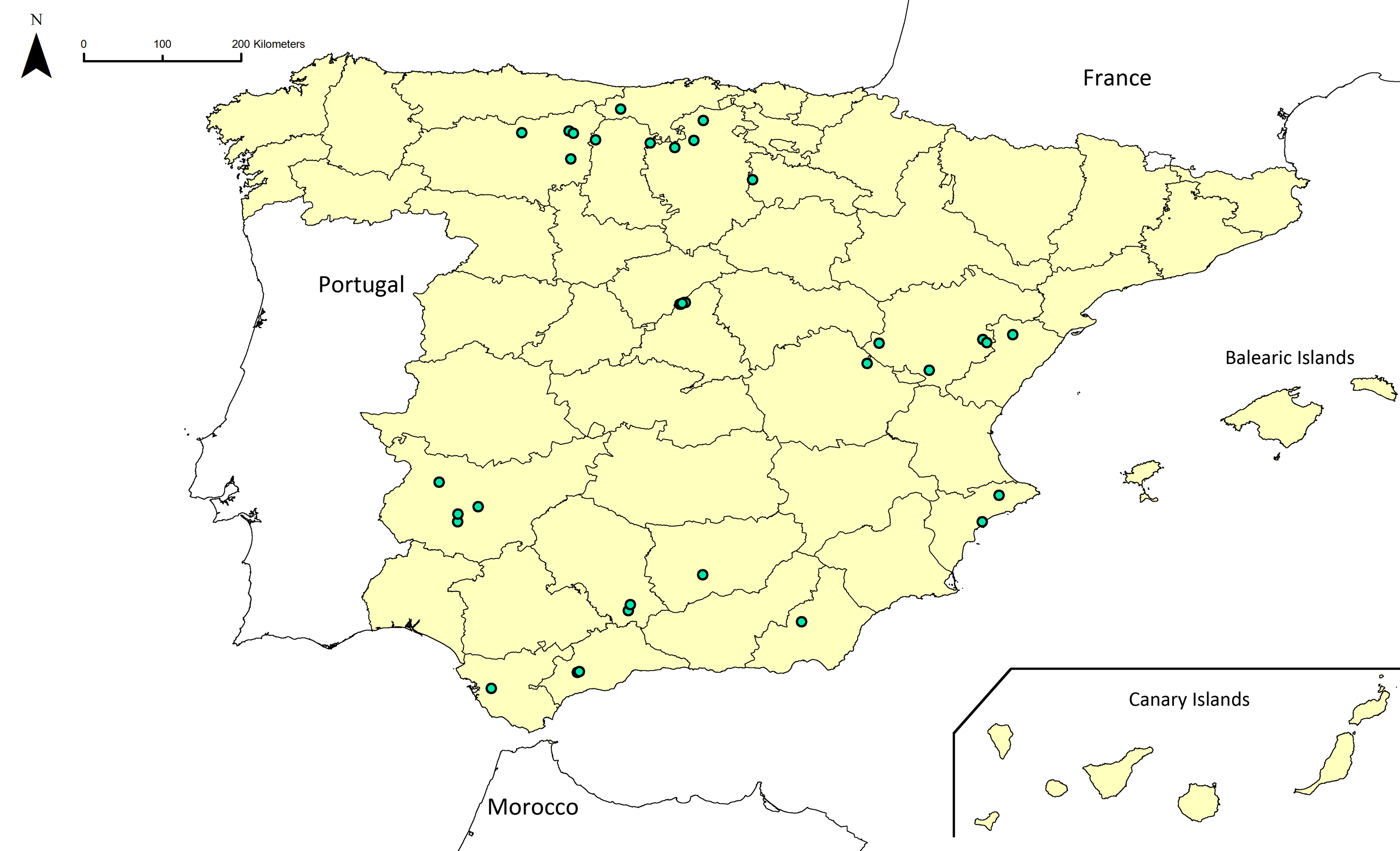

Supplement: Figure S12 [file peerj-05-3494-s014.png]
