# Supplementary material for: Identification of ecogeographical gaps in the Spanish Aegilops collections with potential tolerance to drought and salinity
Source: PeerJ. 2017 Jul 27;5:e3494. doi: 10.7717/peerj.3494 (PMC5534164; doi:10.7717/peerj.3494)
Supplement: Supplemental Information 1 [file peerj-05-3494-s015.pdf]

NUMCAT	ESTADO	NOMBAN	NUMBAN	FECADQ	FAMILIA	GENERO	ESPECI	ESPECI AUTOR	SUBTAX	SUBTAX AUTOR	NOMCOM	NOMLOC	INSREC
NC010107	C	ESP004	BGE005516	198306--	Poaceae	Aegilops	geniculata	Roth			Trigo bastardo		ESP004
NC010108	C	ESP004	BGE005517	198306--	Poaceae	Aegilops	triuncialis	L.			Rompesacos		ESP004
NC010109	C	ESP004	BGE005518	198306--	Poaceae	Aegilops	ventricosa	Tausch			Espiguilla ventruda		ESP004
NC011029	E				Poaceae	Aegilops	geniculata	Roth			Trigo bastardo		ESP080
NC015807	E				Poaceae	Aegilops	triuncialis	L.			Rompesacos	Rompesacos	ESP080
NC015808	E				Poaceae	Aegilops	geniculata	Roth			Trigo bastardo		ESP080
NC015809	E				Poaceae	Aegilops	geniculata	Roth			Trigo bastardo	Rompesacos	ESP080
NC015811	E				Poaceae	Aegilops	triuncialis	L.			Rompesacos	Rompesacos	ESP080
NC015812	E				Poaceae	Aegilops	ventricosa	Tausch			Espiguilla ventruda		ESP080
NC015813	E				Poaceae	Aegilops	geniculata	Roth			Trigo bastardo		ESP080
NC015815	E				Poaceae	Aegilops	triuncialis	L.			Rompesacos	Rompesacos	ESP080
NC015825	E				Poaceae	Aegilops	triuncialis	L.			Rompesacos		ESP080
NC015826	E				Poaceae	Aegilops	geniculata	Roth			Trigo bastardo		ESP080
NC015827	E				Poaceae	Aegilops	triuncialis	L.			Rompesacos		ESP080
NC022308	C	ESP004	BGE022733	1994----	Poaceae	Aegilops	geniculata	Roth			Trigo bastardo		ESP004
NC022311	C	ESP004	BGE021875	1993----	Poaceae	Aegilops	triuncialis	L.			Rompesacos		ESP004
NC022312	C	ESP004	BGE022734	1994----	Poaceae	Aegilops	geniculata	Roth			Trigo bastardo		ESP004
NC022338	C	ESP004	BGE019422	1992----	Poaceae	Aegilops	geniculata	Roth			Trigo bastardo		ESP004
NC022339	C	ESP004	BGE019420	1992----	Poaceae	Aegilops	neglecta	Req. ex Bertol.					ESP004
NC022340	C	ESP004	BGE019424	1992----	Poaceae	Aegilops	triuncialis	L.			Rompesacos		ESP004
NC022349	C	ESP004	BGE019421	1992----	Poaceae	Aegilops	neglecta	Req. ex Bertol.					ESP004
NC022350	C	ESP004	BGE019423	1992----	Poaceae	Aegilops	geniculata	Roth			Trigo bastardo		ESP004
NC024016	E				Poaceae	Aegilops	ventricosa	Tausch			Espiguilla ventruda		ESP004
NC024018	E				Poaceae	Aegilops	geniculata	Roth			Trigo bastardo		ESP004
NC024023	E				Poaceae	Aegilops	ventricosa	Tausch			Espiguilla ventruda		ESP004
NC024038	C	ESP004	BGE021895	1993----	Poaceae	Aegilops	ventricosa	Tausch			Espiguilla ventruda		ESP004
NC024039	C	ESP004	BGE021876	1993----	Poaceae	Aegilops	triuncialis	L.			Rompesacos	Rompesacos	ESP004
NC024042	C	ESP004	BGE022735	1994----	Poaceae	Aegilops	geniculata	Roth			Trigo bastardo		ESP004
NC024050	C	ESP004	BGE021877	1993----	Poaceae	Aegilops	triuncialis	L.			Rompesacos		ESP004
NC024051	C	ESP004	BGE022736	1994----	Poaceae	Aegilops	geniculata	Roth			Trigo bastardo		ESP004
NC024072	C	ESP004	BGE022737	1994----	Poaceae	Aegilops	geniculata	Roth			Trigo bastardo	Rompesacos	ESP004

NC027317	E				Poaceae	Aegilops	triuncialis	L.			Rompesacos		ESP004
NC027318	E				Poaceae	Aegilops	geniculata	Roth			Trigo bastardo		ESP004
NC027323	E				Poaceae	Aegilops	geniculata	Roth			Trigo bastardo		ESP004
NC027324	E				Poaceae	Aegilops	triuncialis	L.			Rompesacos		ESP004
NC027325	E				Poaceae	Aegilops	geniculata	Roth			Trigo bastardo		ESP004
NC027326	E				Poaceae	Aegilops	triuncialis	L.			Rompesacos		ESP004
NC027327	E				Poaceae	Aegilops	geniculata	Roth			Trigo bastardo		ESP004
NC027331	E				Poaceae	Aegilops	geniculata	Roth			Trigo bastardo		ESP004
NC027332	E				Poaceae	Aegilops	triuncialis	L.			Rompesacos		ESP004
NC027334	E				Poaceae	Aegilops	geniculata	Roth			Trigo bastardo		ESP004
NC027335	E				Poaceae	Aegilops	triuncialis	L.			Rompesacos		ESP004
NC027336	E				Poaceae	Aegilops	geniculata	Roth			Trigo bastardo		ESP004
NC027337	E				Poaceae	Aegilops	geniculata	Roth			Trigo bastardo		ESP004
NC027338	E				Poaceae	Aegilops	geniculata	Roth			Trigo bastardo		ESP004
NC027339	E				Poaceae	Aegilops	geniculata	Roth			Trigo bastardo		ESP004
NC027341	E				Poaceae	Aegilops	sp.						ESP004
NC027342	E				Poaceae	Aegilops	geniculata	Roth			Trigo bastardo		ESP004
NC027343	E				Poaceae	Aegilops	geniculata	Roth			Trigo bastardo		ESP004
NC027344	E				Poaceae	Aegilops	geniculata	Roth			Trigo bastardo		ESP004
NC027345	E				Poaceae	Aegilops	biuncialis	Vis.					ESP004
NC027346	E				Poaceae	Aegilops	biuncialis	Vis.					ESP004
NC027349	E				Poaceae	Aegilops	geniculata	Roth			Trigo bastardo		ESP004
NC027350	E				Poaceae	Aegilops	biuncialis	Vis.					ESP004
NC027351	E				Poaceae	Aegilops	biuncialis	Vis.					ESP004
NC027355	E				Poaceae	Aegilops	geniculata	Roth			Trigo bastardo		ESP004
NC027356	E				Poaceae	Aegilops	geniculata	Roth			Trigo bastardo		ESP004
NC027360	E				Poaceae	Aegilops	triuncialis	L.			Rompesacos		ESP004
NC027362	C	ESP004	BGE022738	1994----	Poaceae	Aegilops	geniculata	Roth			Trigo bastardo		ESP004
NC027366	E				Poaceae	Aegilops	geniculata	Roth			Trigo bastardo		ESP004
NC027367	E				Poaceae	Aegilops	triuncialis	L.			Rompesacos		ESP004
NC027369	E				Poaceae	Aegilops	geniculata	Roth			Trigo bastardo		ESP004
NC027370	E				Poaceae	Aegilops	triuncialis	L.			Rompesacos		ESP004
NC027371	E				Poaceae	Aegilops	neglecta	Req. ex Bertol.					ESP004

NC027373	E				Poaceae	Aegilops	geniculata	Roth			Trigo bastardo		ESP004
NC027374	E				Poaceae	Aegilops	triuncialis	L.			Rompesacos		ESP004
NC027376	E				Poaceae	Aegilops	neglecta	Req. ex Bertol.					ESP004
NC027377	E				Poaceae	Aegilops	neglecta	Req. ex Bertol.					ESP004
NC027378	E				Poaceae	Aegilops	geniculata	Roth			Trigo bastardo		ESP004
NC027379	E				Poaceae	Aegilops	triuncialis	L.			Rompesacos		ESP004
NC027380	E				Poaceae	Aegilops	neglecta	Req. ex Bertol.					ESP004
NC027384	E				Poaceae	Aegilops	geniculata	Roth			Trigo bastardo		ESP004
NC027385	E				Poaceae	Aegilops	triuncialis	L.			Rompesacos		ESP004
NC027388	E				Poaceae	Aegilops	geniculata	Roth			Trigo bastardo		ESP004
NC027389	E				Poaceae	Aegilops	triuncialis	L.			Rompesacos		ESP004
NC027390	E				Poaceae	Aegilops	geniculata	Roth			Trigo bastardo		ESP004
NC027393	E				Poaceae	Aegilops	geniculata	Roth			Trigo bastardo		ESP004
NC027394	E				Poaceae	Aegilops	triuncialis	L.			Rompesacos		ESP004
NC027395	E				Poaceae	Aegilops	geniculata	Roth			Trigo bastardo		ESP004
NC027396	E				Poaceae	Aegilops	geniculata	Roth			Trigo bastardo		ESP004
NC027397	E				Poaceae	Aegilops	triuncialis	L.			Rompesacos		ESP004
NC027398	E				Poaceae	Aegilops	neglecta	Req. ex Bertol.					ESP004
NC027401	E				Poaceae	Aegilops	geniculata	Roth			Trigo bastardo		ESP004
NC027402	E				Poaceae	Aegilops	triuncialis	L.			Rompesacos		ESP004
NC027403	E				Poaceae	Aegilops	neglecta	Req. ex Bertol.					ESP004
NC027405	E				Poaceae	Aegilops	geniculata	Roth			Trigo bastardo		ESP004
NC027407	E				Poaceae	Aegilops	triuncialis	L.			Rompesacos		ESP004
NC027409	E				Poaceae	Aegilops	geniculata	Roth			Trigo bastardo		ESP004
NC027410	E				Poaceae	Aegilops	triuncialis	L.			Rompesacos		ESP004
NC027412	E				Poaceae	Aegilops	geniculata	Roth			Trigo bastardo		ESP004
NC027413	E				Poaceae	Aegilops	triuncialis	L.			Rompesacos		ESP004
NC027414	E				Poaceae	Aegilops	ventricosa	Tausch			Espiguilla ventruda		ESP004
NC027416	E				Poaceae	Aegilops	geniculata	Roth			Trigo bastardo		ESP004
NC027417	C	ESP004	BGE021878	1993----	Poaceae	Aegilops	triuncialis	L.			Rompesacos		ESP004
NC027418	E				Poaceae	Aegilops	geniculata	Roth			Trigo bastardo		ESP004
NC027421	C	ESP004	BGE022193	1993----	Poaceae	Aegilops	geniculata	Roth			Trigo bastardo		ESP004
NC027422	C	ESP004	BGE021896	1993----	Poaceae	Aegilops	ventricosa	Tausch			Espiguilla ventruda		ESP004

NC027423	E				Poaceae	Aegilops	triuncialis	L.			Rompesacos		ESP004
NC027424	E				Poaceae	Aegilops	ventricosa	Tausch			Espiguilla ventruda		ESP004
NC027426	C	ESP004	BGE022739	1994----	Poaceae	Aegilops	geniculata	Roth			Trigo bastardo		ESP004
NC027428	C	ESP004	BGE022751	1994----	Poaceae	Aegilops	geniculata	Roth			Trigo bastardo		ESP004
NC027431	E				Poaceae	Aegilops	geniculata	Roth			Trigo bastardo		ESP004
NC027433	E				Poaceae	Aegilops	triuncialis	L.			Rompesacos		ESP004
NC027434	C	ESP004	BGE022740	1994----	Poaceae	Aegilops	geniculata	Roth			Trigo bastardo		ESP004
NC027436	C	ESP004	BGE022194	1993----	Poaceae	Aegilops	geniculata	Roth			Trigo bastardo		ESP004
NC027446	E				Poaceae	Aegilops	geniculata	Roth			Trigo bastardo		ESP004
NC027447	C	ESP004	BGE021879	1993----	Poaceae	Aegilops	triuncialis	L.			Rompesacos		ESP004
NC027448	C	ESP004	BGE022195	1993----	Poaceae	Aegilops	geniculata	Roth			Trigo bastardo		ESP004
NC027450	C	ESP004	BGE021897	1993----	Poaceae	Aegilops	ventricosa	Tausch			Espiguilla ventruda		ESP004
NC027452	E				Poaceae	Aegilops	geniculata	Roth			Trigo bastardo		ESP004
NC027454	C	ESP004	BGE022741	1994----	Poaceae	Aegilops	geniculata	Roth			Trigo bastardo		ESP004
NC027457	E				Poaceae	Aegilops	triuncialis	L.			Rompesacos		ESP004
NC027459	C	ESP004	BGE021880	1993----	Poaceae	Aegilops	triuncialis	L.			Rompesacos		ESP004
NC027460	E				Poaceae	Aegilops	geniculata	Roth			Trigo bastardo		ESP004
NC027462	C	ESP004	BGE021898	1993----	Poaceae	Aegilops	ventricosa	Tausch			Espiguilla ventruda		ESP004
NC027463	C	ESP004	BGE022196	1993----	Poaceae	Aegilops	geniculata	Roth			Trigo bastardo		ESP004
NC043467	C	ESP004	BGE021847	1993----	Poaceae	Aegilops	neglecta	Req. ex Bertol.					ESP043
NC043468	C	ESP004	BGE021848	1993----	Poaceae	Aegilops	neglecta	Req. ex Bertol.					ESP004
NC043469	C	ESP004	BGE021849	1993----	Poaceae	Aegilops	neglecta	Req. ex Bertol.					ESP004
NC043470	C	ESP004	BGE021850	1993----	Poaceae	Aegilops	neglecta	Req. ex Bertol.					ESP004
NC043471	C	ESP004	BGE021851	1993----	Poaceae	Aegilops	neglecta	Req. ex Bertol.					ESP004
NC043472	C	ESP004	BGE021852	1993----	Poaceae	Aegilops	neglecta	Req. ex Bertol.					ESP004
NC043473	C	ESP004	BGE021853	1993----	Poaceae	Aegilops	neglecta	Req. ex Bertol.					ESP004
NC043485	C	ESP004	BGE021881	1993----	Poaceae	Aegilops	triuncialis	L.			Rompesacos		ESP004
NC043486	C	ESP004	BGE021882	1993----	Poaceae	Aegilops	triuncialis	L.			Rompesacos		ESP004
NC043487	C	ESP004	BGE021883	1993----	Poaceae	Aegilops	triuncialis	L.			Rompesacos		ESP004
NC043488	C	ESP004	BGE021884	1993----	Poaceae	Aegilops	triuncialis	L.			Rompesacos		ESP004
NC043489	C	ESP004	BGE021885	1993----	Poaceae	Aegilops	triuncialis	L.			Rompesacos		ESP004
NC043490	C	ESP004	BGE021886	1993----	Poaceae	Aegilops	triuncialis	L.			Rompesacos		ESP004
NC043491	C	ESP004	BGE021887	1993----	Poaceae	Aegilops	triuncialis	L.			Rompesacos		ESP004

NC043492	C	ESP004	BGE021888	1993----	Poaceae	Aegilops	triuncialis	L.			Rompesacos		ESP004
NC043493	C	ESP004	BGE021889	1993----	Poaceae	Aegilops	triuncialis	L.			Rompesacos		ESP004
NC043494	C	ESP004	BGE021890	1993----	Poaceae	Aegilops	triuncialis	L.			Rompesacos		ESP004
NC043495	C	ESP004	BGE021891	1993----	Poaceae	Aegilops	triuncialis	L.			Rompesacos		ESP004
NC043496	C	ESP004	BGE021892	1993----	Poaceae	Aegilops	triuncialis	L.			Rompesacos		ESP004
NC043497	C	ESP004	BGE021893	1993----	Poaceae	Aegilops	triuncialis	L.			Rompesacos		ESP004
NC043498	C	ESP004	BGE021894	1993----	Poaceae	Aegilops	triuncialis	L.			Rompesacos		ESP004
NC043499	C	ESP004	BGE021899	1993----	Poaceae	Aegilops	ventricosa	Tausch			Espiguilla ventruda		ESP004
NC043500	C	ESP004	BGE021900	1993----	Poaceae	Aegilops	ventricosa	Tausch			Espiguilla ventruda		ESP004
NC043501	C	ESP004	BGE021901	1993----	Poaceae	Aegilops	ventricosa	Tausch			Espiguilla ventruda		ESP004
NC043502	C	ESP004	BGE021902	1993----	Poaceae	Aegilops	ventricosa	Tausch			Espiguilla ventruda		ESP004
NC043503	C	ESP004	BGE021903	1993----	Poaceae	Aegilops	ventricosa	Tausch			Espiguilla ventruda		ESP004
NC043504	C	ESP004	BGE021904	1993----	Poaceae	Aegilops	ventricosa	Tausch			Espiguilla ventruda		ESP004
NC043505	C	ESP004	BGE021905	1993----	Poaceae	Aegilops	ventricosa	Tausch			Espiguilla ventruda		ESP004
NC043507	C	ESP004	BGE021907	1993----	Poaceae	Aegilops	biuncialis	Vis.					ESP004
NC043860	C	ESP004	BGE022197	1993----	Poaceae	Aegilops	geniculata	Roth			Trigo bastardo		ESP004
NC043861	C	ESP004	BGE022198	1993----	Poaceae	Aegilops	geniculata	Roth			Trigo bastardo		ESP004
NC044545	C	ESP004	BGE022742	1994----	Poaceae	Aegilops	geniculata	Roth			Trigo bastardo		ESP004
NC044546	C	ESP004	BGE022743	1994----	Poaceae	Aegilops	geniculata	Roth			Trigo bastardo		ESP004
NC044547	C	ESP004	BGE022744	1994----	Poaceae	Aegilops	geniculata	Roth			Trigo bastardo		ESP004
NC044548	C	ESP004	BGE022745	1994----	Poaceae	Aegilops	geniculata	Roth			Trigo bastardo		ESP004
NC044549	C	ESP004	BGE022746	1994----	Poaceae	Aegilops	geniculata	Roth			Trigo bastardo		ESP004
NC044550	C	ESP004	BGE022747	1994----	Poaceae	Aegilops	geniculata	Roth			Trigo bastardo		ESP004
NC044551	C	ESP004	BGE022748	1994----	Poaceae	Aegilops	geniculata	Roth			Trigo bastardo		ESP004
NC044552	C	ESP004	BGE022749	1994----	Poaceae	Aegilops	geniculata	Roth			Trigo bastardo		ESP004
NC044553	C	ESP004	BGE022750	1994----	Poaceae	Aegilops	geniculata	Roth			Trigo bastardo		ESP004
NC050470	C	ESP004	BGE023608	1995----	Poaceae	Aegilops	geniculata	Roth			Trigo bastardo		JPN001
NC050471	C	ESP004	BGE023616	1995----	Poaceae	Aegilops	triuncialis	L.			Rompesacos		JPN001
NC050472	C	ESP004	BGE023609	1995----	Poaceae	Aegilops	geniculata	Roth			Trigo bastardo		JPN001
NC050473	C	ESP004	BGE023624	1995----	Poaceae	Aegilops	ventricosa	Tausch			Espiguilla ventruda		JPN001
NC050474	C	ESP004	BGE023617	1995----	Poaceae	Aegilops	triuncialis	L.			Rompesacos		JPN001
NC050476	C	ESP004	BGE023610	1995----	Poaceae	Aegilops	geniculata	Roth			Trigo bastardo		JPN001
NC050477	C	ESP004	BGE023625	1995----	Poaceae	Aegilops	ventricosa	Tausch			Espiguilla ventruda		JPN001

NC050478	C	ESP004	BGE023618	1995----	Poaceae	Aegilops	triuncialis	L.			Rompesacos		JPN001
NC050480	C	ESP004	BGE023611	1995----	Poaceae	Aegilops	geniculata	Roth			Trigo bastardo		JPN001
NC050481	C	ESP004	BGE023626	1995----	Poaceae	Aegilops	ventricosa	Tausch			Espiguilla ventruda		JPN001
NC050482	C	ESP004	BGE023619	1995----	Poaceae	Aegilops	triuncialis	L.			Rompesacos		JPN001
NC050484	C	ESP004	BGE023612	1995----	Poaceae	Aegilops	geniculata	Roth			Trigo bastardo		JPN001
NC050485	C	ESP004	BGE023627	1995----	Poaceae	Aegilops	ventricosa	Tausch			Espiguilla ventruda		JPN001
NC050486	C	ESP004	BGE023620	1995----	Poaceae	Aegilops	triuncialis	L.			Rompesacos		JPN001
NC050487	C	ESP004	BGE023613	1995----	Poaceae	Aegilops	geniculata	Roth			Trigo bastardo		JPN001
NC050488	C	ESP004	BGE023628	1995----	Poaceae	Aegilops	ventricosa	Tausch			Espiguilla ventruda		JPN001
NC050489	C	ESP004	BGE023621	1995----	Poaceae	Aegilops	triuncialis	L.			Rompesacos		JPN001
NC050490	C	ESP004	BGE023629	1995----	Poaceae	Aegilops	biuncialis	Vis.					JPN001
NC050496	C	ESP004	BGE023614	1995----	Poaceae	Aegilops	geniculata	Roth			Trigo bastardo		JPN001
NC050497	C	ESP004	BGE023622	1995----	Poaceae	Aegilops	triuncialis	L.			Rompesacos		JPN001
NC050499	C	ESP004	BGE023615	1995----	Poaceae	Aegilops	geniculata	Roth			Trigo bastardo		JPN001
NC050500	C	ESP004	BGE023623	1995----	Poaceae	Aegilops	triuncialis	L.			Rompesacos		JPN001
NC050501	C	ESP004	BGE023630	1995----	Poaceae	Aegilops	biuncialis	Vis.					JPN001
NC056065	E				Poaceae	Aegilops	sp.						ESP004
NC061596	C				Poaceae	Aegilops	triuncialis	L.			Rompesacos		ESP003
NC061688	C				Poaceae	Aegilops	geniculata	Roth			Trigo bastardo		ESP004
NC061689	C				Poaceae	Aegilops	triuncialis	L.			Rompesacos		ESP004
NC091744	C	ESP004	BGE044149	20111202	Poaceae	Aegilops	biuncialis	Vis.				Rompesacos	ESP004
NC091745	C	ESP004	BGE043305	20101110	Poaceae	Aegilops	geniculata	Roth			Trigo bastardo	Rompesacos	ESP004
NC091747	C	ESP004	BGE043306	20101110	Poaceae	Aegilops	triuncialis	L.			Rompesacos		ESP004
NC099988	C	ESP004	BGE044646	20120424	Poaceae	Aegilops	geniculata	Roth			Trigo bastardo		ESP022
NC101471	C	ESP004	BGE044647	20120424	Poaceae	Aegilops	geniculata	Roth	subsp. gibb	(Zhuk.) K.H	Trigo bastardo		
NC101472	C	ESP004	BGE044648	20120424	Poaceae	Aegilops	ventricosa	Tausch	var. ventric	(Coss. & Du	Espiguilla ventruda		
NC101473	C	ESP004	BGE044649	20120424	Poaceae	Aegilops	geniculata	Roth	subsp. gibb	(Zhuk.) K.H	Trigo bastardo		
NC101474	C	ESP004	BGE044650	20120424	Poaceae	Aegilops	geniculata	Roth			Trigo bastardo		
NC101475	C	ESP004	BGE044651	20120424	Poaceae	Aegilops	neglecta	Req. ex Ber	subsp. rect	(Zhuk.) K.Hammer			DEU146
NC101476	C	ESP004	BGE044652	20120424	Poaceae	Aegilops	neglecta	Req. ex Ber	subsp. rect	(Zhuk.) K.Hammer			DEU146
NC101477	C	ESP004	BGE044653	20120424	Poaceae	Aegilops	neglecta	Req. ex Ber	subsp. rect	(Zhuk.) K.Hammer			DEU146
NC101478	C	ESP004	BGE044654	20120424	Poaceae	Aegilops	neglecta	Req. ex Ber	subsp. rect	(Zhuk.) K.Hammer			
NC101479	C	ESP004	BGE044655	20120424	Poaceae	Aegilops	geniculata	Roth			Trigo bastardo		

NC101480	C	ESP004	BGE044656	20120424	Poaceae	Aegilops	geniculata	Roth			Trigo bastardo		DEU146
NC101481	C	ESP004	BGE044657	20120424	Poaceae	Aegilops	neglecta	Req. ex Ber	subsp. rect	(Zhuk.) K.Hammer			DEU146
NC101482	C	ESP004	BGE044658	20120424	Poaceae	Aegilops	neglecta	Req. ex Ber	subsp. rect	(Zhuk.) K.Hammer			DEU146
NC101483	C	ESP004	BGE044659	20120424	Poaceae	Aegilops	neglecta	Req. ex Ber	subsp. rect	(Zhuk.) K.Hammer			DEU146
NC101484	C	ESP004	BGE044660	20120424	Poaceae	Aegilops	neglecta	Req. ex Ber	subsp. rect	(Zhuk.) K.Hammer			DEU146
NC101485	C	ESP004	BGE044661	20120424	Poaceae	Aegilops	triuncialis	L.	subsp. triur	Popova	Rompesacos		
NC101486	C	ESP004	BGE044662	20120424	Poaceae	Aegilops	geniculata	Roth			Trigo bastardo		
NC101487	C	ESP004	BGE044663	20120424	Poaceae	Aegilops	ventricosa	Tausch			Espiguilla ventruda		
NC101488	C	ESP004	BGE044664	20120424	Poaceae	Aegilops	triuncialis	L.	subsp. triur	Popova	Rompesacos		
NC101489	C	ESP004	BGE044665	20120424	Poaceae	Aegilops	geniculata	Roth			Trigo bastardo		
NC101490	C	ESP004	BGE044666	20120424	Poaceae	Aegilops	triuncialis	L.			Rompesacos		
NC101491	C	ESP004	BGE044667	20120424	Poaceae	Aegilops	ventricosa	Tausch			Espiguilla ventruda		
NC101492	C	ESP004	BGE044668	20120424	Poaceae	Aegilops	triuncialis	L.	subsp. triur	Popova	Rompesacos		
NC101493	C	ESP004	BGE044669	20120424	Poaceae	Aegilops	geniculata	Roth			Trigo bastardo		
NC101494	C	ESP004	BGE044670	20120424	Poaceae	Aegilops	triuncialis	L.	subsp. triur	(Boiss.) Eig	Rompesacos		
NC101495	C	ESP004	BGE044671	20120424	Poaceae	Aegilops	ventricosa	Tausch	var. comos	(Coss. & Du	Espiguilla ventruda		
NC101496	C	ESP004	BGE044672	20120424	Poaceae	Aegilops	geniculata	Roth			Trigo bastardo		
NC101497	C	ESP004	BGE044673	20120424	Poaceae	Aegilops	geniculata	Roth	subsp. gibb	(Zhuk.) K.H	Trigo bastardo		
NC101498	C	ESP004	BGE044674	20120424	Poaceae	Aegilops	triuncialis	L.	subsp. triur	Eig	Rompesacos		
NC101499	C	ESP004	BGE044675	20120424	Poaceae	Aegilops	geniculata	Roth	subsp. geniculata		Trigo bastardo		
NC101500	C	ESP004	BGE044676	20120424	Poaceae	Aegilops	geniculata	Roth			Trigo bastardo		
NC101501	C	ESP004	BGE044677	20120424	Poaceae	Aegilops	triuncialis	L.			Rompesacos		DEU146
NC101502	C	ESP004	BGE044678	20120424	Poaceae	Aegilops	triuncialis	L.	subsp. triur	Popova	Rompesacos		
NC101503	C	ESP004	BGE044679	20120424	Poaceae	Aegilops	triuncialis	L.	subsp. triur	(Boiss.) Eig	Rompesacos		DEU146
NC101504	C	ESP004	BGE044680	20120424	Poaceae	Aegilops	triuncialis	L.	subsp. triur	(Boiss.) Eig	Rompesacos		
NC101505	C	ESP004	BGE044681	20120424	Poaceae	Aegilops	ventricosa	Tausch			Espiguilla ventruda		
NC101506	C	ESP004	BGE044682	20120424	Poaceae	Aegilops	geniculata	Roth	subsp. geniculata		Trigo bastardo		
NC101507	C	ESP004	BGE044683	20120424	Poaceae	Aegilops	geniculata	Roth			Trigo bastardo		
NC101508	C	ESP004	BGE044684	20120424	Poaceae	Aegilops	geniculata	Roth			Trigo bastardo		
NC101509	C	ESP004	BGE044685	20120424	Poaceae	Aegilops	triuncialis	L.			Rompesacos		
NC101510	C	ESP004	BGE044686	20120424	Poaceae	Aegilops	ventricosa	Tausch			Espiguilla ventruda		
NC101511	C	ESP004	BGE044687	20120424	Poaceae	Aegilops	triuncialis	L.			Rompesacos		
NC101512	C	ESP004	BGE044688	20120424	Poaceae	Aegilops	neglecta	Req. ex Bertol.					DEU146

NC101647	C	ESP004	BGE045860	20131028	Poaceae	Aegilops	biuncialis	Vis.					ESP004
NC101648	C	ESP004	BGE045861	20131028	Poaceae	Aegilops	biuncialis	Vis.					ESP004
NC101649	C	ESP004	BGE045862	20131028	Poaceae	Aegilops	geniculata	Roth			Trigo bastardo		ESP004
NC101650	C	ESP004	BGE045863	20131028	Poaceae	Aegilops	geniculata	Roth			Trigo bastardo		ESP004
NC101651	C	ESP004	BGE045864	20131028	Poaceae	Aegilops	geniculata	Roth			Trigo bastardo		ESP004
NC101652	C	ESP004	BGE045865	20131028	Poaceae	Aegilops	geniculata	Roth			Trigo bastardo		ESP004
NC101653	C	ESP004	BGE044939	20130228	Poaceae	Aegilops	geniculata	Roth			Trigo bastardo		ESP004
NC101654	C	ESP004	BGE045866	20131028	Poaceae	Aegilops	geniculata	Roth			Trigo bastardo		ESP004
NC101655	C	ESP004	BGE044940	20130228	Poaceae	Aegilops	geniculata	Roth			Trigo bastardo		ESP004
NC101656	C	ESP004	BGE045867	20131028	Poaceae	Aegilops	geniculata	Roth			Trigo bastardo		ESP004
NC101657	C	ESP004	BGE045868	20131028	Poaceae	Aegilops	geniculata	Roth			Trigo bastardo		ESP004
NC101658	C	ESP004	BGE045869	20131028	Poaceae	Aegilops	geniculata	Roth			Trigo bastardo		ESP004
NC101659	C	ESP004	BGE045870	20131028	Poaceae	Aegilops	geniculata	Roth			Trigo bastardo		ESP004
NC101660	C	ESP004	BGE045871	20131028	Poaceae	Aegilops	geniculata	Roth			Trigo bastardo		ESP004
NC101661	C	ESP004	BGE045872	20131028	Poaceae	Aegilops	geniculata	Roth			Trigo bastardo		ESP004
NC101662	E				Poaceae	Aegilops	geniculata	Roth			Trigo bastardo		ESP004
NC101663	C	ESP004	BGE045873	20131028	Poaceae	Aegilops	geniculata	Roth			Trigo bastardo		ESP004
NC101664	C	ESP004	BGE045874	20131028	Poaceae	Aegilops	geniculata	Roth			Trigo bastardo		ESP004
NC101665	C	ESP004	BGE045875	20131028	Poaceae	Aegilops	geniculata	Roth			Trigo bastardo		ESP004
NC101666	C	ESP004	BGE045876	20131028	Poaceae	Aegilops	geniculata	Roth			Trigo bastardo		ESP004
NC101667	C	ESP004	BGE044941	20130228	Poaceae	Aegilops	geniculata	Roth			Trigo bastardo		ESP004
NC101668	C	ESP004	BGE045877	20131028	Poaceae	Aegilops	geniculata	Roth			Trigo bastardo		ESP004
NC101669	C	ESP004	BGE045878	20131028	Poaceae	Aegilops	geniculata	Roth			Trigo bastardo		ESP004
NC101670	C	ESP004	BGE045879	20131028	Poaceae	Aegilops	geniculata	Roth			Trigo bastardo		ESP004
NC101671	C	ESP004	BGE044942	20130228	Poaceae	Aegilops	geniculata	Roth			Trigo bastardo		ESP004
NC101672	C	ESP004	BGE045880	20131028	Poaceae	Aegilops	geniculata	Roth			Trigo bastardo		ESP004
NC101673	C	ESP004	BGE045881	20131028	Poaceae	Aegilops	geniculata	Roth			Trigo bastardo		ESP004
NC101674	C	ESP004	BGE045882	20131028	Poaceae	Aegilops	geniculata	Roth			Trigo bastardo		ESP004
NC101675	C	ESP004	BGE045883	20131028	Poaceae	Aegilops	triuncialis	L.			Rompesacos		ESP004
NC101676	C	ESP004	BGE045884	20131028	Poaceae	Aegilops	triuncialis	L.			Rompesacos		ESP004
NC101677	C	ESP004	BGE045885	20131028	Poaceae	Aegilops	triuncialis	L.			Rompesacos		ESP004
NC101678	C	ESP004	BGE045886	20131028	Poaceae	Aegilops	triuncialis	L.			Rompesacos		ESP004
NC101679	C	ESP004	BGE045887	20131028	Poaceae	Aegilops	triuncialis	L.			Rompesacos		ESP004

NC101680	C	ESP004	BGE045888	20131028	Poaceae	Aegilops	triuncialis	L.			Rompesacos		ESP004
NC101681	C	ESP004	BGE045889	20131028	Poaceae	Aegilops	triuncialis	L.			Rompesacos		ESP004
NC101682	C	ESP004	BGE045890	20131028	Poaceae	Aegilops	triuncialis	L.			Rompesacos		ESP004
NC101683	C	ESP004	BGE045891	20131028	Poaceae	Aegilops	triuncialis	L.			Rompesacos		ESP004
NC101684	C	ESP004	BGE045892	20131028	Poaceae	Aegilops	triuncialis	L.			Rompesacos		ESP004
NC101685	C	ESP004	BGE045893	20131028	Poaceae	Aegilops	triuncialis	L.			Rompesacos		ESP004
NC101686	C	ESP004	BGE044943	20130228	Poaceae	Aegilops	triuncialis	L.			Rompesacos		ESP004
NC101687	C	ESP004	BGE045894	20131028	Poaceae	Aegilops	triuncialis	L.			Rompesacos		ESP004
NC101688	C	ESP004	BGE045895	20131028	Poaceae	Aegilops	triuncialis	L.			Rompesacos		ESP004
NC101689	C	ESP004	BGE045896	20131028	Poaceae	Aegilops	triuncialis	L.			Rompesacos		ESP004
NC101690	C	ESP004	BGE045897	20131028	Poaceae	Aegilops	triuncialis	L.			Rompesacos		ESP004
NC101691	C	ESP004	BGE045898	20131028	Poaceae	Aegilops	triuncialis	L.			Rompesacos	Rompesacos	ESP004
NC101692	C	ESP004	BGE045899	20131028	Poaceae	Aegilops	triuncialis	L.			Rompesacos		ESP004
NC101693	C	ESP004	BGE044944	20130228	Poaceae	Aegilops	ventricosa	Tausch			Espiguilla ventruda		ESP004
NC101694	C	ESP004	BGE044945	20130228	Poaceae	Aegilops	ventricosa	Tausch			Espiguilla ventruda		ESP004
NC101695	C	ESP004	BGE044946	20130228	Poaceae	Aegilops	ventricosa	Tausch			Espiguilla ventruda		ESP004
NC101696	C	ESP004	BGE045900	20131028	Poaceae	Aegilops	triuncialis	L.			Rompesacos		ESP004
NC103942	C	ESP004	BGE045901	20131028	Poaceae	Aegilops	geniculata	Roth			Trigo bastardo		ESP004
NC103943	C	ESP004	BGE045902	20131028	Poaceae	Aegilops	triuncialis	L.			Rompesacos		ESP004
NC103944	C	ESP004	BGE045903	20131028	Poaceae	Aegilops	geniculata	Roth			Trigo bastardo		ESP004
NC103945	C	ESP004	BGE047414	20141021	Poaceae	Aegilops	geniculata	Roth			Trigo bastardo		ESP004
NC103946	C	ESP004	BGE047415	20141021	Poaceae	Aegilops	geniculata	Roth			Trigo bastardo		ESP004
NC103947	E				Poaceae	Aegilops	geniculata	Roth			Trigo bastardo		ESP004
NC103948	C	ESP004	BGE047416	20141021	Poaceae	Aegilops	geniculata	Roth			Trigo bastardo		ESP004
NC103949	C	ESP004	BGE047417	20141021	Poaceae	Aegilops	geniculata	Roth			Trigo bastardo		ESP004
NC103950	C	ESP004	BGE047418	20141021	Poaceae	Aegilops	geniculata	Roth			Trigo bastardo		ESP004
NC103951	C	ESP004	BGE045904	20131028	Poaceae	Aegilops	geniculata	Roth			Trigo bastardo		ESP004
NC103952	C				Poaceae	Aegilops	geniculata	Roth			Trigo bastardo		ESP004
NC103953	C	ESP004	BGE047419	20141021	Poaceae	Aegilops	triuncialis	L.			Rompesacos		ESP004
NC103954	C	ESP004	BGE045905	20131028	Poaceae	Aegilops	triuncialis	L.			Rompesacos		ESP004
NC103955	C	ESP004	BGE045906	20131028	Poaceae	Aegilops	geniculata	Roth			Trigo bastardo		ESP004
NC103956	C	ESP004	BGE047420	20141021	Poaceae	Aegilops	geniculata	Roth			Trigo bastardo		ESP004
NC103957	C	ESP004	BGE045907	20131028	Poaceae	Aegilops	triuncialis	L.			Rompesacos		ESP004

NC103958	C	ESP004	BGE047421	20141021	Poaceae	Aegilops	geniculata	Roth			Trigo bastardo		ESP004
NC103959	C	ESP004	BGE045908	20131028	Poaceae	Aegilops	triuncialis	L.			Rompesacos		ESP004
NC103960	E				Poaceae	Aegilops	geniculata	Roth			Trigo bastardo		ESP004
NC103961	C	ESP004	BGE047422	20141021	Poaceae	Aegilops	geniculata	Roth			Trigo bastardo		ESP004
NC103962	C	ESP004	BGE047423	20141021	Poaceae	Aegilops	triuncialis	L.			Rompesacos		ESP004
NC103963	C	ESP004	BGE045909	20131028	Poaceae	Aegilops	triuncialis	L.			Rompesacos		ESP004
NC103964	C	ESP004	BGE047424	20141021	Poaceae	Aegilops	geniculata	Roth			Trigo bastardo		ESP004
NC103965	C	ESP004	BGE047425	20141021	Poaceae	Aegilops	geniculata	Roth			Trigo bastardo		ESP004
NC103966	C	ESP004	BGE045910	20131028	Poaceae	Aegilops	ventricosa	Tausch			Espiguilla ventruda		ESP004
NC103967	C	ESP004	BGE045911	20131028	Poaceae	Aegilops	geniculata	Roth			Trigo bastardo		ESP004
NC103968	C	ESP004	BGE047426	20141021	Poaceae	Aegilops	geniculata	Roth			Trigo bastardo		ESP004
NC103969	C	ESP004	BGE045912	20131028	Poaceae	Aegilops	triuncialis	L.			Rompesacos		ESP004
NC103970	C	ESP004	BGE047427	20141021	Poaceae	Aegilops	triuncialis	L.			Rompesacos		ESP004
NC103971	C	ESP004	BGE047428	20141021	Poaceae	Aegilops	geniculata	Roth			Trigo bastardo		ESP004
NC103972	C	ESP004	BGE047429	20141021	Poaceae	Aegilops	triuncialis	L.			Rompesacos		ESP004
NC103973	C	ESP004	BGE047430	20141021	Poaceae	Aegilops	geniculata	Roth			Trigo bastardo		ESP004
NC103974	C	ESP004	BGE047431	20141021	Poaceae	Aegilops	ventricosa	Tausch			Espiguilla ventruda		ESP004
NC103975	C	ESP004	BGE045913	20131028	Poaceae	Aegilops	geniculata	Roth			Trigo bastardo		ESP004
NC103976	C	ESP004	BGE045914	20131028	Poaceae	Aegilops	triuncialis	L.			Rompesacos		ESP004
NC103977	C	ESP004	BGE045915	20131028	Poaceae	Aegilops	geniculata	Roth			Trigo bastardo		ESP004
NC103978	C	ESP004	BGE045916	20131028	Poaceae	Aegilops	geniculata	Roth			Trigo bastardo		ESP004
NC103979	C	ESP004	BGE047432	20141021	Poaceae	Aegilops	triuncialis	L.			Rompesacos		ESP004
NC103980	C	ESP004	BGE045917	20131028	Poaceae	Aegilops	geniculata	Roth			Trigo bastardo		ESP004
NC103981	C	ESP004	BGE047433	20141021	Poaceae	Aegilops	triuncialis	L.			Rompesacos		ESP004
NC103982	C	ESP004	BGE047434	20141021	Poaceae	Aegilops	geniculata	Roth			Trigo bastardo		ESP004
NC103983	C	ESP004	BGE045918	20131028	Poaceae	Aegilops	geniculata	Roth			Trigo bastardo		ESP004
NC103984	C	ESP004	BGE045919	20131028	Poaceae	Aegilops	triuncialis	L.			Rompesacos		ESP004
NC103985	C	ESP004	BGE047435	20141021	Poaceae	Aegilops	triuncialis	L.			Rompesacos		ESP004
NC103986	C	ESP004	BGE047436	20141021	Poaceae	Aegilops	geniculata	Roth			Trigo bastardo		ESP004
NC103987	C	ESP004	BGE045977	20131212	Poaceae	Aegilops	geniculata	Roth			Trigo bastardo		ESP010
NC103988	C	ESP004	BGE045978	20131212	Poaceae	Aegilops	triuncialis	L.			Rompesacos		ESP010
NC103989	C	ESP004	BGE045979	20131212	Poaceae	Aegilops	geniculata	Roth			Trigo bastardo		ESP010
NC103990	C	ESP004	BGE045980	20131212	Poaceae	Aegilops	geniculata	Roth			Trigo bastardo		ESP010

NC103991	C	ESP004	BGE045981	20131212	Poaceae	Aegilops	geniculata	Roth			Trigo bastardo		ESP010
NC103992	C	ESP004	BGE045982	20131212	Poaceae	Aegilops	triuncialis	L.			Rompesacos		ESP010
NC103993	C	ESP004	BGE045983	20131212	Poaceae	Aegilops	triuncialis	L.			Rompesacos		ESP010
NC103994	C	ESP004	BGE045984	20131212	Poaceae	Aegilops	neglecta	Req. ex Bertol.					ESP010
NC103995	C	ESP004	BGE045985	20131212	Poaceae	Aegilops	geniculata	Roth			Trigo bastardo		ESP010
NC103996	C	ESP004	BGE045986	20131212	Poaceae	Aegilops	triuncialis	L.			Rompesacos		ESP010
NC103997	C	ESP004	BGE045987	20131212	Poaceae	Aegilops	geniculata	Roth			Trigo bastardo		ESP010
NC103998	C	ESP004	BGE045988	20131212	Poaceae	Aegilops	triuncialis	L.			Rompesacos		ESP010
NC103999	C	ESP004	BGE045989	20131212	Poaceae	Aegilops	geniculata	Roth			Trigo bastardo		ESP010
NC104000	C	ESP004	BGE045990	20131212	Poaceae	Aegilops	geniculata	Roth			Trigo bastardo		ESP010
NC104001	C	ESP004	BGE045991	20131212	Poaceae	Aegilops	triuncialis	L.			Rompesacos		ESP010
NC104002	C	ESP004	BGE045992	20131212	Poaceae	Aegilops	geniculata	Roth			Trigo bastardo		ESP010
NC104003	C	ESP004	BGE045993	20131212	Poaceae	Aegilops	triuncialis	L.			Rompesacos		ESP010
NC104004	C	ESP004	BGE045994	20131212	Poaceae	Aegilops	geniculata	Roth			Trigo bastardo		ESP010
NC104005	C	ESP004	BGE045995	20131212	Poaceae	Aegilops	triuncialis	L.			Rompesacos		ESP010
NC104006	C	ESP004	BGE045996	20131212	Poaceae	Aegilops	geniculata	Roth			Trigo bastardo		ESP010
NC104007	C	ESP004	BGE045997	20131212	Poaceae	Aegilops	geniculata	Roth			Trigo bastardo		ESP010
NC104008	C	ESP004	BGE045998	20131212	Poaceae	Aegilops	geniculata	Roth			Trigo bastardo		ESP010
NC104009	C	ESP004	BGE045999	20131212	Poaceae	Aegilops	geniculata	Roth			Trigo bastardo		ESP010
NC104010	C	ESP004	BGE046000	20131212	Poaceae	Aegilops	triuncialis	L.			Rompesacos		ESP010
NC104011	C	ESP004	BGE046001	20131212	Poaceae	Aegilops	triuncialis	L.			Rompesacos		ESP010
NC104012	C	ESP004	BGE046002	20131212	Poaceae	Aegilops	triuncialis	L.			Rompesacos		ESP010
NC104013	C	ESP004	BGE046003	20131212	Poaceae	Aegilops	neglecta	Req. ex Bertol.					ESP010
NC104014	C	ESP004	BGE046004	20131212	Poaceae	Aegilops	triuncialis	L.			Rompesacos		ESP010
NC104015	C	ESP004	BGE046005	20131212	Poaceae	Aegilops	triuncialis	L.			Rompesacos		ESP010
NC104016	C	ESP004	BGE046006	20131212	Poaceae	Aegilops	geniculata	Roth			Trigo bastardo		ESP010
NC104017	C	ESP004	BGE046007	20131212	Poaceae	Aegilops	triuncialis	L.			Rompesacos		ESP010
NC104018	C	ESP004	BGE046008	20131212	Poaceae	Aegilops	triuncialis	L.			Rompesacos		ESP010
NC104019	C	ESP004	BGE046009	20131212	Poaceae	Aegilops	triuncialis	L.			Rompesacos		ESP010
NC104020	C	ESP004	BGE046010	20131212	Poaceae	Aegilops	geniculata	Roth			Trigo bastardo		ESP010
NC104021	C	ESP004	BGE046011	20131212	Poaceae	Aegilops	geniculata	Roth			Trigo bastardo		ESP010
NC104022	C	ESP004	BGE046012	20131212	Poaceae	Aegilops	triuncialis	L.			Rompesacos		ESP010
NC104023	C	ESP004	BGE046013	20131212	Poaceae	Aegilops	geniculata	Roth			Trigo bastardo		ESP010

NC104024	C	ESP004	BGE046014	20131212	Poaceae	Aegilops	triuncialis	L.			Rompesacos		ESP010
NC105771	C	ESP004	BGE046723	20140415	Poaceae	Aegilops	geniculata	Roth			Trigo bastardo		ESP010
NC105772	C	ESP004	BGE046724	20140415	Poaceae	Aegilops	geniculata	Roth			Trigo bastardo		ESP010
NC105773	C	ESP004	BGE046725	20140415	Poaceae	Aegilops	geniculata	Roth			Trigo bastardo		ESP010
NC105774	C	ESP004	BGE046726	20140415	Poaceae	Aegilops	geniculata	Roth			Trigo bastardo		ESP010
NC105775	C	ESP004	BGE046727	20140415	Poaceae	Aegilops	geniculata	Roth			Trigo bastardo		ESP010
NC105776	C	ESP004	BGE046728	20140415	Poaceae	Aegilops	geniculata	Roth					ESP010
NC105777	C	ESP004	BGE046729	20140415	Poaceae	Aegilops	neglecta	Req. ex Bertol.					ESP010
NC105778	C	ESP004	BGE046730	20140415	Poaceae	Aegilops	triuncialis	L.			Rompesacos		ESP010
NC105779	C	ESP004	BGE046731	20140415	Poaceae	Aegilops	triuncialis	L.			Rompesacos		ESP010
NC105780	C	ESP004	BGE046732	20140415	Poaceae	Aegilops	triuncialis	L.			Rompesacos		ESP010
NC105781	C	ESP004	BGE046733	20140415	Poaceae	Aegilops	triuncialis	L.			Rompesacos		ESP010
NC105782	C	ESP004	BGE046734	20140415	Poaceae	Aegilops	triuncialis	L.			Rompesacos		ESP010
NC105783	C	ESP004	BGE046735	20140415	Poaceae	Aegilops	triuncialis	L.			Rompesacos		ESP010
NC105784	C	ESP004	BGE046736	20140415	Poaceae	Aegilops	triuncialis	L.			Rompesacos		ESP010
NC105785	C	ESP004	BGE046737	20140415	Poaceae	Aegilops	triuncialis	L.			Rompesacos		ESP010
NC105786	C	ESP004	BGE046738	20140415	Poaceae	Aegilops	triuncialis	L.			Rompesacos		ESP010
NC105787	C	ESP004	BGE046739	20140415	Poaceae	Aegilops	triuncialis	L.			Rompesacos		ESP010
NC105788	C	ESP004	BGE046740	20140415	Poaceae	Aegilops	triuncialis	L.			Rompesacos		ESP010
NC105789	C	ESP004	BGE046741	20140415	Poaceae	Aegilops	triuncialis	L.			Rompesacos		ESP010
NC105790	C	ESP004	BGE046742	20140415	Poaceae	Aegilops	triuncialis	L.			Rompesacos		ESP010
NC105791	C	ESP004	BGE046743	20140415	Poaceae	Aegilops	triuncialis	L.			Rompesacos		ESP010
NC105792	C	ESP004	BGE046744	20140415	Poaceae	Aegilops	triuncialis	L.			Rompesacos		ESP010
NC105793	C	ESP004	BGE046745	20140415	Poaceae	Aegilops	triuncialis	L.			Rompesacos		ESP010
NC105794	C	ESP004	BGE046746	20140415	Poaceae	Aegilops	triuncialis	L.			Rompesacos		ESP010
NC105795	C	ESP004	BGE046747	20140415	Poaceae	Aegilops	triuncialis	L.			Rompesacos		ESP010
NC105796	C	ESP004	BGE046748	20140415	Poaceae	Aegilops	triuncialis	L.			Rompesacos		ESP010
NC105797	C	ESP004	BGE046749	20140415	Poaceae	Aegilops	triuncialis	L.			Rompesacos		ESP010
NC105798	C	ESP004	BGE046750	20140415	Poaceae	Aegilops	triuncialis	L.			Rompesacos		ESP010
NC105799	C	ESP004	BGE046751	20140415	Poaceae	Aegilops	triuncialis	L.			Rompesacos		ESP010
NC105800	C	ESP004	BGE046752	20140415	Poaceae	Aegilops	triuncialis	L.			Rompesacos		ESP010
NC105801	C	ESP004	BGE046753	20140415	Poaceae	Aegilops	triuncialis	L.			Rompesacos		ESP010
NC105802	C	ESP004	BGE046754	20140415	Poaceae	Aegilops	triuncialis	L.			Rompesacos		ESP010

NC105803	C	ESP004	BGE046755	20140415	Poaceae	Aegilops	triuncialis	L.			Rompesacos		ESP010
NC105804	C	ESP004	BGE046756	20140415	Poaceae	Aegilops	triuncialis	L.			Rompesacos		ESP010
NC105805	C	ESP004	BGE046757	20140415	Poaceae	Aegilops	triuncialis	L.			Rompesacos		ESP010
NC105806	C	ESP004	BGE046758	20140415	Poaceae	Aegilops	triuncialis	L.			Rompesacos		ESP010
NC105807	C	ESP004	BGE046759	20140415	Poaceae	Aegilops	triuncialis	L.			Rompesacos		ESP010
NC105808	C	ESP004	BGE046760	20140415	Poaceae	Aegilops	triuncialis	L.			Rompesacos		ESP010
NC105809	C	ESP004	BGE046761	20140415	Poaceae	Aegilops	triuncialis	L.			Rompesacos		ESP010
NC105810	C	ESP004	BGE046762	20140415	Poaceae	Aegilops	triuncialis	L.			Rompesacos		ESP010
NC105811	C	ESP004	BGE046763	20140415	Poaceae	Aegilops	triuncialis	L.			Rompesacos		ESP010
NC105812	C	ESP004	BGE046764	20140415	Poaceae	Aegilops	triuncialis	L.			Rompesacos		ESP010
NC105813	C	ESP004	BGE046765	20140415	Poaceae	Aegilops	triuncialis	L.			Rompesacos		ESP010
NC105814	C	ESP004	BGE046766	20140415	Poaceae	Aegilops	triuncialis	L.			Rompesacos		ESP010
NC105815	C	ESP004	BGE046767	20140415	Poaceae	Aegilops	triuncialis	L.			Rompesacos		ESP010
NC105816	C	ESP004	BGE046768	20140415	Poaceae	Aegilops	triuncialis	L.			Rompesacos		ESP010
NC105817	C	ESP004	BGE046769	20140415	Poaceae	Aegilops	triuncialis	L.			Rompesacos		ESP010
NC105818	C	ESP004	BGE046770	20140415	Poaceae	Aegilops	triuncialis	L.			Rompesacos		ESP010
NC105819	C	ESP004	BGE046771	20140415	Poaceae	Aegilops	triuncialis	L.			Rompesacos		ESP010
NC105820	C	ESP004	BGE046772	20140415	Poaceae	Aegilops	triuncialis	L.			Rompesacos		ESP010
NC105821	C	ESP004	BGE046773	20140415	Poaceae	Aegilops	triuncialis	L.			Rompesacos		ESP010
NC105822	C	ESP004	BGE046774	20140415	Poaceae	Aegilops	triuncialis	L.			Rompesacos		ESP010
NC105823	C	ESP004	BGE046775	20140415	Poaceae	Aegilops	triuncialis	L.			Rompesacos		ESP010
NC105824	C	ESP004	BGE046776	20140415	Poaceae	Aegilops	triuncialis	L.			Rompesacos		ESP010
NC105825	C	ESP004	BGE046777	20140415	Poaceae	Aegilops	triuncialis	L.			Rompesacos		ESP010
NC105826	C	ESP004	BGE046778	20140415	Poaceae	Aegilops	triuncialis	L.			Rompesacos		ESP010
NC105827	C	ESP004	BGE046779	20140415	Poaceae	Aegilops	triuncialis	L.			Rompesacos		ESP010
NC105828	C	ESP004	BGE046780	20140415	Poaceae	Aegilops	triuncialis	L.			Rompesacos		ESP010
NC105829	C	ESP004	BGE046781	20140415	Poaceae	Aegilops	triuncialis	L.			Rompesacos		ESP010
NC105830	C	ESP004	BGE046782	20140415	Poaceae	Aegilops	triuncialis	L.			Rompesacos		ESP010
NC105831	C	ESP004	BGE046783	20140415	Poaceae	Aegilops	triuncialis	L.			Rompesacos		ESP010
NC105832	C	ESP004	BGE046784	20140415	Poaceae	Aegilops	triuncialis	L.			Rompesacos		ESP010
NC105833	C	ESP004	BGE046785	20140415	Poaceae	Aegilops	triuncialis	L.			Rompesacos		ESP010
NC105834	C	ESP004	BGE046786	20140415	Poaceae	Aegilops	triuncialis	L.			Rompesacos		ESP010
NC105835	C	ESP004	BGE046787	20140415	Poaceae	Aegilops	triuncialis	L.			Rompesacos		ESP010

NC105836	C	ESP004	BGE047437	20141021	Poaceae	Aegilops	triuncialis	L.			Rompesacos		ESP046
NC105837	C	ESP004	BGE047438	20141021	Poaceae	Aegilops	triuncialis	L.			Rompesacos		ESP046
NC106650					Poaceae	Aegilops	geniculata	Roth			Trigo bastardo		ESP046
NC106651	C	ESP004	BGE047984	20150414	Poaceae	Aegilops	geniculata	Roth			Trigo bastardo		ESP046
NC106652					Poaceae	Aegilops	neglecta	Req. ex Bertol.					ESP046
NC106653	C	ESP004	BGE047985	20150414	Poaceae	Aegilops	triuncialis	L.			Rompesacos		ESP046
NC106654					Poaceae	Aegilops	neglecta	Req. ex Bertol.					ESP046
NC106655	C	ESP004	BGE047986	20150414	Poaceae	Aegilops	geniculata	Roth			Trigo bastardo		ESP046
NC106656	C	ESP004	BGE047987	20150414	Poaceae	Aegilops	triuncialis	L.			Rompesacos		ESP046
NC106657					Poaceae	Aegilops	neglecta	Req. ex Bertol.			Rompesacos		ESP046
NC106658	C	ESP004	BGE047988	20150414	Poaceae	Aegilops	triuncialis	L.			Rompesacos		ESP046
NC106659					Poaceae	Aegilops	geniculata	Roth			Trigo bastardo		ESP046
NC106660	C	ESP004	BGE047989	20150414	Poaceae	Aegilops	triuncialis	L.			Rompesacos		ESP046
NC106661	C	ESP004	BGE047990	20150414	Poaceae	Aegilops	triuncialis	L.			Rompesacos		ESP046
NC106662	C	ESP004	BGE047991	20150414	Poaceae	Aegilops	neglecta	Req. ex Bertol.					ESP046
NC106663	C				Poaceae	Aegilops	geniculata	Roth			Trigo bastardo		ESP046
NC106664	C	ESP004	BGE047992	20150414	Poaceae	Aegilops	geniculata	Roth			Trigo bastardo		ESP046
NC106665	C	ESP004	BGE047993	20150414	Poaceae	Aegilops	triuncialis	L.			Rompesacos		ESP046
NC106666	C	ESP004	BGE047994	20150414	Poaceae	Aegilops	triuncialis	L.			Rompesacos		ESP046
NC106667	C	ESP004	BGE047995	20150414	Poaceae	Aegilops	triuncialis	L.			Rompesacos		ESP046
NC106668	C	ESP004	BGE047996	20150414	Poaceae	Aegilops	triuncialis	L.			Rompesacos		ESP046
NC106669					Poaceae	Aegilops	geniculata	Roth			Trigo bastardo		ESP046
NC106670	C	ESP004	BGE047997	20150414	Poaceae	Aegilops	geniculata	Roth			Trigo bastardo		ESP046
NC106671	C	ESP004	BGE047998	20150414	Poaceae	Aegilops	geniculata	Roth			Trigo bastardo		ESP046
NC106672	C	ESP004	BGE047999	20150414	Poaceae	Aegilops	triuncialis	L.			Rompesacos		ESP046
NC106673	C	ESP004	BGE048000	20150414	Poaceae	Aegilops	triuncialis	L.			Rompesacos		ESP046
NC106674	C	ESP004	BGE048001	20150414	Poaceae	Aegilops	geniculata	Roth			Trigo bastardo		ESP046
NC106675	C	ESP004	BGE048002	20150414	Poaceae	Aegilops	triuncialis	L.			Rompesacos		ESP046
NC106676					Poaceae	Aegilops	geniculata	Roth			Trigo bastardo		ESP046
NC106677					Poaceae	Aegilops	neglecta	Req. ex Bertol.			Rompesacos		ESP046
NC106678	C	ESP004	BGE048003	20150414	Poaceae	Aegilops	triuncialis	L.			Rompesacos		ESP046
NC106679	C	ESP004	BGE048004	20150414	Poaceae	Aegilops	triuncialis	L.			Rompesacos		ESP046
NC106680	C	ESP004	BGE048067	20150414	Poaceae	Aegilops	geniculata	Roth			Trigo bastardo		ESP046

NC106681	C	ESP004	BGE048068	20150414	Poaceae	Aegilops	triuncialis	L.			Rompesacos		ESP046
NC106682					Poaceae	Aegilops	geniculata	Roth			Trigo bastardo		ESP046
NC106683	C	ESP004	BGE048069	20150414	Poaceae	Aegilops	triuncialis	L.			Rompesacos		ESP046
NC106684	C	ESP004	BGE048070	20150414	Poaceae	Aegilops	geniculata	Roth			Trigo bastardo		ESP046
NC106685	C	ESP004	BGE048005	20150414	Poaceae	Aegilops	triuncialis	L.			Rompesacos		ESP046
NC106686	C	ESP004	BGE048006	20150414	Poaceae	Aegilops	triuncialis	L.			Rompesacos		ESP046
NC106687	C	ESP004	BGE048007	20150414	Poaceae	Aegilops	triuncialis	L.			Rompesacos		ESP046
NC106688	C	ESP004	BGE048008	20150414	Poaceae	Aegilops	triuncialis	L.			Rompesacos		ESP046
NC106689	C	ESP004	BGE048009	20150414	Poaceae	Aegilops	triuncialis	L.			Rompesacos		ESP046
NC106690	C	ESP004	BGE048010	20150414	Poaceae	Aegilops	geniculata	Roth			Trigo bastardo		ESP046
NC106691	C	ESP004	BGE048011	20150414	Poaceae	Aegilops	geniculata	Roth			Trigo bastardo		ESP046
NC106692	C	ESP004	BGE048012	20150414	Poaceae	Aegilops	triuncialis	L.			Rompesacos		ESP046
NC106693	C	ESP004	BGE048013	20150414	Poaceae	Aegilops	triuncialis	L.			Rompesacos		ESP046
NC106694	C	ESP004	BGE048014	20150414	Poaceae	Aegilops	triuncialis	L.			Rompesacos		ESP046
NC106989	C	ESP004	BGE048015	20150414	Poaceae	Aegilops	triuncialis	L.			Rompesacos		ESP010
NC106990	C	ESP004	BGE048016	20150414	Poaceae	Aegilops	neglecta	Req. ex Bertol.					ESP010
NC106991	C	ESP004	BGE048017	20150414	Poaceae	Aegilops	triuncialis	L.			Rompesacos		ESP010
NC106992	C	ESP004	BGE048018	20150414	Poaceae	Aegilops	triuncialis	L.			Rompesacos		ESP010
NC106993	C	ESP004	BGE048019	20150414	Poaceae	Aegilops	triuncialis	L.			Rompesacos		ESP010
NC106994	C	ESP004	BGE048020	20150414	Poaceae	Aegilops	triuncialis	L.			Rompesacos		ESP010
NC106995	C	ESP004	BGE048021	20150414	Poaceae	Aegilops	neglecta	Req. ex Bertol.					ESP010
NC106996	C	ESP004	BGE048022	20150414	Poaceae	Aegilops	triuncialis	L.			Rompesacos		ESP010
NC106997	C	ESP004	BGE048023	20150414	Poaceae	Aegilops	triuncialis	L.			Rompesacos		ESP010
NC106998	C	ESP004	BGE048024	20150414	Poaceae	Aegilops	triuncialis	L.			Rompesacos		ESP010
NC106999	C	ESP004	BGE048025	20150414	Poaceae	Aegilops	triuncialis	L.			Rompesacos		ESP010
NC107000	C	ESP004	BGE048026	20150414	Poaceae	Aegilops	triuncialis	L.			Rompesacos		ESP010
NC107001	C	ESP004	BGE048027	20150414	Poaceae	Aegilops	geniculata	Roth			Trigo bastardo		ESP010
NC107002	C	ESP004	BGE048028	20150414	Poaceae	Aegilops	triuncialis	L.			Rompesacos		ESP010
NC107003	C	ESP004	BGE048029	20150414	Poaceae	Aegilops	triuncialis	L.			Rompesacos		ESP010
NC107004	C	ESP004	BGE048030	20150414	Poaceae	Aegilops	triuncialis	L.			Rompesacos		ESP010
NC107005	C	ESP004	BGE048031	20150414	Poaceae	Aegilops	triuncialis	L.			Rompesacos		ESP010
NC107006	C	ESP004	BGE048032	20150414	Poaceae	Aegilops	triuncialis	L.			Rompesacos		ESP010
NC107007	C	ESP004	BGE048033	20150414	Poaceae	Aegilops	triuncialis	L.			Rompesacos		ESP010

NC107008	C	ESP004	BGE048034	20150414	Poaceae	Aegilops	geniculata	Roth			Trigo bastardo		ESP010
NC107009	C	ESP004	BGE048035	20150414	Poaceae	Aegilops	triuncialis	L.			Rompesacos		ESP010
NC107010	C	ESP004	BGE048036	20150414	Poaceae	Aegilops	geniculata	Roth			Trigo bastardo		ESP010
NC107011	C	ESP004	BGE048037	20150414	Poaceae	Aegilops	geniculata	Roth			Trigo bastardo		ESP010
NC107012	C	ESP004	BGE048038	20150414	Poaceae	Aegilops	geniculata	Roth			Trigo bastardo		ESP010
NC107013	C	ESP004	BGE048039	20150414	Poaceae	Aegilops	geniculata	Roth			Trigo bastardo		ESP010
NC107014	C	ESP004	BGE048040	20150414	Poaceae	Aegilops	triuncialis	L.			Rompesacos		ESP010
NC107015	C	ESP004	BGE048041	20150414	Poaceae	Aegilops	neglecta	Req. ex Bertol.					ESP010
NC107016	C	ESP004	BGE048042	20150414	Poaceae	Aegilops	geniculata	Roth			Trigo bastardo		ESP010
NC107017	C	ESP004	BGE048043	20150414	Poaceae	Aegilops	neglecta	Req. ex Bertol.					ESP010
NC107018	C	ESP004	BGE048044	20150414	Poaceae	Aegilops	geniculata	Roth			Trigo bastardo		ESP010
NC107019	C	ESP004	BGE048045	20150414	Poaceae	Aegilops	triuncialis	L.			Rompesacos		ESP010
NC107020	C	ESP004	BGE048046	20150414	Poaceae	Aegilops	triuncialis	L.			Rompesacos		ESP010
NC107021	C	ESP004	BGE048047	20150414	Poaceae	Aegilops	geniculata	Roth			Trigo bastardo		ESP010
NC107022	C	ESP004	BGE048048	20150414	Poaceae	Aegilops	geniculata	Roth			Trigo bastardo		ESP010
NC107023	C	ESP004	BGE048049	20150414	Poaceae	Aegilops	neglecta	Req. ex Bertol.					ESP010
NC107024	C	ESP004	BGE048050	20150414	Poaceae	Aegilops	triuncialis	L.			Rompesacos		ESP010
NC107025	C	ESP004	BGE048051	20150414	Poaceae	Aegilops	triuncialis	L.			Rompesacos		ESP010
NC107026	C	ESP004	BGE048052	20150414	Poaceae	Aegilops	neglecta	Req. ex Bertol.					ESP010
NC107027	C	ESP004	BGE048053	20150414	Poaceae	Aegilops	triuncialis	L.			Rompesacos		ESP010
NC107028	C	ESP004	BGE048054	20150414	Poaceae	Aegilops	neglecta	Req. ex Bertol.					ESP010
NC107029	C	ESP004	BGE048055	20150414	Poaceae	Aegilops	geniculata	Roth			Trigo bastardo		ESP010
NC107030	C	ESP004	BGE048056	20150414	Poaceae	Aegilops	neglecta	Req. ex Bertol.					ESP010
NC107031	C	ESP004	BGE048057	20150414	Poaceae	Aegilops	geniculata	Roth			Trigo bastardo		ESP010
NC107032	C	ESP004	BGE048058	20150414	Poaceae	Aegilops	geniculata	Roth			Trigo bastardo		ESP010
NC107033	C	ESP004	BGE048059	20150414	Poaceae	Aegilops	triuncialis	L.			Rompesacos		ESP010
NC107034	C	ESP004	BGE048060	20150414	Poaceae	Aegilops	geniculata	Roth			Trigo bastardo		ESP010
NC107035	C	ESP004	BGE048061	20150414	Poaceae	Aegilops	geniculata	Roth			Trigo bastardo		ESP010
NC107036	C	ESP004	BGE048062	20150414	Poaceae	Aegilops	geniculata	Roth			Trigo bastardo		ESP010
NC107037	C	ESP004	BGE048063	20150414	Poaceae	Aegilops	geniculata	Roth			Rompesacos		ESP010
NC107038	C	ESP004	BGE048064	20150414	Poaceae	Aegilops	triuncialis	L.			Rompesacos		ESP010
NC107039	C	ESP004	BGE048065	20150414	Poaceae	Aegilops	neglecta	Req. ex Bertol.					ESP010
NC107040	C	ESP004	BGE048066	20150414	Poaceae	Aegilops	neglecta	Req. ex Bertol.					ESP010

NC107060	C	ESP004	BGE048071	20150414	Poaceae	Aegilops	triuncialis	L.			Rompesacos		ESP010
NC107541	C				Poaceae	Aegilops	neglecta	Req. ex Bertol.					ESP046
NC107542	C				Poaceae	Aegilops	neglecta	Req. ex Bertol.					ESP004

NUMCAT	CODREC	NUMREC	FECREC	PAIORI	ESTREG	PROVIN	MUNICI	LOCALI	LATITU	LONGIT	ALTITU	TIPMAT
NC010107	E014	3650	19820713	ESP	Castilla y Leon	Burgos	Palacios de La Sierra		4157--N	00307--W	1040	100
NC010108	E014	3652	19820713	ESP	Castilla y Leon	Burgos		Palacios de La Sierra	4159--N	00310--W	1110	100
NC010109	E014	3667	19820714	ESP	Castilla y Leon	Burgos	Valle de Sedano	Sedano,Sedano/Pes	4244--N	00341--W	930	100
NC011029	E004	871	19790817	ESP	Castilla y Leon	Burgos	Sarracin		4215--N	00341--W	865	200
NC015807	E004	702	19790730	ESP	Castilla-La Mancha	Cuenca	Carboneras de Guadazaon		3954--N	00148--W	1026	100
NC015808	E004	703	19790730	ESP	Castilla-La Mancha	Cuenca	Carboneras de Guadazaon		3954--N	00148--W	1026	100
NC015809	E004	706	19790731	ESP	Castilla-La Mancha	Cuenca	Fuertescusa		4028--N	00210--W	989	100
NC015811	E004	710	19790731	ESP	Castilla-La Mancha	Cuenca	Cañamares		4027--N	00214--W	885	200
NC015812	E004	711	19790731	ESP	Castilla-La Mancha	Cuenca	Cañamares		4027--N	00214--W	885	200
NC015813	E004	712	19790731	ESP	Castilla-La Mancha	Cuenca	Cañamares		4027--N	00214--W	885	200
NC015815	E004	734	19790802	ESP	Castilla y Leon	Leon	Almanza	500m	4240--N	00502--W	970	100
NC015825	E004	870	19790817	ESP	Castilla y Leon	Burgos	Sarracin		4215--N	00341--W	865	200
NC015826	E004	873	19790817	ESP	Castilla y Leon	Segovia	Pradales	Carabias	4127--N	00340--W	1000	200
NC015827	E004	872	19790817	ESP	Castilla y Leon	Segovia	Pradales	Carabias	4127--N	00340--W	1000	200
NC022308	E021	318	19870708	ESP	Andalucia	Cadiz	Arcos de la Fronter	El Guijo	3644--N	00552--W	102	100
NC022311	E021	339	19870709	ESP	Andalucia	Cadiz	Olvera		3656--N	00516--W	623	100
NC022312	E021	340	19870709	ESP	Andalucia	Cadiz	Olvera		3656--N	00516--W	623	100
NC022338	E020	013G	19870606	ESP	Andalucia	Cordoba	Espiel	Fuente-Agria				100
NC022339	E020	014G	19870606	ESP	Andalucia	Cordoba	Espiel	Fuente-Agria				100
NC022340	E020	015G	19870606	ESP	Andalucia	Cordoba	Espiel	Fuente-Agria				100
NC022349	E020	024G/1	19870607	ESP	Andalucia	Cordoba	Cordoba	Medina Azahara	3753--N	00452--W	219	100
NC022350	E020	024G/2	19870607	ESP	Andalucia	Cordoba	Cordoba	Medina Azahara	3753--N	00452--W	219	100
NC024016	E021	62	19870629	ESP	Andalucia	Granada	Puebla de Don Fadr	Cortijos Nuevos	3752--N	00220--W	1120	100
NC024018	E021	64	19870629	ESP	Andalucia	Granada	Puebla de Don Fadr	Las Santas	3758--N	00231--W	1216	100
NC024023	E021	69	19870629	ESP	Andalucia	Granada	Puebla de Don Fadr	Las Santas	3758--N	00231--W	1216	100
NC024038	E021	130	19870630	ESP	Andalucia	Granada	Darro		3721--N	00317--W	1120	100
NC024039	E021	137	19870630	ESP	Andalucia	Granada	Huetor de Santillan	El Molinillo	3718--N	00326--W	786	100
NC024042	E021	140	19870630	ESP	Andalucia	Granada	Huetor de Santillan	Puerto de la Mora	3714--N	00329--W	1265	100
NC024050	E021	206	19870702	ESP	Andalucia	Granada	Alhama de Granada	Alcornoquillo	3656--N	00401--W	1078	100
NC024051	E021	207	19870702	ESP	Andalucia	Granada	Alhama de Granada	Alcornoquillo	3656--N	00401--W	1078	100
NC024072	E021	245	19870704	ESP	Andalucia	Granada	Durcal		3659--N	00333--W	783	100

NC027317	E025	001G	198807--	ESP	Andalucia	Jaen	La Carolina	Navas de Tolosa	3817--N	00335--W	505	100
NC027318	E025	002G	198807--	ESP	Andalucia	Jaen	La Carolina	Navas de Tolosa	3817--N	00335--W	505	100
NC027323	E025	007G	198807--	ESP	Andalucia	Jaen	Martos		3743--N	00357--W	753	100
NC027324	E025	008G	198807--	ESP	Andalucia	Jaen	Martos		3743--N	00357--W	753	100
NC027325	E025	009G	198807--	ESP	Andalucia	Granada	Moclin		3720--N	00347--W	1065	100
NC027326	E025	010G	198807--	ESP	Andalucia	Granada	Moclin		3720--N	00347--W	1065	100
NC027327	E025	011G	198807--	ESP	Andalucia	Granada	Moclin		3720--N	00347--W	1065	100
NC027331	E025	015G	198807--	ESP	Andalucia	Granada	Illora		3717--N	00352--W	744	100
NC027332	E025	016G	198807--	ESP	Andalucia	Granada	Illora		3717--N	00352--W	744	100
NC027334	E025	018G	198807--	ESP	Andalucia	Granada	Illora	Alomartes	3715--N	00354--W	775	100
NC027335	E025	019G	198807--	ESP	Andalucia	Granada	Illora	Alomartes	3715--N	00354--W	775	100
NC027336	E025	020G	198807--	ESP	Andalucia	Granada	Illora	Alomartes	3715--N	00354--W	775	100
NC027337	E025	021G	198807--	ESP	Andalucia	Granada	Lanjaron		3655--N	00328--W	659	100
NC027338	E025	022G	198807--	ESP	Andalucia	Granada	Lanjaron		3655--N	00328--W	659	100
NC027339	E025	023G	198807--	ESP	Andalucia	Granada	Motril		3644--N	00331--W	40	100
NC027341	E025	025G	198807--	ESP	Andalucia	Almeria						100
NC027342	E025	026G	198807--	ESP	Andalucia	Granada	Cañar		3655--N	00325--W	1040	100
NC027343	E025	027G	198807--	ESP	Andalucia	Granada	Cañar		3655--N	00325--W	1040	100
NC027344	E025	028G	198807--	ESP	Andalucia	Almeria						100
NC027345	E025	029G	198807--	ESP	Andalucia	Almeria						100
NC027346	E025	030G	198807--	ESP	Andalucia	Almeria						100
NC027349	E025	033G	198807--	ESP	Andalucia	Almeria						100
NC027350	E025	034G	198807--	ESP	Andalucia	Almeria						100
NC027351	E025	035G	198807--	ESP	Andalucia	Almeria						100
NC027355	E025	039G	198807--	ESP	Andalucia	Almeria						100
NC027356	E025	040G	198807--	ESP	Andalucia	Almeria	Los Gallardos		3710--N	00156--W	120	100
NC027360	E025	044G	198807--	ESP	Murcia	Murcia						100
NC027362	E025	046G	198807--	ESP	Murcia	Murcia	Cartagena	El Algar	3739--N	00052--W	40	100
NC027366	E025	050G	198807--	ESP	Andalucia	Cordoba	Cordoba	Alcolea	3755--N	00440--W	109	100
NC027367	E025	051G	198807--	ESP	Andalucia	Cordoba	Cordoba	Alcolea	3755--N	00440--W	109	100
NC027369	E025	053G	198807--	ESP	Andalucia	Cordoba	Almodovar del Rio		3748--N	00501--W	125	100
NC027370	E025	054G	198807--	ESP	Andalucia	Cordoba	Almodovar del Rio		3748--N	00501--W	125	100
NC027371	E025	055G	198807--	ESP	Andalucia	Cordoba	Almodovar del Rio		3748--N	00501--W	125	100

NC027373	E025	057G	198807--	ESP	Andalucia	Sevilla	Cazalla de la Sierra		3755--N	00545--W	593	100
NC027374	E025	058G	198807--	ESP	Andalucia	Sevilla	Cazalla de la Sierra		3755--N	00545--W	593	100
NC027376	E025	060G	198807--	ESP	Andalucia	Sevilla	Cazalla de la Sierra		3755--N	00545--W	593	100
NC027377	E025	061G	198807--	ESP	Andalucia	Sevilla						100
NC027378	E025	062G	198807--	ESP	Andalucia	Sevilla	Cazalla de la Sierra		3755--N	00545--W	593	100
NC027379	E025	063G	198807--	ESP	Andalucia	Sevilla	Cazalla de la Sierra		3755--N	00545--W	593	100
NC027380	E025	064G	198807--	ESP	Andalucia	Sevilla	Cazalla de la Sierra		3755--N	00545--W	593	100
NC027384	E025	068G	198807--	ESP	Andalucia	Huelva	Aljaraque		3716--N	00701--W	39	100
NC027385	E025	069G	198807--	ESP	Andalucia	Huelva						100
NC027388	E025	072G	198807--	ESP	Andalucia	Sevilla	La Puebla del Rio		3716--N	00603--W	30	100
NC027389	E025	073G	198807--	ESP	Andalucia	Cadiz	El Puerto de Santa Maria		3636--N	00613--W	15	100
NC027390	E025	074G	198807--	ESP	Andalucia	Cadiz	El Puerto de Santa Maria		3636--N	00613--W	15	100
NC027393	E025	077G	198807--	ESP	Andalucia	Cadiz	Tarifa		3601--N	00535--W	85	100
NC027394	E025	078G	198807--	ESP	Andalucia	Malaga	Marbella	San Pedro de Alcant	3628--N	00459--W	7	100
NC027395	E025	079G	198807--	ESP	Andalucia	Malaga	Marbella	San Pedro de Alcant	3628--N	00459--W	7	100
NC027396	E025	080G	198807--	ESP	Andalucia	Cadiz	Grazalema		3646--N	00521--W	800	100
NC027397	E025	081G	198807--	ESP	Andalucia	Cadiz	Grazalema		3646--N	00521--W	800	100
NC027398	E025	082G	198807--	ESP	Andalucia	Cadiz	Grazalema		3646--N	00521--W	800	100
NC027401	E025	085G	198807--	ESP	Andalucia	Malaga	Ronda		3644--N	00509--W	739	100
NC027402	E025	086G	198807--	ESP	Andalucia	Malaga	Ronda		3644--N	00509--W	739	100
NC027403	E025	087G	198807--	ESP	Andalucia	Malaga	Ronda		3644--N	00509--W	739	100
NC027405	E025	089G	198807--	ESP	Andalucia	Malaga	Antequera		3700--N	00433--W	577	100
NC027407	E025	091G	198807--	ESP	Andalucia	Malaga	Antequera		3700--N	00433--W	577	100
NC027409	E025	093G	198807--	ESP	Andalucia	Cordoba	Lucena		3724--N	00429--W	485	100
NC027410	E025	094G	198807--	ESP	Andalucia	Cordoba	Lucena		3724--N	00429--W	485	100
NC027412	E025	096G	198807--	ESP	Andalucia	Jaen	Quesada	Puerto de Tiscar	3748--N	00303--W	1112	100
NC027413	E025	097G	198807--	ESP	Andalucia	Jaen	Quesada	Puerto de Tiscar	3748--N	00303--W	1112	100
NC027414	E025	098G	198807--	ESP	Andalucia	Granada	Puebla de Don Fadrique		3757--N	00226--W	1164	100
NC027416	E025	100G	198807--	ESP	Andalucia	Granada	Puebla de Don Fadrique		3757--N	00226--W	1164	100
NC027417	E025	101G	198807--	ESP	Andalucia	Granada	Puebla de Don Fadrique		3757--N	00226--W	1164	100
NC027418	E025	104G	198807--	ESP	Cataluña	Tarragona	El Pont d'Armentera		4123--N	00121--E	349	100
NC027421	E033	107G	19890710	ESP	Castilla-La Mancha	Cuenca	Huelves		4002--N	00252--W	817	100
NC027422	E033	108G	19890710	ESP	Castilla-La Mancha	Cuenca	Campos del Paraiso	Carrascosa del Cam	4002--N	00245--W	898	100

NC027423	E033	109G	19890710	ESP	Castilla-La Mancha	Cuenca	Campos del Paraiso	Carrascosa del Cam	4002--N	00245--W	898	100
NC027424	E033	110G	19890710	ESP	Castilla-La Mancha	Cuenca	Cuenca	Albaladejito	4005--N	00212--W	822	100
NC027426	E033	112G	19890710	ESP	Castilla-La Mancha	Cuenca	Cuenca	Albaladejito	4005--N	00212--W	822	100
NC027428	E033	114G	19890710	ESP	Castilla-La Mancha	Cuenca	Cuenca	Ciudad Encantada	4012--N	00201--W	1320	100
NC027431	E033	117G	19890712	ESP	Valencia	Alicante	Calpe	Peñon de Ifach	3838--N	00002--E	322	100
NC027433	E033	119G	19890712	ESP	Valencia	Alicante	Polop		3837--N	00007--W	236	100
NC027434	E033	120G	19890712	ESP	Valencia	Alicante	Polop		3837--N	00007--W	236	100
NC027436	E033	122G	19890712	ESP	Valencia	Alicante	Vall de Ebo		3848--N	00009--W	394	100
NC027446	E033	132G	19890715	ESP	Cataluña	Barcelona	Sant Quirze del Valles		4132--N	00204--E	188	100
NC027447	E033	133G	19890718	ESP	Castilla-La Mancha	Guadalajar	Alcolea del Pinar		4102--N	00227--W	1205	100
NC027448	E033	134G	19890718	ESP	Castilla-La Mancha	Guadalajar	Alcolea del Pinar		4102--N	00227--W	1205	100
NC027450	E033	136G	19890718	ESP	Castilla-La Mancha	Guadalajar	Maranchon	Turmiel	410037N	0020345W	1122	100
NC027452	E033	138G	19890718	ESP	Castilla-La Mancha	Guadalajar	Maranchon	Turmiel	410037N	0020345W	1122	100
NC027454	E033	140G	19890718	ESP	Aragon	Teruel	Cella		4027--N	00117--W	1023	100
NC027457	E033	143G	19890719	ESP	Aragon	Teruel	Teruel		4020--N	00106--W	912	100
NC027459	E033	145G	19890719	ESP	Aragon	Teruel	Riodeva		4007--N	00108--W	967	100
NC027460	E033	146G	19890719	ESP	Aragon	Teruel	Riodeva		4007--N	00108--W	967	100
NC027462	E033	148G	19890720	ESP	Cataluña	Tarragona	Roquetes	Ports de Beceite				100
NC027463	E033	149G	19890720	ESP	Cataluña	Tarragona	Roquetes	Ports de Beceite				100
NC043467	C055	166G	199006--	ESP	Madrid	Madrid	Collado Villalba		4038--N	00359--W	917	100
NC043468	E037	177G	19900625	ESP	Baleares	Baleares	Soller	isla Mallorca	3945--N	00242--E	41	100
NC043469	E038	203G	19900703	ESP	Castilla-La Mancha	Ciudad Rea	Fuencaliente		3824--N	00418--W	700	100
NC043470	E021	208/3	19870702	ESP	Andalucia	Granada	Alhama de Granada	Alcornoquillo	3656--N	00401--W	1078	100
NC043471	E038	216G	19900709	ESP	Extremadura	Caceres	Belvis de Monroy	Casas de Belvis	3949--N	00535--W	296	100
NC043472	E038	219G	19900710	ESP	Extremadura	Caceres	Caceres	Minas de Aldeamor	3927--N	00623--W	451	100
NC043473	E038	225G	19900711	ESP	Extremadura	Caceres	Baños de Montemayor		4019--N	00551--W	705	100
NC043485	E037	187G	19900628	ESP	Baleares	Baleares	Soller	isla Mallorca	3945--N	00242--E	41	100
NC043486	E038	195G	19900701	ESP	Castilla-La Mancha	Guadalajar	Retiendas		4058--N	00316--W	895	100
NC043487	E038	202G	19900703	ESP	Castilla-La Mancha	Ciudad Rea	Fuencaliente		3824--N	00418--W	700	100
NC043488	E038	210G	19900706	ESP	Castilla y Leon	Segovia	Duruelo		4114--N	00338--W	1114	100
NC043489	E038	215G	19900706	ESP	Castilla y Leon	Segovia	Sepulveda	Duraton	4117--N	00341--W	943	100
NC043490	E038	217G	19900709	ESP	Extremadura	Caceres	Belvis de Monroy	Casas de Belvis	3949--N	00535--W	296	100
NC043491	E038	220G	19900710	ESP	Extremadura	Caceres	Caceres	Minas de Aldeamor	3927--N	00623--W	451	100

NC043492	E038	229G	19900711	ESP	Castilla y Leon	Salamanca	Babilafuente		4058--N	00525--W	801	100
NC043493	E038	232G	19900712	ESP	Castilla y Leon	Salamanca	Pedrosillo el Ralo		4103--N	00532--W	818	100
NC043494	E038	239G	19900712	ESP	Castilla y Leon	Salamanca	Cantalapiedra	Molino de la Villa	4109--N	00510--W	785	100
NC043495	E038	248G	19900713	ESP	Castilla y Leon	Valladolid	Encinas de Esgueva		4145--N	00406--W	832	100
NC043496	E038	251G	19900713	ESP	Castilla y Leon	Segovia	Adrada de Piron		4103--N	00402--W	1019	100
NC043497	E038	253G	19900707	ESP	Castilla-La Mancha	Guadalajar	Guadalajara		4038--N	00309--W	685	100
NC043498	E038	260G	19900722	ESP	Castilla-La Mancha	Guadalajar	Hontoba		4027--N	00302--W	730	100
NC043499	E037	171G	19900624	ESP	Baleares	Baleares	Palma de Mallorca		3934--N	00239--E	15	100
NC043500	E037	174G	19900625	ESP	Baleares	Baleares	Llucmajor		3929--N	00253--E	43	100
NC043501	E037	184G	19900627	ESP	Baleares	Baleares	Ciutadella de Menorca		4000--N	00350--E	17	100
NC043502	E038	230G	19900712	ESP	Castilla y Leon	Salamanca	Pedrosillo el Ralo		4103--N	00532--W	818	100
NC043503	E038	238G	19900712	ESP	Castilla y Leon	Salamanca	Cantalapiedra	Molino de la Villa	4109--N	00510--W	785	100
NC043504	E038	246G	19900713	ESP	Castilla y Leon	Valladolid	Encinas de Esgueva		4145--N	00406--W	832	100
NC043505	E038	250G	19900713	ESP	Castilla y Leon	Segovia	Adrada de Piron		4103--N	00402--W	1019	100
NC043507	E038	265G	19900725	ESP	Madrid	Madrid	Los Santos de la Humosa		4030--N	00315--W	906	100
NC043860	E038	191G	19900618	ESP	Madrid	Madrid	Alcala de Henares		4028--N	00322--W	587	100
NC043861	E038	221G	19900710	ESP	Extremadura	Caceres	Caceres	Minas de Aldeamor	3927--N	00623--W	451	100
NC044545	E037	168G	19900623	ESP	Baleares	Baleares	Felanitx	Porto Colom,Cala S'	3925--N	00313--E	6	100
NC044546	E037	179G	19900626	ESP	Baleares	Baleares	Es Mercadal		3959--N	00405--E	96	100
NC044547	E037	181G	19900626	ESP	Baleares	Baleares		Menorca,Turmadem				100
NC044548	E037	185G	19900627	ESP	Baleares	Baleares	Ciutadella de Meno	Parelleta				100
NC044549	E038	228G	19900711	ESP	Castilla y Leon	Salamanca	Babilafuente		4058--N	00525--W	801	100
NC044550	E038	236G	19900712	ESP	Castilla y Leon	Salamanca	Pajares de la Laguna		4105--N	00530--W	828	100
NC044551	E038	242G	19900712	ESP	Castilla y Leon	Salamanca	Cantalapiedra	Molino de la Villa	4109--N	00510--W	785	100
NC044552	E038	247G	19900713	ESP	Castilla y Leon	Valladolid	Encinas de Esgueva		4145--N	00406--W	832	100
NC044553	C005	442G/1	199110--	ESP	Andalucia	Jaen	Frailes		3729--N	00350--W	973	100
NC050470	E057	8/03/1A	19950803	ESP	Castilla-La Mancha	Cuenca	Huelves	Tarancon/Carrascos	4004--N	00252--W	840	100
NC050471	E057	8/03/1B	19950803	ESP	Castilla-La Mancha	Cuenca	Huelves	Tarancon/Carrascos	4004--N	00252--W	840	100
NC050472	E057	8/03/2A	19950803	ESP	Castilla-La Mancha	Cuenca	Fuentenava de Jaba	Cuenca/Carrascosa	4005--N	00213--W	960	100
NC050473	E057	8/03/2B	19950803	ESP	Castilla-La Mancha	Cuenca	Fuentenava de Jaba	Cuenca/Carrascosa	4005--N	00213--W	960	100
NC050474	E057	8/03/2C	19950803	ESP	Castilla-La Mancha	Cuenca	Fuentenava de Jaba	Cuenca/Carrascosa	4005--N	00213--W	960	100
NC050476	E057	8/03/4A	19950803	ESP	Castilla-La Mancha	Cuenca	Valdemorillo de la Sierra		4002--N	00146--W	1211	100
NC050477	E057	8/03/4B	19950803	ESP	Castilla-La Mancha	Cuenca	Valdemorillo de la Sierra		4002--N	00146--W	1211	100

NC050478	E057	8/03/4C	19950803	ESP	Castilla-La Mancha	Cuenca	Valdemorillo de la Sierra		4002--N	00146--W	1211	100
NC050480	E057	8/04/1A	19950804	ESP	Castilla-La Mancha	Cuenca	Cuenca	Cuenca/Villalba de l	4009--N	00208--W	940	100
NC050481	E057	8/04/1B	19950804	ESP	Castilla-La Mancha	Cuenca	Cuenca	Cuenca/Villalba de l	4009--N	00208--W	940	100
NC050482	E057	8/04/1C	19950804	ESP	Castilla-La Mancha	Cuenca	Cuenca	Cuenca/Villalba de l	4009--N	00208--W	940	100
NC050484	E057	8/04/2A	19950804	ESP	Castilla-La Mancha	Cuenca	Cuenca	Las Majadas/Beteta	4021--N	00200--W	1210	100
NC050485	E057	8/04/2B	19950804	ESP	Castilla-La Mancha	Cuenca	Cuenca	Las Majadas/Beteta	4021--N	00200--W	1210	100
NC050486	E057	8/04/2C	19950804	ESP	Castilla-La Mancha	Cuenca	Cuenca	Las Majadas/Beteta	4021--N	00200--W	1210	100
NC050487	E057	8/04/3A	19950804	ESP	Castilla-La Mancha	Cuenca	Cuenca	casa forestal de Teja	4024--N	00159--W	1080	100
NC050488	E057	8/04/3B	19950804	ESP	Castilla-La Mancha	Cuenca	Cuenca	casa forestal de Teja	4024--N	00159--W	1080	100
NC050489	E057	8/04/3C	19950804	ESP	Castilla-La Mancha	Cuenca	Cuenca	casa forestal de Teja	4024--N	00159--W	1080	100
NC050490	E057	8/04/3D	19950804	ESP	Castilla-La Mancha	Cuenca	Cuenca	casa forestal de Teja	4024--N	00159--W	1080	100
NC050496	E057	8/04/4F	19950804	ESP	Castilla-La Mancha	Cuenca	Poyatos	Beteta/Las Majadas	4025--N	00202--W	1520	100
NC050497	E057	8/04/4G	19950804	ESP	Castilla-La Mancha	Cuenca	Poyatos	Beteta/Las Majadas	4025--N	00202--W	1520	100
NC050499	E057	8/05/1A	19950805	ESP	Castilla-La Mancha	Cuenca	Villaconejos de Trab	Cañaveras/Priego 5	4024--N	00220--W	880	100
NC050500	E057	8/05/1B	19950805	ESP	Castilla-La Mancha	Cuenca	Villaconejos de Trab	Cañaveras/Priego 5	4024--N	00220--W	880	100
NC050501	E057	8/05/1C	19950805	ESP	Castilla-La Mancha	Cuenca	Villaconejos de Trab	Cañaveras/Priego 5	4024--N	00220--W	880	100
NC056065	E025	052G	198807--	ESP	Andalucia	Cordoba	Cordoba	Alcolea	3755--N	00440--W	109	100
NC061596	C013		1974----	ESP	Andalucia	Cadiz	Puerto Real		3631--N	00611--W	10	100
NC061688	C136		1974----	ESP	Madrid	Madrid	Madrid	Ciudad Universitaria				100
NC061689	C136		1974----	ESP	Madrid	Madrid	Madrid	Ciudad Universitaria				100
NC091744		2	20100714	ESP	Madrid	Madrid	Coslada		402616N	0033356W		100
NC091745		3	20100714	ESP	Madrid	Madrid	Coslada		402614N	0033359W		100
NC091747		5	20100714	ESP	Madrid	Madrid	Coslada		402616N	0033356W		100
NC099988		M-115		ESP	Castilla-La Mancha	Toledo			3950--N	0040000W	708	100
NC101471				ESP	Baleares	Baleares	Pollença	Formentor,isla M				100
NC101472				ESP	Baleares	Baleares	Llucmajor	isla Mallorca	392909N	0025341E		100
NC101473				ESP	Cataluña	Barcelona	Barcelona		412259N	0021059E		100
NC101474				ESP	Aragon	Huesca	Nueno	Nocito				100
NC101475		347	19780620	ESP	Extremadura	Caceres	Candeleda	Cerca del Monaster	400651N	0051540W		100
NC101476		348	19780621	ESP	Extremadura	Caceres		Al sur de Plasencia				100
NC101477		357	19780628	ESP	Castilla y Leon	Salamanca		Molinillo,cerca de Bejar				100
NC101478		360	19780630	ESP	Castilla y Leon	Avila		Carretera Piedralves/San Martin de Viera				100
NC101479		361	19780706	ESP								100

NC101480		302	19780603	ESP	Andalucia	Sevilla		ctra. Sevilla/Huelva a 4km de Sanlucar la M			40	100
NC101481		320	19780611	ESP	Andalucia	Huelva	Aracena	Finca La Cigüeñuela	375331N	0063115W		100
NC101482		321	19780611	ESP	Andalucia	Huelva	Aracena	Finca La Cigüeñuela	375331N	0063115W		100
NC101483		330	19780615	ESP	Extremadura	Caceres	Logrosan	Al norte de Logrosa	392053N	0053131W		100
NC101484		334	19780615	ESP	Extremadura	Caceres	Garciaz	Carretera Logrosan-	392242N	0053900W		100
NC101485				ESP				Guara				100
NC101486				ESP								100
NC101487				ESP	Castilla-La Mancha	Cuenca	Cañizares	comarca La Alcarria	403059N	0021500W		100
NC101488				ESP	Andalucia	Jaen	Huelma					100
NC101489				ESP				Pirineos				100
NC101490				ESP				Guara				100
NC101491				ESP	Castilla-La Mancha	Cuenca	Cañizares	omarca La Alcarria	403059N	0021500W		100
NC101492				ESP	Andalucia	Jaen	Huelma					100
NC101493				ESP				Pirineos				100
NC101494				ESP				Guara				100
NC101495				ESP	Castilla-La Mancha	Cuenca	Cañizares	omarca La Alcarria	403059N	0021500W		100
NC101496				ESP	Aragon	Huesca	Jaca		423419N	0002659W	810	100
NC101497				ESP	Aragon	Huesca	Jaca		423419N	0002659W	810	100
NC101498				ESP	Castilla y Leon	Soria	Fuentearmegil	Fuencaliente del Burgo				100
NC101499				ESP								100
NC101500				ESP	Andalucia	Granada	Morelabor	Moreda,40 km al NNE de Granada				100
NC101501		3637		ESP	Andalucia	Cordoba	Peñarroya-Pueblonuevo					100
NC101502				ESP	Andalucia	Cordoba	Peñarroya-Pueblonuevo					100
NC101503		321	19780611	ESP	Andalucia	Huelva	Aracena	Finca La Cigüeñuela	375331N	0063115W		100
NC101504		360	19780630	ESP	Castilla y Leon	Avila		Carretera Piedralves/San Martin de Viera				100
NC101505				ESP	Baleares	Baleares	Porreres		393000N	0030000E		100
NC101506				ESP								100
NC101507				ESP	Andalucia	Granada		Col de Veleta,Sierra Nevada				100
NC101508				ESP	Castilla y Leon	Burgos	Valle de Sedano	Cortiguera				100
NC101509				ESP	Andalucia	Cadiz	Puerto Real					100
NC101510				ESP	Baleares	Baleares	Palma de Mallorca	Bellver	393345N	0023712E		100
NC101511				ESP	Andalucia	Cadiz	Puerto Real					100
NC101512		302	19780603	ESP	Andalucia	Sevilla		ctra. Sevilla/Huelva a 4km de Sanlucar la M			40	100

NC101647	E153		2012----	ESP	Madrid	Madrid	Villalbilla	Los Hueros	402633N	0032120W	672	100
NC101648	E151		2012----	ESP	Madrid	Madrid	Alcala de Henares	El Encin	403041N	0031747W	606	100
NC101649	E151	9	20120705	ESP	Castilla-La Mancha	Albacete	Alcala del Jucar	Casas del Cerro,La V	391102N	0012529W	680	100
NC101650	E151	13	20120706	ESP	Castilla-La Mancha	Albacete	Yeste	Jartos	382345N	0021701W	716	100
NC101651	E151	2-1	20120705	ESP	Castilla-La Mancha	Albacete	Montalvos		391155N	0020206W	725	100
NC101652	E151	3-1	20120705	ESP	Castilla-La Mancha	Albacete	Albacete	Los Yesares	390633N	0014716W	696	100
NC101653	E151	4-1	20120705	ESP	Castilla-La Mancha	Albacete	Fuentealbilla		391555N	0013258W	682	100
NC101654	E151	7-1	20120705	ESP	Castilla-La Mancha	Albacete	Casas de Ves		391630N	0012254W	717	100
NC101655	E151	15-1	20120706	ESP	Castilla-La Mancha	Albacete	Alcaraz		384058N	0022938W	912	100
NC101656	E151	18-1	20120706	ESP	Castilla-La Mancha	Albacete	Alcaraz	El Horcajo	384326N	0022754W	1006	100
NC101657	E151	2-2	20120705	ESP	Castilla-La Mancha	Albacete	Montalvos		391155N	0020206W	725	100
NC101658	E151	3-2	20120705	ESP	Castilla-La Mancha	Albacete	Albacete	Los Yesares	390633N	0014716W	696	100
NC101659	E151	4-2	20120705	ESP	Castilla-La Mancha	Albacete	Fuentealbilla		391555N	0013258W	682	100
NC101660	E151	7-2	20120705	ESP	Castilla-La Mancha	Albacete	Casas de Ves		391630N	0012254W	717	100
NC101661	E151	18-2	20120706	ESP	Castilla-La Mancha	Albacete	Alcaraz	El Horcajo	384326N	0022754W	1006	100
NC101662	E151	7-3	20120705	ESP	Castilla-La Mancha	Albacete	Casas de Ves		391630N	0012254W	717	100
NC101663	E151		20120705	ESP	Madrid	Madrid	Alcala de Henares	Estacion de tren de	403033N	0032014W	596	100
NC101664	E151		2012----	ESP	Madrid	Madrid	Alcala de Henares	El Encin	403041N	0031747W	606	100
NC101665	E152		2012----	ESP	Castilla y Leon	Segovia	Castroserracin	cerca del huerto	412344N	0034802W	1132	100
NC101666	E153		2012----	ESP	Madrid	Madrid	Villalbilla	Los Hueros	402633N	0032120W	672	100
NC101667	E154		2012----	ESP	Castilla y Leon	Soria	Arcos de Jalon	Iruecha	410624N	0020532W	1250	100
NC101668	E153		2012----	ESP	Castilla-La Mancha	Guadalajar	Huertahernando					100
NC101669	E152		2012----	ESP	Castilla y Leon	Segovia	Castroserracin	fuente de la Reina	412314N	0034824W	1092	100
NC101670	E151	14	20120706	ESP	Castilla-La Mancha	Albacete	Yeste	Jartos	382346N	0021704W	724	100
NC101671	E153		2012----	ESP	Madrid	Madrid	Daganzo de Arriba		4032--N	00328--W		100
NC101672	E151	6	20120705	ESP	Castilla-La Mancha	Albacete	Fuentealbilla		391555N	0013258W	682	100
NC101673	E153		2012----	ESP	Madrid	Madrid	Daganzo de Arriba	ladera Carrabazan	403255N	0032812W		100
NC101674	E151	15-2	20120706	ESP	Castilla-La Mancha	Albacete	Alcaraz		384058N	0022938W	912	100
NC101675	E151	8	20120705	ESP	Castilla-La Mancha	Albacete	Casas de Ves		391630N	0012254W	717	100
NC101676	E151	10	20120705	ESP	Castilla-La Mancha	Albacete	Alcala del Jucar	Casas del Cerro,La V	391104N	0012528W	687	100
NC101677	E151	19	20120706	ESP	Castilla-La Mancha	Albacete	Alcaraz	El Horcajo	384326N	0022754W	1006	100
NC101678	E151	5-1	20120705	ESP	Castilla-La Mancha	Albacete	Fuentealbilla		391555N	0013258W	682	100
NC101679	E151	12-1	20120706	ESP	Castilla-La Mancha	Albacete	Yeste	Jartos	382345N	0021701W	712	100

NC101680	E151	16-1	20120706	ESP	Castilla-La Mancha	Albacete	Alcaraz		384058N	0022937W	912	100
NC101681	E151	5-2	20120705	ESP	Castilla-La Mancha	Albacete	Fuentealbilla		391555N	0013258W	682	100
NC101682	E151	12-2	20120706	ESP	Castilla-La Mancha	Albacete	Yeste	Jartos	382345N	0021701W	712	100
NC101683	E151	16-2	20120706	ESP	Castilla-La Mancha	Albacete	Alcaraz		384058N	0022937W	912	100
NC101684	E151		2012----	ESP	Madrid	Madrid	Alcala de Henares	Estacion de tren de	403033N	0032014W	596	100
NC101685	E152		2012----	ESP	Castilla y Leon	Segovia	Castroserracin	cerca del huerto	412344N	0034802W	1132	100
NC101686	E152		2012----	ESP	Castilla y Leon	Segovia	Castroserracin	fuente de la Reina	412314N	0034824W	1092	100
NC101687	E153		2012----	ESP	Madrid	Madrid	Daganzo de Arriba	ladera Carrabazan	403255N	0032812W		100
NC101688	E153		2012----	ESP	Madrid	Madrid	Villalbilla	Los Hueros	402633N	0032120W		100
NC101689	E153		2012----	ESP	Madrid	Madrid	Villalbilla	Los Hueros	402633N	0032120W		100
NC101690	E153		2012----	ESP	Madrid	Madrid	Villalbilla	Los Hueros	402633N	0032120W		100
NC101691	E151	1	20120705	ESP	Castilla-La Mancha	Albacete	Montalvos		391154N	0020207W	731	100
NC101692	E153		2012----	ESP	Castilla-La Mancha	Guadalajar	Huertahernando					100
NC101693	E152		2012----	ESP	Castilla y Leon	Segovia	Castroserracin	cerca del huerto	412344N	0034802W	1132	100
NC101694	E151	20-2	20120706	ESP	Castilla-La Mancha	Albacete	Alcaraz	El Horcajo	384326N	0022754W	1011	100
NC101695	E151	20-1	20120706	ESP	Castilla-La Mancha	Albacete	Alcaraz	El Horcajo	384326N	0022754W	1011	100
NC101696	E155		2012----	ESP	Madrid	Madrid	Alcala de Henares	El Encin	403041N	0031747W	606	100
NC103942	E175	21	20130628	ESP	Castilla-La Mancha	Toledo	Ocaña		395649N	0033034W	730	100
NC103943	E175	22	20130628	ESP	Castilla-La Mancha	Toledo	Añover de Tajo		400008N	0034646W	630	100
NC103944	E175	23	20130628	ESP	Castilla-La Mancha	Toledo	Añover de Tajo		400008N	0034647W	629	100
NC103945	E175	60	20130709	ESP	Castilla-La Mancha	Ciudad Rea	Villarrubia de los Ojos		391237N	0033739W	629	100
NC103946	E175	25	20130628	ESP	Castilla-La Mancha	Toledo	Cabañas de la Sagra	Carretera N401	395902N	0035757W	510	100
NC103947	E175	26/1	20130628	ESP	Castilla-La Mancha	Toledo	Cobisa		394845N	0040157W	674	100
NC103948	E175	26/2	20130628	ESP	Castilla-La Mancha	Toledo	Cobisa		394845N	0040157W	674	100
NC103949	E175	26/3	20130628	ESP	Castilla-La Mancha	Toledo	Cobisa		394845N	0040157W	674	100
NC103950	E175	26/4	20130628	ESP	Castilla-La Mancha	Toledo	Cobisa		394845N	0040157W	674	100
NC103951	E175	27/1	20130628	ESP	Castilla-La Mancha	Toledo	Arges		394823N	0040343W	664	100
NC103952	E175	27/2	20130628	ESP	Castilla-La Mancha	Toledo	Arges		394823N	0040343W	664	100
NC103953	E175	28	20130628	ESP	Castilla-La Mancha	Toledo	Arges		394823N	0040343W	664	100
NC103954	E175	29	20130628	ESP	Castilla-La Mancha	Toledo	Lucillos	Arroyo de Ventalan	400116N	0043654W	430	100
NC103955	E175	30/1	20130628	ESP	Castilla-La Mancha	Toledo	Lucillos	Arroyo de Ventalan	400116N	0043654W	430	100
NC103956	E175	30/2	20130628	ESP	Castilla-La Mancha	Toledo	Lucillos	Arroyo de Ventalan	400116N	0043654W	430	100
NC103957	E175	31	20130628	ESP	Castilla-La Mancha	Toledo	Cazalegas		400045N	0044111W	396	100

NC103958	E175	32	20130628	ESP	Castilla-La Mancha	Toledo	Cazalegas		400045N	0044111W	396	100
NC103959	E175	33	20130628	ESP	Castilla-La Mancha	Toledo	San Roman de los Montes		400426N	0044353W	467	100
NC103960	E175	34	20130628	ESP	Castilla-La Mancha	Toledo	San Roman de los Montes		400428N	0044351W	483	100
NC103961	E175	35	20130708	ESP	Castilla-La Mancha	Ciudad Rea	Herencia		391913N	0032228W	654	100
NC103962	E175	36	20130708	ESP	Castilla-La Mancha	Ciudad Rea	Herencia		391915N	0032225W	655	100
NC103963	E175	37	20130708	ESP	Castilla-La Mancha	Ciudad Rea	Alhambra	N430	385439N	0030502W	803	100
NC103964	E175	38	20130708	ESP	Castilla-La Mancha	Ciudad Rea	Alhambra	Antes del pueblo en	385439N	0030502W	804	100
NC103965	E175	39	20130708	ESP	Castilla-La Mancha	Ciudad Rea	Alhambra	Arroyo de los Greda	385455N	0030642W	790	100
NC103966	E175	40	20130708	ESP	Castilla-La Mancha	Ciudad Rea	Villahermosa		384455N	0024440W	977	100
NC103967	E175	41/1	20130708	ESP	Castilla-La Mancha	Ciudad Rea	Villahermosa		384455N	0024440W	977	100
NC103968	E175	41/2	20130708	ESP	Castilla-La Mancha	Ciudad Rea	Villahermosa		384455N	0024440W	977	100
NC103969	E175	42	20130708	ESP	Castilla-La Mancha	Ciudad Rea	Villahermosa		384455N	0024440W	977	100
NC103970	E175	43	20130708	ESP	Castilla-La Mancha	Ciudad Rea	Villanueva de la Fuente		384113N	0024219W	1005	100
NC103971	E175	44	20130708	ESP	Castilla-La Mancha	Ciudad Rea	Villanueva de la Fuente		384113N	0024219W	1005	100
NC103972	E175	45	20130708	ESP	Castilla-La Mancha	Ciudad Rea	Puebla del Principe	Ermita de la Mairen	383255N	0025538W	944	100
NC103973	E175	46	20130708	ESP	Castilla-La Mancha	Ciudad Rea	Puebla del Principe	Ermita de la Mairen	383255N	0025538W	944	100
NC103974	E175	47	20130708	ESP	Castilla-La Mancha	Ciudad Rea	Puebla del Principe	Ermita de la Mairen	383255N	0025538W	944	100
NC103975	E175	48	20130708	ESP	Castilla-La Mancha	Ciudad Rea	Cozar	Rio Jabalon	384147N	0030312W	819	100
NC103976	E175	49	20130708	ESP	Castilla-La Mancha	Ciudad Rea	Cozar	Rio Jabalon	384147N	0030312W	819	100
NC103977	E175	50	20130708	ESP	Castilla-La Mancha	Ciudad Rea	Cozar	Rio Jabalon	384147N	0030312W	819	100
NC103978	E175	51	20130709	ESP	Castilla-La Mancha	Ciudad Rea	Alcubillas		384514N	0030733W	813	100
NC103979	E175	52	20130709	ESP	Castilla-La Mancha	Ciudad Rea	Alcubillas		384514N	0030733W	813	100
NC103980	E175	53	20130709	ESP	Castilla-La Mancha	Ciudad Rea	Bolaños de Calatrava		385327N	0033825W	667	100
NC103981	E175	54	20130709	ESP	Castilla-La Mancha	Ciudad Rea	Bolaños de Calatrava		385327N	0033825W	667	100
NC103982	E175	55	20130709	ESP	Castilla-La Mancha	Ciudad Rea	Bolaños de Calatrava		385327N	0033825W	667	100
NC103983	E175	56	20130709	ESP	Castilla-La Mancha	Ciudad Rea	Carrion de Calatrava	Crta. Carrion de Cal	390509N	0035200W	616	100
NC103984	E175	57	20130709	ESP	Castilla-La Mancha	Ciudad Rea	Carrion de Calatrava	Crta. Carrion de Cal	390509N	0035200W	616	100
NC103985	E175	58	20130709	ESP	Castilla-La Mancha	Ciudad Rea	Villarrubia de los Ojos		391237N	0033739W	629	100
NC103986	E175	59	20130709	ESP	Castilla-La Mancha	Ciudad Rea	Villarrubia de los Ojos		391237N	0033739W	629	100
NC103987	C549	121	201207--	ESP	Extremadura	Badajoz		BA-27 Km 12. Higue	382108N	0055457W	572	100
NC103988	C549	122	201207--	ESP	Extremadura	Badajoz		BA-27 Km 7. Higuer	381927N	0055244W	577	100
NC103989	C549	123	201207--	ESP	Extremadura	Badajoz		BA-27 Km 7. Higuer	381927N	0055244W	577	100
NC103990	C549	124	201207--	ESP	Extremadura	Badajoz		BA-27 Km 2,1.Higue	381726N	0055027W	604	100

NC103991	C549	125 A	201207--	ESP	Extremadura	Badajoz		Ctra. Badajoz/Alme	384948N	0063932W	246	100
NC103992	C549	126	201207--	ESP	Extremadura	Badajoz		Ctra. Badajoz/Alme	384948N	0063932W	246	100
NC103993	C549	127 A	201207--	ESP	Extremadura	Badajoz		Ctra. Badajoz/Alme	384815N	0063804W	257	100
NC103994	C549	127 B	201207--	ESP	Extremadura	Badajoz		Ctra. Badajoz/Alme	384815N	0063804W	257	100
NC103995	C549	128	201207--	ESP	Extremadura	Badajoz		Ctra. Badajoz/Alme	384815N	0063804W	257	100
NC103996	C549	129	201207--	ESP	Extremadura	Badajoz		Ctra. Solana de los	384329N	0063042W	258	100
NC103997	C549	130	201207--	ESP	Extremadura	Badajoz		Ctra. Solana de los	384329N	0063042W	258	100
NC103998	C549	131 A	201207--	ESP	Extremadura	Badajoz		EX-105. Km 82,4,sal	383701N	0063045W	313	100
NC103999	C549	131 B	201207--	ESP	Extremadura	Badajoz		EX-105. Km 82,4,sal	383701N	0063045W	313	100
NC104000	C549	132	201207--	ESP	Extremadura	Badajoz		N-432 Km 37. Badaj	383813N	0064110W	314	100
NC104001	C549	133	201207--	ESP	Extremadura	Badajoz		N-432 Km 37. Badaj	383813N	0064110W	314	100
NC104002	C549	134	201207--	ESP	Extremadura	Badajoz		N-432 Km 34. Badaj	383906N	0064255W	287	100
NC104003	C549	135	201207--	ESP	Extremadura	Badajoz		N-432 Km 34. Badaj	383906N	0064255W	287	100
NC104004	C549	136	201207--	ESP	Extremadura	Badajoz		N-432 Km 28,9. Bad	384043N	0064545W	301	100
NC104005	C549	137	201207--	ESP	Extremadura	Badajoz		Ctra. La Albuera/Va	384252N	0065049W	264	100
NC104006	C549	138	201207--	ESP	Extremadura	Badajoz		Ctra. La Albuera/Va	384252N	0065049W	264	100
NC104007	C549	139	201207--	ESP	Extremadura	Badajoz		Ctra. La Albuera/Va	384248N	0065525W	259	100
NC104008	C549	140	201207--	ESP	Extremadura	Badajoz		Ctra. La Albuera/Va	384121N	0065724W	264	100
NC104009	C549	141	201207--	ESP	Extremadura	Badajoz		BA-85 Km 7,2. Berla	381747N	0054943W	589	100
NC104010	C549	142	201207--	ESP	Extremadura	Badajoz		BA-85 Km 7,2. Berla	381747N	0054943W	589	100
NC104011	C549	143	201207--	ESP	Extremadura	Badajoz		BA-85 Km 5,6. Berla	381901N	0054931W	538	100
NC104012	C549	144	201207--	ESP	Extremadura	Badajoz		BA-85 Km 2,4. Berla	382040N	0054251W	562	100
NC104013	C549	145	201207--	ESP	Extremadura	Badajoz		BA-85 Km 2,4. Berla	382040N	0054251W	562	100
NC104014	C549	146	201207--	ESP	Extremadura	Badajoz		BA-19 Km 14,5. Ma	382110N	0054840W	557	100
NC104015	C549	147	201207--	ESP	Extremadura	Badajoz		BA-85 Km 8,1. Berla	381905N	0054532W	579	100
NC104016	C549	148 A	201207--	ESP	Extremadura	Badajoz		BA-85 Km 5,05. Ber	381742N	0054407W	594	100
NC104017	C549	148 B	201207--	ESP	Extremadura	Badajoz		BA-85 Km 5,05. Ber	381742N	0054407W	594	100
NC104018	C549	149	201207--	ESP	Extremadura	Badajoz		Ctra. Azuaga/Campi	381759N	0054157W	598	100
NC104019	C549	150	201207--	ESP	Extremadura	Badajoz		Ctra. Azuaga/Campi	382150N	0054325W	565	100
NC104020	C549	151	201207--	ESP	Extremadura	Badajoz		Ctra. Azuaga/Campi	382150N	0054325W	565	100
NC104021	C549	152	201207--	ESP	Extremadura	Badajoz		Ctra. Azuaga/Campi	382140N	0054230W	569	100
NC104022	C549	153	201207--	ESP	Extremadura	Badajoz		EX-111 Km 6,8. Azu	382016N	0054040W	578	100
NC104023	C549	154	201207--	ESP	Extremadura	Badajoz		EX-111 Km 6,8. Azu	382016N	0054040W	578	100

NC104024	C549	155	201207--	ESP	Extremadura	Badajoz		EX-111 Azuaga-Zala	381858N	0054050W	591	100
NC105771	C549	20 B	201207--	ESP	Extremadura	Badajoz	Azuaga	BA-18 Azuaga/Alani	381219N	0054047W	447	100
NC105772	C549	61 B	201207--	ESP	Extremadura	Badajoz	Llerena	EX-103 Llerena/Vale	381715N	0055903W	603	100
NC105773	C549	70 B	201207--	ESP	Extremadura	Badajoz	Berlanga	Camino viejo Magui	381859N	0054807W	551	100
NC105774	C549	92	201207--	ESP	Extremadura	Badajoz	Llera	BA-82 Llera/Usagre	382611N	0060443W	448	100
NC105775	C549	119	201207--	ESP	Extremadura	Badajoz	Higuera de Llerena	BA-27 Higuerade Lle	382157N	0055622W	582	100
NC105776	C549	99 A	201207--	ESP	Extremadura	Badajoz	Hinojosa del Valle	BA-141 Usagre7Hin	382713N	0061110W	451	100
NC105777	C549	101	201207--	ESP	Extremadura	Badajoz	Alange	Ctra. Almendralejo/	384140N	0061511W	340	100
NC105778	C549	1	201207--	ESP	Extremadura	Badajoz	Monesterio	EX-103 Entrada pan	380555N	0061853W	664	100
NC105779	C549	6	201207--	ESP	Extremadura	Badajoz	Castilblanco	N-502 Castilblanco/	391625N	0050507W	448	100
NC105780	C549	8	201207--	ESP	Extremadura	Badajoz	Herrera del Duque	N-502 Castilblanco/	391514N	0050354W	364	100
NC105781	C549	10 B	201207--	ESP	Extremadura	Badajoz	Garbayuela	BA-135 Desvio Fuer	390419N	0050222W	533	100
NC105782	C549	11	201207--	ESP	Extremadura	Badajoz	Monterrubio de la S	BA-51 Castuera/Mo	383559N	0052758W	564	100
NC105783	C549	12	201207--	ESP	Extremadura	Badajoz	Monterrubio de la S	EX-211 Monterrubi	383205N	0052912W	542	100
NC105784	C549	13	201207--	ESP	Extremadura	Badajoz	Peraleda del Zaucej	EX-211 Monterrubi	382959N	0053120W	545	100
NC105785	C549	14	201207--	ESP	Extremadura	Badajoz	Peraleda del Zaucej	BA-159 Peraleda de	382729N	0053352W	562	100
NC105786	C549	15	201207--	ESP	Extremadura	Badajoz	Peraleda del Zaucej	BA-159 Peraleda de	382625N	0053402W	553	100
NC105787	C549	16	201207--	ESP	Extremadura	Badajoz	Granja de Torreherr	Alrededores de Gra	381735N	0053508W	375	100
NC105788	C549	17	201207--	ESP	Extremadura	Badajoz	Granja de Torreherr	pista a Granja de To	381719N	0053353W	604	100
NC105789	C549	18	201207--	ESP	Extremadura	Badajoz	Granja de Torreherr	pista a Granja de To	381515N	0053246W	586	100
NC105790	C549	19	201207--	ESP	Extremadura	Badajoz	Azuaga	Tapias del cemente	381422N	0053302W	565	100
NC105791	C549	20 A	201207--	ESP	Extremadura	Badajoz	Azuaga	BA-18 Azuaga/Alani	381219N	0054047W	447	100
NC105792	C549	23	201207--	ESP	Extremadura	Badajoz	Malcocinado	Cerca de la finca El S	380611N	0054223W	638	100
NC105793	C549	25 A	201207--	ESP	Extremadura	Badajoz	Fuente del Arco	EX-200 Fuente del A	381030N	0055527W	680	100
NC105794	C549	28	201207--	ESP	Extremadura	Badajoz	Montemolin	EX-103 Llerena/Pall	380711N	0060827W	562	100
NC105795	C549	30	201207--	ESP	Extremadura	Badajoz	Monesterio	EX-103 Pallares/ver	380536N	0061525W	704	100
NC105796	C549	38 A	201207--	ESP	Extremadura	Badajoz	Jerez de los Caballe	EX-317 Encinasola/C	381551N	0065323W	341	100
NC105797	C549	41	201207--	ESP	Castilla y Leon	Salamanca	Zamayon	Ctra. Valdelosa/Zam	410942N	0054800W	853	100
NC105798	C549	47	201207--	ESP	Castilla y Leon	Salamanca	Topas	N-630 Salamanca/Z	410934N	0054024W	836	100
NC105799	C549	48	201207--	ESP	Castilla y Leon	Salamanca	Valdelosa	Proximo a Valdelosa	411009N	0054556W	804	100
NC105800	C549	49 A	201207--	ESP	Extremadura	Badajoz	Medina de las Torre	BA-160 Medina de l	381947N	0062558W	439	100
NC105801	C549	50	201207--	ESP	Extremadura	Badajoz	Medina de las Torre	Medina de las Torre	381821N	0062703W	406	100
NC105802	C549	51	201207--	ESP	Extremadura	Badajoz	Fuente de Cantos	Medina de las Torre	381317N	0062328W	570	100

NC105803	C549	53 A	201207--	ESP	Extremadura	Badajoz	Fuente de Cantos	EX-202 Medina de l	381342N	0062038W	546	100
NC105804	C549	54 A	201207--	ESP	Extremadura	Badajoz	Fuente de Cantos	EX-202 Fuente de C	381622N	0061522W	592	100
NC105805	C549	55 A	201207--	ESP	Extremadura	Badajoz	Bienvenida	Fuente de Cantos/B	381723N	0061322W	579	100
NC105806	C549	56	201207--	ESP	Extremadura	Badajoz	Bienvenida	EX-202 Bienvenida/	381934N	0061109W	598	100
NC105807	C549	58 A	201207--	ESP	Extremadura	Badajoz	Usagre	N-432 Badajoz/Gran	381914N	0060805W	593	100
NC105808	C549	59 A	201207--	ESP	Extremadura	Badajoz	Villagarcia de la Tor	N-432 Badajoz/Gran	381812N	0060636W	610	100
NC105809	C549	60	201207--	ESP	Extremadura	Badajoz	Villagarcia de la Tor	N-432 Badajoz/Gran	381628N	0060331W	637	100
NC105810	C549	61 A	201207--	ESP	Extremadura	Badajoz	Llerena	EX-103 Llerena/Vale	381715N	0055903W	603	100
NC105811	C549	62	201207--	ESP	Extremadura	Badajoz	Higuera de Llerena	EX-103 Llerena/Vale	381857N	0055810W	582	100
NC105812	C549	63	201207--	ESP	Extremadura	Badajoz	Higuera de Llerena	Ctra. Llerena/Magu	381938N	0055708W	584	100
NC105813	C549	64	201207--	ESP	Extremadura	Badajoz	Higuera de Llerena	Ctra. Llerena/Magu	382016N	0055544W	580	100
NC105814	C549	65	201207--	ESP	Extremadura	Badajoz	Higuera de Llerena	Ctra. Llerena/Magu	382026N	0055459W	578	100
NC105815	C549	67	201207--	ESP	Extremadura	Badajoz	Berlanga	Ctra. Llerena/Magu	382036N	0055244W	579	100
NC105816	C549	70 A	201207--	ESP	Extremadura	Badajoz	Berlanga	Camino viejo Magui	381859N	0054807W	551	100
NC105817	C549	71	201207--	ESP	Extremadura	Badajoz	Berlanga	Camino viejo Magui	381822N	0054831W	548	100
NC105818	C549	72	201207--	ESP	Extremadura	Badajoz	Berlanga	Camino viejo Magui	381728N	0054856W	555	100
NC105819	C549	74 A	201207--	ESP	Extremadura	Badajoz	Azuaga	Cruce N-432 y ctra.	381533N	0054310W	608	100
NC105820	C549	75	201207--	ESP	Extremadura	Badajoz	San Vicente de Alca	BA-133 San Vicente	392506N	0070329W	520	100
NC105821	C549	76	201207--	ESP	Extremadura	Caceres	Salorino	BA-133 San Vicente	392809N	0070044W	350	100
NC105822	C549	77	201207--	ESP	Extremadura	Caceres	Salorino	BA-133 San Vicente	393013N	0070127W	331	100
NC105823	C549	78	201207--	ESP	Extremadura	Caceres	Valencia de Alcanta	N-521 rio Alburrel K	392805N	0071225W	333	100
NC105824	C549	79	201207--	ESP	Extremadura	Caceres	Alcantara	Ctra. Alcantara/Mat	394300N	0065151W	276	100
NC105825	C549	82	201207--	ESP	Extremadura	Caceres	Mata de Alcantara	Ermita de San Loren	394210N	0064649W	389	100
NC105826	C549	86	201207--	ESP	Extremadura	Caceres	Monroy	EX-390 Torrejon el I	394147N	0060848W	445	100
NC105827	C549	89 A	201207--	ESP	Extremadura	Badajoz	Hinojosa del Valle	BA-131 Hinojosa de	382851N	0060950W	444	100
NC105828	C549	93	201207--	ESP	Extremadura	Badajoz	Usagre	BA-82 Llera/Usagre	382350N	0060733W	479	100
NC105829	C549	97 A	201207--	ESP	Extremadura	Badajoz	Usagre	BA-141 Usagre7Hin	382328N	0061006W	558	100
NC105830	C549	98	201207--	ESP	Extremadura	Badajoz	Hinojosa del Valle	BA-141 Usagre7Hin	382713N	0061110W	451	100
NC105831	C549	105	201207--	ESP	Extremadura	Badajoz	Puebla de la Reina	Ctra. Palomas/Horn	384046N	0060712W	355	100
NC105832	C549	106	201207--	ESP	Extremadura	Badajoz	Puebla de la Reina	Ctra. Puebla de la R	383800N	0060622W	414	100
NC105833	C549	110	201207--	ESP	Extremadura	Badajoz	Usagre	EX-202 Usagre/Vale	382330N	0060614W	483	100
NC105834	C549	115	201207--	ESP	Extremadura	Badajoz	Valencia de las Torr	EX-202 Usagre/Vale	382345N	0060135W	492	100
NC105835	C549	116 A	201207--	ESP	Extremadura	Badajoz	Higuera de Llerena	Valencia de las Torr	382253N	0055957W	531	100

NC105836	E204	AL-1	20120618	ESP	Andalucia	Cordoba	Almodovar del Rio		374911N	0050131W	6	100
NC105837	E204	AL-2	20120618	ESP	Andalucia	Cordoba	Almodovar del Rio		375052N	0050106W	48	100
NC106650	C555	1	20130606	ESP	Andalucia	Cordoba	Cordoba		375329N	0044925W	183	100
NC106651	C555	2	20130606	ESP	Andalucia	Cordoba	Cordoba		375314N	0044943W	193	100
NC106652	C555	3	20130606	ESP	Andalucia	Cordoba	Cordoba		375312N	0044947W	195	100
NC106653	C555	5	20130606	ESP	Andalucia	Cordoba	Cordoba		375301N	0045024W	186	100
NC106654	C555	6	20130608	ESP	Andalucia	Cordoba	Cordoba		375224N	0043448W	228	100
NC106655	C555	7	20130606	ESP	Andalucia	Cordoba	Cordoba		375217N	0045358W	189	100
NC106656	C555	8	20130612	ESP	Andalucia	Cordoba	Almodovar del Rio		375110N	0045957W	264	100
NC106657	C555	9	20130612	ESP	Andalucia	Cordoba	Almodovar del Rio		375141N	0045935W	332	100
NC106658	C555	10	20130612	ESP	Andalucia	Cordoba	Almodovar del Rio		375148N	0045928W	314	100
NC106659	C555	12	20130612	ESP	Andalucia	Cordoba	Almodovar del Rio		375158N	0045919W	320	100
NC106660	C555	13	20130612	ESP	Andalucia	Cordoba	Almodovar del Rio		375205N	0045916W	323	100
NC106661	C555	14	20130612	ESP	Andalucia	Cordoba	Almodovar del Rio		375211N	0045910W	328	100
NC106662	C555	15	20130612	ESP	Andalucia	Cordoba	Almodovar del Rio		375218N	0045906W	339	100
NC106663	C555	16/1	20130612	ESP	Andalucia	Sevilla	Ecija		373626N	0045804W	356	100
NC106664	C555	17	201307--	ESP	Andalucia	Granada	Moclin		372318N	0034818W	794	100
NC106665	C555	18	201307--	ESP	Andalucia	Granada	Moclin		372318N	0034818W	794	100
NC106666	C555	19	20130618	ESP	Andalucia	Cordoba	Cordoba		375646N	0043823W	176	100
NC106667	C555	20	20130618	ESP	Andalucia	Cordoba	Cordoba		375743N	0043809W	207	100
NC106668	C555	21	20130618	ESP	Andalucia	Cordoba	Cordoba		375850N	0043909W	224	100
NC106669	C555	23	20130618	ESP	Andalucia	Cordoba	Cordoba		375538N	0044246W		100
NC106670	C555	24	201307--	ESP	Andalucia	Granada	Dilar		370448N	0033629W	846	100
NC106671	C555	25	20130618	ESP	Andalucia	Cordoba	Cordoba		375537N	0044243W	192	100
NC106672	C555	26	20130618	ESP	Andalucia	Cordoba	Cordoba		375539N	0044246W	193	100
NC106673	C555	27	20130618	ESP	Andalucia	Cordoba	Cordoba		375513N	0044252W	190	100
NC106674	C555	28	20130618	ESP	Andalucia	Cordoba	Cordoba		375528N	0044336W	188	100
NC106675	C555	29	20130618	ESP	Andalucia	Cordoba	Cordoba		375412N	0044410W	129	100
NC106676	C555	30	20130618	ESP	Andalucia	Cordoba	Cordoba		375505N	0044410W	191	100
NC106677	C555	31	20130605	ESP	Andalucia	Cordoba	Posadas		375108N	0050642W	177	100
NC106678	C555	32	20130605	ESP	Andalucia	Cordoba	Posadas		375108N	0050642W	177	100
NC106679	C555	33	20130605	ESP	Andalucia	Cordoba	Posadas		375306N	0050637W	321	100
NC106680	C555	34	20130605	ESP	Andalucia	Cordoba	Posadas		375139N	0050633W	228	100

NC106681	C555	35	20130605	ESP	Andalucia	Cordoba	Villaviciosa de Cordoba		375640N	0050440W	525	100
NC106682	C555	36	20130605	ESP	Andalucia	Cordoba	Villaviciosa de Cordoba		375640N	0050440W	525	100
NC106683	C555	37	20130605	ESP	Andalucia	Cordoba	Villaviciosa de Cordoba		375836N	0050520W	506	100
NC106684	C555	38	20130605	ESP	Andalucia	Cordoba	Villaviciosa de Cordoba		375836N	0050520W	506	100
NC106685	C555	39	20130605	ESP	Andalucia	Cordoba	Villaviciosa de Cordoba		380014N	0050357W	479	100
NC106686	C555	40	20130605	ESP	Andalucia	Sevilla	Fuentes de Andalucia		373104N	0051950W	164	100
NC106687	C555	41	20130605	ESP	Andalucia	Cordoba	Villaviciosa de Cordoba		375847N	0050104W		100
NC106688	C555	42	20130605	ESP	Andalucia	Cordoba	Cordoba		375644N	0045756W		100
NC106689	C555	43	20130605	ESP	Andalucia	Cordoba	Cordoba		375621N	0045442W		100
NC106690	C555	44	201308--	ESP	Andalucia	Granada	Diezma		371911N	0031917W	1180	100
NC106691	C555	45	201308--	ESP	Andalucia	Granada	Gor		372242N	0025917W	1178	100
NC106692	C555	46	201308--	ESP	Andalucia	Granada	Dilar		370456N	0033541W	860	100
NC106693	C555	47	201308--	ESP	Andalucia	Granada	Diezma		371911N	0031917W	1180	100
NC106694	C555	48	201308--	ESP	Andalucia	Granada	Baza	Baul	372843N	0025605W	10339	100
NC106989	C549	2	201207--	ESP	Extremadura	Badajoz	Talarrubias	Embalse Garcia Sola	391017N	0051347W	372	100
NC106990	C549	7	201207--	ESP	Extremadura	Badajoz	Castilblanco	N-502 Castilblanco/	391625N	0050507W	448	100
NC106991	C549	9	201207--	ESP	Extremadura	Badajoz	Herrera del Duque	N-502 Herrera del D	390951N	0050007W	455	100
NC106992	C549	21	201207--	ESP	Andalucia	Sevilla	Guadalcanal	BA-18 Azuaga/Alani	380948N	0054107W	529	100
NC106993	C549	22	201207--	ESP	Extremadura	Badajoz	Malcocinado	BA-18 Azuaga/Alani	380610N	0054223W	638	100
NC106994	C549	24	201207--	ESP	Andalucia	Sevilla	Guadalcanal	BA-18 Desvío Alanis	380435N	0054355W	731	100
NC106995	C549	25 B	201207--	ESP	Extremadura	Badajoz	Fuente del Arco	EX-200 Fuente del A	381030N	0055527W	680	100
NC106996	C549	31	201207--	ESP	Extremadura	Badajoz	Cabeza la Vaca	EX-103 Km 217 Cale	380543N	0062145W	637	100
NC106997	C549	34	201207--	ESP	Extremadura	Badajoz	Segura de Leon	EX-201 Km 38 Segu	380747N	0063220W	645	100
NC106998	C549	35	201207--	ESP	Extremadura	Badajoz	Bodonal de la Sierra	EX-201 Km 44 Bodo	380937N	0063526W	620	100
NC106999	C549	36	201207--	ESP	Extremadura	Badajoz	Bodonal de la Sierra	EX-201 Km 44 Bodo	380951N	0063510W	622	100
NC107000	C549	37	201207--	ESP	Extremadura	Badajoz	Higuera la Real	Ctra. Higuera La Rea	380832N	0064318W	612	100
NC107001	C549	38 B	201207--	ESP	Extremadura	Badajoz	Jerez de los Caballe	EX-317 Encinasola/(	381551N	0065323W	341	100
NC107002	C549	40	201207--	ESP	Castilla y Leon	Salamanca	Ciudad Rodrigo	Ctra. Sancti-Spiritus	403921N	0062702W	773	100
NC107003	C549	42	201207--	ESP	Castilla y Leon	Salamanca	Zamayon	Ctra. Valdelosa/Zam	410924N	0054828W	840	100
NC107004	C549	43	201207--	ESP	Castilla y Leon	Salamanca	Pedrosillo de los Air	Ctra. La Maya/Pedr	404219N	0054007W	875	100
NC107005	C549	44	201207--	ESP	Castilla y Leon	Salamanca	San Pedro de Rozad	Ctra. Salamanca/Ve	405030N	0054752W	866	100
NC107006	C549	45	201207--	ESP	Castilla y Leon	Salamanca		Ctra. Salamanca/Vecinos Km 2				100
NC107007	C549	46 A	201207--	ESP	Castilla y Leon	Salamanca	Cabrillas	Ctra. Tamames-La F	404333N	0060922W	821	100

NC107008	C549	49 B	201207--	ESP	Extremadura	Badajoz	Medina de las Torre	BA-160 Medina de l	381947N	0062558W	439	100
NC107009	C549	52 A	201207--	ESP	Extremadura	Badajoz	Fuente de Cantos	EX-202 Medina de l	381316N	0062149W	496	100
NC107010	C549	52 B	201207--	ESP	Extremadura	Badajoz	Fuente de Cantos	EX-202 Medina de l	381316N	0062149W	496	100
NC107011	C549	53 B	201207--	ESP	Extremadura	Badajoz	Fuente de Cantos	EX-202 Medina de l	381316N	0062149W	496	100
NC107012	C549	54 B	201207--	ESP	Extremadura	Badajoz	Fuente de Cantos	EX-202 Fuente de C	381622N	0061522W	592	100
NC107013	C549	55 B	201207--	ESP	Extremadura	Badajoz	Bienvenida	Fuente de Cantos/B	381723N	0061322W	579	100
NC107014	C549	57 A	201207--	ESP	Extremadura	Badajoz	Usagre	N-432 Badajoz/Gran	382023N	0060943W	588	100
NC107015	C549	57 B	201207--	ESP	Extremadura	Badajoz	Usagre	N-432 Badajoz/Gran	382023N	0060943W	588	100
NC107016	C549	57 C	201207--	ESP	Extremadura	Badajoz	Usagre	N-432 Badajoz/Gran	382023N	0060943W	588	100
NC107017	C549	58 B	201207--	ESP	Extremadura	Badajoz	Usagre	N-432 Badajoz/Gran	381914N	0060805W	593	100
NC107018	C549	59 B	201207--	ESP	Extremadura	Badajoz	Villagarcia de la Tor	N-432 Badajoz/Gran	381812N	0060636W	610	100
NC107019	C549	66	201207--	ESP	Extremadura	Badajoz	Higuera de Llerena	Cruce de las ctras. L	382009N	0055345W	524	100
NC107020	C549	73 A	201207--	ESP	Extremadura	Badajoz	Azuaga	N-432 Berlanga/Azu	381558N	0054551W	609	100
NC107021	C549	73 B	201207--	ESP	Extremadura	Badajoz	Azuaga	N-432 Berlanga/Azu	381558N	0054551W	609	100
NC107022	C549	74 B	201207--	ESP	Extremadura	Badajoz	Azuaga	Cruce N-432 y ctra.	381533N	0054310W	608	100
NC107023	C549	84	201207--	ESP	Extremadura	Caceres	Malpartida de Plase	Salida de Malpartida	395837N	0060304W	440	100
NC107024	C549	85	201207--	ESP	Extremadura	Caceres		EX-390 Torrejón el Rubio/Caceres Km 38,2				100
NC107025	C549	87	201207--	ESP	Extremadura	Caceres	Monroy	Monroy/Trujillo. Pu	393646N	0061317W	412	100
NC107026	C549	88	201207--	ESP	Extremadura	Caceres	Serradilla	Serradilla/Torrejón	394804N	0060754W	399	100
NC107027	C549	91	201207--	ESP	Extremadura	Badajoz	Llera	BA-82 Llera/Usagre	382611N	0060443W	448	100
NC107028	C549	94 A	201207--	ESP	Extremadura	Badajoz	Usagre	BA-82 Llera/Usagre	382236N	0060809W	548	100
NC107029	C549	95	201207--	ESP	Extremadura	Badajoz	Usagre	BA-141 Usagre/Hino	382228N	0060952W	566	100
NC107030	C549	96	201207--	ESP	Extremadura	Badajoz	Usagre	BA-141 Usagre/Hino	382328N	0061006W	558	100
NC107031	C549	107	201207--	ESP	Extremadura	Badajoz	Puebla de la Reina	Ctra. Puebla de la R	383800N	0060622W	414	100
NC107032	C549	108	201207--	ESP	Extremadura	Badajoz	Llera	BA-80 Hornachos/L	382727N	0060419W	488	100
NC107033	C549	109	201207--	ESP	Extremadura	Badajoz	Llera	BA-80 Hornachos/L	382727N	0060419W	488	100
NC107034	C549	111	201207--	ESP	Extremadura	Badajoz	Usagre	EX-202 Usagre-Vale	382330N	0060614W	483	100
NC107035	C549	114	201207--	ESP	Extremadura	Badajoz	Valencia de las Torr	EX-202 Usagre-Vale	382345N	0060135W	492	100
NC107036	C549	116 B	201207--	ESP	Extremadura	Badajoz	Higuera de Llerena	Valencia de las Torr	382253N	0055957W	531	100
NC107037	C549	117 B	201207--	ESP	Extremadura	Badajoz	Higuera de Llerena	BA-27 Higuera de Ll	382214N	0055750W	583	100
NC107038	C549	118	201207--	ESP	Extremadura	Badajoz	Higuera de Llerena	BA-27 Higuera/Berla	382157N	0055622W	582	100
NC107039	C549	81	201207--	ESP	Extremadura	Caceres	Mata de Alcantara	Ermita de San Loren	394210N	0064649W	389	100
NC107040	C549	99 B	201207--	ESP	Extremadura	Badajoz	Hinojosa del Valle	BA-141 Usagre/Hino	382713N	0061110W	451	100

NC107060	C549	5	201207--	ESP	Extremadura	Badajoz	Castilblanco	N-502 Castilblanco/	391625N	0050507W	448	100
NC107541	C555	16/2	20130612	ESP	Andalucia	Sevilla	Ecija		373626N	0045804W	356	100
NC107542	C005	442G/2	199110--	ESP	Andalucia	Jaen	Frailes		3729--N	00350--W	973	100
