# Supplementary material for: Identification of ecogeographical gaps in the Spanish Aegilops collections with potential tolerance to drought and salinity
Source: PeerJ. 2017 Jul 27;5:e3494. doi: 10.7717/peerj.3494 (PMC5534164; doi:10.7717/peerj.3494)
Supplement: Supplemental Information 2 [file peerj-05-3494-s016.pdf]

Date collected	Institution code	Catalogue No	Basis of record	Scientific name	Author	Country	Country (interpreted)	Locality	County	State/Province	Latitude	Longitude	Min altitude
	USA029	1217305	Unknown	Aegilops geniculata		ESP	Spain				39.8333	-4	
	FUND. BIODIVER	1648482	Unknown	Aegilops geniculata Roth	Roth	ESP	Spain	Iglesuela del Cid, Pobla del Bellesta		Te	40.1	-0.1	
00:00.0	REDIAM-CMA	257301	Observation	Aegilops geniculata		ESP	Spain		Polopos	Gr	36.7929	-3.28938	870
00:00.0	REDIAM-CMA	288429	Observation	Aegilops geniculata		ESP	Spain		AlmerÃ-a	Al	36.8478	-2.33234	27
	SIVIM	T-P30031:Aegi	Observation	Aegilops geniculata Roth	Roth	ES	Spain	Bajada a Villa de Ves, Villa de Ves, Albace			39.19	-1.26	0
	SIVIM	T-P30056:Aegi	Observation	Aegilops geniculata Roth	Roth	ES	Spain	Los Callejones, Cofrentes, Valencia			39.19	-1.26	0
	SIVIM	T-P31975:Aegi	Observation	Aegilops geniculata Roth	Roth	ES	Spain	Campillo Arenas (Loma PÃ©rez)			37.49	-3.67	1500
	SIVIM	U-P02803:Aegi	Observation	Aegilops geniculata Roth	Roth	ES	Spain	Castells de Serrella			38.63	-0.24	0
	SIVIM	U-P06640:Aegi	Observation	Aegilops geniculata Roth	Roth	ES	Spain	S. RomÃ£o			37.13	-7.98	240
	SIVIM	U-P06751:Aegi	Observation	Aegilops geniculata Roth	Roth	ES	Spain	Rocha da Pena			37.22	-8.21	0
	RUS001	VIR100602123	Specimen	Aegilops ovata L.		ESP	Spain						
	RUS001	VIR100602102	Specimen	Aegilops ovata L.		ESP	Spain						
	RUS001	VIR100602081	Specimen	Aegilops ovata L.		ESP	Spain						
00:00.0	FUND. BIODIVER	1946404	Unknown	Aegilops geniculata Roth	Roth	ESP	Spain	Albadalejo		CR	38.1	-2.1	900
00:00.0	REDIAM-CMA	4601	Observation	Aegilops geniculata		ESP	Spain		MorÃ³n de la Fron	Se	37.0514	-5.29854	427
00:00.0	REDIAM-CMA	409877	Observation	Aegilops geniculata		ESP	Spain		Torres de AlbÃ¡nc	J	38.4387	-2.68619	1036
00:00.0	REDIAM-CMA	416811	Observation	Aegilops geniculata		ESP	Spain		Tabernas	Al	37.0822	-2.34883	486
	SIVIM	Q-P03045:Aegi	Observation	Aegilops geniculata Roth	Roth	ES	Spain	La Llacuna, camp abandonat entre la carre			41.45	1.44	0
	SIVIM	Q-P03610:Aegi	Observation	Aegilops geniculata Roth	Roth	ES	Spain	Villanueva de Viver			39.99	-0.65	0
	SIVIM	Q-P06904:Aegi	Observation	Aegilops geniculata Roth	Roth	ES	Spain	Subida a la Silla del Cid, Petrer,			38.46	-0.82	0
	SIVIM	R-P02650:Aegi	Observation	Aegilops geniculata Roth	Roth	ES	Spain	Utxesa, a prop de la bassa Espartera			41.43	0.48	160
	SIVIM	R-P06811:Aegi	Observation	Aegilops geniculata Roth	Roth	ES	Spain	Fuensanta, cerro del Gato, Albacete			39.2	-2.07	0
	SIVIM	R-P08524:Aegi	Observation	Aegilops geniculata Roth	Roth	ES	Spain	El Calabrial			36.86	-2.77	140
00:00.0	GDA	GDA30015-1	Specimen	Aegilops ovata L.	L.	ES	Spain	Salamanca, Monte de la Orbada.		SA			0
00:00.0	SEV	99073-1	Specimen	Aegilops geniculata Roth	Roth	ES	Spain	AznalcÃ¡zar. Marisma Gallega		Se			1
	MGC	27261-1	Unknown	Aegilops geniculata Roth	Roth	ES	Spain	Grazalema; Puerto de las Palomas		Ca	0	0	1
	RUS001	VIR100602218	Specimen	Aegilops ovata L.		ESP	Spain						
	FUND. BIODIVER	1043752	Unknown	Aegilops geniculata Roth	Roth	ESP	Spain	Antequera, Torcal		Ma	36.1	-4.1	
	FUND. BIODIVER	1835082	Unknown	Aegilops geniculata Roth	Roth	ESP	Spain	Puerto de la Caracollera		CR	38.1	-4.1	
	FUND. BIODIVER	1888170	Unknown	Aegilops geniculata Roth	Roth	ESP	Spain	Sierra del Caballon		V	39.1	-0.1	
00:00.0	SALA	18743-1	Specimen	Aegilops ovata L. subsp. triaristat	(Willd.) Rou	ES	Spain	_; Garcirrey, Vilvis		Sa			
00:00.0	REDIAM-CMA	386312	Observation	Aegilops geniculata		ESP	Spain		Pruna	Se	37.024	-5.20414	595
00:00.0	REDIAM-CMA	387537	Observation	Aegilops geniculata		ESP	Spain		Guillena	Se	37.5761	-6.0633	99
00:00.0	REDIAM-CMA	388810	Observation	Aegilops geniculata		ESP	Spain		Torres	J	37.7453	-3.5511	1291
00:00.0	REDIAM-CMA	392135	Observation	Aegilops geniculata		ESP	Spain		Antequera	Ma	36.9658	-4.75974	382
	SIVIM	P-P08867:Aegi	Observation	Aegilops geniculata Roth	Roth	ES	Spain	Mas de la Caramassa; H. St. Joan			40.88	0.26	0
	SIVIM	P-P08902:Aegi	Observation	Aegilops geniculata Roth	Roth	ES	Spain	Rodalies de Fredes			40.61	0.16	1110

	SIVIM	P-P09120:Aegi	Observation	Aegilops geniculata Roth	Roth	ES	Spain	Els Garrigons; Beseit			40.79	0.15	0
	SIVIM	P-P11341:Aegi	Observation	Aegilops geniculata Roth	Roth	ES	Spain	Finca de l''Abogat'			40.62	0.39	0
	SIVIM	Q-P01674:Aegi	Observation	Aegilops geniculata Roth	Roth	ES	Spain	Bellida, SacaÃ±et			39.81	-0.78	1300
00:00.0	COFC	41270-1	Specimen	Aegilops geniculata Roth	Roth	ES	Spain	Cabra; arroyo Pozas		Co	37	-4	1
00:00.0	COFC	41391-1	Specimen	Aegilops geniculata Roth	Roth	ES	Spain	Lucena; venta 'La Camila'		Co	37	-4	1
00:00.0	ABH	4848-1	Specimen	Aegilops geniculata Roth	Roth	ES	Spain	Fageca; Pla de la Casa		A	38.64	-0.36	
	BDBCV-General	275547	Observation	Aegilops geniculata		ESPA	Spain	Bolbaite	La Canal de Nava	Valenc	39.0517	-0.74664	
	BDBCV-General	279916	Observation	Aegilops geniculata		ESPA	Spain	Ayora	El Valle de Ayora	Valenc	38.9659	-0.98017	
00:00.0	GDA	GDA12220-1-2	Specimen	Aegilops geniculata Roth.	Roth.	ES	Spain	Granada, SÂª Nevada, CÃ¡Ã±ar, ba		GR			1100
	IDBD-GN	42496	Observation	Aegilops geniculata Roth	Roth	ES	Spain		Caparroso	Na	42.339	-1.65991	330
	GDA	GDA43468-1-3	Specimen	Aegilops geniculata Roth.	Roth.	ES	Spain	Granada, Guadix, Rambla Becerra.		GR			950
00:00.0	SALA	40754-1	Specimen	Aegilops geniculata Roth	Roth	ES	Spain	_; SepÃºlveda, Villaseca		Sg			
00:00.0	SALA	44302-1	Specimen	Aegilops geniculata Roth	Roth	ES	Spain	_; Maestu, Korres		Vi	42.7	-2.44	
00:00.0	COFC	41265-1	Specimen	Aegilops geniculata Roth	Roth	ES	Spain	Lucena; rÃƒÂo Lucena; entre Moril		Co	37	-4	1
00:00.0	HUAL	5970-1	Specimen	Aegilops geniculata Roth	Roth	ES	Spain	SÂª de GÃ¡dor, Las Navas		Al	36.87	-2.77	
	FUND. BIODIVER	1463979	Unknown	Aegilops geniculata Roth	Roth	ESP	Spain	AlÃ²s de Balaguer		L	41.1	0.1	300
	FUND. BIODIVER	1010574	Unknown	Aegilops ovata L.	L.	ESP	Spain	Casa de las Coronadas, MazarrÃ³n		Mu	37.1	-1.1	
00:00.0	FUND. BIODIVER	1774959	Unknown	Aegilops geniculata Roth	Roth	ESP	Spain	Monzon, cerro de Santa Quiteria, El		Hu	41.1	-0.9	365
	FUND. BIODIVER	1811878	Unknown	Aegilops geniculata Roth.	Roth.	ESP	Spain	Lumbier, Biezcas		Na	42.1	-1.1	440
	BC	867024	Specimen	Aegilops geniculata Roth	Roth	ES	Spain	Puerto Real; Calerones. S. Fernand		Ca	36.51	-6.04	
00:00.0	BC	867289	Specimen	Aegilops geniculata Roth	Roth	ES	Spain	Caldes de Malavella; Caldes		Ge	41.8218	2.759357	
00:00.0	REDIAM-CMA	136700	Observation	Aegilops geniculata		ESP	Spain		AlanÃs	Se	38.0121	-5.74394	600
00:00.0	REDIAM-CMA	145760	Observation	Aegilops geniculata		ESP	Spain		Casares	Ma	36.4346	-5.28075	200
00:00.0	REDIAM-CMA	187520	Observation	Aegilops geniculata		ESP	Spain		Oria	Al	37.497	-2.2674	1131
	DEU146	AE 396	Specimen	Aegilops geniculata Roth subsp. gibberosa (Zh		ESP	Spain						
00:00.0	COFC	50177-1	Specimen	Aegilops geniculata Roth	Roth	ES	Spain	Valle del Guadiato; casas 'Lagar de		Co			1
	DEU146	AE 687	Specimen	Aegilops geniculata Roth		ESP	Spain	Pyrenen					
00:00.0	REDIAM-CMA	331457	Observation	Aegilops geniculata		ESP	Spain		Obejo	Co	38.1319	-4.77752	530
00:00.0	REDIAM-CMA	380951	Observation	Aegilops geniculata		ESP	Spain		Vilches	J	38.1807	-3.52692	408
00:00.0	REDIAM-CMA	382523	Observation	Aegilops geniculata		ESP	Spain		MorÃ³n de la Fron	Se	37.0514	-5.29867	427
00:00.0	REDIAM-CMA	383223	Observation	Aegilops geniculata		ESP	Spain		El Gastor	Ca	36.8629	-5.31646	573
	SIVIM	T-P06439:Aegi	Observation	Aegilops geniculata Roth	Roth	ES	Spain	Casas de BenalÃ			39	-0.8	310
	SIVIM	T-P07216:Aegi	Observation	Aegilops geniculata Roth	Roth	ES	Spain	Purroy de la Solana			41.97	0.34	0
	SIVIM	T-P09188:Aegi	Observation	Aegilops geniculata Roth	Roth	ES	Spain	Tudela de Duero			41.53	-4.67	0
	SIVIM	T-P09796:Aegi	Observation	Aegilops geniculata Roth	Roth	ES	Spain	Los YÃ©benes, Toledo			39.56	-3.93	0
	SIVIM	T-P11409:Aegi	Observation	Aegilops geniculata Roth	Roth	ES	Spain	Valle de Valdemeca			40.19	-1.82	1350
	SIVIM	T-P13170:Aegi	Observation	Aegilops geniculata Roth	Roth	ES	Spain	Cicujano			42.72	-2.51	0
	SIVIM	U-P08150:Aegi	Observation	Aegilops geniculata Roth	Roth	ES	Spain	La Nava, Berzocana			39.36	-5.55	0
	SIVIM	U-P13295:Aegi	Observation	Aegilops geniculata Roth	Roth	ES	Spain	En Ctra. Arjonilla-Marmolejo, Km. 6			37.94	-4.13	280
00:00.0	L	766598	Specimen	Aegilops ovata L.		Spain	Spain	Rubi					
00:00.0	SEV	11253-1	Specimen	Aegilops geniculata Roth	Roth	ES	Spain	Pedrosa del Principe		Bu			1

00:00.0	SEV	101370-1	Specimen	Aegilops geniculata Roth	Roth	ES	Spain	Entre MorÃ³n y VillamartÃn		Se			1
00:00.0	SEV	108084-1	Specimen	Aegilops geniculata Roth	Roth	ES	Spain	Alrededores de Baza		Gr			1
00:00.0	SEV	108256-1	Specimen	Aegilops geniculata Roth	Roth	ES	Spain	A 3 Km al Oeste de Cervera		L			1
00:00.0	COA	41157-1	Specimen	Aegilops geniculata Roth	Roth	ES	Spain	Km 6 de Alcaracejos a El Viso		Co	38.38	-5.06	
00:00.0	FUND. BIODIVER	1531728	Unknown	Aegilops ovata		ESP	Spain	camino de regreso de Quintanilla de		S	42.1	-3.1	
	FUND. BIODIVER	1551967	Unknown	Aegilops ovata L.	L.	ESP	Spain	Aranda		Bu	41.1	-3.1	
	FUND. BIODIVER	1620398	Unknown	Aegilops geniculata Roth	Roth	ESP	Spain	MiÃ±era de Luna		Le	42.1	-5.1	
	FUND. BIODIVER	993536	Unknown	Aegilops geniculata		ESP	Spain	ValdepiÃ©lagos		Gu	40.1	-3.1	
00:00.0	REDIAM-CMA	188964	Observation	Aegilops geniculata		ESP	Spain		Tabernas	Al	37.075	-2.34733	452
00:00.0	REDIAM-CMA	220042	Observation	Aegilops geniculata		ESP	Spain		Baza	Gr	37.2386	-2.72849	1911
00:00.0	REDIAM-CMA	230579	Observation	Aegilops geniculata		ESP	Spain		AlhendÃn	Gr	37.0147	-3.7551	1129
	DEU146	AE 587	Specimen	Aegilops geniculata Roth		ESP	Spain	Gelnde von El Encin bei Alcal de Henares					
	SIVIM	T-P13700:Aegi	Observation	Aegilops geniculata Roth	Roth	ES	Spain	Pista a Tarayuela, Cantavieja			40.44	-0.52	1380
	SIVIM	T-P16906:Aegi	Observation	Aegilops geniculata Roth	Roth	ES	Spain	Loeches			40.37	-3.47	0
	SIVIM	T-P19691:Aegi	Observation	Aegilops geniculata Roth	Roth	ES	Spain	Baza, pr. Dos Picos, Loma de la Piedra			37.13	-2.77	1930
	SIVIM	T-P21418:Aegi	Observation	Aegilops geniculata Roth	Roth	ES	Spain	Monterrubio de la ArmuÃ±a			40.97	-5.73	0
	SIVIM	T-P27295:Aegi	Observation	Aegilops geniculata Roth	Roth	ES	Spain	Colina del castillo de Tornos			40.91	-1.45	1040
	SIVIM	T-P28635:Aegi	Observation	Aegilops geniculata Roth	Roth	ES	Spain	BaldÃos de TorrejÃ³n el Rubio			39.71	-6.08	330
00:00.0	COFC	11985-1	Specimen	Aegilops geniculata Roth	Roth	ES	Spain	Almedinilla; Sierra de Albayate		Co	37	-4	1
00:00.0	SEV	108252-1	Specimen	Aegilops geniculata Roth	Roth	ES	Spain	Entre Vich y Moya. Subida al Puerto		B			1
00:00.0	UNEX	05899-1	Observation	Aegilops geniculata Roth	_	ESP	Spain	GuareÃ±a: GuareÃ±a, olivares prÃ³		Ba	38.8	-6.1	
00:00.0	UNEX	26995-1	Observation	Aegilops geniculata Roth	_	ESP	Spain	Magacela: Sustrato calizo. CercanÃ		Ba	38.8	-5.8	
	MUB	102374-1	Specimen	Aegilops geniculata Roth	Roth	ES	Spain	Yecla; Monte ArabÃ		Mu	38.7	-1.33	600
	FUND. BIODIVER	1336000	Unknown	Aegilops geniculata Roth	Roth	ESP	Spain	OrÃsoain, Valdorba, camino a San		Na	42.1	-1.1	700
	FUND. BIODIVER	1354558	Unknown	Aegilops geniculata Roth	Roth	ESP	Spain	Campohermoso		Le	42.1	-5.1	
	FUND. BIODIVER	1366002	Unknown	Aegilops geniculata Roth	Roth	ESP	Spain	Castillo Nuevo, Barranco de la Sierr		Na	42.1	-0.1	1000
	FUND. BIODIVER	78788	Unknown	Aegilops geniculata Roth.	Roth.	ESP	Spain	Mojados		Va	41.1	-4.1	
	FUND. BIODIVER	83531	Unknown	Aegilops geniculata Roth	Roth	ESP	Spain	TÃ¡bara, La BreÃ±ica		Za	41.1	-5.1	
	FUND. BIODIVER	86617	Unknown	Aegilops geniculata		ESP	Spain	Aldehuela de PeriÃ¡Ã±ez, hacia Alm		So	41.1	-2.1	
	SIVIM	S-P09930:Aegi	Observation	Aegilops geniculata Roth	Roth	ES	Spain	serra Aitana, prop del pou de neu de la fon			38.63	-0.35	1360
	SIVIM	S-P13996:Aegi	Observation	Aegilops geniculata Roth	Roth	ES	Spain	Pinhal da Senhora (Freixo do Meio)			39.38	-8.88	132
	SIVIM	T-P01864:Aegi	Observation	Aegilops geniculata Roth	Roth	ES	Spain	Illa Grossa, prop de la Vila d'Eivissa			38.83	1.38	10
	SIVIM	T-P03849:Aegi	Observation	Aegilops geniculata Roth	Roth	ES	Spain	Pto. Altos de AyllÃ³n			41.37	-3.35	1080
	SIVIM	T-P05776:Aegi	Observation	Aegilops geniculata Roth	Roth	ES	Spain	HondÃ³n de las Nieves			38.19	-0.94	0
	SIVIM	T-P06425:Aegi	Observation	Aegilops geniculata Roth	Roth	ES	Spain	Sot de Chera			39.55	-1.02	0
	IPK	31969	Living	Aegilops geniculata Roth		ESP	Spain	Nocito Espagne					
	FUND. BIODIVER	1573681	Unknown	Aegilops geniculata Roth	Roth	ESP	Spain	proximidades de Celada de Roblece		P	42.1	-4.1	
	FUND. BIODIVER	1605287	Unknown	Aegilops ovata		ESP	Spain	Elecha		P			
	FUND. BIODIVER	1643502	Unknown	Aegilops geniculata Roth	Roth	ESP	Spain	Villamayor, Cuesta de Santa Isabel		Z	41.1	-0.1	300
	FUND. BIODIVER	992695	Unknown	Aegilops ovata L.	L.	ESP	Spain	Embid		Gu	40.1	-1.1	
00:00.0	PreservedSpecime	E00086654	Unknown	Aegilops geniculata Roth		ES	Spain						

	FUND. BIODIVER	1643501	Unknown	Aegilops geniculata Roth	Roth	ESP	Spain	Alfocea		Z	41.1	-0.1	280
	FUND. BIODIVER	992694	Unknown	Aegilops ovata L.	L.	ESP	Spain	Turmiel		Gu	40.1	-1.1	
00:00.0	REDIAM-CMA	216451	Observation	Aegilops geniculata		ESP	Spain		Gor	Gr	37.3766	-2.93793	1392
00:00.0	REDIAM-CMA	230355	Observation	Aegilops geniculata		ESP	Spain		DalÃ-as	Al	36.8587	-2.77653	1500
00:00.0	REDIAM-CMA	239985	Observation	Aegilops geniculata		ESP	Spain		El Real de la Jara	Se	37.9406	-6.04852	685
00:00.0	MUB	110756-1	Specimen	Aegilops geniculata Roth	Roth	ES	Spain	Cartagena; El Calvario		Mu	37.583	-0.956	1
00:00.0	REDIAM-CMA	194388	Observation	Aegilops geniculata		ESP	Spain		Quesada	J	37.8164	-3.09821	716
00:00.0	REDIAM-CMA	202875	Observation	Aegilops geniculata		ESP	Spain		Obejo	Co	38.1044	-4.73328	350
00:00.0	REDIAM-CMA	229761	Observation	Aegilops geniculata		ESP	Spain		Pozo AlcÃ³n	J	37.7423	-2.9221	998
00:00.0	REDIAM-CMA	234602	Observation	Aegilops geniculata		ESP	Spain		Belmez	Co	38.255	-5.21906	494
	SIVIM	T-P13697:Aegi	Observation	Aegilops geniculata Roth	Roth	ES	Spain	La Pobla del Bellestar, Iglesuela del Cid			40.44	-0.4	1115
	SIVIM	T-P16887:Aegi	Observation	Aegilops geniculata Roth	Roth	ES	Spain	ValdenuÃ±o			40.73	-3.47	0
	SIVIM	T-P19318:Aegi	Observation	Aegilops geniculata Roth	Roth	ES	Spain	Sierra del RetÃ-n, ladera NE			36.1	-5.88	0
	SIVIM	T-P20880:Aegi	Observation	Aegilops geniculata Roth	Roth	ES	Spain	La Loma, AlmerÃ-a			36.77	-2.55	0
	SIVIM	T-P26238:Aegi	Observation	Aegilops geniculata Roth	Roth	ES	Spain	RincÃ³n de los Huertos, Moratlla			38.12	-1.97	1300
	SIVIM	T-P28632:Aegi	Observation	Aegilops geniculata Roth	Roth	ES	Spain	Cerro del Cesto, Romangordo			39.71	-5.79	290
00:00.0	COFC	52512-1	Specimen	Aegilops geniculata Roth	Roth	ES	Spain	Olivenza; pantano de Piedra Aguda		Ba			1
00:00.0	SALA	40753-1	Specimen	Aegilops geniculata Roth	Roth	ES	Spain	_; PeÃ±afiel, Aldeyuso		Va			
00:00.0	SALA	42163-1	Specimen	Aegilops geniculata Roth	Roth	ES	Spain	_; Entre MorÃ³n y VillamartÃ-n		Se			
	SIVIM	T-P13692:Aegi	Observation	Aegilops geniculata Roth	Roth	ES	Spain	MasÃ-a de la Rambla, Vilafranca			40.43	-0.28	1140
	SIVIM	T-P16886:Aegi	Observation	Aegilops geniculata Roth	Roth	ES	Spain	Paracuellos de Jarama			40.46	-3.58	0
	SIVIM	T-P19062:Aegi	Observation	Aegilops geniculata Roth	Roth	ES	Spain	Blanquizares de Gor			37.31	-3	1300
	SIVIM	T-P20232:Aegi	Observation	Aegilops geniculata Roth	Roth	ES	Spain	Inmediaciones de Puente Genil			37.3	-4.8	150
	SIVIM	T-P26236:Aegi	Observation	Aegilops geniculata Roth	Roth	ES	Spain	Sierra de la Zarza, Caravaca			38.03	-1.86	1300
	SIVIM	T-P28630:Aegi	Observation	Aegilops geniculata Roth	Roth	ES	Spain	Castillo de Mirabel			39.8	-6.31	0
00:00.0	GDA	GDA12219-1-1	Specimen	Aegilops geniculata Roth.	Roth.	ES	Spain	Granada, SÂª Nevada, CÃ¡Ã±ar, ba		GR			1100
00:00.0	GDA	GDA15236-1-1	Specimen	Aegilops geniculata Roth.	Roth.	ES	Spain	Granada, carretera Ã•llora-MontefrÃ-		GR			700
00:00.0	GDA	GDA16059-1-2	Specimen	Aegilops geniculata Roth.	Roth.	ES	Spain	Granada, SÂª de Obeilar, cortijo de		GR			700
00:00.0	GDA	GDA25153-1-1	Specimen	Aegilops geniculata Roth.	Roth.	ES	Spain	Granada, Pedro MartÃ-nez, Mencal		GR			1150
00:00.0	MA	720091-1	Specimen	Aegilops geniculata Roth.	Roth.	ES	Spain	Moncloa		M			
	IDBD-GN	42461	Observation	Aegilops geniculata Roth	Roth	ES	Spain		Milagro	Na	42.2183	-1.72911	285
00:00.0	MA	562837-1	Specimen	Aegilops geniculata Roth.	Roth.	ES	Spain	Cubillo		Sg	41	-3	
00:00.0	HSS	2702	Specimen	Aegilops geniculata Roth	Roth	ES	Spain	Guadajira, Finca La Orden		Ba	38.8722	-6.75254	
00:00.0	COFC	46888-1	Specimen	Aegilops geniculata Roth	Roth	ES	Spain	Fuente Obejuna; aldea El Alcornoca		Co	38	-5	700
	FUND. BIODIVER	1001949	Unknown	Aegilops geniculata		ESP	Spain	Sierra de San Felipe		Cu	40.1	-1.1	
00:00.0	FUND. BIODIVER	1715690	Unknown	Aegilops geniculata Roth	Roth	ESP	Spain	Peraleda, Mas Barrera		Ge	42.1	2.1	
	FUND. BIODIVER	1811868	Unknown	Aegilops geniculata		ESP	Spain	Valtierra		Na	41.1	-1.1	
00:00.0	REDIAM-CMA	294834	Observation	Aegilops geniculata		ESP	Spain		Villanueva del Arz	J	38.1729	-2.87496	780
00:00.0	REDIAM-CMA	326728	Observation	Aegilops geniculata		ESP	Spain		Casares	Ma	36.3902	-5.27833	295
00:00.0	REDIAM-CMA	381901	Observation	Aegilops geniculata		ESP	Spain				37.8578	-3.3783	
00:00.0	REDIAM-CMA	382850	Observation	Aegilops geniculata		ESP	Spain		Jimena de la Fron	Ca	36.4453	-5.44551	97

	GDA	GDA30014-1-2	Specimen	Aegilops geniculata Roth.	Roth.	ES	Spain	Granada.		GR			0
	SIVIM	U-P09882:Aegi	Observation	Aegilops geniculata Roth	Roth	ES	Spain	Cuneta cercana a 'El Sabinar'			38.12	-2.2	0
00:00.0	MGC	60412-1	Unknown	Aegilops geniculata Roth	Roth	ES	Spain	Nerja; P. N. de las Sierras de Tejed		Ma	36.787	-3.79	500
00:00.0	MUB	100429-1	Specimen	Aegilops geniculata Roth	Roth	ES	Spain	Yecla; Sierra de Salinas		Mu	38.468	-1.057	1
	FUND. BIODIVER	1648484	Unknown	Aegilops geniculata Roth	Roth	ESP	Spain	Iglesuela del Cid, Mas de la Loma		Te	40.1	-0.1	
00:00.0	FUND. BIODIVER	1676972	Unknown	Aegilops ovata L.	L.	ESP	Spain	Archipielago de Cabrera, isla de Ca		PM	38.1	2.1	
	FUND. BIODIVER	1701104	Unknown	Aegilops geniculata		ESP	Spain	Mosqueruela, Masico Bielsa		Te	40.1	-0.1	1560
	FUND. BIODIVER	1648486	Unknown	Aegilops geniculata Roth	Roth	ESP	Spain	Cantavieja, Mas de Porcar		Te	40.1	-0.1	
00:00.0	REDIAM-CMA	282131	Observation	Aegilops geniculata		ESP	Spain		Hinojosa del Duq	Co	38.4092	-5.09873	575
00:00.0	REDIAM-CMA	289341	Observation	Aegilops geniculata		ESP	Spain		Santiago-Pontone	J	38.0866	-2.82165	807
00:00.0	REDIAM-CMA	278372	Observation	Aegilops geniculata		ESP	Spain		Guadalcanal	Se	38.0903	-5.74322	666
00:00.0	REDIAM-CMA	282190	Observation	Aegilops geniculata		ESP	Spain		Hinojosa del Duq	Co	38.4156	-5.06208	555
00:00.0	COA	41161-1	Specimen	Aegilops geniculata Roth	Roth	ES	Spain	scuela TÃ©cnica Superior de Ingen		Co	37.84	-4.82	
	SIVIM	T-P30033:Aegi	Observation	Aegilops geniculata Roth	Roth	ES	Spain	Pantano del Molinar, Villa de Ves, Albacete			39.19	-1.26	0
	SIVIM	T-P30058:Aegi	Observation	Aegilops geniculata Roth	Roth	ES	Spain	El Campichuelo, Cofrentes, Valencia			39.19	-1.14	0
	SIVIM	T-P32157:Aegi	Observation	Aegilops geniculata Roth	Roth	ES	Spain	La Foia			40.62	0.51	0
	SIVIM	U-P02907:Aegi	Observation	Aegilops geniculata Roth	Roth	ES	Spain	Vall d'AlcalÃ , Beniaia			38.72	-0.35	0
	SIVIM	U-P06647:Aegi	Observation	Aegilops geniculata Roth	Roth	ES	Spain	Rocha da Pena			37.22	-8.21	250
	SIVIM	U-P07712:Aegi	Observation	Aegilops geniculata Roth	Roth	ES	Spain	Valencia: BÃ©tera			39.54	-0.55	100
	SIVIM	T-P30037:Aegi	Observation	Aegilops geniculata Roth	Roth	ES	Spain	Pr. Barrio del Santuario, Villa de Ves, Albac			39.19	-1.26	0
	SIVIM	T-P30060:Aegi	Observation	Aegilops geniculata Roth	Roth	ES	Spain	Los Callejones, Cofrentes, Valencia			39.19	-1.26	0
	SIVIM	U-P00632:Aegi	Observation	Aegilops geniculata Roth	Roth	ES	Spain	Prox. El Valle de Las Casas			42.7	-5.19	1020
	SIVIM	U-P02909:Aegi	Observation	Aegilops geniculata Roth	Roth	ES	Spain	Vall de Gallinera			38.81	-0.23	0
	SIVIM	U-P06649:Aegi	Observation	Aegilops geniculata Roth	Roth	ES	Spain	Rib. da Quinta do Freixo			37.22	-8.21	2400
	SIVIM	U-P07716:Aegi	Observation	Aegilops geniculata Roth	Roth	ES	Spain	Valencia: Valencia, El Saler			39.26	-0.33	10
00:00.0	GDA	GDA30016-1-2	Specimen	Aegilops geniculata Roth	Roth	ES	Spain	Granada, Golilla de Cartuja.		GR			0
00:00.0	SEV	72965-1	Specimen	Aegilops geniculata Roth	Roth	ES	Spain	Tarifa. Arroyo de los Toriles. Cortijo		Ca			80
00:00.0	SEV	9804-1	Specimen	Aegilops ovata L.	L.	ES	Spain	Ronda. Sierra de las Nieves		Ma			1300
00:00.0	UNEX	10369-1	Observation	Aegilops geniculata Roth	_	ESP	Spain	Olivenza: Pantano de Piedra Aguda		Ba	38.7	-7.2	
00:00.0	MGC	11815-1	Unknown	Aegilops geniculata Roth	Roth	ES	Spain	Sierra Blanca. Carretera de IstÃ¡n		Ma	0	0	1
00:00.0	GDAC	GDAC11966-1	Specimen	Aegilops geniculata Roth.	Roth.	ES	Spain	Granada, Valle del Darro.		GR			0
00:00.0	BC	70799	Specimen	Aegilops geniculata Roth	Roth	ES	Spain	Jodar; JÃ³dar. Las Hermanas		J	37.81	-3.4	600
00:00.0	UNEX	10371-1	Observation	Aegilops geniculata Roth	_	ESP	Spain	Don Benito: Carretera de La Haba.		Ba	38.9	-5.9	
00:00.0	GDA	GDA48832-1-1	Specimen	Aegilops geniculata Roth.	Roth.	ES	Spain	AlmerÃ-a, SÂª de los Filabres, Beni		AL			850
00:00.0	HSS	8961	Specimen	Aegilops geniculata Roth	Roth	ES	Spain	Ctra. AlÃ-a - Puerto de San Vicente		Cc	39.4147	-5.14896	
00:00.0	BDBCV	599503	Observation	Aegilops geniculata		ESP	Spain		Alcoy	Alicant	38.655	-0.478	
	FUND. BIODIVER	1463975	Unknown	Aegilops geniculata Roth	Roth	ESP	Spain	Fontllonga		L	41.1	0.1	650
	FUND. BIODIVER	913680	Unknown	Aegilops ovata L.	L.	ESP	Spain	Massif de:Tibidabo,Levant		B	41.1	1.1	
	FUND. BIODIVER	918144	Unknown	Aegilops ovata L.	L.	ESP	Spain	Montserrat		B	41.1	1.1	
	FUND. BIODIVER	930423	Unknown	Aegilops ovata L.	L.	ESP	Spain	Plans de Prades, Plans de Pages		T	41.1	1.1	
	FUND. BIODIVER	939739	Unknown	Aegilops ovata L.	L.	ESP	Spain	Balaguer		L	41.1	0.1	

00:00.0	GDA	GDA30018-1	Specimen	Aegilops ovata L. var. leptostachy	L.	ES	Spain	Cabrera, puerto.		PM			0
	GDAC	GDAC2931-1	Specimen	Aegilops ovata L.	L.	ES	Spain	Granada, SÂª Nevada, Fuente del H		GR			0
	MA	584691-1	Specimen	Aegilops geniculata Roth.	Roth.	ES	Spain	Murcia		Mu			
00:00.0	REDIAM-CMA	134496	Observation	Aegilops geniculata		ESP	Spain		Santiago-Pontone	J	38.1581	-2.76435	887
00:00.0	REDIAM-CMA	144239	Observation	Aegilops geniculata		ESP	Spain		Aroche	H	37.9202	-6.93969	500
00:00.0	REDIAM-CMA	186205	Observation	Aegilops geniculata		ESP	Spain		Segura de la Sier	J	38.2141	-2.64396	1327
00:00.0	FCO	24499-1	Specimen	Aegilops ovata L.	L.	ES	Spain	Olmedo; Olmedo		Va			
	BC	92763	Specimen	Aegilops ovata L.	L.	ES	Spain	TÃ rrega; St. Eloi TÃ¡rrega		L	41.67	1.14	
00:00.0	HUAL	1132-1	Specimen	Aegilops geniculata Roth	Roth	ES	Spain	SÂª del Pozo, arroyo del Vidrio		J	37.772	-2.926	
	SEV	12009-1	Specimen	Aegilops ovata L.	L.	ES	Spain	Dehesa de la Villa		M			1
	SIVIM	T-P06428:Aegi	Observation	Aegilops geniculata Roth	Roth	ES	Spain	Villargordo del Cabriel			39.46	-1.48	0
	SIVIM	T-P07212:Aegi	Observation	Aegilops geniculata Roth	Roth	ES	Spain	Abiego			42.05	-0.09	0
	SIVIM	T-P09161:Aegi	Observation	Aegilops geniculata Roth	Roth	ES	Spain	Renedo			41.62	-4.68	0
	SIVIM	T-P09366:Aegi	Observation	Aegilops geniculata Roth	Roth	ES	Spain	Patones			40.82	-3.59	0
	SIVIM	T-P11404:Aegi	Observation	Aegilops geniculata Roth	Roth	ES	Spain	Pto. de Cabrejas a Cuenca			40.01	-2.29	1000
	SIVIM	T-P13153:Aegi	Observation	Aegilops geniculata Roth	Roth	ES	Spain	Sierra de Izco			42.62	-1.53	0
	W	42644	Unknown	Aegilops ovata L.		ESP	Spain	Baetica.					
00:00.0	SALA	46259-1	Specimen	Aegilops geniculata Roth	Roth	ES	Spain	_; Cantalapiedra		Sa			
	ADIMAN	22	Observation	Aegilops geniculata		ESP	Spain	EnguÃ-danos		CU	39.6391	-1.66812	
00:00.0	ABH	1595-1	Specimen	Aegilops geniculata Roth	Roth	ES	Spain	Alicante; Vistahermosa		A	38.37	-0.48	
	IPK	32066	Living	Aegilops geniculata Roth		ESP	Spain	Pablo Romaso, Stra?e Sevilla - Huelva, 4 km von Sanlucar la Ma					40
00:00.0	COA	24064-1	Specimen	Aegilops geniculata Roth	Roth	ES	Spain	Antequera. Sierra de Huma. Puerto		Ma	36.85	-4.79	
00:00.0	SALA	5480-1	Specimen	Aegilops ovata L. subsp. triaristat	(Willd.) Rouy	ES	Spain	_; El Cabaco		Sa			
00:00.0	BDBCV	331889	Observation	Aegilops geniculata	_	ES	Spain		La Nucia	A	38.59	-0.07	
00:00.0	SEV	49876-1	Specimen	Aegilops ovata L.	L.	ES	Spain	Higuera de la Sierra		H			1
	BDBCV-General	280033	Observation	Aegilops geniculata		ESPA	Spain	NavarrÃ©s	La Canal de Nava	Valenc	39.0517	-0.74664	
00:00.0	BDBCV	104	Observation	Aegilops geniculata		ESP	Spain	Parque Natural de Penyagolosa		Cs	40.17	-0.36	
00:00.0	MA	587585-1	Specimen	Aegilops geniculata Roth.	Roth.	ES	Spain	AlcalÃ¡ de Henares, Universidad		M			
00:00.0	SEV	5987-1	Specimen	Aegilops ovata L.	L.	ES	Spain	Padilla de Hita		Gu			1
	BDBCV-General	66990	Observation	Aegilops geniculata		ESPA	Spain	Vistabella del Ma	L'AlcalatÃ©n	Castel	40.3045	-0.35257	
00:00.0	COFC	11975-1	Specimen	Aegilops geniculata Roth	Roth	ES	Spain	BenamejÃƒÂ-; rÃƒÂ-o Genil		Co	0	0	1
00:00.0	MA	640320-1	Specimen	Aegilops geniculata Roth	Roth	ES	Spain	Montorio		Bu	42	-3	
00:00.0	LEB	11623-1	Specimen	Aegilops geniculata Roth	Roth	ES	Spain	La Vid		Le	42.92	-5.63	1
	BDBCV-General	77380	Observation	Aegilops geniculata		ESPA	Spain	SacaÃ±et	El Alto Palancia	Castel	39.862	-0.72035	
00:00.0	COFC	11971-1	Specimen	Aegilops geniculata Roth	Roth	ES	Spain	IznÃ¡jar; arroyo de Priego		Co	37	-4	1
00:00.0	MA	750080-1	Specimen	Aegilops geniculata Roth	Roth	ES	Spain	Covarrubias		Bu	42	-3	
	BDBCV-General	276388	Observation	Aegilops geniculata		ESPA	Spain	Bolbaite	La Canal de Nava	Valenc	39.0517	-0.74664	
	BDBCV-General	280034	Observation	Aegilops geniculata		ESPA	Spain	Bolbaite	La Canal de Nava	Valenc	39.0517	-0.74664	
00:00.0	MUB	102371-1	Specimen	Aegilops geniculata Roth	Roth	ES	Spain	Cartagena; Vaguada de Las Carras		Mu	37.622	-1.159	270
	RUS001	VIR100602111	Specimen	Aegilops ovata L.		ESP	Spain						
	RUS001	VIR100602090	Specimen	Aegilops ovata L.		ESP	Spain						

	RUS001	VIR100602340	Specimen	Aegilops ovata L.		ESP	Spain						
	FUND. BIODIVER	120460	Unknown	Aegilops geniculata Roth	Roth	ESP	Spain	Valdevacas de Montejo, Valdevacas		Sg	41.1	-3.1	
	FUND. BIODIVER	1335997	Unknown	Aegilops geniculata Roth	Roth	ESP	Spain	Castillonuevo, Sierra de Leyre		Na	42.1	-0.1	910
	FUND. BIODIVER	70821	Unknown	Aegilops geniculata Roth	Roth	ESP	Spain	Torre Val de San Pedro		Sg	40.1	-3.1	
	FUND. BIODIVER	72900	Unknown	Aegilops geniculata Rotch.	Rotch.	ESP	Spain	Salamanca, Tejares		Sa	40.1	-5.1	
	FUND. BIODIVER	1093231	Unknown	Aegilops geniculata		ESP	Spain	El Arco, CaÃ±averal		Cc	39.1	-6.1	
	FUND. BIODIVER	1093253	Unknown	Aegilops geniculata		ESP	Spain	Almaraz		Cc	39.1	-5.1	
	FUND. BIODIVER	1925488	Unknown	Aegilops geniculata Roth	Roth	ESP	Spain	Elcoaz, Collado Borrokosko		Na	42.1	-1.1	1000
	FUND. BIODIVER	1930606	Unknown	Aegilops geniculata		ESP	Spain	Llucalari		PM	39.1	3.1	
	FUND. BIODIVER	1946395	Unknown	Aegilops geniculata Roth	Roth	ESP	Spain	Provincia de Ciudad Real		CR			
00:00.0	SEV	99210-1	Specimen	Aegilops geniculata Roth	Roth	ES	Spain	Arcos de la Frontera. Alrededores d		Ca			1
00:00.0	REDIAM-CMA	124697	Observation	Aegilops geniculata		ESP	Spain		Constantina	Se	37.911	-5.51221	600
	ESP004	NC027436	Specimen	Aegilops geniculata Roth		ESP	Spain	Vall de Ebo, province of Alicante			38.8	-0.15	394
	ESP004	NC022308	Specimen	Aegilops geniculata Roth		ESP	Spain	El Guijo, Arcos de la Frontera, province of (			36.7333	-5.86667	102
00:00.0	REDIAM-CMA	9417	Observation	Aegilops geniculata		ESP	Spain		Ronda	Ma	36.8286	-5.24134	909
00:00.0	REDIAM-CMA	17024	Observation	Aegilops geniculata		ESP	Spain		Alpandeire	Ma	36.6682	-5.22654	959
	REDIAM-CMA	396504	Observation	Aegilops geniculata		ESP	Spain		Jerez de la Fronte	Ca	36.6108	-5.54314	303
00:00.0	REDIAM-CMA	400852	Observation	Aegilops geniculata		ESP	Spain		Santiago-Pontone	J	38.1179	-2.66737	1400
00:00.0	REDIAM-CMA	411283	Observation	Aegilops geniculata		ESP	Spain		Antas	Al	37.2736	-2.00729	442
00:00.0	REDIAM-CMA	419124	Observation	Aegilops geniculata		ESP	Spain		El Pinar	Gr	36.8785	-3.54754	969
00:00.0	MA	612360-1	Specimen	Aegilops geniculata Roth.	Roth.	ES	Spain	La AzohÃ-a, castillo		Mu	37	-1	
00:00.0	MA	700388-1	Specimen	Aegilops geniculata Roth	Roth	ES	Spain	Villamayor de Calatrava, volcÃ¡n de		CR	38	-4	
	SIVIM	S-P13989:Aegi	Observation	Aegilops geniculata Roth	Roth	ES	Spain	Pinhal da Senhora (Freixo do Meio)			39.38	-8.88	148
	SIVIM	T-P03846:Aegi	Observation	Aegilops geniculata Roth	Roth	ES	Spain	3 km al S de Patones			40.82	-3.59	0
	SIVIM	T-P04225:Aegi	Observation	Aegilops geniculata Roth	Roth	ES	Spain	13 km al W de Lorca			37.67	-1.86	310
	SIVIM	T-P06422:Aegi	Observation	Aegilops geniculata Roth	Roth	ES	Spain	Casas de Benali			38.92	-0.92	0
	FUND. BIODIVER	1648487	Unknown	Aegilops geniculata Roth	Roth	ESP	Spain	Cantavieja		Te	40.1	-0.1	
	FUND. BIODIVER	1340282	Unknown	Aegilops geniculata Roth	Roth	ESP	Spain	FrÃ-as		Bu	42.1	-3.1	550
	FUND. BIODIVER	135668	Unknown	Aegilops ovata		ESP	Spain	Fredes, La Senia, Tossal Rei		Cs	40.1	-0.9	
	FUND. BIODIVER	70864	Unknown	Aegilops ovata L.	L.	ESP	Spain	Parada de Rubiales, camino hacia L		Sa	40.1	-5.1	
	SIVIM	Q-P03601:Aegi	Observation	Aegilops geniculata Roth	Roth	ES	Spain	Castillo de Villamalefa			40.08	-0.41	0
	SIVIM	Q-P06537:Aegi	Observation	Aegilops geniculata Roth	Roth	ES	Spain	Els Estepars, entre Prades i Albarca			41.26	0.85	0
	SIVIM	Q-P08745:Aegi	Observation	Aegilops geniculata Roth	Roth	ES	Spain	Guardo ,			42.7	-4.95	1120
	SIVIM	R-P03722:Aegi	Observation	Aegilops geniculata Roth	Roth	ES	Spain	Sierra del Yugo			42.17	-1.66	320
	SIVIM	R-P08198:Aegi	Observation	Aegilops geniculata Roth	Roth	ES	Spain	Sobre CastellolÃ-, camÃ- de la font del Fer			41.54	1.68	0
	SIVIM	R-P09412:Aegi	Observation	Aegilops geniculata Roth	Roth	ES	Spain	Cirueches			41.1	-2.76	0
00:00.0	COA	23220-1	Specimen	Aegylops geniculata Roth	Roth	ES	Spain	Valle de AbdalajÃ-s. Sierra del Valle		Ma	36.85	-4.79	
00:00.0	REDIAM-CMA	241421	Observation	Aegilops geniculata		ESP	Spain		Alhama de Grana	Gr	37.0173	-3.91252	799
00:00.0	REDIAM-CMA	248510	Observation	Aegilops geniculata		ESP	Spain		Hinojales	H	38.0018	-6.58287	521
00:00.0	REDIAM-CMA	282228	Observation	Aegilops geniculata		ESP	Spain		Villanueva del Du	Co	38.3783	-5.02714	606
00:00.0	REDIAM-CMA	82100	Observation	Aegilops geniculata		ESP	Spain		Puente de GÃ©na	J	38.3136	-2.81692	984

00:00.0	REDIAM-CMA	114127	Observation	Aegilops geniculata		ESP	Spain		Zufre	H	37.8772	-6.3606	383
00:00.0	REDIAM-CMA	126986	Observation	Aegilops geniculata		ESP	Spain		Constantina	Se	37.8999	-5.63561	600
00:00.0	MGC	35100-1	Unknown	Aegilops geniculata Roth	Roth	ES	Spain	MÃ¡laga; Campus de Teatinos		Ma	0	0	1
	SIVIM	T-P30038:Aegi	Observation	Aegilops geniculata Roth	Roth	ES	Spain	Bajada a Pantano del Molinar, Villa de Ves			39.19	-1.26	0
	SIVIM	T-P30061:Aegi	Observation	Aegilops geniculata Roth	Roth	ES	Spain	Pr. Corral Confite, Villamalea, Albacete			39.29	-1.6	0
	SIVIM	U-P00808:Aegi	Observation	Aegilops geniculata Roth	Roth	ES	Spain	Cistierna, Monte Sorriba			42.79	-5.2	0
	SIVIM	U-P02910:Aegi	Observation	Aegilops geniculata Roth	Roth	ES	Spain	Vall de Gallinera			38.81	-0.35	0
	SIVIM	U-P06650:Aegi	Observation	Aegilops geniculata Roth	Roth	ES	Spain	Quinta da Figueirinha			37.13	-8.43	0
	SIVIM	U-P07717:Aegi	Observation	Aegilops geniculata Roth	Roth	ES	Spain	Valencia: Burjassot			39.45	-0.44	0
	SIVIM	S-P04193:Aegi	Observation	Aegilops geniculata Roth	Roth	ES	Spain	km 3 de la carretera de Balaguer a Agramu			41.71	0.83	270
	SIVIM	S-P09961:Aegi	Observation	Aegilops geniculata Roth	Roth	ES	Spain	prop de l'AlfÃ s			38.54	-0.13	100
	SIVIM	S-P14066:Aegi	Observation	Aegilops geniculata Roth	Roth	ES	Spain	Freixo do Meio			39.38	-8.88	145
	SIVIM	T-P01870:Aegi	Observation	Aegilops geniculata Roth	Roth	ES	Spain	Eivissa: el Pla de Vila, al peu dels turons q			38.83	1.38	0
	SIVIM	T-P03858:Aegi	Observation	Aegilops geniculata Roth	Roth	ES	Spain	2 km al SW de Valdeavero			40.56	-3.35	0
	SIVIM	T-P06398:Aegi	Observation	Aegilops geniculata Roth	Roth	ES	Spain	Ayora			39.01	-1.26	0
	ESP004	NC027421	Specimen	Aegilops geniculata Roth		ESP	Spain	Huelves, province of Cuenca			40.0333	-2.86667	817
00:00.0	SALA	52035-1	Specimen	Aegilops geniculata Roth	Roth	ES	Spain	_; Algodre, Las Gavias		Za			
	FUND. BIODIVER	70869	Unknown	Aegilops ovata L.	L.	ESP	Spain	Espino de la Orbada		Sa	40.1	-5.1	
00:00.0	GDA	GDA30009-1	Specimen	Aegilops ovata L.	L.	ES	Spain	MÃ¡laga, AxarquÃ-a.		MA			0
	IPK	32337	Living	Aegilops geniculata Roth		ESP	Spain	Spain: Prov. Granada, Moreda,ca. 40 km NNE of Granada					
	BG-UPM	3053	Unknown	Aegilops geniculata Roth	Roth	ESP	Spain	Ciudad Universitaria		M			
	BC	92741	Specimen	Aegilops ovata L.	L.	ES	Spain	Vallfogona de Riucorb; Vallfogona d		T	41.58	1.26	
00:00.0	REDIAM-CMA	82845	Observation	Aegilops geniculata		ESP	Spain		Ronda	Ma	36.6847	-5.06212	1299
00:00.0	REDIAM-CMA	96603	Observation	Aegilops geniculata		ESP	Spain		El Pedroso	Se	37.7994	-5.78427	350
00:00.0	REDIAM-CMA	107358	Observation	Aegilops geniculata		ESP	Spain		Hornos	J	38.1801	-2.64658	1430
00:00.0	COA	41163-1	Specimen	Aegilops geniculata Roth	Roth	ES	Spain	Km 9 de Posadas a Palma del RÃ-o		Co	37.75	-5.27	
00:00.0	SALA	119746-1	Specimen	Aegilops geniculata Roth	Roth	ES	Spain	Jaca, au confluent du rÃ-o Gas et d		Hu	42.55	-0.61	
	SIVIM	S-P14142:Aegi	Observation	Aegilops geniculata Roth	Roth	ES	Spain	Herdade dos Almendres			42.99	-3.49	265
	SIVIM	T-P03197:Aegi	Observation	Aegilops geniculata Roth	Roth	ES	Spain	Bajo Pueyo (Macizo del TurbÃ³n)			42.33	0.45	1150
	SIVIM	T-P04122:Aegi	Observation	Aegilops geniculata Roth	Roth	ES	Spain	1 km al W de Cofrentes			39.19	-1.14	0
	SIVIM	T-P06408:Aegi	Observation	Aegilops geniculata Roth	Roth	ES	Spain	Andilla			39.82	-0.89	0
00:00.0	BDBCV	590351	Observation	Aegilops geniculata		ESP	Spain		Alcoy	Alicant	38.665	-0.535	
	FUND. BIODIVER	1035830	Unknown	Aegilops ovata		ESP	Spain	Sierra de Aguas, Arroyo de los Hue		Ma	36.1	-4.1	
	FUND. BIODIVER	1043755	Unknown	Aegilops geniculata Roth	Roth	ESP	Spain	IstÃ¡n, Bohornoque		Ma	36.1	-4.1	
00:00.0	FUND. BIODIVER	1835084	Unknown	Aegilops geniculata Roth	Roth	ESP	Spain	Villamayor de Calatrava, volcan del		CR	38.1	-3.1	842
	FUND. BIODIVER	1889261	Unknown	Aegilops geniculata Roth	Roth	ESP	Spain	Sierra de Orihuela		A	37.1	-0.1	
	COA	693-1	Specimen	Aegylops ovata L.	L.	ES	Spain	CÃ³rdoba, casco urbano		Co	37.84	-4.82	
00:00.0	MA	636846-1	Specimen	Aegilops geniculata		ES	Spain	Torre Val de San Pedro, saliendo de		Sg	41	-3	
00:00.0	SALA	78074-1	Specimen	Aegilops geniculata Roth	Roth	ES	Spain	_; JaÃ©n		J	37.76	-4.1	
00:00.0	REDIAM-CMA	384771	Observation	Aegilops geniculata		ESP	Spain				37.7402	-3.15839	
00:00.0	REDIAM-CMA	389202	Observation	Aegilops geniculata		ESP	Spain		Zafarraya	Gr	36.9912	-4.16314	925

00:00.0	REDIAM-CMA	392854	Observation	Aegilops geniculata		ESP	Spain		Torres	J	37.7441	-3.5535	1298
00:00.0	SALA	97806-1	Specimen	Aegilops geniculata Roth	Roth	ES	Spain	_; Mallorca, Puigpunyent, subida ha		Mll	39.63	2.52	
	SIVIM	P-P08869:Aegi	Observation	Aegilops geniculata Roth	Roth	ES	Spain	Els Garrigons; Beseit			40.79	0.15	0
	SIVIM	P-P08920:Aegi	Observation	Aegilops geniculata Roth	Roth	ES	Spain	CoratxÃ			40.61	0.04	1210
	SIVIM	P-P09128:Aegi	Observation	Aegilops geniculata Roth	Roth	ES	Spain	CapÃ§alera Barranc del Retaule; la SÃ¨nia			40.7	0.27	1130
	SIVIM	P-P11352:Aegi	Observation	Aegilops geniculata Roth	Roth	ES	Spain	PaÃ¼ls, rodalies			40.89	0.38	310
	SIVIM	Q-P01679:Aegi	Observation	Aegilops geniculata Roth	Roth	ES	Spain	Las Ramblillas. Abejuela			39.91	-0.89	1500
	SANT	17438	Specimen	Aegilops geniculata Roth		ES	Spain	RubiÃ¡,Cobas hacia Vilardesilva		Or			
00:00.0	SEV	98680-1	Specimen	Aegilops geniculata Roth	Roth	ES	Spain	Montoro		Co			1
	SANT	12400	Specimen	Aegilops ovata L. subsp. ovata		ES	Spain	La Moncloa		M			
00:00.0	UA	100105	Specimen	Aegilops ovata L.		Andalu	Spain	Casares			36.45	-5.28333	
00:00.0	BDBCV	588963	Observation	Aegilops geniculata		ESP	Spain	Parc Natural de la	Alcoy	Alicant	38.719	-0.533	
00:00.0	BDBCV	589880	Observation	Aegilops geniculata		ESP	Spain		Alcoy	Alicant	38.684	-0.58	
00:00.0	COFC	46880-1	Specimen	Aegilops geniculata Roth	Roth	ES	Spain	ctra de Santa MarÃƒÂ-a de Trasierr		Co			1
	FUND. BIODIVER	1000496	Unknown	Aegilops geniculata		ESP	Spain	JÃ¡bala, Boniches		Cu	39.1	-1.1	
	FUND. BIODIVER	101226	Unknown	Aegilops geniculata Roth	Roth	ESP	Spain	Recuerda, Hacia Morales		So	41.1	-2.1	900
	FUND. BIODIVER	1701106	Unknown	Aegilops geniculata		ESP	Spain	Mosqueruela, Cuartel-Balsa de la H		Te	40.1	-0.1	1420
	FUND. BIODIVER	1715682	Unknown	Aegilops geniculata		ESP	Spain	Siurana, entre Brava i Baseia		Ge	42.1	2.1	
00:00.0	BDBCV	334934	Observation	Aegilops geniculata	_	ES	Spain		L'AlfÃ s del Pi	A	38.59	-0.07	
00:00.0	BC	601430	Specimen	Aegilops ovata L.	L.	ES	Spain	l'Espluga de FrancolÃ-; Conca de B		T	41.4	1.15	625
	FUND. BIODIVER	1463973	Unknown	Aegilops geniculata Roth	Roth	ESP	Spain	Serra de MonterÃ³		L	41.1	0.1	360
	FUND. BIODIVER	930421	Unknown	Aegilops ovata L.	L.	ESP	Spain	Puig de Marc, Valle El Titllar		T	41.1	0.1	
	REDIAM-CMA	289960	Observation	Aegilops geniculata		ESP	Spain		AlcalÃ¡ de los Ga	Ca	36.3842	-5.65719	49
00:00.0	REDIAM-CMA	334982	Observation	Aegilops geniculata		ESP	Spain		Los Barrios	Ca	36.1992	-5.44406	50
00:00.0	REDIAM-CMA	381530	Observation	Aegilops geniculata		ESP	Spain		Medina-Sidonia	Ca	36.4552	-5.81214	97
00:00.0	REDIAM-CMA	382670	Observation	Aegilops geniculata		ESP	Spain		La Puebla de Caz	Se	37.1048	-5.28625	399
	FUND. BIODIVER	1050673	Unknown	Aegilops ovata		ESP	Spain	Villaluenga del Rosario		Ca	36.1	-5.1	
	FUND. BIODIVER	1067625	Unknown	Aegilops ovata L.	L.	ESP	Spain	Sevilla		Se	37.1	-5.1	
00:00.0	FUND. BIODIVER	1835074	Unknown	Aegilops geniculata Roth	Roth	ESP	Spain	Moral de Calatrava, sierra de Moral		CR	37.1	-3.1	
	FUND. BIODIVER	1869149	Unknown	Aegilops geniculata Roth	Roth	ESP	Spain	Ciudad Real, La Atalaya		CR	38.1	-3.1	670
	FUND. BIODIVER	1893638	Unknown	Aegilops ovata L.	L.	ESP	Spain	Tenerife, Igueste de Candelaria		Tf	28.1	-16.1	500
	SIVIM	U-P08619:Aegi	Observation	Aegilops geniculata Roth	Roth	ES	Spain	Fortuna			38.2	-1.17	200
	SIVIM	U-P13324:Aegi	Observation	Aegilops geniculata Roth	Roth	ES	Spain	Marmolejo. Prox. Balneario de Aguas Mine			38.03	-4.25	280
00:00.0	MGC	13226-1	Unknown	Aegilops geniculata Roth	Roth	ES	Spain	Sorbas; Los CastaÃ±os		Al	37.18	-2.04	1
00:00.0	REDIAM-CMA	149095	Observation	Aegilops geniculata		ESP	Spain		Santiago-Pontone	J	38.049	-2.64888	1595
00:00.0	REDIAM-CMA	159839	Observation	Aegilops geniculata		ESP	Spain		Zufre	H	37.9373	-6.43405	396
00:00.0	REDIAM-CMA	384200	Observation	Aegilops geniculata		ESP	Spain		Torres	J	37.7376	-3.50333	1624
00:00.0	REDIAM-CMA	385483	Observation	Aegilops geniculata		ESP	Spain		Pegalajar	J	37.7335	-3.53145	1916
00:00.0	REDIAM-CMA	386875	Observation	Aegilops geniculata		ESP	Spain		Cambil	J	37.729	-3.48985	1594
00:00.0	REDIAM-CMA	388504	Observation	Aegilops geniculata		ESP	Spain		Teba	Ma	36.9803	-4.99731	600
	RUS001	VIR100602114	Specimen	Aegilops ovata L.		ESP	Spain						

	RUS001	VIR100602093	Specimen	Aegilops ovata L.		ESP	Spain						
	RUS001	VIR100602072	Specimen	Aegilops ovata L.		ESP	Spain						
	IPK	AE 356	Living	Aegilops geniculata Roth subsp. gibberosa (Zhuk.) K			Spain	Formentor, Majorque, Baleares			39.9664	3.2	
00:00.0	MGC	27652-1	Unknown	Aegilops geniculata Roth	Roth	ES	Spain	Antequera; Torcal		Ma	0	0	1
	SIVIM	T-P07205:Aegi	Observation	Aegilops geniculata Roth	Roth	ES	Spain	Ayerbe			42.24	-0.69	0
	SIVIM	T-P09157:Aegi	Observation	Aegilops geniculata Roth	Roth	ES	Spain	Portillo			41.44	-4.67	0
	SIVIM	T-P09355:Aegi	Observation	Aegilops geniculata Roth	Roth	ES	Spain	Puebla de BaleÃ±a			40.83	-3.23	0
	SIVIM	T-P10781:Aegi	Observation	Aegilops geniculata Roth	Roth	ES	Spain	Faramontanos de TÃ¡bara			41.78	-5.88	0
	SIVIM	T-P13148:Aegi	Observation	Aegilops geniculata Roth	Roth	ES	Spain	Gallipienzo, hacia del Murillo del Fruto			42.44	-1.54	0
	SIVIM	T-P13228:Aegi	Observation	Aegilops geniculata Roth	Roth	ES	Spain	UjuÃ©, de Gallipienzo a Murillo el Fruto			43.25	-1.52	0
	SIVIM	P-P08744:Aegi	Observation	Aegilops geniculata Roth	Roth	ES	Spain	Coll de RedÃ³; Tortosa			40.7	0.15	1040
	SIVIM	P-P08887:Aegi	Observation	Aegilops geniculata Roth	Roth	ES	Spain	Barranc del Grevolar; Arnes			40.79	0.27	0
	SIVIM	P-P09011:Aegi	Observation	Aegilops geniculata Roth	Roth	ES	Spain	CoratxÃ			40.61	0.04	1040
	SIVIM	P-P11096:Aegi	Observation	Aegilops geniculata Roth	Roth	ES	Spain	La Foia			40.62	0.51	0
	SIVIM	Q-P00141:Aegi	Observation	Aegilops geniculata Roth	Roth	ES	Spain	la Morera de Montsant, sortida del grau de			41.25	0.73	0
	FUND. BIODIVER	1077308	Unknown	Aegilops ovata L.	L.	ESP	Spain	PolÃ¡n		To	39.1	-3.1	
	FUND. BIODIVER	1093228	Unknown	Aegilops geniculata		ESP	Spain	Dehesa de los Caballos, Plasencia		Cc	39.1	-5.1	
	FUND. BIODIVER	1925485	Unknown	Aegilops geniculata Roth	Roth	ESP	Spain	Izal		Na	42.1	-0.1	1105
00:00.0	FUND. BIODIVER	1946392	Unknown	Aegilops geniculata Roth	Roth	ESP	Spain	Lagunas de Ruidera, borde de la la		CR	38.1	-2.1	
	IDBD-GN	42427	Observation	Aegilops geniculata Roth	Roth	ES	Spain		Zandio	Na	42.9057	-1.59869	
	IDBD-GN	42449	Observation	Aegilops geniculata Roth	Roth	ES	Spain	OyÃ³n		Vi	42.4987	-2.44756	450
00:00.0	REDIAM-CMA	8613	Observation	Aegilops geniculata		ESP	Spain		Casares	Ma	36.3842	-5.32549	80
00:00.0	REDIAM-CMA	410682	Observation	Aegilops geniculata		ESP	Spain		Siles	J	38.3857	-2.51501	1312
00:00.0	REDIAM-CMA	418343	Observation	Aegilops geniculata		ESP	Spain		Padul	Gr	37.015	-3.64155	799
00:00.0	HSS	12669	Specimen	Aegilops geniculata Roth	Roth	ES	Spain	La Albuera		Ba	38.6943	-6.87309	
	BDBCV-General	93158	Observation	Aegilops geniculata		ESPA	Spain	Caudiel	El Alto Palancia	Castel	40.0397	-0.59724	
	IPK	AE 1145	Living	Aegilops geniculata Roth			Spain	Portugal, Prov. Tras-os-Montes(Alto Douro			41.9008	-6.26056	
00:00.0	SALA	18872-1	Specimen	Aegilops ovata L.	L.	ES	Spain	_; Almenara de Tormes		Sa			
	SIVIM	Q-P03109:Aegi	Observation	Aegilops geniculata Roth	Roth	ES	Spain	La Llacuna, marge del camÃ- de la Font C			41.45	1.44	0
	SIVIM	Q-P07461:Aegi	Observation	Aegilops geniculata Roth	Roth	ES	Spain	La Almarcha, laguna AyrÃ³n			39.65	-2.41	0
	SIVIM	R-P03685:Aegi	Observation	Aegilops geniculata Roth	Roth	ES	Spain	Carretera de Milagro a Cadreita			42.17	-1.78	0
	SIVIM	R-P08185:Aegi	Observation	Aegilops geniculata Roth	Roth	ES	Spain	Can Soteres (CastellolÃ-)			41.54	1.68	0
	SIVIM	R-P08636:Aegi	Observation	Aegilops geniculata Roth	Roth	ES	Spain	Cerro de las Mesillas			36.77	-2.66	0
	IDBD-GN	42420	Observation	Aegilops geniculata Roth	Roth	ES	Spain	Foz de ArbayÃºn-	Yesa	Na	42.6619	-1.23204	
	IDBD-GN	42433	Observation	Aegilops geniculata Roth	Roth	ES	Spain	Arre	Esteribar	Na	42.8338	-1.61256	
	IDBD-GN	42442	Observation	Aegilops geniculata Roth	Roth	ES	Spain	Loma Negra	Bardenas Reales	Na	42.1236	-1.36807	
	IDBD-GN	42454	Observation	Aegilops geniculata Roth	Roth	ES	Spain		Belascoain	Na	42.7598	-1.8403	
	ESP004	NC050484	Specimen	Aegilops geniculata Roth		ESP	Spain	Las Majadas/Beteta 8km NE, Cuenca, prov			40.35	-2	1210
	ESP004	NC044545	Specimen	Aegilops geniculata Roth		ESP	Spain	Porto Colom, Cala S'Algar, Felanitx, provin			39.4167	3.21667	6
	FUND. BIODIVER	1120222	Unknown	Aegilops geniculata Roth	Roth	ESP	Spain	Algodre, Las Gavias		Za	41.1	-5.1	
	FUND. BIODIVER	1135162	Unknown	Aegilops geniculata Roth	Roth	ESP	Spain	MatadeÃ³n		Le	42.1	-5.1	910

	FUND. BIODIVER	115631	Unknown	Aegilops geniculata Roth	Roth	ESP	Spain	Guardo, Guardo		P	42.1	-4.1	
00:00.0	SEV	108247-1	Specimen	Aegilops geniculata Roth	Roth	ES	Spain	Obejo. IntesecciÃ³n de los rÃ-os Cu		Co			1
00:00.0	SEV	108283-1	Specimen	Aegilops geniculata Roth	Roth	ES	Spain	Cazalla de la Sierra		Se			1
00:00.0	BC	612853	Specimen	Aegilops ovata L.	L.	ES	Spain	Vielha e Mijaran; Vall de l'Artiga de		L	42.75	0.74	1000
00:00.0	GDA	GDA30020-1	Specimen	Aegilops ovata L.	L.	ES	Spain	Granada, SÂª Elvira.		GR			0
	REDIAM-CMA	36517	Observation	Aegilops geniculata		ESP	Spain		San JosÃ© del Va	Ca	36.6398	-5.68424	188
00:00.0	REDIAM-CMA	69021	Observation	Aegilops geniculata		ESP	Spain		Torres	J	37.7551	-3.54456	1277
00:00.0	BC	103932	Specimen	Aegilops ovata L.	L.	ES	Spain	Palma de Mallorca; sa Caseta Blanc		PM	39.16	2.94	
	SIVIM	R-P09473:Aegi	Observation	Aegilops geniculata Roth	Roth	ES	Spain	entre SigÃ¼enza y Pelegrina, al lado de un			41.01	-2.64	1110
	SIVIM	R-P10439:Aegi	Observation	Aegilops geniculata Roth	Roth	ES	Spain	K12 carretera del Picacho , AG			36.47	-5.67	0
	SIVIM	R-P11654:Aegi	Observation	Aegilops geniculata Roth	Roth	ES	Spain	Tarifa			44.93	-6.04	0
	SIVIM	S-P01246:Aegi	Observation	Aegilops geniculata Roth	Roth	ES	Spain	l'Espluga Calba, els Graus			41.44	0.96	0
	SIVIM	S-P02020:Aegi	Observation	Aegilops geniculata Roth	Roth	ES	Spain	El PLa d'Albelda			41.79	0.47	0
	SIVIM	S-P03962:Aegi	Observation	Aegilops geniculata Roth	Roth	ES	Spain	l'Espluga Calba, els Bassals			41.44	0.96	0
00:00.0	ABH	4861-1	Specimen	Aegilops geniculata Roth	Roth	ES	Spain	SÂªSerrella, P.Alta, Cova Negra		A	38.7	-0.31	
00:00.0	GDA	GDA30015-1-1	Specimen	Aegilops ovata L.	L.	ES	Spain	Salamanca, Monte de la Orbada.		SA			0
	ESP004	NC027454	Specimen	Aegilops geniculata Roth		ESP	Spain	Cella, province of Teruel			40.45	-1.28333	1023
	MUB	102375-1	Specimen	Aegilops geniculata Roth	Roth	ES	Spain	Cartagena; Carretera de Quitapellej		Mu			1
	FUND. BIODIVER	1487853	Unknown	Aegilops geniculata Roth	Roth	ESP	Spain	P.N. Sierra Nevada		Gr	36.1	-3.1	
	FUND. BIODIVER	1564722	Unknown	Aegilops geniculata Roth.	Roth.	ESP	Spain	Soto de Cerrato		P	41.1	-4.1	
00:00.0	FUND. BIODIVER	1597203	Unknown	Aegilops geniculata Roth	Roth	ESP	Spain	Monte del Duque		Le	41.1	-5.1	
	FUND. BIODIVER	1643496	Unknown	Aegilops geniculata Roth	Roth	ESP	Spain	El Castellar		Z	41.1	-0.1	540
	FUND. BIODIVER	998071	Unknown	Aegilops geniculata		ESP	Spain	CaÃ±ete		Cu	39.1	-1.1	
	FUND. BIODIVER	1715001	Unknown	Aegylops geniculata Roth.	Roth.	ESP	Spain	Sierra las Corchuelas, Parque Natu		Cc	39.1	-5.1	
00:00.0	MGC	49753-1	Unknown	Aegilops geniculata Roth	Roth	ES	Spain	Mijas; Mijas-Costa. Sitio de Calahor		Ma	0	0	1
00:00.0	FUND. BIODIVER	1332455	Unknown	Aegilops geniculata Roth.	Roth.	ESP	Spain	RÃ-o Yeguas		J	38.1	-4.1	
	FUND. BIODIVER	1336001	Unknown	Aegilops geniculata Roth	Roth	ESP	Spain	Rocaforte, el Romeral		Na	42.1	-1.1	560
	FUND. BIODIVER	1340278	Unknown	Aegilops geniculata Roth	Roth	ESP	Spain	FrÃ-as		Bu	42.1	-3.1	600
	FUND. BIODIVER	1354560	Unknown	Aegilops geniculata Roth	Roth	ESP	Spain	Aviados		Le	42.1	-5.1	
	FUND. BIODIVER	1366004	Unknown	Aegilops geniculata Roth	Roth	ESP	Spain	Lumbier, Biezcas		Na	42.1	-1.1	440
	FUND. BIODIVER	70860	Unknown	Aegilops ovata L.	L.	ESP	Spain	Valdunciel		Sa	40.1	-5.1	
	FUND. BIODIVER	78789	Unknown	Aegilops geniculata Roth.	Roth.	ESP	Spain	Portillo		Va	41.1	-4.1	
	RUS001	VIR100602210	Specimen	Aegilops ovata L.		ESP	Spain						
00:00.0	MUB	110869-1	Specimen	Aegilops geniculata Roth	Roth	ES	Spain	Cartagena; Proximidades a PerÃ-n		Mu	37.621	-1.091	1
00:00.0	REDIAM-CMA	201226	Observation	Aegilops geniculata		ESP	Spain		Sorbas	Al	37.0888	-2.08723	399
00:00.0	REDIAM-CMA	212072	Observation	Aegilops geniculata		ESP	Spain		Constantina	Se	37.958	-5.59167	680
00:00.0	REDIAM-CMA	237815	Observation	Aegilops geniculata		ESP	Spain		AlmadÃ©n de la P	Se	37.8729	-5.94914	200
	FUND. BIODIVER	1036793	Unknown	Aegilops ovata L.	L.	ESP	Spain	Sierra Tejeda		Ma	36.1	-3.1	
	FUND. BIODIVER	1043761	Unknown	Aegilops geniculata Roth	Roth	ESP	Spain	RincÃ³n de la Victoria		Ma	36.1	-4.1	
	FUND. BIODIVER	1067620	Unknown	Aegilops ovata		ESP	Spain	Coria del RÃ-o		Se	37.1	-5.1	
	FUND. BIODIVER	1073152	Unknown	Aegilops geniculata		ESP	Spain	DoÃ±a MencÃ-a		Co	37.1	-4.1	

00:00.0	FUND. BIODIVER	1835091	Unknown	Aegilops geniculata Roth	Roth	ESP	Spain	Almagro, volcan de Yezosa, sobre e		CR	38.1	-3.1	853
	FUND. BIODIVER	1891037	Unknown	Aegilops geniculata Roth	Roth	ESP	Spain	Formentera		PM	38.1	1.1	
00:00.0	REDIAM-CMA	88027	Observation	Aegilops geniculata		ESP	Spain		Antequera	Ma	37.0194	-4.51593	698
00:00.0	REDIAM-CMA	126117	Observation	Aegilops geniculata		ESP	Spain		AlcalÃ¡ la Real	J	37.4667	-3.90186	973
00:00.0	HSS	14723	Specimen	Aegilops geniculata Roth	Roth	ES	Spain	La Parra		Ba	38.5098	-6.64918	
	SIVIM	T-P16880:Aegi	Observation	Aegilops geniculata Roth	Roth	ES	Spain	CobeÃ±a			40.55	-3.59	0
	SIVIM	T-P16919:Aegi	Observation	Aegilops geniculata Roth	Roth	ES	Spain	Ciudad Universitaria			40.37	-3.7	0
	SIVIM	T-P20030:Aegi	Observation	Aegilops geniculata Roth	Roth	ES	Spain	El Arco (CaÃ±averal)			39.72	-6.43	0
	SIVIM	T-P26217:Aegi	Observation	Aegilops geniculata Roth	Roth	ES	Spain	RincÃ³n de Benizar, Moratalla			38.21	-1.97	1000
	SIVIM	T-P27682:Aegi	Observation	Aegilops geniculata Roth	Roth	ES	Spain	La Nava, SÂª de Cabra			37.48	-4.47	1020
	SIVIM	T-P29814:Aegi	Observation	Aegilops geniculata Roth	Roth	ES	Spain	Pr Sierra Monterilla, Requena, Valencia			39.28	-1.37	0
	SIVIM	S-P09956:Aegi	Observation	Aegilops geniculata Roth	Roth	ES	Spain	el Captivador (la Nucia)			38.54	-0.13	120
	SIVIM	S-P14062:Aegi	Observation	Aegilops geniculata Roth	Roth	ES	Spain	Serrinha			38.39	-8.54	0
	SIVIM	T-P01866:Aegi	Observation	Aegilops geniculata Roth	Roth	ES	Spain	Eivissa: Pla de Vila, prop de ca n'Escandel			38.83	1.38	0
	SIVIM	T-P03850:Aegi	Observation	Aegilops geniculata Roth	Roth	ES	Spain	2 km al NE de Villar del Campo			41.72	-2.15	1070
	SIVIM	T-P05777:Aegi	Observation	Aegilops geniculata Roth	Roth	ES	Spain	Embalse, HondÃ³n de las Nieves			38.19	-0.94	0
	SIVIM	T-P06426:Aegi	Observation	Aegilops geniculata Roth	Roth	ES	Spain	Los Herreros			39.28	-1.02	0
00:00.0	REDIAM-CMA	383988	Observation	Aegilops geniculata		ESP	Spain		Cambil	J	37.7313	-3.5076	1623
00:00.0	REDIAM-CMA	385159	Observation	Aegilops geniculata		ESP	Spain		Olvera	Ca	36.9542	-5.14035	640
00:00.0	REDIAM-CMA	386640	Observation	Aegilops geniculata		ESP	Spain		Cambil	J	37.6921	-3.51235	1052
00:00.0	REDIAM-CMA	394082	Observation	Aegilops geniculata		ESP	Spain		Chiclana de Segu	J	38.3371	-2.94535	507
00:00.0	GDA	GDA22403-1-2	Specimen	Aegilops geniculata Roth.	Roth.	ES	Spain	Granada, Cogollos Vega, proximida		GR			1100
00:00.0	GDA	GDA30016-1-1	Specimen	Aegilops ovata L.	L.	ES	Spain	Granada, Golilla de Cartuja.		GR			0
	SIVIM	P-P08486:Aegi	Observation	Aegilops geniculata Roth	Roth	ES	Spain	Mas Fontsanta; Vall-de-roures			40.7	0.04	1310
	SIVIM	P-P08875:Aegi	Observation	Aegilops geniculata Roth	Roth	ES	Spain	Tall Nou; Tortosa			40.7	0.15	1140
	SIVIM	P-P08989:Aegi	Observation	Aegilops geniculata Roth	Roth	ES	Spain	Embassament; la Pobla de BenifassÃ			40.61	0.16	0
	SIVIM	P-P10908:Aegi	Observation	Aegilops geniculata Roth	Roth	ES	Spain	Les Hortes, prop del SÃ©nia			40.61	0.16	0
	SIVIM	P-P12754:Aegi	Observation	Aegilops geniculata Roth	Roth	ES	Spain	Carretera de Santa MarÃ-a de Nieva			37.49	-2.09	1090
	IDBD-GN	42466	Observation	Aegilops geniculata Roth	Roth	ES	Spain	Montejurra	Ayegui	Na	42.6311	-2.04392	
	IDBD-GN	42486	Observation	Aegilops geniculata Roth	Roth	ES	Spain		Araia	Vi	42.8852	-2.32165	600
	IDBD-GN	42508	Observation	Aegilops geniculata Roth	Roth	ES	Spain		Lumbier	Na	42.6403	-1.28754	480
00:00.0	BC	92758	Specimen	Aegilops geniculata Roth	Roth	ES	Spain	El Arahal; Empalme de MorÃ³n Cad		Se	37.15	-5.65	
00:00.0	BC	87553	Specimen	Aegilops ovata L.	L.	ES	Spain	Palma de Mallorca; Illa de Cabrera:		PM	39.16	2.94	
	RUS001	VIR100601735	Specimen	Aegilops ovata L.		ESP	Spain						
	FUND. BIODIVER	1370920	Unknown	Aegilops geniculata Roth	Roth	ESP	Spain	San AgustÃ-n de Guadalix		M	40.1	-3.1	
	FUND. BIODIVER	1463982	Unknown	Aegilops geniculata Roth	Roth	ESP	Spain	Santa Maria de MeiÃ		L	41.1	0.1	630
	ADIMAN	24	Observation	Aegilops geniculata		ESP	Spain	EnguÃ-danos		CU	39.6552	-1.57623	
	FUND. BIODIVER	1593211	Unknown	Aegilops geniculata Roth.	Roth.	ESP	Spain	Guardo		P	42.1	-4.1	
00:00.0	SALA	87727-1	Specimen	Aegilops geniculata Roth	Roth	ES	Spain	_; Mestanza, sierra de Puertollano,		CR	38.6	-4.08	
00:00.0	HSS	239	Specimen	Aegilops geniculata Roth	Roth	ES	Spain	Solana de los Barros, ctra. de Alme		Ba	38.6875	-6.52841	
00:00.0	REDIAM-CMA	223713	Observation	Aegilops geniculata		ESP	Spain		AlbuÃ±uelas	Gr	36.8756	-3.73746	1298

00:00.0	REDIAM-CMA	232678	Observation	Aegilops geniculata		ESP	Spain		AlmadÃ©n de la F	Se	37.8842	-5.99781	423
	SIVIM	T-P06446:Aegi	Observation	Aegilops geniculata Roth	Roth	ES	Spain	Alcublas			39.72	-0.78	0
	SIVIM	T-P07219:Aegi	Observation	Aegilops geniculata Roth	Roth	ES	Spain	Capella			42.15	0.33	0
	SIVIM	T-P09208:Aegi	Observation	Aegilops geniculata Roth	Roth	ES	Spain	Soto del Cerrato			41	-4.42	0
	SIVIM	T-P10276:Aegi	Observation	Aegilops geniculata Roth	Roth	ES	Spain	Puerto La Olla (Los Villares)			37.58	-3.9	1360
	SIVIM	T-P11412:Aegi	Observation	Aegilops geniculata Roth	Roth	ES	Spain	CaÃ±ete a Boniches			39.92	-1.71	1050
00:00.0	LEB	4786-1	Specimen	Aegilops geniculata Roth	Roth	ES	Spain	MiÃ±era de Luna		Le	42.91	-5.88	1
00:00.0	COFC	46892-1	Specimen	Aegilops geniculata Roth	Roth	ES	Spain	pantano del Retortillo		Co			1
	FUND. BIODIVER	1002566	Unknown	Aegilops geniculata		ESP	Spain	Bosque de Alpera		Ab	38.1	-1.1	
	FUND. BIODIVER	1025029	Unknown	Aegilops geniculata		ESP	Spain	Alcaudete		J	37.1	-3.1	
	FUND. BIODIVER	1811873	Unknown	Aegilops geniculata Roth	Roth	ESP	Spain	Tudela		Na	41.1	-1.1	
00:00.0	ABH	19004-1	Specimen	Aegilops geniculata Roth	Roth	ES	Spain	Jijona; Monnegre, MolÃ- Capeta		A	38.48	-0.52	
	SIVIM	T-P13736:Aegi	Observation	Aegilops geniculata Roth	Roth	ES	Spain	Cuartel-Balsa de la Huerta, Mosqueruela			40.35	-0.52	1420
	SIVIM	T-P16915:Aegi	Observation	Aegilops geniculata Roth	Roth	ES	Spain	Ajaivir			40.46	-3.58	0
	SIVIM	T-P19972:Aegi	Observation	Aegilops geniculata Roth	Roth	ES	Spain	Coria			39.9	-6.54	263
	SIVIM	T-P25262:Aegi	Observation	Aegilops geniculata Roth	Roth	ES	Spain	Garrigues de Son serra Marina (Mallorca)			39.65	3	0
	SIVIM	T-P27629:Aegi	Observation	Aegilops geniculata Roth	Roth	ES	Spain	Pto. Cerezo (SÂª Horconera)			37.3	-4.35	1300
	SIVIM	T-P28646:Aegi	Observation	Aegilops geniculata Roth	Roth	ES	Spain	Corchuelas de MonfragÃ¼e, TorrejÃ³n el R			39.71	-6.2	300
00:00.0	L	8180	Specimen	Aegilops geniculata Roth		Spain	Spain				42.19	0.12	0
00:00.0	SEV	99074-1	Specimen	Aegilops geniculata Roth	Roth	ES	Spain	PeÃ±Ã³n de AlgÃ¡mitas		Se			1
00:00.0	REDIAM-CMA	328504	Observation	Aegilops geniculata		ESP	Spain		Casares	Ma	36.4752	-5.28967	500
00:00.0	REDIAM-CMA	382098	Observation	Aegilops geniculata		ESP	Spain		Cambil	J	37.6842	-3.53441	899
00:00.0	REDIAM-CMA	383037	Observation	Aegilops geniculata		ESP	Spain		Torres	J	37.7537	-3.49555	1206
00:00.0	BDBCV	99	Observation	Aegilops geniculata		ESP	Spain	Parque Natural de Penyagolosa		Cs	40.2	-0.39	
00:00.0	GDA	GDA19440-1	Specimen	Aegilops geniculata Roth.	Roth.	ES	Spain	JaÃ©n, Colegio Universitario.		J			440
	SIVIM	U-P09949:Aegi	Observation	Aegilops geniculata Roth	Roth	ES	Spain	Cuneta camino Villahermosa			38.66	-2.88	0
	BC	70786	Specimen	Aegilops ovata L.	L.	ES	Spain	Teruel; Teruel		Te	40.32	-1.06	
00:00.0	BC	92738	Specimen	Aegilops geniculata Roth	Roth	ES	Spain	Benaocaz; Benaocaz		Ca	36.71	-5.41	1000
00:00.0	BC	92740	Specimen	Aegilops geniculata Roth	Roth	ES	Spain	Grazalema; Grazalema CÃ¡diz		Ca	36.8	-5.41	
	RUS001	VIR100602107	Specimen	Aegilops ovata L.		ESP	Spain						
	RUS001	VIR100602086	Specimen	Aegilops ovata L.		ESP	Spain						
00:00.0	SALA	122461-1	Specimen	Aegilops geniculata Roth	Roth	ES	Spain	Aldeanueva de Figueroa, pinar dere		Sa			
00:00.0	COA	41159-1	Specimen	Aegilops geniculata Roth	Roth	ES	Spain	Junto a Porvenir		Co	38.28	-5.4	
	FUND. BIODIVER	1093257	Unknown	Aegilops geniculata		ESP	Spain	El Arco, CaÃ±averal		Cc	39.1	-6.1	
00:00.0	FUND. BIODIVER	1946399	Unknown	Aegilops geniculata Roth	Roth	ESP	Spain	Solana del Pino, alrededores		CR	38.1	-3.1	800
	IDBD-GN	42422	Observation	Aegilops geniculata Roth	Roth	ES	Spain	Rocaforte	SangÃ¼esa	Na	42.5947	-1.25222	560
	IDBD-GN	42445	Observation	Aegilops geniculata Roth	Roth	ES	Spain	Iranzu	AbÃ¡rzuza	Na	42.762	-2.08468	
	ESP004	NC050472	Specimen	Aegilops geniculata Roth		ESP	Spain	Cuenca/Carrascosa del Campo 5km W, Fu			40.0833	-2.21667	960
00:00.0	REDIAM-CMA	1615	Observation	Aegilops geniculata		ESP	Spain		Teba	Ma	36.9582	-4.89519	400
00:00.0	REDIAM-CMA	10448	Observation	Aegilops geniculata		ESP	Spain		Casares	Ma	36.4311	-5.24538	274
00:00.0	REDIAM-CMA	18519	Observation	Aegilops geniculata		ESP	Spain		Almonaster la Re	H	37.858	-6.85377	500

00:00.0	REDIAM-CMA	25358	Observation	Aegilops geniculata		ESP	Spain		AlbÃ¡nchez de M	J	37.7874	-3.4867	1200
	REDIAM-CMA	396732	Observation	Aegilops geniculata		ESP	Spain		Ubrique	Ca	36.6405	-5.44642	361
00:00.0	REDIAM-CMA	402583	Observation	Aegilops geniculata		ESP	Spain		QuÃ©ntar	Gr	37.2481	-3.40559	1300
00:00.0	REDIAM-CMA	420189	Observation	Aegilops geniculata		ESP	Spain		Murtas	Gr	36.8564	-3.12628	1191
00:00.0	SALA	57187-1	Specimen	Aegilops geniculata Roth	Roth	ES	Spain	_; Maguilla		Ba			
	ESP004	NC043861	Specimen	Aegilops geniculata Roth		ESP	Spain	Minas de Aldeamoret, Caceres, province of			39.45	-6.38333	451
	IPK	AE 1049	Living	Aegilops geniculata Roth			Spain	Cortiguera (Burgos)			42.7831	-3.28333	
00:00.0	BC	805249	Specimen	Aegilops geniculata Roth	Roth	ES	Spain	el Bruc; Anoia: el Bruc, a l'E de can		B	41.586	1.794	490
	FUND. BIODIVER	1125485	Unknown	Aegilops ovata L.	L.	ESP	Spain	Laguna de Duero		Va	41.1	-4.1	
	FUND. BIODIVER	36856	Unknown	Aegilops geniculata Roth	Roth	ESP	Spain	Araia		Vi	42.1	-2.1	600
	FUND. BIODIVER	62019	Unknown	Aegilops ovata subsp. ovata L.	L.	ESP	Spain	Ciudad Rodrigo		Sa	40.1	-6.1	
	SIVIM	Q-P03038:Aegi	Observation	Aegilops geniculata Roth	Roth	ES	Spain	St. MagÃ- de Brufaganya, prop del santuar			41.44	1.32	0
	SIVIM	Q-P03605:Aegi	Observation	Aegilops geniculata Roth	Roth	ES	Spain	Zucaina			40.08	-0.41	0
	SIVIM	Q-P06541:Aegi	Observation	Aegilops geniculata Roth	Roth	ES	Spain	Els Segalassos, cap a Vilanova de Prades			41.26	0.85	0
	SIVIM	R-P00052:Aegi	Observation	Aegilops geniculata Roth	Roth	ES	Spain	cap al Pont de Montanyana (Montsec d'Est			42.06	0.58	0
	SIVIM	R-P06587:Aegi	Observation	Aegilops geniculata Roth	Roth	ES	Spain	Villa de Ves. S' del BoquerÃ³n. Vado.			39.1	-1.26	0
	SIVIM	R-P08267:Aegi	Observation	Aegilops geniculata Roth	Roth	ES	Spain	Sota can SolÃ de la Balma (el Bruc)			41.54	1.68	0
	FUND. BIODIVER	1120223	Unknown	Aegilops geniculata Roth	Roth	ESP	Spain	Santa MarÃ-a de la Vega		Za	41.1	-5.1	
	FUND. BIODIVER	115632	Unknown	Aegilops geniculata Roth	Roth	ESP	Spain	Guardo, MuÃ±eca		P	42.1	-4.1	
	FUND. BIODIVER	1172116	Unknown	Aegilops ovata L.	L.	ESP	Spain	Rivera de Piernaschicas		Ba			
00:00.0	FUND. BIODIVER	1972307	Unknown	Aegilops geniculata		ESP	Spain	Valle de Escuain, Puertolas, Proxim		Hu	42.1	0.9	1980
00:00.0	REDIAM-CMA	29611	Observation	Aegilops geniculata		ESP	Spain		Torres	J	37.747	-3.52837	1500
00:00.0	REDIAM-CMA	47506	Observation	Aegilops geniculata		ESP	Spain		Torres	J	37.7455	-3.53905	1393
00:00.0	REDIAM-CMA	63143	Observation	Aegilops geniculata		ESP	Spain		Carcabuey	Co	37.4067	-4.30876	753
00:00.0	REDIAM-CMA	71795	Observation	Aegilops geniculata		ESP	Spain		AlcalÃ¡ de los Ga	Ca	36.4872	-5.77109	100
00:00.0	REDIAM-CMA	45264	Observation	Aegilops geniculata		ESP	Spain		Huelma	J	37.7108	-3.46755	1670
00:00.0	REDIAM-CMA	51210	Observation	Aegilops geniculata		ESP	Spain		Torres	J	37.7454	-3.55119	1291
00:00.0	REDIAM-CMA	69667	Observation	Aegilops geniculata		ESP	Spain		Torres	J	37.7496	-3.51124	1250
00:00.0	ABH	538-1	Specimen	Aegilops geniculata Roth	Roth	ES	Spain	Benissa; Serra de BÃ¨rnia		A	38.66	-0.05	
	SIVIM	R-P09693:Aegi	Observation	Aegilops geniculata Roth	Roth	ES	Spain	entre Valdelcubo y Barahona			41.19	-2.76	1130
	SIVIM	R-P10576:Aegi	Observation	Aegilops geniculata Roth	Roth	ES	Spain	Cruce de Medina Sidonia a Paterna de Riv			36.46	-6.01	0
	SIVIM	R-P11714:Aegi	Observation	Aegilops geniculata Roth	Roth	ES	Spain	Cigales			41.71	-4.8	0
	SIVIM	S-P01258:Aegi	Observation	Aegilops geniculata Roth	Roth	ES	Spain	l'Espluga Calba, coll de Portelles			41.44	0.96	0
	SIVIM	S-P03975:Aegi	Observation	Aegilops geniculata Roth	Roth	ES	Spain	la Pobla de Ferran, afores del poble			41.53	1.08	0
00:00.0	SEV	108392-1	Specimen	Aegilops ovata L.	L.	ES	Spain	Los Barrios		Ca			160
	SIVIM	R-P09631:Aegi	Observation	Aegilops geniculata Roth	Roth	ES	Spain	Riba de Santiuste			41.19	-2.76	1100
	SIVIM	R-P10455:Aegi	Observation	Aegilops geniculata Roth	Roth	ES	Spain	K10 de la carretera del Picacho , AG			36.47	-5.67	0
	SIVIM	R-P11655:Aegi	Observation	Aegilops geniculata Roth	Roth	ES	Spain	Tarifa			44.93	-6.04	0
	SIVIM	S-P01247:Aegi	Observation	Aegilops geniculata Roth	Roth	ES	Spain	l'Espluga Calba, els Bassals			41.44	0.96	0
	SIVIM	S-P02150:Aegi	Observation	Aegilops geniculata Roth	Roth	ES	Spain	Sant Ermengol (Aletorn)			41.89	0.95	0
	SIVIM	S-P03965:Aegi	Observation	Aegilops geniculata Roth	Roth	ES	Spain	l'Espluga Calba, Pla de la Creu			41.44	0.96	0

00:00.0	COFC	11989-1	Specimen	Aegilops geniculata Roth	Roth	ES	Spain	Priego de CÃƒÂ³rdoba; cerro Gallar		Co	37	-4	1
00:00.0	BDBCV	112	Observation	Aegilops geniculata		ESP	Spain	Parque Natural de Penyagolosa		Cs			
	RUS001	VIR100602120	Specimen	Aegilops ovata L.		ESP	Spain						
	RUS001	VIR100602099	Specimen	Aegilops ovata L.		ESP	Spain						
	RUS001	VIR100602078	Specimen	Aegilops ovata L.		ESP	Spain						
	RUS001	VIR100602179	Specimen	Aegilops ovata L.		ESP	Spain						
	FUND. BIODIVER	109362	Unknown	Aegilops geniculata Roth.	Roth.	ESP	Spain	Los Barrios de Luna, MiÃ±era		Le	42.1	-5.1	
	FUND. BIODIVER	1946386	Unknown	Aegilops geniculata Roth	Roth	ESP	Spain	Despenaperros		CR	38.1	-3.1	
00:00.0	FUND. BIODIVER	1946407	Unknown	Aegilops geniculata Roth	Roth	ESP	Spain	Almagro, volcan de Yezosa, sobre e		CR	38.1	-3.1	853
00:00.0	SALA	3449-1	Specimen	Aegilops ovata L.	L.	ES	Spain	_; Castellanos de Villiquera		Sa			
00:00.0	BDBCV	590455	Observation	Aegilops geniculata		ESP	Spain		Bocairent	Valenc	38.729	-0.544	
	FUND. BIODIVER	1035513	Unknown	Aegilops ovata		ESP	Spain	Base suroriental de la Sierra de Agu		Ma	36.1	-4.1	
	FUND. BIODIVER	1038228	Unknown	Aegilops geniculata Roth	Roth	ESP	Spain	Sierra de Mijas		Ma	36.1	-4.1	
	FUND. BIODIVER	1835078	Unknown	Aegilops geniculata Roth	Roth	ESP	Spain	Provincia de Ciudad Real		CR			
00:00.0	FUND. BIODIVER	1684420	Unknown	Aegilops ovata L.	L.	ESP	Spain	Los Espartales, junto a charco		Ca	36.1	-5.1	
00:00.0	REDIAM-CMA	404586	Observation	Aegilops geniculata		ESP	Spain		AlmadÃ©n de la F	Se	37.8523	-6.00545	278
00:00.0	REDIAM-CMA	414418	Observation	Aegilops geniculata		ESP	Spain		Espiel	Co	38.099	-4.99319	497
00:00.0	REDIAM-CMA	416912	Observation	Aegilops geniculata		ESP	Spain		Tabernas	Al	37.0933	-2.33977	509
00:00.0	REDIAM-CMA	383394	Observation	Aegilops geniculata		ESP	Spain		Ronda	Ma	36.8286	-5.2415	909
00:00.0	REDIAM-CMA	384438	Observation	Aegilops geniculata		ESP	Spain				37.6384	-3.06367	
00:00.0	REDIAM-CMA	385781	Observation	Aegilops geniculata		ESP	Spain		Antequera	Ma	36.9554	-4.57902	994
00:00.0	REDIAM-CMA	388726	Observation	Aegilops geniculata		ESP	Spain		Torres	J	37.7505	-3.53028	1495
00:00.0	REDIAM-CMA	392018	Observation	Aegilops geniculata		ESP	Spain		Algodonales	Ca	36.9169	-5.40207	591
00:00.0	REDIAM-CMA	250472	Observation	Aegilops geniculata		ESP	Spain		VÃ©lez-Blanco	Al	37.8506	-2.07187	900
	SIVIM	Q-P03051:Aegi	Observation	Aegilops geniculata Roth	Roth	ES	Spain	Castell de Queralt, a l'oest de Sta. Maria de			41.45	1.44	0
	SIVIM	Q-P04292:Aegi	Observation	Aegilops geniculata Roth	Roth	ES	Spain	camÃ- del Boixar a Fredes, El Boixar			40.61	0.04	1150
	SIVIM	Q-P07408:Aegi	Observation	Aegilops geniculata Roth	Roth	ES	Spain	Finca Navodres			39.65	-2.06	0
	SIVIM	R-P02684:Aegi	Observation	Aegilops geniculata Roth	Roth	ES	Spain	rodalies de la bassa Espartera			41.43	0.48	160
	SIVIM	R-P06815:Aegi	Observation	Aegilops geniculata Roth	Roth	ES	Spain	AlcalÃ¡ del JÃºcar, hacia Villavaliente, Alba			39.1	-1.49	0
	SIVIM	R-P08597:Aegi	Observation	Aegilops geniculata Roth	Roth	ES	Spain	30 S WF 3082			36.86	-2.66	1050
	SIVIM	P-P03000:Aegi	Observation	Aegilops geniculata Roth	Roth	ES	Spain	tossal de la vall de SecÃ , prop Salouet (To			41.43	0.48	160
	SIVIM	P-P08863:Aegi	Observation	Aegilops geniculata Roth	Roth	ES	Spain	PantÃ d'Ulldecona; la Pobla de BenifassÃ			40.61	0.16	0
	SIVIM	P-P08895:Aegi	Observation	Aegilops geniculata Roth	Roth	ES	Spain	Mas de la Cova; Arnes			40.79	0.15	0
	SIVIM	P-P09083:Aegi	Observation	Aegilops geniculata Roth	Roth	ES	Spain	PantÃ d'Ulldecona; la Pobla de BenifassÃ			40.61	0.16	0
	SIVIM	P-P11105:Aegi	Observation	Aegilops geniculata Roth	Roth	ES	Spain	Puig de la MisericÃ²rdia			40.44	0.4	160
	SIVIM	Q-P00502:Aegi	Observation	Aegilops geniculata Roth	Roth	ES	Spain	Entre BellvÃ-s i Vilanova de la Barca			41.61	0.71	0
	SIVIM	T-P30043:Aegi	Observation	Aegilops geniculata Roth	Roth	ES	Spain	Pr. Corral de Caracoles, Balsa de Ves, Alb			39.28	-1.26	0
	SIVIM	T-P30065:Aegi	Observation	Aegilops geniculata Roth	Roth	ES	Spain	Camino al Regajo, Jalance, Valencia			39.1	-1.14	0
	SIVIM	U-P02176:Aegi	Observation	Aegilops geniculata Roth	Roth	ES	Spain	E, PuÃ©rtolas, refugio de cueva Foratata			42.59	0.07	2020
	SIVIM	U-P02913:Aegi	Observation	Aegilops geniculata Roth	Roth	ES	Spain	Castells de Serrella			38.72	-0.23	0
	SIVIM	U-P06654:Aegi	Observation	Aegilops geniculata Roth	Roth	ES	Spain	Benafim			37.22	-8.21	2500

	SIVIM	U-P07721:Aegi	Observation	Aegilops geniculata Roth	Roth	ES	Spain	CastellÃ³n: Oropesa del Mar			40.07	0.06	30
00:00.0	GDA	GDA12219-1-2	Specimen	Aegilops geniculata Roth.	Roth.	ES	Spain	Granada, SÂª Nevada, CÃ¡Ã±ar, ba		GR			1100
00:00.0	GDA	GDA13286-1-2	Specimen	Aegilops geniculata Roth.	Roth.	ES	Spain	MÃ¡laga, CoÃn, la AlbuquerÃa.		MA			0
00:00.0	GDA	GDA25153-1-2	Specimen	Aegilops geniculata Roth.	Roth.	ES	Spain	Granada, Pedro MartÃnez, Mencal		GR			1150
00:00.0	BDBCV	331661	Observation	Aegilops geniculata	_	ES	Spain		La Nucia	A	38.59	-0.07	
00:00.0	ABH	11575-1	Specimen	Aegilops geniculata Roth	Roth	ES	Spain	Tibi; RÃo Verde, Finca Terol		A	38.55	-0.61	
	IDBD-GN	42482	Observation	Aegilops geniculata Roth	Roth	ES	Spain	Matacalva	S. MartÃn de Un	Na	42.5184	-1.59522	500
	IDBD-GN	42495	Observation	Aegilops geniculata Roth	Roth	ES	Spain		Arguedas	Na	42.1839	-1.49367	250
	BDBCV-General	276667	Observation	Aegilops geniculata		ESPA	Spain	NavarrÃ©s	La Canal de Nava	Valenc	39.0517	-0.74664	
00:00.0	COA	41165-1	Specimen	Aegilops geniculata Roth	Roth	ES	Spain	A 5 Km de Moriles		Co	37.39	-4.69	
	CZE122	01C2109015	Specimen	Aegilops geniculata Roth		ESP	Spain	Pablo Romaso, Strasse Sevilla - Huelva, 4 km of Sanlucar la Mayor, Sevilla					
	SANT	19359	Specimen	Aegilops geniculata Roth		ES	Spain	Monforte,As Barrioncas		Lu			
	BDBCV-General	276664	Observation	Aegilops geniculata		ESPA	Spain	NavarrÃ©s	La Canal de Nava	Valenc	39.0517	-0.74664	
00:00.0	SALA	66762-1	Specimen	Aegilops geniculata Roth	Roth	ES	Spain	_; BÃ³veda del RÃo Almar		Sa	40.85	-5.22	
00:00.0	GDA	GDA22404-1	Specimen	Aegilops geniculata Roth.	Roth.	ES	Spain	Granada, Cogollos Vega, proximida		GR			1000
00:00.0	MGC	54818-1	Unknown	Aegilops geniculata Roth	Roth	ES	Spain	Estepona; Sierra Bermeja. Por enci		Ma	0	0	240
00:00.0	W	42292	Unknown	Aegilops ovata L.		ESP	Spain	Flora der Balearen. Mallorca: Estellence (N von); 100m					100
	BC	92731	Specimen	Aegilops ovata L.	L.	ES	Spain	AlcarrÃ s; Montagut		L	41.66	0.54	
	BC	92734	Specimen	Aegilops ovata L.	L.	ES	Spain	Sant Josep de sa Talaia; Les Saline		PM	38.88	1.33	
00:00.0	GDA	GDA15905-1	Specimen	Aegilops geniculata Roth.	Roth.	ES	Spain	Granada, SÂª Pelada, barranco de		GR			980
00:00.0	MA	636847-1	Specimen	Aegilops geniculata		ES	Spain	La Granja de San Ildefonso, a 1,300		Sg	40	-4	
00:00.0	SEV	9871-1	Specimen	Aegilops ovata L.	L.	ES	Spain	Carretera de Jerez a Medina cerca		Ca			1
00:00.0	SEV	98931-1	Specimen	Aegilops geniculata Roth	Roth	ES	Spain	Entre VillamartÃn y Prado del Rey		Ca			1
00:00.0	COA	41206-1	Specimen	Aegilops geniculata Roth	Roth	ES	Spain	Sierra Madrona		CR	38.39	-4.26	
	FUND. BIODIVER	1036000	Unknown	Aegilops ovata L.	L.	ESP	Spain	Sierra de Aguas, Carratraca		Ma	36.1	-4.1	
	FUND. BIODIVER	1043757	Unknown	Aegilops geniculata Roth	Roth	ESP	Spain	Sierra de Mijas		Ma	36.1	-4.1	
	FUND. BIODIVER	1072128	Unknown	Aegilops ovata L.	L.	ESP	Spain	Arroyo del Salado, cerca de MorÃ³n		Se	36.1	-5.1	
00:00.0	FUND. BIODIVER	1835086	Unknown	Aegilops geniculata Roth	Roth	ESP	Spain	Villanueva de la Fuente		CR	38.1	-2.1	1000
00:00.0	REDIAM-CMA	383851	Observation	Aegilops geniculata		ESP	Spain		Olvera	Ca	36.9632	-5.36668	300
00:00.0	REDIAM-CMA	386521	Observation	Aegilops geniculata		ESP	Spain		Pruna	Se	37.02	-5.18743	786
00:00.0	REDIAM-CMA	387878	Observation	Aegilops geniculata		ESP	Spain		Medina-Sidonia	Ca	36.4334	-5.84542	109
00:00.0	REDIAM-CMA	393245	Observation	Aegilops geniculata		ESP	Spain		Cambil	J	37.732	-3.50594	1631
00:00.0	COFC	26932-1	Specimen	Aegilops geniculata Roth	Roth	ES	Spain	CardeÃƒÂ±a; coto 'Atalayones'		Co			1
00:00.0	COFC	29013-1	Specimen	Aegilops geniculata Roth	Roth	ES	Spain	BaÃƒÂ±os de la Encina; coto 'Nuev		J			1
	IPK	AE 485	Living	Aegilops geniculata Roth			Spain	Nocito Espagne			42.3219	-0.74472	
	SIVIM	P-P08871:Aegi	Observation	Aegilops geniculata Roth	Roth	ES	Spain	El Retaule, capÃ§alera; la SÃ©nia			40.7	0.27	1130
	SIVIM	P-P08922:Aegi	Observation	Aegilops geniculata Roth	Roth	ES	Spain	Camps d'en Ferrer; el Boixar			40.61	0.04	1090
	SIVIM	P-P09743:Aegi	Observation	Aegilops geniculata Roth	Roth	ES	Spain	Barranc de Capatx; la SÃ©nia			40.7	0.27	1170
	SIVIM	P-P12679:Aegi	Observation	Aegilops geniculata Roth	Roth	ES	Spain	Sierra de MarÃa			37.67	-2.31	1100
	SIVIM	Q-P03034:Aegi	Observation	Aegilops geniculata Roth	Roth	ES	Spain	La Llacuna, carrer no pavimentat que va de			41.45	1.44	0
	FUND. BIODIVER	1489076	Unknown	Aegilops geniculata Roth	Roth	ESP	Spain	P.N. Tablas de Daimiel		CR	38.1	-3.1	

	FUND. BIODIVER	1549511	Unknown	Aegilops ovata		ESP	Spain	Cantabr. (Durango)		Bi	42.1	-2.1	
	FUND. BIODIVER	1603148	Unknown	Aegilops ovata		ESP	Spain	la Lora		P			
	FUND. BIODIVER	1643500	Unknown	Aegilops geniculata Roth	Roth	ESP	Spain	PeÃ±aflor, San CristÃ³bal		Z	41.1	-0.1	300
	FUND. BIODIVER	979131	Unknown	Aegilops ovata L.	L.	ESP	Spain	Tramacastilla		Te	40.1	-1.1	
	FUND. BIODIVER	992693	Unknown	Aegilops ovata L.	L.	ESP	Spain	Tartanedo		Gu	40.1	-1.1	
00:00.0	BC	601432	Specimen	Aegilops ovata L.	L.	ES	Spain	Prades; Muntanyes de Prades: entre		T	41.31	1.03	950
00:00.0	BDBCV	107	Observation	Aegilops geniculata		ESP	Spain	Parque Natural de Penyagolosa		Cs	40.17	-0.4	
00:00.0	BC	800515	Specimen	Aegilops geniculata Roth	Roth	ES	Spain	Gavet de la Conca; Montsec de RÃº		L	42.043	1	1100
00:00.0	REDIAM-CMA	202551	Observation	Aegilops geniculata		ESP	Spain		Torres de AlbÃ¡nc	J	38.4208	-2.65416	700
00:00.0	REDIAM-CMA	216357	Observation	Aegilops geniculata		ESP	Spain		Gor	Gr	37.3964	-2.94029	1283
00:00.0	REDIAM-CMA	229671	Observation	Aegilops geniculata		ESP	Spain		Felix	Al	36.9053	-2.71794	1405
00:00.0	SEV	98679-1	Specimen	Aegilops geniculata Roth	Roth	ES	Spain	Luque, entre Casilla La Lastra y La		Co			1
	GDA	GDA42773-1-2	Specimen	Aegilops geniculata Roth.	Roth.	ES	Spain	Granada, CÃºllar Baza, El Margen,		GR			872
	GDA	GDA43468-1-2	Specimen	Aegilops geniculata Roth.	Roth.	ES	Spain	Granada, Guadix, Rambla Becerra.		GR			950
00:00.0	HUAL	9705-1	Specimen	Aegilops geniculata Roth	Roth	ES	Spain	LubrÃ-n; Sierra de BÃ©dar. El Cam		Al	37.39	-2.47	580
00:00.0	HSS	8982	Specimen	Aegilops geniculata Roth	Roth	ES	Spain	Ctra. AlÃ-a - Puerto de San Vicente		Cc	39.4147	-5.14896	
	SIVIM	T-P13691:Aegi	Observation	Aegilops geniculata Roth	Roth	ES	Spain	MasÃ-a de la Tosquilla, Iglesuela del Cid			40.44	-0.4	1300
	SIVIM	T-P16885:Aegi	Observation	Aegilops geniculata Roth	Roth	ES	Spain	Villaviciosa de OdÃ³n			40.28	-3.94	0
	SIVIM	T-P19061:Aegi	Observation	Aegilops geniculata Roth	Roth	ES	Spain	Cerro de Calabozo			37.22	-3.45	1680
	SIVIM	T-P20231:Aegi	Observation	Aegilops geniculata Roth	Roth	ES	Spain	DoÃ±a MencÃ-a			37.48	-4.35	0
	SIVIM	T-P26224:Aegi	Observation	Aegilops geniculata Roth	Roth	ES	Spain	Proximidades de Biar, Biar			38.55	-0.81	0
	SIVIM	T-P28624:Aegi	Observation	Aegilops geniculata Roth	Roth	ES	Spain	Majadas de la Cruz, TorrejÃ³n el Rubio			39.71	-6.2	340
	FUND. BIODIVER	1043756	Unknown	Aegilops geniculata Roth	Roth	ESP	Spain	MÃ¡laga		Ma	36.1	-4.1	
00:00.0	FUND. BIODIVER	1835085	Unknown	Aegilops geniculata Roth	Roth	ESP	Spain	Villanueva de San Carlos, arroyo Ca		CR	38.1	-3.1	600
00:00.0	REDIAM-CMA	383785	Observation	Aegilops geniculata		ESP	Spain		Cambil	J	37.7325	-3.50661	1627
00:00.0	REDIAM-CMA	384847	Observation	Aegilops geniculata		ESP	Spain		Jimena	J	37.8178	-3.46978	829
00:00.0	REDIAM-CMA	386470	Observation	Aegilops geniculata		ESP	Spain		Torres	J	37.7434	-3.51151	1400
00:00.0	REDIAM-CMA	387848	Observation	Aegilops geniculata		ESP	Spain		Huelma	J	37.7123	-3.46576	1645
00:00.0	REDIAM-CMA	389270	Observation	Aegilops geniculata		ESP	Spain		Alhama de Grana	Gr	37.0319	-4.18365	1465
	IPK	AE 588	Living	Aegilops geniculata Roth			Spain	Pablo Romaso, Stra?e Sevilla - Huelva, 4 k			37.3969	-6.75722	40
	IPK	AE 777	Living	Aegilops geniculata Roth subsp. gibberosa (Zhuk.) K			Spain	W.-Pyrenaen, b. Jaca			42.5719	-0.44972	810
	FUND. BIODIVER	1643504	Unknown	Aegilops geniculata Roth	Roth	ESP	Spain	Plana de Zaragoza, Acampo		Z	41.1	-0.1	600
	FUND. BIODIVER	1648485	Unknown	Aegilops geniculata Roth	Roth	ESP	Spain	Cantavieja, pista a Tarayuela		Te	40.1	-0.1	
00:00.0	FUND. BIODIVER	1676973	Unknown	Aegilops ovata L.	L.	ESP	Spain	Archipielago de Cabrera, isla de Ca		PM	38.1	2.1	
	SIVIM	P-P08870:Aegi	Observation	Aegilops geniculata Roth	Roth	ES	Spain	Plana de la Refoia; PaÃ¼ls			40.89	0.38	0
	SIVIM	P-P08921:Aegi	Observation	Aegilops geniculata Roth	Roth	ES	Spain	Cova del Vidre; Roquetes			40.7	0.27	1040
	SIVIM	P-P09135:Aegi	Observation	Aegilops geniculata Roth	Roth	ES	Spain	Pinar Pla; Fredes			40.7	0.15	1170
	SIVIM	P-P11409:Aegi	Observation	Aegilops geniculata Roth	Roth	ES	Spain	Lo Portell (al Canal del Grau)			40.89	0.38	0
00:00.0	REDIAM-CMA	273018	Observation	Aegilops geniculata		ESP	Spain		CardeÃ±a	Co	38.371	-4.45114	645
00:00.0	REDIAM-CMA	289552	Observation	Aegilops geniculata		ESP	Spain		Chiclana de Segu	J	38.4064	-2.90474	605
	ESP004	NC050499	Specimen	Aegilops geniculata Roth		ESP	Spain	CaÃƒÂ±averas/Priego 5km NE, Villaconejo			40.4	-2.33333	880

	RUS001	VIR100602184	Specimen	Aegilops ovata L.		ESP	Spain						
	SIVIM	T-P30034:Aegi	Observation	Aegilops geniculata Roth	Roth	ES	Spain	Presa del Molinar, Villa de Ves, Albacete			39.19	-1.26	0
	SIVIM	T-P30059:Aegi	Observation	Aegilops geniculata Roth	Roth	ES	Spain	El Campichuelo, Cofrentes, Valencia			39.19	-1.14	0
	SIVIM	T-P32181:Aegi	Observation	Aegilops geniculata Roth	Roth	ES	Spain	Barranc d'Aiguaoliva			40.8	0.39	0
	SIVIM	U-P02908:Aegi	Observation	Aegilops geniculata Roth	Roth	ES	Spain	Vall d'AlcalÃ , Beniaia			38.72	-0.35	0
	SIVIM	U-P06648:Aegi	Observation	Aegilops geniculata Roth	Roth	ES	Spain	Benafim			37.22	-8.21	0
	SIVIM	U-P07715:Aegi	Observation	Aegilops geniculata Roth	Roth	ES	Spain	Valencia: Massamagrell, El Bogalar			39.54	-0.43	0
	FUND. BIODIVER	1141316	Unknown	Aegilops ovata subsp. ovata L.	L.	ESP	Spain	Virgen del Camino		Le	42.1	-5.1	
	FUND. BIODIVER	116427	Unknown	Aegilops geniculata Roth.	Roth.	ESP	Spain	Ãscar		Va	41.1	-4.1	
	FUND. BIODIVER	36860	Unknown	Aegilops geniculata Roth	Roth	ESP	Spain	Labastida		Vi	42.1	-2.1	500
	RUS001	VIR100602116	Specimen	Aegilops ovata L.		ESP	Spain						
	RUS001	VIR100602095	Specimen	Aegilops ovata L.		ESP	Spain						
	RUS001	VIR100602074	Specimen	Aegilops ovata L.		ESP	Spain						
	FUND. BIODIVER	1050672	Unknown	Aegilops ovata		ESP	Spain	Ubrique		Ca	36.1	-5.1	
	FUND. BIODIVER	1067624	Unknown	Aegilops ovata L.	L.	ESP	Spain	AlcalÃ¡ de Guadaira		Se	37.1	-5.1	
00:00.0	FUND. BIODIVER	1835073	Unknown	Aegilops geniculata Roth	Roth	ESP	Spain	La Solana, sierra de Alhambra, arro		CR	38.1	-2.1	
	FUND. BIODIVER	1891041	Unknown	Aegilops geniculata Roth	Roth	ESP	Spain	Formentera		PM	38.1	1.1	
00:00.0	UPS	V-200860	Specimen	Aegilops geniculata		Spain	Spain			Baleares			
00:00.0	BDBCV	593594	Observation	Aegilops geniculata		ESP	Spain		Alcoy	Alicant	38.684	-0.545	
00:00.0	BDBCV	594036	Observation	Aegilops geniculata		ESP	Spain		Alcoy	Alicant	38.665	-0.535	
	FUND. BIODIVER	1085537	Unknown	Aegilops geniculata		ESP	Spain	Ajalvir		M	40.1	-3.1	
	FUND. BIODIVER	1909855	Unknown	Aegilops geniculata Roth	Roth	ESP	Spain	Artajona, hacia Larraga, barranco D		Na	42.1	-1.1	330
00:00.0	FUND. BIODIVER	1946390	Unknown	Aegilops geniculata Roth	Roth	ESP	Spain	Moral de Calatrava, sierra de Moral		CR	37.1	-3.1	
00:00.0	REDIAM-CMA	32663	Observation	Aegilops geniculata		ESP	Spain		Chiclana de Segu	J	38.3858	-2.91595	644
00:00.0	REDIAM-CMA	385404	Observation	Aegilops geniculata		ESP	Spain		Estepona	Ma	36.4257	-5.16378	80
	RUS001	VIR100602180	Specimen	Aegilops ovata L.		ESP	Spain						
00:00.0	MA	772821-1	Specimen	Aegilops geniculata Roth.	Roth.	ES	Spain	Palma del RÃo; arroyo Mahoma en		Co			
00:00.0	FCO	4046-1	Specimen	Aegilops ovata L.	L.	ES	Spain	Morata de TajuÃ±a		M			
00:00.0	REDIAM-CMA	8329	Observation	Aegilops geniculata		ESP	Spain		Montellano	Se	36.9868	-5.55283	330
00:00.0	REDIAM-CMA	15252	Observation	Aegilops geniculata		ESP	Spain		Almonaster la Re	H	37.8576	-6.86489	495
00:00.0	REDIAM-CMA	395695	Observation	Aegilops geniculata		ESP	Spain		Luque	Co	37.5221	-4.25826	979
00:00.0	REDIAM-CMA	418295	Observation	Aegilops geniculata		ESP	Spain		LÃ¡char	Gr	37.189	-3.84431	550
	SIVIM	R-P10342:Aegi	Observation	Aegilops geniculata Roth	Roth	ES	Spain	Cortijo de OjÃ©n , Los Barrios			36.11	-5.66	0
	SIVIM	R-P10586:Aegi	Observation	Aegilops geniculata Roth	Roth	ES	Spain	Los Gallos , AG			36.38	-5.78	0
	SIVIM	R-P11719:Aegi	Observation	Aegilops geniculata Roth	Roth	ES	Spain	Quintanilla de Trigueros			41.8	-4.68	0
	SIVIM	S-P01264:Aegi	Observation	Aegilops geniculata Roth	Roth	ES	Spain	les Borges Blanques, ermita de Sant Salva			41.44	0.84	350
	SIVIM	S-P02637:Aegi	Observation	Aegilops geniculata Roth	Roth	ES	Spain	Riu Fred (Ã€ger)			41.97	0.7	0
	SIVIM	S-P04163:Aegi	Observation	Aegilops geniculata Roth	Roth	ES	Spain	voltants de Sant Jordi de Muller			41.71	0.83	260
00:00.0	BDBCV	599124	Observation	Aegilops geniculata		ESP	Spain		Alcoy	Alicant	38.656	-0.5	
	FUND. BIODIVER	1370922	Unknown	Aegilops geniculata Roth	Roth	ESP	Spain	Guadalix de la Sierra		M	40.1	-3.1	
	FUND. BIODIVER	1463961	Unknown	Aegilops geniculata Roth	Roth	ESP	Spain	Pla d'Albelda		L	41.1	0.1	440

	FUND. BIODIVER	92446	Unknown	Aegilops geniculata Roth	Roth	ESP	Spain	FuentidueÃ±a (El Rebollo)		Sg	41.1	-3.1	
00:00.0	BDBCV	590638	Observation	Aegilops geniculata		ESP	Spain		Alcoy	Alicant	38.693	-0.58	
	FUND. BIODIVER	1035355	Unknown	Aegilops ovata		ESP	Spain	Sierra de Aguas, Arroyo de los Hue		Ma	36.1	-4.1	
00:00.0	FUND. BIODIVER	1835077	Unknown	Aegilops geniculata Roth	Roth	ESP	Spain	Piedrabuena, volcan de Piedrabuen		CR	38.1	-3.1	600
	SIVIM	P-P08743:Aegi	Observation	Aegilops geniculata Roth	Roth	ES	Spain	Mola de CatÃ-; Tortosa			40.79	0.27	1160
	SIVIM	P-P08886:Aegi	Observation	Aegilops geniculata Roth	Roth	ES	Spain	Barranc de la Carrasca; la SÃ©nia			40.7	0.15	1050
	SIVIM	P-P09010:Aegi	Observation	Aegilops geniculata Roth	Roth	ES	Spain	Mola de CatÃ-; Tortosa			40.79	0.27	1300
	SIVIM	P-P11094:Aegi	Observation	Aegilops geniculata Roth	Roth	ES	Spain	Puig de la Nau			40.44	0.4	0
	SIVIM	Q-P03105:Aegi	Observation	Aegilops geniculata Roth	Roth	ES	Spain	La Llacuna, carrer assolellat, poc fressat (A			41.45	1.44	0
	SIVIM	Q-P05491:Aegi	Observation	Aegilops geniculata Roth	Roth	ES	Spain	Peralada, camÃ- entre Sant Joan Sesclose			42.27	3	0
	SIVIM	Q-P07459:Aegi	Observation	Aegilops geniculata Roth	Roth	ES	Spain	Zafra de ZÃ¡ncara			39.83	-2.64	0
	SIVIM	R-P03683:Aegi	Observation	Aegilops geniculata Roth	Roth	ES	Spain	Carretera de Arguedas a Tudela			42.08	-1.67	0
	SIVIM	R-P06819:Aegi	Observation	Aegilops geniculata Roth	Roth	ES	Spain	Villa de Ves, Casa Sandunga, Albacete			39.19	-1.26	0
	SIVIM	R-P08601:Aegi	Observation	Aegilops geniculata Roth	Roth	ES	Spain	CaparidÃ¡n			36.86	-3	1600
00:00.0	REDIAM-CMA	147110	Observation	Aegilops geniculata		ESP	Spain		CardeÃ±a	Co	38.3047	-4.22874	490
00:00.0	REDIAM-CMA	153375	Observation	Aegilops geniculata		ESP	Spain		CardeÃ±a	Co	38.1932	-4.25801	695
00:00.0	REDIAM-CMA	167351	Observation	Aegilops geniculata		ESP	Spain		Villanueva del Re	Co	38.2108	-5.17653	597
00:00.0	REDIAM-CMA	176733	Observation	Aegilops geniculata		ESP	Spain		QuÃ©ntar	Gr	37.2334	-3.42024	1296
00:00.0	REDIAM-CMA	384420	Observation	Aegilops geniculata		ESP	Spain		El Burgo	Ma	36.7948	-4.99281	1000
00:00.0	REDIAM-CMA	385748	Observation	Aegilops geniculata		ESP	Spain		Villanueva de San	Se	37.0625	-5.18991	401
00:00.0	REDIAM-CMA	388689	Observation	Aegilops geniculata		ESP	Spain		Campillos	Ma	37.0196	-4.8872	499
	REDIAM-CMA	394992	Observation	Aegilops geniculata		ESP	Spain		San JosÃ© del Va	Ca	36.6307	-5.66115	100
	SIVIM	T-P06448:Aegi	Observation	Aegilops geniculata Roth	Roth	ES	Spain	El Oro			39.28	-0.91	0
	SIVIM	T-P07221:Aegi	Observation	Aegilops geniculata Roth	Roth	ES	Spain	Tamarite de Litera			41.79	0.35	310
	SIVIM	T-P09226:Aegi	Observation	Aegilops geniculata Roth	Roth	ES	Spain	Reinoso de Cerrato			41.9	-4.44	0
	SIVIM	T-P10277:Aegi	Observation	Aegilops geniculata Roth	Roth	ES	Spain	UrbanizaciÃ³n La Pandera (Los Villares)			37.58	-3.9	1240
	SIVIM	T-P11413:Aegi	Observation	Aegilops geniculata Roth	Roth	ES	Spain	CaÃ±ete a Boniches			39.92	-1.71	1060
	SIVIM	T-P13208:Aegi	Observation	Aegilops geniculata Roth	Roth	ES	Spain	TreviÃ±o, Arrieta			42.72	-2.75	0
	SIVIM	P-P02995:Aegi	Observation	Aegilops geniculata Roth	Roth	ES	Spain	tossal de la vall de Gebut (Aitona)			41.43	0.36	155
	SIVIM	P-P08862:Aegi	Observation	Aegilops geniculata Roth	Roth	ES	Spain	Matarredona; Sant Carles de la RÃ pita			40.62	0.51	0
	SIVIM	P-P08892:Aegi	Observation	Aegilops geniculata Roth	Roth	ES	Spain	Barranc de la Canaleta; Horta de Sant Joa			40.88	0.26	0
	SIVIM	P-P09076:Aegi	Observation	Aegilops geniculata Roth	Roth	ES	Spain	PaÃ¼ls, rodalies			40.89	0.38	310
	SIVIM	P-P11104:Aegi	Observation	Aegilops geniculata Roth	Roth	ES	Spain	Riu SÃ©nia, baix les casetes del PantÃ			40.61	0.16	0
	SIVIM	Q-P00144:Aegi	Observation	Aegilops geniculata Roth	Roth	ES	Spain	Montsant d'Albarca			41.25	0.73	0
00:00.0	LEB	4787-1	Specimen	Aegilops geniculata Roth	Roth	ES	Spain	Pedrosa PrÃ-ncipe		Bu			1
	DEU146	AE 865	Specimen	Aegilops geniculata Roth		ESP	Spain	Spain: Prov. Granada, Moreda,ca. 40 km NNE of Granada					
00:00.0	HSS	5682	Specimen	Aegilops geniculata Roth	Roth	ES	Spain	Olivenza, Sierra de Alor		Ba	38.6982	-7.10294	
00:00.0	SALA	94173-1	Specimen	Aegilops geniculata Roth	Roth	ES	Spain	_; Garraf		B	41.19	1.81	
00:00.0	BDBCV	599662	Observation	Aegilops geniculata		ESP	Spain	Parc Natural de la	Alcoy	Alicant	38.727	-0.486	
	FUND. BIODIVER	1370926	Unknown	Aegilops geniculata Roth	Roth	ESP	Spain	El Molar		M	40.1	-3.1	
	FUND. BIODIVER	1463963	Unknown	Aegilops geniculata Roth	Roth	ESP	Spain	MontferrÃºs de Camporrells		L	41.1	0.1	680

	FUND. BIODIVER	960553	Unknown	Aegilops ovata		ESP	Spain	Jaca		Hu	42.1	-0.1	
00:00.0	REDIAM-CMA	129142	Observation	Aegilops geniculata		ESP	Spain		Constantina	Se	37.921	-5.71627	430
00:00.0	MA	599703-1	Specimen	Aegilops ovata Roth vel aff.	Roth vel aff.	ES	Spain	La Gomera. AlajerÃ³. Carretera Play		Tf			
00:00.0	SEV	71063-1	Specimen	Aegilops geniculata Roth	Roth	ES	Spain	Tarifa. Margen Norte del Embalse d		Ca			100
00:00.0	SEV	89992-1	Specimen	Aegilops ovata L.	L.	ES	Spain	Castellar de la Frontera. Pantano de		Ca			1
00:00.0	MGC	43911-1	Unknown	Aegilops geniculata Roth	Roth	ES	Spain	BenahavÃ-s; Sierra Palmitera. Carre		Ma	36.6	-5.063	1100
	SIVIM	T-P06456:Aegi	Observation	Aegilops geniculata Roth	Roth	ES	Spain	Sinarcas			39.73	-1.24	0
	SIVIM	T-P09142:Aegi	Observation	Aegilops geniculata Roth	Roth	ES	Spain	Iscar			41.45	-4.55	0
	SIVIM	T-P09228:Aegi	Observation	Aegilops geniculata Roth	Roth	ES	Spain	Tudela de Duero			41.53	-4.67	0
	SIVIM	T-P10394:Aegi	Observation	Aegilops geniculata Roth	Roth	ES	Spain	Camino del Pto. de Quejigales, pasado Co			36.66	-5.12	1120
	SIVIM	T-P11862:Aegi	Observation	Aegilops geniculata Roth	Roth	ES	Spain	El Portillo			42.51	-5.55	0
	SIVIM	T-P13210:Aegi	Observation	Aegilops geniculata Roth	Roth	ES	Spain	UjuÃ©, hacia Gallipienzo			43.25	-1.52	0
00:00.0	BDBCV	334644	Observation	Aegilops geniculata	_	ES	Spain		La Nucia	A	38.59	-0.07	
00:00.0	BC	70787	Specimen	Aegilops ovata L.	L.	ES	Spain	Madrid; El Pardo		M	40.51	-3.77	
00:00.0	COFC	25777-1	Specimen	Aegilops geniculata Roth	Roth	ES	Spain	Zufre; Sierra de Aracena		H	37	-6	1
	BDBCV-General	40635	Observation	Aegilops geniculata		ESPA	Spain	Vall de Almonacid	El Alto Palancia	Castel	39.9472	-0.48344	
00:00.0	HUAL	12330-1	Specimen	Aegilops geniculata Roth	Roth	ES	Spain	SÂª de GÃ¡dor, cara S		Al			2000
00:00.0	MGC	27651-1	Unknown	Aegilops geniculata Roth	Roth	ES	Spain	MÃ¡laga; Montes de MÃ¡laga. Carre		Ma	36.773	-4.339	1
00:00.0	MGC	33083-1	Unknown	Aegilops geniculata Roth	Roth	ES	Spain	Benaocaz; _		Ca	0	0	1
00:00.0	BDBCV	120	Observation	Aegilops geniculata		ESP	Spain	Parque Natural de Penyagolosa		Cs	40.23	-0.41	
00:00.0	MGC	16226-1	Unknown	Aegilops geniculata Roth	Roth	ES	Spain	MÃ¡laga; Churriana. Sierra de Torre		Ma	36.63	-4.51	1
00:00.0	MGC	59967-1	Unknown	Aegilops geniculata Roth	Roth	ES	Spain	Canillas de Aceituno; P. N. de las S		Ma	36.893	-4.083	940
	RUS001	VIR100602141	Specimen	Aegilops ovata L.		ESP	Spain						
	RUS001	VIR100602119	Specimen	Aegilops ovata L.		ESP	Spain						
	RUS001	VIR100602098	Specimen	Aegilops ovata L.		ESP	Spain						
00:00.0	FUND. BIODIVER	1946387	Unknown	Aegilops geniculata Roth	Roth	ESP	Spain	Herencia, cerro Navajo, ladera SE		CR	39.1	-3.1	660
00:00.0	BDBCV	589424	Observation	Aegilops geniculata		ESP	Spain		Alcoy	Alicant	38.72	-0.544	
00:00.0	COFC	46884-1	Specimen	Aegilops geniculata Roth	Roth	ES	Spain	Hornachuelos; embalse del municip		Co			1
	FUND. BIODIVER	1715686	Unknown	Aegilops geniculata Roth	Roth	ESP	Spain	Aiguamolls de l'Empurda		Ge	41.1	2.1	
	FUND. BIODIVER	1762688	Unknown	Aegilops geniculata		ESP	Spain	Volcan de Yezosa		CR	38.1	-3.1	
	FUND. BIODIVER	1811864	Unknown	Aegilops geniculata		ESP	Spain	Tudela, carretera a Ejea de los Cab		Na	41.1	-1.1	
00:00.0	Marimurtra	4473-1	Specimen	Aegilops geniculata Roth.	Roth.	ES	Spain	Son Rapinya		Mll			
00:00.0	REDIAM-CMA	21193	Observation	Aegilops geniculata		ESP	Spain		CoÃ-n	Ma	36.6544	-4.77467	200
00:00.0	REDIAM-CMA	410467	Observation	Aegilops geniculata		ESP	Spain		Siles	J	38.3684	-2.49878	1481
00:00.0	REDIAM-CMA	416969	Observation	Aegilops geniculata		ESP	Spain		Tabernas	Al	37.112	-2.34252	578
00:00.0	REDIAM-CMA	293535	Observation	Aegilops geniculata		ESP	Spain		Almonaster la Re	H	37.7966	-6.86709	350
00:00.0	REDIAM-CMA	338316	Observation	Aegilops geniculata		ESP	Spain		Antequera	Ma	36.9754	-4.73263	417
00:00.0	REDIAM-CMA	381734	Observation	Aegilops geniculata		ESP	Spain		Ardales	Ma	36.9013	-4.91748	731
00:00.0	GDA	GDA12219-1	Specimen	Aegilops geniculata Roth.	Roth.	ES	Spain	Granada, SÂª Nevada, CÃ¡Ã±ar, ba		GR			1100
	SIVIM	Q-P03053:Aegi	Observation	Aegilops geniculata Roth	Roth	ES	Spain	Montclar, vora la carretera (BerguedÃ)			41.99	1.67	0
	SIVIM	Q-P04604:Aegi	Observation	Aegilops geniculata Roth	Roth	ES	Spain	VentallÃ³, entre el Puig d'EstraguÃ©s i Vila			42.09	3	0

	SIVIM	Q-P07447:Aegi	Observation	Aegilops geniculata Roth	Roth	ES	Spain	Honrubia			39.56	-2.3	0
	SIVIM	R-P03514:Aegi	Observation	Aegilops geniculata Roth	Roth	ES	Spain	Agost			38.37	-0.71	0
	SIVIM	R-P06816:Aegi	Observation	Aegilops geniculata Roth	Roth	ES	Spain	AlcalÃ¡ del JÃºcar, rambla de San Lorenzo			39.1	-1.49	0
	SIVIM	R-P08598:Aegi	Observation	Aegilops geniculata Roth	Roth	ES	Spain	Ctjo. de la Cruz			36.77	-2.77	1400
00:00.0	W	42291	Unknown	Aegilops ovata L.		ESP	Spain	Plantae Gallicae. Gard: environs d'Ales, a Bagard.					
	SIVIM	U-P08807:Aegi	Observation	Aegilops geniculata Roth	Roth	ES	Spain	El Salvador (Ulea)			38.11	-1.4	220
	MA	584690-1	Specimen	Aegilops geniculata Roth.	Roth.	ES	Spain	Madrid		M			
00:00.0	JBS	463-1	Specimen	Aegilops ovata L. subsp. triaristat	(Willd.) Rouy	ES	Spain	Escorca; Clot d'Albarca		Mll	39.84	2.88	1
00:00.0	GDA	GDA15905-1-2	Specimen	Aegilops geniculata Roth.	Roth.	ES	Spain	Granada, SÂª Pelada, barranco de		GR			980
00:00.0	GDA	GDA23046-1-1	Specimen	Aegilops geniculata Roth.	Roth.	ES	Spain	Granada, Padul, cerro de los Molino		GR			750
	GDA	GDA30019-1-1	Specimen	Aegilops ovata L.	L.	ES	Spain	Valladolid, Cuesta de Maruquera.		VA			0
1879-06-1	BC	824678	Specimen	Aegilops ovata L.	L.	ES	Spain	Terrassa		B			
	IDBD-GN	42467	Observation	Aegilops geniculata Roth	Roth	ES	Spain		Esquinza	Na	42.657	-1.92152	
	IDBD-GN	42478	Observation	Aegilops geniculata Roth	Roth	ES	Spain	Antxoriz	Esteribar	Na	42.8693	-1.57503	600
	IDBD-GN	42500	Observation	Aegilops geniculata Roth	Roth	ES	Spain	Vigas	UjuÃ©	Na	42.4361	-1.4998	700
	DEU146	AE 649	Specimen	Aegilops geniculata Roth		ESP	Spain	Pyrenen, Spanien					
00:00.0	SEV	100515-1	Specimen	Aegilops geniculata Roth	Roth	ES	Spain	RÃo Guadiato. Cerro del Trigo		Co			1
00:00.0	SEV	108139-1	Specimen	Aegilops geniculata Roth	Roth	ES	Spain	Villaverde del RÃo		Se			1
00:00.0	SEV	108257-1	Specimen	Aegilops geniculata Roth	Roth	ES	Spain	Sierra Nevada. Subida al Pico Velet		Gr			1800
00:00.0	GDA	GDA15236-1	Specimen	Aegilops geniculata Roth.	Roth.	ES	Spain	Granada, carretera Ãllora-MontefrÃ		GR			700
	RUS001	VIR100602121	Specimen	Aegilops ovata L.		ESP	Spain						
	RUS001	VIR100602100	Specimen	Aegilops ovata L.		ESP	Spain						
	RUS001	VIR100602079	Specimen	Aegilops ovata L.		ESP	Spain						
	IPK	32131	Living	Aegilops geniculata Roth		ESP	Spain	Pyrenaen, Spanien					
00:00.0	SALA	8160-1	Specimen	Aegilops ovata L.	L.	ES	Spain	_; Aldealengua		Sa			
00:00.0	SALA	85593-1	Specimen	Aegilops geniculata Roth	Roth	ES	Spain	_; Castellanos de Villiquera		Sa	41.06	-5.64	
	FUND. BIODIVER	1074145	Unknown	Aegilops geniculata		ESP	Spain	Baena		Co	37.1	-4.1	
	FUND. BIODIVER	1093242	Unknown	Aegilops geniculata		ESP	Spain	Coria		Cc	39.1	-6.1	
00:00.0	FUND. BIODIVER	1946406	Unknown	Aegilops geniculata Roth	Roth	ESP	Spain	Alhambra, sierra de Alhambra, arroy		CR	38.1	-2.1	
	GDA	GDA30014-1-1	Specimen	Aegilops ovata L.	L.	ES	Spain	Granada.		GR			0
00:00.0	REDIAM-CMA	6630	Observation	Aegilops geniculata		ESP	Spain		Luque	Co	37.4939	-4.26578	899
00:00.0	REDIAM-CMA	14219	Observation	Aegilops geniculata		ESP	Spain		Torres	J	37.7784	-3.50605	923
00:00.0	REDIAM-CMA	20685	Observation	Aegilops geniculata		ESP	Spain		Cambil	J	37.7313	-3.50761	1623
00:00.0	REDIAM-CMA	416895	Observation	Aegilops geniculata		ESP	Spain		Tabernas	Al	37.0847	-2.33597	480
00:00.0	SEV	99209-1	Specimen	Aegilops geniculata Roth	Roth	ES	Spain	Constantina. El Robledo		Se			800
00:00.0	SEV	100518-1	Specimen	Aegilops geniculata Roth	Roth	ES	Spain	Luque. Faldas del Cerro del Algarro		Co			1
00:00.0	SEV	108142-1	Specimen	Aegilops geniculata Roth	Roth	ES	Spain	Palma del RÃo. Arroyo Mahoma		Co			1
00:00.0	SEV	108260-1	Specimen	Aegilops geniculata Roth	Roth	ES	Spain	Puerto de Santa MarÃa		Ca			1
	SIVIM	Q-P03048:Aegi	Observation	Aegilops geniculata Roth	Roth	ES	Spain	La Llacuna, talÃºs al costat del camÃ de la			41.45	1.44	0
	SIVIM	Q-P03814:Aegi	Observation	Aegilops geniculata Roth	Roth	ES	Spain	Cortes de Arenoso			40.17	-0.53	1440
	SIVIM	Q-P04212:Aegi	Observation	Aegilops geniculata Roth	Roth	ES	Spain	Lacort			42.41	0.2	0

	SIVIM	Q-P06906:Aegi	Observation	Aegilops geniculata Roth	Roth	ES	Spain	SÂª del Cid, La Almadraba, Petrer,			38.46	-0.82	0
	SIVIM	R-P02683:Aegi	Observation	Aegilops geniculata Roth	Roth	ES	Spain	pendent entre Utxesa i la central de FECSA			41.43	0.48	150
	SIVIM	R-P06813:Aegi	Observation	Aegilops geniculata Roth	Roth	ES	Spain	Albacete, Los Yesares, Albacete			39.11	-1.84	0
	SIVIM	R-P06814:Aegi	Observation	Aegilops geniculata Roth	Roth	ES	Spain	Albacete, cantera de Los Yesares, Albacet			39.11	-1.84	0
	SIVIM	R-P08592:Aegi	Observation	Aegilops geniculata Roth	Roth	ES	Spain	Punta del Sabinar			36.68	-2.77	0
00:00.0	BDBCV	115	Observation	Aegilops geniculata		ESP	Spain	Parque Natural de Penyagolosa		Cs			
	FUND. BIODIVER	1531939	Unknown	Aegilops ovata L.	L.	ESP	Spain	Mataporquera		S	42.1	-3.1	
	FUND. BIODIVER	1587949	Unknown	Aegilops ovata		ESP	Spain	Tierra de Campos		P			
	FUND. BIODIVER	993576	Unknown	Aegilops geniculata		ESP	Spain	TamajÃ³n		Gu	40.1	-3.1	
	FUND. BIODIVER	99839	Unknown	Aegilops geniculata Roth	Roth	ESP	Spain	TÃ¡bara		Za	41.1	-5.1	
00:00.0	SALA	24374-1	Specimen	Aegilops geniculata Roth	Roth	ES	Spain	_; Tiedra		Va			
00:00.0	GDAC	GDAC42935-1	Specimen	Aegilops geniculata Roth.	Roth.	ES	Spain	AlmerÃa, LÃºcar, SÂª de LÃºcar.		AL			0
00:00.0	MA	650528-1	Specimen	Aegilops geniculata Roth.	Roth.	ES	Spain	Embalse del Campillo		M			
00:00.0	REDIAM-CMA	189097	Observation	Aegilops geniculata		ESP	Spain		Tabernas	Al	37.0823	-2.34895	486
00:00.0	REDIAM-CMA	204002	Observation	Aegilops geniculata		ESP	Spain		Segura de la Sier	J	38.2856	-2.67313	787
00:00.0	GDA	GDA7741-1-2	Specimen	Aegilops geniculata Roth.	Roth.	ES	Spain	Madrid, La Moncloa.		M			0
	SIVIM	T-P13701:Aegi	Observation	Aegilops geniculata Roth	Roth	ES	Spain	MasÃa de Porcar, Cantavieja			40.44	-0.4	1400
	SIVIM	T-P16907:Aegi	Observation	Aegilops geniculata Roth	Roth	ES	Spain	Loeches			40.37	-3.47	0
	SIVIM	T-P19965:Aegi	Observation	Aegilops geniculata Roth	Roth	ES	Spain	Dehesa de los Caballos (Plasencia)			39.98	-6.18	0
	SIVIM	T-P24600:Aegi	Observation	Aegilops geniculata Roth	Roth	ES	Spain	Calizas entre BayÃ¡rcal y Paterna			36.95	-3	1300
	SIVIM	T-P27297:Aegi	Observation	Aegilops geniculata Roth	Roth	ES	Spain	Loma prÃ³xima a la Zaida (Used)			41	-1.69	1050
	SIVIM	T-P28636:Aegi	Observation	Aegilops geniculata Roth	Roth	ES	Spain	El Ejido, Saucedilla			39.8	-5.68	280
	DEU146	AE 777	Specimen	Aegilops geniculata Roth subsp. gibberosa (Zh		ESP	Spain	W.-Pyrenen, b. Jaca					810
	CZE122	01C2109058	Specimen	Aegilops geniculata subsp. gibberosa (ZHUK.)		ESP	Spain	Formentor, Majorque, Baleares					
00:00.0	SEV	108146-1	Specimen	Aegilops geniculata Roth	Roth	ES	Spain	RÃo Guadalmellato. Finca Ribera E		Co			1
00:00.0	SEV	108263-1	Specimen	Aegilops geniculata Roth	Roth	ES	Spain	Above Los Barrios		Ca			300
	RUS001	VIR100602115	Specimen	Aegilops ovata L.		ESP	Spain						
	RUS001	VIR100602094	Specimen	Aegilops ovata L.		ESP	Spain						
	RUS001	VIR100602073	Specimen	Aegilops ovata L.		ESP	Spain						
	BDBCV-General	274782	Observation	Aegilops geniculata		ESPA	Spain	Chella	La Canal de Nava	Valenc	39.0517	-0.74664	
	FUND. BIODIVER	1093227	Unknown	Aegilops geniculata		ESP	Spain	Dehesa de los Caballos, Plasencia		Cc	39.1	-5.1	
	FUND. BIODIVER	1918074	Unknown	Aegilops geniculata		ESP	Spain	Artajona, hacia Larraga, barranco D		Na	42.1	-1.1	330
00:00.0	FUND. BIODIVER	1946391	Unknown	Aegilops geniculata Roth	Roth	ESP	Spain	Malagon-Los Quiles, olivos		CR	39.1	-3.1	
00:00.0	REDIAM-CMA	8458	Observation	Aegilops geniculata		ESP	Spain		Olvera	Ca	36.9272	-5.36345	390
00:00.0	REDIAM-CMA	15760	Observation	Aegilops geniculata		ESP	Spain		Torres	J	37.7537	-3.4955	1206
00:00.0	REDIAM-CMA	410667	Observation	Aegilops geniculata		ESP	Spain		Villarrodrigo	J	38.4797	-2.64294	900
00:00.0	REDIAM-CMA	415471	Observation	Aegilops geniculata		ESP	Spain		Cortes de Baza	Gr	37.7479	-2.85508	931
00:00.0	REDIAM-CMA	418329	Observation	Aegilops geniculata		ESP	Spain		Jayena	Gr	36.9899	-3.79275	1300
	CZE122	01C2109083	Specimen	Aegilops geniculata Roth		ESP	Spain	Pyrenaeen, Spanien					
00:00.0	ABH	42119-1	Specimen	Aegilops geniculata Roth	Roth	ES	Spain	Campello; Cala Baeza		A	38.47	-0.33	
00:00.0	SEV	30534-1	Specimen	Aegilops ovata L.	L.	ES	Spain	Entre MorÃ³n y Pruna, cercanÃas d		Se			1

00:00.0	SEV	36034-1	Specimen	Aegilops ovata L.	L.	ES	Spain	Entre Arcos de la Frontera y El Bos		Ca			1
	SIVIM	Q-P03107:Aegi	Observation	Aegilops geniculata Roth	Roth	ES	Spain	La Llacuna, carrer una mica ombrejat (Ano			41.45	1.44	0
	SIVIM	Q-P05493:Aegi	Observation	Aegilops geniculata Roth	Roth	ES	Spain	Peralada, regall de la pista que mena de P			42.27	3	0
	SIVIM	Q-P07460:Aegi	Observation	Aegilops geniculata Roth	Roth	ES	Spain	Torrebuceit			39.92	-2.53	0
	SIVIM	R-P03684:Aegi	Observation	Aegilops geniculata Roth	Roth	ES	Spain	Valtierra			42.17	-1.66	0
	SIVIM	R-P06910:Aegi	Observation	Aegilops geniculata Roth	Roth	ES	Spain	Fuentealbilla, hacia el JÃºcar 1 Km, Albace			39.2	-1.61	0
	SIVIM	R-P08602:Aegi	Observation	Aegilops geniculata Roth	Roth	ES	Spain	BalsÃ³n de las Hoyuelas			36.86	-2.88	1850
1876-06-2	BC	SBB-1184	Specimen	Aegilops geniculata Roth	Roth	ES	Spain	La Alcarria (Guadalajara)		GU			
	GDA	GDA30012-1	Specimen	Aegilops ovata L.	L.	ES	Spain	Madrid, Arganda.		M			0
	BDBCV-General	74115	Observation	Aegilops geniculata		ESPA	Spain	Pina de Montalgra	El Alto Palancia	Castel	40.0397	-0.59724	
00:00.0	COFC	11967-1	Specimen	Aegilops geniculata Roth	Roth	ES	Spain	Priego de CÃ³rdoba; pico Leones		Co	37	-4	1
00:00.0	COFC	11993-1	Specimen	Aegilops geniculata Roth	Roth	ES	Spain	Priego de CÃƒÂ³rdoba; cerro los Ye		Co	37	-5	1
00:00.0	COFC	16300-1	Specimen	Aegilops geniculata Roth	Roth	ES	Spain	Hinojosa del Duque; estaciÃƒÂ³n de		Co	38	-5	1
00:00.0	COFC	1978-1	Specimen	Aegilops ovata L.	L.	ES	Spain	Santa Maria de Trasierra; rÃƒÂ-o G		Co			1
00:00.0	ABH	679-1	Specimen	Aegilops geniculata Roth	Roth	ES	Spain	NÃ-jar; CÂºGata,MÃ³nsul,Ensen.Me		Al	36.73	-2.16	
00:00.0	ABH	8651-1	Specimen	Aegilops geniculata Roth	Roth	ES	Spain	Crevillente; Canal del Trasvase		A	38.23	-0.84	
00:00.0	BDBCV	105	Observation	Aegilops geniculata		ESP	Spain	Parque Natural de Penyagolosa		Cs	40.17	-0.37	
00:00.0	SEV	5986-1	Specimen	Aegilops ovata L.	L.	ES	Spain	Las Cuerlas, junto a la Laguna de G		Z			1
	IDBD-GN	42457	Observation	Aegilops geniculata Roth	Roth	ES	Spain		Falces	Na	42.3946	-1.79238	
	IDBD-GN	42470	Observation	Aegilops geniculata Roth	Roth	ES	Spain	Selva	Petilla de AragÃ³r	Na	42.4486	-1.12254	950
	IDBD-GN	42479	Observation	Aegilops geniculata Roth	Roth	ES	Spain	Osteriz	Esteribar	Na	42.9134	-1.50051	700
	IDBD-GN	42491	Observation	Aegilops geniculata Roth	Roth	ES	Spain	RÃ-o Arga	Puente la Reina	Na	42.6741	-1.82359	340
00:00.0	HUAL	6058-1	Specimen	Aegilops geniculata Roth	Roth	ES	Spain	SÂª de GÃ¡dor, Fuente Nueva		Al	36.825	-2.849	
	BDBCV-General	77226	Observation	Aegilops geniculata		ESPA	Spain	El Toro	El Alto Palancia	Castel	39.9542	-0.83434	
	BDBCV-General	81794	Observation	Aegilops geniculata		ESPA	Spain				39.8596	-0.60353	
00:00.0	COFC	11970-1	Specimen	Aegilops geniculata Roth	Roth	ES	Spain	Cabra; Casilla de Buenavista		Co	37	-4	1
	ESP004	NC027448	Specimen	Aegilops geniculata Roth		ESP	Spain	Alcolea del Pinar, province of Guadalajara			41.0333	-2.45	1205
	ESP004	NC022312	Specimen	Aegilops geniculata Roth		ESP	Spain	Olvera, province of Cadiz			36.9333	-5.26667	623
00:00.0	ABH	48967-1	Specimen	Aegilops geniculata Roth	Roth	ES	Spain	Salinas; ctra. a Villena, bancales pa		A	38.52	-0.91	
00:00.0	MUB	102377-1	Specimen	Aegilops geniculata Roth	Roth	ES	Spain	Mula; Sierra EspuÃ±a		Mu	37.917	-1.584	1
00:00.0	BDBCV	589311	Observation	Aegilops geniculata		ESP	Spain		Alcoy	Alicant	38.683	-0.534	
00:00.0	COFC	46883-1	Specimen	Aegilops geniculata Roth	Roth	ES	Spain	ctra hacia Sevilla, Km 53-54; finca 'l		Co			1
	FUND. BIODIVER	1018236	Unknown	Aegilops ovata L.	L.	ESP	Spain	La Gabia		Gr	36.1	-3.1	
	FUND. BIODIVER	1715685	Unknown	Aegilops geniculata		ESP	Spain	Peraleda, regall de la pista que mer		Ge	42.1	2.1	
	FUND. BIODIVER	1762687	Unknown	Aegilops geniculata		ESP	Spain	Morron de Villamayor		CR	38.1	-3.1	
	FUND. BIODIVER	1811863	Unknown	Aegilops geniculata		ESP	Spain	Arguedas, carretera a Tudela		Na	41.1	-1.1	
00:00.0	FUND. BIODIVER	1336387	Unknown	Aegilops geniculata Roth	Roth	ESP	Spain	Montorio		Bu	42.1	-3.1	
	FUND. BIODIVER	1340280	Unknown	Aegilops geniculata Roth	Roth	ESP	Spain	FrÃ-as		Bu	42.1	-3.1	600
	FUND. BIODIVER	1366008	Unknown	Aegilops geniculata Roth	Roth	ESP	Spain	Salvatierra, Barranco de la Garona		Z	42.1	-0.1	600
	FUND. BIODIVER	70862	Unknown	Aegilops ovata L.	L.	ESP	Spain	Moriscos, camino hacia Gomecello		Sa	40.1	-5.1	
	FUND. BIODIVER	78791	Unknown	Aegilops geniculata Roth.	Roth.	ESP	Spain	Olmos de Esgueva		Va	41.1	-4.1	

	FUND. BIODIVER	847027	Unknown	Aegilops ovata L.	L.	ESP	Spain	Sierra de la Murta		V	38.1	-0.1	
00:00.0	BDBCV	118	Observation	Aegilops geniculata		ESP	Spain	Parque Natural de Penyagolosa		Cs	40.21	-0.41	
00:00.0	REDIAM-CMA	291675	Observation	Aegilops geniculata		ESP	Spain		Cortes de Baza	Gr	37.751	-2.84783	901
00:00.0	REDIAM-CMA	324067	Observation	Aegilops geniculata		ESP	Spain		AlmadÃ©n de la F	Se	37.8523	-6.00558	278
00:00.0	REDIAM-CMA	337740	Observation	Aegilops geniculata		ESP	Spain		Teba	Ma	36.985	-4.85772	573
00:00.0	REDIAM-CMA	381715	Observation	Aegilops geniculata		ESP	Spain		Coripe	Se	37.002	-5.38201	389
00:00.0	REDIAM-CMA	382748	Observation	Aegilops geniculata		ESP	Spain		Torres	J	37.7784	-3.50617	923
00:00.0	GDA	GDA30017-1-2	Specimen	Aegilops geniculata Roth.	Roth.	ES	Spain	Granada, Dehesa de Montejo.		GR			0
00:00.0	ABH	23285-1	Specimen	Aegilops geniculata Roth	Roth	ES	Spain	Villena; Cabezo Redondo		A	38.64	-0.9	
00:00.0	ABH	34284-1	Specimen	Aegilops geniculata Roth	Roth	ES	Spain	Villena; La Serrata, prox. Collado de		A	38.58	-0.93	
00:00.0	REDIAM-CMA	88573	Observation	Aegilops geniculata		ESP	Spain		Campillos	Ma	37.0731	-4.96988	599
00:00.0	REDIAM-CMA	94937	Observation	Aegilops geniculata		ESP	Spain		Almonte	H	37.2735	-6.54163	66
00:00.0	REDIAM-CMA	126480	Observation	Aegilops geniculata		ESP	Spain		Castillo de Locub	J	37.5166	-3.91959	800
	SIVIM	U-P08629:Aegi	Observation	Aegilops geniculata Roth	Roth	ES	Spain	Fuente del Pino (Jumilla)			38.47	-1.39	0
	SIVIM	S-P04169:Aegi	Observation	Aegilops geniculata Roth	Roth	ES	Spain	Santa CecÃlia de VoltregÃ , entre Sorribes			41.9	2.15	0
	SIVIM	S-P09959:Aegi	Observation	Aegilops geniculata Roth	Roth	ES	Spain	prop de la font de l'Arbre, serra Aitana (Co			38.63	-0.35	1200
	SIVIM	S-P14064:Aegi	Observation	Aegilops geniculata Roth	Roth	ES	Spain	Serrinha			38.39	-8.54	0
	SIVIM	T-P01868:Aegi	Observation	Aegilops geniculata Roth	Roth	ES	Spain	Eivissa: damunt la cala d'Aubarca			39.01	1.26	0
	SIVIM	T-P03854:Aegi	Observation	Aegilops geniculata Roth	Roth	ES	Spain	1 km al S de ValdejeÃ±a			41.72	-2.27	1030
	SIVIM	T-P06389:Aegi	Observation	Aegilops geniculata Roth	Roth	ES	Spain	Ayora			39.01	-1.03	0
00:00.0	GDA	GDA18052-1-2	Specimen	Aegilops geniculata Roth	Roth	ES	Spain	Granada, SÂª Nevada, Monachil, ba		GR			1600
00:00.0	GDA	GDA22403-1-1	Specimen	Aegilops geniculata Roth.	Roth.	ES	Spain	Granada, Cogollos Vega, proximida		GR			1100
00:00.0	BDBCV	596578	Observation	Aegilops geniculata		ESP	Spain		Ibi	Alicant	38.648	-0.57	
00:00.0	BDBCV	596933	Observation	Aegilops geniculata		ESP	Spain		Alcoy	Alicant	38.675	-0.546	
	FUND. BIODIVER	1141315	Unknown	Aegilops ovata subsp. ovata L.	L.	ESP	Spain	Vegas del Condado		Le	42.1	-5.1	
	FUND. BIODIVER	36859	Unknown	Aegilops geniculata Roth	Roth	ESP	Spain	Corres		Vi	42.1	-2.1	700
	FUND. BIODIVER	57500	Unknown	Aegilops geniculata Roth	Roth	ESP	Spain	Matilla de los CaÃ±os del rÃo		Sa	40.1	-5.1	
	IDBD-GN	42465	Observation	Aegilops geniculata Roth	Roth	ES	Spain		Marcilla	Na	42.3309	-1.73291	
	IDBD-GN	42476	Observation	Aegilops geniculata Roth	Roth	ES	Spain	Foz de Benasa	Navascues	Na	42.7098	-1.12689	690
	IDBD-GN	42498	Observation	Aegilops geniculata Roth	Roth	ES	Spain	Soila	Korres	Vi	42.7057	-2.43351	800
00:00.0	GDA	GDA10274-1-1	Specimen	Aegilops geniculata Roth. var. pu	Roth.	ES	Spain	Granada, SÂª de Mecina, Pitres, fal		GR			1150
00:00.0	REDIAM-CMA	64128	Observation	Aegilops geniculata		ESP	Spain		Salar	Gr	37.0943	-4.06345	889
	IDBD-GN	42429	Observation	Aegilops geniculata Roth	Roth	ES	Spain	Las PeÃ±as-Oqui	Lumbier	Na	42.6619	-1.23204	5250
	IDBD-GN	42441	Observation	Aegilops geniculata Roth	Roth	ES	Spain	Chucho Alto	UjuÃ©	Na	42.5445	-1.52156	650
	SIVIM	R-P10341:Aegi	Observation	Aegilops geniculata Roth	Roth	ES	Spain	Casas del Corchadillo , Jerez de la Fronter			36.47	-5.67	0
	SIVIM	R-P10581:Aegi	Observation	Aegilops geniculata Roth	Roth	ES	Spain	RincÃ³n de Soriano , AG			36.29	-5.67	0
	SIVIM	R-P11718:Aegi	Observation	Aegilops geniculata Roth	Roth	ES	Spain	Quintanilla de Trigueros			41.8	-4.68	0
	SIVIM	S-P01262:Aegi	Observation	Aegilops geniculata Roth	Roth	ES	Spain	les Borges Blanques, mas del Cunco			41.44	0.84	0
	SIVIM	S-P04075:Aegi	Observation	Aegilops geniculata Roth	Roth	ES	Spain	Montan de Tost (vall de la Vansa)			42.16	1.3	1180
00:00.0	GDA	GDA30016-1	Specimen	Aegilops ovata L.	L.	ES	Spain	Granada, Golilla de Cartuja.		GR			0
	FUND. BIODIVER	1531949	Unknown	Aegilops ovata L.	L.	ESP	Spain	Mena		Bu	42.1	-3.1	

	FUND. BIODIVER	99840	Unknown	Aegilops geniculata Roth	Roth	ESP	Spain	Pozuelo de TÃ¡bara		Za	41.1	-5.1	
00:00.0	UNEX	10377-1	Observation	Aegilops geniculata Roth	_	ESP	Spain	Zafra: Rivera de Zafra. 29SQC25		Ba	38.4	-6.5	
00:00.0	BC	70805	Specimen	Triticum ovatum (L.) Gren. & God	(L.) Gren. &	ES	Spain	Sant Antoni de Portmany; in Ebuso:		PM	38.97	1.33	
	BC	140318	Specimen	Aegilops ovata L.	L.	ES	Spain	BenicÃ ssim; Regno valentino: Beni		Cs	40.03	0.01	
00:00.0	REDIAM-CMA	189207	Observation	Aegilops geniculata		ESP	Spain		Cazorla	J	37.9142	-2.99668	976
00:00.0	REDIAM-CMA	196464	Observation	Aegilops geniculata		ESP	Spain		Cazorla	J	37.8685	-2.92666	1426
00:00.0	REDIAM-CMA	220160	Observation	Aegilops geniculata		ESP	Spain		AlbuÃ±uelas	Gr	36.9279	-3.7462	1250
00:00.0	REDIAM-CMA	236029	Observation	Aegilops geniculata		ESP	Spain		Almonaster la Re	H	37.8696	-6.75319	592
	ESP004	NC050480	Specimen	Aegilops geniculata Roth		ESP	Spain	Cuenca/Villalba de la Sierra 8km N, Cuenc			40.15	-2.13333	940
	SIVIM	T-P13702:Aegi	Observation	Aegilops geniculata Roth	Roth	ES	Spain	Cantavieja			40.44	-0.52	1400
	SIVIM	T-P16908:Aegi	Observation	Aegilops geniculata Roth	Roth	ES	Spain	TamajÃ³n			40.92	-3.35	0
	SIVIM	T-P16909:Aegi	Observation	Aegilops geniculata Roth	Roth	ES	Spain	Fresneda de Allarejos			39.92	-2.41	0
	SIVIM	T-P19966:Aegi	Observation	Aegilops geniculata Roth	Roth	ES	Spain	Dehesa de los Caballos (Plasencia)			39.98	-6.18	0
	SIVIM	T-P24601:Aegi	Observation	Aegilops geniculata Roth	Roth	ES	Spain	Pr. Monterrey			37.04	-3	1250
	SIVIM	T-P27379:Aegi	Observation	Aegilops geniculata Roth	Roth	ES	Spain	Fuentealbilla			39.2	-1.61	0
	SIVIM	T-P27382:Aegi	Observation	Aegilops geniculata Roth	Roth	ES	Spain	Yebra			40.29	-3	0
	SIVIM	T-P28637:Aegi	Observation	Aegilops geniculata Roth	Roth	ES	Spain	Casatejada			39.8	-5.68	280
00:00.0	FUND. BIODIVER	1972309	Unknown	Aegilops geniculata		ESP	Spain	Valle de Escuain, Puertolas, refugio		Hu	42.1	0.9	2010
	FUND. BIODIVER	51240	Unknown	Aegilops ovata subsp. ovata L.	L.	ESP	Spain	Almenara de Tormes, tesos		Sa	40.1	-5.1	
	FUND. BIODIVER	56189	Unknown	Aegilops ovata L.	L.	ESP	Spain	Bocigas		Va	41.1	-4.1	
	FUND. BIODIVER	60942	Unknown	Aegilops ovata subsp. ovata L.	L.	ESP	Spain	San Esteban de la Sierra		Sa	40.1	-5.1	
00:00.0	GDA	GDA48984-1-1	Specimen	Aegilops geniculata Roth	Roth	ES	Spain	AlmerÃa, SÂª de los Filabres, Beni		AL			800
00:00.0	MA	718181-1	Specimen	Aegilops geniculata Roth.	Roth.	ES	Spain	Aibar, Olatz.		Na	42	-1	
	FUND. BIODIVER	1463968	Unknown	Aegilops geniculata Roth	Roth	ESP	Spain	Les Avellanes, monestir		L	41.1	0.1	580
	FUND. BIODIVER	918137	Unknown	Aegilops ovata var. ambigua Nob	Nob.	ESP	Spain	San CristÃ³fol de Montserrat		B	41.1	1.1	
	FUND. BIODIVER	96900	Unknown	Aegilops geniculata Roth.	Roth.	ESP	Spain	Fermoselle		Za			
	FUND. BIODIVER	974822	Unknown	Aegilops ovata L.	L.	ESP	Spain	Calatayud		Z	41.1	-1.1	
00:00.0	ABH	4013-1	Specimen	Aegilops geniculata Roth	Roth	ES	Spain	Confrides; SÂªAitana, prox. Font de		A	38.66	-0.29	
00:00.0	ABH	5011-1	Specimen	Aegilops geniculata Roth	Roth	ES	Spain	Orxeta; a la eixida		A	38.56	-0.27	
00:00.0	GDAC	GDAC31112-1	Specimen	Aegilops geniculata Roth.	Roth.	ES	Spain	AlmerÃa, Llanos de Tabernas, km.		AL			500
00:00.0	REDIAM-CMA	27584	Observation	Aegilops geniculata		ESP	Spain		Pegalajar	J	37.7331	-3.5323	1893
00:00.0	REDIAM-CMA	132236	Observation	Aegilops geniculata		ESP	Spain		Constantina	Se	37.9093	-5.7217	397
00:00.0	REDIAM-CMA	142694	Observation	Aegilops geniculata		ESP	Spain		Santa Olalla del C	H	37.9049	-6.21679	499
00:00.0	BDBCV	113	Observation	Aegilops geniculata		ESP	Spain	Parque Natural de Penyagolosa		Cs			
	SIVIM	R-P09636:Aegi	Observation	Aegilops geniculata Roth	Roth	ES	Spain	entre SigÃ¼enza y Pelegrina			41.01	-2.64	1100
	SIVIM	R-P10471:Aegi	Observation	Aegilops geniculata Roth	Roth	ES	Spain	Salinas de Toscano , AlcalÃ¡ de los Gazule			36.47	-5.79	0
	SIVIM	R-P11658:Aegi	Observation	Aegilops geniculata Roth	Roth	ES	Spain	Tarifa			44.93	-6.04	0
	SIVIM	S-P01251:Aegi	Observation	Aegilops geniculata Roth	Roth	ES	Spain	MaldÃ , a VilamajÃ³			41.53	0.96	0
	SIVIM	S-P03968:Aegi	Observation	Aegilops geniculata Roth	Roth	ES	Spain	els Omellons, camÃ de l'IRYDA			41.44	0.96	0
1872-08-0	BC	SBB-1191	Specimen	Aegilops ovata L.	L.	ES	Spain	prope Barcinonem		B			
00:00.0	JBS	462-1	Specimen	Aegilops ovata L.	L.	ES	Spain	Escorca; Puig Roig		Mll	39.86	2.87	1

	GDA	GDA30014-1	Specimen	Aegilops ovata L.	L.	ES	Spain	Granada.		GR			0
00:00.0	SEV	99077-1	Specimen	Aegilops geniculata Roth	Roth	ES	Spain	Bornos		Ca			1
	SIVIM	T-P06963:Aegi	Observation	Aegilops geniculata Roth	Roth	ES	Spain	VillamaÃ±Ã¡n			42.24	-5.66	0
	SIVIM	T-P09153:Aegi	Observation	Aegilops geniculata Roth	Roth	ES	Spain	Portillo			41.44	-4.67	0
	SIVIM	T-P09351:Aegi	Observation	Aegilops geniculata Roth	Roth	ES	Spain	TamajÃ³n			40.92	-3.35	1000
	SIVIM	T-P10777:Aegi	Observation	Aegilops geniculata Roth	Roth	ES	Spain	Pozuelo de TÃ¡bara			41.78	-6	0
	SIVIM	T-P11869:Aegi	Observation	Aegilops geniculata Roth	Roth	ES	Spain	Valverde Enrique			42.24	-5.3	0
	SIVIM	T-P13215:Aegi	Observation	Aegilops geniculata Roth	Roth	ES	Spain	Aibar			42.53	-1.41	0
	ESP004	NC027463	Specimen	Aegilops geniculata Roth		ESP	Spain	Ports de Beceite, Roquetes, province of Tarragona					
00:00.0	MUB	102373-1	Specimen	Aegilops geniculata Roth	Roth	ES	Spain	Riopar; CaÃ±ada de los Mojones		Ab	38.447	-2.444	1260
00:00.0	GDA	GDA23046-1-2	Specimen	Aegilops geniculata Roth.	Roth.	ES	Spain	Granada, Padul, cerro de los Molino		GR			750
	SANT	16352	Specimen	Aegilops geniculata Roth		ES	Spain	RubiÃ¡. Cobas		Or			
	FUND. BIODIVER	1335999	Unknown	Aegilops geniculata Roth	Roth	ESP	Spain	Monreal, Higa		Na	42.1	-1.1	1280
	FUND. BIODIVER	1354405	Unknown	Aegilops geniculata Roth	Roth	ESP	Spain	Viguera		Lo	42.1	-2.1	1100
	FUND. BIODIVER	82306	Unknown	Aegilops geniculata Roth.	Roth.	ESP	Spain	Fresno de la Fuente		Sg	41.1	-3.1	
	SEV	11997-1	Specimen	Aegilops ovata L.	L.	ES	Spain	Madrid		M			1
00:00.0	MA	569312-1	Specimen	Aegilops geniculata Roth.	Roth.	ES	Spain	Siguero (AldealapeÃ±a)		Sg			
00:00.0	ABH	31838-1	Specimen	Aegilops geniculata Roth	Roth	ES	Spain	Orihuela; Dehesa de Campoamor		A	37.96	-0.8	
00:00.0	MGC	20416-1	Unknown	Aegilops geniculata Roth	Roth	ES	Spain	Sierra Tejeda. Entre AlcaucÃ-n y Pu		Gr	0	0	1
	IDBD-GN	42468	Observation	Aegilops geniculata Roth	Roth	ES	Spain	Caparreta	Gallipienzo	Na	42.5158	-1.4005	400
	IDBD-GN	42488	Observation	Aegilops geniculata Roth	Roth	ES	Spain		OyÃ³n	Vi	42.4987	-2.44756	450
	IDBD-GN	42499	Observation	Aegilops geniculata Roth	Roth	ES	Spain	RÃ-o Arga	Falces	Na	42.3675	-1.7929	300
	IDBD-GN	42510	Observation	Aegilops geniculata Roth	Roth	ES	Spain		Lumbier	Na	42.6585	-1.29924	440
	REDIAM-CMA	85752	Observation	Aegilops geniculata		ESP	Spain		AlcalÃ¡ de los Ga	Ca	36.5088	-5.68	200
00:00.0	REDIAM-CMA	111122	Observation	Aegilops geniculata		ESP	Spain		AlcalÃ¡ de los Ga	Ca	36.4537	-5.73779	99
00:00.0	REDIAM-CMA	125288	Observation	Aegilops geniculata		ESP	Spain		Aracena	H	37.8672	-6.46356	643
00:00.0	BDBCV	97	Observation	Aegilops geniculata		ESP	Spain	Parque Natural de Penyagolosa		Cs	40.17	-0.4	
	ADIMAN	33	Observation	Aegilops geniculata		ESP	Spain	EnguÃ-danos		CU	39.6715	-1.63478	
00:00.0	BC	597976	Specimen	Aegilops ovata L.	L.	ES	Spain	Almatret; SegriÃ : Vers Almatret		L	41.3	0.43	
	SIVIM	S-P09872:Aegi	Observation	Aegilops geniculata Roth	Roth	ES	Spain	prop del port de Tudons, serra Aitana (Pen			38.63	-0.35	1000
	SIVIM	S-P13995:Aegi	Observation	Aegilops geniculata Roth	Roth	ES	Spain	Pinhal da Senhora (Freixo do Meio)			39.38	-8.88	128
	SIVIM	T-P01860:Aegi	Observation	Aegilops geniculata Roth	Roth	ES	Spain	Lluc, prop de can Llovera (fons de la vall d'			39.74	2.76	0
	SIVIM	T-P03848:Aegi	Observation	Aegilops geniculata Roth	Roth	ES	Spain	0.5 km al NE de Aldea de S. Esteban			41.55	-3.35	0
	SIVIM	T-P05761:Aegi	Observation	Aegilops geniculata Roth	Roth	ES	Spain	HondÃ³n de las Nieves			38.19	-0.94	0
	SIVIM	T-P06424:Aegi	Observation	Aegilops geniculata Roth	Roth	ES	Spain	Cortes de PallÃ¡s			39.19	-1.03	340
00:00.0	MA	528744-1	Specimen	Aegilops geniculata Roth	Roth	ES	Spain	ChinchÃ³n, Casa de David		M			
00:00.0	UNEX	10376-1	Observation	Aegilops geniculata Roth	_	ESP	Spain	Feria: Pastizales. 29SQC16		Ba	38.5	-6.6	
00:00.0	UNEX	10378-1	Observation	Aegilops geniculata Roth	_	ESP	Spain	Alconera: Sra. Alconera. 29SQC15		Ba	38.4	-6.6	
00:00.0	BDBCV	597329	Observation	Aegilops geniculata		ESP	Spain		Bocairent	Valenc	38.729	-0.555	
	FUND. BIODIVER	117644	Unknown	Aegilops ovata L.	L.	ESP	Spain	Castrillo de la Reina		Bu	41.1	-2.1	
	FUND. BIODIVER	51241	Unknown	Aegilops ovata subsp. triaristata	L.	ESP	Spain	Villarino		Sa	41.1	-6.1	

	FUND. BIODIVER	56190	Unknown	Aegilops ovata L.	L.	ESP	Spain	Pedrajas de San Esteban		Va	41.1	-4.1	
	FUND. BIODIVER	60943	Unknown	Aegilops ovata subsp. triaristata	L.	ESP	Spain	Tejeda y Segoyuela		Sa			
00:00.0	REDIAM-CMA	27877	Observation	Aegilops geniculata		ESP	Spain		Olvera	Ca	36.9663	-5.11216	602
00:00.0	REDIAM-CMA	46746	Observation	Aegilops geniculata		ESP	Spain		Huelma	J	37.7081	-3.46371	1600
00:00.0	GDAC	GDAC42433-1	Specimen	Aegilops geniculata Roth.	Roth.	ES	Spain	Granada, SÂª de LÃºjar, cerca del E		GR			0
00:00.0	SALA	5582-1	Specimen	Aegilops ovata L. subsp. triaristat	(Willd.) Rouy	ES	Spain	_; San Esteban de la Sierra		Sa			
	SIVIM	R-P09637:Aegi	Observation	Aegilops geniculata Roth	Roth	ES	Spain	entre SigÃ¼enza y Pelegrina			41.01	-2.64	1100
	SIVIM	R-P10530:Aegi	Observation	Aegilops geniculata Roth	Roth	ES	Spain	De Benalup a Cantarranas , Vejer de la Fro			36.28	-5.89	0
	SIVIM	R-P11659:Aegi	Observation	Aegilops geniculata Roth	Roth	ES	Spain	Tarifa			44.93	-6.04	0
	SIVIM	S-P01253:Aegi	Observation	Aegilops geniculata Roth	Roth	ES	Spain	els Omellons, camÃ- de l'IRYDA			41.44	0.96	0
	SIVIM	S-P03970:Aegi	Observation	Aegilops geniculata Roth	Roth	ES	Spain	Vinaixa, la Solana			41.44	0.84	0
	IDBD-GN	42444	Observation	Aegilops geniculata Roth	Roth	ES	Spain	Sarasate	Iza	Na	42.8988	-1.78257	
	IDBD-GN	42453	Observation	Aegilops geniculata Roth	Roth	ES	Spain		GoÃ±i	Na	42.8498	-1.83861	
00:00.0	SALA	4627-1	Specimen	Aegilops ovata L.	L.	ES	Spain	_; Carretera de Jerez a Medina cerc		Ca			
00:00.0	SALA	52032-1	Specimen	Aegilops geniculata Roth	Roth	ES	Spain	_; Fresno de la Ribera		Za			
00:00.0	BDBCV	599221	Observation	Aegilops geniculata		ESP	Spain		Alcoy	Alicant	38.664	-0.477	
	FUND. BIODIVER	1376732	Unknown	Aegilops geniculata Roth.	Roth.	ESP	Spain	Sierra de Baza, Parque Natural		Gr	37.1	-2.1	
	FUND. BIODIVER	1463972	Unknown	Aegilops geniculata Roth	Roth	ESP	Spain	Montroig		L	41.1	0.1	400
	FUND. BIODIVER	918140	Unknown	Aegilops ovata L.	L.	ESP	Spain	Plana de Vich		B	41.1	2.1	
	FUND. BIODIVER	930420	Unknown	Aegilops ovata L.	L.	ESP	Spain	L'Aleixar-Vilaplana del Camp		T	41.1	0.1	
00:00.0	SALA	42465-1	Specimen	Aegilops geniculata (L.) Roth.	(L.) Roth.	ES	Spain	_; Encinas de Esgueva		Va			
00:00.0	SALA	52036-1	Specimen	Aegilops geniculata Roth	Roth	ES	Spain	_; Santa MarÃ-a de la Vega		Za			
00:00.0	REDIAM-CMA	144079	Observation	Aegilops geniculata		ESP	Spain		Casares	Ma	36.3977	-5.2619	79
	SIVIM	T-P06967:Aegi	Observation	Aegilops geniculata Roth	Roth	ES	Spain	Puente Castro			42.51	-5.55	0
	SIVIM	T-P09156:Aegi	Observation	Aegilops geniculata Roth	Roth	ES	Spain	CabezÃ³n			41.71	-4.68	0
	SIVIM	T-P09354:Aegi	Observation	Aegilops geniculata Roth	Roth	ES	Spain	ValdepeÃ±as de la Sierra			40.83	-3.47	0
	SIVIM	T-P10780:Aegi	Observation	Aegilops geniculata Roth	Roth	ES	Spain	Moreruela de TÃ¡bara			41.78	-5.88	0
	SIVIM	T-P13222:Aegi	Observation	Aegilops geniculata Roth	Roth	ES	Spain	De San AdriÃ¡n a Peralta			42.26	-1.9	0
	BDBCV-General	30392	Observation	Aegilops geniculata		ESPA	Spain	Tibi	L'AlcoiÃ	Alicant	38.5092	-0.64898	
	BDBCV-General	34269	Observation	Aegilops geniculata		ESPA	Spain	Agres	El Comtat	Alicant	38.7769	-0.52512	
	BDBCV-General	77616	Observation	Aegilops geniculata		ESPA	Spain	El Toro	El Alto Palancia	Castel	39.9542	-0.83434	
00:00.0	COFC	11984-1	Specimen	Aegilops geniculata Roth	Roth	ES	Spain	Lucena; Jauja		Co	37	-4	1
00:00.0	SALA	24376-1	Specimen	Aegilops geniculata Roth	Roth	ES	Spain	_; San CebriÃ¡n de Mazote		Va			
00:00.0	SALA	30596-1	Specimen	Aegilops geniculata Roth	Roth	ES	Spain	_; Castrillo de la GuareÃ±a		Za			
00:00.0	COA	28711-1	Specimen	Aegilops geniculata Roth	Roth	ES	Spain	Antequera, Monte Hacho, Partido de		Ma	36.95	-4.57	
00:00.0	BDBCV	108	Observation	Aegilops geniculata		ESP	Spain	Parque Natural de Penyagolosa		Cs			
00:00.0	COA	27409-1	Specimen	Aegilops geniculata Roth	Roth	ES	Spain	AlhaurÃ-n de la Torre, subida hacia		Ma	36.59	-4.56	
	FUND. BIODIVER	1696522	Unknown	Aegilops geniculata Roth	Roth	ESP	Spain	Puertomingalvo, El Letrado		Te	40.1	-0.1	1500
00:00.0	SEV	108264-1	Specimen	Aegilops geniculata Roth	Roth	ES	Spain	Entre Las Pajanosas y Guillena		Se			1
00:00.0	REDIAM-CMA	243390	Observation	Aegilops geniculata		ESP	Spain		Cortegana	H	37.9188	-6.83212	612
00:00.0	REDIAM-CMA	276041	Observation	Aegilops geniculata		ESP	Spain		Pedroche	Co	38.4834	-4.74614	554

00:00.0	REDIAM-CMA	283073	Observation	Aegilops geniculata		ESP	Spain		Ãšbeda	J	38.1007	-3.36147	526
00:00.0	SALA	56962-1	Specimen	Aegilops geniculata Roth	Roth	ES	Spain	_; Pelabravo		Sa	40.89	-5.61	
00:00.0	SALA	60046-1	Specimen	Aegilops ovata L.	L.	ES	Spain	_; PeÃ±ausende		Za			
00:00.0	GDA	GDA48228-1-2	Specimen	Aegilops geniculata Roth	Roth	ES	Spain	Granada, SÂª de las Chanzas, Mon		GR			900
00:00.0	HUAL	5689-1	Specimen	Aegilops geniculata Roth	Roth	ES	Spain	SÂª de GÃ¡dor, Balsa de La SeÃ±o		Al	36.879	-2.792	
	SIVIM	T-P30047:Aegi	Observation	Aegilops geniculata Roth	Roth	ES	Spain	El Campichuelo, Cofrentes, Valencia			39.19	-1.14	0
	SIVIM	T-P30114:Aegi	Observation	Aegilops geniculata Roth	Roth	ES	Spain	El Campichuelo, Cofrentes, Valencia			39.19	-1.14	0
	SIVIM	U-P02501:Aegi	Observation	Aegilops geniculata Roth	Roth	ES	Spain	Benissa Calp			38.63	0.01	0
	SIVIM	U-P02919:Aegi	Observation	Aegilops geniculata Roth	Roth	ES	Spain	Pego Vall d'Ebo			38.81	-0.23	0
	SIVIM	U-P06713:Aegi	Observation	Aegilops geniculata Roth	Roth	ES	Spain	CabeÃ§a Aguda			37.13	-8.21	190
	SIVIM	U-P08063:Aegi	Observation	Aegilops geniculata Roth	Roth	ES	Spain	Collado del Bote, La Nava, Berzocana			39.36	-5.55	0
	BDBCV-General	274783	Observation	Aegilops geniculata		ESPA	Spain	Chella	La Canal de Nava	Valenc	39.0517	-0.74664	
00:00.0	COFC	46881-1	Specimen	Aegilops geniculata Roth	Roth	ES	Spain	rÃƒÂ-o BembÃƒÂ©zar; inmediacion		Co			1
	FUND. BIODIVER	1000497	Unknown	Aegilops geniculata		ESP	Spain	Venta de Cabrejas		Cu	39.1	-2.1	
	FUND. BIODIVER	1701107	Unknown	Aegilops geniculata		ESP	Spain	Vilafranca, Masia de la Rambla		Te	40.1	-0.1	1140
	FUND. BIODIVER	1715683	Unknown	Aegilops geniculata		ESP	Spain	Vilanova de la Muga, a tocar de les		Ge	42.1	2.1	
00:00.0	SEV	50703-1	Specimen	Aegilops geniculata Roth	Roth	ES	Spain	Montellano, Sierra de Montellano		Se			400
	RUS001	VIR100602225	Specimen	Aegilops ovata L.		ESP	Spain						
	RUS001	VIR100602160	Specimen	Aegilops ovata L.		ESP	Spain						
00:00.0	MA	772819-1	Specimen	Aegilops geniculata Roth.	Roth.	ES	Spain	Rute; Camorro de la Isla. Margen de		Co			
	FUND. BIODIVER	1052572	Unknown	Aegilops geniculata Roth	Roth	ESP	Spain	Montes de Propios de Jerez de la F		Ca	36.1	-5.1	
	FUND. BIODIVER	1067626	Unknown	Aegilops ovata L.	L.	ESP	Spain	AlcalÃ¡ de Guadaira		Se	37.1	-5.1	
00:00.0	FUND. BIODIVER	1835075	Unknown	Aegilops geniculata Roth	Roth	ESP	Spain	Malagon-Los Quiles, olivos		CR	39.1	-3.1	
00:00.0	REDIAM-CMA	335070	Observation	Aegilops geniculata		ESP	Spain		Los Barrios	Ca	36.1966	-5.44382	49
00:00.0	REDIAM-CMA	381562	Observation	Aegilops geniculata		ESP	Spain		Teba	Ma	36.9583	-4.89506	400
00:00.0	REDIAM-CMA	382694	Observation	Aegilops geniculata		ESP	Spain				37.8492	-3.13436	
00:00.0	FUND. BIODIVER	1774961	Unknown	Aegilops geniculata Roth	Roth	ESP	Spain	Monzon, camino del Valle Tamarite,		Hu	41.1	0.1	290
	FUND. BIODIVER	1811880	Unknown	Aegilops geniculata Roth.	Roth.	ESP	Spain	Salvatierra, Barranco de la Garona		Na	42.1	-0.1	600
	SIVIM	U-P08620:Aegi	Observation	Aegilops geniculata Roth	Roth	ES	Spain	Sierra del Carche			38.38	-1.16	1060
	BDBCV-General	74114	Observation	Aegilops geniculata		ESPA	Spain	Villamalur	El Alto Mijares	Castel	39.9446	-0.36651	
	BDBCV-General	76828	Observation	Aegilops geniculata		ESPA	Spain	El Toro	El Alto Palancia	Castel	39.9542	-0.83434	
00:00.0	REDIAM-CMA	385671	Observation	Aegilops geniculata		ESP	Spain		Marbella	Ma	36.5376	-4.93724	284
00:00.0	COFC	11966-1	Specimen	Aegilops geniculata Roth	Roth	ES	Spain	Priego de CÃ³rdoba; Sierra de Alba		Co	37	-4	1
00:00.0	COFC	11992-1	Specimen	Aegilops geniculata Roth	Roth	ES	Spain	Luque; cerro del Algarrobo		Co	37	-4	1
00:00.0	COFC	16299-1	Specimen	Aegilops geniculata Roth	Roth	ES	Spain	BelalcÃƒÂ¡zar; carretera a Cabeza		Co	38	-5	1
00:00.0	REDIAM-CMA	382550	Observation	Aegilops geniculata		ESP	Spain		Carratraca	Ma	36.8457	-4.79154	698
00:00.0	REDIAM-CMA	383266	Observation	Aegilops geniculata		ESP	Spain		Casares	Ma	36.4311	-5.24556	274
00:00.0	COFC	11986-1	Specimen	Aegilops geniculata Roth	Roth	ES	Spain	Priego de CÃƒÂ³rdoba; caserÃƒÂ-o		Co	37	-4	1
	SIVIM	P-P08762:Aegi	Observation	Aegilops geniculata Roth	Roth	ES	Spain	Rodalies del Mas de Trencaladres; Fredes			40.7	0.15	1150
	SIVIM	P-P08889:Aegi	Observation	Aegilops geniculata Roth	Roth	ES	Spain	Barranc de Closetes; Horta de Sant Joan			40.79	0.27	0
	SIVIM	P-P09015:Aegi	Observation	Aegilops geniculata Roth	Roth	ES	Spain	Tossal d'en GrillÃ³; Prat de Comte			40.88	0.26	0

	SIVIM	P-P11097:Aegi	Observation	Aegilops geniculata Roth	Roth	ES	Spain	EstaciÃ³ de Freginals			40.62	0.39	115
	SIVIM	Q-P00142:Aegi	Observation	Aegilops geniculata Roth	Roth	ES	Spain	la Figuera, prÃ²xim al poble			41.16	0.73	0
	SIVIM	U-P08152:Aegi	Observation	Aegilops geniculata Roth	Roth	ES	Spain	Dehesa Boyal, LogrosÃ¡n,			39.27	-5.55	0
	SIVIM	U-P13297:Aegi	Observation	Aegilops geniculata Roth	Roth	ES	Spain	Ctra. AndÃºjar-Arjona, Km. 6			38.03	-4.13	260
	RUS001	VIR100602219	Specimen	Aegilops ovata L.		ESP	Spain						
00:00.0	UPS	V-147335	Specimen	Aegilops ovata		Spain	Spain			Madrid			450
00:00.0	MA	626604-1	Specimen	Aegilops ovata L.	L.	ES	Spain	Embalse de Ulldecona, pista del Va		Cs			
	FUND. BIODIVER	1035623	Unknown	Aegilops ovata		ESP	Spain	Laderas del Arroyo de Paredones, S		Ma	36.1	-4.1	
	FUND. BIODIVER	1043751	Unknown	Aegilops geniculata Roth	Roth	ESP	Spain	Antequera		Ma	36.1	-4.1	
	FUND. BIODIVER	1062079	Unknown	Aegilops geniculata Roth	Roth	ESP	Spain	Sierra de Aracena		H	37.1	-6.1	
	FUND. BIODIVER	1070040	Unknown	Aegilops ovata L.	L.	ESP	Spain	Carmona		Se	37.1	-5.1	
00:00.0	FUND. BIODIVER	1835081	Unknown	Aegilops geniculata Roth	Roth	ESP	Spain	Torralba de Calatrava, Campomojad		CR	38.1	-3.1	610
00:00.0	HSS	13747	Specimen	Aegilops geniculata Roth	Roth	ES	Spain	Alconera, Puerto de Calatrava		Ba	38.4149	-6.42321	500
00:00.0	GDA	GDA30018-1-2	Specimen	Aegilops geniculata Roth.	Roth.	ES	Spain	Cabrera, puerto.		PM			0
00:00.0	REDIAM-CMA	383722	Observation	Aegilops geniculata		ESP	Spain		Ronda	Ma	36.7499	-5.18745	563
00:00.0	REDIAM-CMA	384670	Observation	Aegilops geniculata		ESP	Spain		Cambil	J	37.6923	-3.51134	1052
00:00.0	REDIAM-CMA	392101	Observation	Aegilops geniculata		ESP	Spain		Olvera	Ca	36.9663	-5.11219	602
	SIVIM	P-P08866:Aegi	Observation	Aegilops geniculata Roth	Roth	ES	Spain	Coll Blanc; Horta de Sant Joan			40.88	0.26	0
	SIVIM	P-P08900:Aegi	Observation	Aegilops geniculata Roth	Roth	ES	Spain	Torrent de l'Avellanar; CoratxÃ			40.61	0.04	0
	SIVIM	P-P09117:Aegi	Observation	Aegilops geniculata Roth	Roth	ES	Spain	Coll Blanc; Horta de Sant Joan			40.88	0.26	0
	SIVIM	P-P11253:Aegi	Observation	Aegilops geniculata Roth	Roth	ES	Spain	Barranc d'Aiguaoliva			40.43	0.28	0
	SIVIM	Q-P00911:Aegi	Observation	Aegilops geniculata Roth	Roth	ES	Spain	BesalÃº, prop del MolÃ- Nou			42.18	2.51	160
00:00.0	BDBCV	598770	Observation	Aegilops geniculata		ESP	Spain	Parc Natural de la	Alcoy	Alicant	38.728	-0.509	
	FUND. BIODIVER	1370924	Unknown	Aegilops geniculata Roth	Roth	ESP	Spain	Guadalix de la Sierra		M	40.1	-3.1	
	FUND. BIODIVER	1463962	Unknown	Aegilops geniculata Roth	Roth	ESP	Spain	Pla de les Gesses		L	41.1	0.1	320
	FUND. BIODIVER	925115	Unknown	Aegilops ovata L.	L.	ESP	Spain	Cuenca del GaiÃ¡		T	41.1	1.1	
	ESP004	NC044551	Specimen	Aegilops geniculata Roth		ESP	Spain	Molino de la Villa, Cantalapiedra, province			41.15	-5.16667	785
	FUND. BIODIVER	1141567	Unknown	Aegilops geniculata Roth	Roth	ESP	Spain	VillamaÃ±Ã¡n		Le	42.1	-5.1	760
	FUND. BIODIVER	116430	Unknown	Aegilops geniculata Roth.	Roth.	ESP	Spain	Aguasal		Va	41.1	-4.1	
	FUND. BIODIVER	58087	Unknown	Aegilops geniculata Roth	Roth	ESP	Spain	Cantalapiedra		Sa	40.1	-5.1	
	FUND. BIODIVER	62079	Unknown	Aegilops ovata subsp. triaristata		ESP	Spain	Castillejo de MartÃ-n Viejo, Paradin		Sa	40.1	-6.1	
00:00.0	REDIAM-CMA	177562	Observation	Aegilops geniculata		ESP	Spain		Villacarrillo	J	38.0679	-2.93999	1284
00:00.0	GDAC	GDAC32220-1	Specimen	Aegilops geniculata Roth.	Roth.	ES	Spain	Granada, SÂª de Parapanda, Casa		GR			0
00:00.0	BC	126481	Specimen	Aegilops ovata L.	L.	ES	Spain	Palma de Mallorca; Cabrera		PM	39.16	2.94	
1872-08-0	BC	608748	Specimen	Aegilops ovata L.	L.	ES	Spain	Barcelona; Regionis sub-montanae,		B	41.41	2.1	
00:00.0	ABH	3762-1	Specimen	Aegilops ovata L.	L.	ES	Spain	Balones;		A	38.73	-0.35	
00:00.0	ABH	39281-1	Specimen	Aegilops geniculata Roth	Roth	ES	Spain	Alicante; campos de cultivo, prox. S		A	38.37	-0.55	
00:00.0	UNEX	26933-1	Observation	Aegilops geniculata Roth	_	ESP	Spain	Magacela: Sustrato calizo. CercanÃ		Ba	38.8	-5.8	
00:00.0	BDBCV	109	Observation	Aegilops geniculata		ESP	Spain	Parque Natural de Penyagolosa		Cs			
00:00.0	HSS	5751	Specimen	Aegilops geniculata Roth	Roth	ES	Spain	Olivenza, Sierra de Alor		Ba	38.6982	-7.10294	
	SIVIM	T-P06449:Aegi	Observation	Aegilops geniculata Roth	Roth	ES	Spain	Cofrentes			39.19	-1.26	0

	SIVIM	T-P07604:Aegi	Observation	Aegilops geniculata Roth	Roth	ES	Spain	Carchelejo (JaÃ©n)			37.58	-3.67	1420
	SIVIM	T-P07980:Aegi	Observation	Aegilops geniculata Roth	Roth	ES	Spain	Artajona, hacia Larraga, bco. Duiderra			42.53	-1.9	330
	SIVIM	T-P09227:Aegi	Observation	Aegilops geniculata Roth	Roth	ES	Spain	Soto de Cerrato			41	-4.42	0
	SIVIM	T-P10392:Aegi	Observation	Aegilops geniculata Roth	Roth	ES	Spain	Proximidades Cueva Bermeja. Ronda			36.66	-5.12	1150
	SIVIM	T-P11558:Aegi	Observation	Aegilops geniculata Roth	Roth	ES	Spain	La Robla			42.78	-5.68	1190
	SIVIM	T-P11559:Aegi	Observation	Aegilops geniculata Roth	Roth	ES	Spain	Valle del A. Huergas			42.78	-5.68	1020
	SIVIM	T-P13209:Aegi	Observation	Aegilops geniculata Roth	Roth	ES	Spain	Sta. Cruz del Campezo			42.63	-2.39	0
00:00.0	REDIAM-CMA	34340	Observation	Aegilops geniculata		ESP	Spain		Torres	J	37.7436	-3.51154	1400
00:00.0	REDIAM-CMA	65782	Observation	Aegilops geniculata		ESP	Spain		GaucÃ-n	Ma	36.4723	-5.35533	290
00:00.0	REDIAM-CMA	74753	Observation	Aegilops geniculata		ESP	Spain		Ronda	Ma	36.6905	-5.05548	1300
	ESP004	NC024072	Specimen	Aegilops geniculata Roth		ESP	Spain	Durcal, province of Granada			36.9833	-3.55	783
00:00.0	COFC	41281-1	Specimen	Aegilops geniculata Roth	Roth	ES	Spain	Baena; Torre Morena		Co	37	-4	1
	SIVIM	R-P09417:Aegi	Observation	Aegilops geniculata Roth	Roth	ES	Spain	Bochones			41.19	-2.88	1180
	SIVIM	R-P10352:Aegi	Observation	Aegilops geniculata Roth	Roth	ES	Spain	Los Santos , AG			36.38	-5.78	1
	SIVIM	R-P11635:Aegi	Observation	Aegilops geniculata Roth	Roth	ES	Spain	AlcalÃ¡ de los Gazules, La Joya			36.38	-5.78	0
	SIVIM	S-P01006:Aegi	Observation	Aegilops geniculata Roth	Roth	ES	Spain	Sant Boi de Llobregat, vora el cementiri			41.27	1.92	0
	SIVIM	S-P01717:Aegi	Observation	Aegilops geniculata Roth	Roth	ES	Spain	Salines de Vilanova de la Sal			41.88	0.7	0
	SIVIM	S-P03955:Aegi	Observation	Aegilops geniculata Roth	Roth	ES	Spain	l'Albi			41.35	0.84	0
	FUND. BIODIVER	118441	Unknown	Aegilops geniculata Roth.	Roth.	ESP	Spain	La Pola de GordÃ³n, Beberino		Le	42.1	-5.1	
	REDIAM-CMA	92247	Observation	Aegilops geniculata		ESP	Spain		Jerez de la Fronte	Ca	36.6103	-5.5419	310
00:00.0	REDIAM-CMA	108007	Observation	Aegilops geniculata		ESP	Spain		La Granada de R	H	37.7567	-6.48854	507
00:00.0	REDIAM-CMA	121488	Observation	Aegilops geniculata		ESP	Spain		Ronda	Ma	36.6881	-5.04452	1299
	SANT	9803	Specimen	Aegilops ovata L.		ES	Spain	QuereÃ±o		Le			
00:00.0	SALA	16052-1	Specimen	Aegilops ovata L. subsp. triaristat	(Willd.) Rouy	ES	Spain	_; Villares de Yeltes, Pedro Ã•varo		Sa			
00:00.0	UPS	V-147200	Specimen	Aegilops ovata		Spain	Spain	Strax utanfÃ¶r Ciudad Universitaria		Madrid			
	SIVIM	S-P14649:Aegi	Observation	Aegilops geniculata Roth	Roth	ES	Spain	Km. 10 Carretera del Picacho (A.G) ,			36.47	-5.67	0
	SIVIM	T-P03841:Aegi	Observation	Aegilops geniculata Roth	Roth	ES	Spain	2 km al SW de Camarma del CaÃ±o			40.55	-3.47	0
	SIVIM	T-P04200:Aegi	Observation	Aegilops geniculata Roth	Roth	ES	Spain	6 km al SE de Los Yesares			39.02	-1.72	0
	SIVIM	T-P06417:Aegi	Observation	Aegilops geniculata Roth	Roth	ES	Spain	Aldea de La Torre			39.64	-1.36	0
00:00.0	W	41845	Unknown	Aegilops geniculata Roth		ESP	Spain	Prov. Mursia: Sierra de la Muela: N von Alhama de Mursia, W-Se					600
	IPK	70017	Living	Aegilops geniculata Roth		ESP	Spain	Col de Veleta, Sierra Nevada,Prov. de Granada, Espagne					
	IDBD-GN	42489	Observation	Aegilops geniculata Roth	Roth	ES	Spain	Barranco Grande	Bardenas Reales	Na	42.2291	-1.50471	270
	IDBD-GN	42501	Observation	Aegilops geniculata Roth	Roth	ES	Spain	RÃ-o AragÃ³n	UjuÃ©	Na	42.4261	-1.4271	400
00:00.0	COFC	36342-1	Specimen	Aegilops geniculata Roth	Roth	ES	Spain	Encinas Reales; Ventas del rÃƒÂ-o		Co			1
	RUS001	VIR100602108	Specimen	Aegilops ovata L.		ESP	Spain						
00:00.0	BDBCV	332305	Observation	Aegilops geniculata	_	ES	Spain		La Nucia	A	38.59	-0.07	
	FUND. BIODIVER	1946398	Unknown	Aegilops geniculata Roth	Roth	ESP	Spain	Puerto de la Caracollera		CR	38.1	-4.1	
00:00.0	COFC	41151-1	Specimen	Aegilops geniculata Roth	Roth	ES	Spain	Baena; rÃƒÂ-o Guadajoz; entre el m		Co	37	-4	1
00:00.0	COFC	41288-1	Specimen	Aegilops geniculata Roth	Roth	ES	Spain	Lucena; rÃƒÂ-o Anzur; puente de la		Co	37	-4	1
00:00.0	MGC	41211-1	Unknown	Aegilops ovata L.	L.	ES	Spain	Antequera; Torcal de Antequera. RÃ		Ma	0	0	1
	BDBCV-General	66452	Observation	Aegilops geniculata		ESPA	Spain	Ares del Maestre	L'Alt Maestrat	Castel	40.3889	-0.11368	

00:00.0	REDIAM-CMA	774	Observation	Aegilops geniculata		ESP	Spain		JaÃ©n	J	37.8611	-3.67092	477
00:00.0	REDIAM-CMA	10315	Observation	Aegilops geniculata		ESP	Spain		Ronda	Ma	36.7499	-5.18735	563
00:00.0	REDIAM-CMA	18453	Observation	Aegilops geniculata		ESP	Spain		CaÃ±ete la Real	Ma	36.9835	-5.02176	715
00:00.0	REDIAM-CMA	402379	Observation	Aegilops geniculata		ESP	Spain		Segura de la Sier	J	38.2142	-2.64409	1327
00:00.0	REDIAM-CMA	408257	Observation	Aegilops geniculata		ESP	Spain		Frailes	J	37.5065	-3.83089	1199
00:00.0	REDIAM-CMA	415837	Observation	Aegilops geniculata		ESP	Spain		Cazorla	J	37.8826	-2.89774	1521
00:00.0	REDIAM-CMA	420119	Observation	Aegilops geniculata		ESP	Spain		La Granada de R	H	37.7688	-6.51256	453
00:00.0	SEV	100516-1	Specimen	Aegilops geniculata Roth	Roth	ES	Spain	Behind Facinas, Tarifa district		Ca			50
00:00.0	SEV	101906-1	Specimen	Aegilops geniculata Roth	Roth	ES	Spain	El Garrobo		Se			1
00:00.0	SEV	108140-1	Specimen	Aegilops geniculata Roth	Roth	ES	Spain	MatalascaÃ±as. Camping Catapun		H			1
	SIVIM	Q-P03037:Aegi	Observation	Aegilops geniculata Roth	Roth	ES	Spain	La Llacuna, carrer no pavimentat (Anoia)			41.45	1.44	0
	SIVIM	Q-P03604:Aegi	Observation	Aegilops geniculata Roth	Roth	ES	Spain	San Vicente			40.08	-0.53	0
	SIVIM	Q-P06540:Aegi	Observation	Aegilops geniculata Roth	Roth	ES	Spain	Els Segalassos, cap a Vilanova de Prades			41.26	0.85	0
	SIVIM	R-P00051:Aegi	Observation	Aegilops geniculata Roth	Roth	ES	Spain	rodalies d'Alsamora (Montsec d'Ares)			42.06	0.7	0
	SIVIM	R-P08210:Aegi	Observation	Aegilops geniculata Roth	Roth	ES	Spain	Castell de Sant Jaume (CastellolÃ-)			41.54	1.68	0
00:00.0	GDA	GDA30017-1-1	Specimen	Aegilops ovata L.	L.	ES	Spain	Granada, Dehesa de Montejo.		GR			0
	BDBCV-General	2704	Observation	Aegilops geniculata		ESPA	Spain	Andilla	Los Serranos	Valenc	39.8642	-0.83717	
	BDBCV-General	274333	Observation	Aegilops geniculata		ESPA	Spain	Enguera	La Canal de Nava	Valenc	39.0517	-0.74664	
00:00.0	HUAL	1135-1	Specimen	Aegilops geniculata Roth	Roth	ES	Spain	SÂª de Cazorla, Fuente de la Teja		J	37.97	-2.903	
00:00.0	SALA	115619-1	Specimen	Aegilops geniculata Roth	Roth	ES	Spain	RubiÃ¡, Vilar de Silva		Or	42.46	-6.84	
00:00.0	UPS	V-147200	Specimen	Aegilops ovata		Spain	Spain			Madrid			650
	FUND. BIODIVER	1060681	Unknown	Aegilops ovata L.	L.	ESP	Spain			H			
	FUND. BIODIVER	1067631	Unknown	Aegilops ovata L.	L.	ESP	Spain	Las Rozas, Encarnaciones, alreded		Se	36.1	-5.1	
	FUND. BIODIVER	1835080	Unknown	Aegilops geniculata Roth	Roth	ESP	Spain	Tablas de Daimiel, isla del Pan		CR	38.1	-3.1	
	FUND. BIODIVER	1886757	Unknown	Aegilops geniculata		ESP	Spain	Renedo de Esgueva, zonas altas de		Va	41.1	-4.1	
	DEU146	AE 588	Specimen	Aegilops geniculata Roth		ESP	Spain	Pablo Romaso, Strae Sevilla - Huelva, 4 km von Sanlcar la Mayo					40
00:00.0	REDIAM-CMA	383541	Observation	Aegilops geniculata		ESP	Spain				37.7866	-3.2805	
00:00.0	REDIAM-CMA	384591	Observation	Aegilops geniculata		ESP	Spain				37.6353	-3.15607	
00:00.0	REDIAM-CMA	387197	Observation	Aegilops geniculata		ESP	Spain		Olvera	Ca	36.9904	-5.12281	500
00:00.0	REDIAM-CMA	388758	Observation	Aegilops geniculata		ESP	Spain		Priego de CÃ³rdo	Co	37.384	-4.31477	639
00:00.0	ABH	33518-1	Specimen	Aegilops geniculata Roth	Roth	ES	Spain	Altea; SÂª de BÃ¨rnia		A	38.64	-0.02	
	SIVIM	P-P08865:Aegi	Observation	Aegilops geniculata Roth	Roth	ES	Spain	Barranc del Camp; PaÃ¼ls			40.88	0.26	0
	SIVIM	P-P08898:Aegi	Observation	Aegilops geniculata Roth	Roth	ES	Spain	Coll Blanc; Horta de Sant Joan			40.88	0.26	0
	SIVIM	P-P09114:Aegi	Observation	Aegilops geniculata Roth	Roth	ES	Spain	PaÃ¼ls, rodalies			40.89	0.38	310
	SIVIM	P-P11229:Aegi	Observation	Aegilops geniculata Roth	Roth	ES	Spain	La Foia			40.62	0.51	0
	SIVIM	Q-P00856:Aegi	Observation	Aegilops geniculata Roth	Roth	ES	Spain	TortellÃ , prop de can Quetxa, vora el Llierc			42.18	2.51	210
00:00.0	MA	700389-1	Specimen	Aegilops geniculata Roth	Roth	ES	Spain	Almagro, volcÃ¡n de lYezosa, sobre		CR	38	-3	
	MA	693426-1	Specimen	Aegilops geniculata Roth.	Roth.	ES	Spain	Jaca, au confluent du rio Gas et du		Hu	42	0	
00:00.0	HUAL	1136-1	Specimen	Aegilops geniculata Roth	Roth	ES	Spain	SÂª de Cazorla, de La Fuente del O		J	37.907	-2.949	
	ESP004	NC024051	Specimen	Aegilops geniculata Roth		ESP	Spain	Alcornoquillo, Alhama de Granada, provinc			36.9333	-4.01667	1078
00:00.0	HSS	12594	Specimen	Aegilops geniculata Roth	Roth	ES	Spain	Villafranca de los Barros		Ba	38.5924	-6.30208	

	FUND. BIODIVER	1340021	Unknown	Aegilops geniculata Roth	Roth	ESP	Spain	Madrid, Campus Universitario Monc		M	39.1	-3.1	
	FUND. BIODIVER	80849	Unknown	Aegilops geniculata Roth	Roth	ESP	Spain	SepÃºlveda, SepÃºlveda, en la expl		Sg	41.1	-3.1	
	FUND. BIODIVER	860300	Unknown	Aegilops ovata L.	L.	ESP	Spain	Alrededores de Polop		A	38.1	0.9	
00:00.0	SEV	107984-1	Specimen	Aegilops geniculata Roth	Roth	ES	Spain	Sierra de las Nieves		Ma			1
00:00.0	SEV	108250-1	Specimen	Aegilops geniculata Roth	Roth	ES	Spain	Priego de CÃ³rdoba. Arcenes de la		Co			1
00:00.0	SEV	108286-1	Specimen	Aegilops geniculata Roth	Roth	ES	Spain	Puente Genil, entre el rÃo Anzur y		Co			1
00:00.0	Marimurtra	4474-1	Specimen	Aegilops geniculata L. var. caprer	P.C. Palau F	ES	Spain	Cabrera; Alrededores del Puerto		Mll			
00:00.0	REDIAM-CMA	78753	Observation	Aegilops geniculata		ESP	Spain		MarÃa	Al	37.7033	-2.24312	1135
00:00.0	REDIAM-CMA	83852	Observation	Aegilops geniculata		ESP	Spain		Priego de CÃ³rdo	Co	37.3713	-4.28262	1266
00:00.0	REDIAM-CMA	121811	Observation	Aegilops geniculata		ESP	Spain		Torres	J	37.7366	-3.50979	1587
	IDBD-GN	42424	Observation	Aegilops geniculata Roth	Roth	ES	Spain		Tudela	Na	42.0368	-1.61192	
	SANT	27300	Specimen	Aegilops geniculata Roth		ES	Spain	RubiÃ¡,Vilardesilva		Or			
	SIVIM	S-P14653:Aegi	Observation	Aegilops geniculata Roth	Roth	ES	Spain	Ermita de los Santos (A.G)			36.38	-5.78	0
	SIVIM	T-P03842:Aegi	Observation	Aegilops geniculata Roth	Roth	ES	Spain	5 km al SW de Torrelaguna			40.73	-3.59	0
	SIVIM	T-P04203:Aegi	Observation	Aegilops geniculata Roth	Roth	ES	Spain	1 km al SW de El Viso			39.19	-1.26	0
	SIVIM	T-P06418:Aegi	Observation	Aegilops geniculata Roth	Roth	ES	Spain	Alcotas			39.82	-1.01	0
	FUND. BIODIVER	56191	Unknown	Aegilops ovata L.	L.	ESP	Spain	Olmedo		Va	41.1	-4.1	
	FUND. BIODIVER	60944	Unknown	Aegilops ovata subsp. triaristata	L.	ESP	Spain	El Cabaco		Sa	40.1	-5.1	
00:00.0	COFC	46895-1	Specimen	Aegilops geniculata Roth	Roth	ES	Spain	Posadas; tÃƒÂ©rmino municipal; pi		Co			1
	FUND. BIODIVER	1025032	Unknown	Aegilops ovata		ESP	Spain	E. Martos y Alcaudete		J	37.1	-3.1	
	FUND. BIODIVER	103038	Unknown	Aegilops geniculata Roth	Roth	ESP	Spain	BoÃ±ar, Rucayo		Le	42.1	-5.1	
	FUND. BIODIVER	1811876	Unknown	Aegilops geniculata Roth	Roth	ESP	Spain	Loma Negra		Na	41.1	-1.1	
00:00.0	BC	636311	Specimen	Aegilops geniculata Roth	Roth	ES	Spain	CollbatÃ³; Baix Llobregat: CollbatÃ³		B	41.568	1.831	340
	FUND. BIODIVER	1549491	Unknown	Aegilops ovata		ESP	Spain	regno Legion. (Villafranca del Bierzo		Le			
	FUND. BIODIVER	1565147	Unknown	Aegilops ovata		ESP	Spain	Hornillo de Cerrato		P	41.1	-4.1	850
	FUND. BIODIVER	1603055	Unknown	Aegilops ovata		ESP	Spain	la Lora		P			
	FUND. BIODIVER	1643499	Unknown	Aegilops geniculata Roth	Roth	ESP	Spain	PeÃ±aflor, Los Rasos		Z	41.1	-0.1	360
	FUND. BIODIVER	979130	Unknown	Aegilops ovata L.	L.	ESP	Spain	Blancas		Te	40.1	-1.1	
	FUND. BIODIVER	261211	Unknown	Aegylops ovata L.	L.	ESP	Spain	Mirca, carretera de Mirca		Tf	28.1	-17.1	150
00:00.0	REDIAM-CMA	28265	Observation	Aegilops geniculata		ESP	Spain		Pegalajar	J	37.7335	-3.53137	1916
00:00.0	REDIAM-CMA	38150	Observation	Aegilops geniculata		ESP	Spain		Cambil	J	37.7289	-3.48985	1594
00:00.0	REDIAM-CMA	47035	Observation	Aegilops geniculata		ESP	Spain		Almonaster la Re	H	37.8332	-6.76085	383
00:00.0	REDIAM-CMA	71144	Observation	Aegilops geniculata		ESP	Spain		AlcalÃ¡ de los Ga	Ca	36.4842	-5.75324	139
00:00.0	REDIAM-CMA	305945	Observation	Aegilops geniculata		ESP	Spain		Siles	J	38.3453	-2.50652	1248
00:00.0	REDIAM-CMA	364003	Observation	Aegilops geniculata		ESP	Spain		LubrÃn	Al	37.2026	-2.01598	600
00:00.0	REDIAM-CMA	382314	Observation	Aegilops geniculata		ESP	Spain				37.666	-3.30664	
00:00.0	REDIAM-CMA	383134	Observation	Aegilops geniculata		ESP	Spain		Casares	Ma	36.475	-5.29342	445
00:00.0	HUAL	5784-1	Specimen	Aegilops geniculata Roth	Roth	ES	Spain	Velefique; SÂª de Los Filabres, La C		Al	37.203	-2.397	
	SIVIM	R-P09690:Aegi	Observation	Aegilops geniculata Roth	Roth	ES	Spain	Riba de Santiuste			41.19	-2.76	1000
	SIVIM	R-P10531:Aegi	Observation	Aegilops geniculata Roth	Roth	ES	Spain	De AlcalÃ¡ de los Gazules a Benalup			36.28	-5.89	0
	SIVIM	R-P11661:Aegi	Observation	Aegilops geniculata Roth	Roth	ES	Spain	Tarifa			44.93	-6.04	0

	SIVIM	S-P01255:Aegi	Observation	Aegilops geniculata Roth	Roth	ES	Spain	Vinaixa, la Solana			41.44	0.84	0
	SIVIM	S-P03971:Aegi	Observation	Aegilops geniculata Roth	Roth	ES	Spain	Vinaixa, la Solana			41.44	0.84	0
	SIVIM	U-P08145:Aegi	Observation	Aegilops geniculata Roth	Roth	ES	Spain	Dehesa Boyal, LogrosÃ¡n			39.27	-5.55	0
	SIVIM	U-P13293:Aegi	Observation	Aegilops geniculata Roth	Roth	ES	Spain	En Ctra. Arjonilla-Marmolejo, Km. 5			37.94	-4.13	280
00:00.0	REDIAM-CMA	192420	Observation	Aegilops geniculata		ESP	Spain		Quesada	J	37.8604	-3.06141	714
00:00.0	REDIAM-CMA	202249	Observation	Aegilops geniculata		ESP	Spain		Obejo	Co	38.1193	-4.75416	455
00:00.0	REDIAM-CMA	229161	Observation	Aegilops geniculata		ESP	Spain		Almonaster la Re	H	37.8592	-6.89251	508
00:00.0	REDIAM-CMA	234436	Observation	Aegilops geniculata		ESP	Spain		Almonaster la Re	H	37.8931	-6.81165	703
00:00.0	COFC	52511-1	Specimen	Aegilops geniculata Roth	Roth	ES	Spain	Don Benito; carretera de La Haba		Ba	38	-5	300
00:00.0	COFC	46887-1	Specimen	Aegilops geniculata Roth	Roth	ES	Spain	ctra hacia Sevilla; finca 'Los Cabezo		Co			1
	SIVIM	T-P13665:Aegi	Observation	Aegilops geniculata Roth	Roth	ES	Spain	Mosqueruela			40.35	-0.52	1480
	SIVIM	T-P16884:Aegi	Observation	Aegilops geniculata Roth	Roth	ES	Spain	Majadahonda			40.46	-3.94	0
	SIVIM	T-P19060:Aegi	Observation	Aegilops geniculata Roth	Roth	ES	Spain	Base de Santa BÃ¡rbara			37.31	-2.88	1550
	SIVIM	T-P20230:Aegi	Observation	Aegilops geniculata Roth	Roth	ES	Spain	Proximidades de Baena			37.57	-4.35	0
	SIVIM	T-P26223:Aegi	Observation	Aegilops geniculata Roth	Roth	ES	Spain	Molinicos, Molinicos			38.39	-2.31	100
	SIVIM	T-P28615:Aegi	Observation	Aegilops geniculata Roth	Roth	ES	Spain	Villareal de San Carlos			39.71	-6.08	320
	FUND. BIODIVER	1713441	Unknown	Aegilops geniculata		ESP	Spain	Mallorca, Calvia, finca Ses Algorfes		PM	39.1	2.1	120
00:00.0	FUND. BIODIVER	1715689	Unknown	Aegilops geniculata Roth	Roth	ESP	Spain	Peraleda, prop a Mas Barrera		Ge	42.1	2.1	
	FUND. BIODIVER	1811867	Unknown	Aegilops geniculata		ESP	Spain	Milagro, carretera a Cadreita		Na	42.1	-1.1	
	FUND. BIODIVER	1834167	Unknown	Aegilops ovata L.	L.	ESP	Spain	Melilla, pinares de Rostrogordo		Me	35.1	-2.1	
00:00.0	UNEX	02110-1	Observation	Aegilops geniculata Roth	_	ESP	Spain	De Alcazaba a Trebolar.		Al	36.83	-2.45	
00:00.0	UNEX	28621-1	Observation	Aegilops geniculata Roth	_	ESP	Spain	Campanario: Cruz de Piedra Escrita		Ba	38.8	-5.6	
00:00.0	MA	682917-1	Specimen	Aegilops geniculata Roth	Roth	ES	Spain	PeÃ±a de Las Ventosa (CillÃ³rigo -		S			
00:00.0	GDA	GDA30009-1-1	Specimen	Aegilops ovata L.	L.	ES	Spain	MÃ¡laga, AxarquÃ-a.		MA			0
00:00.0	REDIAM-CMA	326234	Observation	Aegilops geniculata		ESP	Spain		Casares	Ma	36.4025	-5.30482	94
00:00.0	REDIAM-CMA	382830	Observation	Aegilops geniculata		ESP	Spain				37.6366	-3.1322	
00:00.0	LEB	10101-1	Specimen	Aegilops geniculata Roth	Roth	ES	Spain	Rucayo		Le	42.93	-5.27	1
	SIVIM	U-P09878:Aegi	Observation	Aegilops geniculata Roth	Roth	ES	Spain	Cuneta en'Casas Blancas'			38.84	-3	0
	GDA	GDA42773-1-1	Specimen	Aegilops geniculata Roth.	Roth.	ES	Spain	Granada, CÃºllar Baza, El Margen,		GR			872
00:00.0	MGC	62200-1	Unknown	Aegilops geniculata Roth	Roth	ES	Spain	Puebla de los Infantes (La); Al Este		Se	37.793	-5.357	200
	SANT	27752	Specimen	Aegilops geniculata Roth		ES	Spain	Garraf		B			
1876-06-1	W	42322	Unknown	Aegilops ovata L.		ESP	Spain	In ruderatia prope Aranjuez. In itinere hispanico-lustianico.					
00:00.0	COA	26638-1	Specimen	Aegilops geniculata Roth	Roth	ES	Spain	AlhaurÃ-n de la Torre, Arroyo del Ca		Ma	36.59	-4.56	
	ADIMAN	20	Observation	Aegilops geniculata		ESP	Spain	EnguÃ-danos		CU	39.6249	-1.60269	
	FUND. BIODIVER	80850	Unknown	Aegilops geniculata Roth	Roth	ESP	Spain	SepÃºlveda, SepÃºlveda, en el fond		Sg	41.1	-3.1	
	FUND. BIODIVER	860301	Unknown	Aegilops ovata L.	L.	ESP	Spain	Sierra de San JuliÃ¡n		A	38.1	-0.1	
00:00.0	REDIAM-CMA	78901	Observation	Aegilops geniculata		ESP	Spain		Santiago-Pontone	J	38.1163	-2.67872	1400
00:00.0	REDIAM-CMA	83941	Observation	Aegilops geniculata		ESP	Spain		Ronda	Ma	36.7218	-5.04831	1200
00:00.0	REDIAM-CMA	124008	Observation	Aegilops geniculata		ESP	Spain		Quesada	J	37.7643	-3.01848	900
00:00.0	COA	41221-1	Specimen	Aegilops geniculata Roth	Roth	ES	Spain	Km 17 de Cabra a Carcabuey		Co	37.4	-4.36	
	SIVIM	S-P10636:Aegi	Observation	Aegilops geniculata Roth	Roth	ES	Spain	Fuentealbilla hacia Abenjibre			39.2	-1.61	0

	SIVIM	S-P14655:Aegi	Observation	Aegilops geniculata Roth	Roth	ES	Spain	Rancho del Pino (P.R) ,			36.46	-5.9	0
	SIVIM	T-P03843:Aegi	Observation	Aegilops geniculata Roth	Roth	ES	Spain	3 km al NE de Uceda			40.83	-3.47	0
	SIVIM	T-P04206:Aegi	Observation	Aegilops geniculata Roth	Roth	ES	Spain	8 km al W de BaÃ±os de Fuensanto			37.67	-2.09	0
	SIVIM	T-P06419:Aegi	Observation	Aegilops geniculata Roth	Roth	ES	Spain	Bugarra			39.63	-0.78	350
00:00.0	SALA	83253-1	Specimen	Aegilops geniculata Roth	Roth	ES	Spain	_; Granada, Silla del Moro		Gr			
00:00.0	BDBCV	332918	Observation	Aegilops geniculata	_	ES	Spain		La Nucia	A	38.59	-0.07	
00:00.0	SEV	99214-1	Specimen	Aegilops geniculata Roth	Roth	ES	Spain	Sierra de AlbarracÃ-n		Te			1
00:00.0	COFC	11977-1	Specimen	Aegilops geniculata Roth	Roth	ES	Spain	Puente Genil; entre rÃƒÂ-o Anzur y		Co	37	-4	1
00:00.0	SEV	98934-1	Specimen	Aegilops geniculata Roth	Roth	ES	Spain	Almodovar del RÃ-o, PeÃ±a del Agu		Co			1
00:00.0	BDBCV	102	Observation	Aegilops geniculata		ESP	Spain	Parque Natural de Penyagolosa		Cs	40.19	-0.35	
00:00.0	BDBCV	121	Observation	Aegilops geniculata		ESP	Spain	Parque Natural de Penyagolosa		Cs	40.18	-0.4	
00:00.0	MGC	66244-1	Unknown	Aegilops geniculata Roth	Roth	ES	Spain	Colmenar; Cerro de la Caldera		Ma	36.944	-4.32	850
	ADIMAN	30	Observation	Aegilops geniculata		ESP	Spain	EnguÃ-danos		CU	39.66	-1.576	
	BDBCV-General	147997	Observation	Aegilops geniculata		ESPA	Spain	Puebla de San Mi	El RincÃ³n de Ade	Valenc	40.0484	-1.06579	
00:00.0	BDBCV	333608	Observation	Aegilops geniculata	_	ES	Spain		L'AlfÃ s del Pi	A	38.59	-0.07	
00:00.0	GDA	GDA48228-1-1	Specimen	Aegilops geniculata Roth	Roth	ES	Spain	Granada, SÂª de las Chanzas, Mon		GR			900
	ADIMAN	26	Observation	Aegilops geniculata		ESP	Spain	EnguÃ-danos		CU	39.6588	-1.57338	
	ADIMAN	29	Observation	Aegilops geniculata		ESP	Spain	EnguÃ-danos		CU	39.6597	-1.57496	
00:00.0	MGC	11104-1	Unknown	Aegilops geniculata Roth	Roth	ES	Spain	Nerja ; Sierra de Nerja. Abastecimie		Ma	36.7644	-3.83842	1
00:00.0	FUND. BIODIVER	1373118	Unknown	Aegilops geniculata Roth	Roth	ESP	Spain	Villapalacios, barranco del arroyo B		Ab	38.1	-2.1	900
	FUND. BIODIVER	1463965	Unknown	Aegilops geniculata Roth	Roth	ESP	Spain	Gerb		L	41.1	0.1	230
00:00.0	REDIAM-CMA	130525	Observation	Aegilops geniculata		ESP	Spain		Constantina	Se	37.9139	-5.71797	442
00:00.0	REDIAM-CMA	141832	Observation	Aegilops geniculata		ESP	Spain		Zufre	H	37.8786	-6.39067	323
00:00.0	REDIAM-CMA	170389	Observation	Aegilops geniculata		ESP	Spain		AndÃºjar	J	38.25	-4.08282	611
00:00.0	COFC	41296-1	Specimen	Aegilops geniculata Roth	Roth	ES	Spain	Puente Genil; Sierra del Castillo		Co	37	-4	1
	SIVIM	T-P06468:Aegi	Observation	Aegilops geniculata Roth	Roth	ES	Spain	Onteniente			38.82	-0.69	0
	SIVIM	T-P09144:Aegi	Observation	Aegilops geniculata Roth	Roth	ES	Spain	Mota del MarquÃ©s			41.61	-5.28	0
	SIVIM	T-P09348:Aegi	Observation	Aegilops geniculata Roth	Roth	ES	Spain	Torrelaguna			40.73	-3.59	0
	SIVIM	T-P10773:Aegi	Observation	Aegilops geniculata Roth	Roth	ES	Spain	Dehesa de Misleo (Moreruela de TÃ¡bara)			41.78	-5.88	0
	SIVIM	T-P11866:Aegi	Observation	Aegilops geniculata Roth	Roth	ES	Spain	Palanquinos			42.42	-5.55	0
	SIVIM	T-P13212:Aegi	Observation	Aegilops geniculata Roth	Roth	ES	Spain	Tirapu, hacia Artajona			42.62	-1.78	0
00:00.0	MA	618139-1	Specimen	Aegilops geniculata Roth	Roth	ES	Spain	Mallorca, Puigpunyent, subida hacia		Mll	39	2	
00:00.0	COA	8366-1	Specimen	Aegilops geniculata Roth	Roth	ES	Spain	Carcabuey, La Luca		Co	37.4	-4.36	
00:00.0	UNEX	10374-1	Observation	Aegilops geniculata Roth	_	ESP	Spain	GuareÃ±a: Torrefresneda. 29SQD2		Ba	39.3	-6.4	
00:00.0	SALA	16051-1	Specimen	Aegilops ovata L. subsp. triaristat	(Willd.) Rouy	ES	Spain	_; San Felices de los Gallegos		Sa			
00:00.0	COFC	36341-1	Specimen	Aegilops geniculata Roth	Roth	ES	Spain	Villanueva de Algaidas; cortijo de la		Ma			1
00:00.0	COFC	29841-1	Specimen	Aegilops geniculata Roth	Roth	ES	Spain	CardeÃƒÂ±a; Torrubia		Co			1
00:00.0	LEB	4787-1	Specimen	Aegilops geniculata Roth	Roth	ES	Spain	Pedrosa PrÃ-ncipe		Bu			1
	DEU146	AE 421	Specimen	Aegilops geniculata Roth subsp. gibberosa (Zh		ESP	Spain	Barcelona					
00:00.0	COFC	41150-1	Specimen	Aegilops geniculata Roth	Roth	ES	Spain	Santaella; arroyo de Sal		Co	37	-4	1
00:00.0	COFC	41284-1	Specimen	Aegilops geniculata Roth	Roth	ES	Spain	Puente Genil; Laguna Salada		Co	37	-4	1

00:00.0	MGC	43184-1	Unknown	Aegilops geniculata Roth	Roth	ES	Spain	MÃ¡laga; Campus de Teatinos		Ma	0	0	1
00:00.0	GDAC	GDAC39915-1	Specimen	Aegilops geniculata Roth.	Roth.	ES	Spain	Granada, SÂª de Castril, cercanÃ-a		GR			1140
	BC	70814	Specimen	Aegilops ovata L.	L.	ES	Spain	Arganda del Rey; Dehesa de Argan		M	40.33	-3.41	
	FUND. BIODIVER	1370928	Unknown	Aegilops geniculata Roth	Roth	ESP	Spain	RedueÃ±a		M	40.1	-3.1	
	FUND. BIODIVER	1463964	Unknown	Aegilops geniculata Roth	Roth	ESP	Spain	Penya Roja de Valldellou		L	41.1	0.1	420
00:00.0	MA	651157-1	Specimen	Aegilops geniculata Roth	Roth	ES	Spain	TÃ¡bara, La BreÃ±ica		Za	41	-5	
00:00.0	REDIAM-CMA	170195	Observation	Aegilops geniculata		ESP	Spain		AndÃºjar	J	38.2485	-4.08147	626
00:00.0	SEV	107985-1	Specimen	Aegilops geniculata Roth	Roth	ES	Spain	CÃ³rdoba. Colegio de La Aduana		Co			1
00:00.0	SEV	108251-1	Specimen	Aegilops geniculata Roth	Roth	ES	Spain	Lucena. Alrededores del Cortijo del		Co			1
	SIVIM	T-P06467:Aegi	Observation	Aegilops geniculata Roth	Roth	ES	Spain	Benisoda			38.82	-0.58	350
	SIVIM	T-P09143:Aegi	Observation	Aegilops geniculata Roth	Roth	ES	Spain	PeÃ±aflor de Hornija			41.71	-5.04	0
	SIVIM	T-P09347:Aegi	Observation	Aegilops geniculata Roth	Roth	ES	Spain	PontÃ³n de la Oliva			40.83	-3.47	0
	SIVIM	T-P10772:Aegi	Observation	Aegilops geniculata Roth	Roth	ES	Spain	Dehesa de Misleo (Moreruela de TÃ¡bara)			41.78	-5.88	0
	SIVIM	T-P11865:Aegi	Observation	Aegilops geniculata Roth	Roth	ES	Spain	Bracas			43.14	-5.58	0
	SIVIM	T-P13211:Aegi	Observation	Aegilops geniculata Roth	Roth	ES	Spain	UjuÃ©, Gallipienzo-Murillo del Fruto			43.25	-1.52	0
	IDBD-GN	42437	Observation	Aegilops geniculata Roth	Roth	ES	Spain		Olave	Na	42.8471	-1.5939	
	IDBD-GN	42447	Observation	Aegilops geniculata Roth	Roth	ES	Spain		Funes	Na	42.3227	-1.80589	
	ESP004	NC044547	Specimen	Aegilops geniculata Roth		ESP	Spain	Menorca, Turmadem, province of Baleares					
00:00.0	BDBCV	597129	Observation	Aegilops geniculata		ESP	Spain		Bocairent	Valenc	38.72	-0.556	
	FUND. BIODIVER	1120220	Unknown	Aegilops geniculata Roth	Roth	ESP	Spain	Fresno de la Ribera, La Cernia		Za	41.1	-5.1	
	FUND. BIODIVER	1135160	Unknown	Aegilops geniculata Roth	Roth	ESP	Spain	Castrofuerte		Le	42.1	-5.1	743
	FUND. BIODIVER	51166	Unknown	Aegilops ovata subsp. triaristata	L.	ESP	Spain	La Fregeneda		Sa	40.1	-6.1	
	FUND. BIODIVER	56041	Unknown	Aegilops geniculata Roth.	Roth.	ESP	Spain	Villaescusa de Roa, junto a Fuentec		Bu	41.1	-3.1	
00:00.0	BC	839941	Specimen	Aegilops geniculata Roth	Roth	ES	Spain	Jaca; Jaca, au confluent du rÃ-o Ga		Hu	42.558	-0.606	720
00:00.0	MGC	46463-1	Unknown	Aegilops geniculata Roth	Roth	ES	Spain	San JosÃ© del Valle; P. N. Los Alco		Ca	36.68	-5.591	350
00:00.0	REDIAM-CMA	35819	Observation	Aegilops geniculata		ESP	Spain		Cambil	J	37.6921	-3.51238	1052
00:00.0	REDIAM-CMA	44634	Observation	Aegilops geniculata		ESP	Spain		Huelma	J	37.7123	-3.46598	1645
00:00.0	REDIAM-CMA	50460	Observation	Aegilops geniculata		ESP	Spain		Torres	J	37.7505	-3.5302	1495
00:00.0	REDIAM-CMA	68379	Observation	Aegilops geniculata		ESP	Spain		Torres	J	37.774	-3.5736	964
00:00.0	REDIAM-CMA	76600	Observation	Aegilops geniculata		ESP	Spain		BÃ©lmez de la M	J	37.7513	-3.34852	770
	SIVIM	R-P09469:Aegi	Observation	Aegilops geniculata Roth	Roth	ES	Spain	Riba de Santiuste			41.19	-2.76	1000
	SIVIM	R-P10419:Aegi	Observation	Aegilops geniculata Roth	Roth	ES	Spain	K 11 de la carretera del Picacho , AG			36.47	-5.67	0
	SIVIM	R-P11646:Aegi	Observation	Aegilops geniculata Roth	Roth	ES	Spain	Tarifa			44.93	-6.04	0
	SIVIM	S-P01244:Aegi	Observation	Aegilops geniculata Roth	Roth	ES	Spain	l'Espluga Calba, afores del poble			41.44	0.96	0
	SIVIM	S-P02018:Aegi	Observation	Aegilops geniculata Roth	Roth	ES	Spain	GÃ rzola			41.89	0.95	0
	SIVIM	S-P03960:Aegi	Observation	Aegilops geniculata Roth	Roth	ES	Spain	els Omells de Na Gaia, afores del poble			41.44	0.96	0
00:00.0	SALA	112289-1	Specimen	Aegilops geniculata Roth	Roth	ES	Spain	Renedo de Esgueva		Va	41.64	-4.64	
00:00.0	GDA	GDA25366-1-1	Specimen	Aegilops geniculata Roth.	Roth.	ES	Spain	AlmerÃ-a, NÃ-jar, FernÃ¡n PÃ©rez.		AL			200
	IDBD-GN	42463	Observation	Aegilops geniculata Roth	Roth	ES	Spain		Marcilla	Na	42.3083	-1.7273	330
	IDBD-GN	42484	Observation	Aegilops geniculata Roth	Roth	ES	Spain		Sansol	Na	42.5697	-2.26417	480
	ESP004	NC044550	Specimen	Aegilops geniculata Roth		ESP	Spain	Pajares de la Laguna, province of Salamar			41.0833	-5.5	828

00:00.0	GDA	GDA13286-1	Specimen	Aegilops ovata L.	L.	ES	Spain	MÃ¡laga, CoÃ-n, la AlbuquerÃ-a.		MA			0
	SANT	5125	Specimen	Aegilops ovata L.		ES	Spain	Sierra Vizcuerno		Z			
	FUND. BIODIVER	1135157	Unknown	Aegilops geniculata Roth	Roth	ESP	Spain	Castrovega de Valmadrigal		Le	42.1	-5.1	
	FUND. BIODIVER	58155	Unknown	Aegilops ovata L.	L.	ESP	Spain	Babilafuente		Sa	40.1	-5.1	
	FUND. BIODIVER	62080	Unknown	Aegilops ovata subsp. ovata L.	L.	ESP	Spain	Cabrillas		Sa	40.1	-6.1	
	FUND. BIODIVER	1696524	Unknown	Aegilops geniculata Roth	Roth	ESP	Spain	Iglesuela del Cid, torre Nicasi		Te	40.1	-0.1	1500
	FUND. BIODIVER	1699512	Unknown	Aegilops geniculata		ESP	Spain	Mosqueruela		Te	40.1	-0.1	1480
	FUND. BIODIVER	1700450	Unknown	Aegilops geniculata		ESP	Spain	Puertomingalvo, El Letrado		Te	40.1	-0.1	1525
00:00.0	ABH	10631-1	Specimen	Aegilops geniculata Roth	Roth	ES	Spain	Villena; El Polovar		A	38.61	-0.9	
00:00.0	REDIAM-CMA	243815	Observation	Aegilops geniculata		ESP	Spain		AgrÃ³n	Gr	36.9991	-3.81397	1298
00:00.0	REDIAM-CMA	252352	Observation	Aegilops geniculata		ESP	Spain		Los BlÃ¡zquez	Co	38.4402	-5.40853	555
00:00.0	REDIAM-CMA	276158	Observation	Aegilops geniculata		ESP	Spain		Posadas	Co	37.8196	-5.16685	190
00:00.0	REDIAM-CMA	281713	Observation	Aegilops geniculata		ESP	Spain		BelalcÃ¡zar	Co	38.5038	-5.03497	525
00:00.0	REDIAM-CMA	283726	Observation	Aegilops geniculata		ESP	Spain		La Carolina	J	38.295	-3.59662	600
	REDIAM-CMA	34888	Observation	Aegilops geniculata		ESP	Spain		San JosÃ© del Va	Ca	36.6651	-5.60996	200
00:00.0	REDIAM-CMA	49723	Observation	Aegilops geniculata		ESP	Spain		Torres	J	37.7472	-3.52847	1500
00:00.0	REDIAM-CMA	57471	Observation	Aegilops geniculata		ESP	Spain		El Castillo de las	Se	37.6572	-6.24441	233
00:00.0	REDIAM-CMA	66315	Observation	Aegilops geniculata		ESP	Spain		Arroyo del Ojanco	J	38.3326	-2.84543	872
00:00.0	MGC	29529-1	Unknown	Aegilops geniculata Roth	Roth	ES	Spain	Ubrique; El Saltadero, Sierra Baja		Ca	36.675	-5.423	1000
00:00.0	MGC	34463-1	Unknown	Aegilops geniculata Roth	Roth	ES	Spain	Parauta; Cortijo Quejigales		Ma	36.71	-5.07	1300
	SIVIM	T-P30049:Aegi	Observation	Aegilops geniculata Roth	Roth	ES	Spain	Pr. Arenal de Las Salinas, Casas de Ves, A			39.28	-1.37	0
	SIVIM	T-P30116:Aegi	Observation	Aegilops geniculata Roth	Roth	ES	Spain	El Campichuelo, Cofrentes, Valencia			39.19	-1.14	0
	SIVIM	U-P02611:Aegi	Observation	Aegilops geniculata Roth	Roth	ES	Spain	Castells de Serrella			38.72	-0.23	0
	SIVIM	U-P06715:Aegi	Observation	Aegilops geniculata Roth	Roth	ES	Spain	Moncarapacho			37.04	-7.87	140
	SIVIM	U-P08125:Aegi	Observation	Aegilops geniculata Roth	Roth	ES	Spain	Berzocana			39.36	-5.55	0
	SIVIM	R-P09418:Aegi	Observation	Aegilops geniculata Roth	Roth	ES	Spain	Bochones			41.19	-2.88	1180
	SIVIM	R-P10354:Aegi	Observation	Aegilops geniculata Roth	Roth	ES	Spain	Salida desde AlcalÃ¡ de los Gazules hacia			36.38	-5.78	1
	SIVIM	R-P11639:Aegi	Observation	Aegilops geniculata Roth	Roth	ES	Spain	AlcalÃ¡ de los Gazules, vega del Pradillo			36.38	-5.78	0
	SIVIM	S-P01240:Aegi	Observation	Aegilops geniculata Roth	Roth	ES	Spain	l'Albi			41.35	0.84	0
	SIVIM	S-P01989:Aegi	Observation	Aegilops geniculata Roth	Roth	ES	Spain	Vilanova de MeiÃ , marge de la ctra. que d			41.98	0.94	0
	SIVIM	S-P03956:Aegi	Observation	Aegilops geniculata Roth	Roth	ES	Spain	Vinaixa, carretera de l'Albi			41.35	0.84	0
	ESP004	NC022350	Specimen	Aegilops geniculata Roth		ESP	Spain	Medina Azahara, Cordoba, province of Cor			37.8833	-4.86667	219
	MUB	102372-1	Specimen	Aegilops geniculata Roth	Roth	ES	Spain	La Union; Puerto de Portman, Portm		Mu	37.59	-0.854	2
00:00.0	HUAL	1133-1	Specimen	Aegilops geniculata Roth	Roth	ES	Spain	SÂª del Pozo, prÃ³x. Nava de San P		J	37.889	-2.881	
00:00.0	COFC	11983-1	Specimen	Aegilops geniculata Roth	Roth	ES	Spain	Rute; Sierra de Rute		Co	37	-4	1
00:00.0	COFC	16198-1	Specimen	Aegilops geniculata Roth	Roth	ES	Spain	Hinojosa del Duque; rÃƒÂ-o ZÃƒÂºj		Co	38	-5	1
00:00.0	ABH	23284-1	Specimen	Aegilops geniculata Roth	Roth	ES	Spain	Villena; Cabezo de las Lechuzas		A	38.56	-0.93	
	FUND. BIODIVER	1335998	Unknown	Aegilops geniculata Roth	Roth	ESP	Spain	Lumbier, foz del rÃ-o Irati		Na	42.1	-1.1	450
	FUND. BIODIVER	70822	Unknown	Aegilops geniculata Roth	Roth	ESP	Spain	Cubillo		Sg	40.1	-3.1	
	FUND. BIODIVER	82305	Unknown	Aegilops geniculata Roth.	Roth.	ESP	Spain	Cedillo de la Torre		Sg	41.1	-3.1	
00:00.0	MGC	67086-1	Unknown	Aegilops ovata L.	L.	ES	Spain	Ciudad Universitaria		M	0	0	1

00:00.0	MGC	7598-1	Unknown	Aegilops ovata L.	L.	ES	Spain	San Roque; Punta Mala		Ca	0	0	1
00:00.0	REDIAM-CMA	79804	Observation	Aegilops geniculata		ESP	Spain		Villarrodrigo	J	38.5055	-2.7314	766
00:00.0	REDIAM-CMA	93948	Observation	Aegilops geniculata		ESP	Spain		Almonaster la Re	H	37.7836	-6.70527	382
	SIVIM	S-P09770:Aegi	Observation	Aegilops geniculata Roth	Roth	ES	Spain	guixos del Captivador (la Nucia)			38.54	-0.13	120
	SIVIM	S-P13990:Aegi	Observation	Aegilops geniculata Roth	Roth	ES	Spain	Pinhal da Senhora (Freixo do Meio)			39.38	-8.88	144
	SIVIM	T-P01738:Aegi	Observation	Aegilops geniculata Roth	Roth	ES	Spain	Mallorca: Lluc, prop de Son Llovera (fons d			39.74	2.76	0
	SIVIM	T-P03847:Aegi	Observation	Aegilops geniculata Roth	Roth	ES	Spain	3 km al SE de Fuentecambron			41.46	-3.35	1030
	SIVIM	T-P04227:Aegi	Observation	Aegilops geniculata Roth	Roth	ES	Spain	1 km al E de Mora de Santiago			38.57	-1.62	0
	SIVIM	T-P06423:Aegi	Observation	Aegilops geniculata Roth	Roth	ES	Spain	Ayelo de Malferit			38.82	-0.58	350
00:00.0	GDA	GDA16011-1	Specimen	Aegilops geniculata Roth	Roth	ES	Spain	Granada, SÂª de Madrid, Lagunazo		GR			1200
00:00.0	ABH	23714-1	Specimen	Aegilops geniculata Roth	Roth	ES	Spain	Bollullos de la MitaciÃ³n;		Se	37.28	-6.18	
	ADIMAN	28	Observation	Aegilops geniculata		ESP	Spain	EnguÃ-danos		CU	39.6594	-1.57509	
	IDBD-GN	42431	Observation	Aegilops geniculata Roth	Roth	ES	Spain	Loma Negra	Bardenas Reales	Na	42.1236	-1.36807	
	ESP004	NC043860	Specimen	Aegilops geniculata Roth		ESP	Spain	Alcala de Henares, province of Madrid			40.4667	-3.36667	587
	DEU146	AE 485	Specimen	Aegilops geniculata Roth		ESP	Spain	Nocito Espagne					
00:00.0	UNEX	10350-1	Observation	Aegilops geniculata Roth	_	ESP	Spain	Fuente del Maestre: Alrededores. 2		Ba	38.5	-6.5	
00:00.0	FUND. BIODIVER	1972308	Unknown	Aegilops geniculata		ESP	Spain	Valle de Escuain, Puertolas, refugio		Hu	42.1	0.9	2020
	FUND. BIODIVER	56188	Unknown	Aegilops ovata L.	L.	ESP	Spain	Aguasal		Va	41.1	-4.1	
	FUND. BIODIVER	59599	Unknown	Aegilops geniculata Roth	Roth	ESP	Spain	Monterrubio de ArmuÃ±a		Sa	40.1	-5.1	
00:00.0	BDBCV	589927	Observation	Aegilops geniculata		ESP	Spain		Alcoy	Alicant	38.675	-0.58	
00:00.0	COFC	46885-1	Specimen	Aegilops geniculata Roth	Roth	ES	Spain	Palma del RÃƒÂ-o; rÃƒÂ-o Guadalc		Co			1
	FUND. BIODIVER	1024006	Unknown	Aegilops ovata L.	L.	ESP	Spain	Valdeazores		J	38.1	-3.1	
	FUND. BIODIVER	1028170	Unknown	Aegilops geniculata Roth.	Roth.	ESP	Spain	ValdepeÃ±as de JaÃ©n, carretera a		J	37.1	-3.1	
	FUND. BIODIVER	1715687	Unknown	Aegilops geniculata		ESP	Spain	Ventallo, entre el puig d'Estragues i		Ge	41.1	2.1	
	FUND. BIODIVER	1762689	Unknown	Aegilops geniculata		ESP	Spain	Volcan de Piedrabuena		CR	38.1	-4.1	
	FUND. BIODIVER	1811865	Unknown	Aegilops geniculata		ESP	Spain	Vedado de Eguaras		Na	42.1	-1.1	480
	FUND. BIODIVER	183144	Unknown	Aegilops ovata L.	L.	ESP	Spain			Cs			
00:00.0	SALA	27689-1	Specimen	Aegilops ovata L.	L.	ES	Spain	_; Cartagena, San Felix		Mu			
00:00.0	REDIAM-CMA	27082	Observation	Aegilops geniculata		ESP	Spain		Algodonales	Ca	36.9169	-5.40203	591
00:00.0	REDIAM-CMA	37456	Observation	Aegilops geniculata		ESP	Spain		Cambil	J	37.7287	-3.49122	1581
00:00.0	REDIAM-CMA	293889	Observation	Aegilops geniculata		ESP	Spain		La Puerta de Seg	J	38.3846	-2.80506	687
00:00.0	REDIAM-CMA	325611	Observation	Aegilops geniculata		ESP	Spain		Casares	Ma	36.3965	-5.26232	66
00:00.0	REDIAM-CMA	339068	Observation	Aegilops geniculata		ESP	Spain		Antequera	Ma	36.9731	-4.77276	371
00:00.0	REDIAM-CMA	381746	Observation	Aegilops geniculata		ESP	Spain				37.8442	-3.40138	
00:00.0	REDIAM-CMA	382814	Observation	Aegilops geniculata		ESP	Spain		Olvera	Ca	36.9272	-5.36328	390
00:00.0	GDA	GDA25152-1-1	Specimen	Aegilops geniculata Roth.	Roth.	ES	Spain	Granada, Pedro MartÃ-nez, Mencal		GR			1150
	SIVIM	R-P09635:Aegi	Observation	Aegilops geniculata Roth	Roth	ES	Spain	entre SigÃ¼enza y Pelegrina			41.01	-2.64	1100
	SIVIM	R-P10459:Aegi	Observation	Aegilops geniculata Roth	Roth	ES	Spain	Ermita de los Santos , AG			36.38	-5.78	0
	SIVIM	R-P10461:Aegi	Observation	Aegilops geniculata Roth	Roth	ES	Spain	Rancho del Pino , PR			36.46	-5.9	0
	SIVIM	R-P11657:Aegi	Observation	Aegilops geniculata Roth	Roth	ES	Spain	Tarifa			44.93	-6.04	0
	SIVIM	S-P01250:Aegi	Observation	Aegilops geniculata Roth	Roth	ES	Spain	l'Espluga Calba, Pla de la Creu			41.44	0.96	0

	SIVIM	S-P02153:Aegi	Observation	Aegilops geniculata Roth	Roth	ES	Spain	Mont-roig, al vessant sud			41.8	0.83	0
	SIVIM	S-P02166:Aegi	Observation	Aegilops geniculata Roth	Roth	ES	Spain	Pla de les Gesses (Ivars de Noguera)			41.79	0.47	320
	SIVIM	S-P03966:Aegi	Observation	Aegilops geniculata Roth	Roth	ES	Spain	MaldÃ , a VilamajÃ³			41.53	0.96	0
	SIVIM	U-P09293:Aegi	Observation	Aegilops geniculata Roth	Roth	ES	Spain	Orea, borde de pista forestal			40.55	-1.81	1500
	IDBD-GN	42460	Observation	Aegilops geniculata Roth	Roth	ES	Spain	Iranzu	AbÃ¡rzuza	Na	42.762	-2.08468	
	IDBD-GN	42473	Observation	Aegilops geniculata Roth	Roth	ES	Spain	La Sarda	Liedena	Na	42.6215	-1.23928	600
	IDBD-GN	42481	Observation	Aegilops geniculata Roth	Roth	ES	Spain	Estrib.sierra IllÃ³n	NavascuÃ©s	Na	42.7084	-1.04145	1100
	IDBD-GN	42494	Observation	Aegilops geniculata Roth	Roth	ES	Spain	LezÃ¡un	Estella	Na	42.7658	-2.00518	830
00:00.0	SEV	108082-1	Specimen	Aegilops geniculata Roth	Roth	ES	Spain	SerranÃ-a de Ronda. Sierra de las		Ma			900
00:00.0	SEV	108254-1	Specimen	Aegilops geniculata Roth	Roth	ES	Spain	Benavente. Salida hacia Madrid. Ce		Za			1
00:00.0	GDA	GDA10273-1	Specimen	Aegilops geniculata Roth.	Roth.	ES	Spain	Granada, Ãzbor, camino de LanjarÃ		GR			600
00:00.0	ABH	2366-1	Specimen	Aegilops geniculata Roth	Roth	ES	Spain	XÃ bia; Muntanyar de Baix		A	38.77	0.18	
00:00.0	ABH	34379-1	Specimen	Aegilops geniculata Roth	Roth	ES	Spain	Alicante; Fontcalent, yesos		A	38.36	-0.61	
00:00.0	COFC	38700-1	Specimen	Aegilops ovata L.	L.	ES	Spain	ctra. de Santo Domingo		Co			1
00:00.0	FUND. BIODIVER	1578713	Unknown	Aegilops geniculata		ESP	Spain	Ruesga		P	42.1	-4.1	
	FUND. BIODIVER	1618204	Unknown	Aegilops ovata L.	L.	ESP	Spain	Mora de Luna		Le	42.1	-5.1	
	FUND. BIODIVER	1643503	Unknown	Aegilops geniculata Roth	Roth	ESP	Spain	Mediana, Llanos de la Salada		Z	41.1	-0.1	370
	FUND. BIODIVER	993534	Unknown	Aegilops geniculata		ESP	Spain	Casar de Talamanca		Gu	40.1	-3.1	
	FUND. BIODIVER	1481198	Unknown	Aegilops geniculata Roth	Roth	ESP	Spain	P.N. ArchipiÃ©lago de Cabrera		PM	38.1	2.1	
	FUND. BIODIVER	1554580	Unknown	Aegilops ovata L.	L.	ESP	Spain	Cervera		P	42.1	-4.1	
	FUND. BIODIVER	995300	Unknown	Aegilops ovata L.	L.	ESP	Spain	Almonacid del Marquesado y Villare		Cu	39.1	-2.1	
	FUND. BIODIVER	1001978	Unknown	Aegylops geniculata		ESP	Spain	CaÃ±ete a Boniches		Cu	39.1	-1.1	
00:00.0	REDIAM-CMA	217415	Observation	Aegilops geniculata		ESP	Spain		Chimeneas	Gr	37.1373	-3.90014	724
00:00.0	REDIAM-CMA	230462	Observation	Aegilops geniculata		ESP	Spain		Padules	Al	36.9194	-2.79756	1706
00:00.0	REDIAM-CMA	235102	Observation	Aegilops geniculata		ESP	Spain		El Cerro de AndÃ	H	37.7467	-7.05958	241
00:00.0	GDAC	GDAC33714-1	Specimen	Aegilops geniculata Roth.	Roth.	ES	Spain	Granada, Loja, SÂª de Loja, base d		GR			800
00:00.0	COFC	52513-1	Specimen	Aegilops geniculata Roth	Roth	ES	Spain	Motril; cabo de Sacratif		Gr			1
00:00.0	BC	634093	Specimen	Aegilops geniculata Roth	Roth	ES	Spain	Pineda de Mar; Pineda		B	41.5512	2.640455	
00:00.0	BC	852007	Specimen	Aegilops ovata L.	L.	ES	Spain	Santa EulÃ lia de RonÃ§ana; Sta E		B	41.653	2.213	
00:00.0	BC	859865	Specimen	Aegilops ovata L.	L.	ES	Spain	Riner; SOLSONÃˆS - Riner: El Mira		L	41.95	1.49	
	SIVIM	T-P13698:Aegi	Observation	Aegilops geniculata Roth	Roth	ES	Spain	Torre de Nicasi, Iglesuela del Cid			40.43	-0.28	1140
	SIVIM	T-P16888:Aegi	Observation	Aegilops geniculata Roth	Roth	ES	Spain	Entre Casar de Talamanca y Mesones			40.64	-3.47	0
	SIVIM	T-P19655:Aegi	Observation	Aegilops geniculata Roth	Roth	ES	Spain	Camino de Los Toros (AlmodÃ³var)			37.83	-5.04	130
	SIVIM	T-P21023:Aegi	Observation	Aegilops geniculata Roth	Roth	ES	Spain	Puerto Viejo, Los Villares			37.58	-3.9	1550
	SIVIM	T-P28633:Aegi	Observation	Aegilops geniculata Roth	Roth	ES	Spain	Castillo de Mirabel			39.8	-6.31	0
00:00.0	REDIAM-CMA	189400	Observation	Aegilops geniculata		ESP	Spain		Tabernas	Al	37.0846	-2.33597	480
00:00.0	REDIAM-CMA	221175	Observation	Aegilops geniculata		ESP	Spain		AlbuÃ±uelas	Gr	36.8802	-3.75077	1299
00:00.0	REDIAM-CMA	236255	Observation	Aegilops geniculata		ESP	Spain		Castril	Gr	37.8532	-2.78067	1244
	SIVIM	T-P13704:Aegi	Observation	Aegilops geniculata Roth	Roth	ES	Spain	Inicio pista del Letrado, Puertomingalvo			40.26	-0.53	1500
	SIVIM	T-P16911:Aegi	Observation	Aegilops geniculata Roth	Roth	ES	Spain	TamajÃ³n			40.92	-3.35	0
	SIVIM	T-P19968:Aegi	Observation	Aegilops geniculata Roth	Roth	ES	Spain	Finca de Valdelasyeguas (Aliseda)			39.36	-6.79	0

	SIVIM	T-P25001:Aegi	Observation	Aegilops geniculata Roth	Roth	ES	Spain	Sant Antoni Abad			38.92	1.26	0
	SIVIM	T-P27404:Aegi	Observation	Aegilops geniculata Roth	Roth	ES	Spain	Villalgordo del Marquesado			39.65	-2.53	0
	SIVIM	T-P28639:Aegi	Observation	Aegilops geniculata Roth	Roth	ES	Spain	Cancho del Guarro, TorrejÃ³n el Rubio			39.71	-6.08	350
	FUND. BIODIVER	1370918	Unknown	Aegilops geniculata Roth	Roth	ESP	Spain	Soto del Real		M	40.1	-3.1	
	FUND. BIODIVER	1463981	Unknown	Aegilops geniculata Roth	Roth	ESP	Spain	Puig de Sant Ermengol		L	41.1	0.1	460
00:00.0	COFC	36343-1	Specimen	Aegilops geniculata Roth	Roth	ES	Spain	Palma del RÃƒÂ-o; arroyo Mahoma		Co			1
00:00.0	MGC	62675-1	Unknown	Aegilops geniculata Roth	Roth	ES	Spain	Villalba del Alcor; Camino de las Mi		H	37.458	-6.518	153
00:00.0	REDIAM-CMA	146546	Observation	Aegilops geniculata		ESP	Spain		CanjÃ¡yar	Al	36.9854	-2.73454	601
00:00.0	HSS	2694	Specimen	Aegilops geniculata Roth	Roth	ES	Spain	VillagarcÃ-a de la Torre		Ba	38.3169	-6.0836	530
00:00.0	SALA	14385-1	Specimen	Aegilops ovata L. subsp. ovata	L.	ES	Spain	_; Cabrillas		Sa			
00:00.0	COFC	41152-1	Specimen	Aegilops geniculata Roth	Roth	ES	Spain	Cabra; Salinas de Arias; Fuente de		Co	37	-4	1
00:00.0	COFC	41290-1	Specimen	Aegilops geniculata Roth	Roth	ES	Spain	Cabra; entre la vÃƒÂ-a del tren y la		Co	37	-4	1
	SIVIM	T-P06444:Aegi	Observation	Aegilops geniculata Roth	Roth	ES	Spain	Requena			39.46	-1.14	0
	SIVIM	T-P07218:Aegi	Observation	Aegilops geniculata Roth	Roth	ES	Spain	Litera			42.06	0.58	0
	SIVIM	T-P09204:Aegi	Observation	Aegilops geniculata Roth	Roth	ES	Spain	Mojados			41.35	-4.67	0
	SIVIM	T-P10144:Aegi	Observation	Aegilops geniculata Roth	Roth	ES	Spain	K12 Carretera del Picacho, AlcalÃ¡ de los (			36.38	-5.78	0
	SIVIM	T-P11411:Aegi	Observation	Aegilops geniculata Roth	Roth	ES	Spain	CaÃ±ete			40.01	-1.71	1130
	SIVIM	T-P13179:Aegi	Observation	Aegilops geniculata Roth	Roth	ES	Spain	De Genevilla a Aguilar de CodÃ©s			42.54	-2.51	0
00:00.0	LEB	4785-1	Specimen	Aegilops geniculata Roth	Roth	ES	Spain	Monte Grande. Valencia de Don Jua		Le	42.2	-5.48	1
00:00.0	W	42352	Unknown	Aegilops ovata L.		ESP	Spain	Montejo on la Sierra (Madrid).					
00:00.0	SEV	99075-1	Specimen	Aegilops geniculata Roth	Roth	ES	Spain	Entre MorÃ³n de la Frontera y Villan		Se			1
	FUND. BIODIVER	1463966	Unknown	Aegilops geniculata Roth	Roth	ESP	Spain	La Garriga de Gerb		L	41.1	0.1	350
	SANT	55884	Specimen	Aegilops geniculata Roth		ES	Spain	Sevilla, ctra. Utrera Km 1, Campus		Se			
00:00.0	REDIAM-CMA	155622	Observation	Aegilops geniculata		ESP	Spain		Hornos	J	38.1825	-2.71894	945
	SIVIM	T-P06961:Aegi	Observation	Aegilops geniculata Roth	Roth	ES	Spain	ArdÃ³n			42.42	-5.67	0
	SIVIM	T-P09145:Aegi	Observation	Aegilops geniculata Roth	Roth	ES	Spain	Iscar			41.45	-4.55	0
	SIVIM	T-P09349:Aegi	Observation	Aegilops geniculata Roth	Roth	ES	Spain	ValdepeÃ±as de la Sierra			40.83	-3.47	0
	SIVIM	T-P10774:Aegi	Observation	Aegilops geniculata Roth	Roth	ES	Spain	Faramontanos de TÃ¡bara			41.78	-5.88	0
	SIVIM	T-P11867:Aegi	Observation	Aegilops geniculata Roth	Roth	ES	Spain	Ardoncino			42.42	-5.67	0
	SIVIM	T-P13213:Aegi	Observation	Aegilops geniculata Roth	Roth	ES	Spain	Agorreta, pto. Erro			42.89	-1.53	0
00:00.0	UNEX	05898-1	Observation	Aegilops geniculata Roth	_	ESP	Spain	La Haba: La Haba, depÃ³sitos mioc		Ba	38.9	-5.9	
00:00.0	GDA	GDA30010-1-2	Specimen	Aegilops geniculata Roth	Roth	ES	Spain	JaÃ©n, SÂª MÃ¡gina, MojÃ³n Blanc		J			1400
	ADIMAN	37	Observation	Aegilops geniculata		ESP	Spain	EnguÃ-danos		CU	39.6827	-1.60778	
	ADIMAN	38	Observation	Aegilops geniculata		ESP	Spain	EnguÃ-danos		CU	39.6848	-1.66702	
	RUS001	VIR100602208	Specimen	Aegilops ovata L.		ESP	Spain						
	FUND. BIODIVER	1043763	Unknown	Aegilops geniculata Roth	Roth	ESP	Spain	Sierra Tejeda		Ma	36.1	-3.1	
00:00.0	FUND. BIODIVER	1835071	Unknown	Aegilops geniculata Roth	Roth	ESP	Spain	Herencia, cerro Navajo, ladera SE		CR	39.1	-3.1	660
	FUND. BIODIVER	1891039	Unknown	Aegilops geniculata Roth	Roth	ESP	Spain	Formentera		PM	38.1	1.1	
00:00.0	GDAC	GDAC36990-1	Specimen	Aegilops geniculata Roth.	Roth.	ES	Spain	Granada, Iznalloz, prox. Cortijo de D		GR			950
00:00.0	REDIAM-CMA	384045	Observation	Aegilops geniculata		ESP	Spain		Coripe	Se	36.9818	-5.37872	300
00:00.0	REDIAM-CMA	385335	Observation	Aegilops geniculata		ESP	Spain		Pegalajar	J	37.7331	-3.53242	1893

00:00.0	REDIAM-CMA	386693	Observation	Aegilops geniculata		ESP	Spain		AlgÃ¡mitas	Se	37.0218	-5.1761	662
00:00.0	REDIAM-CMA	390793	Observation	Aegilops geniculata		ESP	Spain		Ronda	Ma	36.7058	-5.03536	1205
	SIVIM	P-P08512:Aegi	Observation	Aegilops geniculata Roth	Roth	ES	Spain	Font del Retaule; la SÃ©nia			40.7	0.15	1030
	SIVIM	P-P08877:Aegi	Observation	Aegilops geniculata Roth	Roth	ES	Spain	Boixet; la SÃ©nia			40.7	0.15	1220
	SIVIM	P-P09005:Aegi	Observation	Aegilops geniculata Roth	Roth	ES	Spain	Boixet; la SÃ©nia			40.7	0.15	1240
	SIVIM	P-P11089:Aegi	Observation	Aegilops geniculata Roth	Roth	ES	Spain	Les Tosses, Sant Joan del Pas			40.53	0.4	220
	SIVIM	P-P12762:Aegi	Observation	Aegilops geniculata Roth	Roth	ES	Spain	Chirivel			37.58	-2.32	1040
00:00.0	BC	113136	Specimen	Aegilops ovata L.	L.	ES	Spain	Renau; Renau		T	41.22	1.27	
00:00.0	GDA	GDA10274-1	Specimen	Aegilops geniculata Roth. var. pu	Roth.	ES	Spain	Granada, SÂª de Mecina, Pitres, fal		GR			1150
	IPK	31888	Living	Aegilops geniculata Roth subsp. gibberosa (Zh		ESP	Spain						
	IPK	77875	Living	Aegilops geniculata Roth		ESP	Spain	Gelande von El Encin bei Alcala de Henares					
	SEV	5988-1	Specimen	Aegilops ovata L.	L.	ES	Spain	CarabaÃ±a		M			1
00:00.0	HUAL	1131-1	Specimen	Aegilops geniculata Roth	Roth	ES	Spain	SÂª de Cazorla, Puerto de Las Palo		J	37.952	-2.937	
	ESP004	NC050487	Specimen	Aegilops geniculata Roth		ESP	Spain	casa forestal de Tejadillos, Cuenca, provin			40.4	-1.98333	1080
	ESP004	NC044548	Specimen	Aegilops geniculata Roth		ESP	Spain	Parelleta, Ciutatdella de Menorca, province of Baleares					
	BDBCV-General	77391	Observation	Aegilops geniculata		ESPA	Spain	SacaÃ±et	El Alto Palancia	Castel	39.862	-0.72035	
00:00.0	COFC	11972-1	Specimen	Aegilops geniculata Roth	Roth	ES	Spain	Priego de CÃƒÂ³rdoba; Sierra de la		Co	37	-4	1
00:00.0	BDBCV	597283	Observation	Aegilops geniculata		ESP	Spain		Bocairent	Valenc	38.72	-0.567	
	FUND. BIODIVER	1120219	Unknown	Aegilops geniculata Roth	Roth	ESP	Spain	Abezames, Gafos		Za	41.1	-5.1	
	FUND. BIODIVER	1135159	Unknown	Aegilops geniculata Roth	Roth	ESP	Spain	ArdÃ³n		Le	42.1	-5.1	820
	FUND. BIODIVER	51165	Unknown	Aegilops ovata subsp. triaristata	L.	ESP	Spain	San Felices de los Gallegos		Sa	40.1	-6.1	
	FUND. BIODIVER	56040	Unknown	Aegilops geniculata Roth.	Roth.	ESP	Spain	Encinas de Esgueva, La Revuelta, r		Va	41.1	-3.1	
	FUND. BIODIVER	59183	Unknown	Aegilops geniculata Roth	Roth	ESP	Spain	Pelabravo		Sa	40.1	-5.1	
	FUND. BIODIVER	65367	Unknown	Aegilops geniculata Roth.	Roth.	ESP	Spain	Guijuelo		Sa	40.1	-5.1	
00:00.0	GDA	GDA16011-1-2	Specimen	Aegilops geniculata Roth	Roth	ES	Spain	Granada, SÂª de Madrid, Lagunazo		GR			1200
00:00.0	MA	657097-1	Specimen	Aegilops geniculata Roth.	Roth.	ES	Spain	Segovia		Sg	40	-4	
	GDA	GDA30012-1-2	Specimen	Aegilops geniculata Roth.	Roth.	ES	Spain	Madrid, Arganda.		M			0
	IDBD-GN	42458	Observation	Aegilops geniculata Roth	Roth	ES	Spain	Foz	Lumbier	Na	42.6638	-1.354	5250
	IDBD-GN	42502	Observation	Aegilops geniculata Roth	Roth	ES	Spain	PeÃ±a Izaga	Izagaonda	Na	42.7144	-1.43204	1200
00:00.0	REDIAM-CMA	68137	Observation	Aegilops geniculata		ESP	Spain		Puente de GÃ©na	J	38.3347	-2.78705	722
00:00.0	REDIAM-CMA	75935	Observation	Aegilops geniculata		ESP	Spain		BÃ©lmez de la M	J	37.7198	-3.39622	1059
00:00.0	HSS	2654	Specimen	Aegilops geniculata Roth	Roth	ES	Spain	Solana de los Barros		Ba	38.6875	-6.52841	
	SIVIM	R-P09421:Aegi	Observation	Aegilops geniculata Roth	Roth	ES	Spain	Valdelcubo			41.19	-2.76	1010
	SIVIM	R-P10418:Aegi	Observation	Aegilops geniculata Roth	Roth	ES	Spain	K 10 de la carretera del Picacho , AG			36.47	-5.67	0
	SIVIM	R-P11643:Aegi	Observation	Aegilops geniculata Roth	Roth	ES	Spain	AlcalÃ¡ de los Gazules			36.38	-5.78	1
	SIVIM	S-P01242:Aegi	Observation	Aegilops geniculata Roth	Roth	ES	Spain	l'Espluga Calba, els Bassals			41.44	0.96	0
	SIVIM	S-P02017:Aegi	Observation	Aegilops geniculata Roth	Roth	ES	Spain	Serra de MonterÃ³			41.8	0.83	0
	SIVIM	S-P03959:Aegi	Observation	Aegilops geniculata Roth	Roth	ES	Spain	l'Espluga Calba, afores del poble			41.44	0.96	0
00:00.0	BC	112335	Specimen	Aegilops ovata L.	L.	ES	Spain	Soria; Soria. Laderas degradadas d		So	41.78	-2.46	
00:00.0	W	41844	Unknown	Aegilops geniculata Roth		ESP	Spain	Prov. Jaen: S von Jaen; im Rio des Campillo-Tal, bei Cazalla, ar					750
	BDBCV-General	277599	Observation	Aegilops geniculata		ESPA	Spain	Enguera	La Canal de Nava	Valenc	39.0517	-0.74664	

	BDBCV-General	279878	Observation	Aegilops geniculata		ESPA	Spain	Cortes de PallÃ¡s	El Valle de Ayora	Valenc	39.236	-0.97245	
00:00.0	GDA	GDA30015-1-2	Specimen	Aegilops geniculata Roth.	Roth.	ES	Spain	Salamanca, Monte de la Orbada.		SA			0
00:00.0	JBAG	1918-1	Specimen	Aegilops geniculata Roth	Roth	ES	Spain	Cillorigo de LiÃƒÂ©bana; CabaÃƒÂ		S	43.22	-4.62	600
00:00.0	SALA	21957-1	Specimen	Aegilops geniculata Roth	Roth	ES	Spain	_; San CebriÃ¡n de Mazote		Va			
00:00.0	GDA	GDA30017-1	Specimen	Aegilops ovata L.	L.	ES	Spain	Granada, Dehesa de Montejo.		GR			0
	FUND. BIODIVER	1674401	Unknown	Aegilops geniculata		ESP	Spain	Cazalegas		To	39.1	-4.1	440
00:00.0	FUND. BIODIVER	1684447	Unknown	Aegilops ovata L.	L.	ESP	Spain	Cuneta carretera de Ubrique a El Bo		Ca	36.1	-5.1	
	FUND. BIODIVER	1696523	Unknown	Aegilops geniculata Roth	Roth	ESP	Spain	Iglesuela del Cid, mas de la Loma		Te	40.1	-0.1	1500
	FUND. BIODIVER	1700449	Unknown	Aegilops geniculata		ESP	Spain	Cantavieja, Masia de Porcar		Te	40.1	-0.1	1400
00:00.0	SEV	100520-1	Specimen	Aegilops geniculata Roth	Roth	ES	Spain	Rute. Subida al Cerro del Castillo a		Co			1
00:00.0	SEV	108144-1	Specimen	Aegilops geniculata Roth	Roth	ES	Spain	Priego de CÃ³rdoba. Sierra de Pollo		Co			1
00:00.0	SEV	108145-1	Specimen	Aegilops geniculata Roth	Roth	ES	Spain	BenamejÃ-, entre BenamejÃ- y el T		Co			1
00:00.0	SEV	108262-1	Specimen	Aegilops geniculata Roth	Roth	ES	Spain	Above Grazalema		Ca			1000
	BDBCV-General	275930	Observation	Aegilops geniculata		ESPA	Spain	Bolbaite	La Canal de Nava	Valenc	39.0517	-0.74664	
	BDBCV-General	280032	Observation	Aegilops geniculata		ESPA	Spain	NavarrÃ©s	La Canal de Nava	Valenc	39.0517	-0.74664	
00:00.0	REDIAM-CMA	276142	Observation	Aegilops geniculata		ESP	Spain		Posadas	Co	37.819	-5.15728	200
00:00.0	REDIAM-CMA	281437	Observation	Aegilops geniculata		ESP	Spain		El Viso	Co	38.5198	-4.94798	538
00:00.0	REDIAM-CMA	283083	Observation	Aegilops geniculata		ESP	Spain		Ãšbeda	J	38.0929	-3.4162	482
	SIVIM	T-P30048:Aegi	Observation	Aegilops geniculata Roth	Roth	ES	Spain	Pr. Charca Lubio, Balsa de Ves, Albacete			39.19	-1.26	0
	SIVIM	T-P30115:Aegi	Observation	Aegilops geniculata Roth	Roth	ES	Spain	El Campichuelo, Cofrentes, Valencia			39.19	-1.14	0
	SIVIM	U-P02538:Aegi	Observation	Aegilops geniculata Roth	Roth	ES	Spain	Vall de Gallinera			38.81	-0.35	0
	SIVIM	U-P02921:Aegi	Observation	Aegilops geniculata Roth	Roth	ES	Spain	Benissa, Els Lleus			38.63	0.01	0
	SIVIM	U-P06714:Aegi	Observation	Aegilops geniculata Roth	Roth	ES	Spain	Esteveira			37.13	-8.21	250
	SIVIM	U-P08121:Aegi	Observation	Aegilops geniculata Roth	Roth	ES	Spain	Minas Srra. de San CristÃ³bal, LogrosÃ¡n			39.27	-5.55	0
	BDBCV-General	2710	Observation	Aegilops geniculata		ESPA	Spain	Andilla	Los Serranos	Valenc	39.8642	-0.83717	
	BDBCV-General	274781	Observation	Aegilops geniculata		ESPA	Spain	Chella	La Canal de Nava	Valenc	39.0517	-0.74664	
00:00.0	SEV	101369-1	Specimen	Aegilops geniculata Roth	Roth	ES	Spain	Valencia		V			1
00:00.0	SEV	108083-1	Specimen	Aegilops geniculata Roth	Roth	ES	Spain	Sierra Tejeda. De Canillas de Aceitu		Ma			900
00:00.0	SEV	108255-1	Specimen	Aegilops geniculata Roth	Roth	ES	Spain	Carretera de CÃ³rdoba a Sevilla. En		Co			1
00:00.0	MA	753634-1	Specimen	Aegilops ovata L.	L.	ES	Spain	Barranco de las Gayombas		Gr			
00:00.0	GDAC	GDAC32770-1	Specimen	Aegilops geniculata Roth.	Roth.	ES	Spain	Granada, SÂª Elvira.		GR			650
00:00.0	COA	41162-1	Specimen	Aegilops geniculata Roth	Roth	ES	Spain	Km 6 de Nueva Carteya a Castro de		Co	37.58	-4.59	
00:00.0	HUAL	5973-1	Specimen	Aegilops geniculata Roth	Roth	ES	Spain	SÂª de GÃ¡dor, CaparidÃ¡n		Al	36.934	-2.905	
00:00.0	MA	569166-1	Specimen	Aegilops geniculata Roth	Roth	ES	Spain	Hontalbilla		Sg			
	FUND. BIODIVER	1000495	Unknown	Aegilops geniculata		ESP	Spain	Fresneda de Allarejos		Cu	39.1	-2.1	
	FUND. BIODIVER	1701105	Unknown	Aegilops geniculata		ESP	Spain	Iglesuela del Cid, Pobla del Bellesta		Te	40.1	-0.1	1120
	FUND. BIODIVER	1811881	Unknown	Aegilops ovata		ESP	Spain	Pena Forca Oriental, Rio Subordan,		Hu	42.1	-0.1	1000
00:00.0	COFC	46889-1	Specimen	Aegilops geniculata Roth	Roth	ES	Spain	Hornachuelos; tÃƒÂ©rmino municip		Co			1
	FUND. BIODIVER	1001955	Unknown	Aegilops geniculata		ESP	Spain	Puerto de Cabrejas a Cuenca		Cu	39.1	-2.1	
	FUND. BIODIVER	102488	Unknown	Aegilops geniculata Roth	Roth	ESP	Spain	Padiernos		Av	40.1	-4.1	
	FUND. BIODIVER	1715691	Unknown	Aegilops geniculata Roth	Roth	ESP	Spain	Aiguamolls de l'Empurda		Ge	41.1	2.1	

	FUND. BIODIVER	1811869	Unknown	Aegilops geniculata		ESP	Spain	Sierra de San Pedro, a Gallipienzo,		Na	42.1	-1.1	
	FUND. BIODIVER	1811870	Unknown	Aegilops geniculata		ESP	Spain	Bardena Negra		Na	42.1	-1.1	400
	SEV	82969-1	Specimen	Aegilops ovata L.	L.	ES	Spain	San Roque. El Almendral		Ca			1
00:00.0	SEV	98678-1	Specimen	Aegilops geniculata Roth	Roth	ES	Spain	Entre Torrecampo y San Benito. RÃ		Co			1
00:00.0	REDIAM-CMA	309156	Observation	Aegilops geniculata		ESP	Spain		Mijas	Ma	36.5873	-4.71193	163
00:00.0	REDIAM-CMA	334922	Observation	Aegilops geniculata		ESP	Spain		San Roque	Ca	36.2289	-5.39513	50
00:00.0	REDIAM-CMA	381481	Observation	Aegilops geniculata		ESP	Spain		Huelma	J	37.6765	-3.46982	1149
00:00.0	REDIAM-CMA	382588	Observation	Aegilops geniculata		ESP	Spain		Casares	Ma	36.3951	-5.28492	269
00:00.0	REDIAM-CMA	383370	Observation	Aegilops geniculata		ESP	Spain		Montellano	Se	36.9868	-5.55268	330
00:00.0	REDIAM-CMA	368269	Observation	Aegilops geniculata		ESP	Spain		Obejo	Co	38.1343	-4.78279	533
00:00.0	REDIAM-CMA	381969	Observation	Aegilops geniculata		ESP	Spain		Cambil	J	37.6927	-3.51003	1061
	SIVIM	U-P08153:Aegi	Observation	Aegilops geniculata Roth	Roth	ES	Spain	Dehesa Boyal, LogrosÃ¡n			39.27	-5.55	0
	SIVIM	U-P13322:Aegi	Observation	Aegilops geniculata Roth	Roth	ES	Spain	Marmolejo. Prox. Balneario de Aguas Mine			38.03	-4.25	280
	SANT	51660	Specimen	Aegilops geniculata Roth		ES	Spain	RubiÃ¡; Cobas, entre la Central de (		Or			
	SIVIM	U-P09883:Aegi	Observation	Aegilops geniculata Roth	Roth	ES	Spain	Borde de cultivo de cebada en Ossa de Mo			38.93	-2.76	0
	HUN003	RCAT069711	Specimen	Aegilops geniculata Roth		ESP	Spain						
00:00.0	MGC	11817-1	Unknown	Aegilops geniculata Roth	Roth	ES	Spain	Sierra de OjÃ©n. Dehesa de Boorn		Ma	36.6355	-4.90721	1
00:00.0	MGC	14239-1	Unknown	Aegilops geniculata Roth	Roth	ES	Spain	RincÃ³n de la Victoria; _		Ma	36.719	-4.327	1
	FUND. BIODIVER	1653412	Unknown	Aegilops geniculata		ESP	Spain	Fuentealbilla, hacia Abenjibre		Ab	39.1	-1.1	
	FUND. BIODIVER	1696520	Unknown	Aegilops geniculata Roth	Roth	ESP	Spain	Cantavieja, pista a Tarayuela		Te	40.1	-0.1	1500
	FUND. BIODIVER	1700270	Unknown	Aegilops geniculata		ESP	Spain	Iglesuela del Cid, Masia de la Tosqu		Te	40.1	-0.1	1300
	IPK	32178	Living	Aegilops geniculata Roth		ESP	Spain	W.-Pyrenaen b. Jaca					810
	GDA	GDA30013-1-1	Specimen	Aegilops ovata L.	L.	ES	Spain	Madrid, Aranjuez.		M			0
00:00.0	REDIAM-CMA	242688	Observation	Aegilops geniculata		ESP	Spain		AlhendÃ-n	Gr	37.0367	-3.70528	975
00:00.0	REDIAM-CMA	280077	Observation	Aegilops geniculata		ESP	Spain		Adamuz	Co	38.0162	-4.47505	200
	SIVIM	T-P30045:Aegi	Observation	Aegilops geniculata Roth	Roth	ES	Spain	El Campichuelo, gravera, Jalance, Valencia			39.19	-1.26	0
	SIVIM	T-P30071:Aegi	Observation	Aegilops geniculata Roth	Roth	ES	Spain	Cuesta de subida al Santuario, Villa de Ves			39.19	-1.26	0
	SIVIM	U-P02495:Aegi	Observation	Aegilops geniculata Roth	Roth	ES	Spain	Pego			38.81	-0.12	0
	SIVIM	U-P02915:Aegi	Observation	Aegilops geniculata Roth	Roth	ES	Spain	La Xara XÃ bia			38.72	0	0
	SIVIM	U-P06656:Aegi	Observation	Aegilops geniculata Roth	Roth	ES	Spain	Barreiras Brancas			37.13	-8.09	2500
	SIVIM	U-P08060:Aegi	Observation	Aegilops geniculata Roth	Roth	ES	Spain	La Nava, Berzocana			39.36	-5.55	0
00:00.0	COFC	46886-1	Specimen	Aegilops geniculata Roth	Roth	ES	Spain	ctra CO-140, al pantano del Retortil		Co			1
	FUND. BIODIVER	1024007	Unknown	Aegilops ovata L.	L.	ESP	Spain	Valle de las Correderas		J	38.1	-3.1	
	FUND. BIODIVER	1028171	Unknown	Aegilops geniculata Roth.	Roth.	ESP	Spain	ValdepeÃ±as de JaÃ©n		J	37.1	-3.1	
	FUND. BIODIVER	1713416	Unknown	Aegilops geniculata		ESP	Spain	Mallorca, Calvia, finca Ses Algorfes		PM	39.1	2.1	110
	FUND. BIODIVER	1715688	Unknown	Aegilops geniculata Roth	Roth	ESP	Spain	Aiguamolls de l'Empurda		Ge	42.1	2.1	
	FUND. BIODIVER	1811866	Unknown	Aegilops geniculata		ESP	Spain	Fitero		Na	41.1	-1.1	300
00:00.0	REDIAM-CMA	325698	Observation	Aegilops geniculata		ESP	Spain		Ronda	Ma	36.8375	-5.13667	794
00:00.0	REDIAM-CMA	381852	Observation	Aegilops geniculata		ESP	Spain		MorÃ³n de la Fron	Se	37.1001	-5.3781	396
00:00.0	MGC	5442-1	Unknown	Aegilops geniculata Roth	Roth	ES	Spain	MÃ¡laga; Monte de las Tres Letras		Ma	36.72	-4.4	1
	SIVIM	U-P09295:Aegi	Observation	Aegilops geniculata Roth	Roth	ES	Spain	Frias de Albarracin, pista hacia El Vallecillo			40.28	-1.7	1550

	MA	700959-1	Specimen	Aegilops ovata L.	L.	ES	Spain	Ciudad Universitaria		M			
	IPK	32254	Living	Aegilops geniculata Roth subsp. gibberosa (Zh		ESP	Spain	W.-Pyrenaen, b. Jaca					810
00:00.0	BC	648626	Specimen	Aegilops geniculata Roth	Roth	ES	Spain	MontjuÃ¯c		?			
00:00.0	SALA	112288-1	Specimen	Aegilops geniculata Roth	Roth	ES	Spain	Renedo de Esgueva		Va	41.65	-4.61	
00:00.0	SALA	99835-1	Specimen	Aegilops geniculata Roth	Roth	ES	Spain	_; Castellvi de Rosanes		B	41.46	1.8	
00:00.0	UNEX	11786-1	Observation	Aegilops geniculata Roth	_	ESP	Spain	Aliseda: Finca Valdelayegua. 29SP		Ba	39.4	-6.8	
	SEV	10642-1	Specimen	Aegilops ovata L.	L.	ES	Spain	Madrid		M			1
00:00.0	SALA	98643-1	Specimen	Aegilops geniculata Roth	Roth	ES	Spain	_; Fraga		Z			
00:00.0	GDA	GDA25366-1	Specimen	Aegilops geniculata Roth.	Roth.	ES	Spain	AlmerÃ-a, NÃ-jar, FernÃ¡n PÃ©rez.		AL			200
00:00.0	COA	41169-1	Specimen	Aegilops geniculata Roth	Roth	ES	Spain	Palma del RÃ-o		Co	37.65	-5.38	
00:00.0	BC	627487	Specimen	Aegilops ovata L.	L.	ES	Spain	VandellÃ²s i l'Hospitalet de l'Infant; (		T	40.95	0.92	150
00:00.0	MGC	32940-1	Unknown	Aegilops geniculata Roth	Roth	ES	Spain	Yunquera; Sierra de las Nieves		Ma	0	0	1400
	GDA	GDA30019-1	Specimen	Aegilops ovata L.	L.	ES	Spain	Valladolid, Cuesta de Maruquera.		VA			0
00:00.0	BC	92760	Specimen	Aegilops ovata L.	L.	ES	Spain	Formentera; La Mola Formentera		PM	38.7	1.56	
00:00.0	MGC	62201-1	Unknown	Aegilops geniculata Roth	Roth	ES	Spain	Puebla de los Infantes (La); Entre C		Se	37.764	-5.492	200
00:00.0	MGC	34981-1	Unknown	Aegilops geniculata Roth	Roth	ES	Spain	MÃ¡laga; Campus de Teatinos		Ma	0	0	1
	SEV	7917-1	Specimen	Aegilops ovata L.	L.	ES	Spain	Puebla del RÃ-o		Se			1
00:00.0	SALA	85592-1	Specimen	Aegilops geniculata Roth	Roth	ES	Spain	_; Cabrerizos		Sa			
	FUND. BIODIVER	70868	Unknown	Aegilops ovata L.	L.	ESP	Spain	ValverdÃ³n		Sa	40.1	-5.1	
	BDBCV-General	128855	Observation	Aegilops geniculata		ESPA	Spain				40.0484	-1.06579	
00:00.0	SEV	99208-1	Specimen	Aegilops geniculata Roth	Roth	ES	Spain	Entre Toledo y Polan		To			1
	SIVIM	S-P10115:Aegi	Observation	Aegilops geniculata Roth	Roth	ES	Spain	penyes de l'Albardar, serra de Serrella (Qu			38.63	-0.35	1220
	SIVIM	S-P14134:Aegi	Observation	Aegilops geniculata Roth	Roth	ES	Spain	Cromeleque dos Almendres			42.99	-3.49	0
	SIVIM	T-P01896:Aegi	Observation	Aegilops geniculata Roth	Roth	ES	Spain	Mallorca: al nord de sa Pobla, carretera de			39.74	3	0
	SIVIM	T-P04112:Aegi	Observation	Aegilops geniculata Roth	Roth	ES	Spain	7 km al NW de Yecla			38.65	-1.27	0
	SIVIM	T-P06406:Aegi	Observation	Aegilops geniculata Roth	Roth	ES	Spain	Alcublas			39.72	-0.78	0
00:00.0	MGC	16225-1	Unknown	Aegilops geniculata Roth	Roth	ES	Spain	AlhaurÃ-n de la Torre; Sierra de Mija		Ma	36.63	-4.62	1
00:00.0	UNEX	27691-1	Observation	Aegilops geniculata Roth	_	ESP	Spain	Magacela: Herbazal viario. Sustrato		Ba	38.8	-5.8	
	ESP004	NC050470	Specimen	Aegilops geniculata Roth		ESP	Spain	Tarancon/Carrascosa del Campo 14km E,			40.0667	-2.86667	840
00:00.0	SALA	1562-1	Specimen	Aegilops ovata L.	L.	ES	Spain	_; La Orbada, Monte de La Orbada		Sa			
	FUND. BIODIVER	1115803	Unknown	Aegilops geniculata Rothm.	Rothm.	ESP	Spain	Moreruela de TÃ¡bara		Za	41.1	-5.1	
	FUND. BIODIVER	1141313	Unknown	Aegilops ovata subsp. ovata L.	L.	ESP	Spain	VillargusÃ¡n		Le			
	FUND. BIODIVER	36857	Unknown	Aegilops geniculata Roth	Roth	ESP	Spain	Arreo		Vi	42.1	-2.1	700
	FUND. BIODIVER	62020	Unknown	Aegilops ovata subsp. triaristata		ESP	Spain	Ciudad Rodrigo		Sa	40.1	-6.1	
	COFC	124-1	Specimen	Aegilops ovata L.	L.	ES	Spain	_		?			1
	GDA	GDA30013-1	Specimen	Aegilops ovata L.	L.	ES	Spain	Madrid, Aranjuez.		M			0
	GDA	GDA43468-1	Specimen	Aegilops geniculata Roth.	Roth.	ES	Spain	Granada, Guadix, Rambla Becerra.		GR			950
	REDIAM-CMA	30110	Observation	Aegilops geniculata		ESP	Spain		San JosÃ© del Va	Ca	36.6822	-5.5861	322
	REDIAM-CMA	39541	Observation	Aegilops geniculata		ESP	Spain		San JosÃ© del Va	Ca	36.6305	-5.66641	189
00:00.0	REDIAM-CMA	47846	Observation	Aegilops geniculata		ESP	Spain		Almonaster la Re	H	37.8379	-6.76089	392
00:00.0	REDIAM-CMA	52856	Observation	Aegilops geniculata		ESP	Spain		PeÃ±aflor	Se	37.7492	-5.36302	169

00:00.0	REDIAM-CMA	63292	Observation	Aegilops geniculata		ESP	Spain		BaÃ±os de la Enc	J	38.1926	-3.84157	489
00:00.0	BDBCV	101	Observation	Aegilops geniculata		ESP	Spain	Parque Natural de Penyagolosa		Cs			
00:00.0	SALA	18742-1	Specimen	Aegilops ovata L. subsp. triaristat	(Willd.) Rouy	ES	Spain	_; Villarino de los Aires		Sa			
	SIVIM	R-P09694:Aegi	Observation	Aegilops geniculata Roth	Roth	ES	Spain	Muduex, camino en lo alto del pÃ¡ramo			40.83	-3	1010
	SIVIM	R-P10578:Aegi	Observation	Aegilops geniculata Roth	Roth	ES	Spain	Casas de Pelea , AlcalÃ¡ de los Gazules			36.38	-5.78	0
	SIVIM	R-P11716:Aegi	Observation	Aegilops geniculata Roth	Roth	ES	Spain	Cigales			41.71	-4.8	0
	SIVIM	S-P01260:Aegi	Observation	Aegilops geniculata Roth	Roth	ES	Spain	l'Espluga Calba, els Graus			41.44	0.96	0
	SIVIM	S-P03976:Aegi	Observation	Aegilops geniculata Roth	Roth	ES	Spain	Nalec, barranc de ComangrÃ s			41.53	1.08	0
00:00.0	BDBCV	335974	Observation	Aegilops geniculata	_	ES	Spain	Parc Natural de la	L'AlfÃ s del Pi	A	38.59	-0.07	
00:00.0	COFC	36345-1	Specimen	Aegilops geniculata Roth	Roth	ES	Spain	BenamejÃƒÂ-; cruce del rÃƒÂ-o Ge		Co			1
00:00.0	GDA	GDA10273-1-2	Specimen	Aegilops ovata L. pro parte	L. pro parte	ES	Spain	Granada, Ã•zbor, camino de LanjarÃ		GR			600
00:00.0	GDA	GDA13286-1-1	Specimen	Aegilops ovata L.	L.	ES	Spain	MÃ¡laga, CoÃ-n, la AlbuquerÃ-a.		MA			0
00:00.0	FCO	20763-1	Specimen	Aegilops ovata L.	L.	ES	Spain	Madrid; Madrid, Ciudad Universitaria		M			
00:00.0	BC	92764	Specimen	Aegilops ovata L.	L.	ES	Spain	Sant Guim de Freixenet; St. Guim L		L	41.68	1.38	
00:00.0	ABH	52964-1	Specimen	Aegilops geniculata Roth	Roth	ES	Spain	Biar; Biar-Banyeres, Camino Cueva		A	38.65	-0.72	
	FUND. BIODIVER	1554636	Unknown	Aegilops ovata L.	L.	ESP	Spain	LeÃ³n		Le	42.1	-5.1	
00:00.0	FUND. BIODIVER	1593500	Unknown	Aegilops geniculata		ESP	Spain	Guardo		P	42.1	-4.1	1120
	FUND. BIODIVER	998069	Unknown	Aegilops geniculata		ESP	Spain	Cuenca		Cu	39.1	-1.1	
00:00.0	COFC	41156-1	Specimen	Aegilops geniculata Roth	Roth	ES	Spain	Santaella; rÃƒÂ-o Cabra; cerro Cam		Co	37	-4	1
00:00.0	COFC	41294-1	Specimen	Aegilops geniculata Roth	Roth	ES	Spain	Baena; rÃƒÂ-o Guadajoz; carretera		Co	37	-4	1
00:00.0	COA	41168-1	Specimen	Aegilops geniculata Roth	Roth	ES	Spain	Sierra del Castillo, cerca de Espiel,		Co	38.11	-5.05	
00:00.0	BDBCV	335582	Observation	Aegilops geniculata	_	ES	Spain	Parc Natural de la	L'AlfÃ s del Pi	A	38.59	-0.07	
00:00.0	ABH	10072-1	Specimen	Aegilops geniculata Roth	Roth	ES	Spain	Novelda; prox. Collado de Benisa		A	38.49	-0.74	
00:00.0	COFC	41154-1	Specimen	Aegilops geniculata Roth	Roth	ES	Spain	entre Cabra y Nueva Carteya		Co	37	-4	1
00:00.0	COFC	41292-1	Specimen	Aegilops geniculata Roth	Roth	ES	Spain	Lucena; Km-63 de la carretera entre		Co	37	-4	1
00:00.0	REDIAM-CMA	226005	Observation	Aegilops geniculata		ESP	Spain		Fonelas	Gr	37.4396	-3.17109	778
00:00.0	REDIAM-CMA	233144	Observation	Aegilops geniculata		ESP	Spain		La Nava	H	37.9565	-6.72782	500
	PRT005	2402	Specimen	Aegilops ovata L.		ESP	Spain						
00:00.0	SALA	16050-1	Specimen	Aegilops ovata L. subsp. triaristat	(Willd.) Rouy	ES	Spain	_; Villares de Yeltes		Sa			
	SIVIM	T-P15403:Aegi	Observation	Aegilops geniculata Roth	Roth	ES	Spain	Castrovega del Valmadrigal			42.24	-5.3	0
	SIVIM	T-P16917:Aegi	Observation	Aegilops geniculata Roth	Roth	ES	Spain	CobeÃ±a			40.55	-3.59	0
	SIVIM	T-P19983:Aegi	Observation	Aegilops geniculata Roth	Roth	ES	Spain	Almaraz			39.8	-5.68	320
	SIVIM	T-P25938:Aegi	Observation	Aegilops geniculata Roth	Roth	ES	Spain	JaÃ©n (polÃ-gono Llanos del Valle)			37.85	-3.79	0
	SIVIM	T-P27678:Aegi	Observation	Aegilops geniculata Roth	Roth	ES	Spain	PrÃ³x. Cj. MolejÃ³n, SÂª Gallinera			37.39	-4.35	0
	SIVIM	T-P29794:Aegi	Observation	Aegilops geniculata Roth	Roth	ES	Spain	Fuente de la Salaboreja, Casas de Ves, Al			39.19	-1.37	0
	SIVIM	T-P26949:Aegi	Observation	Aegilops ovata L.	L.	ES	Spain	*			41.33	-0.25	340
00:00.0	COFC	41269-1	Specimen	Aegilops geniculata Roth	Roth	ES	Spain	Puente Genil; rÃƒÂ-o Genil; presa C		Co	37	-4	1
00:00.0	GDA	GDA30029-1-2	Specimen	Aegilops geniculata Roth.	Roth.	ES	Spain	JaÃ©n, SÂª MÃ¡gina, SÂª de la Cru		J			1000
00:00.0	GDA	GDA7741-1-1	Specimen	Aegilops ovata L. var. pubiglumis	L.	ES	Spain	Madrid, La Moncloa.		M			0
00:00.0	ABH	43522-1	Specimen	Aegilops geniculata Roth	Roth	ES	Spain	La ArmuÃ±a;		Sa			
00:00.0	LEB	4786-1	Specimen	Aegilops geniculata Roth	Roth	ES	Spain	MiÃ±era de Luna		Le	42.91	-5.88	1

00:00.0	COFC	41149-1	Specimen	Aegilops geniculata Roth	Roth	ES	Spain	Cabra; rÃƒÂ-o SantamarÃƒÂ-a; arro		Co	37	-4	1
00:00.0	COFC	41283-1	Specimen	Aegilops geniculata Roth	Roth	ES	Spain	Cabra; 'La Chacona'		Co	37	-5	1
00:00.0	COFC	46890-1	Specimen	Aegilops geniculata Roth	Roth	ES	Spain	cortijo 'Guadalora', ctra CO-140, Km		Co			1
	FUND. BIODIVER	1001970	Unknown	Aegilops geniculata		ESP	Spain	Valle de Valdemeca		Cu	40.1	-1.1	
	FUND. BIODIVER	102489	Unknown	Aegilops geniculata Roth	Roth	ESP	Spain	MuÃ±ogalindo		Av	40.1	-4.1	
	FUND. BIODIVER	1811871	Unknown	Aegilops geniculata Roth	Roth	ESP	Spain	Monte Pena, Sierra de Santo Domin		Na	42.1	-1.1	900
	IDBD-GN	42462	Observation	Aegilops geniculata Roth	Roth	ES	Spain	Higa	Monreal	Na	42.6977	-1.53013	1000
	IDBD-GN	42483	Observation	Aegilops geniculata Roth	Roth	ES	Spain		Viana	Na	42.4981	-2.33803	480
	IDBD-GN	42505	Observation	Aegilops geniculata Roth	Roth	ES	Spain	Villa romana frent	Lumbier	Na	42.6403	-1.28754	440
	REDIAM-CMA	296430	Observation	Aegilops geniculata		ESP	Spain		Jerez de la Fronte	Ca	36.575	-5.60497	383
00:00.0	REDIAM-CMA	327285	Observation	Aegilops geniculata		ESP	Spain		Casares	Ma	36.4348	-5.28044	200
00:00.0	REDIAM-CMA	327313	Observation	Aegilops geniculata		ESP	Spain		Casares	Ma	36.4339	-5.28117	199
00:00.0	REDIAM-CMA	382015	Observation	Aegilops geniculata		ESP	Spain		MorÃ³n de la Fron	Se	37.0684	-5.33556	399
00:00.0	REDIAM-CMA	382037	Observation	Aegilops geniculata		ESP	Spain				37.8829	-3.41014	
00:00.0	REDIAM-CMA	382936	Observation	Aegilops geniculata		ESP	Spain		Montellano	Se	36.9646	-5.5211	299
	SIVIM	U-P09884:Aegi	Observation	Aegilops geniculata Roth	Roth	ES	Spain	Erial entre Ossa y Villahermosa			38.66	-2.88	0
00:00.0	GDA	GDA30020-1-2	Specimen	Aegilops geniculata Roth.	Roth.	ES	Spain	Granada, SÂª Elvira.		GR			0
	IPK	31910	Living	Aegilops geniculata Roth subsp. gibberosa (Zh		ESP	Spain	Barcelona					
	GDA	GDA30019-1-2	Specimen	Aegilops geniculata Roth.	Roth.	ES	Spain	Valladolid, Cuesta de Maruquera.		VA			0
	DEU146	AE 1049	Specimen	Aegilops geniculata Roth		ESP	Spain	Cortiguera (Burgos)					
1887-06-1	US	1018345.284	Specimen	Aegilops ovata		Spain	Spain	Algerciras		Cadiz			
00:00.0	SALA	52034-1	Specimen	Aegilops geniculata Roth	Roth	ES	Spain	_; Aspariegos, Las Cabecinas		Za			
00:00.0	SEV	108244-1	Specimen	Aegilops geniculata Roth	Roth	ES	Spain	Priego de CÃ³rdoba. Cerro Gallardo		Co			1
00:00.0	SEV	108280-1	Specimen	Aegilops geniculata Roth	Roth	ES	Spain	Villaverde del RÃ-o		Se			1
00:00.0	MGC	60333-1	Unknown	Aegilops geniculata Roth	Roth	ES	Spain	AlcaucÃ-n; P. N. de las Sierras de T		Ma	36.911	-4.106	600
	BDBCV-General	274784	Observation	Aegilops geniculata		ESPA	Spain	Chella	La Canal de Nava	Valenc	39.0517	-0.74664	
	ADIMAN	39	Observation	Aegilops geniculata		ESP	Spain	EnguÃ-danos		CU	39.7161	-1.55425	
00:00.0	SEV	108245-1	Specimen	Aegilops geniculata Roth	Roth	ES	Spain	Priego de CÃ³rdoba. Cerro de los Y		Co			1
00:00.0	SEV	108281-1	Specimen	Aegilops geniculata Roth	Roth	ES	Spain	Lucena. Cerro de la Galeota		Co			1
	ADIMAN	21	Observation	Aegilops geniculata		ESP	Spain	EnguÃ-danos		CU	39.6387	-1.66992	
00:00.0	MGC	44024-1	Unknown	Aegilops geniculata Roth	Roth	ES	Spain	Estepona; Sierra Bermeja. Tinao de		Ma	0	0	160
00:00.0	MGC	45949-1	Unknown	Aegilops geniculata Roth	Roth	ES	Spain	Ronda; P. N. Sierra de las Nieves. C		Ma	36.7	-5.032	1
00:00.0	MGC	45950-1	Unknown	Aegilops geniculata Roth	Roth	ES	Spain	Ronda; P. N. Sierra de las Nieves. F		Ma	36.691	-5.043	1300
00:00.0	BC	597975	Specimen	Aegilops ovata L.	L.	ES	Spain	Almacelles; SegriÃ : Vers Almacelle		L	41.75	0.41	
00:00.0	SALA	81681-1	Specimen	Aegilops geniculata Roth	Roth	ES	Spain	_; GuareÃ±a		Ba			
00:00.0	MGC	62695-1	Unknown	Aegilops geniculata Roth	Roth	ES	Spain	Zufre; Entre Dehesa de las Esposas		H	37.78	-6.44	421
	GDAC	GDAC2932-1	Specimen	Aegilops ovata L.	L.	ES	Spain	Murcia, alrededores de Cartagena.		MU			0
00:00.0	BC	70803	Specimen	Triticum ovatum (L.) Gren. & God	(L.) Gren. &	ES	Spain	Ontigola; Castella nova: Ontigola, c		To			
00:00.0	SEV	100517-1	Specimen	Aegilops geniculata Roth	Roth	ES	Spain	Rute. Barranco cercano al Cortijo de		Co			1
00:00.0	SEV	102469-1	Specimen	Aegilops geniculata Roth	Roth	ES	Spain	Carmona		Se			1
00:00.0	SEV	108141-1	Specimen	Aegilops geniculata Roth	Roth	ES	Spain	Cortijo Guadalora. Arroyo Guadalor		Co			1

00:00.0	SEV	108259-1	Specimen	Aegilops geniculata Roth	Roth	ES	Spain	Arcos de la Frontera. Pantano del G		Ca			1
00:00.0	UPS	V-200860	Specimen	Aegilops geniculata		Spain	Spain	Mallorca, Muro		Baleares			
00:00.0	COA	41166-1	Specimen	Aegilops geniculata Roth	Roth	ES	Spain	PeÃ±arrolla-Pueblonuevo		Co	38.29	-5.29	
	COA	41210-1	Specimen	Aegilops geniculata Roth	Roth	ES	Spain	Sierra Madrona		CR	38.39	-4.26	
	CZE122	01C2109013	Specimen	Aegilops geniculata Roth		ESP	Spain	Gelaende von El Enci bai Alcala de Henares					
00:00.0	SEV	99212-1	Specimen	Aegilops geniculata Roth	Roth	ES	Spain	Entre El Gandul y Trujillo		Se			1
00:00.0	BDBCV	588904	Observation	Aegilops geniculata		ESP	Spain	Parc Natural de la	Alcoy	Alicant	38.683	-0.534	
	FUND. BIODIVER	1696526	Unknown	Aegilops geniculata Roth	Roth	ESP	Spain	Puertomingalvo, pista del Letrado		Te	40.1	-0.1	1500
	FUND. BIODIVER	1700452	Unknown	Aegilops geniculata		ESP	Spain	Puertomingalvo, inicio pista del Letr		Te	40.1	-0.1	1500
	SANT	19840	Specimen	Aegilops ovata L.		ES	Spain	Saelices de la Sal		Gu			
	BDBCV-General	25256	Observation	Aegilops geniculata		ESPA	Spain	Villena	L'Alt VinalopÃ³	Alicant	38.6036	-0.87555	
00:00.0	UNEX	10373-1	Observation	Aegilops geniculata Roth	_	ESP	Spain	Jerez de los Caballeros: Brovales.R		Ba	38.3	-6.7	
00:00.0	REDIAM-CMA	276585	Observation	Aegilops geniculata		ESP	Spain		El Guijo	Co	38.5249	-4.71878	486
00:00.0	MA	597793-1	Specimen	Aegilops geniculata Roth.	Roth.	ES	Spain	puerto de Pozazal		S	42	-4	
00:00.0	HUAL	12474-1	Specimen	Aegilops geniculata Roth	Roth	ES	Spain	NÃ-jar; P.N. Cabo de Gata-NÃ-jar, F		Al	36.849	-2.041	
00:00.0	GDA	GDA23046-1	Specimen	Aegilops geniculata Roth.	Roth.	ES	Spain	Granada, Padul, cerro de los Molino		GR			750
00:00.0	GDAC	GDAC24838-1	Specimen	Aegilops geniculata Roth.	Roth.	ES	Spain	Zamora, Castrillo de la GuareÃ±a.		ZA			0
	SIVIM	T-P30051:Aegi	Observation	Aegilops geniculata Roth	Roth	ES	Spain	Pr. Hazas del Calvario, Casas IbÃ¡Ã±ez, A			39.19	-1.49	0
	SIVIM	T-P30176:Aegi	Observation	Aegilops geniculata Roth	Roth	ES	Spain	Pr. Las Salinas, Casas de Ves, Albacete			39.28	-1.37	0
	SIVIM	U-P02614:Aegi	Observation	Aegilops geniculata Roth	Roth	ES	Spain	Castells de Serrella			38.63	-0.24	0
	SIVIM	U-P06720:Aegi	Observation	Aegilops geniculata Roth	Roth	ES	Spain	CabeÃ§a Gorda			37.13	-8.21	240
	SIVIM	U-P08134:Aegi	Observation	Aegilops geniculata Roth	Roth	ES	Spain	La Nava, Berzocana			39.36	-5.55	0
00:00.0	BC	806706	Specimen	Aegilops geniculata Roth	Roth	ES	Spain	Granada; Silla del Moro, colina de la		Gr	37.18	-3.62	850
00:00.0	MGC	25572-1	Unknown	Aegilops geniculata Roth	Roth	ES	Spain	Entre Alhama y AlcaicerÃ-a		Gr	0	0	1
00:00.0	FUND. BIODIVER	1684419	Unknown	Aegilops ovata L.	L.	ESP	Spain	Margenes Arroyo El Descansadero		Ca	36.1	-5.1	
	SANT	8524	Specimen	Aegilops ovata L. var. leptostachya Mihi		ES	Spain	Cabrera,en el Puerto		PM			
00:00.0	SEV	98933-1	Specimen	Aegilops geniculata Roth	Roth	ES	Spain	BoltaÃ±a. Cauce del RÃ-o Ara		Hu			1
00:00.0	REDIAM-CMA	274354	Observation	Aegilops geniculata		ESP	Spain		BelalcÃ¡zar	Co	38.5377	-5.09983	530
00:00.0	REDIAM-CMA	279235	Observation	Aegilops geniculata		ESP	Spain		CÃ³rdoba	Co	37.9421	-4.69126	183
00:00.0	COFC	29473-1	Specimen	Aegilops geniculata Roth	Roth	ES	Spain	Espiel; El Paquillo		Co			1
00:00.0	BC	93042	Specimen	Aegilops ovata L.	L.	ES	Spain	Benifallet; circa Balnearium		T	40.94	0.56	540
00:00.0	BC	830207	Specimen	Aegilops ovata L.	L.	ES	Spain	Palma de Mallorca; Cabrera		PM	39.16	2.94	
	SIVIM	T-P30042:Aegi	Observation	Aegilops geniculata Roth	Roth	ES	Spain	AlterÃ³n de Bucar, Jalance, Valencia			39.19	-1.14	330
	SIVIM	T-P30064:Aegi	Observation	Aegilops geniculata Roth	Roth	ES	Spain	Puntal blanco-Las Salinas, Casas de Ves,			39.19	-1.37	0
	SIVIM	U-P00821:Aegi	Observation	Aegilops geniculata Roth	Roth	ES	Spain	Prox. El Valle de Las Casas			42.7	-5.07	1130
	SIVIM	U-P02912:Aegi	Observation	Aegilops geniculata Roth	Roth	ES	Spain	Castells de Serrella			38.63	-0.24	0
	SIVIM	U-P06653:Aegi	Observation	Aegilops geniculata Roth	Roth	ES	Spain	Rocha da Pena			37.22	-8.21	0
	SIVIM	U-P07720:Aegi	Observation	Aegilops geniculata Roth	Roth	ES	Spain	CastellÃ³n: Almenara			44.1	-14.87	0
	BC	92063	Specimen	Aegilops ovata L.	L.	ES	Spain	Zamora; Zamora		Za	41.48	-5.69	
00:00.0	BC	642003	Specimen	Aegilops geniculata Roth	Roth	ES	Spain	Luque; Luque (Andalusia)		Co	37.53	-4.3	
	RUS001	VIR100602122	Specimen	Aegilops ovata L.		ESP	Spain						

	RUS001	VIR100602101	Specimen	Aegilops ovata L.		ESP	Spain						
	RUS001	VIR100602080	Specimen	Aegilops ovata L.		ESP	Spain						
	W	42089	Unknown	Aegilops ovata L.		ESP	Spain	Madrid. par MuBoissier.					
	FUND. BIODIVER	1074140	Unknown	Aegilops ovata		ESP	Spain	Puente Genil		Co	37.1	-4.1	
00:00.0	FUND. BIODIVER	1946405	Unknown	Aegilops geniculata Roth	Roth	ESP	Spain	Alamillo, laderas del cerro del Tamb		CR	38.1	-4.1	600
	FUND. BIODIVER	1648479	Unknown	Aegilops geniculata Roth	Roth	ESP	Spain	Puertomingalvo, pista del Letrado		Te	40.1	-0.1	
	FUND. BIODIVER	1696528	Unknown	Aegilops geniculata Roth	Roth	ESP	Spain	Iglesuela del Cid, pista arriba del ma		Te	40.1	-0.1	1500
	FUND. BIODIVER	1700454	Unknown	Aegilops geniculata		ESP	Spain	Cantavieja, pista a Tarayuela		Te	40.1	-0.1	1380
00:00.0	UNEX	10375-1	Observation	Aegilops geniculata Roth	_	ESP	Spain	La Morera: Entre La Morera y Noga		Ba	38.5	-6.7	
00:00.0	REDIAM-CMA	6104	Observation	Aegilops geniculata		ESP	Spain		Carratraca	Ma	36.8456	-4.79157	698
00:00.0	REDIAM-CMA	398043	Observation	Aegilops geniculata		ESP	Spain		Villanueva de la R	J	38.1802	-3.85999	397
00:00.0	REDIAM-CMA	409981	Observation	Aegilops geniculata		ESP	Spain		Villarrodrigo	J	38.4673	-2.67613	893
00:00.0	REDIAM-CMA	414387	Observation	Aegilops geniculata		ESP	Spain		VÃ-car	Al	36.8309	-2.63638	359
00:00.0	REDIAM-CMA	416854	Observation	Aegilops geniculata		ESP	Spain		Tabernas	Al	37.0823	-2.34965	487
00:00.0	REDIAM-CMA	244040	Observation	Aegilops geniculata		ESP	Spain		Aroche	H	37.9472	-6.89504	399
00:00.0	REDIAM-CMA	255121	Observation	Aegilops geniculata		ESP	Spain		Chirivel	Al	37.6102	-2.3181	1256
00:00.0	REDIAM-CMA	276712	Observation	Aegilops geniculata		ESP	Spain		Dos Torres	Co	38.55	-4.82413	491
	IPK	AE 1168	Living	Aegilops geniculata Roth			Spain	Tras-os-Montes (Alto Douro), Franca - Brag			41.9008	-6.26056	
	SIVIM	Q-P03046:Aegi	Observation	Aegilops geniculata Roth	Roth	ES	Spain	St. MagÃ- de Brufaganya, vora el camÃ- (E			41.45	1.44	0
	SIVIM	Q-P03626:Aegi	Observation	Aegilops geniculata Roth	Roth	ES	Spain	Torrechiva			39.99	-0.42	340
	SIVIM	Q-P06905:Aegi	Observation	Aegilops geniculata Roth	Roth	ES	Spain	Rambla de los Molinos, Petrer,			38.46	-0.82	0
	SIVIM	R-P02664:Aegi	Observation	Aegilops geniculata Roth	Roth	ES	Spain	marge de la carretera de Torres al campan			41.43	0.48	150
	SIVIM	R-P06812:Aegi	Observation	Aegilops geniculata Roth	Roth	ES	Spain	Albacete, hacia El Encinar de Los Yesares			39.02	-1.84	0
	SIVIM	R-P08527:Aegi	Observation	Aegilops geniculata Roth	Roth	ES	Spain	CaparidÃ¡n			36.86	-3	160
	SIVIM	T-P29983:Aegi	Observation	Aegilops geniculata Roth	Roth	ES	Spain	Pr. Las Eras, AlcalÃ¡ del JÃºcar, Albacete			39.19	-1.49	0
	SIVIM	T-P30053:Aegi	Observation	Aegilops geniculata Roth	Roth	ES	Spain	Barranco de Mingo AndrÃ©s, Villa de Ves,			39.19	-1.37	0
	SIVIM	T-P30248:Aegi	Observation	Aegilops geniculata Roth	Roth	ES	Spain	Campo del Cura/Embarcaderos, Cofrentes			39.19	-1.14	322
	SIVIM	U-P02616:Aegi	Observation	Aegilops geniculata Roth	Roth	ES	Spain	Vall d'AlcalÃ , AlcalÃ			38.72	-0.35	0
	SIVIM	U-P06636:Aegi	Observation	Aegilops geniculata Roth	Roth	ES	Spain	Paderne			37.13	-8.21	0
	SIVIM	U-P06722:Aegi	Observation	Aegilops geniculata Roth	Roth	ES	Spain	Rocha da Pena			37.22	-8.09	0
00:00.0	BC	146632	Specimen	Aegilops ovata L.	L.	ES	Spain	Vistabella del Maestrazgo; Vistabell		Cs	40.3	-0.23	
	COFC	123-1	Specimen	Aegilops ovata L.	L.	ES	Spain	Casa de Campo		M			1
00:00.0	GDA	GDA25153-1	Specimen	Aegilops geniculata Roth.	Roth.	ES	Spain	Granada, Pedro MartÃ-nez, Mencal		GR			1150
00:00.0	GDAC	GDAC26134-1	Specimen	Aegilops geniculata Roth.	Roth.	ES	Spain	Granada, SÂª de Baza, cerca de Na		GR			1450
00:00.0	BC	70800	Specimen	Aegilops geniculata Roth	Roth	ES	Spain	Cabra de Santo Cristo; Cabra del S		J	37.72	-3.28	1000
00:00.0	MA	718180-1	Specimen	Aegilops geniculata Roth.	Roth.	ES	Spain	LiÃ©dena, La Sarda.		Na	42	-1	
	IPK	32306	Living	Aegilops geniculata Roth subsp. geniculata		ESP	Spain						
00:00.0	BC	92729	Specimen	Aegilops ovata L.	L.	ES	Spain	Castelldefels; Castelldefels		B	41.32	1.98	
00:00.0	BC	92736	Specimen	Aegilops ovata L.	L.	ES	Spain	Almacelles; Almacelles Lleida		L	41.75	0.41	
00:00.0	SEV	99076-1	Specimen	Aegilops geniculata Roth	Roth	ES	Spain	Entre Valdezufre y Jabuguillo		H			1
	CZE122	01C2109008	Specimen	Aegilops geniculata Roth		ESP	Spain	Nocito Espagne (Spanien)					

00:00.0	MGC	18798-1	Unknown	Aegilops geniculata Roth	Roth	ES	Spain	Mijas; Sitio de Calahonda		Ma	36.54	-4.73	60
00:00.0	BC	860253	Specimen	Aegilops ovata L.	L.	ES	Spain	Palma de Mallorca; Mallorca: Son R		PM	39.61	2.59	
00:00.0	GDA	GDA25152-1	Specimen	Aegilops geniculata Roth.	Roth.	ES	Spain	Granada, Pedro MartÃ-nez, Mencal		GR			1150
00:00.0	FCO	4046-1	Specimen	Aegilops ovata L.	L.	ES	Spain	Morata de TajuÃƒÂ±a		M			
00:00.0	BC	70797	Specimen	Aegilops ovata L.	L.	ES	Spain	Castrillo de la Reina; Castrillo de la		Bu	41.96	-3.18	
00:00.0	BDBCV	117	Observation	Aegilops geniculata		ESP	Spain	Parque Natural de Penyagolosa		Cs			
	RUS001	VIR100602106	Specimen	Aegilops ovata L.		ESP	Spain						
00:00.0	BDBCV	591312	Observation	Aegilops geniculata		ESP	Spain		Alcoy	Alicant	38.684	-0.568	
	FUND. BIODIVER	1090389	Unknown	Aegilops geniculata		ESP	Spain	CobeÃ±a		M	40.1	-3.1	
	FUND. BIODIVER	1093215	Unknown	Aegilops ovata L.	L.	ESP	Spain			Cc			
	FUND. BIODIVER	1093236	Unknown	Aegilops geniculata		ESP	Spain	Finca de Araya, Arroyo de la Luz		Cc	39.1	-6.1	
00:00.0	FUND. BIODIVER	1946400	Unknown	Aegilops geniculata Roth	Roth	ESP	Spain	Villamayor de Calatrava, volcan del		CR	38.1	-3.1	842
00:00.0	REDIAM-CMA	18852	Observation	Aegilops geniculata		ESP	Spain		Antequera	Ma	36.952	-4.58012	978
00:00.0	REDIAM-CMA	396749	Observation	Aegilops geniculata		ESP	Spain		Vejer de la Fronte	Ca	36.3657	-6.00016	100
00:00.0	REDIAM-CMA	402773	Observation	Aegilops geniculata		ESP	Spain		Cazorla	J	37.9335	-2.91713	1188
00:00.0	REDIAM-CMA	408744	Observation	Aegilops geniculata		ESP	Spain		Vilches	J	38.312	-3.42388	596
00:00.0	REDIAM-CMA	413720	Observation	Aegilops geniculata		ESP	Spain		Cortegana	H	37.937	-6.7906	465
00:00.0	REDIAM-CMA	415911	Observation	Aegilops geniculata		ESP	Spain		Quesada	J	37.841	-3.04288	805
	SIVIM	Q-P03039:Aegi	Observation	Aegilops geniculata Roth	Roth	ES	Spain	La Llacuna, prop del dipÃ²sit d'aigua (Anoia			41.45	1.44	0
	SIVIM	Q-P03606:Aegi	Observation	Aegilops geniculata Roth	Roth	ES	Spain	Villamalur			39.99	-0.42	0
	SIVIM	Q-P06787:Aegi	Observation	Aegilops geniculata Roth	Roth	ES	Spain	Batech, Petrer,			38.46	-0.82	0
	SIVIM	R-P00085:Aegi	Observation	Aegilops geniculata Roth	Roth	ES	Spain	rodalies de Moror, vers el barranc del Bosc			41.98	0.82	0
	SIVIM	R-P06671:Aegi	Observation	Aegilops geniculata Roth	Roth	ES	Spain	Fuensanta. Cerro del Gato.			39.2	-2.07	0
	SIVIM	R-P08276:Aegi	Observation	Aegilops geniculata Roth	Roth	ES	Spain	Sota can SolÃ de la Balma (el Bruc)			41.54	1.68	0
00:00.0	GDA	GDA25366-1-2	Specimen	Aegilops geniculata Roth.	Roth.	ES	Spain	AlmerÃ-a, NÃ-jar, FernÃ¡n PÃ©rez.		AL			200
	CZE122	01C2109082	Specimen	Aegilops geniculata Roth		ESP	Spain						
00:00.0	MGC	28989-1	Unknown	Aegilops geniculata Roth	Roth	ES	Spain	Alhama de Granada; Sierra Tejeda.		Gr	36.956	-4.028	1100
	IDBD-GN	42464	Observation	Aegilops geniculata Roth	Roth	ES	Spain		MaÃ±eru	Na	42.6697	-1.84197	
	IDBD-GN	42475	Observation	Aegilops geniculata Roth	Roth	ES	Spain		Los Arcos	Na	42.5241	-2.17948	500
	IDBD-GN	42497	Observation	Aegilops geniculata Roth	Roth	ES	Spain		La Negra	Na	42.1025	-1.47141	400
	IDBD-GN	42506	Observation	Aegilops geniculata Roth	Roth	ES	Spain	RÃ-o Urrobi	Orbaiz	Na	42.8306	-1.36793	600
00:00.0	GDA	GDA10274-1-3	Specimen	Aegilops geniculata Roth. var. pu	Roth.	ES	Spain	Granada, SÂª de Mecina, Pitres, fal		GR			1150
	IDBD-GN	42430	Observation	Aegilops geniculata Roth	Roth	ES	Spain		Caparroso	Na	42.3069	-1.606	
	IDBD-GN	42451	Observation	Aegilops geniculata Roth	Roth	ES	Spain		GoÃ±i	Na	42.8498	-1.83861	
00:00.0	SALA	78447-1	Specimen	Aegilops geniculata Roth	Roth	ES	Spain	_; Coria del Rio		Se			
00:00.0	SEV	99080-1	Specimen	Aegilops geniculata Roth	Roth	ES	Spain	Sierra Blanca de OjÃ©n		Ma			1
00:00.0	BDBCV	589112	Observation	Aegilops geniculata		ESP	Spain		Alcoy	Alicant	38.674	-0.523	
00:00.0	FUND. BIODIVER	1774960	Unknown	Aegilops geniculata Roth	Roth	ESP	Spain	Almunia de San Juan		Hu	41.1	0.1	370
	FUND. BIODIVER	1811879	Unknown	Aegilops geniculata Roth.	Roth.	ESP	Spain	Lumbier, La Oquia		Na	42.1	-1.1	600
	BDBCV-General	81753	Observation	Aegilops geniculata		ESPA	Spain	Altura	El Alto Palancia	Castel	39.8596	-0.60353	
00:00.0	COFC	11968-1	Specimen	Aegilops geniculata Roth	Roth	ES	Spain	Lucena; cortijo 'El Duque'		Co	37	-4	1

00:00.0	COFC	11994-1	Specimen	Aegilops geniculata Roth	Roth	ES	Spain	Rute; Cierzos Altos		Co	37	-4	1
00:00.0	COFC	1979-1	Specimen	Aegilops ovata L.	L.	ES	Spain	rÃƒÂ-o Guadiato; cerro del Trigo		Co			1
00:00.0	REDIAM-CMA	364592	Observation	Aegilops geniculata		ESP	Spain		Turre	Al	37.1352	-1.94053	100
00:00.0	REDIAM-CMA	381439	Observation	Aegilops geniculata		ESP	Spain		BailÃ©n	J	38.1176	-3.72405	313
00:00.0	REDIAM-CMA	382534	Observation	Aegilops geniculata		ESP	Spain		Cortegana	H	37.8904	-6.84593	630
00:00.0	REDIAM-CMA	383240	Observation	Aegilops geniculata		ESP	Spain				37.7228	-3.3086	
00:00.0	SEV	99216-1	Specimen	Aegilops geniculata Roth	Roth	ES	Spain	Alrededores de Estepona		Ma			1
00:00.0	GDA	GDA19440-1-1	Specimen	Aegilops geniculata Roth.	Roth.	ES	Spain	JaÃ©n, Colegio Universitario.		J			440
00:00.0	GDA	GDA22404-1-1	Specimen	Aegilops geniculata Roth.	Roth.	ES	Spain	Granada, Cogollos Vega, proximida		GR			1000
	SIVIM	U-P08151:Aegi	Observation	Aegilops geniculata Roth	Roth	ES	Spain	Dehesa Boyal, LogrosÃ¡n			39.27	-5.55	0
	SIVIM	U-P13296:Aegi	Observation	Aegilops geniculata Roth	Roth	ES	Spain	Cruce Ctras. Torredonjimeno-Porcuna con			37.76	-4.02	0
	BDBCV-General	276665	Observation	Aegilops geniculata		ESPA	Spain	NavarrÃ©s	La Canal de Nava	Valenc	39.0517	-0.74664	
	IDBD-GN	42477	Observation	Aegilops geniculata Roth	Roth	ES	Spain	Olatz	Aibar	Na	42.6143	-1.3614	760
	FUND. BIODIVER	1115801	Unknown	Aegilops geniculata Rothm.	Rothm.	ESP	Spain	Pozuelo de TÃ¡bara		Za	41.1	-5.1	
	FUND. BIODIVER	113680	Unknown	Aegilops geniculata Roth	Roth	ESP	Spain	Renedo		Va	41.1	-3.1	
	FUND. BIODIVER	36855	Unknown	Aegilops geniculata Roth	Roth	ESP	Spain	Martioda		Vi	42.1	-2.1	500
00:00.0	REDIAM-CMA	28545	Observation	Aegilops geniculata		ESP	Spain		Antequera	Ma	36.9656	-4.75981	382
00:00.0	REDIAM-CMA	38404	Observation	Aegilops geniculata		ESP	Spain		Tarifa	Ca	36.1044	-5.73452	197
	W	42150	Unknown	Aegilops ovata L.		ESP	Spain	Broto, Aragon.					
1852-05-2	W	42405	Unknown	Aegilops ovata L.		ESP	Spain	Puerto de Segovia.					
	SIVIM	R-P09692:Aegi	Observation	Aegilops geniculata Roth	Roth	ES	Spain	entre Valdelcubo y Barahona			41.19	-2.76	1140
	SIVIM	R-P10554:Aegi	Observation	Aegilops geniculata Roth	Roth	ES	Spain	El Saltillo , AG			36.46	-5.9	0
	SIVIM	R-P11664:Aegi	Observation	Aegilops geniculata Roth	Roth	ES	Spain	Tarifa			44.93	-6.04	0
	SIVIM	S-P01257:Aegi	Observation	Aegilops geniculata Roth	Roth	ES	Spain	Fulleda, afores del poble			41.44	0.96	0
	SIVIM	S-P03973:Aegi	Observation	Aegilops geniculata Roth	Roth	ES	Spain	l'Espluga Calba, coll de Portelles			41.44	0.96	0
00:00.0	SEV	82968-1	Specimen	Aegilops ovata L.	L.	ES	Spain	Castellar de la Frontera. Pantano de		Ca			1
00:00.0	SEV	98677-1	Specimen	Aegilops geniculata Roth	Roth	ES	Spain	Antequera. El Torcal		Ma			1
00:00.0	ABH	8994-1	Specimen	Aegilops geniculata Roth	Roth	ES	Spain	Villena; Bco. Boqueres		A	38.58	-0.91	
00:00.0	MGC	11816-1	Unknown	Aegilops geniculata Roth	Roth	ES	Spain	Sierra Blanca. Carretera de OjÃ©n		Ma	0	0	1
00:00.0	ABH	15911-1	Specimen	Aegilops geniculata Roth	Roth	ES	Spain	La Romana; Sierra del Reclot, Cava		A	38.39	-0.93	
	FUND. BIODIVER	1463977	Unknown	Aegilops geniculata Roth	Roth	ESP	Spain	Peralba		L	41.1	0.1	780
	FUND. BIODIVER	1476047	Unknown	Aegilops geniculata Roth.	Roth.	ESP	Spain	Sierra de Santa Pola, Alicante, hast		A	37.1	-0.1	
	FUND. BIODIVER	913754	Unknown	Aegilops ovata L.	L.	ESP	Spain	Massif de St. LlorenÃ§		B	41.1	1.1	
	FUND. BIODIVER	918146	Unknown	Aegilops ovata var. ambigua Vay	Vayr.	ESP	Spain	Montserrat		B	41.1	1.1	
00:00.0	ABH	33337-1	Specimen	Aegilops geniculata Roth	Roth	ES	Spain	Sant Josep; pr. Cala de Comte		Ib	38.96	1.22	
00:00.0	REDIAM-CMA	162405	Observation	Aegilops geniculata		ESP	Spain		Espiel	Co	38.1641	-5.10323	600
00:00.0	MGC	72818-1	Unknown	Aegilops geniculata Roth	Roth	ES	Spain	Casares; Camino de Los Pobres		Ma	36.442	-5.315	190
	SIVIM	T-P06435:Aegi	Observation	Aegilops geniculata Roth	Roth	ES	Spain	Cheste			39.45	-0.79	250
	SIVIM	T-P07214:Aegi	Observation	Aegilops geniculata Roth	Roth	ES	Spain	Fraga			41.33	0.13	300
	SIVIM	T-P09164:Aegi	Observation	Aegilops geniculata Roth	Roth	ES	Spain	Tudela de Duero			41.53	-4.67	0
	SIVIM	T-P09370:Aegi	Observation	Aegilops geniculata Roth	Roth	ES	Spain	PontÃ³n de la Oliva			40.83	-3.47	0

	SIVIM	T-P11407:Aegi	Observation	Aegilops geniculata Roth	Roth	ES	Spain	Tragacete			40.28	-1.82	1240
	SIVIM	T-P13161:Aegi	Observation	Aegilops geniculata Roth	Roth	ES	Spain	Aibar			42.53	-1.41	0
00:00.0	BC	873754	Specimen	Aegilops ovata L.	L.	ES	Spain	Vallirana; Vallirana, les Casetes de		B	41.41	1.86	
	IDBD-GN	42421	Observation	Aegilops geniculata Roth	Roth	ES	Spain	Puerto de Iso	Foz de ArbayÃºn-	Na	42.6619	-1.23204	
	IDBD-GN	42434	Observation	Aegilops geniculata Roth	Roth	ES	Spain	Ostiz	Odieta	Na	42.9148	-1.61074	
	IDBD-GN	42443	Observation	Aegilops geniculata Roth	Roth	ES	Spain	Asiain	Olza	Na	42.8498	-1.83861	
00:00.0	SALA	57788-1	Specimen	Aegilops geniculata Roth	Roth	ES	Spain	_; Garraf		B	41.19	1.81	
00:00.0	SALA	60050-1	Specimen	Aegilops ovata L.	L.	ES	Spain	_; Villar del Buey		Za			
00:00.0	MA	753633-1	Specimen	Aegilops ovata L.	L.	ES	Spain	Collado del 'Tio Chaquetas'		Gr			
	FUND. BIODIVER	1648481	Unknown	Aegilops geniculata Roth	Roth	ESP	Spain	Iglesuela del Cid, pista arriba del Ma		Te	40.1	-0.1	
	FUND. BIODIVER	1700456	Unknown	Aegilops geniculata		ESP	Spain	Iglesuela del Cid, la Pobla del Belle		Te	40.1	-0.1	1115
00:00.0	REDIAM-CMA	244161	Observation	Aegilops geniculata		ESP	Spain		Arenas del Rey	Gr	36.9841	-3.86531	945
00:00.0	REDIAM-CMA	257287	Observation	Aegilops geniculata		ESP	Spain		Turre	Al	37.0844	-1.93552	856
00:00.0	REDIAM-CMA	277396	Observation	Aegilops geniculata		ESP	Spain		Dos Torres	Co	38.4647	-4.93768	563
	BDBCV-General	74112	Observation	Aegilops geniculata		ESPA	Spain	Ludiente	El Alto Mijares	Castel	40.1246	-0.35957	
	BDBCV-General	76807	Observation	Aegilops geniculata		ESPA	Spain	El Toro	El Alto Palancia	Castel	39.952	-0.71736	
00:00.0	COFC	11990-1	Specimen	Aegilops geniculata Roth	Roth	ES	Spain	Priego de CÃƒÂ³rdoba; la TiÃƒÂ±os		Co	37	-4	1
	SIVIM	T-P30030:Aegi	Observation	Aegilops geniculata Roth	Roth	ES	Spain	Bajada a Villa de Ves, Villa de Ves, Albace			39.19	-1.26	0
	SIVIM	T-P30055:Aegi	Observation	Aegilops geniculata Roth	Roth	ES	Spain	El Campichuelo, Cofrentes, Valencia			39.19	-1.14	0
	SIVIM	T-P31969:Aegi	Observation	Aegilops geniculata Roth	Roth	ES	Spain	Entre Pico MÃ¡gina-AlmadÃ©n			38.74	-4.84	1640
	SIVIM	U-P02802:Aegi	Observation	Aegilops geniculata Roth	Roth	ES	Spain	Castells de Serrella			38.63	-0.24	0
	SIVIM	U-P06639:Aegi	Observation	Aegilops geniculata Roth	Roth	ES	Spain	Amendoeira			37.13	-7.98	220
	SIVIM	U-P06724:Aegi	Observation	Aegilops geniculata Roth	Roth	ES	Spain	Rocha de Messines			37.22	-8.32	240
00:00.0	COA	41203-1	Specimen	Aegilops geniculata Roth	Roth	ES	Spain	Posadas		Co	37.75	-5.16	
00:00.0	COFC	41147-1	Specimen	Aegilops geniculata Roth	Roth	ES	Spain	Lucena; arroyo Salado; entre el mur		Co	37	-4	1
00:00.0	ABH	23283-1	Specimen	Aegilops geniculata Roth	Roth	ES	Spain	Vall de Gallinera; Castillo de Galline		A	38.83	-0.19	
	ADIMAN	35	Observation	Aegilops geniculata		ESP	Spain	EnguÃ-danos		CU	39.6775	-1.69292	
00:00.0	UNEX	30111-1	Observation	Aegilops geniculata Roth	_	ESP	Spain	Magacela: Olivar sobre calizas. 395		Ba	38.8	-5.8	
00:00.0	BDBCV	597843	Observation	Aegilops geniculata		ESP	Spain	Parc Natural de la	Alcoy	Alicant	38.728	-0.509	
	FUND. BIODIVER	1339635	Unknown	Aegilops geniculata Roth.	Roth.	ESP	Spain	Albacete, provincia		Ab	38.1	-2.1	
	FUND. BIODIVER	70870	Unknown	Aegilops ovata L.	L.	ESP	Spain	Villaverde de GuareÃ±a		Sa	40.1	-5.1	
	FUND. BIODIVER	78964	Unknown	Aegilops ovata L.	L.	ESP	Spain	Aldealengua		Sa	40.1	-5.1	
	FUND. BIODIVER	902845	Unknown	Aegilops ovata L.	L.	ESP	Spain	Prats de LlusanÃ©s, sitios estÃ©rile		Ge	41.1	1.1	
00:00.0	GDA	GDA10273-1-1	Specimen	Aegilops geniculata Roth.	Roth.	ES	Spain	Granada, Ãbor, camino de LanjarÃ		GR			600
00:00.0	REDIAM-CMA	83092	Observation	Aegilops geniculata		ESP	Spain		Aracena	H	37.8168	-6.58857	548
00:00.0	SEV	71064-1	Specimen	Aegilops geniculata Roth	Roth	ES	Spain	Los Barrios. El Tiradero. Quejigar		Ca			180
	SIVIM	S-P14150:Aegi	Observation	Aegilops geniculata Roth	Roth	ES	Spain	Serrinha			38.39	-8.54	0
	SIVIM	T-P03837:Aegi	Observation	Aegilops geniculata Roth	Roth	ES	Spain	4 km al W de Majadahonda			40.46	-3.94	0
	SIVIM	T-P04174:Aegi	Observation	Aegilops geniculata Roth	Roth	ES	Spain	1 km al NE de Villamayor			41.62	-0.83	300
	SIVIM	T-P06409:Aegi	Observation	Aegilops geniculata Roth	Roth	ES	Spain	Mogente			38.82	-0.81	0
00:00.0	COFC	46896-1	Specimen	Aegilops geniculata Roth	Roth	ES	Spain	Posadas; ctra de Villaviciosa de CÃ		Co			1

	FUND. BIODIVER	1025033	Unknown	Aegilops ovata		ESP	Spain	MengÃbar, BailÃ©n		J	37.1	-3.1	
00:00.0	FUND. BIODIVER	1774958	Unknown	Aegilops geniculata Roth	Roth	ESP	Spain	Monzon		Hu	41.1	0.1	320
	FUND. BIODIVER	1811877	Unknown	Aegilops geniculata Roth.	Roth.	ESP	Spain	Castillonuevo, Barranco de la Sierra		Na	42.1	-0.1	1000
	IDBD-GN	42419	Observation	Aegilops geniculata Roth	Roth	ES	Spain	El PerdÃ³n	Galar	Na	42.7585	-1.71811	
	IDBD-GN	42428	Observation	Aegilops geniculata Roth	Roth	ES	Spain	Zandio	OlÃ¡ibar	Na	42.9057	-1.59869	
	IDBD-GN	42440	Observation	Aegilops geniculata Roth	Roth	ES	Spain		Milagro	Na	42.2183	-1.72911	
00:00.0	REDIAM-CMA	331382	Observation	Aegilops geniculata		ESP	Spain		Villanueva del Re	Co	38.2241	-5.15149	486
00:00.0	REDIAM-CMA	382443	Observation	Aegilops geniculata		ESP	Spain				37.7919	-3.25425	
00:00.0	REDIAM-CMA	383165	Observation	Aegilops geniculata		ESP	Spain		Torres	J	37.7807	-3.49506	1133
	BDBCV-General	89865	Observation	Aegilops geniculata		ESPA	Spain				40.6588	0.102069	
00:00.0	BDBCV	599154	Observation	Aegilops geniculata		ESP	Spain		Alcoy	Alicant	38.655	-0.489	
	FUND. BIODIVER	1463967	Unknown	Aegilops geniculata Roth	Roth	ESP	Spain	Les Avellanes		L	41.1	0.1	600
	SIVIM	U-P08146:Aegi	Observation	Aegilops geniculata Roth	Roth	ES	Spain	Dehesa Boyal, LogrosÃ¡n			39.27	-5.55	0
	SIVIM	U-P13294:Aegi	Observation	Aegilops geniculata Roth	Roth	ES	Spain	En Ctra. Arjonilla-Marmolejo, Km. 7			37.94	-4.13	280
00:00.0	REDIAM-CMA	131295	Observation	Aegilops geniculata		ESP	Spain		Constantina	Se	37.9145	-5.71779	442
00:00.0	REDIAM-CMA	155746	Observation	Aegilops geniculata		ESP	Spain		Zalamea la Real	H	37.6196	-6.71556	349
00:00.0	REDIAM-CMA	170662	Observation	Aegilops geniculata		ESP	Spain		Espiel	Co	38.1696	-5.02417	596
00:00.0	FCO	24499-1	Specimen	Aegilops ovata L.	L.	ES	Spain	Olmedo; Olmedo		Va			
	SIVIM	T-P06962:Aegi	Observation	Aegilops geniculata Roth	Roth	ES	Spain	Ardoncino			42.42	-5.67	0
	SIVIM	T-P09152:Aegi	Observation	Aegilops geniculata Roth	Roth	ES	Spain	Cogeces de Iscar			41.45	-4.55	0
	SIVIM	T-P09350:Aegi	Observation	Aegilops geniculata Roth	Roth	ES	Spain	TorrebeleÃ±a			40.83	-3.23	0
	SIVIM	T-P10775:Aegi	Observation	Aegilops geniculata Roth	Roth	ES	Spain	Puente Quintos			41.78	-5.88	0
	SIVIM	T-P11868:Aegi	Observation	Aegilops geniculata Roth	Roth	ES	Spain	ArdÃ³n			42.42	-5.67	0
	SIVIM	T-P13214:Aegi	Observation	Aegilops geniculata Roth	Roth	ES	Spain	De Burgi a NavascuÃ©s, pto. Coronas			42.7	-1.16	0
00:00.0	FUND. BIODIVER	1684421	Unknown	Aegilops ovata L.	L.	ESP	Spain	El Bosque, junto campo de futbol		Ca	36.1	-5.1	
	FUND. BIODIVER	1696519	Unknown	Aegilops geniculata Roth	Roth	ESP	Spain	Iglesuela del Cid, pista arriba del ma		Te	40.1	-0.1	1500
	FUND. BIODIVER	1700269	Unknown	Aegilops geniculata		ESP	Spain	Vilafranca, Masia de la Rambla		Te	40.1	-0.1	1140
00:00.0	BC	70806	Specimen	Aegilops geniculata Roth	Roth	ES	Spain	Lanteira; in Sierra Nevada: Lanteria		Gr	37.18	-3.17	1400
00:00.0	COA	6151-1	Specimen	Aegilops geniculata Roth	Roth	ES	Spain	Lucena, Ermita de Nuestra SeÃ±ora		Co	37.38	-4.47	
00:00.0	REDIAM-CMA	264724	Observation	Aegilops geniculata		ESP	Spain		Cazorla	J	37.9096	-2.91996	1175
00:00.0	REDIAM-CMA	274492	Observation	Aegilops geniculata		ESP	Spain		Valsequillo	Co	38.5051	-5.42837	495
00:00.0	ABH	50264-1	Specimen	Aegilops geniculata Roth	Roth	ES	Spain	Jijona; puerto de la Carrasqueta		A	38.61	-0.48	
00:00.0	ABH	5169-1	Specimen	Aegilops geniculata Roth	Roth	ES	Spain	AlcalÃ¡ de los Gazules; a 3 km haci		Ca			
	SIVIM	T-P30044:Aegi	Observation	Aegilops geniculata Roth	Roth	ES	Spain	El Carril, Balsa de Ves, Albacete			39.19	-1.26	0
	SIVIM	T-P30069:Aegi	Observation	Aegilops geniculata Roth	Roth	ES	Spain	Puente del rÃo Cabriel, Cofrentes, Valenc			39.19	-1.14	335
	SIVIM	U-P02177:Aegi	Observation	Aegilops geniculata Roth	Roth	ES	Spain	E, PuÃ©rtolas, refugio de cueva Foratata			42.59	0.07	2010
	SIVIM	U-P02914:Aegi	Observation	Aegilops geniculata Roth	Roth	ES	Spain	Castells de Serrella			38.72	-0.23	0
	SIVIM	U-P06655:Aegi	Observation	Aegilops geniculata Roth	Roth	ES	Spain	Morgado de Apra			37.13	-7.98	2700
	SIVIM	U-P08058:Aegi	Observation	Aegilops geniculata Roth	Roth	ES	Spain	La Nava, Berzocana			39.36	-5.55	0
00:00.0	SEV	99215-1	Specimen	Aegilops geniculata Roth	Roth	ES	Spain	Coria del RÃo. Finca La Jampa		Se			1
00:00.0	MA	569167-1	Specimen	Aegilops geniculata Roth	Roth	ES	Spain	Aldeayuso		Va			

	IDBD-GN	42459	Observation	Aegilops geniculata Roth	Roth	ES	Spain		MaÃ±eru	Na	42.6697	-1.84197	
	IDBD-GN	42503	Observation	Aegilops geniculata Roth	Roth	ES	Spain	Foz de Benasa	Navascues	Na	42.709	-1.07807	870
	FUND. BIODIVER	1043758	Unknown	Aegilops geniculata Roth	Roth	ESP	Spain	Mollina		Ma	36.1	-4.1	
	FUND. BIODIVER	1072129	Unknown	Aegilops ovata L.	L.	ESP	Spain	MorÃ³n de la Frontera, alrededores		Se	36.1	-5.1	
	FUND. BIODIVER	1835087	Unknown	Aegilops geniculata Roth	Roth	ESP	Spain	Valle de Alcudia		CR			
00:00.0	FUND. BIODIVER	1835088	Unknown	Aegilops geniculata Roth	Roth	ESP	Spain	Albadalejo		CR	38.1	-2.1	900
00:00.0	HSS	240	Specimen	Aegilops geniculata Roth	Roth	ES	Spain	Alconera		Ba	38.4149	-6.42321	
00:00.0	REDIAM-CMA	384909	Observation	Aegilops geniculata		ESP	Spain		Olvera	Ca	36.9542	-5.17758	670
00:00.0	REDIAM-CMA	387977	Observation	Aegilops geniculata		ESP	Spain		Huelma	J	37.7108	-3.46739	1670
00:00.0	REDIAM-CMA	389522	Observation	Aegilops geniculata		ESP	Spain		Carcabuey	Co	37.448	-4.30181	1000
	SIVIM	P-P08872:Aegi	Observation	Aegilops geniculata Roth	Roth	ES	Spain	Mola de CatÃ-; Tortosa			40.79	0.27	1310
	SIVIM	P-P08923:Aegi	Observation	Aegilops geniculata Roth	Roth	ES	Spain	Plana de la Refoia; PaÃ¼ls			40.89	0.38	0
	SIVIM	P-P09744:Aegi	Observation	Aegilops geniculata Roth	Roth	ES	Spain	Barranc del Grevolar; Arnes			40.79	0.27	0
	SIVIM	P-P12682:Aegi	Observation	Aegilops geniculata Roth	Roth	ES	Spain	Cerca de la Sierra de las Estancias			37.58	-2.09	0
1876-04-1	W	42323	Unknown	Aegilops ovata L.		ESP	Spain	In collibus prope Algeciras. In Itinere hispanico-lustianico.					
	ADIMAN	19	Observation	Aegilops geniculata		ESP	Spain	EnguÃ-danos		CU	39.6243	-1.60257	
	CZE122	01C2109112	Specimen	Aegilops geniculata Roth		ESP	Spain	W.-Pyrenaeen, b. Jaca, 810 m					
00:00.0	COA	41167-1	Specimen	Aegilops geniculata Roth	Roth	ES	Spain	Cabra, cerca de casilla Buenavista		Co	37.4	-4.47	
	BDBCV-General	34268	Observation	Aegilops geniculata		ESPA	Spain	Agres	El Comtat	Alicant	38.7769	-0.52512	
00:00.0	MGC	56526-1	Unknown	Aegilops geniculata Roth	Roth	ES	Spain	MÃ¡laga; FÃ¡brica de Cemento. Zor		Ma	36.719	-4.316	1
00:00.0	GDA	GDA30010-1	Specimen	Aegilops ovata L.	L.	ES	Spain	JaÃ©n, SÂª MÃ¡gina, MojÃ³n Blanc		J			1400
	ADIMAN	27	Observation	Aegilops geniculata		ESP	Spain	EnguÃ-danos		CU	39.6591	-1.57412	
00:00.0	SALA	16092-1	Specimen	Aegilops ovata L. subsp. triaristat	(Willd.) Rouy	ES	Spain	_; San Felices de los Gallegos		Sa			
00:00.0	SEV	9868-1	Specimen	Aegilops ovata L.	L.	ES	Spain	Sierra Blanca		Ma			1020
00:00.0	SEV	23822-1	Specimen	Aegilops ovata L.	L.	ES	Spain	La Mola, O-Seite, Wegrand nahe de		Fo			120
	RUS001	VIR100602125	Specimen	Aegilops ovata L.		ESP	Spain						
	RUS001	VIR100602124	Specimen	Aegilops ovata L.		ESP	Spain						
	RUS001	VIR100602103	Specimen	Aegilops ovata L.		ESP	Spain						
	RUS001	VIR100602082	Specimen	Aegilops ovata L.		ESP	Spain						
	FUND. BIODIVER	1073156	Unknown	Aegilops ovata		ESP	Spain	Baena		Co	37.1	-4.1	
	FUND. BIODIVER	1093218	Unknown	Aegilops ovata L.	L.	ESP	Spain	Gredos		Cc	40.1	-5.1	
	FUND. BIODIVER	1093239	Unknown	Aegilops geniculata		ESP	Spain	Cerro de Aldeamoret		Cc	39.1	-6.1	
	FUND. BIODIVER	1946403	Unknown	Aegilops geniculata Roth	Roth	ESP	Spain	Valle de Alcudia		CR			
00:00.0	COFC	25324-1	Specimen	Aegilops geniculata Roth	Roth	ES	Spain	Constantina; carretera a Las Navas		Se			1
00:00.0	COFC	28810-1	Specimen	Aegilops geniculata Roth	Roth	ES	Spain	Andujar; parque natural; coto 'Sella		J			1
00:00.0	REDIAM-CMA	3871	Observation	Aegilops geniculata		ESP	Spain		Huelma	J	37.6765	-3.46964	1149
00:00.0	REDIAM-CMA	13694	Observation	Aegilops geniculata		ESP	Spain		Lora del RÃ-o	Se	37.6814	-5.5688	180
00:00.0	REDIAM-CMA	19892	Observation	Aegilops geniculata		ESP	Spain		Cambil	J	37.7326	-3.50659	1627
00:00.0	REDIAM-CMA	403181	Observation	Aegilops geniculata		ESP	Spain		Villanueva de la F	J	38.3366	-3.86157	694
00:00.0	REDIAM-CMA	409780	Observation	Aegilops geniculata		ESP	Spain		Segura de la Sier	J	38.021	-2.65598	1512
00:00.0	REDIAM-CMA	416767	Observation	Aegilops geniculata		ESP	Spain		Tabernas	Al	37.0748	-2.34735	452

00:00.0	REDIAM-CMA	421464	Observation	Aegilops geniculata		ESP	Spain		Felix	Al	36.9051	-2.72227	1399
00:00.0	BCN	BCN-S 21	Specimen	Aegilops ovata L.	L.	EspaÃ	Spain	Marge de l'Embassament de Les Es		LERIDA			
00:00.0	COFC	36346-1	Specimen	Aegilops geniculata Roth	Roth	ES	Spain	Rute; Camorro de la Isla		Co			1
	SIVIM	Q-P03044:Aegi	Observation	Aegilops geniculata Roth	Roth	ES	Spain	La Llacuna, part baixa del camÃ- de la Fon			41.45	1.44	0
	SIVIM	Q-P03609:Aegi	Observation	Aegilops geniculata Roth	Roth	ES	Spain	Argelita			39.99	-0.42	0
	SIVIM	Q-P06903:Aegi	Observation	Aegilops geniculata Roth	Roth	ES	Spain	Batech, Petrer,			38.46	-0.82	0
	SIVIM	R-P02649:Aegi	Observation	Aegilops geniculata Roth	Roth	ES	Spain	rodalies de Torres de Segre			41.52	0.48	160
	SIVIM	R-P06793:Aegi	Observation	Aegilops geniculata Roth	Roth	ES	Spain	Casas de Juan NÃºÃ±ez, hacia Jorquera 7			39.11	-1.61	0
	SIVIM	R-P08448:Aegi	Observation	Aegilops geniculata Roth	Roth	ES	Spain	CaparidÃ¡n			36.86	-3	1600
	RUS001	VIR100602110	Specimen	Aegilops ovata L.		ESP	Spain						
	RUS001	VIR100602089	Specimen	Aegilops ovata L.		ESP	Spain						
	FUND. BIODIVER	1082854	Unknown	Aegilops ovata		ESP	Spain	ChinchÃ³n		M	39.1	-3.1	
	FUND. BIODIVER	1925489	Unknown	Aegilops geniculata Roth	Roth	ESP	Spain	Itoiz		Na	42.1	-1.1	510
	FUND. BIODIVER	1931162	Unknown	Aegilops geniculata		ESP	Spain	Garrigas de Son Serra de Marina		PM	39.1	2.1	
	FUND. BIODIVER	1946396	Unknown	Aegilops geniculata Roth	Roth	ESP	Spain	Tablas de Daimiel, isla del Pan		CR	38.1	-3.1	
00:00.0	COFC	41155-1	Specimen	Aegilops geniculata Roth	Roth	ES	Spain	Lucena; rÃƒÂ-o Anzur; entre Lucena		Co	37	-4	1
00:00.0	COFC	41293-1	Specimen	Aegilops geniculata Roth	Roth	ES	Spain	Lucena; carretera entre el municipio		Co	37	-4	1
00:00.0	UNEX	10370-1	Observation	Aegilops geniculata Roth	_	ESP	Spain	Olivenza: Pantano de Piedra Aguda		Ba	38.7	-7.2	
00:00.0	UNEX	10372-1	Observation	Aegilops geniculata Roth	_	ESP	Spain	Hornachos: Sra. de Hornachos. 29		Ba	38.5	-6.00001	
	ADIMAN	31	Observation	Aegilops geniculata		ESP	Spain	EnguÃ-danos		CU	39.6637	-1.57665	
	ADIMAN	34	Observation	Aegilops geniculata		ESP	Spain	EnguÃ-danos		CU	39.6775	-1.69292	
00:00.0	REDIAM-CMA	9555	Observation	Aegilops geniculata		ESP	Spain		Jimena de la Fron	Ca	36.4452	-5.44555	97
00:00.0	REDIAM-CMA	17482	Observation	Aegilops geniculata		ESP	Spain		Medina-Sidonia	Ca	36.4233	-5.95815	99
00:00.0	REDIAM-CMA	24608	Observation	Aegilops geniculata		ESP	Spain		Jimena	J	37.8179	-3.46986	829
	REDIAM-CMA	396533	Observation	Aegilops geniculata		ESP	Spain		Cortes de la Fron	Ma	36.566	-5.43678	860
00:00.0	REDIAM-CMA	401137	Observation	Aegilops geniculata		ESP	Spain		Santa Olalla del C	H	37.9019	-6.1926	510
00:00.0	REDIAM-CMA	405365	Observation	Aegilops geniculata		ESP	Spain		Villacarrillo	J	38.0666	-2.93749	1282
00:00.0	REDIAM-CMA	415554	Observation	Aegilops geniculata		ESP	Spain		Cazorla	J	37.822	-2.85862	1400
00:00.0	REDIAM-CMA	419311	Observation	Aegilops geniculata		ESP	Spain		Padul	Gr	36.9879	-3.74211	1199
	SIVIM	Q-P03035:Aegi	Observation	Aegilops geniculata Roth	Roth	ES	Spain	La Llacuna, carrer no pavimentat que va de			41.45	1.44	0
	SIVIM	Q-P03602:Aegi	Observation	Aegilops geniculata Roth	Roth	ES	Spain	Villahermosa			40.17	-0.41	0
	SIVIM	Q-P06538:Aegi	Observation	Aegilops geniculata Roth	Roth	ES	Spain	Els Estepars, entre Prades i Albarca			41.26	0.85	0
	SIVIM	Q-P09164:Aegi	Observation	Aegilops geniculata Roth	Roth	ES	Spain	Portugal			39.2	-7.72	202
	SIVIM	R-P04059:Aegi	Observation	Aegilops geniculata Roth	Roth	ES	Spain	Vic, Sant Jordi			41.9	2.27	0
	SIVIM	R-P08205:Aegi	Observation	Aegilops geniculata Roth	Roth	ES	Spain	Sota el coll dels Brucs, vers CastelloIÃ-			41.54	1.68	0
	FUND. BIODIVER	1463969	Unknown	Aegilops geniculata Roth	Roth	ESP	Spain	Vilanova de la Sal, salines		L	41.1	0.1	550
	FUND. BIODIVER	1463970	Unknown	Aegilops geniculata Roth	Roth	ESP	Spain	Tartareu		L	41.1	0.1	600
	FUND. BIODIVER	918138	Unknown	Aegilops ovata L.	L.	ESP	Spain	Garriga		B	41.1	2.1	
	FUND. BIODIVER	96901	Unknown	Aegilops geniculata Roth.	Roth.	ESP	Spain	Pereruela, San RomÃ¡n de los Infan		Za	41.1	-5.1	
	ESP004	NC044546	Specimen	Aegilops geniculata Roth		ESP	Spain	Es Mercadal, province of Baleares			39.9833	4.08333	96
00:00.0	REDIAM-CMA	157860	Observation	Aegilops geniculata		ESP	Spain		Espiel	Co	38.2182	-4.96802	661

00:00.0	REDIAM-CMA	171551	Observation	Aegilops geniculata		ESP	Spain		Santa Olalla del C	H	37.921	-6.21392	498
00:00.0	REDIAM-CMA	181957	Observation	Aegilops geniculata		ESP	Spain		Siles	J	38.3288	-2.54036	1231
	FUND. BIODIVER	1115793	Unknown	Aegilops geniculata Rothm.	Rothm.	ESP	Spain	Faramontanos de TÃ¡bara		Za	41.1	-5.1	
	FUND. BIODIVER	1120221	Unknown	Aegilops geniculata Roth	Roth	ESP	Spain	Aspariegos, Las Cabecinas		Za	41.1	-5.1	
	FUND. BIODIVER	1135161	Unknown	Aegilops geniculata Roth	Roth	ESP	Spain	Puente Villarente		Le	42.1	-5.1	754
	FUND. BIODIVER	51167	Unknown	Aegilops ovata subsp. triaristata	L.	ESP	Spain	Villares de Yeltes		Sa	40.1	-6.1	
00:00.0	GDA	GDA48832-1	Specimen	Aegilops geniculata Roth.	Roth.	ES	Spain	AlmerÃ-a, SÂª de los Filabres, Beni		AL			850
	SIVIM	T-P06964:Aegi	Observation	Aegilops geniculata Roth	Roth	ES	Spain	Castrofuerte			42.15	-5.54	0
	SIVIM	T-P09154:Aegi	Observation	Aegilops geniculata Roth	Roth	ES	Spain	Portillo			41.44	-4.67	0
	SIVIM	T-P09352:Aegi	Observation	Aegilops geniculata Roth	Roth	ES	Spain	Alpedrete de la Sierra			40.83	-3.47	0
	SIVIM	T-P10778:Aegi	Observation	Aegilops geniculata Roth	Roth	ES	Spain	Pozuelo de TÃ¡bara			41.78	-6	0
	SIVIM	T-P12076:Aegi	Observation	Aegilops geniculata Roth	Roth	ES	Spain	Ardoncino			42.42	-5.67	0
	SIVIM	T-P13216:Aegi	Observation	Aegilops geniculata Roth	Roth	ES	Spain	Alto de Lerga			42.53	-1.53	0
00:00.0	BC	632230	Specimen	Aegilops ovata L.	L.	ES	Spain	Vilanova del CamÃ-; Anoia: Vilanov		B	41.59	1.62	300
00:00.0	BC	830209	Specimen	Aegilops geniculata Roth	Roth	ES	Spain	Palma; Canal de les Quatre Quarter		PM	39.1192	2.884495	
00:00.0	REDIAM-CMA	36413	Observation	Aegilops geniculata		ESP	Spain		NÃ-jar	Al	36.8118	-2.09101	98
	SIVIM	R-P09471:Aegi	Observation	Aegilops geniculata Roth	Roth	ES	Spain	Muduex, zona superior del pÃ¡ramo, cuneta			40.83	-3	1020
	SIVIM	R-P10436:Aegi	Observation	Aegilops geniculata Roth	Roth	ES	Spain	La Peguera , AG			36.29	-5.67	0
	SIVIM	R-P11647:Aegi	Observation	Aegilops geniculata Roth	Roth	ES	Spain	Tarifa			44.93	-6.04	0
	SIVIM	S-P01245:Aegi	Observation	Aegilops geniculata Roth	Roth	ES	Spain	els Omells de Na Gaia, afores del poble			41.44	0.96	0
	SIVIM	S-P02019:Aegi	Observation	Aegilops geniculata Roth	Roth	ES	Spain	Santa Linya, vers l'estaciÃ³ de FFCC			41.89	0.83	0
	SIVIM	S-P03961:Aegi	Observation	Aegilops geniculata Roth	Roth	ES	Spain	l'Espluga Calba, els Graus			41.44	0.96	0
00:00.0	UNEX	10379-1	Observation	Aegilops geniculata Roth	_	ESP	Spain	Burguillos del Cerro: Berrocales. 2		Ba	38.4	-6.7	
	ESP004	NC010107	Specimen	Aegilops geniculata Roth		ESP	Spain	Palacios de La Sierra, province of Burgos			41.95	-3.11667	1040
	FUND. BIODIVER	1539227	Unknown	Aegilops ovata L.	L.	ESP	Spain	pte. de Castro		Le	42.1	-5.1	
	FUND. BIODIVER	1554599	Unknown	Aegilops ovata L.	L.	ESP	Spain	PeÃ±a Corada		Le	42.1	-4.1	
00:00.0	FUND. BIODIVER	1593497	Unknown	Aegilops geniculata		ESP	Spain	MuÃ±eca		P	42.1	-4.1	1180
	FUND. BIODIVER	998068	Unknown	Aegilops geniculata		ESP	Spain	Altos de Cabrejas		Cu	39.1	-2.1	
00:00.0	SALA	112291-1	Specimen	Aegilops geniculata Roth	Roth	ES	Spain	Renedo de Esgueva		Va	41.64	-4.61	
00:00.0	REDIAM-CMA	190413	Observation	Aegilops geniculata		ESP	Spain		San NicolÃ¡s del	Se	37.9566	-5.62419	650
00:00.0	REDIAM-CMA	224935	Observation	Aegilops geniculata		ESP	Spain		Ãtrabo	Gr	36.8248	-3.62137	887
00:00.0	BC	634095	Specimen	Aegilops ovata L.	L.	ES	Spain	Montgat; Montgat		B	41.5	2.34	
	SIVIM	T-P14685:Aegi	Observation	Aegilops geniculata Roth	Roth	ES	Spain	Amposta (Pla de Gallos)			40.62	0.51	0
	SIVIM	T-P16916:Aegi	Observation	Aegilops geniculata Roth	Roth	ES	Spain	Ciudad Universitaria			40.37	-3.7	0
	SIVIM	T-P19982:Aegi	Observation	Aegilops geniculata Roth	Roth	ES	Spain	Almaraz			39.8	-5.68	320
	SIVIM	T-P25937:Aegi	Observation	Aegilops geniculata Roth	Roth	ES	Spain	Prox. Torreperogil			38.03	-3.34	0
	SIVIM	T-P27630:Aegi	Observation	Aegilops geniculata Roth	Roth	ES	Spain	Pto. Cerezo (SÂª Horconera)			37.3	-4.35	1310
	SIVIM	T-P28647:Aegi	Observation	Aegilops geniculata Roth	Roth	ES	Spain	Colmenar del Negrete, Serradilla			39.71	-6.2	240
00:00.0	MA	595269-1	Specimen	Aegilops ovata L.	L.	ES	Spain			L			
00:00.0	COFC	41157-1	Specimen	Aegilops geniculata Roth	Roth	ES	Spain	Luque; cerro Juan MartÃƒÂ-n		Co	37	-4	1
00:00.0	COFC	41295-1	Specimen	Aegilops geniculata Roth	Roth	ES	Spain	Baena; cerro Valladolid		Co	37	-4	1

00:00.0	MGC	4346-1	Unknown	Aegilops ovata var. pubiglumis (J	(Jord. et Fou	ES	Spain	La Moncloa		M	0	0	1
00:00.0	SEV	98682-1	Specimen	Aegilops geniculata Roth	Roth	ES	Spain	Priego de CÃ³rdoba, camino cercan		Co			1
00:00.0	SEV	98930-1	Specimen	Aegilops geniculata Roth	Roth	ES	Spain	Calatayud. Sierra Bicor		Z			1
00:00.0	FUND. BIODIVER	1580531	Unknown	Aegilops geniculata		ESP	Spain	PeÃ±a Oracada		P			1150
00:00.0	BDBCV	106	Observation	Aegilops geniculata		ESP	Spain	Parque Natural de Penyagolosa		Cs	40.24	-0.39	
00:00.0	REDIAM-CMA	195946	Observation	Aegilops geniculata		ESP	Spain		Espiel	Co	38.1372	-5.031	589
00:00.0	REDIAM-CMA	203907	Observation	Aegilops geniculata		ESP	Spain		Constantina	Se	37.8959	-5.68627	608
00:00.0	REDIAM-CMA	219391	Observation	Aegilops geniculata		ESP	Spain		AlbuÃ±uelas	Gr	36.932	-3.60643	651
00:00.0	REDIAM-CMA	230569	Observation	Aegilops geniculata		ESP	Spain		DalÃ-as	Al	36.8785	-2.81529	1896
00:00.0	REDIAM-CMA	235597	Observation	Aegilops geniculata		ESP	Spain		Cortegana	H	37.7962	-6.96111	336
00:00.0	REDIAM-CMA	240852	Observation	Aegilops geniculata		ESP	Spain		Cumbres Mayores	H	38.0266	-6.71127	714
	ADIMAN	23	Observation	Aegilops geniculata		ESP	Spain	EnguÃ-danos		CU	39.6551	-1.5768	
	SIVIM	T-P13699:Aegi	Observation	Aegilops geniculata Roth	Roth	ES	Spain	MasÃ-a de la Loma, Iglesuela del Cid			40.44	-0.4	1320
	SIVIM	T-P16889:Aegi	Observation	Aegilops geniculata Roth	Roth	ES	Spain	ValdepiÃ©lagos			40.73	-3.47	0
	SIVIM	T-P19690:Aegi	Observation	Aegilops geniculata Roth	Roth	ES	Spain	GÃ©rgal, pr. al pueblo			37.04	-2.55	0
	SIVIM	T-P21026:Aegi	Observation	Aegilops geniculata Roth	Roth	ES	Spain	Prox. Finca La Beata, ValdepeÃ±as de JaÃ			37.58	-3.9	1120
	SIVIM	T-P28634:Aegi	Observation	Aegilops geniculata Roth	Roth	ES	Spain	La CaÃ±ada, Romangordo			39.71	-5.79	290
00:00.0	GDA	GDA16649-1-2	Specimen	Aegilops geniculata Roth	Roth	ES	Spain	Granada, SÂª de Parapanda, carret		GR			750
00:00.0	GDA	GDA10274-1-2	Specimen	Aegilops ovata L. pro parte	L. pro parte	ES	Spain	Granada, SÂª de Mecina, Pitres, fal		GR			1150
	IDBD-GN	42474	Observation	Aegilops geniculata Roth	Roth	ES	Spain		Lerga	Na	42.5535	-1.52135	750
	IDBD-GN	42485	Observation	Aegilops geniculata Roth	Roth	ES	Spain		Viana	Na	42.4985	-2.41105	450
	IDBD-GN	42507	Observation	Aegilops geniculata Roth	Roth	ES	Spain	Foz de Lumbier	LiÃ©dena	Na	42.6406	-1.31192	420
00:00.0	GDAC	GDAC35212-1	Specimen	Aegilops geniculata Roth.	Roth.	ES	Spain	AlmerÃ-a, Cuevas de Almanzora, p		AL			0
00:00.0	BC	96445	Specimen	Aegilops ovata L.	L.	ES	Spain	Barcelona; Can Gomis (Barcelona)		B	41.41	2.1	
00:00.0	UNEX	26487-1	Observation	Aegilops geniculata Roth	_	ESP	Spain	Magacela: Calizas de Magacela, su		Ba	38.8	-5.8	
00:00.0	BC	70801	Specimen	Aegilops ovata L.	L.	ES	Spain	la Cellera de Ter; Sorrals del Ter, s		Ge	41.96	2.58	
	ESP004	NC044553	Specimen	Aegilops geniculata Roth		ESP	Spain	Frailes, province of Jaen			37.4833	-3.83333	973
00:00.0	SALA	18839-1	Specimen	Aegilops ovata L.	L.	ES	Spain	_; Almenara de Tormes		Sa			
	FUND. BIODIVER	1141561	Unknown	Aegilops geniculata Roth	Roth	ESP	Spain	Ardoncino		Le	42.1	-5.1	830
	FUND. BIODIVER	116428	Unknown	Aegilops geniculata Roth.	Roth.	ESP	Spain	Almenara de Adaja		Va	41.1	-4.1	
	FUND. BIODIVER	36861	Unknown	Aegilops geniculata Roth	Roth	ESP	Spain	OyÃ³n		Vi	42.1	-2.1	450
	FUND. BIODIVER	54856	Unknown	Aegilops geniculata Roth	Roth	ESP	Spain	BÃ³veda del rÃ-o Almar		Sa	40.1	-4.1	
00:00.0	GDA	GDA48984-1	Specimen	Aegilops geniculata Roth	Roth	ES	Spain	AlmerÃ-a, SÂª de los Filabres, Beni		AL			800
00:00.0	REDIAM-CMA	64803	Observation	Aegilops geniculata		ESP	Spain		Loja	Gr	37.0786	-4.11425	1323
00:00.0	REDIAM-CMA	73584	Observation	Aegilops geniculata		ESP	Spain		AlcalÃ¡ de los Ga	Ca	36.4812	-5.74975	112
00:00.0	BC	830210	Specimen	Aegilops ovata L.	L.	ES	Spain	Palma de Mallorca; Cabrera, a sota		PM	39.16	2.94	
	IPK	AE 1345	Living	Aegilops geniculata Roth			Spain	Sudspanien: Andalusien, Sierranevada, ca			36.9164	-3.51667	
00:00.0	SEV	108246-1	Specimen	Aegilops geniculata Roth	Roth	ES	Spain	Priego de CÃ³rdoba. Sierra de Alba		Co			1
00:00.0	SEV	108282-1	Specimen	Aegilops geniculata Roth	Roth	ES	Spain	Carretra de CÃ³rdoba a Palma del F		Co			1
	SIVIM	R-P09414:Aegi	Observation	Aegilops geniculata Roth	Roth	ES	Spain	Alcolea del Pinar			41.01	-2.52	1200
	SIVIM	R-P10345:Aegi	Observation	Aegilops geniculata Roth	Roth	ES	Spain	La Joya , AlcalÃ¡ de los Gazules			36.47	-5.79	0

	SIVIM	R-P11631:Aegi	Observation	Aegilops geniculata Roth	Roth	ES	Spain	Jerez de la Frontera, Casas del Corchadillo			36.65	-6.2	0
	SIVIM	R-P11725:Aegi	Observation	Aegilops geniculata Roth	Roth	ES	Spain	Quintanilla de Trigueros			41.8	-4.68	0
	SIVIM	S-P01265:Aegi	Observation	Aegilops geniculata Roth	Roth	ES	Spain	l'Espluga Calba, els Graus			41.44	0.96	0
	SIVIM	S-P03953:Aegi	Observation	Aegilops geniculata Roth	Roth	ES	Spain	el Vilosell			41.35	0.84	0
00:00.0	MGC	45952-1	Unknown	Aegilops geniculata Roth	Roth	ES	Spain	Ronda; P. N. Sierra de las Nieves. F		Ma	36.691	-5.054	1330
	FUND. BIODIVER	1564672	Unknown	Aegilops geniculata		ESP	Spain	Hornillo de Cerrato		P	41.1	-4.1	830
	FUND. BIODIVER	998070	Unknown	Aegilops geniculata		ESP	Spain	Tragacete		Cu	40.1	-1.1	
00:00.0	COA	32826-1	Specimen	Aegilops geniculata Roth	Roth	ES	Spain	Rute, Llanos de Don Juan		Co	37.4	-4.47	
00:00.0	COA	28013-1	Specimen	Aegilops geniculata Roth	Roth	ES	Spain	Sierra MÃ¡gina, El Carluco		J	37.68	-3.45	
00:00.0	REDIAM-CMA	190448	Observation	Aegilops geniculata		ESP	Spain		Tabernas	Al	37.1121	-2.34253	578
00:00.0	SALA	59781-1	Specimen	Aegilops geniculata Roth	Roth	ES	Spain	_; Taravilla, Laguna de la Parra		Gu	40.65	-1.98	
	SIVIM	T-P16879:Aegi	Observation	Aegilops geniculata Roth	Roth	ES	Spain	HÃºmera			40.37	-3.82	0
	SIVIM	T-P16918:Aegi	Observation	Aegilops geniculata Roth	Roth	ES	Spain	Ciudad Universitaria			40.37	-3.7	0
	SIVIM	T-P20028:Aegi	Observation	Aegilops geniculata Roth	Roth	ES	Spain	Cuesta de Araya (Garrovillas)			39.63	-6.55	250
	SIVIM	T-P25943:Aegi	Observation	Aegilops geniculata Roth	Roth	ES	Spain	Prox. Jimena			37.76	-3.56	0
	SIVIM	T-P27680:Aegi	Observation	Aegilops geniculata Roth	Roth	ES	Spain	Sierra de la Lastra			37.48	-4.35	0
	SIVIM	T-P29800:Aegi	Observation	Aegilops geniculata Roth	Roth	ES	Spain	Las Arenillas, Alborea, Albacete			39.19	-1.49	0
00:00.0	SALA	60048-1	Specimen	Aegilops geniculata Roth	Roth	ES	Spain	_; Pereruela		Za			
00:00.0	SEV	61118-1	Specimen	Aegilops geniculata Roth	Roth	ES	Spain	Algodonales, Sierra de LÃ-jar		Ca			500
00:00.0	COFC	11976-1	Specimen	Aegilops geniculata Roth	Roth	ES	Spain	Priego de CÃƒÂ³rdoba; Sierra de Po		Co	37	-4	1
00:00.0	COFC	22145-1	Specimen	Aegilops geniculata Roth	Roth	ES	Spain	Sorbas; a 1,8 Km al este de 'Los Ca		Al			1
00:00.0	COFC	11952-1	Specimen	Aegilops geniculata Roth	Roth	ES	Spain	Priego de CÃ³rdoba; entre el munici		Co	37	-4	1
00:00.0	COFC	11988-1	Specimen	Aegilops geniculata Roth	Roth	ES	Spain	Rute; Sierra de Rute; pico las Cruce		Co	37	-4	1
	RUS001	VIR100602209	Specimen	Aegilops ovata L.		ESP	Spain						
	ADIMAN	25	Observation	Aegilops geniculata		ESP	Spain	EnguÃ-danos		CU	39.6574	-1.57234	
	FUND. BIODIVER	1036794	Unknown	Aegilops ovata L.	L.	ESP	Spain	Sierra Almijara		Ma	36.1	-3.1	
	FUND. BIODIVER	1043762	Unknown	Aegilops geniculata Roth	Roth	ESP	Spain	San Pedro de AlcÃ¡ntara		Ma	36.1	-4.1	
	FUND. BIODIVER	1050669	Unknown	Aegilops geniculata Roth	Roth	ESP	Spain	Sierras de Algeciras		Ca	35.1	-5.1	
	FUND. BIODIVER	1067621	Unknown	Aegilops ovata		ESP	Spain	Puebla del RÃ-o		Se	37.1	-5.1	
	FUND. BIODIVER	1073153	Unknown	Aegilops geniculata		ESP	Spain	Puente Genil		Co	37.1	-4.1	
	FUND. BIODIVER	1835070	Unknown	Aegilops geniculata Roth	Roth	ESP	Spain	Despenaperros		CR	38.1	-3.1	
	FUND. BIODIVER	1891038	Unknown	Aegilops geniculata Roth	Roth	ESP	Spain	Formentera		PM	38.1	1.1	
00:00.0	REDIAM-CMA	385280	Observation	Aegilops geniculata		ESP	Spain		AlcalÃ¡ del Valle	Ca	36.9471	-5.10383	600
00:00.0	REDIAM-CMA	388258	Observation	Aegilops geniculata		ESP	Spain		Torres	J	37.7455	-3.53911	1393
00:00.0	REDIAM-CMA	394128	Observation	Aegilops geniculata		ESP	Spain		Chirivel	Al	37.6432	-2.21357	1256
00:00.0	BC	92737	Specimen	Aegilops geniculata Roth	Roth	ES	Spain	Dos Hermanas; Dos Hermanas		Se	37.33	-5.88	
	SIVIM	P-P08511:Aegi	Observation	Aegilops geniculata Roth	Roth	ES	Spain	Pinar Pla; Fredes			40.7	0.15	1190
	SIVIM	P-P08876:Aegi	Observation	Aegilops geniculata Roth	Roth	ES	Spain	CoratxÃ , mola			40.61	0.04	1250
	SIVIM	P-P09001:Aegi	Observation	Aegilops geniculata Roth	Roth	ES	Spain	Moleta d'Alfara; Alfara			40.89	0.38	0
	SIVIM	P-P11088:Aegi	Observation	Aegilops geniculata Roth	Roth	ES	Spain	Matarredona, Sant Carles de la RÃ pita			40.62	0.51	0
	SIVIM	P-P12759:Aegi	Observation	Aegilops geniculata Roth	Roth	ES	Spain	Chirivel			37.58	-2.32	1020

00:00.0	MA	772793-1	Specimen	Aegilops geniculata Roth.	Roth.	ES	Spain	Encinas Reales; Ventas del RÃ-o A		Co			
	FUND. BIODIVER	1050671	Unknown	Aegilops ovata		ESP	Spain	Sierra de Ubrique		Ca	36.1	-5.1	
00:00.0	FUND. BIODIVER	1835072	Unknown	Aegilops geniculata Roth	Roth	ESP	Spain	Herencia, de Herencia a Villarta de		CR	39.1	-3.1	700
	FUND. BIODIVER	1891040	Unknown	Aegilops geniculata Roth	Roth	ESP	Spain	Formentera		PM	38.1	1.1	
00:00.0	REDIAM-CMA	384089	Observation	Aegilops geniculata		ESP	Spain				37.6694	-3.41946	
00:00.0	REDIAM-CMA	385385	Observation	Aegilops geniculata		ESP	Spain		Ronda	Ma	36.8604	-5.11114	758
00:00.0	REDIAM-CMA	386789	Observation	Aegilops geniculata		ESP	Spain		Cambil	J	37.7288	-3.4911	1581
00:00.0	REDIAM-CMA	388402	Observation	Aegilops geniculata		ESP	Spain		Cambil	J	37.7341	-3.52508	1963
00:00.0	REDIAM-CMA	394393	Observation	Aegilops geniculata		ESP	Spain		Hornos	J	38.1801	-2.64656	1430
	DEU146	AE 356	Specimen	Aegilops geniculata Roth subsp. gibberosa (Zh		ESP	Spain	Formentor, Majorque, Baleares					
	SIVIM	P-P08526:Aegi	Observation	Aegilops geniculata Roth	Roth	ES	Spain	Barranc dels Cirers; la SÃ©nia			40.7	0.15	1070
	SIVIM	P-P08878:Aegi	Observation	Aegilops geniculata Roth	Roth	ES	Spain	Barranc dels Cirers; la SÃ©nia			40.7	0.15	1070
	SIVIM	P-P09007:Aegi	Observation	Aegilops geniculata Roth	Roth	ES	Spain	Les Foies; Alfara			40.79	0.27	1140
	SIVIM	P-P11090:Aegi	Observation	Aegilops geniculata Roth	Roth	ES	Spain	Finca de l''Abogat'			40.62	0.39	0
	SIVIM	P-P12763:Aegi	Observation	Aegilops geniculata Roth	Roth	ES	Spain	Chirivel			37.58	-2.32	1040
	ESP004	NC027434	Specimen	Aegilops geniculata Roth		ESP	Spain	Polop, province of Alicante			38.6167	-0.11667	236
00:00.0	MUB	102380-1	Specimen	Aegilops geniculata Roth	Roth	ES	Spain	Cartagena; Vaguada de Las Carras		Mu	37.622	-1.159	270
00:00.0	BDBCV	597938	Observation	Aegilops geniculata		ESP	Spain		Alcoy	Alicant	38.728	-0.498	
	FUND. BIODIVER	1340283	Unknown	Aegilops geniculata Roth	Roth	ESP	Spain	FrÃ-as		Bu	42.1	-3.1	530
	FUND. BIODIVER	70865	Unknown	Aegilops ovata L.	L.	ESP	Spain	Castellanos de Villiquera, arroyo de		Sa	40.1	-5.1	
	FUND. BIODIVER	76095	Unknown	Aegilops geniculata Roth.	Roth.	ESP	Spain	Castrillo de la GuareÃ±a		Za	41.1	-5.1	
	IDBD-GN	42456	Observation	Aegilops geniculata Roth	Roth	ES	Spain	Valladana	Aibar	Na	42.5737	-1.35638	4500
00:00.0	REDIAM-CMA	95179	Observation	Aegilops geniculata		ESP	Spain		Cabra	Co	37.492	-4.3728	1000
	BDBCV-General	279915	Observation	Aegilops geniculata		ESPA	Spain	Ayora	El Valle de Ayora	Valenc	38.9659	-0.98017	
	SIVIM	S-P04194:Aegi	Observation	Aegilops geniculata Roth	Roth	ES	Spain	km 3 de la carretera de Balaguer a Agramu			41.71	0.83	280
	SIVIM	S-P09962:Aegi	Observation	Aegilops geniculata Roth	Roth	ES	Spain	rodalies del port de Confrides (Confrides)			38.63	-0.35	0
	SIVIM	S-P14067:Aegi	Observation	Aegilops geniculata Roth	Roth	ES	Spain	Freixo do Meio			39.38	-8.88	144
	SIVIM	T-P01871:Aegi	Observation	Aegilops geniculata Roth	Roth	ES	Spain	Eivissa: el Pla de Vila, prop de ca n'Escand			38.83	1.38	0
	SIVIM	T-P04050:Aegi	Observation	Aegilops geniculata Roth	Roth	ES	Spain	11.5 km al W de S. MartÃ-n de Pusa			39.73	-4.86	0
	SIVIM	T-P06401:Aegi	Observation	Aegilops geniculata Roth	Roth	ES	Spain	BuÃ±ol			39.37	-0.91	0
1887-06-1	US	1018345	Specimen	Aegilops ovata		Spain	Spain	Algerciras		Cadiz			
00:00.0	GDAC	GDAC32510-1	Specimen	Aegilops geniculata Roth.	Roth.	ES	Spain	MÃ¡laga, Antequera, El Torcal.		MA			0
00:00.0	SALA	10061-1	Specimen	Aegilops ovata L. subsp. triaristat	(Willd.) Rouy	ES	Spain	_; Ciudad Rodrigo		Sa			
	GDA	GDA30012-1-1	Specimen	Aegilops ovata L.	L.	ES	Spain	Madrid, Arganda.		M			0
	BC	149182	Specimen	Aegilops ovata L.	L.	ES	Spain	Sant Pere de Vilamajor; St. Pere de		B	41.69	2.34	
00:00.0	BC	634094	Specimen	Aegilops geniculata Roth	Roth	ES	Spain	Dosrius; Dosrius - LlinÃ¡s		B	41.5502	2.400648	
00:00.0	W	41846	Unknown	Aegilops geniculata Roth		ESP	Spain	Prov. Granada: S vom Dilar: von der Eremita de las Nieves gege					900
00:00.0	MGC	27653-1	Unknown	Aegilops geniculata Roth	Roth	ES	Spain	Mollina; Sierra de la Camorra		Ma	0	0	1
	ESP004	NC022338	Specimen	Aegilops geniculata Roth		ESP	Spain	Fuente-Agria, Espiel, province of Cordoba					
00:00.0	MUB	102376-1	Specimen	Aegilops geniculata Roth	Roth	ES	Spain	Moratalla; Sierra de Villafuerte		Mu	38.17	-2.14	1
	FUND. BIODIVER	1336002	Unknown	Aegilops geniculata Roth	Roth	ESP	Spain	UjuÃ©, camino de Chucho Alto		Na	42.1	-1.1	650

	FUND. BIODIVER	1340279	Unknown	Aegilops geniculata Roth	Roth	ESP	Spain	OÃ±a, hacia Pino de Bureba		Bu	42.1	-3.1	600
	FUND. BIODIVER	1366006	Unknown	Aegilops geniculata Roth	Roth	ESP	Spain	Lumbier, La OquÃ-a		Na	42.1	-1.1	600
	FUND. BIODIVER	70861	Unknown	Aegilops ovata L.	L.	ESP	Spain	Arcediano, camino a La VellÃ©s		Sa	40.1	-5.1	
	FUND. BIODIVER	78790	Unknown	Aegilops geniculata Roth.	Roth.	ESP	Spain	Quintanilla de Arriba		Va	41.1	-4.1	
	FUND. BIODIVER	86868	Unknown	Aegilops geniculata		ESP	Spain	Arguijo		So	41.1	-2.1	
00:00.0	REDIAM-CMA	80780	Observation	Aegilops geniculata		ESP	Spain		El Saucejo	Se	37.0967	-5.11389	524
00:00.0	REDIAM-CMA	88245	Observation	Aegilops geniculata		ESP	Spain		Castellar de la Fr	Ca	36.2683	-5.42621	47
	REDIAM-CMA	94646	Observation	Aegilops geniculata		ESP	Spain		Cortes de la Fron	Ma	36.5503	-5.49688	405
00:00.0	REDIAM-CMA	112903	Observation	Aegilops geniculata		ESP	Spain		Carcabuey	Co	37.4415	-4.29195	700
00:00.0	REDIAM-CMA	126300	Observation	Aegilops geniculata		ESP	Spain		Constantina	Se	37.9253	-5.50986	500
00:00.0	BC	866346	Specimen	Aegilops ovata L.	L.	ES	Spain	AlcalÃ¡ de la Selva; La Virgen de la		Te	40.3591	-0.76228	
	SIVIM	S-P04168:Aegi	Observation	Aegilops geniculata Roth	Roth	ES	Spain	Taradell, al turÃ³ de Mont-rodon			41.81	2.15	0
	SIVIM	S-P09958:Aegi	Observation	Aegilops geniculata Roth	Roth	ES	Spain	font de l'Espinal, serra Aitana (Alcoleja)			38.63	-0.35	1150
	SIVIM	S-P14063:Aegi	Observation	Aegilops geniculata Roth	Roth	ES	Spain	Serrinha			38.39	-8.54	0
	SIVIM	T-P01867:Aegi	Observation	Aegilops geniculata Roth	Roth	ES	Spain	Eivissa: Pla de Vila, km 3 de la carretera de			38.83	1.38	0
	SIVIM	T-P03851:Aegi	Observation	Aegilops geniculata Roth	Roth	ES	Spain	7 km al S de Abejar			41.73	-2.87	1120
	SIVIM	T-P06371:Aegi	Observation	Aegilops geniculata Roth	Roth	ES	Spain	Camporrobles			39.55	-1.48	0
	FUND. BIODIVER	1481133	Unknown	Aegilops ovata L.	Req. ex Bert	ESP	Spain	P.N. ArchipiÃ©lago de Cabrera		PM	38.1	2.1	
	FUND. BIODIVER	1554537	Unknown	Aegilops ovata L.	L.	ESP	Spain	Pancorbo		Bu	42.1	-2.1	
	FUND. BIODIVER	1625896	Unknown	Aegilops ovata L.	L.	ESP	Spain	Partido FarmacÃ©utico de Revenga		P	42.1	-4.1	
00:00.0	REDIAM-CMA	189251	Observation	Aegilops geniculata		ESP	Spain		Tabernas	Al	37.0822	-2.34979	487
00:00.0	REDIAM-CMA	232278	Observation	Aegilops geniculata		ESP	Spain		AlmadÃ©n de la F	Se	37.8838	-5.99816	417
	SIVIM	T-P13703:Aegi	Observation	Aegilops geniculata Roth	Roth	ES	Spain	El Letrado, Puertomingalvo			40.26	-0.53	1525
	SIVIM	T-P16910:Aegi	Observation	Aegilops geniculata Roth	Roth	ES	Spain	Ajalvir			40.46	-3.58	0
	SIVIM	T-P19967:Aegi	Observation	Aegilops geniculata Roth	Roth	ES	Spain	El Arco (CaÃ±averal)			39.72	-6.43	0
	SIVIM	T-P24602:Aegi	Observation	Aegilops geniculata Roth	Roth	ES	Spain	Entre Paterna y Monterrey			37.04	-3	1100
	SIVIM	T-P27383:Aegi	Observation	Aegilops geniculata Roth	Roth	ES	Spain	Almoguera			40.29	-3	0
	SIVIM	T-P28638:Aegi	Observation	Aegilops geniculata Roth	Roth	ES	Spain	Mesa Mamalutera, Jaraicejo			39.34	-6.24	0
	IDBD-GN	42423	Observation	Aegilops geniculata Roth	Roth	ES	Spain	Sierra de Leire	Castillonuevo	Na	42.6546	-1.05535	910
	IDBD-GN	42446	Observation	Aegilops geniculata Roth	Roth	ES	Spain	Araia		Vi	42.8852	-2.32165	600
	IDBD-GN	42455	Observation	Aegilops geniculata Roth	Roth	ES	Spain		Sarasate	Na	42.8988	-1.78257	
	ADIMAN	32	Observation	Aegilops geniculata		ESP	Spain	EnguÃ-danos		CU	39.6671	-1.71038	
	BDBCV-General	66066	Observation	Aegilops geniculata		ESPA	Spain	Ares del Maestre	L'Alt Maestrat	Castel	40.3889	-0.11368	
00:00.0	BC	92762	Specimen	Aegilops ovata L.	L.	ES	Spain	Mataporquera; Mataporquera Santa		S	42.85	-4.16	950
00:00.0	SALA	14331-1	Specimen	Aegilops ovata L. subsp. triaristat	(Willd.) Rouy	ES	Spain	_; Ciudad Rodrigo		Sa			
00:00.0	SALA	16032-1	Specimen	Aegilops ovata L. subsp. triaristat	(Willd.) Rouy	ES	Spain	_; La Fregeneda		Sa			
00:00.0	UNEX	34905-1	Observation	Aegilops geniculata Roth	_	ESP	Spain	Badajoz:		Ba	38.8797	-6.8605	
	FUND. BIODIVER	1463974	Unknown	Aegilops geniculata Roth	Roth	ESP	Spain	ValldarnÃ s, entre Cubells i Camara		L	41.1	0.1	340
	FUND. BIODIVER	1475536	Unknown	Aegilops geniculata Roth.	Roth.	ESP	Spain	Sierra del Ricote		Mu	37.1	-1.1	
	FUND. BIODIVER	930422	Unknown	Aegilops ovata L.	L.	ESP	Spain	Siurana de Prades, Capafonts		T	41.1	0.1	
00:00.0	MUB	110231-1	Specimen	Aegilops geniculata Roth	Roth	ES	Spain	Caravaca; Los Prados		Mu	37.974	-1.833	1

	SIVIM	T-P06427:Aegi	Observation	Aegilops geniculata Roth	Roth	ES	Spain	Casas de Madrona			38.92	-1.15	0
	SIVIM	T-P07211:Aegi	Observation	Aegilops geniculata Roth	Roth	ES	Spain	PeÃ±alba			41.42	-0.12	300
	SIVIM	T-P09160:Aegi	Observation	Aegilops geniculata Roth	Roth	ES	Spain	Mota del MarquÃ©s			41.61	-5.28	0
	SIVIM	T-P09356:Aegi	Observation	Aegilops geniculata Roth	Roth	ES	Spain	Puebla de VallÃ©s			40.92	-3.35	1000
	SIVIM	T-P11403:Aegi	Observation	Aegilops geniculata Roth	Roth	ES	Spain	Altos de Cabrejas			40.01	-2.41	1150
	SIVIM	T-P13151:Aegi	Observation	Aegilops geniculata Roth	Roth	ES	Spain	Montevite			42.81	-2.87	0
	FUND. BIODIVER	1648480	Unknown	Aegilops geniculata Roth	Roth	ESP	Spain	Iglesuela del Cid, pista arriba del Ma		Te	40.1	-0.1	
	FUND. BIODIVER	1700455	Unknown	Aegilops geniculata		ESP	Spain	Iglesuela del Cid,Torre de Nicasi		Te	40.1	-0.1	1140
	BC	805573	Specimen	Aegilops geniculata Roth	Roth	ES	Spain	Orihuela del Tremedal; Vora el Gall		Te	40.4635	-1.70236	
00:00.0	REDIAM-CMA	276726	Observation	Aegilops geniculata		ESP	Spain		Santa Eufemia	Co	38.5595	-4.83145	473
00:00.0	REDIAM-CMA	287912	Observation	Aegilops geniculata		ESP	Spain		MontizÃ³n	J	38.3875	-3.01716	793
00:00.0	LEB	4785-1	Specimen	Aegilops geniculata Roth	Roth	ES	Spain	Monte Grande. Valencia de Don Ju		Le	42.2	-5.48	1
	SIVIM	T-P30020:Aegi	Observation	Aegilops geniculata Roth	Roth	ES	Spain	Ejidos de Alborea, Albacete			39.19	-1.49	350
	SIVIM	T-P30054:Aegi	Observation	Aegilops geniculata Roth	Roth	ES	Spain	Pr. Corral Confite, Villamalea, Albacete			39.29	-1.6	0
	SIVIM	T-P31968:Aegi	Observation	Aegilops geniculata Roth	Roth	ES	Spain	Puerto de la Mata (MÃ¡gina)			37.67	-3.56	1600
	SIVIM	U-P02723:Aegi	Observation	Aegilops geniculata Roth	Roth	ES	Spain	XÃ bia, Portitxol			38.72	0.12	0
	SIVIM	U-P06638:Aegi	Observation	Aegilops geniculata Roth	Roth	ES	Spain	Penina			37.22	-8.21	300
	SIVIM	U-P06723:Aegi	Observation	Aegilops geniculata Roth	Roth	ES	Spain	Rocha dos Soidos			37.22	-8.21	0
00:00.0	COFC	41282-1	Specimen	Aegilops geniculata Roth	Roth	ES	Spain	Cabra; Atalayas		Co	37	-4	1
	FUND. BIODIVER	1549353	Unknown	Aegilops ovata		ESP	Spain	Gallec. (comarca del Ferrol)					
	FUND. BIODIVER	1558107	Unknown	Aegilops geniculata		ESP	Spain	Soto de Cerrato		P	41.1	-4.1	840
	FUND. BIODIVER	1564847	Unknown	Aegilops ovata		ESP	Spain	Soto de Cerrato		P	41.1	-4.1	830
	FUND. BIODIVER	1643497	Unknown	Aegilops geniculata Roth	Roth	ESP	Spain	El Castellar		Z	41.1	-0.1	660
	FUND. BIODIVER	979128	Unknown	Aegilops ovata L.	L.	ESP	Spain	Valdealgorfa		Te	40.1	0.9	
00:00.0	ABH	8923-1	Specimen	Aegilops geniculata Roth	Roth	ES	Spain	Petrer; SÂª del Cid		A	38.48	-0.76	
00:00.0	REDIAM-CMA	212626	Observation	Aegilops geniculata		ESP	Spain		EscÃºzar	Gr	37.035	-3.81354	1022
00:00.0	REDIAM-CMA	228677	Observation	Aegilops geniculata		ESP	Spain		Felix	Al	36.905	-2.72243	1399
00:00.0	COFC	52510-1	Specimen	Aegilops geniculata Roth	Roth	ES	Spain	Brovales; _		Ba	38	-6	1
00:00.0	GDA	GDA30010-1-1	Specimen	Aegilops ovata L.	L.	ES	Spain	JaÃ©n, SÂª MÃ¡gina, MojÃ³n Blanc		J			1400
	SIVIM	T-P13229:Aegi	Observation	Aegilops geniculata Roth	Roth	ES	Spain	De Artajona a Larraga			42.53	-1.9	350
	SIVIM	T-P16881:Aegi	Observation	Aegilops geniculata Roth	Roth	ES	Spain	Colmenar Viejo			40.64	-3.82	0
	SIVIM	T-P16920:Aegi	Observation	Aegilops geniculata Roth	Roth	ES	Spain	Ciudad Universitaria			40.37	-3.7	0
	SIVIM	T-P20228:Aegi	Observation	Aegilops geniculata Roth	Roth	ES	Spain	Entre Alcaudete y Martos			37.58	-4.13	0
	SIVIM	T-P26219:Aegi	Observation	Aegilops geniculata Roth	Roth	ES	Spain	Cortijo del Bebedor, Moratalla			38.03	-2.08	0
	SIVIM	T-P27890:Aegi	Observation	Aegilops geniculata Roth	Roth	ES	Spain	Weiderasen am Parador de Arguis, S-Pyre			42.24	-0.45	1050
00:00.0	COA	23221-1	Specimen	Aegylops geniculata Roth	Roth	ES	Spain	Antequera. Sierra Llana		Ma	36.94	-4.8	
00:00.0	I.E.L.	140	Specimen	Aegilops geniculata	Roth	ES	Spain	Arenal de la Virge	Villena	ALICANTE			510
00:00.0	GDAC	GDAC31114-1	Specimen	Aegilops geniculata Roth.	Roth.	ES	Spain	AlmerÃ-a, carretera AlmerÃ-a-Gran		AL			780
	ESP004	NC027426	Specimen	Aegilops geniculata Roth		ESP	Spain	Albaladejito, Cuenca, province of Cuenca			40.0833	-2.2	822
00:00.0	SEV	35317-1	Specimen	Aegilops ovata L.	L.	ES	Spain	Alrededores del aeropuerto de San		Se			1
00:00.0	BDBCV	598488	Observation	Aegilops geniculata		ESP	Spain		Alcoy	Alicant	38.683	-0.534	

	FUND. BIODIVER	1189435	Unknown	Aegilops ovata L.	L.	ESP	Spain			PM			
	FUND. BIODIVER	70867	Unknown	Aegilops ovata L.	L.	ESP	Spain	La Velles		Sa	40.1	-5.1	
	FUND. BIODIVER	856281	Unknown	Aegilops ovata		ESP	Spain	Alicante, hacia Talaia		A	38.1	-0.1	
00:00.0	SALA	66780-1	Specimen	Aegilops geniculata Roth	Roth	ES	Spain	_; BÃ³veda del RÃo Almar		Sa	40.85	-5.22	
00:00.0	SALA	75142-1	Specimen	Aegilops geniculata Roth	Roth	ES	Spain	_; Garrovillas de AlconÃ©tar		Cc	0	0	
00:00.0	FCO	20763-1	Specimen	Aegilops ovata L.	L.	ES	Spain	Madrid; Madrid, Ciudad Universitari		M			
00:00.0	REDIAM-CMA	82279	Observation	Aegilops geniculata		ESP	Spain		Ronda	Ma	36.6878	-5.05694	1300
00:00.0	REDIAM-CMA	95440	Observation	Aegilops geniculata		ESP	Spain		Zurgena	Al	37.3358	-2.03804	302
00:00.0	REDIAM-CMA	117316	Observation	Aegilops geniculata		ESP	Spain		Quesada	J	37.8216	-3.01651	1099
	BDBCV-General	74113	Observation	Aegilops geniculata		ESPA	Spain	Cirat	El Alto Mijares	Castel	40.0372	-0.48014	
00:00.0	COFC	11965-1	Specimen	Aegilops geniculata Roth	Roth	ES	Spain	Puente Genil; rÃo Anzur		Co	37	-4	1
00:00.0	COFC	11991-1	Specimen	Aegilops geniculata Roth	Roth	ES	Spain	Priego de CÃƒÂ³rdoba; entre el mu		Co	37	-4	1
00:00.0	COFC	16298-1	Specimen	Aegilops geniculata Roth	Roth	ES	Spain	Los BlÃƒÂ¡zquez; carretera hacia L		Co	38	-5	1
	SIVIM	S-P09969:Aegi	Observation	Aegilops geniculata Roth	Roth	ES	Spain	penyÃ³ de la Costera (Relleu)			38.54	-0.36	320
	SIVIM	S-P14073:Aegi	Observation	Aegilops geniculata Roth	Roth	ES	Spain	Freixo do Meio			39.38	-8.88	173
	SIVIM	T-P01884:Aegi	Observation	Aegilops geniculata Roth	Roth	ES	Spain	Eivissa: vora el torrent de Boscastell, al co			38.92	1.26	0
	SIVIM	T-P04103:Aegi	Observation	Aegilops geniculata Roth	Roth	ES	Spain	1 km al N de Elche de la Sierra			38.39	-2.08	0
	SIVIM	T-P06405:Aegi	Observation	Aegilops geniculata Roth	Roth	ES	Spain	Requena			39.37	-1.14	0
	FUND. BIODIVER	1188175	Unknown	Aegilops ovata L.	L.	ESP	Spain	Valldemossa		PM	39.1	2.1	
	FUND. BIODIVER	120459	Unknown	Aegilops geniculata Roth	Roth	ESP	Spain	Valdevacas de Montejo, Valdevaca		Sg	41.1	-3.1	
	FUND. BIODIVER	1335996	Unknown	Aegilops geniculata Roth	Roth	ESP	Spain	Aibar, Valladana		Na	42.1	-1.1	450
	FUND. BIODIVER	1351576	Unknown	Aegilops geniculata Roth	Roth	ESP	Spain	Chodes, Cantera de yeso		Z	41.1	-1.1	430
	FUND. BIODIVER	70820	Unknown	Aegilops geniculata Roth	Roth	ESP	Spain	San Ildefonso o la Granja		Sg	40.1	-3.1	
	FUND. BIODIVER	72899	Unknown	Aegilops geniculata Rotch.	Rotch.	ESP	Spain	DoÃ±inos de Salamanca		Sa	40.1	-5.1	
	FUND. BIODIVER	861709	Unknown	Aegilops ovata L.	L.	ESP	Spain	BesalÃº		Ge	42.1	2.1	
00:00.0	SEV	98932-1	Specimen	Aegilops geniculata Roth	Roth	ES	Spain	Algodonales, Sierra de LÃjar		Ca			700
00:00.0	REDIAM-CMA	84382	Observation	Aegilops geniculata		ESP	Spain		Santiago-Pontone	J	38.0664	-2.59998	1409
00:00.0	REDIAM-CMA	99452	Observation	Aegilops geniculata		ESP	Spain		AlmadÃ©n de la F	Se	37.8523	-6.00557	278
00:00.0	REDIAM-CMA	110342	Observation	Aegilops geniculata		ESP	Spain		Conil de la Fronte	Ca	36.3417	-6.01408	50
	SIVIM	S-P13988:Aegi	Observation	Aegilops geniculata Roth	Roth	ES	Spain	Pinhal da Senhora (Freixo do Meio)			39.38	-8.88	141
	SIVIM	T-P03845:Aegi	Observation	Aegilops geniculata Roth	Roth	ES	Spain	3 km al SW de Almiruete			41.01	-3.35	1040
	SIVIM	T-P04217:Aegi	Observation	Aegilops geniculata Roth	Roth	ES	Spain	2 km al N de Jumilla			38.47	-1.39	0
	SIVIM	T-P06421:Aegi	Observation	Aegilops geniculata Roth	Roth	ES	Spain	Siete Aguas			39.46	-1.02	0
	DEU146	AE 971	Specimen	Aegilops geniculata subsp. geniculata Roth		ESP	Spain						
00:00.0	COFC	25601-1	Specimen	Aegilops geniculata Roth	Roth	ES	Spain	casco urbano; Sotos de la Albolafia		Co			1
	FUND. BIODIVER	1463980	Unknown	Aegilops geniculata Roth	Roth	ESP	Spain	GÃ rzola		L	41.1	0.1	540
	FUND. BIODIVER	976282	Unknown	Aegilops ovata L.	L.	ESP	Spain	Torrecilla de AlcaÃ±iz		Te	40.1	0.9	
	SIVIM	T-P06443:Aegi	Observation	Aegilops geniculata Roth	Roth	ES	Spain	La Yesa			39.82	-1.01	0
	SIVIM	T-P07217:Aegi	Observation	Aegilops geniculata Roth	Roth	ES	Spain	Litera			42.03	-0.62	0
	SIVIM	T-P09197:Aegi	Observation	Aegilops geniculata Roth	Roth	ES	Spain	Simancas			40.63	-4.89	0
	SIVIM	T-P10141:Aegi	Observation	Aegilops geniculata Roth	Roth	ES	Spain	La Peguera, AlcalÃ¡ de los Gazules			36.38	-5.78	120

	SIVIM	T-P11410:Aegi	Observation	Aegilops geniculata Roth	Roth	ES	Spain	Sierra de San Felipe			40.37	-1.93	1370
	SIVIM	T-P13171:Aegi	Observation	Aegilops geniculata Roth	Roth	ES	Spain	Agorreta, pto. Erro			42.89	-1.53	0
00:00.0	GDA	GDA10273-1-3	Specimen	Aegilops geniculata Roth.	Roth.	ES	Spain	Granada, Ãzbor, camino de LanjarÃ		GR			600
00:00.0	COFC	36344-1	Specimen	Aegilops geniculata Roth	Roth	ES	Spain	Santaella; Km-19-20 de la ctra. hac		Co			1
	IDBD-GN	42417	Observation	Aegilops geniculata Roth	Roth	ES	Spain	Sierra de Alaiz	Monreal	Na	42.6655	-1.47598	1000
	IDBD-GN	42438	Observation	Aegilops geniculata Roth	Roth	ES	Spain		Pamplona	Na	42.8471	-1.5939	
	IDBD-GN	42450	Observation	Aegilops geniculata Roth	Roth	ES	Spain		Belascoain	Na	42.7598	-1.8403	
00:00.0	COFC	41153-1	Specimen	Aegilops geniculata Roth	Roth	ES	Spain	Baena; rÃƒÂ-o Guadajoz; carretera		Co	37	-4	1
00:00.0	COFC	41291-1	Specimen	Aegilops geniculata Roth	Roth	ES	Spain	Cabra; los Cerrajones		Co	37	-4	1
	MGC	358-1	Unknown	Aegilops ovata L.	L.	ES	Spain	_		Va	0	0	1
00:00.0	SEV	20869-1	Specimen	Aegilops ovata L.	L.	ES	Spain	La Mola, O-Seite, Wegrand nahe de		Fo			120
00:00.0	MUB	109836-1	Specimen	Aegilops geniculata Roth	Roth	ES	Spain	Aledo; Estrecho de la Arboleja		Mu	37.782	-1.598	1
00:00.0	FUND. BIODIVER	1535538	Unknown	Aegilops ovata		ESP	Spain	parte occidental de Mataporquera		S	42.1	-3.1	
	FUND. BIODIVER	1593210	Unknown	Aegilops geniculata Roth.	Roth.	ESP	Spain	MuÃ±eca		P	42.1	-4.1	
	BDBCV-General	66311	Observation	Aegilops geniculata		ESPA	Spain	Ares del Maestre	L'Alt Maestrat	Castel	40.3889	-0.11368	
	BDBCV-General	276487	Observation	Aegilops geniculata		ESPA	Spain	Bolbaite	La Canal de Nava	Valenc	39.0517	-0.74664	
	BDBCV-General	279300	Observation	Aegilops geniculata		ESPA	Spain	Teresa de Cofren	El Valle de Ayora	Valenc	39.146	-0.97503	
00:00.0	REDIAM-CMA	189696	Observation	Aegilops geniculata		ESP	Spain		Tabernas	Al	37.0932	-2.33949	509
00:00.0	REDIAM-CMA	199595	Observation	Aegilops geniculata		ESP	Spain		Espiel	Co	38.1521	-5.11281	618
00:00.0	REDIAM-CMA	223243	Observation	Aegilops geniculata		ESP	Spain		SerÃ³n	Al	37.3821	-2.57656	993
00:00.0	REDIAM-CMA	236586	Observation	Aegilops geniculata		ESP	Spain		Fuente Obejuna	Co	38.2292	-5.35616	582
	CZE122	01C2109092	Specimen	Aegilops geniculata Roth		ESP	Spain	Pyrenaeen					
	SIVIM	T-P13733:Aegi	Observation	Aegilops geniculata Roth	Roth	ES	Spain	MasÃ-a de la Rambla, Vilafranca			40.43	-0.28	1140
	SIVIM	T-P16914:Aegi	Observation	Aegilops geniculata Roth	Roth	ES	Spain	Venta de Cabrejas			40.01	-2.41	0
	SIVIM	T-P19971:Aegi	Observation	Aegilops geniculata Roth	Roth	ES	Spain	Cerro de Aldeamoret (CÃ¡ceres)			39.36	-6.44	0
	SIVIM	T-P25256:Aegi	Observation	Aegilops geniculata Roth	Roth	ES	Spain	Camp abandonat prop de Macarella (Meno			39.92	3.93	0
	SIVIM	T-P27623:Aegi	Observation	Aegilops geniculata Roth	Roth	ES	Spain	Prados de la Nava, Sierra de Cabra			37.48	-4.47	1010
	SIVIM	T-P28644:Aegi	Observation	Aegilops geniculata Roth	Roth	ES	Spain	Cerros de la Plaza, Serradilla			39.8	-6.19	230
00:00.0	COA	41158-1	Specimen	Aegilops geniculata Roth	Roth	ES	Spain	A 5 Km de Villafranca desde el pant		Co	37.94	-4.71	
	CZE122	01C2109096	Specimen	Aegilops geniculata Roth		ESP	Spain	W.-Pyrenaeen b. Jaca, 810 m					
	CZE122	01C2109061	Specimen	Aegilops geniculata subsp. gibberosa (ZHUK.)		ESP	Spain	Barcelona					
00:00.0	SALA	60047-1	Specimen	Aegilops ovata L.	L.	ES	Spain	_; PeÃ±ausende		Za			
00:00.0	SEV	30537-1	Specimen	Aegilops ovata L.	L.	ES	Spain	AlgÃ¡mitas, Sierra del TablÃ³n		Se			1
00:00.0	SEV	5985-1	Specimen	Aegilops ovata L.	L.	ES	Spain	Playa de MazagÃ³n		Mu			1
00:00.0	SEV	57523-1	Specimen	Aegilops geniculata Roth	Roth	ES	Spain	Entre Coripe y Montellano		Se			1
	FUND. BIODIVER	1696521	Unknown	Aegilops geniculata Roth	Roth	ESP	Spain	Cantavieja, mas de Porcar		Te	40.1	-0.1	1500
	BDBCV-General	68482	Observation	Aegilops geniculata		ESPA	Spain				40.3045	0.352572	
	BDBCV-General	81774	Observation	Aegilops geniculata		ESPA	Spain				39.8596	-0.60353	
00:00.0	COFC	11969-1	Specimen	Aegilops geniculata Roth	Roth	ES	Spain	Rute; rÃ-o Anzur		Co	37	-4	1
00:00.0	COFC	11951-1	Specimen	Aegilops geniculata Roth	Roth	ES	Spain	Priego de CÃ³rdoba; entre el munici		Co	37	-4	1
00:00.0	COFC	11987-1	Specimen	Aegilops geniculata Roth	Roth	ES	Spain	Rute; Sierra de Rute; cerro del Cast		Co	37	-4	1

00:00.0	REDIAM-CMA	243266	Observation	Aegilops geniculata		ESP	Spain		Padul	Gr	37.0054	-3.69502	1023
00:00.0	REDIAM-CMA	265482	Observation	Aegilops geniculata		ESP	Spain		Lucainena de las	Al	37.0207	-2.09744	368
00:00.0	REDIAM-CMA	280192	Observation	Aegilops geniculata		ESP	Spain		Adamuz	Co	38.0204	-4.52603	211
00:00.0	REDIAM-CMA	282971	Observation	Aegilops geniculata		ESP	Spain		Vilches	J	38.1303	-3.4751	395
00:00.0	MGC	66063-1	Unknown	Aegilops geniculata Roth	Roth	ES	Spain	Periana; Entre los Cortijos Chamizo		Ma	36.955	-4.219	700
	SIVIM	T-P30046:Aegi	Observation	Aegilops geniculata Roth	Roth	ES	Spain	Pr. Casa de AntÃ³n, Jalance, Valencia			39.19	-1.14	0
	SIVIM	T-P30072:Aegi	Observation	Aegilops geniculata Roth	Roth	ES	Spain	Carretera a San AntÃ³n, Villamalea, Albace			39.29	-1.6	0
	SIVIM	U-P02497:Aegi	Observation	Aegilops geniculata Roth	Roth	ES	Spain	Pego Vall d'Ebo			38.81	-0.12	0
	SIVIM	U-P02916:Aegi	Observation	Aegilops geniculata Roth	Roth	ES	Spain	La Xara XÃ bia			38.72	0	0
	SIVIM	U-P06710:Aegi	Observation	Aegilops geniculata Roth	Roth	ES	Spain	Zimbral de Baixo			37.13	-8.21	220
	SIVIM	U-P08061:Aegi	Observation	Aegilops geniculata Roth	Roth	ES	Spain	La Nava, Berzocana			39.36	-5.55	0
	COA	41223-1	Specimen	Aegilops ovata L.	L.	ES	Spain	Arganda		M	40.29	-3.47	
	MA	573230-1	Specimen	Aegilops ovata L.	L.	ES	Spain			M			
	RUS001	VIR100602112	Specimen	Aegilops ovata L.		ESP	Spain						
	RUS001	VIR100602091	Specimen	Aegilops ovata L.		ESP	Spain						
	FUND. BIODIVER	1093252	Unknown	Aegilops geniculata		ESP	Spain	Almaraz		Cc	39.1	-5.1	
	FUND. BIODIVER	1925487	Unknown	Aegilops geniculata Roth	Roth	ESP	Spain	Yesa		Na	42.1	-0.1	540
	FUND. BIODIVER	1930605	Unknown	Aegilops geniculata		ESP	Spain	Macarella, calvero de un ullastrar		PM	39.1	3.1	
	FUND. BIODIVER	1946394	Unknown	Aegilops geniculata Roth	Roth	ESP	Spain	Provincia de Ciudad Real		CR			
00:00.0	BC	70798	Specimen	Aegilops geniculata Roth	Roth	ES	Spain	Albanchez de Ubeda; Loma Vaquer		J	37.72	-3.4	1400
00:00.0	REDIAM-CMA	9316	Observation	Aegilops geniculata		ESP	Spain		Coripe	Se	36.9818	-5.3787	300
00:00.0	REDIAM-CMA	16857	Observation	Aegilops geniculata		ESP	Spain		Almonaster la Re	H	37.8611	-6.85602	503
00:00.0	REDIAM-CMA	23877	Observation	Aegilops geniculata		ESP	Spain		Cambil	J	37.6922	-3.51134	1052
00:00.0	REDIAM-CMA	396408	Observation	Aegilops geniculata		ESP	Spain		Gerena	Se	37.5246	-6.19006	74
00:00.0	REDIAM-CMA	400669	Observation	Aegilops geniculata		ESP	Spain		Santiago-Pontone	J	38.1232	-2.54803	1544
00:00.0	REDIAM-CMA	415526	Observation	Aegilops geniculata		ESP	Spain		Cazorla	J	37.8457	-2.89258	1251
00:00.0	REDIAM-CMA	418744	Observation	Aegilops geniculata		ESP	Spain		Chimeneas	Gr	37.1496	-3.91098	701
00:00.0	MGC	11345-1	Unknown	Aegilops geniculata Roth	Roth	ES	Spain	Algodonales; Sierra de LÃ-jar		Ca	0	0	500
	SIVIM	Q-P03600:Aegi	Observation	Aegilops geniculata Roth	Roth	ES	Spain	Ludiente			40.08	-0.41	0
	SIVIM	Q-P06536:Aegi	Observation	Aegilops geniculata Roth	Roth	ES	Spain	Els Estepars, entre Prades i Albarca			41.26	0.85	0
	SIVIM	Q-P08643:Aegi	Observation	Aegilops geniculata Roth	Roth	ES	Spain	Ruesga ,			42.89	-4.59	1030
	SIVIM	R-P03707:Aegi	Observation	Aegilops geniculata Roth	Roth	ES	Spain	Carretera de Tudela a Ejea de los Caballer			41.99	-1.67	0
	SIVIM	R-P08196:Aegi	Observation	Aegilops geniculata Roth	Roth	ES	Spain	Torrent de GÃ¼ells, vora CastellolÃ-			41.54	1.68	0
	SIVIM	R-P09399:Aegi	Observation	Aegilops geniculata Roth	Roth	ES	Spain	Alcolea del Pinar			41.01	-2.52	1120
00:00.0	MUB	107914-1	Specimen	Aegilops geniculata Roth	Roth	ES	Spain	Ricote; Embalse del MayÃ©s		Mu	38.112	-1.375	1
	FUND. BIODIVER	1463971	Unknown	Aegilops geniculata Roth	Roth	ESP	Spain	Ã€ger, riu Fred		L	41.1	0.1	650
	FUND. BIODIVER	96902	Unknown	Aegilops geniculata Roth.	Roth.	ESP	Spain	Pereruela		Za	41.1	-5.1	
	SEV	11060-1	Specimen	Aegilops ovata L.	L.	ES	Spain	Inmediaciones de Sevilla		Se			1
00:00.0	REDIAM-CMA	158733	Observation	Aegilops geniculata		ESP	Spain		Valverde del Cam	H	37.6003	-6.80383	172
	SIVIM	T-P06966:Aegi	Observation	Aegilops geniculata Roth	Roth	ES	Spain	MatadeÃ³n			42.24	-5.42	0
	SIVIM	T-P09155:Aegi	Observation	Aegilops geniculata Roth	Roth	ES	Spain	Portillo			41.44	-4.67	0

	SIVIM	T-P09353:Aegi	Observation	Aegilops geniculata Roth	Roth	ES	Spain	PontÃ³n de la Oliva			40.83	-3.47	0
	SIVIM	T-P10779:Aegi	Observation	Aegilops geniculata Roth	Roth	ES	Spain	Moreruela de TÃ¡bara			41.78	-5.88	0
	SIVIM	T-P13217:Aegi	Observation	Aegilops geniculata Roth	Roth	ES	Spain	Gallipienzo, hacia Murillo del Fruto			42.44	-1.54	0
00:00.0	MA	633044-1	Specimen	Aegilops geniculata		ES	Spain	Loeches		M			
00:00.0	JBS	464-1	Specimen	Aegilops ovata L.	L.	ES	Spain	SÃ³ller; Olivar d'Es Fenas		Mll			1
	SANT	38068	Specimen	Aegilops geniculata Roth		ES	Spain	El Frago		Z			
	FUND. BIODIVER	1696525	Unknown	Aegilops geniculata Roth	Roth	ESP	Spain	Iglesuela del Cid, la Pobla del Belle		Te	40.1	-0.1	1500
	FUND. BIODIVER	1699513	Unknown	Aegilops geniculata		ESP	Spain	Mosqueruela, La Barraca		Te	40.1	-0.1	1100
	FUND. BIODIVER	1700451	Unknown	Aegilops geniculata		ESP	Spain	Cantavieja		Te	40.1	-0.1	1400
00:00.0	REDIAM-CMA	243860	Observation	Aegilops geniculata		ESP	Spain		AgrÃ³n	Gr	37.0191	-3.86256	1105
00:00.0	COFC	41148-1	Specimen	Aegilops geniculata Roth	Roth	ES	Spain	Puente Genil; laguna de TÃƒÂ-scar		Co	37	-4	1
	SIVIM	T-P30050:Aegi	Observation	Aegilops geniculata Roth	Roth	ES	Spain	Pr. Corral de la Rada, Villamalea, Albacete			39.29	-1.6	0
	SIVIM	T-P30140:Aegi	Observation	Aegilops geniculata Roth	Roth	ES	Spain	Casa de La Desesperada, Casas de Ves, A			39.28	-1.37	0
	SIVIM	U-P02613:Aegi	Observation	Aegilops geniculata Roth	Roth	ES	Spain	Castells de Serrella			38.63	-0.24	0
	SIVIM	U-P06719:Aegi	Observation	Aegilops geniculata Roth	Roth	ES	Spain	Vale LoulÃ©			37.13	-8.21	100
	SIVIM	U-P08133:Aegi	Observation	Aegilops geniculata Roth	Roth	ES	Spain	Los Lotes, CaÃ±amero			39.27	-5.43	0
	ESP004	NC027362	Specimen	Aegilops geniculata Roth		ESP	Spain	El Algar, Cartagena, province of Murcia			37.65	-0.86667	40
	BC	92732	Specimen	Aegilops ovata L.	L.	ES	Spain	Vallfogona de Riucorb; Vallfogona d		T	41.58	1.26	
00:00.0	BDBCV	597600	Observation	Aegilops geniculata		ESP	Spain	Parc Natural de la	Alcoy	Alicant	38.673	-0.488	
	FUND. BIODIVER	118440	Unknown	Aegilops geniculata Roth.	Roth.	ESP	Spain	La Pola de GordÃ³n, Santa LucÃ-a		Le	42.1	-5.1	
	FUND. BIODIVER	1189439	Unknown	Aegilops ovata L.	L.	ESP	Spain	Rafal Fort		PM	39.1	3.1	
	FUND. BIODIVER	70871	Unknown	Aegilops ovata subsp. triaristata	L.	ESP	Spain	Pajares de la Laguna, Laguna Gem		Sa	40.1	-5.1	
	FUND. BIODIVER	85990	Unknown	Aegilops geniculata		ESP	Spain	Herrera de Soria		So	41.1	-2.1	
	FUND. BIODIVER	902846	Unknown	Aegilops ovata L.	L.	ESP	Spain	BorredÃ¡		B	41.1	1.1	
00:00.0	REDIAM-CMA	83203	Observation	Aegilops geniculata		ESP	Spain		Arroyo del Ojanco	J	38.3019	-2.85053	949
00:00.0	REDIAM-CMA	91512	Observation	Aegilops geniculata		ESP	Spain		Barbate	Ca	36.2348	-5.9117	44
00:00.0	REDIAM-CMA	121094	Observation	Aegilops ovata		ESP	Spain		Cazorla	J	37.9223	-2.82109	1667
	SIVIM	S-P14152:Aegi	Observation	Aegilops geniculata Roth	Roth	ES	Spain	Serrinha			38.39	-8.54	350
	SIVIM	T-P03840:Aegi	Observation	Aegilops geniculata Roth	Roth	ES	Spain	1 km al S de TamajÃ³n			40.92	-3.35	1000
	SIVIM	T-P04197:Aegi	Observation	Aegilops geniculata Roth	Roth	ES	Spain	6 km al N de Yecla			38.65	-1.16	0
	SIVIM	T-P04198:Aegi	Observation	Aegilops geniculata Roth	Roth	ES	Spain	5 km al NW de Yecla			38.56	-1.16	0
	SIVIM	T-P06416:Aegi	Observation	Aegilops geniculata Roth	Roth	ES	Spain	Ayora			39.01	-1.03	0
00:00.0	GDA	GDA48984-1-2	Specimen	Aegilops geniculata Roth	Roth	ES	Spain	AlmerÃ-a, SÂª de los Filabres, Beni		AL			800
00:00.0	GDA	GDA54069-1-1	Specimen	Aegilops geniculata Roth.	Roth.	ES	Spain	Granada, SÂª Parapanda, cerca Ctj		GR			1000
00:00.0	ABH	32259-1	Specimen	Aegilops geniculata Roth	Roth	ES	Spain	Alicante; Colonia de la Albufereta		A	38.36	-0.45	
00:00.0	ABH	36927-1	Specimen	Aegilops geniculata Roth	Roth	ES	Spain	Orihuela; Cabo PeÃ±as		A	37.93	-0.72	
00:00.0	BDBCV	98	Observation	Aegilops geniculata		ESP	Spain	Parque Natural de Penyagolosa		Cs	40.19	-0.4	
	IDBD-GN	42435	Observation	Aegilops geniculata Roth	Roth	ES	Spain		Arre	Na	42.8162	-1.64964	
00:00.0	GDAC	GDAC21284-1	Specimen	Aegilops geniculata Roth.	Roth.	ES	Spain	Madrid, Embalse de Santillana, en l		M			0
00:00.0	SALA	47672-1	Specimen	Aegilops geniculata Roth	Roth	ES	Spain	_; Gujiuelo		Sa			
1872-08-0	BC	70796	Specimen	Aegilops ovata L.	L.	ES	Spain	Barcelona; Reginoes sub-montanae		B	41.41	2.1	

	STU	Main-1-1966	Specimen	Aegilops geniculata Roth		ES	Spain	Bei Carromina S Cardona					
	RUS001	VIR100602109	Specimen	Aegilops ovata L.		ESP	Spain						
	RUS001	VIR100602088	Specimen	Aegilops ovata L.		ESP	Spain						
00:00.0	BDBCV	596241	Observation	Aegilops geniculata		ESP	Spain		Alcoy	Alicant	38.666	-0.546	
	FUND. BIODIVER	1084632	Unknown	Aegilops ovata L.	L.	ESP	Spain	Tielmes		M	40.1	-3.1	
	FUND. BIODIVER	1086010	Unknown	Aegilops geniculata Roth.	Roth.	ESP	Spain	Arganda del Rey		M	40.1	-3.1	
	FUND. BIODIVER	1093233	Unknown	Aegilops geniculata		ESP	Spain	Finca de Valdelasyeguas, Aliseda		Cc	39.1	-6.1	
	FUND. BIODIVER	1093255	Unknown	Aegilops geniculata		ESP	Spain	Cuesta de Araya, Garrovillas		Cc	39.1	-6.1	
	FUND. BIODIVER	1925490	Unknown	Aegilops geniculata Roth	Roth	ESP	Spain	Imizcoz		Na	42.1	-1.1	820
	FUND. BIODIVER	1931466	Unknown	Aegilops geniculata		ESP	Spain	Cerca de Macarella		PM	39.1	3.1	
00:00.0	FUND. BIODIVER	1946397	Unknown	Aegilops geniculata Roth	Roth	ESP	Spain	Torralba de Calatrava, Campomojad		CR	38.1	-3.1	610
00:00.0	REDIAM-CMA	229	Observation	Aegilops geniculata		ESP	Spain		Jerez de la Fronte	Ca	36.5956	-6.02769	65
00:00.0	REDIAM-CMA	9965	Observation	Aegilops geniculata		ESP	Spain		Cambil	J	37.6843	-3.53434	899
00:00.0	REDIAM-CMA	17689	Observation	Aegilops geniculata		ESP	Spain		Almonaster la Re	H	37.8581	-6.85792	500
	REDIAM-CMA	396587	Observation	Aegilops geniculata		ESP	Spain		Cortes de la Fron	Ma	36.5504	-5.49637	405
00:00.0	REDIAM-CMA	401602	Observation	Aegilops geniculata		ESP	Spain		Santiago-Pontone	J	38.0839	-2.55711	1222
00:00.0	REDIAM-CMA	408240	Observation	Aegilops geniculata		ESP	Spain		Frailes	J	37.5063	-3.82599	1210
00:00.0	REDIAM-CMA	412045	Observation	Aegilops geniculata		ESP	Spain		La Granada de R	H	37.7568	-6.48862	507
00:00.0	REDIAM-CMA	415579	Observation	Aegilops geniculata		ESP	Spain		Pozo AlcÃ³n	J	37.7709	-2.90465	950
00:00.0	HUAL	1134-1	Specimen	Aegilops geniculata Roth	Roth	ES	Spain	SÂª del Pozo, rÃ-o Turrillas		J	37.727	-2.972	
	SIVIM	Q-P03036:Aegi	Observation	Aegilops geniculata Roth	Roth	ES	Spain	La Llacuna, campm abandonat al costat or			41.45	1.44	0
	SIVIM	Q-P03603:Aegi	Observation	Aegilops geniculata Roth	Roth	ES	Spain	San Vicente			40.08	-0.53	0
	SIVIM	Q-P06539:Aegi	Observation	Aegilops geniculata Roth	Roth	ES	Spain	Els Estepars, entre Prades i Albarca			41.26	0.85	0
	SIVIM	R-P00050:Aegi	Observation	Aegilops geniculata Roth	Roth	ES	Spain	barranc del riu Boix (Montsec de RÃºbies)			41.98	0.94	0
	SIVIM	R-P04067:Aegi	Observation	Aegilops geniculata Roth	Roth	ES	Spain	Taradell, Torrellebreta, prop de la carretera			41.81	2.15	0
	SIVIM	R-P08206:Aegi	Observation	Aegilops geniculata Roth	Roth	ES	Spain	Sota el coll dels Brucs, vers CastellolÃ-			41.54	1.68	0
	SIVIM	S-P01349:Aegi	Observation	Aegilops ovata L.	L.	ES	Spain	Ports de Tortosa: Les Bassetes			40.61	0.16	0
00:00.0	LEB	11623-1	Specimen	Aegilops geniculata Roth	Roth	ES	Spain	La Vid		Le	42.92	-5.63	1
00:00.0	MGC	63379-1	Unknown	Aegilops geniculata Roth	Roth	ES	Spain	Ronda; Mures		Ma	36.76	-5.224	820
00:00.0	COFC	46891-1	Specimen	Aegilops geniculata Roth	Roth	ES	Spain	entrada al camino de servicio del ca		Co			1
	FUND. BIODIVER	1025028	Unknown	Aegilops geniculata		ESP	Spain	E. Martos y Alcaudete		J	37.1	-3.1	
	FUND. BIODIVER	1811872	Unknown	Aegilops geniculata Roth	Roth	ESP	Spain	Bardena Blanca		Na	42.1	-1.1	
00:00.0	BC	601429	Specimen	Aegilops ovata L.	L.	ES	Spain	Prades; Muntanyes de Prades: Plan		T	41.31	1.03	1100
00:00.0	REDIAM-CMA	299500	Observation	Aegilops geniculata		ESP	Spain		Cazorla	J	37.8331	-2.88303	1378
00:00.0	REDIAM-CMA	328237	Observation	Aegilops geniculata		ESP	Spain		Casares	Ma	36.4402	-5.22379	200
00:00.0	REDIAM-CMA	382963	Observation	Aegilops geniculata		ESP	Spain				37.7715	-3.28777	
00:00.0	PreservedSpecime	E00086655	Unknown	Aegilops geniculata Roth		ES	Spain						
	SIVIM	U-P09941:Aegi	Observation	Aegilops geniculata Roth	Roth	ES	Spain	Pastizal cerca de Casas Blancas			38.84	-3	0
	ESP004	NC050476	Specimen	Aegilops geniculata Roth		ESP	Spain	Valdemorillo Sierra, province of Cuenca			40.0333	-1.76667	1211
	FUND. BIODIVER	36854	Unknown	Aegilops geniculata Roth	Roth	ESP	Spain	BerrÃ-cano		Vi	42.1	-2.1	600
00:00.0	REDIAM-CMA	51569	Observation	Aegilops geniculata		ESP	Spain		Zufre	H	37.7679	-6.46041	477

00:00.0	REDIAM-CMA	71644	Observation	Aegilops geniculata		ESP	Spain		Torres	J	37.7509	-3.50086	1311
	SIVIM	R-P09691:Aegi	Observation	Aegilops geniculata Roth	Roth	ES	Spain	Atienza, baldÃ-o			41.19	-2.88	1140
	SIVIM	R-P10553:Aegi	Observation	Aegilops geniculata Roth	Roth	ES	Spain	La Calderona , AG			36.47	-5.79	0
	SIVIM	R-P11662:Aegi	Observation	Aegilops geniculata Roth	Roth	ES	Spain	Tarifa			44.93	-6.04	0
	SIVIM	S-P01256:Aegi	Observation	Aegilops geniculata Roth	Roth	ES	Spain	Vinaixa, la Solana			41.44	0.84	0
	SIVIM	S-P03972:Aegi	Observation	Aegilops geniculata Roth	Roth	ES	Spain	Fulleda, afores del poble			41.44	0.96	0
00:00.0	BDBCV	110	Observation	Aegilops geniculata		ESP	Spain	Parque Natural de Penyagolosa		Cs			
	SANT	23214	Specimen	Aegilops geniculata Roth		ES	Spain	Monforte,As Barrioncas		Lu			
00:00.0	BC	124057	Specimen	Aegilops geniculata Roth	Roth	ES	Spain	San Fernando; Baetica: S. Fernand		Ca	36.52	-6.15	
00:00.0	BC	125251	Specimen	Aegilops ovata L.	L.	ES	Spain	Palma de Mallorca; Puig de Picamo		PM	39.16	2.94	
00:00.0	SALA	10062-1	Specimen	Aegilops ovata L. subsp. triaristat	(Willd.) Rouy	ES	Spain	_; Castillejo de MartÃ-n Viejo, Parad		Sa			
00:00.0	HSS	8884	Specimen	Aegilops geniculata Roth	Roth	ES	Spain	Santo Domingo (proximidades)		Ba	38.6982	-7.10294	
00:00.0	ABH	33032-1	Specimen	Aegilops geniculata Roth	Roth	ES	Spain	MonÃ³var; Sierra del Reclot, Almorc		A	38.39	-0.96	
00:00.0	MGC	27654-1	Unknown	Aegilops geniculata Roth	Roth	ES	Spain	Marbella; Sierra Real. RÃ-o Guadai		Ma	0	0	1
	IDBD-GN	42418	Observation	Aegilops geniculata Roth	Roth	ES	Spain	El PerdÃ³n	Galar	Na	42.7585	-1.71811	
	IDBD-GN	42439	Observation	Aegilops geniculata Roth	Roth	ES	Spain	riberas del Arga	Olave	Na	42.8471	-1.5939	
	IDBD-GN	42448	Observation	Aegilops geniculata Roth	Roth	ES	Spain	Corres		Vi	42.6967	-2.42139	700
00:00.0	SEV	30543-1	Specimen	Aegilops ovata L.	L.	ES	Spain	Entre MorÃ³n y Pruna. Dehesa Rein		Se			1
00:00.0	SEV	53816-1	Specimen	Aegilops ovata L.	L.	ES	Spain	Playa de MazarrÃ³n		Mu			1
00:00.0	SEV	5990-1	Specimen	Aegilops ovata L.	L.	ES	Spain	Aldeaquemada		J			1
00:00.0	REDIAM-CMA	189544	Observation	Aegilops geniculata		ESP	Spain		Tabernas	Al	37.0935	-2.33965	509
00:00.0	REDIAM-CMA	208510	Observation	Aegilops geniculata		ESP	Spain		Constantina	Se	37.9391	-5.55344	669
00:00.0	REDIAM-CMA	223161	Observation	Aegilops geniculata		ESP	Spain		SerÃ³n	Al	37.3652	-2.54428	877
00:00.0	REDIAM-CMA	232387	Observation	Aegilops geniculata		ESP	Spain		AlmadÃ©n de la F	Se	37.8833	-5.99781	417
	BDBCV-General	79717	Observation	Aegilops geniculata		ESPA	Spain	Burriana	La Plana Baixa	Castel	39.8462	-0.03904	
00:00.0	COFC	11974-1	Specimen	Aegilops geniculata Roth	Roth	ES	Spain	Lucena; cerro la Galeota		Co	37	-4	1
	SIVIM	T-P13732:Aegi	Observation	Aegilops geniculata Roth	Roth	ES	Spain	Pobla del Bellestar, Iglesuela del Cid			40.44	-0.4	1120
	SIVIM	T-P16913:Aegi	Observation	Aegilops geniculata Roth	Roth	ES	Spain	Boniches			39.92	-1.71	0
	SIVIM	T-P19970:Aegi	Observation	Aegilops geniculata Roth	Roth	ES	Spain	Cerro de Aldeamoret (CÃ¡ceres)			39.36	-6.44	0
	SIVIM	T-P25252:Aegi	Observation	Aegilops geniculata Roth	Roth	ES	Spain	Macarella, calvero de un 'ullastrar' (Menorc			39.92	3.93	0
	SIVIM	T-P27424:Aegi	Observation	Aegilops geniculata Roth	Roth	ES	Spain	OntÃ-gola			39.92	-3.58	0
	SIVIM	T-P28643:Aegi	Observation	Aegilops geniculata Roth	Roth	ES	Spain	Collado de Murcia, TorrejÃ³n el Rubio			44.1	-14.74	0
	FUND. BIODIVER	1036574	Unknown	Aegilops ovata		ESP	Spain	MÃ¡laga		Ma	36.1	-4.1	
	FUND. BIODIVER	1043760	Unknown	Aegilops geniculata Roth	Roth	ESP	Spain	OjÃ©n, Sierra Blanca		Ma	36.1	-4.1	
	FUND. BIODIVER	1072714	Unknown	Aegilops geniculata L.	L.	ESP	Spain	Antigua Finca Las Rocillas, a 400 m		Ba	38.1	-6.1	
00:00.0	FUND. BIODIVER	1835090	Unknown	Aegilops geniculata Roth	Roth	ESP	Spain	Alhambra, sierra de Alhambra, arroy		CR	38.1	-2.1	
	FUND. BIODIVER	1891036	Unknown	Aegilops geniculata Roth	Roth	ESP	Spain	Formentera		PM	38.1	1.1	
00:00.0	REDIAM-CMA	383932	Observation	Aegilops geniculata		ESP	Spain		AlgatocÃ-n	Ma	36.565	-5.29219	809
00:00.0	REDIAM-CMA	393974	Observation	Aegilops geniculata		ESP	Spain		VÃ©lez-Blanco	Al	37.7482	-2.10958	979
	IPK	AE 421	Living	Aegilops geniculata Roth subsp. gibberosa (Zhuk.) K			Spain	Barcelona			41.3831	2.183056	
	SIVIM	P-P08462:Aegi	Observation	Aegilops geniculata Roth	Roth	ES	Spain	Fredes			40.7	0.15	1100

	SIVIM	P-P08874:Aegi	Observation	Aegilops geniculata Roth	Roth	ES	Spain	Moletes de Venanci; Alfara			40.79	0.27	1140
	SIVIM	P-P08927:Aegi	Observation	Aegilops geniculata Roth	Roth	ES	Spain	Les Foies; Alfara			40.79	0.27	1180
	SIVIM	P-P10766:Aegi	Observation	Aegilops geniculata Roth	Roth	ES	Spain	Els Carrascalets			40.61	0.28	0
	SIVIM	P-P12753:Aegi	Observation	Aegilops geniculata Roth	Roth	ES	Spain	Los Cerricos			37.49	-2.2	1110
	RUS001	VIR100602113	Specimen	Aegilops ovata L.		ESP	Spain						
	RUS001	VIR100602092	Specimen	Aegilops ovata L.		ESP	Spain						
	FUND. BIODIVER	1077309	Unknown	Aegilops ovata L.	L.	ESP	Spain	Toledo, tÃ©rmino municipal		To	39.1	-3.1	
	FUND. BIODIVER	1925486	Unknown	Aegilops geniculata Roth	Roth	ESP	Spain	Lumbier, Foz de Lumbier		Na	42.1	-1.1	420
00:00.0	FUND. BIODIVER	1946393	Unknown	Aegilops geniculata Roth	Roth	ESP	Spain	Piedrabuena, volcan de Piedrabuen		CR	38.1	-3.1	600
00:00.0	REDIAM-CMA	8848	Observation	Aegilops geniculata		ESP	Spain		VÃ©lez-Blanco	Al	37.7474	-2.11651	997
00:00.0	REDIAM-CMA	16592	Observation	Aegilops geniculata		ESP	Spain		Torres	J	37.7807	-3.49505	1133
	REDIAM-CMA	395873	Observation	Aegilops geniculata		ESP	Spain		AlcalÃ¡ de los Ga	Ca	36.535	-5.65004	417
00:00.0	REDIAM-CMA	405186	Observation	Aegilops geniculata		ESP	Spain		Villanueva del Arz	J	38.1643	-2.89883	797
00:00.0	REDIAM-CMA	410717	Observation	Aegilops geniculata		ESP	Spain		Aroche	H	37.9596	-7.05413	300
00:00.0	REDIAM-CMA	418431	Observation	Aegilops geniculata		ESP	Spain		Jayena	Gr	36.9637	-3.75387	1253
	SIVIM	Q-P03110:Aegi	Observation	Aegilops geniculata Roth	Roth	ES	Spain	La Llacuna, sota el DipÃ²sit de les AigÃ¼e			41.45	1.44	0
	SIVIM	Q-P06535:Aegi	Observation	Aegilops geniculata Roth	Roth	ES	Spain	Els Estepars, entre Prades i Albarca			41.26	0.85	0
	SIVIM	Q-P07462:Aegi	Observation	Aegilops geniculata Roth	Roth	ES	Spain	Cervera del Llano			39.74	-2.53	0
	SIVIM	R-P03705:Aegi	Observation	Aegilops geniculata Roth	Roth	ES	Spain	Carretera de Tudela a Ejea de los Caballer			41.99	-1.67	0
	SIVIM	R-P08192:Aegi	Observation	Aegilops geniculata Roth	Roth	ES	Spain	Castell de CastellolÃ-, part culminant d'un t			41.54	1.68	0
	SIVIM	R-P08638:Aegi	Observation	Aegilops geniculata Roth	Roth	ES	Spain	Cerro Lobos			36.77	-2.66	0
00:00.0	BC	70810	Specimen	Aegilops geniculata Roth	Roth	ES	Spain	Burgos; Burgos		Bu	42.32	-3.67	
00:00.0	BC	96443	Specimen	Aegilops ovata L.	L.	ES	Spain	Terrassa; Les fonts de Terrassa		B	41.5	2.1	
1870-06-0	BC	608749	Specimen	Aegilops ovata L.	L.	ES	Spain	Barcelona; Montjuich		B	41.32	2.22	
00:00.0	W	41900	Unknown	Aegilops geniculata Roth		ESP	Spain	Barcelona: RubÃ-, chemin de S. Mus					
00:00.0	BDBCV	597051	Observation	Aegilops geniculata		ESP	Spain		Bocairent	Valenc	38.729	-0.567	
	FUND. BIODIVER	1141314	Unknown	Aegilops ovata subsp. ovata L.	L.	ESP	Spain	Villafranca del Bierzo		Le	42.1	-6.1	
	FUND. BIODIVER	36858	Unknown	Aegilops geniculata Roth	Roth	ESP	Spain	Ocio		Vi	42.1	-2.1	600
	FUND. BIODIVER	54282	Unknown	Aegilops ovata L.	L.	ESP	Spain	Alba de Tormes, cercanÃ-as de la e		Sa	40.1	-5.1	
	FUND. BIODIVER	62021	Unknown	Aegilops ovata subsp. triaristata		ESP	Spain	Retortillo, Gordolobal		Sa	40.1	-6.1	
	SIVIM	R-P10321:Aegi	Observation	Aegilops geniculata Roth	Roth	ES	Spain	Base del Picacho en el refugio , AlcalÃ¡ de			36.47	-5.67	0
	SIVIM	R-P10579:Aegi	Observation	Aegilops geniculata Roth	Roth	ES	Spain	CaÃ±apena , Medina Sidonia			36.46	-5.9	0
	SIVIM	R-P11717:Aegi	Observation	Aegilops geniculata Roth	Roth	ES	Spain	Quintanilla de Trigueros			41.8	-4.68	0
	SIVIM	S-P01261:Aegi	Observation	Aegilops geniculata Roth	Roth	ES	Spain	l'Espluga Calba, Pla de la Creu			41.44	0.96	0
	SIVIM	S-P03977:Aegi	Observation	Aegilops geniculata Roth	Roth	ES	Spain	Blancafort, a les Carreres			41.35	1.08	0
	SEV	11061-1	Specimen	Aegilops ovata L.	L.	ES	Spain	AlcalÃ¡ de GuadaÃ-ra		Se			1
00:00.0	SEV	99213-1	Specimen	Aegilops geniculata Roth	Roth	ES	Spain	Algeciras. La Linea de la ConcepciÃ		Ca			1
00:00.0	BC	92733	Specimen	Aegilops geniculata Roth	Roth	ES	Spain	Alcala de Guadaira; AlcalÃ¡ de Gua		Se	37.33	-5.88	
	BC	92735	Specimen	Aegilops ovata L.	L.	ES	Spain	Eivissa; Pla de Vila Ibiza		PM	38.88	1.44	
00:00.0	MGC	25573-1	Unknown	Aegilops geniculata Roth	Roth	ES	Spain	Entre CÃ¡rtama y Pizarra		Ma	0	0	1
00:00.0	PreservedSpecime	E00086653	Unknown	Aegilops geniculata Roth		ES	Spain						

00:00.0	SALA	112290-1	Specimen	Aegilops geniculata Roth	Roth	ES	Spain	Renedo de Esgueva		Va	41.64	-4.61	
00:00.0	MA	651051-1	Specimen	Aegilops geniculata Roth.	Roth.	ES	Spain	BaÃ±os de la Encina, Casas de Nav		J	38	-3	
00:00.0	MA	552476-1	Specimen	Aegilops geniculta Roth.	Roth.	ES	Spain	Daimiel, Tablas de Daimiel, Isla de A		CR			
00:00.0	GDAC	GDAC32950-1	Specimen	Aegilops geniculata Roth.	Roth.	ES	Spain	Granada, Llano de la Perdiz.		GR			0
00:00.0	MGC	27650-1	Unknown	Aegilops geniculata Roth	Roth	ES	Spain	MÃ¡laga; Alrededores de la Finca de		Ma	36.763	-4.417	1
00:00.0	GDAC	GDAC26135-1	Specimen	Aegilops geniculata Roth.	Roth.	ES	Spain	Granada, SÂª de Baza, Cortijo del F		GR			0
00:00.0	SALA	10063-1	Specimen	Aegilops ovata L. subsp. ovata	L.	ES	Spain	_; Ciudad Rodrigo		Sa			
00:00.0	COFC	41140-1	Specimen	Aegilops geniculata Roth	Roth	ES	Spain	Luque; Laguna del Salobral		Co	37	-4	1
00:00.0	MGC	27655-1	Unknown	Aegilops geniculata Roth	Roth	ES	Spain	Canillas de Aceituno ; Sierra de Tej		Ma	0	0	1
00:00.0	MUB	102378-1	Specimen	Aegilops geniculata Roth	Roth	ES	Spain	Lorca; Campo Coy		Mu	37.947	-1.913	1
	FUND. BIODIVER	1340281	Unknown	Aegilops geniculata Roth	Roth	ESP	Spain	Ranera		Bu	42.1	-3.1	750
	FUND. BIODIVER	70863	Unknown	Aegilops ovata L.	L.	ESP	Spain	La Orbada		Sa	40.1	-5.1	
	FUND. BIODIVER	78792	Unknown	Aegilops geniculata Roth.	Roth.	ESP	Spain	Soto de Cerrato		P	41.1	-4.1	
	RUS001	VIR100602178	Specimen	Aegilops ovata L.		ESP	Spain						
00:00.0	REDIAM-CMA	89374	Observation	Aegilops geniculata		ESP	Spain		Cambil	J	37.7198	-3.49866	1384
	FUND. BIODIVER	1035554	Unknown	Aegilops ovata		ESP	Spain	Zonas elevadas de la Sierra de Agu		Ma	36.1	-4.1	
	FUND. BIODIVER	104259	Unknown	Aegilops ovata L.	L.	ESP	Spain	CastrejÃ³n de la PeÃ±a, entre Castr		P	42.1	-4.1	
	FUND. BIODIVER	1835079	Unknown	Aegilops geniculata Roth	Roth	ESP	Spain	Provincia de Ciudad Real		CR			
	SIVIM	S-P04192:Aegi	Observation	Aegilops geniculata Roth	Roth	ES	Spain	km 3 de la carretera de Balaguer a Agramu			41.71	0.83	280
	SIVIM	S-P09960:Aegi	Observation	Aegilops geniculata Roth	Roth	ES	Spain	coll de la Coveta de la Moscarda, Puig Car			38.54	-0.24	0
	SIVIM	S-P14065:Aegi	Observation	Aegilops geniculata Roth	Roth	ES	Spain	Freixo do Meio			39.38	-8.88	146
	SIVIM	T-P01869:Aegi	Observation	Aegilops geniculata Roth	Roth	ES	Spain	Eivissa: el Pla de Vila, prop de ca n'Escand			38.83	1.38	0
	SIVIM	T-P03855:Aegi	Observation	Aegilops geniculata Roth	Roth	ES	Spain	9 km al WSW de Tarazona			41.81	-1.91	0
	SIVIM	T-P06391:Aegi	Observation	Aegilops geniculata Roth	Roth	ES	Spain	Teulada			39.54	-0.78	200
00:00.0	REDIAM-CMA	383407	Observation	Aegilops geniculata		ESP	Spain				37.7901	-3.19949	
00:00.0	REDIAM-CMA	384571	Observation	Aegilops geniculata		ESP	Spain		El Burgo	Ma	36.7947	-4.99265	1000
00:00.0	REDIAM-CMA	385913	Observation	Aegilops geniculata		ESP	Spain		CoÃn	Ma	36.6545	-4.77487	200
00:00.0	REDIAM-CMA	387038	Observation	Aegilops geniculata		ESP	Spain		Pruna	Se	36.989	-5.19707	738
00:00.0	REDIAM-CMA	388751	Observation	Aegilops geniculata		ESP	Spain		Antequera	Ma	36.98	-4.77917	416
	IPK	AE 698	Living	Aegilops geniculata Roth			Spain	W.-Pyrenaen b. Jaca			42.5719	-0.44972	810
	SIVIM	P-P03209:Aegi	Observation	Aegilops geniculata Roth	Roth	ES	Spain	vall Major, marge de la carretera a Sarroca			41.43	0.48	160
	SIVIM	P-P08864:Aegi	Observation	Aegilops geniculata Roth	Roth	ES	Spain	Riu Matarranya; Beseit			40.79	0.15	0
	SIVIM	P-P08897:Aegi	Observation	Aegilops geniculata Roth	Roth	ES	Spain	Coll Blanc; Horta de Sant Joan			40.88	0.26	0
	SIVIM	P-P09094:Aegi	Observation	Aegilops geniculata Roth	Roth	ES	Spain	de l'Avellanar; CoratxÃ			40.61	0.04	0
	SIVIM	P-P11106:Aegi	Observation	Aegilops geniculata Roth	Roth	ES	Spain	Les Esquarterades			40.62	0.39	125
	SIVIM	Q-P00852:Aegi	Observation	Aegilops geniculata Roth	Roth	ES	Spain	BesalÃº, prop d'Argelaguer i vora el FluviÃ			42.18	2.63	180
00:00.0	GDA	GDA48832-1-2	Specimen	Aegilops geniculata Roth.	Roth.	ES	Spain	AlmerÃa, SÂª de los Filabres, Beni		AL			850
00:00.0	SALA	66707-1	Specimen	Aegilops geniculata Roth	Roth	ES	Spain	_; Peraleda de la Mata		Cc	39.85	-5.47	
	DEU146	AE 1345	Specimen	Aegilops geniculata Roth		ESP	Spain	Sdspanien: Andalusien, Sierranevada, ca. 30 km sdstlich von Granada, Lanjaron					
00:00.0	ABH	32667-1	Specimen	Aegilops geniculata Roth	Roth	ES	Spain	El Campillo; Sierra de Segura		J			
00:00.0	SEV	5989-1	Specimen	Aegilops ovata L.	L.	ES	Spain	Ciudad Universitaria		M			1

00:00.0	SEV	108649-1	Specimen	Aegilops geniculata Roth	Roth	ES	Spain	Entre Rute y Llanos de Don Juan. R		Co			1
	BDBCV-General	79716	Observation	Aegilops geniculata		ESPA	Spain	Burriana	La Plana Baixa	Castel	39.9362	-0.03713	
00:00.0	COFC	11973-1	Specimen	Aegilops geniculata Roth	Roth	ES	Spain	Cabra; entre el municipio y Carcabu		Co	37	-4	1
	IDBD-GN	42425	Observation	Aegilops geniculata Roth	Roth	ES	Spain	Co. S.Pelayo	OrÃ-soain	Na	42.6081	-1.56882	700
	IDBD-GN	42426	Observation	Aegilops geniculata Roth	Roth	ES	Spain	Mte. Gorostiz	Monreal	Na	42.6655	-1.47598	1000
	IDBD-GN	42436	Observation	Aegilops geniculata Roth	Roth	ES	Spain		Ostiz	Na	42.9148	-1.61074	
	BDBCV-General	277334	Observation	Aegilops geniculata		ESPA	Spain	Bolbaite	La Canal de Nava	Valenc	39.0517	-0.74664	
00:00.0	COA	41164-1	Specimen	Aegilops geniculata Roth	Roth	ES	Spain	A 12 Km de Puente Genil desde Sa		Co	37.39	-4.92	
00:00.0	SALA	69023-1	Specimen	Aegilops geniculata Roth	Roth	ES	Spain	_; BÃ³veda del RÃ-o Almar		Sa	40.85	-5.22	
00:00.0	SEV	98681-1	Specimen	Aegilops geniculata Roth	Roth	ES	Spain	Conil de la Frontera. Acantilados de		Ca			30
00:00.0	SEV	98929-1	Specimen	Aegilops geniculata Roth	Roth	ES	Spain	Entre Embid y Las Cuerlas, rÃ-o Pie		Gu			1
	ESP003	NC061688	Specimen	Aegilops geniculata Roth		ESP	Spain	Ciudad Universitaria, Madrid, province of Madrid					
	ESP004	NC050496	Specimen	Aegilops geniculata Roth		ESP	Spain	Beteta/Las Majadas 17km SE, Poyatos, pro			40.4167	-2.03333	1520
	ESP004	NC044552	Specimen	Aegilops geniculata Roth		ESP	Spain	Encinas de Esgueva, province of Valladolid			41.75	-4.1	832
	FUND. BIODIVER	1035699	Unknown	Aegilops ovata		ESP	Spain	Sierra de Aguas, Carratraca		Ma	36.1	-4.1	500
	FUND. BIODIVER	1043753	Unknown	Aegilops geniculata Roth	Roth	ESP	Spain	Canillas de Aceituno		Ma	36.1	-3.1	
	FUND. BIODIVER	1043754	Unknown	Aegilops geniculata Roth	Roth	ESP	Spain	Carratraca, Sierra de Aguas		Ma	36.1	-4.1	
00:00.0	FUND. BIODIVER	1835083	Unknown	Aegilops geniculata Roth	Roth	ESP	Spain	Solana del Pino, alrededores		CR	38.1	-3.1	800
	FUND. BIODIVER	1888171	Unknown	Aegilops geniculata Roth	Roth	ESP	Spain	Sierra del Caballon		V	39.1	-0.1	
	FUND. BIODIVER	116429	Unknown	Aegilops geniculata Roth.	Roth.	ESP	Spain	Aldea de San Miguel		Va	41.1	-4.1	
	FUND. BIODIVER	54857	Unknown	Aegilops geniculata Roth	Roth	ESP	Spain	Macotera		Sa	40.1	-5.1	
00:00.0	REDIAM-CMA	383734	Observation	Aegilops geniculata		ESP	Spain				37.7906	-3.19452	
00:00.0	REDIAM-CMA	48268	Observation	Aegilops geniculata		ESP	Spain		Cambil	J	37.7341	-3.52504	1963
00:00.0	REDIAM-CMA	55399	Observation	Aegilops geniculata		ESP	Spain		CaÃ±ete la Real	Ma	36.9467	-5.09623	600
00:00.0	REDIAM-CMA	65623	Observation	Aegilops geniculata		ESP	Spain		El Burgo	Ma	36.784	-4.90666	854
	SIVIM	P-P08868:Aegi	Observation	Aegilops geniculata Roth	Roth	ES	Spain	Mas de la Caramassa; H. St. Joan			40.88	0.26	0
	SIVIM	P-P08904:Aegi	Observation	Aegilops geniculata Roth	Roth	ES	Spain	Corral de la Bassa; la SÃ©nia			40.7	0.15	1220
	SIVIM	P-P09121:Aegi	Observation	Aegilops geniculata Roth	Roth	ES	Spain	Barranc de les Canals; Arnes			40.88	0.15	0
	SIVIM	P-P11347:Aegi	Observation	Aegilops geniculata Roth	Roth	ES	Spain	Lo PelÃ³s			40.62	0.39	160
	SIVIM	Q-P01675:Aegi	Observation	Aegilops geniculata Roth	Roth	ES	Spain	Bellida, SacaÃ±et			39.81	-0.78	1300
00:00.0	LEB	10101-1	Specimen	Aegilops geniculata Roth	Roth	ES	Spain	Rucayo		Le	42.93	-5.27	1
	SIVIM	R-P09415:Aegi	Observation	Aegilops geniculata Roth	Roth	ES	Spain	Alcolea del Pinar			41.01	-2.52	1200
	SIVIM	R-P10349:Aegi	Observation	Aegilops geniculata Roth	Roth	ES	Spain	Vega del Pradillo , AG			36.38	-5.78	0
	SIVIM	R-P11632:Aegi	Observation	Aegilops geniculata Roth	Roth	ES	Spain	Los Barrios, cortijo de OjÃ©n			42.68	-6.19	0
	SIVIM	S-P01002:Aegi	Observation	Aegilops geniculata Roth	Roth	ES	Spain	Sota CervellÃ³, al vessant esquerre de la ri			41.36	1.92	130
	SIVIM	S-P01266:Aegi	Observation	Aegilops geniculata Roth	Roth	ES	Spain	Fulleda, afores del poble			41.44	0.96	0
	SIVIM	S-P03954:Aegi	Observation	Aegilops geniculata Roth	Roth	ES	Spain	Juncosa, al damunt de la Venta de la Serra			41.25	0.73	0
00:00.0	BC	96446	Specimen	Aegilops ovata L.	L.	ES	Spain	Barcelona; Antiga pineta sobre els E		B	41.41	2.1	
00:00.0	BC	634096	Specimen	Aegilops ovata L.	L.	ES	Spain	Argentona; Argentona		B	41.59	2.34	
00:00.0	HUAL	9906-1	Specimen	Aegilops geniculata Roth	Roth	ES	Spain	HuÃ©rcal-Overa; El Palomar		Al	37.43	-2.01	464
	IPK	60295	Living	Aegilops geniculata Roth subsp. geniculata		ESP	Spain						

00:00.0	BC	830208	Specimen	Aegilops ovata L.	L.	ES	Spain	Palma de Mallorca; Caseta Blanca,		PM	39.16	2.94	
00:00.0	MUB	109110-1	Specimen	Aegilops geniculata Roth	Roth	ES	Spain	Calasparra; BaÃ±os de Gilico		Mu	38.179	-1.636	350
	FUND. BIODIVER	1463976	Unknown	Aegilops geniculata Roth	Roth	ESP	Spain	Sant Linya, vers l'estaciÃ³		L	41.1	0.1	560
	FUND. BIODIVER	1476046	Unknown	Aegilops geniculata Roth.	Roth.	ESP	Spain	Sierra de Santa Pola, Alicante, hast		A	37.1	-0.1	
	FUND. BIODIVER	913740	Unknown	Aegilops ovata L.	L.	ESP	Spain	Massif de Ordal		B	41.1	1.1	
	FUND. BIODIVER	930424	Unknown	Aegilops ovata L.	L.	ESP	Spain	Macizo de La Guardia		T	41.1	0.1	
	FUND. BIODIVER	94006	Unknown	Aegilops geniculata Roth	Roth	ESP	Spain	TÃ¡bara, La BreÃ±ica		Za	41.1	-5.1	
	SIVIM	T-P06429:Aegi	Observation	Aegilops geniculata Roth	Roth	ES	Spain	Sinarcas			39.64	-1.25	0
	SIVIM	T-P07213:Aegi	Observation	Aegilops geniculata Roth	Roth	ES	Spain	Collado de Foradada			42.33	0.32	1015
	SIVIM	T-P09163:Aegi	Observation	Aegilops geniculata Roth	Roth	ES	Spain	Hornillo del Cerrato			41.9	-4.32	0
	SIVIM	T-P09369:Aegi	Observation	Aegilops geniculata Roth	Roth	ES	Spain	Torrelaguna			40.73	-3.59	0
	SIVIM	T-P11405:Aegi	Observation	Aegilops geniculata Roth	Roth	ES	Spain	Cuenca			40.01	-2.17	0
	SIVIM	T-P13156:Aegi	Observation	Aegilops geniculata Roth	Roth	ES	Spain	Montevite			42.81	-2.87	0
	IPK	32167	Living	Aegilops geniculata Roth		ESP	Spain	Pyrenaen					
00:00.0	BC	601431	Specimen	Aegilops ovata L.	L.	ES	Spain	l'Aleixar; Baix Camp: Vers l'Aleixar		T	41.22	1.03	270
00:00.0	GDAC	GDAC37349-1	Specimen	Aegilops geniculata Roth.	Roth.	ES	Spain	AlmerÃa, SÂª de GÃ¡dor, BarjelÃ.		AL			1700
	RUS001	VIR100602105	Specimen	Aegilops ovata L.		ESP	Spain						
	RUS001	VIR100602084	Specimen	Aegilops ovata L.		ESP	Spain						
	FUND. BIODIVER	1093216	Unknown	Aegilops ovata L.	L.	ESP	Spain	Virgen de la MontaÃ±a		Cc	39.1	-6.1	
	FUND. BIODIVER	1115791	Unknown	Aegilops geniculata Rothm.	Rothm.	ESP	Spain	Dehesa de Misleo		Za	41.1	-5.1	
00:00.0	FUND. BIODIVER	1946401	Unknown	Aegilops geniculata Roth	Roth	ESP	Spain	Villanueva de San Carlos, arroyo Ca		CR	38.1	-3.1	600
00:00.0	REDIAM-CMA	3120	Observation	Aegilops geniculata		ESP	Spain		MorÃ³n de la Fron	Se	37.1001	-5.37823	396
00:00.0	REDIAM-CMA	13082	Observation	Aegilops geniculata		ESP	Spain		AlgatocÃn	Ma	36.5651	-5.29199	809
00:00.0	REDIAM-CMA	19264	Observation	Aegilops geniculata		ESP	Spain		Campillos	Ma	37.0195	-4.88715	499
00:00.0	REDIAM-CMA	409105	Observation	Aegilops geniculata		ESP	Spain		Benatae	J	38.356	-2.68545	687
	SIVIM	Q-P03040:Aegi	Observation	Aegilops geniculata Roth	Roth	ES	Spain	La Tossa de Montbui, cim (Anoia)			41.54	1.56	0
	SIVIM	Q-P03607:Aegi	Observation	Aegilops geniculata Roth	Roth	ES	Spain	Villamalur			39.99	-0.42	0
	SIVIM	Q-P06873:Aegi	Observation	Aegilops geniculata Roth	Roth	ES	Spain	Loma BadÃ¡, Petrer,			38.37	-0.82	0
	SIVIM	R-P00087:Aegi	Observation	Aegilops geniculata Roth	Roth	ES	Spain	rodalies de Moror, vers el barranc del Bosc			41.98	0.82	0
	SIVIM	R-P06775:Aegi	Observation	Aegilops geniculata Roth	Roth	ES	Spain	Bormate, Hacia Mahora, Albacete			39.11	-1.72	0
	SIVIM	R-P08278:Aegi	Observation	Aegilops geniculata Roth	Roth	ES	Spain	Sota can Soteres (CastellolÃ)			41.54	1.68	0
00:00.0	GDA	GDA15905-1-1	Specimen	Aegilops geniculata Roth.	Roth.	ES	Spain	Granada, SÂª Pelada, barranco de		GR			980
00:00.0	UPS	V-147335	Specimen	Aegilops ovata		Spain	Spain	Aranjuez - Ontigola		Madrid			
	IDBD-GN	42469	Observation	Aegilops geniculata Roth	Roth	ES	Spain		Garde	Na	42.7457	-0.86312	850
	IDBD-GN	42490	Observation	Aegilops geniculata Roth	Roth	ES	Spain		Viana	Na	42.4985	-2.41105	450
	IDBD-GN	42511	Observation	Aegilops geniculata Roth	Roth	ES	Spain	PeÃ±a El Aguila	Bardenas Reales	Na	42.0287	-1.34031	480
00:00.0	SALA	10134-1	Specimen	Aegilops ovata L. subsp. triaristat	(Willd.) Rouy	ES	Spain	_; Retortillo		Sa			
	FUND. BIODIVER	1696527	Unknown	Aegilops geniculata Roth	Roth	ESP	Spain	Cantavieja		Te	40.1	-0.1	1500
	FUND. BIODIVER	1700453	Unknown	Aegilops geniculata		ESP	Spain	Iglesuela del Cid, Masia de la Loma		Te	40.1	-0.1	1320
00:00.0	REDIAM-CMA	281940	Observation	Aegilops geniculata		ESP	Spain		Hinojosa del Duqu	Co	38.4776	-5.16648	539
00:00.0	REDIAM-CMA	284963	Observation	Aegilops geniculata		ESP	Spain		Torres	J	37.7455	-3.5388	1393

00:00.0	MGC	69932-1	Unknown	Aegilops geniculata Roth	Roth	ES	Spain	VÃ©lez-MÃ¡laga; Almayate. El PeÃ¡		Ma	36.7365	-4.1184	1
	SIVIM	T-P29942:Aegi	Observation	Aegilops geniculata Roth	Roth	ES	Spain	Bajada a Los CÃ¡rceles, Villamalea, Albace			39.38	-1.6	0
	SIVIM	T-P30052:Aegi	Observation	Aegilops geniculata Roth	Roth	ES	Spain	Hoya Alejo, Balsa de Ves, Albacete			39.19	-1.26	0
	SIVIM	T-P30177:Aegi	Observation	Aegilops geniculata Roth	Roth	ES	Spain	Pr. Loma de Palacios, Alborea			39.28	-1.37	0
	SIVIM	U-P02615:Aegi	Observation	Aegilops geniculata Roth	Roth	ES	Spain	Vall d'AlcalÃ , Beniaia			38.72	-0.35	0
	SIVIM	U-P06721:Aegi	Observation	Aegilops geniculata Roth	Roth	ES	Spain	Rocha da Pena			37.22	-8.09	0
00:00.0	PreservedSpecime	E00086652	Unknown	Aegilops geniculata Roth		ES	Spain						
00:00.0	ABH	39341-1	Specimen	Aegilops geniculata Roth	Roth	ES	Spain	Alicante; campos prox. cuartel de R		A	38.37	-0.63	
00:00.0	GDA	GDA15236-1-2	Specimen	Aegilops geniculata Roth.	Roth.	ES	Spain	Granada, carretera Ãlora-MontefrÃ		GR			700
	IDBD-GN	42472	Observation	Aegilops geniculata Roth	Roth	ES	Spain	Foz de Burgui	Burgui	Na	42.7079	-1.01705	600
	IDBD-GN	42493	Observation	Aegilops geniculata Roth	Roth	ES	Spain		ArrÃ³niz	Na	42.5865	-2.09335	500
	DEU146	AE 616	Specimen	Aegilops geniculata Roth		ESP	Spain						
00:00.0	SEV	108253-1	Specimen	Aegilops geniculata Roth	Roth	ES	Spain	Entre Rosa y CadaquÃ©s		Ge			1
	BDBCV-General	275929	Observation	Aegilops geniculata		ESPA	Spain	Bolbaite	La Canal de Nava	Valenc	39.0517	-0.74664	
	BDBCV-General	279171	Observation	Aegilops geniculata		ESPA	Spain	Serra	El Camp de TÃºri	Valenc	39.6745	-0.3768	
	SANT	40704	Specimen	Aegilops geniculata Roth		ES	Spain	Alava: BaÃ±os de Ebro		Vi			
	FUND. BIODIVER	1648483	Unknown	Aegilops geniculata Roth	Roth	ESP	Spain	Iglesuela del Cid, Torre Nicasi		Te	40.1	-0.1	
	SIVIM	T-P30032:Aegi	Observation	Aegilops geniculata Roth	Roth	ES	Spain	Bajada a Pantano del Molinar, Villa de Ves			39.19	-1.26	0
	SIVIM	T-P30057:Aegi	Observation	Aegilops geniculata Roth	Roth	ES	Spain	Los Callejones, Cofrentes, Valencia			39.19	-1.26	0
	SIVIM	T-P31978:Aegi	Observation	Aegilops geniculata Roth	Roth	ES	Spain	Sierra de la Marceral (Campillo de Arenas)			37.49	-3.67	1550
	SIVIM	U-P02905:Aegi	Observation	Aegilops geniculata Roth	Roth	ES	Spain	Castells de Serrella			38.72	-0.23	0
	SIVIM	U-P06644:Aegi	Observation	Aegilops geniculata Roth	Roth	ES	Spain	Zimbreira			37.22	-8.32	180
	SIVIM	U-P06755:Aegi	Observation	Aegilops geniculata Roth	Roth	ES	Spain	Estoi			37.04	-7.98	160
	GDA	GDA43468-1-1	Specimen	Aegilops geniculata Roth.	Roth.	ES	Spain	Granada, Guadix, Rambla Becerra.		GR			950
	ESP004	NC044549	Specimen	Aegilops geniculata Roth		ESP	Spain	Babilafuente, province of Salamanca			40.9667	-5.41667	801
	FUND. BIODIVER	1141586	Unknown	Aegilops geniculata Roth	Roth	ESP	Spain	Puente Castro		Le	42.1	-5.1	910
	FUND. BIODIVER	51164	Unknown	Aegilops ovata subsp. ovata L.	L.	ESP	Spain	San Felices de los Gallegos, ladera		Sa	40.1	-6.1	
	SIVIM	R-P09419:Aegi	Observation	Aegilops geniculata Roth	Roth	ES	Spain	Entre Cambias y Cirueches			41.1	-2.76	0
	SIVIM	R-P10357:Aegi	Observation	Aegilops geniculata Roth	Roth	ES	Spain	El Sauzal , AG			36.47	-5.67	0
	SIVIM	R-P11641:Aegi	Observation	Aegilops geniculata Roth	Roth	ES	Spain	AlcalÃ¡ de los Gazules, Los Santos			36.38	-5.78	1
	SIVIM	S-P01241:Aegi	Observation	Aegilops geniculata Roth	Roth	ES	Spain	Vinaixa, carretera de l'Albi			41.35	0.84	0
	SIVIM	S-P02016:Aegi	Observation	Aegilops geniculata Roth	Roth	ES	Spain	Serra de MonterÃ³			41.8	0.83	0
	SIVIM	S-P03957:Aegi	Observation	Aegilops geniculata Roth	Roth	ES	Spain	l'Espluga Calba, els Bassals			41.44	0.96	0
	SIVIM	T-P02260:Aegi	Observation	Aegilops ovata L.	L.	ES	Spain	Algezares, cerca de los yacimientos de yes			37.93	-1.17	120
	RUS001	VIR100602104	Specimen	Aegilops ovata L.		ESP	Spain						
	RUS001	VIR100602083	Specimen	Aegilops ovata L.		ESP	Spain						
00:00.0	GDA	GDA30018-1-1	Specimen	Aegilops ovata L. var. leptostachy	L.	ES	Spain	Cabrera, puerto.		PM			0
	FUND. BIODIVER	1090545	Unknown	Aegilops ovata L.	L.	ESP	Spain	Villarejo de SalvanÃ©s		M	39.1	-3.1	
	FUND. BIODIVER	1093217	Unknown	Aegilops ovata L.	L.	ESP	Spain	Valle de Plasencia		Cc	39.1	-5.1	
	FUND. BIODIVER	1093238	Unknown	Aegilops geniculata		ESP	Spain	Cerro de Aldeamoret		Cc	39.1	-6.1	
	FUND. BIODIVER	1115792	Unknown	Aegilops geniculata Rothm.	Rothm.	ESP	Spain	Puente Quintos		Za	41.1	-5.1	

00:00.0	FUND. BIODIVER	1946402	Unknown	Aegilops geniculata Roth	Roth	ESP	Spain	Villanueva de la Fuente		CR	38.1	-2.1	1000
00:00.0	REDIAM-CMA	3225	Observation	Aegilops geniculata		ESP	Spain		Antequera	Ma	36.9427	-4.77629	598
00:00.0	REDIAM-CMA	13615	Observation	Aegilops geniculata		ESP	Spain		Pruna	Se	37.0199	-5.18736	786
00:00.0	REDIAM-CMA	19502	Observation	Aegilops geniculata		ESP	Spain		Marbella	Ma	36.5376	-4.93711	284
00:00.0	REDIAM-CMA	26755	Observation	Aegilops geniculata		ESP	Spain		Torres	J	37.7455	-3.53878	1393
00:00.0	REDIAM-CMA	397073	Observation	Aegilops geniculata		ESP	Spain		AlcalÃ¡ de los Ga	Ca	36.4807	-5.72213	108
00:00.0	REDIAM-CMA	416431	Observation	Aegilops geniculata		ESP	Spain		Quesada	J	37.8048	-2.97588	1800
00:00.0	REDIAM-CMA	420722	Observation	Aegilops geniculata		ESP	Spain		Hinojosa del Duq	Co	38.4794	-5.31874	528
	SIVIM	Q-P03042:Aegi	Observation	Aegilops geniculata Roth	Roth	ES	Spain	La Llacuna, part marginal d'un carrer no pa			41.45	1.44	0
	SIVIM	Q-P03608:Aegi	Observation	Aegilops geniculata Roth	Roth	ES	Spain	Torralba del Pinar			39.99	-0.54	0
	SIVIM	Q-P06902:Aegi	Observation	Aegilops geniculata Roth	Roth	ES	Spain	Batech, Petrer,			38.46	-0.82	0
	SIVIM	R-P06792:Aegi	Observation	Aegilops geniculata Roth	Roth	ES	Spain	Jorquera, hacia AlcalÃ¡ del JÃºcar 5 Km, A			39.1	-1.49	0
	SIVIM	R-P08446:Aegi	Observation	Aegilops geniculata Roth	Roth	ES	Spain	El Calabrial			36.86	-2.77	1400
00:00.0	MGC	32137-1	Unknown	Aegilops geniculata Roth	Roth	ES	Spain	IstÃ¡n; Nacimiento de RÃo Verde		Ma	0	0	1
00:00.0	MGC	33778-1	Unknown	Aegilops geniculata Roth	Roth	ES	Spain	Grazalema; Cerca de la Cara del BA		Ca	0	0	1
00:00.0	MA	553358-1	Specimen	Aegilops ovata L.	L.	ES	Spain	OrduÃ±a, camino bajo de la Virgen		Bi			
	RUS001	VIR100602118	Specimen	Aegilops ovata L.		ESP	Spain						
	RUS001	VIR100602097	Specimen	Aegilops ovata L.		ESP	Spain						
	RUS001	VIR100602076	Specimen	Aegilops ovata L.		ESP	Spain						
00:00.0	SALA	124534-1	Specimen	Aegilops geniculata Roth	Roth	ES	Spain	Torreadrada		Sg	41.43	-5.05	
00:00.0	BDBCV	594063	Observation	Aegilops geniculata		ESP	Spain		Alcoy	Alicant	38.665	-0.523	
	FUND. BIODIVER	1085535	Unknown	Aegilops geniculata		ESP	Spain	Loeches		M	40.1	-3.1	
	FUND. BIODIVER	1093224	Unknown	Aegilops ovata L.	L.	ESP	Spain	MontaÃ±a y Calerizo de CÃ¡ceres		Cc	39.1	-6.1	
	FUND. BIODIVER	110577	Unknown	Aegilops geniculata Roth	Roth	ESP	Spain	Cervera de Pisuerga, Celada de Ro		P	42.1	-4.1	
00:00.0	FUND. BIODIVER	1946388	Unknown	Aegilops geniculata Roth	Roth	ESP	Spain	Herencia, de Herencia a Villarta de		CR	39.1	-3.1	700
00:00.0	REDIAM-CMA	7225	Observation	Aegilops geniculata		ESP	Spain		Montellano	Se	36.9646	-5.52123	299
00:00.0	REDIAM-CMA	410524	Observation	Aegilops geniculata		ESP	Spain		GÃ©nave	J	38.4182	-2.74533	799
00:00.0	REDIAM-CMA	414513	Observation	Aegilops geniculata		ESP	Spain		Baza	Gr	37.2365	-2.69532	1947
00:00.0	REDIAM-CMA	417019	Observation	Aegilops geniculata		ESP	Spain		Tabernas	Al	37.0856	-2.33728	483
	SIVIM	Q-P03084:Aegi	Observation	Aegilops geniculata Roth	Roth	ES	Spain	La Llacuna (Anoia)			41.45	1.44	0
	SIVIM	Q-P04629:Aegi	Observation	Aegilops geniculata Roth	Roth	ES	Spain	Vilanova de la Muga, a tocar de les extracc			42.27	3	0
	SIVIM	Q-P07448:Aegi	Observation	Aegilops geniculata Roth	Roth	ES	Spain	Honrubia			39.56	-2.3	0
	SIVIM	R-P03515:Aegi	Observation	Aegilops geniculata Roth	Roth	ES	Spain	Agost			38.37	-0.71	0
	SIVIM	R-P06817:Aegi	Observation	Aegilops geniculata Roth	Roth	ES	Spain	AlcalÃ¡ del JÃºcar, Albacete			39.1	-1.49	0
	SIVIM	R-P08599:Aegi	Observation	Aegilops geniculata Roth	Roth	ES	Spain	CaparidÃ¡n			36.86	-3	1600
00:00.0	MA	558591-1	Specimen	Aegilops geniculata Roth.	Roth.	ES	Spain	Alto Tajo, Finca Belvalle		Cu	40	-1	
00:00.0	COFC	41264-1	Specimen	Aegilops geniculata Roth	Roth	ES	Spain	Baena; cerro AlbendÃƒÂ-n		Co	37	-4	1
00:00.0	MA	719340-1	Specimen	Aegilops geniculata Roth	Roth	ES	Spain	AlcÃ¡ntara, regato de Remolinas, ve		Cc	39	-6	
00:00.0	MGC	40318-1	Unknown	Aegilops geniculata Roth	Roth	ES	Spain	Parauta; Cerro Serrana		Ma	36.671	-5.132	1100
	IPK	96762	Living	Aegilops geniculata Roth		ESP	Spain	Sudspanien: Andalusien, Sierranevada, ca. 30 km sudostlich von Granada, Lanja					
	DEU146	AE 833	Specimen	Aegilops geniculata subsp. geniculata Roth		ESP	Spain						

00:00.0	SEV	99078-1	Specimen	Aegilops geniculata Roth	Roth	ES	Spain	San NicolÃ¡s del Puerto. Cerro del H		Se			670
	FUND. BIODIVER	1377175	Unknown	Aegilops geniculata Roth	Roth	ESP	Spain	Vilardesilva		Or	42.1	-6.1	450
	FUND. BIODIVER	1463978	Unknown	Aegilops geniculata Roth	Roth	ESP	Spain	Serra d'Arquells		L	41.1	0.1	500
	FUND. BIODIVER	913768	Unknown	Aegilops ovata L.	L.	ESP	Spain	Serra Superior del Valles		B	41.1	2.1	
00:00.0	SALA	52033-1	Specimen	Aegilops geniculata Roth	Roth	ES	Spain	_; Abezames, Gafos		Za			
00:00.0	REDIAM-CMA	144537	Observation	Aegilops geniculata		ESP	Spain		Santa Olalla del C	H	37.9026	-6.19197	510
00:00.0	REDIAM-CMA	162426	Observation	Aegilops geniculata		ESP	Spain		Berja	Al	36.8899	-2.9924	609
00:00.0	REDIAM-CMA	186821	Observation	Aegilops geniculata		ESP	Spain		Carboneros	J	38.2668	-3.68864	400
00:00.0	COFC	7352-1	Specimen	Aegilops geniculata Roth	Roth	ES	Spain	Valle del Guadalmellato; puente Mo		Co			1
	SIVIM	T-P06438:Aegi	Observation	Aegilops geniculata Roth	Roth	ES	Spain	Chiva			39.45	-0.79	300
	SIVIM	T-P07215:Aegi	Observation	Aegilops geniculata Roth	Roth	ES	Spain	Ballobar			41.51	0.12	180
	SIVIM	T-P09166:Aegi	Observation	Aegilops geniculata Roth	Roth	ES	Spain	Cogeces de Iscar			41.45	-4.55	0
	SIVIM	T-P09795:Aegi	Observation	Aegilops geniculata Roth	Roth	ES	Spain	carretera de Toledo a Navahermosa			39.55	-4.51	0
	SIVIM	T-P11408:Aegi	Observation	Aegilops geniculata Roth	Roth	ES	Spain	Tragacete			40.28	-1.82	1240
	SIVIM	T-P13168:Aegi	Observation	Aegilops geniculata Roth	Roth	ES	Spain	Sanguesa			42.52	-1.29	0
00:00.0	MGC	20417-1	Unknown	Aegilops geniculata Roth	Roth	ES	Spain	Alhama de Granada; Sierra Tejeda.		Gr	36.956	-4.028	1
00:00.0	MGC	21970-1	Unknown	Aegilops geniculata Roth	Roth	ES	Spain	Entre El Gandul y Trujillo		Se	0	0	1
00:00.0	ABH	10053-1	Specimen	Aegilops geniculata Roth	Roth	ES	Spain	Petrer; camino Petrer-RincÃ³n Bello		A	38.49	-0.73	
00:00.0	BDBCV	103	Observation	Aegilops geniculata		ESP	Spain	Parque Natural de Penyagolosa		Cs	40.18	-0.35	
00:00.0	GDA	GDA16011-1-1	Specimen	Aegilops geniculata Roth	Roth	ES	Spain	Granada, SÂª de Madrid, Lagunazo		GR			1200
	IDBD-GN	42471	Observation	Aegilops geniculata Roth	Roth	ES	Spain		GÃ¼esa	Na	42.7992	-1.0875	680
	IDBD-GN	42480	Observation	Aegilops geniculata Roth	Roth	ES	Spain	La Blanca	Bardenas Reales	Na	43.1113	-1.48339	290
	IDBD-GN	42492	Observation	Aegilops geniculata Roth	Roth	ES	Spain		MaÃ±eru	Na	42.6744	-1.86019	600
	IPK	70813	Living	Aegilops geniculata Roth		ESP	Spain	Cortiguera (Burgos)					
00:00.0	MGC	67703-1	Unknown	Aegilops geniculata Roth	Roth	ES	Spain	Monda; Sierra Alpujata. Entre los al		Ma	36.586	-4.816	910
00:00.0	MA	626752-1	Specimen	Aegilops ovata L.	L.	ES	Spain	Pista de Fredes al monte Caro, barr		Cs	40	0	
	MA	630045-1	Specimen	Aegilops geniculata Roth	Roth	ES	Spain	CamaleÃ±o, pr. Mogrovejo		S	43	-4	
1896-08-2	BC	70785	Specimen	Aegilops ovata L.	L.	ES	Spain	Monreal del Campo; Monreal del Ca		Te	40.78	-1.4	
	BC	92730	Specimen	Aegilops ovata L.	L.	ES	Spain	Puigverd de Lleida; Puigvert LÃ©rid		L	41.58	0.78	
00:00.0	BC	92739	Specimen	Aegilops geniculata Roth	Roth	ES	Spain	Puerto Real; Marquesado Cadiz		Ca	36.51	-6.04	
00:00.0	MA	772820-1	Specimen	Aegilops geniculata Roth.	Roth.	ES	Spain	Santaella; km 19-20 de la carretera		Co			
00:00.0	BDBCV	590004	Observation	Aegilops geniculata		ESP	Spain		Alcoy	Alicant	38.693	-0.568	
	FUND. BIODIVER	1060398	Unknown	Aegilops ovata		ESP	Spain	Los Barrios		Ca	35.1	-5.1	
	FUND. BIODIVER	1067627	Unknown	Aegilops ovata L.	L.	ESP	Spain	Pedroso		Se	37.1	-5.1	
00:00.0	FUND. BIODIVER	1835076	Unknown	Aegilops geniculata Roth	Roth	ESP	Spain	Lagunas de Ruidera, borde de la lag		CR	38.1	-2.1	
	FUND. BIODIVER	1895398	Unknown	Aegilops geniculata Roth.	Roth.	ESP	Spain	Valle de Mena, Irus		Bu	42.1	-3.1	540
00:00.0	REDIAM-CMA	384284	Observation	Aegilops geniculata		ESP	Spain				37.5778	-3.19644	
00:00.0	REDIAM-CMA	385714	Observation	Aegilops geniculata		ESP	Spain		Torres	J	37.7471	-3.52848	1500
00:00.0	REDIAM-CMA	386925	Observation	Aegilops geniculata		ESP	Spain		Pruna	Se	36.9757	-5.18493	600
00:00.0	REDIAM-CMA	388626	Observation	Aegilops geniculata		ESP	Spain		Torres	J	37.7472	-3.52846	1500
00:00.0	REDIAM-CMA	391903	Observation	Aegilops geniculata		ESP	Spain		Luque	Co	37.5279	-4.2658	729

00:00.0	REDIAM-CMA	394884	Observation	Aegilops geniculata		ESP	Spain		Puente de GÃ©na	J	38.3397	-2.81073	672
00:00.0	GDAC	GDAC38757-1	Specimen	Aegilops geniculata Roth.	Roth.	ES	Spain	Granada, Vega de Granada.		GR			0
	SIVIM	P-P08808:Aegi	Observation	Aegilops geniculata Roth	Roth	ES	Spain	Barranc de Canals; PaÃ¼ls			40.89	0.38	0
	SIVIM	P-P08813:Aegi	Observation	Aegilops geniculata Roth	Roth	ES	Spain	PaÃ¼ls, rodalies			40.89	0.38	310
	SIVIM	P-P08891:Aegi	Observation	Aegilops geniculata Roth	Roth	ES	Spain	Mas de les Eres; Horta de Sant Joan			40.88	0.26	0
	SIVIM	P-P09019:Aegi	Observation	Aegilops geniculata Roth	Roth	ES	Spain	La Vallcanera; la SÃ©nia			40.7	0.15	1200
	SIVIM	P-P11098:Aegi	Observation	Aegilops geniculata Roth	Roth	ES	Spain	Corral de l'Ama			40.34	0.29	190
	SIVIM	P-P11102:Aegi	Observation	Aegilops geniculata Roth	Roth	ES	Spain	Corral de l'Ama			40.34	0.29	190
	SIVIM	Q-P00143:Aegi	Observation	Aegilops geniculata Roth	Roth	ES	Spain	la Figuera			41.16	0.73	0
00:00.0	SEV	100519-1	Specimen	Aegilops geniculata Roth	Roth	ES	Spain	Priego de CÃ³rdoba. Alrededores de		Co			1
00:00.0	SEV	108143-1	Specimen	Aegilops geniculata Roth	Roth	ES	Spain	Almodovar. Camino vecinal de los T		Co			1
00:00.0	SEV	108261-1	Specimen	Aegilops geniculata Roth	Roth	ES	Spain	Almedinilla. RÃ-o Almedinilla en las		Co			1
	BDBCV-General	274780	Observation	Aegilops geniculata		ESPA	Spain	Chella	La Canal de Nava	Valenc	39.0517	-0.74664	
	FUND. BIODIVER	1554585	Unknown	Aegilops ovata L.	L.	ESP	Spain	Briviesca		Bu	41.1	-3.1	
	FUND. BIODIVER	987187	Unknown	Aegilops ovata subsp. eu-murinu	Brig.	ESP	Spain	Soria		So	41.1	-2.1	
00:00.0	REDIAM-CMA	198481	Observation	Aegilops geniculata		ESP	Spain		Cazorla	J	37.882	-2.96264	1339
00:00.0	REDIAM-CMA	236385	Observation	Aegilops geniculata		ESP	Spain		Fuente Obejuna	Co	38.2147	-5.38945	683
00:00.0	GDA	GDA30020-1-1	Specimen	Aegilops ovata L.	L.	ES	Spain	Granada, SÂª Elvira.		GR			0
	SIVIM	T-P13728:Aegi	Observation	Aegilops geniculata Roth	Roth	ES	Spain	Masico Bielsa, Mosqueruela			40.35	-0.52	1560
	SIVIM	T-P16912:Aegi	Observation	Aegilops geniculata Roth	Roth	ES	Spain	JÃ¡baga			40.01	-2.29	0
	SIVIM	T-P19969:Aegi	Observation	Aegilops geniculata Roth	Roth	ES	Spain	Finca de Araya (Arroyo de la Luz)			39.45	-6.67	0
	SIVIM	T-P25249:Aegi	Observation	Aegilops geniculata Roth	Roth	ES	Spain	Llucalari (Menorca)			39.83	4.05	0
	SIVIM	T-P27423:Aegi	Observation	Aegilops geniculata Roth	Roth	ES	Spain	OntÃ-gola			39.92	-3.58	0
	SIVIM	T-P28641:Aegi	Observation	Aegilops geniculata Roth	Roth	ES	Spain	Arroyo del Pino, BelvÃ-s de Monroy			39.8	-5.68	280
	GDA	GDA30013-1-2	Specimen	Aegilops geniculata Roth.	Roth.	ES	Spain	Madrid, Aranjuez.		M			0
00:00.0	MA	552233-1	Specimen	Aegilops geniculata Roth.	Roth.	ES	Spain	Daimiel, Tablas de Daimiel, isla de		CR			
	GDA	GDA42773-1	Specimen	Aegilops geniculata Roth.	Roth.	ES	Spain	Granada, CÃºllar Baza, El Margen,		GR			872
00:00.0	HUAL	1130-1	Specimen	Aegilops geniculata Roth	Roth	ES	Spain	SÂª de Cazorla, Quesada		J	37.835	-3.063	
	IPK	32099	Living	Aegilops geniculata Roth		ESP	Spain						
	DEU146	AE 973	Specimen	Aegilops geniculata Roth		ESP	Spain	Col de Veleta, Sierra Nevada,Prov. de Granada, Espagne					
	BDBCV-General	276666	Observation	Aegilops geniculata		ESPA	Spain	NavarrÃ©s	La Canal de Nava	Valenc	39.0517	-0.74664	
	FUND. BIODIVER	1648488	Unknown	Aegilops geniculata Roth	Roth	ESP	Spain	Puertomingalvo, El Letrado		Te	40.1	-0.1	
00:00.0	REDIAM-CMA	250299	Observation	Aegilops geniculata		ESP	Spain		AlÃ¡jar	H	37.8303	-6.64405	399
00:00.0	REDIAM-CMA	279159	Observation	Aegilops geniculata		ESP	Spain		CÃ³rdoba	Co	37.9443	-4.66266	154
	SIVIM	T-P30041:Aegi	Observation	Aegilops geniculata Roth	Roth	ES	Spain	Losa del Hocico, Casas de Ves, Albacete			39.19	-1.37	0
	SIVIM	T-P30062:Aegi	Observation	Aegilops geniculata Roth	Roth	ES	Spain	Pr. Las Salinas, Casas de Ves, Albacete			39.28	-1.37	0
	SIVIM	T-P30063:Aegi	Observation	Aegilops geniculata Roth	Roth	ES	Spain	Pr. Las Salinas, Casas de Ves, Albacete			39.28	-1.37	0
	SIVIM	U-P00814:Aegi	Observation	Aegilops geniculata Roth	Roth	ES	Spain	Prado de La GuzpeÃ±a			42.7	-5.07	1090
	SIVIM	U-P02911:Aegi	Observation	Aegilops geniculata Roth	Roth	ES	Spain	Vall d'AlcalÃ			38.72	-0.23	0
	SIVIM	U-P06651:Aegi	Observation	Aegilops geniculata Roth	Roth	ES	Spain	CabeÃ§o da CÃ¢mara			37.04	-8.1	0
	SIVIM	U-P06652:Aegi	Observation	Aegilops geniculata Roth	Roth	ES	Spain	Cerro dos Fatos			37.04	-8.1	1000

	SIVIM	U-P07719:Aegi	Observation	Aegilops geniculata Roth	Roth	ES	Spain	Valencia: Pugol, motorway A-7 borders			39.53	-0.32	10
00:00.0	MA	750086-1	Specimen	Aegilops geniculata Roth	Roth	ES	Spain	Covarrubias, carretera a Mecerreye		Bu	42	-3	
00:00.0	GDA	GDA54083-1-1	Specimen	Aegilops geniculata Roth.	Roth.	ES	Spain	Granada, Alhama, carril al cortijo El		GR			1100
00:00.0	SEV	99211-1	Specimen	Aegilops geniculata Roth	Roth	ES	Spain	Sierra de Rute. Arroyo cercano al C		Co			1
00:00.0	MGC	25571-1	Unknown	Aegilops geniculata Roth	Roth	ES	Spain	La Lantejuela-El Rubio		Ma	0	0	1
00:00.0	ABH	41689-1	Specimen	Aegilops geniculata Roth	Roth	ES	Spain	Alicante; Cabo de Huertas		A	38.35	-0.41	
00:00.0	ABH	51243-1	Specimen	Aegilops geniculata Roth	Roth	ES	Spain	ArchipiÃ©lago de Cabrera; Can Feli		Mll	39.14	2.94	
00:00.0	GDA	GDA19440-1-2	Specimen	Aegilops geniculata Roth.	Roth.	ES	Spain	JaÃ©n, Colegio Universitario.		J			440
00:00.0	GDA	GDA22404-1-2	Specimen	Aegilops geniculata Roth.	Roth.	ES	Spain	Granada, Cogollos Vega, proximida		GR			1000
	IDBD-GN	42487	Observation	Aegilops geniculata Roth	Roth	ES	Spain		Korres	Vi	42.6967	-2.42139	700
	IDBD-GN	42509	Observation	Aegilops geniculata Roth	Roth	ES	Spain		Yesa	Na	42.6206	-1.17834	540
00:00.0	BDBCV	332216	Observation	Aegilops geniculata	_	ES	Spain	Tossal del Captiva	La Nucia	A	38.59	-0.07	
00:00.0	MGC	59844-1	Unknown	Aegilops geniculata Roth	Roth	ES	Spain	Arenas del Rey; P. N. de las Sierras		Gr	36.913	-3.87	1050
00:00.0	BDBCV	589417	Observation	Aegilops geniculata		ESP	Spain		Alcoy	Alicant	38.728	-0.532	
00:00.0	BDBCV	589902	Observation	Aegilops geniculata		ESP	Spain		Alcoy	Alicant	38.675	-0.569	
00:00.0	COFC	46882-1	Specimen	Aegilops geniculata Roth	Roth	ES	Spain	arroyo Guadalbaida; ctra hacia Sevi		Co			1
	FUND. BIODIVER	1715684	Unknown	Aegilops geniculata		ESP	Spain	Peraleda, marge del cami entre San		Ge	42.1	2.1	
00:00.0	REDIAM-CMA	315392	Observation	Aegilops geniculata		ESP	Spain		Cuevas del Becer	Ma	36.879	-5.01529	698
00:00.0	REDIAM-CMA	335103	Observation	Aegilops geniculata		ESP	Spain		Los Barrios	Ca	36.1946	-5.44261	44
00:00.0	REDIAM-CMA	381578	Observation	Aegilops geniculata		ESP	Spain				37.5814	-3.27229	
00:00.0	REDIAM-CMA	382723	Observation	Aegilops geniculata		ESP	Spain		Casares	Ma	36.3841	-5.32553	80
	SIVIM	U-P08621:Aegi	Observation	Aegilops geniculata Roth	Roth	ES	Spain	La Cella (Jumilla)			38.38	-1.51	0
00:00.0	BC	866348	Specimen	Aegilops ovata L.	L.	ES	Spain	Puebla Tornesa; La Pobla Tornesa		Cs	40.0703	-0.04864	
	DEU146	AE 698	Specimen	Aegilops geniculata Roth		ESP	Spain	W.-Pyrenen b. Jaca					810
00:00.0	BDBCV	111	Observation	Aegilops geniculata		ESP	Spain	Parque Natural de Penyagolosa		Cs			
00:00.0	SEV	108248-1	Specimen	Aegilops geniculata Roth	Roth	ES	Spain	Rute. Subida a Cierzos Altos, desde		Co			1
00:00.0	SEV	108284-1	Specimen	Aegilops geniculata Roth	Roth	ES	Spain	Arroyo Guadalbaida		Co			1
	BDBCV-General	264837	Observation	Aegilops geniculata		ESPA	Spain	Ayora	El Valle de Ayora	Valenc	39.0538	-0.86212	
	BDBCV-General	272104	Observation	Aegilops geniculata		ESPA	Spain	Real de Montroi	La Ribera Alta	Valenc	39.3218	-0.73799	
00:00.0	MA	584695-1	Specimen	Aegilops geniculata Roth.	Roth.	ES	Spain	Los Villares		J			
	W	42309	Unknown	Aegilops ovata L.		ESP	Spain	PyrÃ©nÃ©es Espagnoles. Barcelona, Ã Martorelle.					
00:00.0	HUAL	5849-1	Specimen	Aegilops geniculata Roth	Roth	ES	Spain	Caniles; SÂª de Baza-Los Filabres,		Gr	37.366	-2.735	
00:00.0	COFC	46893-1	Specimen	Aegilops geniculata Roth	Roth	ES	Spain	ctra de Palma del RÃƒÂ-o, Km-5; ar		Co			1
	FUND. BIODIVER	1811874	Unknown	Aegilops geniculata Roth	Roth	ESP	Spain	Loma Negra		Na	41.1	-1.1	
00:00.0	REDIAM-CMA	299653	Observation	Aegilops geniculata		ESP	Spain		BaÃ±os de la Enc	J	38.3541	-3.8397	603
00:00.0	REDIAM-CMA	328569	Observation	Aegilops geniculata		ESP	Spain		Casares	Ma	36.4632	-5.30306	270
00:00.0	REDIAM-CMA	363907	Observation	Aegilops geniculata		ESP	Spain		LubrÃ-n	Al	37.1956	-2.03129	600
00:00.0	REDIAM-CMA	382164	Observation	Aegilops geniculata		ESP	Spain		Antequera	Ma	36.9426	-4.77625	598
00:00.0	REDIAM-CMA	383103	Observation	Aegilops geniculata		ESP	Spain				37.7446	-3.29064	
	SIVIM	U-P08139:Aegi	Observation	Aegilops geniculata Roth	Roth	ES	Spain	La Nava, Berzocana			39.36	-5.55	0
	SIVIM	U-P09953:Aegi	Observation	Aegilops geniculata Roth	Roth	ES	Spain	Carretera de Ossa a Ruidera			38.93	-2.76	0

00:00.0	BDBCV	114	Observation	Aegilops geniculata		ESP	Spain	Parque Natural de Penyagolosa		Cs			
1868-06-0	BC	70795	Specimen	Aegilops ovata L.	L.	ES	Spain	Barcelona; de Barcelona a Badalon		B	41.41	2.22	
00:00.0	COA	41160-1	Specimen	Aegilops geniculata Roth	Roth	ES	Spain	De Moriles a Monturque		Co	37.39	-4.69	
00:00.0	SEV	71065-1	Specimen	Aegilops geniculata Roth	Roth	ES	Spain	Algeciras. Cerro del Rayo. Cantera		Ca			200
00:00.0	GDAC	GDAC31113-1	Specimen	Aegilops geniculata Roth.	Roth.	ES	Spain	AlmerÃ-a, Llanos de Tabernas, alre		AL			500
	BC	608751	Specimen	Aegilops ovata L.	L.	ES	Spain	Barcelona; Pedralbes		B	41.41	2.1	
	RUS001	VIR100602117	Specimen	Aegilops ovata L.		ESP	Spain						
	RUS001	VIR100602096	Specimen	Aegilops ovata L.		ESP	Spain						
	RUS001	VIR100602075	Specimen	Aegilops ovata L.		ESP	Spain						
00:00.0	SALA	124553-1	Specimen	Aegilops geniculata Roth	Roth	ES	Spain	Merindad de Valdeporres, Quintana		Bu	42.96	-4.94	
	FUND. BIODIVER	1077194	Unknown	Aegilops ovata L.	L.	ESP	Spain	OntÃ-gola		To	39.1	-3.1	
	FUND. BIODIVER	1085536	Unknown	Aegilops geniculata		ESP	Spain	Ciudad Universitaria		M	40.1	-3.1	
	FUND. BIODIVER	1093225	Unknown	Aegilops ovata		ESP	Spain	Peraleda de la Mata, Toril		Cc	39.1	-5.1	
00:00.0	FUND. BIODIVER	1946389	Unknown	Aegilops geniculata Roth	Roth	ESP	Spain	La Solana, sierra de Alhambra, arro		CR	38.1	-2.1	
00:00.0	REDIAM-CMA	7861	Observation	Aegilops geniculata		ESP	Spain		Cambil	J	37.6928	-3.51003	1061
00:00.0	REDIAM-CMA	15189	Observation	Aegilops geniculata		ESP	Spain		Pruna	Se	36.9888	-5.19692	738
00:00.0	REDIAM-CMA	21580	Observation	Aegilops geniculata		ESP	Spain		Torres	J	37.7376	-3.50344	1624
00:00.0	REDIAM-CMA	399101	Observation	Aegilops geniculata		ESP	Spain		La Puebla de los	Se	37.7601	-5.4931	200
00:00.0	REDIAM-CMA	410640	Observation	Aegilops geniculata		ESP	Spain		GÃ©nave	J	38.4675	-2.77183	770
00:00.0	REDIAM-CMA	414659	Observation	Aegilops geniculata		ESP	Spain		Baza	Gr	37.458	-2.878	1146
00:00.0	REDIAM-CMA	417031	Observation	Aegilops geniculata		ESP	Spain		Tabernas	Al	37.0931	-2.33943	509
	MA	566169-1	Specimen	Aegilops ovata L.	L.	ES	Spain	Aranjuez		M			
00:00.0	HSS	12597	Specimen	Aegilops geniculata Roth	Roth	ES	Spain	Villafranca de los Barros		Ba	38.5924	-6.30208	
	SIVIM	Q-P03093:Aegi	Observation	Aegilops geniculata Roth	Roth	ES	Spain	Solsona, marge de camp (SolsonÃ¨s)			41.99	1.43	0
	SIVIM	Q-P04652:Aegi	Observation	Aegilops geniculata Roth	Roth	ES	Spain	Siurana, entre el mas Brava i Baseia			42.18	3	0
	SIVIM	Q-P07458:Aegi	Observation	Aegilops geniculata Roth	Roth	ES	Spain	Olmedilla de AlarcÃ³n			39.56	-2.18	0
	SIVIM	R-P03682:Aegi	Observation	Aegilops geniculata Roth	Roth	ES	Spain	Carretera de Arguedas a Tudela			42.08	-1.67	0
	SIVIM	R-P06818:Aegi	Observation	Aegilops geniculata Roth	Roth	ES	Spain	Casas de Ves, Barranco de Mingo, Albacet			39.1	-1.38	0
	SIVIM	R-P08600:Aegi	Observation	Aegilops geniculata Roth	Roth	ES	Spain	BarjalÃ-			36.86	-2.88	1650
00:00.0	SALA	83850-1	Specimen	Aegilops geniculata Roth	Roth	ES	Spain	_; La FuensaviÃ±an		Gu			
00:00.0	SEV	99079-1	Specimen	Aegilops geniculata Roth	Roth	ES	Spain	Carretera de Sevilla a MorÃ³n. RÃ-o		Se			1
	ESP004	NC024042	Specimen	Aegilops geniculata Roth		ESP	Spain	Puerto de la Mora, Huetor-Santillan, provin			37.2333	-3.48333	1265
	FUND. BIODIVER	1186718	Unknown	Aegilops ovata L.	L.	ESP	Spain	Son Coll-Banyalbufar		PM	39.1	2.1	
	FUND. BIODIVER	120458	Unknown	Aegilops geniculata Roth	Roth	ESP	Spain	Montejo de la Vega de la Serrezuela		Sg	41.1	-3.1	
	FUND. BIODIVER	80851	Unknown	Aegilops geniculata Roth	Roth	ESP	Spain	Carrascal del RÃ-o, Carrascal del R		Sg	41.1	-3.1	
	FUND. BIODIVER	996412	Unknown	Triticum ovatum G.G.	G.G.	ESP	Spain	Puente Vadillos		Cu	40.1	-2.1	
00:00.0	REDIAM-CMA	93163	Observation	Aegilops geniculata		ESP	Spain		Cabra	Co	37.4915	-4.37514	1000
	SIVIM	S-P12764:Aegi	Observation	Aegilops geniculata Roth	Roth	ES	Spain	Fitero , Navarra ,			41.99	-1.91	0
	SIVIM	T-P03844:Aegi	Observation	Aegilops geniculata Roth	Roth	ES	Spain	1 km al SW de Almiruete			41.01	-3.23	1010
	SIVIM	T-P04209:Aegi	Observation	Aegilops geniculata Roth	Roth	ES	Spain	1 km al SW de Moratalla			38.12	-1.97	0
	SIVIM	T-P06420:Aegi	Observation	Aegilops geniculata Roth	Roth	ES	Spain	Siete Aguas			39.46	-0.9	0

00:00.0	BC	102135	Specimen	Aegilops ovata L.	L.	ES	Spain	Palma de Mallorca; debajo de Sa Vi		PM	39.16	2.94	
00:00.0	BC	634092	Specimen	Aegilops geniculata Roth	Roth	ES	Spain	MatarÃ³; MatarÃ³ Vistalegre		B	41.5502	2.400648	
00:00.0	MGC	67834-1	Unknown	Aegilops geniculata Roth	Roth	ES	Spain	MÃ¡laga; Campus de Teatinos		Ma	0	0	1
00:00.0	BDBCV	100	Observation	Aegilops geniculata		ESP	Spain	Parque Natural de Penyagolosa		Cs	40.25	-0.31	
00:00.0	BDBCV	119	Observation	Aegilops geniculata		ESP	Spain	Parque Natural de Penyagolosa		Cs	40.22	-0.41	
00:00.0	UNEX	11782-1	Observation	Aegilops geniculata Roth	_	ESP	Spain	Motril: Cabo de Sacratif.		Gr	36.7445	-3.48726	
	SEV	10641-1	Specimen	Aegilops ovata L.	L.	ES	Spain	Aranjuez, Cerro del Parnasso		M			1
	SANT	42238	Specimen	Aegilops geniculata Roth		ES	Spain	Castellvi de Rosanes		B			
00:00.0	GDA	GDA22403-1	Specimen	Aegilops geniculata Roth.	Roth.	ES	Spain	Granada, Cogollos Vega, proximida		GR			1100
00:00.0	ABH	3071-1	Specimen	Aegilops geniculata Roth	Roth	ES	Spain	Vistabella del Maestrazgo; alred. de		Cs	40.32	-0.34	
	IDBD-GN	42432	Observation	Aegilops geniculata Roth	Roth	ES	Spain	Bardena Blanca	Bardenas Reales	Na	42.2153	-1.48686	
	IDBD-GN	42452	Observation	Aegilops geniculata Roth	Roth	ES	Spain	Asiain	Olza	Na	42.8498	-1.83861	
	ADIMAN	36	Observation	Aegilops geniculata		ESP	Spain	EnguÃdanos		CU	39.6771	-1.60451	
	BDBCV-General	279879	Observation	Aegilops geniculata		ESPA	Spain	Teresa de Cofren	El Valle de Ayora	Valenc	39.146	-0.97503	
00:00.0	GDAC	GDAC41825-1	Specimen	Aegilops geniculata Roth.	Roth.	ES	Spain	CÃ³rdoba, SÂª Morena, Barranco R		CO			400
00:00.0	BC	92726	Specimen	Aegilops ovata L.	L.	ES	Spain	GuimerÃ ; GuimerÃ¡		L	41.58	1.14	
	FUND. BIODIVER	1036558	Unknown	Aegilops ovata		ESP	Spain	MÃ¡laga		Ma	36.1	-4.1	
	FUND. BIODIVER	1043759	Unknown	Aegilops geniculata Roth	Roth	ESP	Spain	Nerja		Ma	36.1	-3.1	
	FUND. BIODIVER	1050666	Unknown	Aegilops ovata L.	L.	ESP	Spain	RegiÃ³n inferior y submontana		Ca			
	FUND. BIODIVER	1066943	Unknown	Aegilops ovata L.	L.	ESP	Spain	MorÃ³n		Se	36.1	-5.1	
	FUND. BIODIVER	1072130	Unknown	Aegilops ovata L.	L.	ESP	Spain	Pruna, AlgÃ¡mitas, Sierra del TablÃ		Se	36.1	-4.1	
00:00.0	FUND. BIODIVER	1835089	Unknown	Aegilops geniculata Roth	Roth	ESP	Spain	Alamillo, laderas del cerro del Tamb		CR	38.1	-4.1	600
00:00.0	REDIAM-CMA	383889	Observation	Aegilops geniculata		ESP	Spain				37.6699	-3.42674	
00:00.0	REDIAM-CMA	384932	Observation	Aegilops geniculata		ESP	Spain		Alpandeire	Ma	36.668	-5.22667	959
00:00.0	REDIAM-CMA	384972	Observation	Aegilops geniculata		ESP	Spain		AlbÃ¡nchez de MA	J	37.7874	-3.48674	1200
00:00.0	REDIAM-CMA	388204	Observation	Aegilops geniculata		ESP	Spain		CaÃ±ete la Real	Ma	36.9835	-5.02187	715
00:00.0	REDIAM-CMA	389921	Observation	Aegilops geniculata		ESP	Spain		Carcabuey	Co	37.4127	-4.3319	751
	SIVIM	P-P08342:Aegi	Observation	Aegilops geniculata Roth	Roth	ES	Spain	Lo Sotet (Massalcoreig)			41.42	0.24	0
	SIVIM	P-P08873:Aegi	Observation	Aegilops geniculata Roth	Roth	ES	Spain	CamÃ de la Gavalda; Tortosa			40.79	0.27	1100
	SIVIM	P-P08925:Aegi	Observation	Aegilops geniculata Roth	Roth	ES	Spain	Coll de l'AssucÃ ; Roquetes			40.7	0.27	1180
	SIVIM	P-P09746:Aegi	Observation	Aegilops geniculata Roth	Roth	ES	Spain	CoratxÃ			40.61	0.04	1210
	SIVIM	P-P12716:Aegi	Observation	Aegilops geniculata Roth	Roth	ES	Spain	Refugio de la UmbrÃa			37.67	-2.2	1400
00:00.0	GDA	GDA48364-1-2	Specimen	Aegilops ovata L.	L.	ES	Spain	Guadalajara, ArbancÃ³n, Barranco d		GU			950
00:00.0	GDA	GDA7741-1	Specimen	Aegilops ovata L. var. pubiglumis	L.	ES	Spain	Madrid, La Moncloa.		M			0
	STU	Main-1-4616	Specimen	Aegilops geniculata Roth		ES	Spain	Kreuzung La Losa-Puebla-Santiago, WP 27					
00:00.0	GDAC	GDAC37573-1	Specimen	Aegilops geniculata Roth.	Roth.	ES	Spain	Granada, Parque Natural de la SÂª		GR			1700
00:00.0	BC	132783	Specimen	Aegilops ovata L.	L.	ES	Spain	Palma de Mallorca; Mallorca: Son R		PM	39.61	2.59	
00:00.0	SEV	108249-1	Specimen	Aegilops geniculata Roth	Roth	ES	Spain	Alrededores de Jauja		Co			1
00:00.0	SEV	108285-1	Specimen	Aegilops geniculata Roth	Roth	ES	Spain	BÃ©lmez. Lomas del Pedregosillo		Co			1
	BDBCV-General	272105	Observation	Aegilops geniculata		ESPA	Spain	Real de Montroi	La Ribera Alta	Valenc	39.3195	-0.62208	
	FUND. BIODIVER	1549487	Unknown	Aegilops ovata		ESP	Spain	utraque Cast. (Burgos)		Bu	42.1	-3.1	

	FUND. BIODIVER	1558123	Unknown	Aegilops geniculata		ESP	Spain	Reinoso de Cerrato		P	41.1	-4.1	850
	FUND. BIODIVER	1564979	Unknown	Aegilops geniculata		ESP	Spain	Soto de Cerrato		P	41.1	-4.1	810
	FUND. BIODIVER	1600822	Unknown	Aegilops geniculata		ESP	Spain	Valderas, Monte del Duque		Le	41.1	-5.1	760
	FUND. BIODIVER	1643498	Unknown	Aegilops geniculata Roth	Roth	ESP	Spain	Juslibol		Z	41.1	-0.1	230
	FUND. BIODIVER	979129	Unknown	Aegilops ovata L.	L.	ESP	Spain	CercanÃ-as de Teruel		Te	40.1	-0.1	
	FUND. BIODIVER	261210	Unknown	Aegylops ovata L.	L.	ESP	Spain	La Palma		Tf	28.1	-17.1	
00:00.0	REDIAM-CMA	215548	Observation	Aegilops geniculata		ESP	Spain		Huesa	J	37.7221	-3.10966	678
	SIVIM	T-P13664:Aegi	Observation	Aegilops geniculata Roth	Roth	ES	Spain	La Barraca, Mosqueruela			40.35	-0.4	1100
	SIVIM	T-P16882:Aegi	Observation	Aegilops geniculata Roth	Roth	ES	Spain	Ribatejada			40.64	-3.47	0
	SIVIM	T-P19059:Aegi	Observation	Aegilops geniculata Roth	Roth	ES	Spain	Proximidades a NarvÃ¡ez			37.31	-2.88	1200
	SIVIM	T-P20229:Aegi	Observation	Aegilops geniculata Roth	Roth	ES	Spain	Alcaudete, lÃ-mite con la provincia de CÃ³r			37.58	-4.13	0
	SIVIM	T-P26221:Aegi	Observation	Aegilops geniculata Roth	Roth	ES	Spain	Arenales de Las Virtudes, Villena			38.56	-0.93	0
	SIVIM	T-P28614:Aegi	Observation	Aegilops geniculata Roth	Roth	ES	Spain	Finca de los Cuartos, ValdecaÃ±as de Tajo			39.71	-5.68	320
00:00.0	SALA	40752-1	Specimen	Aegilops geniculata Roth	Roth	ES	Spain	_; FuentidueÃ±a		Sg			
00:00.0	MGC	60410-1	Unknown	Aegilops geniculata Roth	Roth	ES	Spain	Nerja; P. N. de las Sierras de Tejed		Ma	36.787	-3.79	500
00:00.0	COFC	46894-1	Specimen	Aegilops geniculata Roth	Roth	ES	Spain	Posadas; arroy de la Vega en ctra d		Co			1
	FUND. BIODIVER	1811875	Unknown	Aegilops geniculata Roth	Roth	ESP	Spain	Milagro		Na	42.1	-1.1	
00:00.0	REDIAM-CMA	303012	Observation	Aegilops geniculata		ESP	Spain		Jimera de LÃ-bar	Ma	36.6435	-5.29242	449
00:00.0	REDIAM-CMA	382183	Observation	Aegilops geniculata		ESP	Spain				37.7037	-3.21633	
	SIVIM	U-P08142:Aegi	Observation	Aegilops geniculata Roth	Roth	ES	Spain	Collado del Bote, La Nava, Berzocana			39.36	-5.55	0
	SIVIM	U-P09956:Aegi	Observation	Aegilops geniculata Roth	Roth	ES	Spain	Cuneta cerca de Casas Blancas			38.84	-3	0
00:00.0	BDBCV	116	Observation	Aegilops geniculata		ESP	Spain	Parque Natural de Penyagolosa		Cs			
00:00.0	GDA	GDA48228-1	Specimen	Aegilops geniculata Roth	Roth	ES	Spain	Granada, SÂª de las Chanzas, Mon		GR			900
00:00.0	BC	92761	Specimen	Aegilops ovata L.	L.	ES	Spain	Eivissa; Ibiza		PM	38.88	1.44	
00:00.0	BC	606724	Specimen	Aegilops ovata L.	L.	ES	Spain	Sunyer; SegriÃ : Sunyer		L	41.48	0.54	
	ESP004	NC027428	Specimen	Aegilops geniculata Roth		ESP	Spain	Ciudad Encantada, Cuenca, province of Cu			40.2	-2.01667	1320
00:00.0	MUB	102381-1	Specimen	Aegilops geniculata Roth	Roth	ES	Spain	Yecla; Las Atalayas		Mu	38.7	-1.22	820
	FUND. BIODIVER	1189434	Unknown	Aegilops ovata L.	L.	ESP	Spain	CamÃ- Ses Cases d'Es Port		PM	38.1	2.1	
	FUND. BIODIVER	70866	Unknown	Aegilops ovata L.	L.	ESP	Spain	Aldeanueva de Figueroa		Sa	40.1	-5.1	
	FUND. BIODIVER	856220	Unknown	Aegilops ovata		ESP	Spain	Alicante, Talaia-Rambla Ovelles		A	38.1	-0.1	
00:00.0	REDIAM-CMA	106905	Observation	Aegilops geniculata		ESP	Spain		Siles	J	38.4637	-2.57869	1184
	REDIAM-CMA	116370	Observation	Aegilops geniculata		ESP	Spain		AlcalÃ¡ de los Ga	Ca	36.3777	-5.65329	50
00:00.0	REDIAM-CMA	127763	Observation	Aegilops geniculata		ESP	Spain		Cazorla	J	37.9223	-2.82109	1667
	SIVIM	S-P05489:Aegi	Observation	Aegilops geniculata Roth	Roth	ES	Spain	Granollers de la Plana, serrat de Puig-rodÃ			41.9	2.15	0
	SIVIM	S-P09963:Aegi	Observation	Aegilops geniculata Roth	Roth	ES	Spain	pr. Confrides			38.63	-0.35	0
	SIVIM	S-P14072:Aegi	Observation	Aegilops geniculata Roth	Roth	ES	Spain	Freixo do Meio			39.38	-8.88	156
	SIVIM	T-P01881:Aegi	Observation	Aegilops geniculata Roth	Roth	ES	Spain	Mallorca: obac de CÃºber			39.74	2.76	0
	SIVIM	T-P04072:Aegi	Observation	Aegilops geniculata Roth	Roth	ES	Spain	8 km al N de JÃ³dar			37.85	-3.45	300
	SIVIM	T-P06404:Aegi	Observation	Aegilops geniculata Roth	Roth	ES	Spain	Los Pedrones			39.28	-1.14	0
	IPK	31849	Living	Aegilops geniculata Roth subsp. gibberosa (Zh		ESP	Spain	Formentor, Majorque, Baleares					
00:00.0	GDA	GDA10271-1	Specimen	Aegilops lorentii Hochst.	Hochst.	ES	Spain	Granada, SÂª Nevada, CherÃ-n, ca		GR			650

	FUND. BIODIVER	1027212	Unknown	Aegilops biuncialis Vis.	Vis.	ESP	Spain	Marmolejo		J	37.1	-3.1	200
00:00.0	SEV	100338-1	Specimen	Aegilops lorentii Hochst.	Hochst.	ES	Spain	Entre Venta de los Yesos y Taberna		Al			450
	FUND. BIODIVER	1029113	Unknown	Aegilops lorentii Hochst.	Hochst.	ESP	Spain	AndÃºjar, desembocadura del JÃ¡nc		J	37.1	-3.1	200
00:00.0	SEV	82914-1	Specimen	Aegilops lorentii Hochst.	Hochst.	ES	Spain	AlmerÃ-a. Rambla, cerca de su des		Al			1
	FUND. BIODIVER	1376734	Unknown	Aegilops lorentii Hochst.	Hochst.	ESP	Spain	Sierra de Baza, Parque Natural		Gr	37.1	-2.1	
	GDAC	GDAC29636-1	Specimen	Aegilops lorentii Hochst.	Hochst.	ES	Spain	Granada, SÂª de Parapanda, carret		GR			1000
00:00.0	BC	615226	Specimen	Aegilops biuncialis Vis.	Vis.	ES	Spain	Fraga; Baix Cinca: La Serreta Negra		Hu	41.38	-0.07	
	IPK	32165	Living	Aegilops lorentii Hochst. var. velutina (Zhuk.) K	ESP		Spain	Zaorejas					
00:00.0	GDA	GDA16649-1	Specimen	Aegilops lorentii Hochst.	Hochst.	ES	Spain	Granada, SÂª de Parapanda, carret		GR			750
	FUND. BIODIVER	1946408	Unknown	Aegilops lorentii Hochst	Hochst	ESP	Spain	Lagunas de Ruidera, laguna Cenag		CR	38.1	-2.1	
00:00.0	SEV	102784-1	Specimen	Aegilops lorentii Hochst.	Hochst.	ES	Spain	Entre Sorbas y Tabernas. Venta de		Al			500
00:00.0	GDA	GDA18052-1	Specimen	Aegilops lorentii Hochst.	Hochst.	ES	Spain	Granada, SÂª Nevada, Monachil, ba		GR			1600
00:00.0	HUAL	6057-1	Specimen	Aegilops lorentii Hochst.	Hochst.	ES	Spain	SÂª de GÃ¡dor, BalsÃ³n de Las Hoy		Al	36.879	-2.815	
	ESP004	NC043507	Specimen	Aegilops lorentii Hochst.		ESP	Spain	Los Santos de la Humosa, province of Mad			40.5	-3.25	906
00:00.0	SEV	108147-1	Specimen	Aegilops lorentii Hochst.	Hochst.	ES	Spain	10 Km E of Tabernas. Venta de los		Al			520
	ESP004	NC050490	Specimen	Aegilops lorentii Hochst.		ESP	Spain	casa forestal de Tejadillos, Cuenca, provin			40.4	-1.98333	1080
00:00.0	REDIAM-CMA	51568	Observation	Aegilops biuncialis		ESP	Spain		Zufre	H	37.768	-6.4605	477
	FUND. BIODIVER	1029112	Unknown	Aegilops lorentii Hochst.	Hochst.	ESP	Spain	Villanueva del Arzobispo a los Olmi		J	37.1	-2.1	620
00:00.0	GDA	GDA16059-1-1	Specimen	Aegilops lorentii Hochst.	Hochst.	ES	Spain	Granada, SÂª de Obeilar, cortijo de		GR			700
00:00.0	GDA	GDA16059-1	Specimen	Aegilops lorentii Hochst.	Hochst.	ES	Spain	Granada, SÂª de Obeilar, cortijo de		GR			700
	FUND. BIODIVER	1029114	Unknown	Aegilops lorentii Hochst.	Hochst.	ESP	Spain	AndÃºjar, RÃ-o Valmayor, Valdelag		J	38.1	-4.1	500
00:00.0	GDAC	GDAC26142-1	Specimen	Aegilops lorentii Hochst.	Hochst.	ES	Spain	Granada, SÂª de Baza, Cortijo de B		GR			0
	FUND. BIODIVER	1487851	Unknown	Aegilops lorentii Hochst.	Hochst.	ESP	Spain	P.N. Sierra Nevada		Gr	36.1	-3.1	
	FUND. BIODIVER	1029115	Unknown	Aegilops lorentii Hochst.	Hochst.	ESP	Spain	AndÃºjar, desembocadura del Valde		J	38.1	-4.1	400
00:00.0	GDA	GDA12220-1-1	Specimen	Aegilops lorentii Hochst.	Hochst.	ES	Spain	Granada, SÂª Nevada, CÃ¡Ã±ar, ba		GR			1100
00:00.0	GDAC	GDAC14186-1	Specimen	Aegilops lorentii Hochst.	Hochst.	ES	Spain	AlmerÃ-a, El Perdigal.		AL			0
00:00.0	GDA	GDA16649-1-1	Specimen	Aegilops lorentii Hochst.	Hochst.	ES	Spain	Granada, SÂª de Parapanda, carret		GR			750
00:00.0	COA	41205-1	Specimen	Aegilops biuncialis Vis.	Vis.	ES	Spain	JardÃ-n BotÃ¡nico de CÃ³rdoba		Co	37.84	-4.82	
00:00.0	SEV	89993-1	Specimen	Aegilops lorentii Hochst.	Hochst.	ES	Spain	Los Barrios		Ca			1
00:00.0	REDIAM-CMA	397187	Observation	Aegilops biuncialis		ESP	Spain		Antequera	Ma	37.0212	-4.52803	521
	FUND. BIODIVER	1086009	Unknown	Aegilops lorentii Hochst.	Hochst.	ESP	Spain	Arganda del Rey		M	40.1	-3.1	
00:00.0	MA	647980-1	Specimen	Aegilops lorentii Hochst.	Hochst.	ES	Spain	El Alquian		Al	36	-2	
	FUND. BIODIVER	1835092	Unknown	Aegilops lorentii Hochst	Hochst	ESP	Spain	Lagunas de Ruidera, laguna Cenag		CR	38.1	-2.1	
1899-12-3	REDIAM-CMA	390897	Observation	Aegilops lorentii		ESP	Spain		NÃ-jar	Al	36.8109	-2.06205	15
	DEU146	AE 685	Specimen	Aegilops lorentii Hochst. var. velutina (Zhuk.) K	ESP		Spain	Zaorejas					
00:00.0	GDA	GDA12220-1	Specimen	Aegilops lorentii Hochst.	Hochst.	ES	Spain	Granada, SÂª Nevada, CÃ¡Ã±ar, ba		GR			1100
	IPK	AE 685	Living	Aegilops lorentii Hochst. var. velutina (Zhuk.) K.Hamr			Spain	Zaorejas			40.7664	-2.8	
	FUND. BIODIVER	1013131	Unknown	Aegilops lorentii Hoschst.	Hoschst.	ESP	Spain	Llano de la Charca del Sabinal		Al	36.1	-2.1	
00:00.0	GDA	GDA10272-1-1	Specimen	Aegilops lorentii Hochst.	Hochst.	ES	Spain	Granada, SÂª Nevada, LanjarÃ³n, a		GR			700
	ESP004	NC050501	Specimen	Aegilops lorentii Hochst.		ESP	Spain	CaÃƒÂ±averas/Priego 5km NE, Villaconejo			40.4	-2.33333	880
00:00.0	GDAC	GDAC26141-1	Specimen	Aegilops lorentii Hochst.	Hochst.	ES	Spain	Granada, SÂª de Baza, entre La Ca		GR			0

00:00.0	GDA	GDA10271-1-1	Specimen	Aegilops lorentii Hochst.	Hochst.	ES	Spain	Granada, SÂª Nevada, CherÃ-n, ca		GR			650
	CZE122	01C2101247	Specimen	Aegilops lorentii var. velutina (ZHUK.) HAMME		ESP	Spain	Zaorejas					
	FUND. BIODIVER	58086	Unknown	Aegilops lorentii Hochst	Hochst	ESP	Spain	Cantalapiedra		Sa	40.1	-5.1	
00:00.0	GDA	GDA10272-1	Specimen	Aegilops lorentii Hochst.	Hochst.	ES	Spain	Granada, SÂª Nevada, LanjarÃ³n, a		GR			700
00:00.0	BC	92749	Specimen	Aegilops biuncialis Vis.	Vis.	ES	Spain	Madrid; Cerro Negro, Madrid		M	40.42	-3.65	
00:00.0	GDA	GDA18052-1-1	Specimen	Aegilops lorentii Hochst.	Hochst.	ES	Spain	Granada, SÂª Nevada, Monachil, ba		GR			1600
	GDAC	GDAC29633-1	Specimen	Aegilops lorentii Hochst.	Hochst.	ES	Spain	Granada, base de la SÂª de Parapa		GR			1000
	FUND. BIODIVER	1093240	Unknown	Aegilops neglecta		ESP	Spain	Cerro de Aldeamoret		Cc	39.1	-6.1	
	FUND. BIODIVER	1093262	Unknown	Aegilops neglecta		ESP	Spain	Villanueva de la Vera		Cc	39.1	-5.1	
00:00.0	FUND. BIODIVER	1946426	Unknown	Aegilops neglecta Req. ex Bertol	Req. ex Bert	ESP	Spain	Viso del Marques, Camino Real de		CR	38.1	-3.1	840
00:00.0	REDIAM-CMA	13972	Observation	Aegilops neglecta		ESP	Spain		Aroche	H	37.9182	-7.06018	252
00:00.0	REDIAM-CMA	19893	Observation	Aegilops neglecta		ESP	Spain		Cambil	J	37.7326	-3.50653	1627
00:00.0	REDIAM-CMA	397747	Observation	Aegilops neglecta		ESP	Spain		CardeÃ±a	Co	38.1896	-4.28181	724
	SIVIM	S-P00124:Aegi	Observation	Aegilops neglecta Req. ex Bertol.	Req. ex Bert	ES	Spain	*			41.54	2.28	150
	SIVIM	S-P02910:Aegi	Observation	Aegilops neglecta Req. ex Bertol.	Req. ex Bert	ES	Spain	VallÃ¨s: entre Terrassa et Matadepera, vers			41.54	1.92	0
	SIVIM	S-P04845:Aegi	Observation	Aegilops neglecta Req. ex Bertol.	Req. ex Bert	ES	Spain	cerca de Fuentes			41.43	-0.72	0
	SIVIM	S-P06264:Aegi	Observation	Aegilops neglecta Req. ex Bertol.	Req. ex Bert	ES	Spain	del SE de Linares de Mora			40.26	-0.64	0
	FUND. BIODIVER	1835104	Unknown	Aegilops neglecta Req. ex Bertol	Req. ex Bert	ESP	Spain	Provincia de Ciudad Real		CR			
00:00.0	REDIAM-CMA	383732	Observation	Aegilops neglecta		ESP	Spain				37.7906	-3.19446	
	SIVIM	Q-P02279:Aegi	Observation	Aegilops neglecta Req. ex Bertol.	Req. ex Bert	ES	Spain	Ciutadella: Macarella			39.92	3.93	20
	SIVIM	Q-P07552:Aegi	Observation	Aegilops neglecta Req. ex Bertol.	Req. ex Bert	ES	Spain	Villaescusa de Haro, castillo sobre el rÃ-o Z			39.56	-2.76	0
	SANT	57125	Specimen	Aegilops neglecta Req. ex Bertol.		ES	Spain	Tielmes (Madrid)		M			
00:00.0	COFC	46897-1	Specimen	Aegilops neglecta Req. ex Bertol.	Req. ex Bert	ES	Spain	Hornachuelos; ctra de San Calixto,		Co			1
00:00.0	COFC	46898-1	Specimen	Aegilops neglecta Req. ex Bertol.	Req. ex Bert	ES	Spain	rÃƒÂ-o NÃƒÂ©valo; paredones de '		Co			1
00:00.0	REDIAM-CMA	306235	Observation	Aegilops neglecta		ESP	Spain		Fuente de Piedra	Ma	37.1153	-4.79047	414
	SIVIM	U-P08101:Aegi	Observation	Aegilops neglecta Req. ex Bertol.	Req. ex Bert	ES	Spain	EstaciÃ³n Ferrocarril, LogrosÃ¡n			39.27	-5.55	0
00:00.0	SEV	108291-1	Specimen	Aegilops neglecta Req. ex Bertol.	Req. ex Bert	ES	Spain	Jeez de la Frontera		Ca			1
00:00.0	REDIAM-CMA	196327	Observation	Aegilops neglecta		ESP	Spain		Espiel	Co	38.1379	-5.03336	591
00:00.0	REDIAM-CMA	203908	Observation	Aegilops neglecta		ESP	Spain		Constantina	Se	37.8959	-5.68622	608
00:00.0	COFC	52514-1	Specimen	Aegilops neglecta Req. ex Bertol.	Req. ex Bert	ES	Spain	Moraleja; _		Cc	0	0	1
	SIVIM	T-P17808:Aegi	Observation	Aegilops neglecta Req. ex Bertol.	Req. ex Bert	ES	Spain	OntÃ-gola			39.92	-3.58	0
	SIVIM	T-P18904:Aegi	Observation	Aegilops neglecta Req. ex Bertol.	Req. ex Bert	ES	Spain	AlcalÃ¡ de Henares, finca La Clota			40.46	-3.47	0
	SIVIM	T-P19973:Aegi	Observation	Aegilops neglecta Req. ex Bertol.	Req. ex Bert	ES	Spain	Cerro de Aldeamoret (CÃ¡ceres)			39.36	-6.44	0
00:00.0	SEV	101365-1	Specimen	Aegilops neglecta Req. ex Bertol.	Req. ex Bert	ES	Spain	Constantina. Carretera de El Pedros		Se			500
	ESP004	NC043467	Specimen	Aegilops neglecta REQ. ex BERTOL.		ESP	Spain	Collado Villalba, province of Madrid			40.6333	-3.98333	917
00:00.0	UNEX	30188-1	Observation	Aegilops neglecta Req. ex Bertol.	_	ESP	Spain	Magacela: El Berrocal, berceales. 4		Ba	38.8	-5.8	
	FUND. BIODIVER	1331445	Unknown	Aegilops neglecta Req.	Req.	ESP	Spain	Comarca de la Vera		Cc	39.1	-5.1	
00:00.0	HUAL	13299-1	Specimen	Aegilops neglecta Req.ex Bertol s	(Vis) Asch. &	ES	Spain	Tabernas; Al este de la Sartenilla		Al	37.022	-2.41	360
00:00.0	REDIAM-CMA	203203	Observation	Aegilops neglecta		ESP	Spain		AlanÃ-s	Se	38.0682	-5.6412	582
	SIVIM	T-P18898:Aegi	Observation	Aegilops neglecta Req. ex Bertol.	Req. ex Bert	ES	Spain	AlcalÃ¡ de Henares, finca La Clota			40.46	-3.47	0
	SIVIM	T-P19971:Aegi	Observation	Aegilops neglecta Req. ex Bertol.	Req. ex Bert	ES	Spain	Cerro de Aldeamoret (CÃ¡ceres)			39.36	-6.44	0

	SIVIM	T-P20224:Aegi	Observation	Aegilops neglecta Req. ex Bertol.	Req. ex Bert	ES	Spain	Puente Genil			37.3	-4.8	150
	SIVIM	T-P17780:Aegi	Observation	Aegilops neglecta Req. ex Bertol.	Req. ex Bert	ES	Spain	Tielmes			40.19	-3.35	0
	SIVIM	T-P19970:Aegi	Observation	Aegilops neglecta Req. ex Bertol.	Req. ex Bert	ES	Spain	Cerro de Aldeamoret (CÃ¡ceres)			39.36	-6.44	0
	SIVIM	T-P20223:Aegi	Observation	Aegilops neglecta Req. ex Bertol.	Req. ex Bert	ES	Spain	BailÃ©n			38.03	-3.79	340
00:00.0	HUAL	5785-1	Specimen	Aegilops neglecta Req. ex Bertol.	Req. ex Bert	ES	Spain	Tabernas; SÂª de Los Filabres, Los		Al	37.112	-2.342	
	SIVIM	Q-P00067:Aegi	Observation	Aegilops neglecta Req. ex Bertol.	Req. ex Bert	ES	Spain	vessant obac de la vall de la Coma, encreu			41.43	0.6	240
	SIVIM	Q-P02001:Aegi	Observation	Aegilops neglecta Req. ex Bertol.	Req. ex Bert	ES	Spain	Sant Joan			40.17	-0.41	1275
00:00.0	BC	652329	Specimen	Aegilops neglecta Req. ex Bertol.	Req. ex Bert	ES	Spain	Cazorla; Fuente del Oso, Sierra de		J	37.916	-2.937	
00:00.0	REDIAM-CMA	268945	Observation	Aegilops neglecta		ESP	Spain		Villanueva de CÃ	Co	38.2141	-4.59238	715
00:00.0	REDIAM-CMA	240934	Observation	Aegilops neglecta		ESP	Spain		Constantina	Se	37.8561	-5.50723	500
00:00.0	REDIAM-CMA	261446	Observation	Aegilops neglecta		ESP	Spain		Villanueva de CÃ	Co	38.1805	-4.656	633
	SIVIM	T-P27005:Aegi	Observation	Aegilops neglecta Req. ex Bertol.	Req. ex Bert	ES	Spain	*			42.08	-1.54	0
	SIVIM	T-P27017:Aegi	Observation	Aegilops neglecta Req. ex Bertol.	Req. ex Bert	ES	Spain	*			41.62	-1.31	320
00:00.0	HSS	13195	Specimen	Aegilops neglecta Req. ex Bertol	Req. ex Bert	ES	Spain	BaÃ±os de Montemayor		Cc	40.2996	-5.88265	
00:00.0	UNEX	10367-1	Observation	Aegilops neglecta Req. ex Bertol.	_	ESP	Spain	Badajoz: Altozanos de la carretera		Ba	38.8	-7.00001	
00:00.0	COFC	50175-1	Specimen	Aegilops neglecta Req. ex Bertol.	Req. ex Bert	ES	Spain	Valle del Guadiato; puente de la Ca		Co			1
	DEU146	AE 590	Specimen	Aegilops neglecta Req. ex Bertol. subsp. recta		ESP	Spain	Finca La Cigueela, stlich Aracena, Huelva					
	DEU146	AE 583	Specimen	Aegilops neglecta Req. ex Bertol. subsp. recta		ESP	Spain	bei Monasterio de Yuste, Sierra de Gredos, Cceres					
00:00.0	SALA	82102-1	Specimen	Aegilops neglecta Req. ex Bertol	Req. ex Bert	ES	Spain	_; Valverde del Fresno		Cc	40.27	-7	
	CZE122	01C2108005	Specimen	Aegilops neglecta subsp. recta (ZHUK.) HAMM		ESP	Spain	S Plasencia, Caceres					
00:00.0	SEV	108288-1	Specimen	Aegilops neglecta Req. ex Bertol.	Req. ex Bert	ES	Spain	Guadalcanal. Finca de las Monjas		Se			700
00:00.0	MGC	22231-1	Unknown	Aegilops neglecta Req.	Req.	ES	Spain	MÃ¡laga; Cerro de San AntÃ³n		Ma	0	0	1
00:00.0	COA	41228-1	Specimen	Aegilops neglecta Req. ex Bertol.	Req. ex Bert	ES	Spain	JardÃ-n BotÃ¡nico de CÃ³rdoba		Co	37.84	-4.82	
00:00.0	SEV	108289-1	Specimen	Aegilops neglecta Req. ex Bertol.	Req. ex Bert	ES	Spain	Entre Las Pajanosas y El Ronquillo,		Se			1
00:00.0	COFC	21308-1	Specimen	Aegilops neglecta Req. ex Bertol.	Req. ex Bert	ES	Spain	Sierra de Hornachuelos; 'Alta Baja'		Co			1
00:00.0	GDA	GDA10272-1-3	Specimen	Aegilops neglecta Req ex Bertol	Req ex Berto	ES	Spain	Granada, SÂª Nevada, LanjarÃ³n, a		GR			700
00:00.0	COFC	21304-1	Specimen	Aegilops neglecta Req. ex Bertol.	Req. ex Bert	ES	Spain	Sierra de Hornachuelos; 'Los Arena		Co			1
00:00.0	MA	722767-1	Specimen	Aegilops neglecta Req. ex Bertol	Req. ex Bert	ES	Spain	San Lorenzo de Calatrava, umbrÃ-a		CR	38	-3	
	ESP004	NC043470	Specimen	Aegilops neglecta REQ. ex BERTOL.		ESP	Spain	Alcornoquillo, Alhama de Granada, provinc			36.9333	-4.01667	1078
00:00.0	FUND. BIODIVER	1946416	Unknown	Aegilops neglecta Req. ex Bertol	Req. ex Bert	ESP	Spain	Mestanza, valle del rio Robledillo		CR	38.1	-3.1	440
00:00.0	REDIAM-CMA	110603	Observation	Aegilops neglecta		ESP	Spain		Zufre	H	37.8815	-6.31671	396
00:00.0	MUB	102379-1	Specimen	Aegilops neglecta Req. ex Bertol.	Req. ex Bert	ES	Spain	Cartagena; Barrio de la ConcepciÃ³		Mu			1
00:00.0	REDIAM-CMA	405253	Observation	Aegilops neglecta		ESP	Spain		Villanueva del Arz	J	38.1511	-2.87873	1189
00:00.0	UNEX	10368-1	Observation	Aegilops neglecta Req. ex Bertol.	_	ESP	Spain	Cristina: Por Sra. Cristina hacia Val		Ba	38.9	-6.3	
	SIVIM	T-P10779:Aegi	Observation	Aegilops neglecta Req. ex Bertol.	Req. ex Bert	ES	Spain	Moreruela de TÃ¡bara			41.78	-5.88	0
	SIVIM	T-P15729:Aegi	Observation	Aegilops neglecta Req. ex Bertol.	Req. ex Bert	ES	Spain	bordes de la carretera Ã•lora-Carratraca, ba			36.76	-4.79	0
00:00.0	UNEX	10362-1	Observation	Aegilops neglecta Req. ex Bertol.	_	ESP	Spain	Moraleja: 29SPE93		Cc	40	-6.8	
00:00.0	HSS	2703	Specimen	Aegilops neglecta Req. ex Bertol	Req. ex Bert	ES	Spain	Guadajira, Finca La Orden		Ba	38.8722	-6.75254	
00:00.0	GDA	GDA10271-1-2	Specimen	Aegilops neglecta Req ex Bertol	Req ex Berto	ES	Spain	Granada, SÂª Nevada, CherÃ-n, ca		GR			650
	FUND. BIODIVER	78793	Unknown	Aegilops neglecta Req. ex Portol	Req. ex Port	ESP	Spain	Cogeces de Ã•scar		Va	41.1	-4.1	
	SIVIM	S-P01347:Aegi	Observation	Aegilops neglecta Req. ex Bertol.	Req. ex Bert	ES	Spain	Camino que va de Prades a Rojals			41.26	0.97	1100

	SIVIM	S-P04706:Aegi	Observation	Aegilops neglecta Req. ex Bertol.	Req. ex Bert	ES	Spain	Acampo de Baerla, sobre la Cartuja de Mir			41.53	-0.84	240
00:00.0	REDIAM-CMA	89424	Observation	Aegilops neglecta		ESP	Spain		BailÃ©n	J	38.1431	-3.80587	393
00:00.0	MGC	28803-1	Unknown	Aegilops neglecta Req.	Req.	ES	Spain	Ronda; Sierra de las Nieves. Quejig		Ma	0	0	1
	SIVIM	T-P27021:Aegi	Observation	Aegilops neglecta Req. ex Bertol.	Req. ex Bert	ES	Spain	*			41.53	-1.2	0
	SIVIM	T-P27832:Aegi	Observation	Aegilops neglecta Req. ex Bertol.	Req. ex Bert	ES	Spain	s Zuera (sw Huesca-Aragonien)			41.8	-0.83	0
	SIVIM	T-P10904:Aegi	Observation	Aegilops neglecta Req. ex Bertol.	Req. ex Bert	ES	Spain	Villanueva de la Vera			40.08	-5.46	350
00:00.0	FUND. BIODIVER	1348605	Unknown	Aegilops neglecta Req. ex Bertol	Req. ex Bert	ESP	Spain	Puebla de Don Rodrigo, Puente de		CR	38.1	-4.1	490
	SIVIM	T-P15745:Aegi	Observation	Aegilops neglecta Req. ex Bertol.	Req. ex Bert	ES	Spain	Arroyo Paredones, Sierra de Aguas			36.85	-4.79	350
00:00.0	FUND. BIODIVER	1835106	Unknown	Aegilops neglecta Req. ex Bertol	Req. ex Bert	ESP	Spain	San Lorenzo de Calatrava, umbria d		CR	38.1	-3.1	620
00:00.0	REDIAM-CMA	383784	Observation	Aegilops neglecta		ESP	Spain		Cambil	J	37.7325	-3.50666	1627
00:00.0	REDIAM-CMA	387746	Observation	Aegilops neglecta		ESP	Spain		Huelma	J	37.7126	-3.46648	1674
00:00.0	MA	711939-1	Specimen	Aegilops neglecta Req. ex Bertol.	Req. ex Bert	ES	Spain	Cabezarrubias del Puerto, Valle de		CR	38	-4	
	SIVIM	Q-P02281:Aegi	Observation	Aegilops neglecta Req. ex Bertol.	Req. ex Bert	ES	Spain	Ciutadella: Cala En Blanes			39.92	3.7	10
	SIVIM	U-P13951:Aegi	Observation	Aegilops neglecta Req. ex Bertol.	Req. ex Bert	ES	Spain	El Bonillo			38.84	-2.42	1040
00:00.0	MGC	61703-1	Unknown	Aegilops neglecta Req.	Req.	ES	Spain	Antequera; Torcal		Ma	36.96	-4.556	1200
	FUND. BIODIVER	1027216	Unknown	Aegilops neglecta Req. ex Bertol	Req. ex Bert	ESP	Spain	Marmolejo		J	38.1	-3.1	700
00:00.0	MA	680799-1	Specimen	Aegilops neglecta Reg. ex Bertol	Reg. ex Bert	ES	Spain	SerrejÃ³n 'El Pizarral'		Cc			
	FUND. BIODIVER	96904	Unknown	Aegilops neglecta Reg. ex Bertol	Reg. ex Bert	ESP	Spain	Fermoselle		Za			
00:00.0	REDIAM-CMA	364978	Observation	Aegilops neglecta		ESP	Spain		Turre	Al	37.0976	-1.91683	689
	FUND. BIODIVER	1043766	Unknown	Aegilops neglecta Req. ex Bertol	Req. ex Bert	ESP	Spain	CoÃ-n		Ma	36.1	-4.1	
	FUND. BIODIVER	1835096	Unknown	Aegilops neglecta Req. ex Bertol	Req. ex Bert	ESP	Spain	intercalacion basaltica del Alamillo		CR	38.1	-4.1	
00:00.0	FUND. BIODIVER	1835117	Unknown	Aegilops neglecta Req. ex Bertol	Req. ex Bert	ESP	Spain	Brazatortas, valle del arroyo de la C		CR	38.1	-4.1	740
	SIVIM	P-P08980:Aegi	Observation	Aegilops neglecta Req. ex Bertol.	Req. ex Bert	ES	Spain	Tall Nou; Tortosa			40.7	0.15	1140
	SIVIM	Q-P01989:Aegi	Observation	Aegilops neglecta Req. ex Bertol.	Req. ex Bert	ES	Spain	Vora el Mas de la Tancada			40.26	-0.41	1200
	SIVIM	U-P08105:Aegi	Observation	Aegilops neglecta Req. ex Bertol.	Req. ex Bert	ES	Spain	Los Cercones, LogrosÃ¡n			39.27	-5.55	0
00:00.0	REDIAM-CMA	133079	Observation	Aegilops neglecta		ESP	Spain		Santa Olalla del C	H	37.8228	-6.19183	461
00:00.0	REDIAM-CMA	173031	Observation	Aegilops neglecta		ESP	Spain		Arroyomolinos de	H	38.0383	-6.37496	899
00:00.0	SEV	98936-1	Specimen	Aegilops neglecta Req. ex Bertol.	Req. ex Bert	ES	Spain	Entre Posadas y Villaviciosa. Arroyo		Co			1
	SIVIM	Q-P02144:Aegi	Observation	Aegilops neglecta Req. ex Bertol.	Req. ex Bert	ES	Spain	Sant Joan			40.17	-0.41	1275
	FUND. BIODIVER	1093249	Unknown	Aegilops neglecta		ESP	Spain	Almaraz		Cc	39.1	-5.1	
00:00.0	FUND. BIODIVER	1946413	Unknown	Aegilops neglecta Req. ex Bertol	Req. ex Bert	ESP	Spain	Fuencaliente, valle del rio Cereceda		CR	38.1	-4.1	860
00:00.0	REDIAM-CMA	395815	Observation	Aegilops neglecta		ESP	Spain		Luque	Co	37.4975	-4.25858	945
00:00.0	REDIAM-CMA	400297	Observation	Aegilops neglecta		ESP	Spain		Zufre	H	37.8745	-6.35116	378
00:00.0	UNEX	06714-1	Observation	Aegilops neglecta Req. ex Bertol.	_	ESP	Spain	Los Santos de Maimona: Cerro San		Ba	38.5	-6.5	
	SIVIM	S-P04680:Aegi	Observation	Aegilops neglecta Req. ex Bertol.	Req. ex Bert	ES	Spain	Vilanova de la Barca, orilla izquierda del Se			41.61	0.71	0
	SIVIM	S-P05305:Aegi	Observation	Aegilops neglecta Req. ex Bertol.	Req. ex Bert	ES	Spain	SamalÃºs, al costat de la carretera, en un t			41.63	2.27	0
00:00.0	REDIAM-CMA	27044	Observation	Aegilops neglecta		ESP	Spain		La Puebla de los	Se	37.8384	-5.4243	399
	SIVIM	T-P00931:Aegi	Observation	Aegilops neglecta Req. ex Bertol.	Req. ex Bert	ES	Spain	Base de Sierra Mariola			38.73	-0.46	0
	SIVIM	T-P09146:Aegi	Observation	Aegilops neglecta Req. ex Bertol.	Req. ex Bert	ES	Spain	Portillo			41.44	-4.67	0
	ESP004	NC022339	Specimen	Aegilops neglecta REQ. ex BERTOL.		ESP	Spain	Fuente-Agria, Espiel, province of Cordoba					
00:00.0	MGC	45953-1	Unknown	Aegilops neglecta Req.	Req.	ES	Spain	Yunquera; P. N. Sierra de las Nieve		Ma	36.71	-4.965	1150

	FUND. BIODIVER	74650	Unknown	Aegilops neglecta Req. ex Bertol	Req. ex Bert	ESP	Spain	Montemayor del rÃ-o		Sa	40.1	-5.1	
00:00.0	FUND. BIODIVER	1835112	Unknown	Aegilops neglecta Req. ex Bertol	Req. ex Bert	ESP	Spain	Aldea del Rey, cerro de la Vaqueriz		CR	38.1	-3.1	750
00:00.0	MA	627624-1	Specimen	Aegilops neglecta Req. & Bertol	Req. & Berto	ES	Spain	Piedrabuena		CR	39	-4	
00:00.0	REDIAM-CMA	112202	Observation	Aegilops neglecta		ESP	Spain		Zufre	H	37.8746	-6.35084	390
	SIVIM	T-P18894:Aegi	Observation	Aegilops neglecta Req. ex Bertol.	Req. ex Bert	ES	Spain	AlcalÃ¡ de Henares, finca La Clota			40.46	-3.47	0
	SIVIM	T-P19966:Aegi	Observation	Aegilops neglecta Req. ex Bertol.	Req. ex Bert	ES	Spain	Dehesa de los Caballos (Plasencia)			39.98	-6.18	0
	SIVIM	T-P20210:Aegi	Observation	Aegilops neglecta Req. ex Bertol.	Req. ex Bert	ES	Spain	Los Barrios, prÃ³ximo de Tiradero			36.11	-5.66	100
	SIVIM	T-P15732:Aegi	Observation	Aegilops neglecta Req. ex Bertol.	Req. ex Bert	ES	Spain	zonas elevadas de la Sierra de Aguas			36.85	-4.79	0
00:00.0	REDIAM-CMA	388257	Observation	Aegilops neglecta		ESP	Spain		Torres	J	37.7454	-3.53914	1393
00:00.0	GDA	GDA25152-1-2	Specimen	Aegilops neglecta Req ex Bertol	Req ex Berto	ES	Spain	Granada, Pedro MartÃ-nez, Mencal		GR			1150
	SIVIM	U-P13963:Aegi	Observation	Aegilops neglecta Req. ex Bertol.	Req. ex Bert	ES	Spain	Robledo			38.75	-2.42	0
00:00.0	COA	41234-1	Specimen	Aegilops neglecta Req. ex Bertol.	Req. ex Bert	ES	Spain	Cabra, B		Co	37.4	-4.47	
	FUND. BIODIVER	1482315	Unknown	Aegilops neglecta Req. ex Bertol	Req. ex Bert	ESP	Spain	P.N. CabaÃ±eros		CR			
00:00.0	REDIAM-CMA	128460	Observation	Aegilops neglecta		ESP	Spain		Cazalla de la Sier	Se	37.9301	-5.71158	443
00:00.0	REDIAM-CMA	200049	Observation	Aegilops neglecta		ESP	Spain		Constantina	Se	37.8506	-5.63428	499
	SIVIM	T-P18887:Aegi	Observation	Aegilops neglecta Req. ex Bertol.	Req. ex Bert	ES	Spain	AlcalÃ¡ de Henares, finca La Clota			40.46	-3.47	0
	SIVIM	T-P19657:Aegi	Observation	Aegilops neglecta Req. ex Bertol.	Req. ex Bert	ES	Spain	Finca 'La Torre' (Posadas)			37.74	-5.15	345
	SIVIM	T-P20030:Aegi	Observation	Aegilops neglecta Req. ex Bertol.	Req. ex Bert	ES	Spain	El Arco (CaÃ±averal)			39.72	-6.43	0
00:00.0	UNEX	25102-1	Observation	Aegilops neglecta Req. ex Bertol.	_	ESP	Spain	Cazorla: Nava de San Pablo. 1680 r		J	37.9	-2.9	
00:00.0	REDIAM-CMA	361674	Observation	Aegilops neglecta		ESP	Spain		Villanueva de CÃ	Co	38.2359	-4.60798	700
	SIVIM	P-P04337:Aegi	Observation	Aegilops neglecta Req. ex Bertol.	Req. ex Bert	ES	Spain	entre Nerja y Frigiliana			36.77	-3.89	150
	SIVIM	Q-P02014:Aegi	Observation	Aegilops neglecta Req. ex Bertol.	Req. ex Bert	ES	Spain	Pla de Baix			40.25	-0.29	1125
	CZE122	01C2108009	Specimen	Aegilops neglecta subsp. recta (ZHUK.) HAMM		ESP	Spain	nahe der Strasse zwischen Logrosan und Guadalupe, Caceres					
	FUND. BIODIVER	1946421	Unknown	Aegilops neglecta Req. ex Bertol	Req. ex Bert	ESP	Spain	Real Valle de Alcudia		CR			
00:00.0	GDAC	GDAC24837-1	Specimen	Aegilops neglecta Req. ex Bertol	Req. ex Bert	ES	Spain	Zamora, CaÃ±izal.		ZA			0
	IPK	639118	Living	Aegilops neglecta Req. ex Bertol. subsp. recta		ESP	Spain						
	FUND. BIODIVER	52931	Unknown	Aegilops neglecta Req. ex Bertol	Req. ex Bert	ESP	Spain	Arapiles		Sa	40.1	-5.1	
	SIVIM	S-P00076:Aegi	Observation	Aegilops neglecta Req. ex Bertol.	Req. ex Bert	ES	Spain	*			41.45	1.92	0
	SIVIM	S-P02872:Aegi	Observation	Aegilops neglecta Req. ex Bertol.	Req. ex Bert	ES	Spain	massif du Tibidabo: entre la gare infÃ©rieu			41.36	2.04	260
	SIVIM	S-P04716:Aegi	Observation	Aegilops neglecta Req. ex Bertol.	Req. ex Bert	ES	Spain	entre la Cartuja de Miraflores y Torrecilla de			41.53	-0.84	320
	SIVIM	S-P06246:Aegi	Observation	Aegilops neglecta Req. ex Bertol.	Req. ex Bert	ES	Spain	sierra CorbalÃ¡n			40.36	-0.99	0
00:00.0	REDIAM-CMA	36569	Observation	Aegilops neglecta		ESP	Spain		Posadas	Co	37.9167	-5.10924	457
00:00.0	SEV	61119-1	Specimen	Aegilops neglecta Req. ex Bertol.	Req. ex Bert	ES	Spain	Algodonales, Sierra de LÃ-jar		Ca			500
	SIVIM	T-P06096:Aegi	Observation	Aegilops neglecta Req. ex Bertol.	Req. ex Bert	ES	Spain	altiplanicie de Venta de Yesos			37.04	-2.32	0
	SIVIM	T-P09180:Aegi	Observation	Aegilops neglecta Req. ex Bertol.	Req. ex Bert	ES	Spain	Hornillo de Cerrato			41.9	-4.32	0
	SIVIM	T-P00935:Aegi	Observation	Aegilops neglecta Req. ex Bertol.	Req. ex Bert	ES	Spain	Teulada			38.72	0	200
	SIVIM	T-P09148:Aegi	Observation	Aegilops neglecta Req. ex Bertol.	Req. ex Bert	ES	Spain	Bercero			41.53	-5.15	0
	FUND. BIODIVER	1093243	Unknown	Aegilops neglecta		ESP	Spain	Coria		Cc	39.1	-6.1	
00:00.0	FUND. BIODIVER	1946429	Unknown	Aegilops neglecta Req. ex Bertol	Req. ex Bert	ESP	Spain	Aldea del Rey, cerro de la Higuera		CR	38.1	-3.1	790
	DEU146	AE 589	Specimen	Aegilops neglecta Req. ex Bertol. subsp. recta		ESP	Spain	Finca La Cigueela, stlich Aracena, Huelva					
	FUND. BIODIVER	1043770	Unknown	Aegilops neglecta Req. ex Bertol	Req. ex Bert	ESP	Spain	Ronda, Sierra de las Nieves		Ma	36.1	-4.1	

00:00.0	FUND. BIODIVER	1835100	Unknown	Aegilops neglecta Req. ex Bertol	Req. ex Bert	ESP	Spain	Mestanza, valle del rio Robledillo		CR	38.1	-3.1	440
00:00.0	REDIAM-CMA	410323	Observation	Aegilops neglecta		ESP	Spain		GÃ©nave	J	38.4534	-2.73925	768
00:00.0	REDIAM-CMA	386964	Observation	Aegilops neglecta		ESP	Spain		Aroche	H	37.8779	-6.97677	315
00:00.0	REDIAM-CMA	241776	Observation	Aegilops neglecta		ESP	Spain		El Real de la Jara	Se	37.9218	-6.1311	496
00:00.0	REDIAM-CMA	282312	Observation	Aegilops neglecta		ESP	Spain		Hinojosa del Duq	Co	38.4053	-5.16516	600
	SIVIM	S-P00311:Aegi	Observation	Aegilops neglecta Req. ex Bertol.	Req. ex Bert	ES	Spain	*			41.45	2.16	0
	SIVIM	S-P04949:Aegi	Observation	Aegilops neglecta Req. ex Bertol.	Req. ex Bert	ES	Spain	Osera, barranco del Hospital			41.52	-0.6	240
	SIVIM	S-P06270:Aegi	Observation	Aegilops neglecta Req. ex Bertol.	Req. ex Bert	ES	Spain	de la comarca de Linares de Mora			40.26	-0.64	0
	SIVIM	Q-P02164:Aegi	Observation	Aegilops neglecta Req. ex Bertol.	Req. ex Bert	ES	Spain	Sant Joan			40.17	-0.41	1275
	SIVIM	T-P27052:Aegi	Observation	Aegilops neglecta Req. ex Bertol.	Req. ex Bert	ES	Spain	*			41.52	-0.6	270
	SIVIM	T-P28618:Aegi	Observation	Aegilops neglecta Req. ex Bertol.	Req. ex Bert	ES	Spain	Dehesa de Valdeobispo, Almaraz			39.8	-5.68	350
	SIVIM	T-P31983:Aegi	Observation	Aegilops neglecta Req. ex Bertol.	Req. ex Bert	ES	Spain	AÃ±over de Tajo, cerca de la carretera de			39.92	-3.81	0
00:00.0	SEV	108292-1	Specimen	Aegilops neglecta Req. ex Bertol.	Req. ex Bert	ES	Spain	Puerto de Santa MarÃ-a		Ca			1
00:00.0	COA	41176-1	Specimen	Aegilops neglecta Req. ex Bertol.	Req. ex Bert	ES	Spain	Km 15 al sur de CardeÃ±a		Co	38.12	-4.37	
00:00.0	UNEX	10360-1	Observation	Aegilops neglecta Req. ex Bertol.	_	ESP	Spain	LogrosÃ¡n: Entre LogrosÃ¡n y Bezo		Cc	39.4	-5.6	
00:00.0	S	S05-10783	Specimen	Aegilops neglecta Req. ex Bertol.	Req. ex Bert	Spain	Spain	Habitat in Europa australis lectam ad ? misit B. M. Dnus Petr. Loefling, Botanicus					
00:00.0	COA	41173-1	Specimen	Aegilops neglecta Req. ex Bertol.	Req. ex Bert	ES	Spain	Km 10 de Espiel a Alcaracejos		Co	38.2	-5.06	
00:00.0	FUND. BIODIVER	1835108	Unknown	Aegilops neglecta Req. ex Bertol	Req. ex Bert	ESP	Spain	sierra de Alhambra		CR	38.1	-2.1	
00:00.0	COFC	25338-1	Specimen	Aegilops neglecta Req. ex Bertol.	Req. ex Bert	ES	Spain	Cazalla de la Sierra; estaciÃƒÂ³n C		Se			1
	SIVIM	R-P02062:Aegi	Observation	Aegilops neglecta Req. ex Bertol.	Req. ex Bert	ES	Spain	Batea, cap a Nonasp, als lÃ-mits fronterers			41.06	0.26	220
	SIVIM	U-P13955:Aegi	Observation	Aegilops neglecta Req. ex Bertol.	Req. ex Bert	ES	Spain	Viveros			38.75	-2.65	1020
00:00.0	REDIAM-CMA	192713	Observation	Aegilops neglecta		ESP	Spain		Tabernas	Al	37.0854	-2.33726	483
	SIVIM	T-P18897:Aegi	Observation	Aegilops neglecta Req. ex Bertol.	Req. ex Bert	ES	Spain	AlcalÃ¡ de Henares, finca La Clota			40.46	-3.47	0
	SIVIM	T-P19969:Aegi	Observation	Aegilops neglecta Req. ex Bertol.	Req. ex Bert	ES	Spain	Finca de Araya (Arroyo de la Luz)			39.45	-6.67	0
	SIVIM	T-P20222:Aegi	Observation	Aegilops neglecta Req. ex Bertol.	Req. ex Bert	ES	Spain	MengÃ-bar			37.94	-3.91	300
	FUND. BIODIVER	1065854	Unknown	Aegilops neglecta Req. & Bertol	Req. & Berto	ESP	Spain	Sierra de Aracena		H	37.1	-6.1	
00:00.0	FUND. BIODIVER	1835107	Unknown	Aegilops neglecta Req. ex Bertol	Req. ex Bert	ESP	Spain	Solana del Pino, Alhorin		CR	38.1	-3.1	660
00:00.0	MGC	13225-1	Unknown	Aegilops neglecta Req.	Req.	ES	Spain	Tabernas; Venta de los Yesos		Al	37.0873	-2.29373	1
	SIVIM	Q-P02282:Aegi	Observation	Aegilops neglecta Req. ex Bertol.	Req. ex Bert	ES	Spain	Ciutadella: Cala En Blanes			39.92	3.7	10
	SIVIM	U-P13953:Aegi	Observation	Aegilops neglecta Req. ex Bertol.	Req. ex Bert	ES	Spain	El Bonillo			38.93	-2.53	1040
	SIVIM	T-P27016:Aegi	Observation	Aegilops neglecta Req. ex Bertol.	Req. ex Bert	ES	Spain	*			41.42	-0.24	340
	SIVIM	T-P29409:Aegi	Observation	Aegilops neglecta Req. ex Bertol.	Req. ex Bert	ES	Spain	Cerros de Enix, AlmerÃ-a			36.86	-2.66	0
	FUND. BIODIVER	57501	Unknown	Aegilops neglecta Req. ex Bertol	Req. ex Bert	ESP	Spain	Matilla de los CaÃ±os del rÃ-o		Sa	40.1	-5.1	
	FUND. BIODIVER	1043765	Unknown	Aegilops neglecta Req. ex Bertol	Req. ex Bert	ESP	Spain	Alora		Ma	36.1	-4.1	
00:00.0	FUND. BIODIVER	1835095	Unknown	Aegilops neglecta Req. ex Bertol	Req. ex Bert	ESP	Spain	Fuencaliente, sierra del Cotillo		CR	38.1	-4.1	820
00:00.0	FUND. BIODIVER	1835116	Unknown	Aegilops neglecta Req. ex Bertol	Req. ex Bert	ESP	Spain	Alcoba, Cabaneros, Guarreras		CR	39.1	-4.1	
	FUND. BIODIVER	1093247	Unknown	Aegilops neglecta		ESP	Spain	Cerro Calero, Campillo de Deleitosa		Cc	39.1	-5.1	
00:00.0	FUND. BIODIVER	1946411	Unknown	Aegilops neglecta Req. ex Bertol	Req. ex Bert	ESP	Spain	Fuencaliente, sierra del Cotillo		CR	38.1	-4.1	820
00:00.0	FUND. BIODIVER	1946433	Unknown	Aegilops neglecta Req. ex Bertol	Req. ex Bert	ESP	Spain	Brazatortas, valle del arroyo de la C		CR	38.1	-4.1	740
00:00.0	REDIAM-CMA	386792	Observation	Aegilops neglecta		ESP	Spain		Cambil	J	37.7288	-3.49105	1581
00:00.0	REDIAM-CMA	394623	Observation	Aegilops neglecta		ESP	Spain		CardeÃ±a	Co	38.2463	-4.28955	740

00:00.0	REDIAM-CMA	22028	Observation	Aegilops neglecta		ESP	Spain		Mijas	Ma	36.5965	-4.70744	208
00:00.0	REDIAM-CMA	399793	Observation	Aegilops neglecta		ESP	Spain		Hornachuelos	Co	37.9957	-5.45272	623
00:00.0	REDIAM-CMA	410665	Observation	Aegilops neglecta		ESP	Spain		Villarrodrigo	J	38.4798	-2.64301	900
	FUND. BIODIVER	1043769	Unknown	Aegilops neglecta Req. ex Bertol	Req. ex Bert	ESP	Spain	Mijas		Ma	36.1	-4.1	
00:00.0	FUND. BIODIVER	1835099	Unknown	Aegilops neglecta Req. ex Bertol	Req. ex Bert	ESP	Spain	Piedrabuena, chopera proxima a la		CR	38.1	-4.1	700
	SIVIM	Q-P02125:Aegi	Observation	Aegilops neglecta Req. ex Bertol.	Req. ex Bert	ES	Spain	Pla de Baix			40.25	-0.29	1125
	SIVIM	S-P05049:Aegi	Observation	Aegilops neglecta Req. ex Bertol.	Req. ex Bert	ES	Spain	orilla del GÃ¡llego, entre Zaragoza y Zuera			41.71	-0.83	240
	SIVIM	S-P06516:Aegi	Observation	Aegilops neglecta Req. ex Bertol.	Req. ex Bert	ES	Spain	entre Son Coll et Banyalbufar			39.65	2.41	200
00:00.0	REDIAM-CMA	391936	Observation	Aegilops neglecta		ESP	Spain		Chiclana de Segu	J	38.375	-2.96811	779
00:00.0	COFC	50169-1	Specimen	Aegilops neglecta Req. ex Bertol.	Req. ex Bert	ES	Spain	Valle del Guadiato; puente de la Ca		Co			1
	SIVIM	T-P16713:Aegi	Observation	Aegilops neglecta Req. ex Bertol.	Req. ex Bert	ES	Spain	La Gabia			37.04	-3.67	0
	SIVIM	Q-P02157:Aegi	Observation	Aegilops neglecta Req. ex Bertol.	Req. ex Bert	ES	Spain	Vistabella, marge de la carretera			40.25	-0.29	1250
00:00.0	SALA	71828-1	Specimen	Aegilops neglecta Req. ex Bertol	Req. ex Bert	ES	Spain	_; SerrejÃ³n		Cc	39.82	-5.8	
00:00.0	UNEX	10363-1	Observation	Aegilops neglecta Req. ex Bertol.	_	ESP	Spain	Sancti Spiritus: Embalse del ZÃºjar.		Ba	38.8	-5.3	
	FUND. BIODIVER	1465440	Unknown	Aegilops neglecta Req. ex Bertol	Req. ex Bert	ESP	Spain	JÃ¡novas		Hu	42.1	0.9	670
00:00.0	REDIAM-CMA	153880	Observation	Aegilops neglecta		ESP	Spain		CardeÃ±a	Co	38.1958	-4.26616	703
00:00.0	REDIAM-CMA	170172	Observation	Aegilops neglecta		ESP	Spain		Aracena	H	37.9518	-6.54547	400
00:00.0	COFC	41266-1	Specimen	Aegilops neglecta Req. ex Bertol.	Req. ex Bert	ES	Spain	Cabra; cerro la Cumbre		Co	37	-4	766
00:00.0	SEV	9725-1	Specimen	Aegilops neglecta Req. ex Bertol.	Req. ex Bert	ES	Spain	Ronda. Sierra de las Nieves		Ma			1300
00:00.0	MA	597119-1	Specimen	Aegilops neglecta Req. ex Bertol.	Req. ex Bert	ES	Spain	Brazatortas, proximidades del arroy		CR	38	-4	
00:00.0	FUND. BIODIVER	1946430	Unknown	Aegilops neglecta Req. ex Bertol	Req. ex Bert	ESP	Spain	Abenojar, entre Casa de la Solana y		CR	38.1	-4.1	680
	FUND. BIODIVER	1712962	Unknown	Aegilops neglecta Req. ex Bertol	Req. ex Bert	ESP	Spain	Menorca, Es Berrecks de Sta. Anna		PM	39.97	4	
	FUND. BIODIVER	1810918	Unknown	Aegilops neglecta		ESP	Spain	Monterrubio de la Armuna, arroyo d		Sa	40.1	-5.1	800
00:00.0	ABH	39237-1	Specimen	Aegilops neglecta Req ex Bertol.	Req ex Berto	ES	Spain	Alicante; Cabo de las Huertas, Lom		A	38.35	-0.43	
00:00.0	REDIAM-CMA	14729	Observation	Aegilops neglecta		ESP	Spain		Aroche	H	37.9174	-7.05408	258
00:00.0	REDIAM-CMA	398329	Observation	Aegilops neglecta		ESP	Spain		Hornachuelos	Co	37.9562	-5.34326	473
00:00.0	REDIAM-CMA	404587	Observation	Aegilops neglecta		ESP	Spain		AlmadÃ©n de la F	Se	37.8524	-6.00544	278
00:00.0	REDIAM-CMA	414511	Observation	Aegilops neglecta		ESP	Spain		Baza	Gr	37.2364	-2.69537	1947
	SIVIM	S-P00322:Aegi	Observation	Aegilops neglecta Req. ex Bertol.	Req. ex Bert	ES	Spain	*			41.36	2.04	130
	SIVIM	S-P02914:Aegi	Observation	Aegilops neglecta Req. ex Bertol.	Req. ex Bert	ES	Spain	Serra Superior del VallÃ¨s: Sabarrugues, a			41.63	2.15	0
	SIVIM	S-P04957:Aegi	Observation	Aegilops neglecta Req. ex Bertol.	Req. ex Bert	ES	Spain	Fuentes en la carretera de Belchite			41.43	-0.72	250
	SIVIM	S-P06277:Aegi	Observation	Aegilops neglecta Req. ex Bertol.	Req. ex Bert	ES	Spain	de la comarca de Linares-Valdelinares			40.26	-0.64	0
	SIVIM	Q-P01997:Aegi	Observation	Aegilops neglecta Req. ex Bertol.	Req. ex Bert	ES	Spain	Prop el Mas del Collet			40.26	-0.41	1225
00:00.0	FUND. BIODIVER	1946428	Unknown	Aegilops neglecta Req. ex Bertol	Req. ex Bert	ESP	Spain	Aldea del Rey, cerro de la Vaqueriz		CR	38.1	-3.1	750
00:00.0	REDIAM-CMA	404249	Observation	Aegilops neglecta		ESP	Spain		AlmadÃ©n de la F	Se	37.8996	-6.04941	500
00:00.0	REDIAM-CMA	409983	Observation	Aegilops neglecta		ESP	Spain		Villarrodrigo	J	38.4674	-2.67598	893
00:00.0	REDIAM-CMA	414416	Observation	Aegilops neglecta		ESP	Spain		Espiel	Co	38.0991	-4.99322	497
	SIVIM	S-P00157:Aegi	Observation	Aegilops neglecta Req. ex Bertol.	Req. ex Bert	ES	Spain	*			41.36	1.92	0
	SIVIM	S-P02911:Aegi	Observation	Aegilops neglecta Req. ex Bertol.	Req. ex Bert	ES	Spain	massif du St. LlorenÃ§: Riera de les Arene			41.54	1.92	0
	SIVIM	S-P04933:Aegi	Observation	Aegilops neglecta Req. ex Bertol.	Req. ex Bert	ES	Spain	llanura de Huesca			42.06	-0.46	0
	SIVIM	S-P06268:Aegi	Observation	Aegilops neglecta Req. ex Bertol.	Req. ex Bert	ES	Spain	del SE de Linares de Mora			40.26	-0.64	0

00:00.0	REDIAM-CMA	220043	Observation	Aegilops neglecta		ESP	Spain		Baza	Gr	37.2387	-2.72858	1911
00:00.0	REDIAM-CMA	231462	Observation	Aegilops neglecta		ESP	Spain		AlmadÃ©n de la P	Se	37.888	-6.06136	501
	SIVIM	T-P17882:Aegi	Observation	Aegilops neglecta Req. ex Bertol.	Req. ex Bert	ES	Spain	margen de la carretera de Totana a Mazarr			37.67	-2.54	0
	SIVIM	T-P19974:Aegi	Observation	Aegilops neglecta Req. ex Bertol.	Req. ex Bert	ES	Spain	Dehesa de Valdeobispo (Almaraz)			39.8	-5.68	350
	CZE122	01C2108006	Specimen	Aegilops neglecta subsp. recta (ZHUK.) HAMM		ESP	Spain	bei Molinillo nahe Bejar, Salamanca					
00:00.0	SEV	106434-1	Specimen	Aegilops neglecta Req. ex Bertol.	Req. ex Bert	ES	Spain	Coripe, Sierra		Se			1
	FUND. BIODIVER	1093248	Unknown	Aegilops neglecta		ESP	Spain	Almaraz		Cc	39.1	-5.1	
	FUND. BIODIVER	1946412	Unknown	Aegilops neglecta Req. ex Bertol	Req. ex Bert	ESP	Spain	intercalacion basaltica del Alamillo		CR	38.1	-4.1	
	SIVIM	S-P06689:Aegi	Observation	Aegilops neglecta Req. ex Bertol.	Req. ex Bert	ES	Spain	environs de Valldemossa			39.65	2.53	0
00:00.0	COFC	21303-1	Specimen	Aegilops neglecta Req. ex Bertol.	Req. ex Bert	ES	Spain	Sierra de Hornachuelos; 'Mosqueros		Co			1
00:00.0	FUND. BIODIVER	1712651	Unknown	Aegilops neglecta Req ex. Bertol	Req ex. Bert	ESP	Spain	Menorca, Ciutadella, Sta. Anna, Es		PM	39.1	3.1	
	FUND. BIODIVER	86871	Unknown	Aegilops neglecta		ESP	Spain	Arguijo		So	41.1	-2.1	
	SIVIM	Q-P01996:Aegi	Observation	Aegilops neglecta Req. ex Bertol.	Req. ex Bert	ES	Spain	Pla de Baix			40.25	-0.29	1125
	SIVIM	U-P08108:Aegi	Observation	Aegilops neglecta Req. ex Bertol.	Req. ex Bert	ES	Spain	Los Cercones, LogrosÃ¡n			39.27	-5.55	0
	SIVIM	T-P10903:Aegi	Observation	Aegilops neglecta Req. ex Bertol.	Req. ex Bert	ES	Spain	Valverde de la Vega, 'La Vega'			40.08	-5.46	300
	SIVIM	T-P15734:Aegi	Observation	Aegilops neglecta Req. ex Bertol.	Req. ex Bert	ES	Spain	Arroyo de Paredones, Sierra de Aguas			36.85	-4.79	0
	FUND. BIODIVER	116426	Unknown	Aegilops neglecta Req. ex Bertol	Req. ex Bert	ESP	Spain	Almenara de Adaja		Va	41.1	-4.1	
00:00.0	REDIAM-CMA	53068	Observation	Aegilops neglecta		ESP	Spain		Aroche	H	37.8926	-7.00186	300
00:00.0	REDIAM-CMA	205257	Observation	Aegilops neglecta		ESP	Spain		Hornachuelos	Co	37.9071	-5.29759	375
00:00.0	REDIAM-CMA	232277	Observation	Aegilops neglecta		ESP	Spain		AlmadÃ©n de la P	Se	37.8838	-5.99813	417
00:00.0	COFC	52515-1	Specimen	Aegilops neglecta Req. ex Bertol.	Req. ex Bert	ES	Spain	Moraleja; _		Cc	0	0	1
	SIVIM	T-P17937:Aegi	Observation	Aegilops neglecta Req. ex Bertol.	Req. ex Bert	ES	Spain	RÃ-o Aguas			37.04	-2.1	0
	SIVIM	T-P19975:Aegi	Observation	Aegilops neglecta Req. ex Bertol.	Req. ex Bert	ES	Spain	Cerro Calero (Campillo de Deleitosa)			39.62	-5.67	0
	FUND. BIODIVER	1120225	Unknown	Aegilops neglecta Req. ex Bertol	Req. ex Bert	ESP	Spain	Pinilla de Toro, El Ama		Za	41.1	-5.1	
00:00.0	REDIAM-CMA	37457	Observation	Aegilops neglecta		ESP	Spain		Cambil	J	37.7289	-3.49125	1581
00:00.0	REDIAM-CMA	46745	Observation	Aegilops neglecta		ESP	Spain		Huelma	J	37.7081	-3.46366	1600
00:00.0	BC	836688	Specimen	Aegilops neglecta Req. ex Bertol.	Req. ex Bert	ES	Spain	Andujar; AndÃºjar, Base de la Sierra		J	38.379	-4.174	800
	SIVIM	T-P00940:Aegi	Observation	Aegilops neglecta Req. ex Bertol.	Req. ex Bert	ES	Spain	Agost; proximidades del ferrocarril			38.37	-0.71	240
00:00.0	SEV	9956-1	Specimen	Aegilops neglecta Req. ex Bertol.	Req. ex Bert	ES	Spain	Cazalla de la Sierra, entre la estaciÃ		Se			570
	ESP004	NC043468	Specimen	Aegilops neglecta REQ. ex BERTOL.		ESP	Spain	isla Mallorca, Soller, province of Baleares			39.75	2.7	41
	ESP004	NC022349	Specimen	Aegilops neglecta REQ. ex BERTOL.		ESP	Spain	Medina Azahara, Cordoba, province of Cor			37.8833	-4.86667	219
00:00.0	HSS	12653	Specimen	Aegilops neglecta Req. ex Bertol	Req. ex Bert	ES	Spain	CÃ¡ceres, proximidades de la ciuda		Cc	39.4952	-6.38377	
00:00.0	MGC	2438-1	Unknown	Aegilops neglecta Req.	Req.	ES	Spain	MÃ¡laga; El Palo. Arroyo de los Pilo		Ma	36.72	-4.4	1
	SIVIM	T-P15731:Aegi	Observation	Aegilops neglecta Req. ex Bertol.	Req. ex Bert	ES	Spain	zonas elevadas de la Sierra de Aguas			36.85	-4.79	0
00:00.0	REDIAM-CMA	38149	Observation	Aegilops neglecta		ESP	Spain		Cambil	J	37.729	-3.48975	1594
	SIVIM	T-P00941:Aegi	Observation	Aegilops neglecta Req. ex Bertol.	Req. ex Bert	ES	Spain	Alicante; proximidades de los Doce Puente			38.28	-0.48	30
	SIVIM	T-P06092:Aegi	Observation	Aegilops neglecta Req. ex Bertol.	Req. ex Bert	ES	Spain	de la comarca del RÃ-o Aguas			37.13	-1.98	160
	FUND. BIODIVER	1451608	Unknown	Aegilops neglecta Req. ex Bertol	Req. ex Bert	ESP	Spain	Villaluenga del Rosario, Bodega Las		Ca	36.1	-5.1	700
	FUND. BIODIVER	96903	Unknown	Aegilops neglecta Req. ex Bertol	Req. ex Bert	ESP	Spain	Pereruela		Za	41.1	-5.1	
00:00.0	SEV	49880-1	Specimen	Aegilops neglecta Req. ex Bertol.	Req. ex Bert	ES	Spain	Entre La UmbrÃ-a y Puerto Moral		H			1
00:00.0	REDIAM-CMA	159582	Observation	Aegilops neglecta		ESP	Spain		Zufre	H	37.9366	-6.43487	392

00:00.0	MGC	59843-1	Unknown	Aegilops neglecta Req.	Req.	ES	Spain	Arenas del Rey; P. N. de las Sierras		Gr	36.913	-3.87	1050
00:00.0	REDIAM-CMA	251178	Observation	Aegilops neglecta		ESP	Spain		AlÃ¡jar	H	37.8285	-6.63281	454
00:00.0	COFC	39483-1	Specimen	Aegilops neglecta Req. ex Bertol.	Req. ex Bert	ES	Spain	cerro de 'La Cumbre'; Mojonera		Co	37	-4	766
	SIVIM	T-P27084:Aegi	Observation	Aegilops neglecta Req. ex Bertol.	Req. ex Bert	ES	Spain	*			41.71	-1.07	300
	SIVIM	T-P28911:Aegi	Observation	Aegilops neglecta Req. ex Bertol.	Req. ex Bert	ES	Spain	Presa de ValdecaÃ±as			39.71	-5.68	330
	SIVIM	T-P31987:Aegi	Observation	Aegilops neglecta Req. ex Bertol.	Req. ex Bert	ES	Spain	de AlcalÃ¡ de Henares, finca La Clota , Pro			40.46	-3.47	0
	SIVIM	U-P06656:Aegi	Observation	Aegilops neglecta Req. ex Bertol.	Req. ex Bert	ES	Spain	Barreiras Brancas			37.13	-8.09	2500
	FUND. BIODIVER	1027217	Unknown	Aegilops neglecta Req. ex Bertol	Req. ex Bert	ESP	Spain	Marmolejo		J	38.1	-3.1	600
00:00.0	SEV	34909-1	Specimen	Aegilops neglecta Req. ex Bertol.	Req. ex Bert	ES	Spain	CardeÃ±a, finca de Torrubia		Co			1
	FUND. BIODIVER	1043767	Unknown	Aegilops neglecta Req. ex Bertol	Req. ex Bert	ESP	Spain	Estepona		Ma	36.1	-4.1	
00:00.0	FUND. BIODIVER	1835097	Unknown	Aegilops neglecta Req. ex Bertol	Req. ex Bert	ESP	Spain	Fuencaliente, valle del rio Cereceda		CR	38.1	-4.1	860
	FUND. BIODIVER	1869150	Unknown	Aegilops neglecta Req. ex Bertol	Req. ex Bert	ESP	Spain	Ciudad Real, La Atalaya		CR	38.1	-3.1	670
00:00.0	REDIAM-CMA	312053	Observation	Aegilops neglecta		ESP	Spain		GaucÃn	Ma	36.48	-5.3365	198
00:00.0	REDIAM-CMA	365042	Observation	Aegilops neglecta		ESP	Spain		Turre	Al	37.089	-1.90968	719
00:00.0	COFC	46878-1	Specimen	Aegilops neglecta Req. ex Bertol.	Req. ex Bert	ES	Spain	casco urbano; Avda del Aeropuerto		Co			1
	FUND. BIODIVER	1027214	Unknown	Aegilops neglecta Req. ex Bertol	Req. ex Bert	ESP	Spain	Marmolejo		J	38.1	-4.1	500
	SIVIM	P-P02535:Aegi	Observation	Aegilops neglecta Req. ex Bertol.	Req. ex Bert	ES	Spain	Bell-lloc del Pla, bajo la carretera de Atzen			40.16	0.06	0
	SIVIM	P-P08985:Aegi	Observation	Aegilops neglecta Req. ex Bertol.	Req. ex Bert	ES	Spain	Les Eres; Horta de Sant Joan			40.79	0.27	0
	SIVIM	Q-P01994:Aegi	Observation	Aegilops neglecta Req. ex Bertol.	Req. ex Bert	ES	Spain	CamÃ de Vistabella al Mas del Collet			40.26	-0.41	1250
	SIVIM	U-P08106:Aegi	Observation	Aegilops neglecta Req. ex Bertol.	Req. ex Bert	ES	Spain	Los Cercones, LogrosÃ¡n			39.27	-5.55	0
	SIVIM	U-P10309:Aegi	Observation	Aegilops neglecta Req. ex Bertol.	Req. ex Bert	ES	Spain	entre Pueblo de Alfambra y el de Cuevas L			40.36	-1.11	0
00:00.0	REDIAM-CMA	384202	Observation	Aegilops neglecta		ESP	Spain		Torres	J	37.7376	-3.50342	1624
00:00.0	REDIAM-CMA	386876	Observation	Aegilops neglecta		ESP	Spain		Cambil	J	37.7289	-3.48992	1594
00:00.0	REDIAM-CMA	381444	Observation	Aegilops neglecta		ESP	Spain		BailÃ©n	J	38.1035	-3.72688	304
	SIVIM	Q-P02146:Aegi	Observation	Aegilops neglecta Req. ex Bertol.	Req. ex Bert	ES	Spain	CamÃ damunt la Masia de ManÃ§anars			40.26	-0.41	1300
00:00.0	MGC	66810-1	Unknown	Aegilops neglecta Req.	Req.	ES	Spain	Llanos de LÃbar		Ma	36.667	-5.333	1
	SIVIM	Q-P01774:Aegi	Observation	Aegilops neglecta Req. ex Bertol.	Req. ex Bert	ES	Spain	Alicante, hacia la Talaia			38.28	-0.59	0
	SIVIM	U-P08103:Aegi	Observation	Aegilops neglecta Req. ex Bertol.	Req. ex Bert	ES	Spain	EstaciÃ³n Ferrocarril, LogrosÃ¡n			39.27	-5.55	0
00:00.0	MA	766617-1	Specimen	Aegilops neglecta Req. ex Bertol.	Req. ex Bert	ES	Spain	Magacela: El Berrocal		Ba	38	-5	
	FUND. BIODIVER	1835103	Unknown	Aegilops neglecta Req. ex Bertol	Req. ex Bert	ESP	Spain	Provincia de Ciudad Real		CR			
00:00.0	REDIAM-CMA	386224	Observation	Aegilops neglecta		ESP	Spain		Mijas	Ma	36.53	-4.70788	100
00:00.0	REDIAM-CMA	387384	Observation	Aegilops neglecta		ESP	Spain		Cala	H	37.9389	-6.27786	600
00:00.0	REDIAM-CMA	388808	Observation	Aegilops neglecta		ESP	Spain		Torres	J	37.7455	-3.55106	1291
	SIVIM	Q-P02274:Aegi	Observation	Aegilops neglecta Req. ex Bertol.	Req. ex Bert	ES	Spain	Ciutadella: trois-cents mÃ¨tres Ã l'Est du p			39.83	3.81	15
	SIVIM	Q-P07483:Aegi	Observation	Aegilops neglecta Req. ex Bertol.	Req. ex Bert	ES	Spain	Montalbo			39.83	-2.76	0
00:00.0	FUND. BIODIVER	1465439	Unknown	Aegilops neglecta Req. ex Bertol	Req. ex Bert	ESP	Spain	Villanueva de Sigena, RÃo Alcanad		Hu	41.1	0.9	200
	FUND. BIODIVER	47602	Unknown	Aegilops neglecta Req. ex Bertol	Req. ex Bert	ESP	Spain	CastronuÃ±o, Valdecierva, Pico Re		Va	41.1	-5.1	
	FUND. BIODIVER	54858	Unknown	Aegilops neglecta Req. ex Bertol	Req. ex Bert	ESP	Spain	BÃ³veda del rÃo Almar		Sa	40.1	-4.1	
00:00.0	REDIAM-CMA	147145	Observation	Aegilops neglecta		ESP	Spain		Santa Olalla del C	H	37.898	-6.21474	500
00:00.0	REDIAM-CMA	153659	Observation	Aegilops neglecta		ESP	Spain		Siles	J	38.318	-2.51618	1300
00:00.0	UNEX	05896-1	Observation	Aegilops neglecta Req. ex Bertol.	_	ESP	Spain	Zarza la Mayor: Zarza la Mayor. 29		Cc	39.8	-6.9	

00:00.0	REDIAM-CMA	43033	Observation	Aegilops neglecta		ESP	Spain		Hornachuelos	Co	37.867	-5.20328	209
	SIVIM	T-P00897:Aegi	Observation	Aegilops neglecta Req. ex Bertol.	Req. ex Bert	ES	Spain	Sierra de Mariola, en la base			38.73	-0.46	0
	SIVIM	T-P02226:Aegi	Observation	Aegilops neglecta Req. ex Bertol.	Req. ex Bert	ES	Spain	Escombreras (cercanÃ-as del poblado)			37.56	-0.96	30
	FUND. BIODIVER	86017	Unknown	Aegilops neglecta		ESP	Spain	Santa MarÃ-a de las Hoyas, sierra c		So	41.1	-2.1	
00:00.0	REDIAM-CMA	98012	Observation	Aegilops neglecta		ESP	Spain		AndÃºjar	J	38.0751	-3.9698	402
00:00.0	SALA	47669-1	Specimen	Aegilops neglecta Req. ex Bertol	Req. ex Bert	ES	Spain	_; Guijuelo		Sa			
00:00.0	SALA	122460-1	Specimen	Aegilops neglecta Req.	Req.	ES	Spain	Pajares de la Laguna, camino a Arc		Sa			
	FUND. BIODIVER	1093234	Unknown	Aegilops neglecta		ESP	Spain	Finca de Valdelasyeguas, Aliseda		Cc	39.1	-6.1	
	FUND. BIODIVER	1093256	Unknown	Aegilops neglecta		ESP	Spain	El Arco, CaÃ±averal		Cc	39.1	-6.1	
	FUND. BIODIVER	1946420	Unknown	Aegilops neglecta Req. ex Bertol	Req. ex Bert	ESP	Spain	Provincia de Ciudad Real		CR			
00:00.0	MGC	43198-1	Unknown	Aegilops neglecta Req.	Req.	ES	Spain	Pasado Puerto de la Mora		Ca	0	0	1
00:00.0	REDIAM-CMA	396705	Observation	Aegilops neglecta		ESP	Spain		CardeÃ±a	Co	38.217	-4.27821	750
	SIVIM	S-P04398:Aegi	Observation	Aegilops neglecta Req. ex Bertol.	Req. ex Bert	ES	Spain	entre la Ermita de San MartÃ-n y Monegrillo			41.61	-0.47	320
	SIVIM	S-P04715:Aegi	Observation	Aegilops neglecta Req. ex Bertol.	Req. ex Bert	ES	Spain	Paridera de Baerla, cerca de Miraflores			41.53	-0.84	250
	SIVIM	S-P07891:Aegi	Observation	Aegilops neglecta Req. ex Bertol.	Req. ex Bert	ES	Spain	marge de la carretera del Port de la Selva a			42.27	3.12	30
00:00.0	FUND. BIODIVER	1835102	Unknown	Aegilops neglecta Req. ex Bertol	Req. ex Bert	ESP	Spain	Puebla de Don Rodrigo, puente de		CR	38.1	-4.1	490
00:00.0	REDIAM-CMA	386009	Observation	Aegilops neglecta		ESP	Spain		Mijas	Ma	36.5965	-4.7075	208
	SIVIM	Q-P02165:Aegi	Observation	Aegilops neglecta Req. ex Bertol.	Req. ex Bert	ES	Spain	Vers la Font de l'Espino			40.26	-0.41	1225
00:00.0	UNEX	10366-1	Observation	Aegilops neglecta Req. ex Bertol.	_	ESP	Spain	Badajoz: BÃ³toa. 29SPD71		Ba	38.9	-7.00001	
00:00.0	GDA	GDA48983-1-1	Specimen	Aegilops neglecta Raf. ex Bertol	Raf. ex Berto	ES	Spain	AlmerÃ-a, SÂª de los Filabres, Beni		AL			800
00:00.0	MA	596929-1	Specimen	Aegilops neglecta Reg. ex Bertol	Reg. ex Bert	ES	Spain	Solana del Pino, AlhorÃ-n		CR	38	-4	
00:00.0	SEV	101363-1	Specimen	Aegilops neglecta Req. ex Bertol.	Req. ex Bert	ES	Spain	Trassierra. Margen derecha del rÃ-c		Co			1
00:00.0	REDIAM-CMA	92865	Observation	Aegilops neglecta		ESP	Spain		BaÃ±os de la Enc	J	38.2547	-3.7794	400
00:00.0	REDIAM-CMA	98144	Observation	Aegilops neglecta		ESP	Spain		AlmadÃ©n de la F	Se	37.7827	-6.086	499
00:00.0	REDIAM-CMA	108009	Observation	Aegilops neglecta		ESP	Spain		La Granada de R	H	37.7568	-6.48847	507
	IPK	96763	Living	Aegilops neglecta Req. ex Bertol.		ESP	Spain	Sudspanien: Andalusien, Sierranevada, ca. 30 km sudostlich von Granada, Lanja					
	FUND. BIODIVER	1115799	Unknown	Aegilops neglecta Req. ex Bertol	Req. ex Bert	ESP	Spain	Moreruela de TÃ¡bara		Za	41.1	-5.1	
	FUND. BIODIVER	1013432	Unknown	Aegilops neglecta Req. & Bertol	Req. & Berto	ESP	Spain	Los Yesos, Tabernas, alrededores		Al	36.1	-2.1	
00:00.0	REDIAM-CMA	330244	Observation	Aegilops neglecta		ESP	Spain		Villanueva del Re	Co	38.1607	-5.12647	731
00:00.0	REDIAM-CMA	379299	Observation	Aegilops neglecta		ESP	Spain		Rus	J	38.1	-3.42589	520
	SIVIM	T-P00949:Aegi	Observation	Aegilops neglecta Req. ex Bertol.	Req. ex Bert	ES	Spain	Proximidades de la Cueva de Canalobre			38.46	-0.47	0
	SIVIM	T-P09171:Aegi	Observation	Aegilops neglecta Req. ex Bertol.	Req. ex Bert	ES	Spain	Mota del MarquÃ©s			41.61	-5.28	0
	SIVIM	U-P09188:Aegi	Observation	Aegilops neglecta Req. ex Bertol.	Req. ex Bert	ES	Spain	Monterrubio de la ArmuÃ±a, arroyo de la E			40.97	-5.73	0
00:00.0	REDIAM-CMA	238480	Observation	Aegilops neglecta		ESP	Spain		Cumbres Mayores	H	38.0091	-6.66433	510
	SIVIM	T-P18896:Aegi	Observation	Aegilops neglecta Req. ex Bertol.	Req. ex Bert	ES	Spain	AlcalÃ¡ de Henares, finca La Clota			40.46	-3.47	0
	SIVIM	T-P20220:Aegi	Observation	Aegilops neglecta Req. ex Bertol.	Req. ex Bert	ES	Spain	Baena			37.57	-4.35	0
	FUND. BIODIVER	1762691	Unknown	Aegilops neglecta		ESP	Spain	Volcan de Piedrabuena		CR	38.1	-4.1	
00:00.0	UNEX	10364-1	Observation	Aegilops neglecta Req. ex Bertol.	_	ESP	Spain	Moraleja: 29SPE93		Cc	40	-6.8	
00:00.0	REDIAM-CMA	381897	Observation	Aegilops neglecta		ESP	Spain				37.8578	-3.3783	
	SIVIM	Q-P02000:Aegi	Observation	Aegilops neglecta Req. ex Bertol.	Req. ex Bert	ES	Spain	Vistabella			40.25	-0.29	1250
	ESP004	NC043473	Specimen	Aegilops neglecta REQ. ex BERTOL.		ESP	Spain	BaÃƒÂ±os de Montemayor, province of Ca			40.3167	-5.85	705

00:00.0	SALA	75168-1	Specimen	Aegilops neglecta Req. ex Bertol	Req. ex Bert	ES	Spain	_; Garrovillas de AlconÃ©tar		Cc	0	0	
00:00.0	SEV	49878-1	Specimen	Aegilops neglecta Req. ex Bertol.	Req. ex Bert	ES	Spain	Fuenteheridos		H			1
00:00.0	MGC	7597-1	Unknown	Aegilops neglecta Req.	Req.	ES	Spain	Estepona; San Diego		Ma	36.44	-5.18	1
	IPK	AE 591	Living	Aegilops neglecta Req. ex Bertol. subsp. recta (Zhuk			Spain	nordlich Logrosan, Caceres			39.3481	-5.52528	
00:00.0	REDIAM-CMA	148070	Observation	Aegilops neglecta		ESP	Spain		Santa Olalla del C	H	37.8975	-6.21447	500
00:00.0	REDIAM-CMA	154179	Observation	Aegilops neglecta		ESP	Spain		Santa Olalla del C	H	37.8936	-6.22024	496
00:00.0	COFC	50171-1	Specimen	Aegilops neglecta Req. ex Bertol.	Req. ex Bert	ES	Spain	Valle del Guadiato; rÃ-o de la Cabri		Co			1
	FUND. BIODIVER	1465441	Unknown	Aegilops neglecta Req. ex Bertol	Req. ex Bert	ESP	Spain	AniÃ©s		Hu	42.1	-0.1	1000
00:00.0	REDIAM-CMA	129438	Observation	Aegilops neglecta		ESP	Spain		Cala	H	37.9626	-6.25218	496
00:00.0	REDIAM-CMA	153925	Observation	Aegilops neglecta		ESP	Spain		Santa Olalla del C	H	37.8934	-6.22009	496
00:00.0	REDIAM-CMA	177592	Observation	Aegilops neglecta		ESP	Spain		AlanÃ-s	Se	38.0277	-5.70092	676
00:00.0	SEV	101364-1	Specimen	Aegilops neglecta Req. ex Bertol.	Req. ex Bert	ES	Spain	Entre Constantina y El Pedroso de l		Se			1
00:00.0	SEV	108287-1	Specimen	Aegilops neglecta Req. ex Bertol.	Req. ex Bert	ES	Spain	Sierra de Rute. Arroyo cercano al C		Co			1
	FUND. BIODIVER	65368	Unknown	Aegilops neglecta Req. ex Bertol	Req. ex Bert	ESP	Spain	Guijuelo		Sa	40.1	-5.1	
00:00.0	JBS	465-1	Specimen	Aegilops neglecta Req. ex Bertol.	Req. ex Bert	ES	Spain	Barcelona; Barcelona		CT			1
	SIVIM	T-P00694:Aegi	Observation	Aegilops neglecta Req. ex Bertol.	Req. ex Bert	ES	Spain	Villena			38.56	-0.93	0
	SIVIM	T-P00929:Aegi	Observation	Aegilops neglecta Req. ex Bertol.	Req. ex Bert	ES	Spain	Polop; estribaciones de Aitana			38.54	-0.24	0
00:00.0	GDA	GDA48364-1-1	Specimen	Aegilops neglecta Req. ex Bertol.	Req. ex Bert	ES	Spain	Guadalajara, ArbancÃ³n, Barranco d		GU			950
	SANT	50813	Specimen	Aegilops neglecta Req. ex Bertol.		ES	Spain	ArbancÃ³n, Barranco de la Hoz. For		Gu			
	FUND. BIODIVER	1674403	Unknown	Aegilops neglecta		ESP	Spain	Lucillos, hacia Cardiel de los Monte		To	39.1	-4.1	350
00:00.0	SEV	49879-1	Specimen	Aegilops neglecta Req. ex Bertol.	Req. ex Bert	ES	Spain	Entre La UmbrÃ-a y Puerto Moral		H			1
00:00.0	REDIAM-CMA	251508	Observation	Aegilops neglecta		ESP	Spain		Linares de la Sier	H	37.851	-6.61128	600
	SIVIM	T-P31988:Aegi	Observation	Aegilops neglecta Req. ex Bertol.	Req. ex Bert	ES	Spain	de AlcalÃ¡ de Henares, finca La Clota , Pro			40.46	-3.47	0
	SIVIM	U-P06721:Aegi	Observation	Aegilops neglecta Req. ex Bertol.	Req. ex Bert	ES	Spain	Rocha da Pena			37.22	-8.09	0
	SIVIM	T-P00899:Aegi	Observation	Aegilops neglecta Req. ex Bertol.	Req. ex Bert	ES	Spain	Base de la sierra Aitana			38.63	-0.35	0
	SIVIM	T-P02247:Aegi	Observation	Aegilops neglecta Req. ex Bertol.	Req. ex Bert	ES	Spain	Coto Dorda (Cartagena, al sur del camino d			37.57	-1.07	0
	ESP004	NC043469	Specimen	Aegilops neglecta REQ. ex BERTOL.		ESP	Spain	Fuencaliente, province of Ciudad Real			38.4	-4.3	700
00:00.0	HUAL	9326-1	Specimen	Aegilops neglecta Req. ex Bertol.	Req. ex Bert	ES	Spain	TÃ-jola; La Ventilla		Al	37.2	-2.03	875
	FUND. BIODIVER	120461	Unknown	Aegilops neglecta Req. ex Bertol	Req. ex Bert	ESP	Spain	Montejo de la Vega de la Serrezuela		Sg	41.1	-3.1	
00:00.0	REDIAM-CMA	111026	Observation	Aegilops neglecta		ESP	Spain		Segura de la Sier	J	38.2998	-2.65012	1092
	SIVIM	T-P15730:Aegi	Observation	Aegilops neglecta Req. ex Bertol.	Req. ex Bert	ES	Spain	zonas elevadas de la Sierra de Aguas			36.85	-4.79	0
00:00.0	MA	596937-1	Specimen	Aegilops neglecta Req. ex Bertol	Req. ex Bert	ES	Spain	Fuencaliente, valle del rÃ-o Cerecec		CR	38	-4	
	FUND. BIODIVER	1120224	Unknown	Aegilops neglecta Req. ex Bertol	Req. ex Bert	ESP	Spain	Fresno de la Ribera, La Cernia		Za	41.1	-5.1	
00:00.0	FUND. BIODIVER	1712963	Unknown	Aegilops neglecta Req. ex Bertol	Req. ex Bert	ESP	Spain	Menorca, Alaior, Son Ladico		PM	39.1	3.1	100
00:00.0	REDIAM-CMA	51211	Observation	Aegilops neglecta		ESP	Spain		Torres	J	37.7454	-3.55122	1291
	SIVIM	T-P00938:Aegi	Observation	Aegilops neglecta Req. ex Bertol.	Req. ex Bert	ES	Spain	Barranco de las Ovejas			38.28	-0.59	0
	SIVIM	T-P06090:Aegi	Observation	Aegilops neglecta Req. ex Bertol.	Req. ex Bert	ES	Spain	de la comarca del RÃ-o Aguas			37.13	-1.98	150
	SIVIM	T-P09149:Aegi	Observation	Aegilops neglecta Req. ex Bertol.	Req. ex Bert	ES	Spain	Amusquillo			41.72	-4.32	0
	SIVIM	Q-P01998:Aegi	Observation	Aegilops neglecta Req. ex Bertol.	Req. ex Bert	ES	Spain	Vora la carretera de Vistabella a CastellÃ³			40.25	-0.29	1225
	IDBD-GN	42504	Observation	Aegilops neglecta Req. ex Bertol.	Req. ex Bert	ES	Spain		Tafalla	Na	42.4884	-1.72365	
	SIVIM	T-P17786:Aegi	Observation	Aegilops neglecta Req. ex Bertol.	Req. ex Bert	ES	Spain	Villarejo de SalvanÃ©s			40.1	-3.35	0

	SIVIM	T-P18901:Aegi	Observation	Aegilops neglecta Req. ex Bertol.	Req. ex Bert	ES	Spain	AÃ±over de Tajo, cerca de la carretera de			39.92	-3.81	0
	SIVIM	T-P19972:Aegi	Observation	Aegilops neglecta Req. ex Bertol.	Req. ex Bert	ES	Spain	Coria			39.9	-6.54	263
	SIVIM	T-P20226:Aegi	Observation	Aegilops neglecta Req. ex Bertol.	Req. ex Bert	ES	Spain	Villaluenga del Rosario			36.66	-5.46	0
00:00.0	REDIAM-CMA	198022	Observation	Aegilops neglecta		ESP	Spain		Espiel	Co	38.141	-5.11551	641
00:00.0	COFC	52516-1	Specimen	Aegilops neglecta Req. ex Bertol.	Req. ex Bert	ES	Spain	Logrosan; entre el municipio y Bezo		Cc	39	-5	700
	SIVIM	T-P18880:Aegi	Observation	Aegilops neglecta Req. ex Bertol.	Req. ex Bert	ES	Spain	Valle de La Mosson			43.52	3.86	0
	SIVIM	T-P20387:Aegi	Observation	Aegilops neglecta Req. ex Bertol.	Req. ex Bert	ES	Spain	Dehesa Raso de Portillo. Boecillo			41.44	-4.79	0
00:00.0	REDIAM-CMA	137486	Observation	Aegilops neglecta		ESP	Spain		Cala	H	37.9715	-6.27351	493
00:00.0	REDIAM-CMA	152367	Observation	Aegilops neglecta		ESP	Spain		AlmadÃ©n de la P	Se	37.872	-5.95244	200
00:00.0	HSS	6552	Specimen	Aegilops neglecta Req. ex Bertol	Req. ex Bert	ES	Spain	SalvaleÃ³n		Ba	38.512	-6.76379	
00:00.0	MGC	40317-1	Unknown	Aegilops neglecta Req.	Req.	ES	Spain	Ronda; Cerro del Cincho		Ma	36.697	-5.177	820
00:00.0	REDIAM-CMA	142640	Observation	Aegilops neglecta		ESP	Spain		CardeÃ±a	Co	38.2194	-4.27598	750
00:00.0	REDIAM-CMA	180121	Observation	Aegilops neglecta		ESP	Spain		Constantina	Se	37.9654	-5.5678	657
	IPK	77844	Living	Aegilops neglecta Req. ex Bertol. subsp. recta		ESP	Spain	nordlich Logrosan, Caceres					
	FUND. BIODIVER	1048664	Unknown	Aegilops neglecta Req ex Bertol	Req ex Berto	ESP	Spain	Montes de Propios de Jerez de la F		Ca	36.1	-5.1	
	FUND. BIODIVER	1050670	Unknown	Aegilops neglecta Req. & Bertol	Req. & Berto	ESP	Spain	Sierra de Algarrobo		Ca	35.1	-5.1	
	FUND. BIODIVER	1835093	Unknown	Aegilops neglecta Req. ex Bertol	Req. ex Bert	ESP	Spain	Despenaperros		CR	38.1	-3.1	
00:00.0	FUND. BIODIVER	1835114	Unknown	Aegilops neglecta Req. ex Bertol	Req. ex Bert	ESP	Spain	Abenojar, entre Casa de la Solana y		CR	38.1	-4.1	680
00:00.0	REDIAM-CMA	388383	Observation	Aegilops neglecta		ESP	Spain		Rute	Co	37.3216	-4.34827	734
	SIVIM	Q-P02123:Aegi	Observation	Aegilops neglecta Req. ex Bertol.	Req. ex Bert	ES	Spain	Rambla del Pla			40.26	-0.41	1150
	SIVIM	U-P13966:Aegi	Observation	Aegilops neglecta Req. ex Bertol.	Req. ex Bert	ES	Spain	El Bonillo			38.75	-2.65	0
00:00.0	REDIAM-CMA	35040	Observation	Aegilops neglecta		ESP	Spain		Torres	J	37.7807	-3.49442	1146
00:00.0	REDIAM-CMA	58200	Observation	Aegilops neglecta		ESP	Spain		Orcera	J	38.4633	-2.81497	753
	SIVIM	T-P00693:Aegi	Observation	Aegilops neglecta Req. ex Bertol.	Req. ex Bert	ES	Spain	Villena			38.56	-0.93	0
	SIVIM	T-P00904:Aegi	Observation	Aegilops neglecta Req. ex Bertol.	Req. ex Bert	ES	Spain	Alto de la Carrasqueta			38.55	-0.59	1000
00:00.0	SALA	30604-1	Specimen	Aegilops neglecta Req. ex Bertol	Req. ex Bert	ES	Spain	_; Cuelgamures		Za			
00:00.0	GDAC	GDAC36990-2	Specimen	Aegilops neglecta Req. ex Bertol	Req. ex Bert	ES	Spain	Granada, Iznalloz, prox. Cortijo de D		GR			950
	FUND. BIODIVER	1674402	Unknown	Aegilops neglecta		ESP	Spain	Talavera, carretera a Segurilla		To	39.1	-4.1	440
00:00.0	MGC	52705-1	Unknown	Aegilops neglecta Req.	Req.	ES	Spain	BenarrabÃ¡; La Mediana		Ma	36.524	-5.273	150
	SIVIM	T-P27105:Aegi	Observation	Aegilops neglecta Req. ex Bertol.	Req. ex Bert	ES	Spain	*			41.34	-0.37	240
00:00.0	MA	711523-1	Specimen	Aegilops neglecta Req. ex Bertol.	Req. ex Bert	ES	Spain	ArbancÃ³n, Barranco de la Hoz		Gu	4	-3	
	FUND. BIODIVER	1011319	Unknown	Aegilops neglecta Req. ex Bertol	Req. ex Bert	ESP	Spain	Sierra de EspuÃ±a		Mu	37.1	-1.1	1200
	FUND. BIODIVER	1027215	Unknown	Aegilops neglecta Req. ex Bertol	Req. ex Bert	ESP	Spain	Marmolejo		J	38.1	-3.1	600
00:00.0	FUND. BIODIVER	1774962	Unknown	Aegilops neglecta Req. ex Bertol	Req. ex Bert	ESP	Spain	Almunia de San Juan, Ariestolas, rib		Hu	41.1	-0.9	270
	FUND. BIODIVER	1715692	Unknown	Aegilops neglecta		ESP	Spain	Roses, marge de cami al puig de la		Ge	42.1	2.1	
00:00.0	REDIAM-CMA	326892	Observation	Aegilops neglecta		ESP	Spain		BaÃ±os de la Enc	J	38.2645	-3.70205	500
	SIVIM	Q-P01780:Aegi	Observation	Aegilops neglecta Req. ex Bertol.	Req. ex Bert	ES	Spain	Alicante, entre la Talaia y la Rambla de les			38.28	-0.59	0
	SIVIM	U-P08104:Aegi	Observation	Aegilops neglecta Req. ex Bertol.	Req. ex Bert	ES	Spain	Los Cercones, LogrosÃ¡n			39.27	-5.55	0
	SIVIM	P-P04301:Aegi	Observation	Aegilops neglecta Req. ex Bertol.	Req. ex Bert	ES	Spain	Fredes, salida del pueblo			40.7	0.15	1230
	SIVIM	Q-P02002:Aegi	Observation	Aegilops neglecta Req. ex Bertol.	Req. ex Bert	ES	Spain	Sant Joan			40.17	-0.41	1275
00:00.0	COFC	26611-1	Specimen	Aegilops neglecta Req. ex Bertol.	Req. ex Bert	ES	Spain	Cumbres Mayores; huerto de la Lun		H			1

00:00.0	REDIAM-CMA	251090	Observation	Aegilops neglecta		ESP	Spain		AlÃ¡jar	H	37.8285	-6.63236	457
00:00.0	UNEX	28637-1	Observation	Aegilops neglecta Req. ex Bertol.	_	ESP	Spain	Cabeza del Buey: Pastizal llano de		Ba	38.6	-5.3	
	SIVIM	T-P27059:Aegi	Observation	Aegilops neglecta Req. ex Bertol.	Req. ex Bert	ES	Spain	*			41.4	0.46	280
	SIVIM	T-P28620:Aegi	Observation	Aegilops neglecta Req. ex Bertol.	Req. ex Bert	ES	Spain	Cerro Calero, Campillo de Deleitosa			39.59	-6.84	0
	SIVIM	T-P28621:Aegi	Observation	Aegilops neglecta Req. ex Bertol.	Req. ex Bert	ES	Spain	Cerro Calero, Campillo de Deleitosa			39.59	-6.84	0
	SIVIM	T-P28906:Aegi	Observation	Aegilops neglecta Req. ex Bertol.	Req. ex Bert	ES	Spain	Almaraz			39.71	-5.68	270
	SIVIM	T-P31985:Aegi	Observation	Aegilops neglecta Req. ex Bertol.	Req. ex Bert	ES	Spain	de AlcalÃ¡ de Henares, finca La Clota , Pro			40.46	-3.47	0
	FUND. BIODIVER	1762690	Unknown	Aegilops neglecta		ESP	Spain	Cerro de la Higuera		CR	39.1	-4.1	
	SIVIM	Q-P01999:Aegi	Observation	Aegilops neglecta Req. ex Bertol.	Req. ex Bert	ES	Spain	Vistabella			40.25	-0.29	1250
00:00.0	GDAC	GDAC26136-1	Specimen	Aegilops neglecta Req. ex Bertol	Req. ex Bert	ES	Spain	Granada, SÂª de Baza, entre la esta		GR			0
00:00.0	SALA	71582-1	Specimen	Aegilops neglecta Req. ex Bertol	Req. ex Bert	ES	Spain	_; BelvÃ-s de Monroy, Casas de Be		Cc			
00:00.0	COA	41227-1	Specimen	Aegilops neglecta Req. ex Bertol.	Req. ex Bert	ES	Spain	JardÃ-n BotÃ¡nico de CÃ³rdoba		Co	37.84	-4.82	
00:00.0	MGC	28802-1	Unknown	Aegilops neglecta Req.	Req.	ES	Spain	AlhaurÃ-n el Grande; Sierra de Mija		Ma	0	0	1
00:00.0	SEV	71060-1	Specimen	Aegilops neglecta Req. ex Bertol.	Req. ex Bert	ES	Spain	Algeciras. Cerro del Rayo		Ca			200
	FUND. BIODIVER	76098	Unknown	Aegilops neglecta Req. Ex Berto	Req. Ex Bert	ESP	Spain	Cuelgamures		Za	41.1	-5.1	
	IPK	AE 1194	Living	Aegilops neglecta Req. ex Bertol.			Spain	Pablo Romaso, Stra?e Sevilla - Huelva, 4 k			37.3969	-6.75722	40
00:00.0	MGC	2520-1	Unknown	Aegilops neglecta Req.	Req.	ES	Spain	Ãlora; _		Ma	36.81	-4.74	1
	FUND. BIODIVER	1125956	Unknown	Aegilops neglecta Req. ex Bertol	Req. ex Bert	ESP	Spain	Dehesa Raso de Portillo		Va	41.1	-4.1	
	SIVIM	T-P06097:Aegi	Observation	Aegilops neglecta Req. ex Bertol.	Req. ex Bert	ES	Spain	altiplanicie de Venta de Yesos			37.04	-2.32	0
	SIVIM	T-P09184:Aegi	Observation	Aegilops neglecta Req. ex Bertol.	Req. ex Bert	ES	Spain	Soto del Cerrato			41	-4.42	0
00:00.0	GDA	GDA48364-1	Specimen	Aegilops neglecta Reg. ex Bertol.	Reg. ex Bert	ES	Spain	Guadalajara, ArbancÃ³n, Barranco d		GU			950
00:00.0	COFC	36333-1	Specimen	Aegilops neglecta Req. ex Bertol.	Req. ex Bert	ES	Spain	IznÃƒÂ¡jar; cerro de la Camorrilla y		Co			1
00:00.0	REDIAM-CMA	200945	Observation	Aegilops neglecta		ESP	Spain		Constantina	Se	37.8637	-5.66971	538
	SIVIM	T-P18892:Aegi	Observation	Aegilops neglecta Req. ex Bertol.	Req. ex Bert	ES	Spain	AÃ±over de Tajo, cerca de la carretera de			39.92	-3.81	0
	SIVIM	T-P19691:Aegi	Observation	Aegilops neglecta Req. ex Bertol.	Req. ex Bert	ES	Spain	Baza, pr. Dos Picos, Loma de la Piedra			37.13	-2.77	1930
	SIVIM	T-P20042:Aegi	Observation	Aegilops neglecta Req. ex Bertol.	Req. ex Bert	ES	Spain	Cerro Calero (Campillo de Deleitosa)			39.62	-5.67	300
	SIVIM	T-P20338:Aegi	Observation	Aegilops neglecta Req. ex Bertol.	Req. ex Bert	ES	Spain	La Pedraja de Portillo			41.44	-4.67	0
	IPK	AE 585	Living	Aegilops neglecta Req. ex Bertol. subsp. recta (Zhuk.			Spain	bei Molinillo nahe Bejar, Salamanca			40.4664	-5.06667	
	FUND. BIODIVER	1715693	Unknown	Aegilops neglecta		ESP	Spain	Palau-saverdera, prat pasturat entre		Ge	42.1	2.1	
	SIVIM	P-P02751:Aegi	Observation	Aegilops neglecta Req. ex Bertol.	Req. ex Bert	ES	Spain	Vallirana, en el comienzo de la carretera de			41.36	1.8	350
	SIVIM	P-P04303:Aegi	Observation	Aegilops neglecta Req. ex Bertol.	Req. ex Bert	ES	Spain	la SÃ¨nia, umbrÃ-a del Tossal del Rei, baja			40.61	0.16	1280
	SIVIM	Q-P02003:Aegi	Observation	Aegilops neglecta Req. ex Bertol.	Req. ex Bert	ES	Spain	Pla de Dalt, vers la Font de l'Espino			40.26	-0.41	1250
00:00.0	SALA	30597-1	Specimen	Aegilops neglecta Req. ex Bertol	Req. ex Bert	ES	Spain	_; CaÃ±izal		Za			
00:00.0	SALA	30598-1	Specimen	Aegilops neglecta Req. ex Bertol	Req. ex Bert	ES	Spain	_; El Pego		Za			
00:00.0	GDA	GDA30025-1-2	Specimen	Aegilops neglecta Req. ex Bertol.	Req. ex Bert	ES	Spain	CÃ³rdoba, Santo Domingo.		CO			0
00:00.0	MGC	63378-1	Unknown	Aegilops neglecta Req.	Req.	ES	Spain	BenaojÃ¡n; Cueva del Gato		Ma	36.759	-5.235	440
00:00.0	JBS	461-1	Specimen	Aegilops neglecta Req. ex Bertol.	Req. ex Bert	ES	Spain	SÃ³ller; Can Prohom		Mll	39.77	2.68	1
	W	42569	Unknown	Aegilops neglecta Req. ex Bertol.		ESP	Spain	Catalogne: Barcelone, sables granitiques du Tibidabo.					
00:00.0	COA	41177-1	Specimen	Aegilops neglecta Req. ex Bertol.	Req. ex Bert	ES	Spain	De Villanueva a CardeÃ±a, C420 Ki		Co	38.3	-4.6	
00:00.0	REDIAM-CMA	243892	Observation	Aegilops neglecta		ESP	Spain		Aroche	H	37.9438	-6.88225	472
00:00.0	REDIAM-CMA	241720	Observation	Aegilops neglecta		ESP	Spain		El Real de la Jara	Se	37.9217	-6.13187	495

00:00.0	REDIAM-CMA	250300	Observation	Aegilops neglecta		ESP	Spain		AlÃ¡jar	H	37.8301	-6.64393	399
	SIVIM	T-P27051:Aegi	Observation	Aegilops neglecta Req. ex Bertol.	Req. ex Bert	ES	Spain	*			41.89	-1.19	320
	SIVIM	T-P31982:Aegi	Observation	Aegilops neglecta Req. ex Bertol.	Req. ex Bert	ES	Spain	AÃ±over de Tajo, cerca de la carretera de I			39.92	-3.81	0
	SIVIM	U-P06651:Aegi	Observation	Aegilops neglecta Req. ex Bertol.	Req. ex Bert	ES	Spain	CabeÃ§o da CÃ¢mara			37.04	-8.1	0
	IPK	AE 589	Living	Aegilops neglecta Req. ex Bertol. subsp. recta (Zhuk.			Spain	Finca La Cigue?ela, ostlich Aracena, Huelv			37.8919	-6.52083	
	FUND. BIODIVER	1093241	Unknown	Aegilops neglecta		ESP	Spain	Cerro de Aldeamoret		Cc	39.1	-6.1	
	FUND. BIODIVER	1093263	Unknown	Aegilops neglecta Req.	Req.	ESP	Spain	Comarca de la Vera		Cc	39.1	-5.1	
00:00.0	FUND. BIODIVER	1946427	Unknown	Aegilops neglecta Req. ex Bertol	Req. ex Bert	ESP	Spain	bajada Puerto Caracollera		CR	38.1	-4.1	
00:00.0	MA	618898-1	Specimen	Aegilops neglecta Req ex Bertol	Req ex Berto	ES	Spain	Cazorla, Nava de San Pablo		J	37	-2	
00:00.0	COA	37952-1	Specimen	Aegilops neglecta Req. ex Bertol.	Req. ex Bert	ES	Spain	Carcabuey		Co	37.4	-4.36	
00:00.0	REDIAM-CMA	20684	Observation	Aegilops neglecta		ESP	Spain		Cambil	J	37.7311	-3.5075	1623
00:00.0	REDIAM-CMA	403396	Observation	Aegilops neglecta		ESP	Spain		CardeÃ±a	Co	38.2418	-4.27396	710
00:00.0	REDIAM-CMA	281956	Observation	Aegilops neglecta		ESP	Spain		Hinojosa del Duq	Co	38.4694	-5.16592	541
	SIVIM	S-P00145:Aegi	Observation	Aegilops neglecta Req. ex Bertol.	Req. ex Bert	ES	Spain	*			41.27	1.92	0
	SIVIM	S-P04921:Aegi	Observation	Aegilops neglecta Req. ex Bertol.	Req. ex Bert	ES	Spain	La Almolda			41.51	-0.24	0
	SIVIM	S-P06267:Aegi	Observation	Aegilops neglecta Req. ex Bertol.	Req. ex Bert	ES	Spain	del SE de Linares de Mora			40.26	-0.64	0
	IPK	77871	Living	Aegilops neglecta Req. ex Bertol. subsp. recta		ESP	Spain	bei Molinillo nahe Bejar, Salamanca					
00:00.0	UNEX	10365-1	Observation	Aegilops neglecta Req. ex Bertol.	_	ESP	Spain	Cilleros: Sierra de Santa Olalla. 29T		Cc	40.1	-5.7	
00:00.0	COA	41171-1	Specimen	Aegilops neglecta Req. ex Bertol.	Req. ex Bert	ES	Spain	4 Km al norte de Hornachuelos		Co	37.84	-5.27	
00:00.0	SALA	68751-1	Specimen	Aegilops neglecta Req. ex Bertol	Req. ex Bert	ES	Spain	_; Calzada de Oropesa		To			
00:00.0	GDAC	GDAC21285-1	Specimen	Aegilops neglecta Req. ex Bertol	Req. ex Bert	ES	Spain	Madrid, Embalse de Santillana.		M			0
	IPK	77862	Living	Aegilops neglecta Req. ex Bertol. subsp. recta		ESP	Spain	sudlich Plasencia, Caceres					
	FUND. BIODIVER	1093258	Unknown	Aegilops neglecta		ESP	Spain	Dehesa de Valdeobispo, Almaraz		Cc	39.1	-6.1	
00:00.0	FUND. BIODIVER	1946422	Unknown	Aegilops neglecta Req. ex Bertol	Req. ex Bert	ESP	Spain	San Lorenzo de Calatrava, umbria d		CR	38.1	-3.1	620
	IPK	AE 592	Living	Aegilops neglecta Req. ex Bertol. subsp. recta (Zhuk.			Spain	Stra?e Logrosan - Guadalupe, Caceres			39.3783	-5.65	
	SIVIM	R-P05383:Aegi	Observation	Aegilops neglecta Req. ex Bertol.	Req. ex Bert	ES	Spain	Entre RamacastaÃ±as y MombeltrÃ¡n			40.18	-5.11	0
	SIVIM	S-P00108:Aegi	Observation	Aegilops neglecta Req. ex Bertol.	Req. ex Bert	ES	Spain	*			41.27	1.92	0
	SIVIM	S-P02874:Aegi	Observation	Aegilops neglecta Req. ex Bertol.	Req. ex Bert	ES	Spain	massif du Tibidabo: a l'est de Molins de Re			41.36	1.92	200
	SIVIM	S-P04718:Aegi	Observation	Aegilops neglecta Req. ex Bertol.	Req. ex Bert	ES	Spain	entre Valmadrid y Torrecilla			41.44	-0.96	270
	SIVIM	S-P06248:Aegi	Observation	Aegilops neglecta Req. ex Bertol.	Req. ex Bert	ES	Spain	Valdelinares			40.36	-0.99	0
	DEU146	AE 586	Specimen	Aegilops neglecta Req. ex Bertol. subsp. recta		ESP	Spain	Strae Piedralaves - S. Martin de Viera, Avila					
00:00.0	SALA	81480-1	Specimen	Aegilops neglecta Req. ex Bertol	Req. ex Bert	ES	Spain	_; Tejeda de TiÃ©tar		Cc	40.02	-5.87	
00:00.0	HUAL	5971-1	Specimen	Aegilops neglecta Req. ex Bertol.	Req. ex Bert	ES	Spain	SÂª de GÃ¡dor, Cerro Los Lobos		Al	36.825	-2.58	
00:00.0	COFC	46899-1	Specimen	Aegilops neglecta Req. ex Bertol.	Req. ex Bert	ES	Spain	Hornachuelos; camino vecinal de V		Co			1
	FUND. BIODIVER	1027213	Unknown	Aegilops neglecta Req. ex Bertol	Req. ex Bert	ESP	Spain	Marmolejo		J	38.1	-3.1	800
00:00.0	SEV	108385-1	Specimen	Aegilops neglecta Req. ex Bertol.	Req. ex Bert	ES	Spain	Carretera a San Calixto. Hornachue		Co			1
00:00.0	COFC	16301-1	Specimen	Aegilops neglecta Req. ex Bertol.	Req. ex Bert	ES	Spain	Fuente Obejuna; carretera hacia Lo		Co	38	-5	1
00:00.0	REDIAM-CMA	331666	Observation	Aegilops neglecta		ESP	Spain		Obejo	Co	38.1316	-4.75797	500
	SIVIM	U-P08102:Aegi	Observation	Aegilops neglecta Req. ex Bertol.	Req. ex Bert	ES	Spain	EstaciÃ³n Ferrocarril, LogrosÃ¡n			39.27	-5.55	0
00:00.0	COA	41172-1	Specimen	Aegilops neglecta Req. ex Bertol.	Req. ex Bert	ES	Spain	3 Km al norte de Santa Eufemia		Co	38.56	-4.95	
	SIVIM	T-P01105:Aegi	Observation	Aegilops neglecta Req. ex Bertol.	Req. ex Bert	ES	Spain	3 Km north of the towm of Eivissa, near the			38.92	1.38	0

	SIVIM	T-P09179:Aegi	Observation	Aegilops neglecta Req. ex Bertol.	Req. ex Bert	ES	Spain	Iscar			41.45	-4.55	0
00:00.0	REDIAM-CMA	135523	Observation	Aegilops neglecta		ESP	Spain		Zufre	H	37.8984	-6.38652	394
00:00.0	REDIAM-CMA	149882	Observation	Aegilops neglecta		ESP	Spain		AlanÃs	Se	38.0049	-5.57515	699
00:00.0	REDIAM-CMA	173335	Observation	Aegilops neglecta		ESP	Spain		AlanÃs	Se	38.0127	-5.58362	665
00:00.0	REDIAM-CMA	186568	Observation	Aegilops neglecta		ESP	Spain		Cala	H	37.9759	-6.35497	594
	SIVIM	T-P17447:Aegi	Observation	Aegilops neglecta Req. ex Bertol.	Req. ex Bert	ES	Spain	ChinchÃ³n			40.1	-3.46	0
00:00.0	REDIAM-CMA	268700	Observation	Aegilops neglecta		ESP	Spain		Adamuz	Co	38.1049	-4.53754	495
00:00.0	REDIAM-CMA	287959	Observation	Aegilops neglecta		ESP	Spain		Aroche	H	37.8919	-6.91043	400
	SIVIM	U-P08094:Aegi	Observation	Aegilops neglecta Req. ex Bertol.	Req. ex Bert	ES	Spain	Los Cercones, LogrosÃ¡n			39.27	-5.55	0
00:00.0	MGC	72819-1	Unknown	Aegilops neglecta Req.	Req.	ES	Spain	Casares; Camino de Los Pobres		Ma	36.442	-5.315	190
00:00.0	SALA	77322-1	Specimen	Aegilops neglecta Req. ex Bertol	Req. ex Bert	ES	Spain	_; GargÃ¼era		Cc	40.06	-5.93	
00:00.0	FUND. BIODIVER	1348606	Unknown	Aegilops neglecta Req. ex Bertol	Req. ex Bert	ESP	Spain	Piedrabuena		CR	39.1	-4.1	700
00:00.0	REDIAM-CMA	91412	Observation	Aegilops neglecta		ESP	Spain		GuarromÃ¡n	J	38.1169	-3.83244	300
00:00.0	REDIAM-CMA	96813	Observation	Aegilops neglecta		ESP	Spain		Hornachuelos	Co	37.9958	-5.45269	623
00:00.0	REDIAM-CMA	117947	Observation	Aegilops neglecta		ESP	Spain		Villacarrillo	J	38.0899	-2.89065	1326
	SIVIM	T-P15746:Aegi	Observation	Aegilops neglecta Req. ex Bertol.	Req. ex Bert	ES	Spain	Arroyo Paredones, Sierra de Aguas			36.85	-4.79	350
00:00.0	REDIAM-CMA	379467	Observation	Aegilops neglecta		ESP	Spain		Sabiote	J	38.1245	-3.30942	412
00:00.0	REDIAM-CMA	148156	Observation	Aegilops neglecta		ESP	Spain		AlanÃs	Se	37.9979	-5.55618	694
00:00.0	REDIAM-CMA	181777	Observation	Aegilops neglecta		ESP	Spain		Cortelazor	H	37.9124	-6.61782	699
00:00.0	COFC	29532-1	Specimen	Aegilops neglecta Req. ex Bertol.	Req. ex Bert	ES	Spain	Alcaracejos; El Escorial		Co			1
00:00.0	REDIAM-CMA	242517	Observation	Aegilops neglecta		ESP	Spain		Almonaster la Re	H	37.9021	-6.78941	500
00:00.0	REDIAM-CMA	250879	Observation	Aegilops neglecta		ESP	Spain		AlÃ¡jar	H	37.8295	-6.62766	470
00:00.0	REDIAM-CMA	282354	Observation	Aegilops neglecta		ESP	Spain		Hinojosa del Duq	Co	38.4139	-5.13989	611
	SIVIM	T-P27058:Aegi	Observation	Aegilops neglecta Req. ex Bertol.	Req. ex Bert	ES	Spain	*			41.25	-0.49	300
	SIVIM	T-P28619:Aegi	Observation	Aegilops neglecta Req. ex Bertol.	Req. ex Bert	ES	Spain	Dehesa de Valdeobispo			39.8	-5.68	0
	SIVIM	T-P31984:Aegi	Observation	Aegilops neglecta Req. ex Bertol.	Req. ex Bert	ES	Spain	de AlcalÃ¡ de Henares, finca La Clota , Pro			40.46	-3.47	0
	SIVIM	U-P06653:Aegi	Observation	Aegilops neglecta Req. ex Bertol.	Req. ex Bert	ES	Spain	Rocha da Pena			37.22	-8.21	0
00:00.0	SALA	82103-1	Specimen	Aegilops neglecta Req. ex Bertol	Req. ex Bert	ES	Spain	_; Valverde del Fresno		Cc	40.27	-7	
00:00.0	SALA	85591-1	Specimen	Aegilops neglecta Req. ex Bertol	Req. ex Bert	ES	Spain	_; Aldeanueva de Figueroa		Sa	41.15	-5.53	
00:00.0	FUND. BIODIVER	1835109	Unknown	Aegilops neglecta Req. ex Bertol	Req. ex Bert	ESP	Spain	Viso del Marques, sierra del Agua		CR	38.1	-3.1	900
00:00.0	REDIAM-CMA	393247	Observation	Aegilops neglecta		ESP	Spain		Cambil	J	37.7321	-3.50604	1631
	SIVIM	R-P02067:Aegi	Observation	Aegilops neglecta Req. ex Bertol.	Req. ex Bert	ES	Spain	Miravet			40.98	0.5	180
	SIVIM	U-P13957:Aegi	Observation	Aegilops neglecta Req. ex Bertol.	Req. ex Bert	ES	Spain	El Ballestero			38.75	-2.53	0
	DEU146	AE 592	Specimen	Aegilops neglecta Req. ex Bertol. subsp. recta		ESP	Spain	Strae Logrosan - Guadalupe, Cceres					
00:00.0	SEV	108750-1	Specimen	Aegilops neglecta Req. ex Bertol.	Req. ex Bert	ES	Spain	Entre Benaocaz y Grazalema		Ca			500
00:00.0	COA	41174-1	Specimen	Aegilops neglecta Req. ex Bertol.	Req. ex Bert	ES	Spain	Km 27 de CÃ³rdoba a Villaviciosa		Co	38.02	-4.94	
00:00.0	SEV	98935-1	Specimen	Aegilops neglecta Req. ex Bertol.	Req. ex Bert	ES	Spain	Gerena		Se			1
00:00.0	GDA	GDA48983-1-2	Specimen	Aegilops neglecta Raf. ex Bertol	Raf. ex Bert	ES	Spain	AlmerÃa, SÂª de los Filabres, Beni		AL			800
	IPK	AE 583	Living	Aegilops neglecta Req. ex Bertol. subsp. recta (Zhuk.			Spain	bei Monasterio de Yuste, Sierra de Gredos			40.1142	-5.26111	
00:00.0	FUND. BIODIVER	1946425	Unknown	Aegilops neglecta Req. ex Bertol	Req. ex Bert	ESP	Spain	Viso del Marques, sierra del Agua		CR	38.1	-3.1	900
00:00.0	REDIAM-CMA	413830	Observation	Aegilops neglecta		ESP	Spain		Villanueva de CÃ	Co	38.2294	-4.5728	656

	SIVIM	S-P00118:Aegi	Observation	Aegilops neglecta Req. ex Bertol.	Req. ex Bert	ES	Spain	*			41.36	2.04	350
	SIVIM	S-P02895:Aegi	Observation	Aegilops neglecta Req. ex Bertol.	Req. ex Bert	ES	Spain	massif d'Ordal: entre St. VicenÃ§ dels Hort			41.36	1.92	130
	SIVIM	S-P04751:Aegi	Observation	Aegilops neglecta Req. ex Bertol.	Req. ex Bert	ES	Spain	1 km al sur de Valmadrid			41.35	-0.96	0
	SIVIM	S-P06258:Aegi	Observation	Aegilops neglecta Req. ex Bertol.	Req. ex Bert	ES	Spain	de Albentosa, en el altiplano de SarriÃ³n			40.09	-0.88	0
	FUND. BIODIVER	1093232	Unknown	Aegilops neglecta		ESP	Spain	El Arco, CaÃ±averal		Cc	39.1	-6.1	
	FUND. BIODIVER	1093254	Unknown	Aegilops neglecta		ESP	Spain	El Portanchito, casas de la pizarra		Cc	39.1	-6.1	
00:00.0	FUND. BIODIVER	1946417	Unknown	Aegilops neglecta Req. ex Bertol	Req. ex Bert	ESP	Spain	Puebla del Principe, cercanias del c		CR	38.1	-2.1	940
00:00.0	FUND. BIODIVER	1946418	Unknown	Aegilops neglecta Req. ex Bertol	Req. ex Bert	ESP	Spain	Puebla de Don Rodrigo, puente de		CR	38.1	-4.1	490
00:00.0	REDIAM-CMA	412042	Observation	Aegilops neglecta		ESP	Spain		La Granada de R.	H	37.7568	-6.48854	507
	SIVIM	S-P01348:Aegi	Observation	Aegilops neglecta Req. ex Bertol.	Req. ex Bert	ES	Spain	Els Clots (Almescar, camino del Toscar)			40.79	0.27	1000
	SIVIM	S-P04369:Aegi	Observation	Aegilops neglecta Req. ex Bertol.	Req. ex Bert	ES	Spain	Ejea de los Caballeros, Santa Anastasia			42.07	-1.3	300
	SIVIM	S-P04707:Aegi	Observation	Aegilops neglecta Req. ex Bertol.	Req. ex Bert	ES	Spain	MagallÃ³n			41.81	-1.55	0
	SIVIM	S-P07755:Aegi	Observation	Aegilops neglecta Req. ex Bertol.	Req. ex Bert	ES	Spain	camÃ- sota QuermanÃ§Ã³			42.27	3	0
00:00.0	FUND. BIODIVER	1374900	Unknown	Aegilops neglecta Req. ex Bertol	Req. ex Bert	ESP	Spain	Montes de Toledo, Parque Natural c		CR	39.1	-4.1	
00:00.0	REDIAM-CMA	142696	Observation	Aegilops neglecta		ESP	Spain		Santa Olalla del C	H	37.905	-6.21659	499
00:00.0	REDIAM-CMA	148403	Observation	Aegilops neglecta		ESP	Spain		Santa Olalla del C	H	37.8977	-6.21484	500
00:00.0	REDIAM-CMA	45155	Observation	Aegilops neglecta		ESP	Spain		Rute	Co	37.3271	-4.34503	860
	SIVIM	T-P00695:Aegi	Observation	Aegilops neglecta Req. ex Bertol.	Req. ex Bert	ES	Spain	Villena			38.56	-0.93	0
	SIVIM	T-P00930:Aegi	Observation	Aegilops neglecta Req. ex Bertol.	Req. ex Bert	ES	Spain	Polop; estribaciones de Aitana			38.54	-0.24	0
00:00.0	REDIAM-CMA	200656	Observation	Aegilops neglecta		ESP	Spain		Tabernas	Al	37.0844	-2.29263	518
	SIVIM	T-P18888:Aegi	Observation	Aegilops neglecta Req. ex Bertol.	Req. ex Bert	ES	Spain	AlcalÃ¡ de Henares, finca La Clota			40.46	-3.47	0
	SIVIM	T-P19683:Aegi	Observation	Aegilops neglecta Req. ex Bertol.	Req. ex Bert	ES	Spain	Tabernas, Venta de los Yesos			37.04	-2.32	0
	SIVIM	T-P20038:Aegi	Observation	Aegilops neglecta Req. ex Bertol.	Req. ex Bert	ES	Spain	Finca de Valdeobispo (Almaraz)			39.8	-5.68	350
00:00.0	COA	41175-1	Specimen	Aegilops neglecta Req. ex Bertol.	Req. ex Bert	ES	Spain	4 Km al norte de Villaharta		Co	38.11	-4.94	
00:00.0	COFC	26930-1	Specimen	Aegilops neglecta Req. ex Bertol.	Req. ex Bert	ES	Spain	CardeÃƒÂ±a; coto 'Santa Elena'		Co			1
	SIVIM	T-P18903:Aegi	Observation	Aegilops neglecta Req. ex Bertol.	Req. ex Bert	ES	Spain	AlcalÃ¡ de Henares, finca La Clota			40.46	-3.47	0
	DEU146	AE 584	Specimen	Aegilops neglecta Req. ex Bertol. subsp. recta		ESP	Spain	sdlich Plasencia, Cceres					
00:00.0	GDA	GDA48983-1	Specimen	Aegilops neglecta Raf. ex Bertol	Raf. ex Berto	ES	Spain	AlmerÃ-a, SÂª de los Filabres, Beni		AL			800
	BDBCV-General	279917	Observation	Aegilops neglecta		ESPA	Spain	Teresa de Cofren	El Valle de Ayora	Valenc	39.146	-0.97503	
00:00.0	REDIAM-CMA	42432	Observation	Aegilops neglecta		ESP	Spain		Cambil	J	37.7325	-3.50607	1631
00:00.0	REDIAM-CMA	48194	Observation	Aegilops neglecta		ESP	Spain		Rute	Co	37.3278	-4.33226	800
	FUND. BIODIVER	1715000	Unknown	Aegylops neglecta Req. Ex Berto	Req. Ex Bert	ESP	Spain	Sierra las Corchuelas, Parque Natu		Cc	39.1	-5.1	
00:00.0	REDIAM-CMA	201188	Observation	Aegilops neglecta		ESP	Spain		Belmez	Co	38.2501	-5.28261	585
00:00.0	REDIAM-CMA	233373	Observation	Aegilops neglecta		ESP	Spain		Fuente Obejuna	Co	38.1378	-5.34838	599
	SIVIM	T-P18893:Aegi	Observation	Aegilops neglecta Req. ex Bertol.	Req. ex Bert	ES	Spain	AÃ±over de Tajo, cerca de la carretera de I			39.92	-3.81	0
	SIVIM	T-P19965:Aegi	Observation	Aegilops neglecta Req. ex Bertol.	Req. ex Bert	ES	Spain	Dehesa de los Caballos (Plasencia)			39.98	-6.18	0
	SIVIM	T-P20209:Aegi	Observation	Aegilops neglecta Req. ex Bertol.	Req. ex Bert	ES	Spain	Proximidades de Ubrique, en direcciÃ³n Su			36.66	-5.46	0
00:00.0	MGC	71551-1	Unknown	Aegilops neglecta Req.	Req.	ES	Spain	Yunquera; Sierra Cabrilla. Los Labra		Ma	36.756	-4.921	880
00:00.0	SEV	49877-1	Specimen	Aegilops neglecta Req. ex Bertol.	Req. ex Bert	ES	Spain	Entre Aracena y Alajar		H			1
00:00.0	SEV	5992-1	Specimen	Aegilops neglecta Req. ex Bertol.	Req. ex Bert	ES	Spain	BaÃ±os de Montemayor, hacia el pa		Cc			1
00:00.0	SEV	34911-1	Specimen	Aegilops neglecta Req. ex Bertol.	Req. ex Bert	ES	Spain	CardeÃ±a, finca de YegÃ¼erizo		Co			1

00:00.0	UNEX	10361-1	Observation	Aegilops neglecta Req. ex Bertol.	_	ESP	Spain	Sierra de Oliva. 29SQC49		Ba	38.7	-6.2	
00:00.0	HSS	6539	Specimen	Aegilops neglecta Req. ex Bertol	Req. ex Bert	ES	Spain	SalvaleÃ³n		Ba	38.512	-6.76379	
00:00.0	FUND. BIODIVER	1835113	Unknown	Aegilops neglecta Req. ex Bertol	Req. ex Bert	ESP	Spain	Aldea del Rey, cerro de la Higuera		CR	38.1	-3.1	790
00:00.0	REDIAM-CMA	383990	Observation	Aegilops neglecta		ESP	Spain		Cambil	J	37.7313	-3.50758	1623
00:00.0	REDIAM-CMA	390752	Observation	Aegilops neglecta		ESP	Spain		GuarromÃ¡n	J	38.1271	-3.83028	318
	IPK	77834	Living	Aegilops neglecta Req. ex Bertol. subsp. recta		ESP	Spain	Finca La Cigue?ela, ostlich Aracena, Huelva					
	SIVIM	Q-P02122:Aegi	Observation	Aegilops neglecta Req. ex Bertol.	Req. ex Bert	ES	Spain	Rambla del Pla			40.26	-0.41	1150
	SIVIM	Q-P07352:Aegi	Observation	Aegilops neglecta Req. ex Bertol.	Req. ex Bert	ES	Spain	Monteagudo de las Salinas			39.74	-1.94	1000
	SIVIM	U-P13965:Aegi	Observation	Aegilops neglecta Req. ex Bertol.	Req. ex Bert	ES	Spain	El Ballestero			38.84	-2.53	1020
	FUND. BIODIVER	1043764	Unknown	Aegilops neglecta Req. ex Bertol	Req. ex Bert	ESP	Spain	AlhaurÃ-n el Grande, Sierra de Mija		Ma	36.1	-4.1	
	FUND. BIODIVER	1835094	Unknown	Aegilops neglecta Req. ex Bertol	Req. ex Bert	ESP	Spain	Intercalacion calizo-devonica de Alm		CR	38.1	-4.1	
00:00.0	FUND. BIODIVER	1835115	Unknown	Aegilops neglecta Req. ex Bertol	Req. ex Bert	ESP	Spain	Alcubillas, alrededores		CR	38.1	-2.1	830
	DEU146	AE 591	Specimen	Aegilops neglecta Req. ex Bertol. subsp. recta		ESP	Spain	nrdlich Logrosan, Cceres					
00:00.0	ABH	35188-1	Specimen	Aegilops neglecta Req ex Bertol.	Req ex Berto	ES	Spain	Alicante; Lomas del Garbinet, prox.		A	38.38	-0.48	
	SIVIM	Q-P02124:Aegi	Observation	Aegilops neglecta Req. ex Bertol.	Req. ex Bert	ES	Spain	Rambla del Pla			40.26	-0.41	1150
	FUND. BIODIVER	78794	Unknown	Aegilops neglecta Req. ex Portol	Req. ex Port	ESP	Spain	Mojados		Va	41.1	-4.1	
	FUND. BIODIVER	89107	Unknown	Aegilops neglecta Req. ex Bertol	Req. ex Bert	ESP	Spain	Barraco, Valle de Iruelas		Av	40.1	-4.1	
00:00.0	REDIAM-CMA	89956	Observation	Aegilops neglecta		ESP	Spain		BailÃ©n	J	38.1431	-3.80582	393
00:00.0	REDIAM-CMA	106904	Observation	Aegilops neglecta		ESP	Spain		Siles	J	38.4637	-2.57873	1184
00:00.0	REDIAM-CMA	127414	Observation	Aegilops neglecta		ESP	Spain		Cazalla de la Sier	Se	37.9297	-5.71184	443
	SIVIM	T-P15739:Aegi	Observation	Aegilops neglecta Req. ex Bertol.	Req. ex Bert	ES	Spain	Sierra de Aguas			36.85	-4.79	0
00:00.0	BC	836694	Specimen	Aegilops neglecta Req. ex Bertol.	Req. ex Bert	ES	Spain	Cazorla; Cazorla, Nava de San Pab		J	37.934	-2.824	1680
00:00.0	FUND. BIODIVER	1332456	Unknown	Aegilops neglecta Req. ex Bertol	Req. ex Bert	ESP	Spain	Enjambradero		J	38.1	-3.1	
	SIVIM	T-P10901:Aegi	Observation	Aegilops neglecta Req. ex Bertol.	Req. ex Bert	ES	Spain	Tejeda de TiÃ©tar, 'Castillejo'			39.98	-5.92	0
	SIVIM	T-P15733:Aegi	Observation	Aegilops neglecta Req. ex Bertol.	Req. ex Bert	ES	Spain	Arroyo de Paredones, Sierra de Aguas			36.85	-4.79	0
	FUND. BIODIVER	99841	Unknown	Aegilops neglecta Req. ex Bertol	Req. ex Bert	ESP	Spain	Moreruela de TÃ¡bara		Za	41.1	-5.1	
00:00.0	REDIAM-CMA	196990	Observation	Aegilops neglecta		ESP	Spain		Constantina	Se	37.9341	-5.67872	476
	SIVIM	T-P18801:Aegi	Observation	Aegilops neglecta Req. ex Bertol.	Req. ex Bert	ES	Spain	Cerca de FabrÃ©gues			43.53	3.74	20
	SIVIM	T-P19982:Aegi	Observation	Aegilops neglecta Req. ex Bertol.	Req. ex Bert	ES	Spain	Almaraz			39.8	-5.68	320
00:00.0	SALA	52038-1	Specimen	Aegilops neglecta Req. ex Bertol	Req. ex Bert	ES	Spain	_; Pinilla de Toro, El Ama		Za			
	FUND. BIODIVER	1376736	Unknown	Aegilops neglecta Req. ex Bertol	Req. ex Bert	ESP	Spain	Sierra de Baza, Parque Natural		Gr	37.1	-2.1	
00:00.0	REDIAM-CMA	133617	Observation	Aegilops neglecta		ESP	Spain		Santa Olalla del C	H	37.8211	-6.19126	454
00:00.0	REDIAM-CMA	144125	Observation	Aegilops neglecta		ESP	Spain		Santa Olalla del C	H	37.9018	-6.19071	538
00:00.0	REDIAM-CMA	173078	Observation	Aegilops neglecta		ESP	Spain		AlanÃ-s	Se	38.0138	-5.58565	668
00:00.0	REDIAM-CMA	185540	Observation	Aegilops neglecta		ESP	Spain		Cala	H	37.9694	-6.34761	647
00:00.0	COFC	47891-1	Specimen	Aegilops neglecta Req. ex Bertol.	Req. ex Bert	ES	Spain	Aroche; Sierra Pelada; rivera de los		H			1
	IPK	32071	Living	Aegilops neglecta Req. ex Bertol. subsp. recta		ESP	Spain	Stra?e Logrosan - Guadalupe, Caceres					
00:00.0	REDIAM-CMA	268689	Observation	Aegilops neglecta		ESP	Spain		Adamuz	Co	38.1042	-4.53587	495
00:00.0	REDIAM-CMA	281966	Observation	Aegilops neglecta		ESP	Spain		Hinojosa del Duqu	Co	38.4647	-5.15782	550
	SIVIM	T-P29325:Aegi	Observation	Aegilops neglecta Req. ex Bertol.	Req. ex Bert	ES	Spain	Atienza, Guadalajara			41.19	-2.88	1100
	SIVIM	U-P08093:Aegi	Observation	Aegilops neglecta Req. ex Bertol.	Req. ex Bert	ES	Spain	Los Cercones, LogrosÃ¡n			39.27	-5.55	0

00:00.0	REDIAM-CMA	191082	Observation	Aegilops neglecta		ESP	Spain		Constantina	Se	37.9421	-5.63295	699
00:00.0	REDIAM-CMA	201761	Observation	Aegilops neglecta		ESP	Spain		Villaviciosa de CÃ	Co	37.9795	-5.16225	318
00:00.0	REDIAM-CMA	233759	Observation	Aegilops neglecta		ESP	Spain		Espiel	Co	38.1589	-4.90692	700
00:00.0	REDIAM-CMA	237816	Observation	Aegilops neglecta		ESP	Spain		AlmadÃ©n de la F	Se	37.8729	-5.94928	200
	SIVIM	T-P18895:Aegi	Observation	Aegilops neglecta Req. ex Bertol.	Req. ex Bert	ES	Spain	AlcalÃ¡ de Henares, finca La Clota			40.46	-3.47	0
	SIVIM	T-P19967:Aegi	Observation	Aegilops neglecta Req. ex Bertol.	Req. ex Bert	ES	Spain	El Arco (CaÃ±averal)			39.72	-6.43	0
	SIVIM	T-P20212:Aegi	Observation	Aegilops neglecta Req. ex Bertol.	Req. ex Bert	ES	Spain	Sierra de Ubrique			36.66	-5.46	0
	CZE122	01C2108008	Specimen	Aegilops neglecta subsp. recta (ZHUK.) HAMM		ESP	Spain	N Logrosan, Caceres					
	FUND. BIODIVER	76097	Unknown	Aegilops neglecta Req. Ex Berto	Req. Ex Bert	ESP	Spain	CaÃ±izal		Za	41.1	-5.1	
00:00.0	COFC	29693-1	Specimen	Aegilops neglecta Req. ex Bertol.	Req. ex Bert	ES	Spain	Adamuz; coto La ViÃƒÂ±uela		Co			1
00:00.0	REDIAM-CMA	128095	Observation	Aegilops neglecta		ESP	Spain		Cazalla de la Sier	Se	37.9295	-5.712	439
	IPK	AE 1346	Living	Aegilops neglecta Req. ex Bertol.			Spain	Sudspanien: Andalusien, Sierranevada, ca			36.9164	-3.51667	
	SIVIM	T-P15744:Aegi	Observation	Aegilops neglecta Req. ex Bertol.	Req. ex Bert	ES	Spain	Arroyo Paredones, Sierra de Aguas			36.85	-4.79	300
	ESP004	NC043471	Specimen	Aegilops neglecta REQ. ex BERTOL.		ESP	Spain	Casas de Belvis, Belvis de Monroy, provinc			39.8167	-5.58333	296
	SIVIM	T-P15728:Aegi	Observation	Aegilops neglecta Req. ex Bertol.	Req. ex Bert	ES	Spain	bordes de la carretera Ã•ora-Carratraca, ba			36.76	-4.79	0
00:00.0	COFC	29014-1	Specimen	Aegilops neglecta Req. ex Bertol.	Req. ex Bert	ES	Spain	Andujar; coto 'El Gorgojil'		J			1
00:00.0	REDIAM-CMA	164801	Observation	Aegilops neglecta		ESP	Spain		Espiel	Co	38.2123	-4.99033	640
00:00.0	SEV	108749-1	Specimen	Aegilops neglecta Req. ex Bertol.	Req. ex Bert	ES	Spain	Near Coin		Ma			360
	DEU146	AE 1648	Specimen	Aegilops neglecta Req. ex Bertol. subsp. recta		ESP	Spain						
00:00.0	SALA	36020-1	Specimen	Aegilops neglecta Req. ex Bertol	Req. ex Bert	ES	Spain	_; Montemayor del RÃo		Sa			
00:00.0	SEV	108290-1	Specimen	Aegilops neglecta Req. ex Bertol.	Req. ex Bert	ES	Spain	Algodonales. Sierra de LÃjar		Ca			700
	CZE122	01C2108007	Specimen	Aegilops neglecta subsp. recta (ZHUK.) HAMM		ESP	Spain	Finca la Ciguinela E Aracena, 2 km von Puerto Moral, Huelva					
	SIVIM	T-P18884:Aegi	Observation	Aegilops neglecta Req. ex Bertol.	Req. ex Bert	ES	Spain	Al pie de la colina de St. Bauzille			43.53	3.74	0
	SIVIM	T-P20027:Aegi	Observation	Aegilops neglecta Req. ex Bertol.	Req. ex Bert	ES	Spain	El Portanchito, Casas de la pizarra (CÃ¡cer			39.45	-6.44	0
	SIVIM	T-P26920:Aegi	Observation	Aegilops neglecta Req. ex Bertol.	Req. ex Bert	ES	Spain	*			41.33	-0.25	330
00:00.0	SEV	34910-1	Specimen	Aegilops neglecta Req. ex Bertol.	Req. ex Bert	ES	Spain	CardeÃ±a, Km 168 de la carretera d		Co			1
00:00.0	FCO	24526-1	Specimen	Aegilops neglecta Req. ex Bertol.	Req. ex Bert	ES	Spain	Cazorla; Nava de San Pablo		J			
00:00.0	COFC	16302-1	Specimen	Aegilops neglecta Req. ex Bertol.	Req. ex Bert	ES	Spain	Fuente Obejuna; rÃƒÂo ZÃƒÂºjar e		Co	38	-5	1
	SIVIM	T-P27063:Aegi	Observation	Aegilops neglecta Req. ex Bertol.	Req. ex Bert	ES	Spain	*			41.62	-1.31	0
	SIVIM	T-P28907:Aegi	Observation	Aegilops neglecta Req. ex Bertol.	Req. ex Bert	ES	Spain	Campillo de Deleitosa			39.59	-6.95	0
	SIVIM	T-P31986:Aegi	Observation	Aegilops neglecta Req. ex Bertol.	Req. ex Bert	ES	Spain	de AlcalÃ¡ de Henares, finca La Clota , Pro			40.46	-3.47	0
	SIVIM	U-P06655:Aegi	Observation	Aegilops neglecta Req. ex Bertol.	Req. ex Bert	ES	Spain	Morgado de Apra			37.13	-7.98	2700
00:00.0	SALA	71778-1	Specimen	Aegilops neglecta Req. ex Bertol	Req. ex Bert	ES	Spain	_; Casatejada		Cc	39.88	-5.69	
	FUND. BIODIVER	1093230	Unknown	Aegilops neglecta		ESP	Spain	Dehesa de los Caballos, Plasencia		Cc	39.1	-5.1	
00:00.0	FUND. BIODIVER	1946415	Unknown	Aegilops neglecta Req. ex Bertol	Req. ex Bert	ESP	Spain	Piedrabuena, chopera proxima a la		CR	38.1	-4.1	700
00:00.0	COFC	25775-1	Specimen	Aegilops neglecta Req. ex Bertol.	Req. ex Bert	ES	Spain	Santa Olalla del Cala; Sierra de Ara		H	37	-6	1
	IPK	77861	Living	Aegilops neglecta Req. ex Bertol. subsp. recta		ESP	Spain	bei Monasterio de Yuste, Sierra de Gredos, Caceres					
00:00.0	FCO	27632-1	Specimen	Aegilops neglecta Req. ex Bertol.	Req. ex Bert	ES	Spain	ArbancÃ³n, Barranco de la Hoz		Gu			
	SIVIM	S-P04328:Aegi	Observation	Aegilops neglecta Req. ex Bertol.	Req. ex Bert	ES	Spain	Los Palacios, entre La Almunia y El Frasno			41.45	-1.44	0
	SIVIM	S-P04694:Aegi	Observation	Aegilops neglecta Req. ex Bertol.	Req. ex Bert	ES	Spain	Retuerta de Pina			41.43	-0.36	280
00:00.0	REDIAM-CMA	132852	Observation	Aegilops neglecta		ESP	Spain		Constantina	Se	37.905	-5.72686	387

00:00.0	REDIAM-CMA	143540	Observation	Aegilops neglecta		ESP	Spain		Santa Olalla del C	H	37.9043	-6.20486	500
00:00.0	REDIAM-CMA	148509	Observation	Aegilops neglecta		ESP	Spain		AlanÃ-s	Se	37.9986	-5.55581	694
00:00.0	REDIAM-CMA	171967	Observation	Aegilops neglecta		ESP	Spain		Santa Olalla del C	H	37.9207	-6.21355	498
00:00.0	HUAL	9536-1	Specimen	Aegilops neglecta Req. ex Bertol.	Req. ex Bert	ES	Spain	TÃ-jola; La Ventilla		Al	37.2	-2.03	875
	FUND. BIODIVER	1674404	Unknown	Aegilops neglecta		ESP	Spain	San RomÃ¡n		To	39.1	-4.1	480
00:00.0	REDIAM-CMA	252482	Observation	Aegilops neglecta		ESP	Spain		Valsequillo	Co	38.4274	-5.3969	573
	IPK	32067	Living	Aegilops neglecta Req. ex Bertol.		ESP	Spain	Pablo Romaso, Stra?e Sevilla - Huelva, 4 km von Sanlucar la Ma					40
	FUND. BIODIVER	1339636	Unknown	Aegilops neglecta Req. ex Bertol	Req. ex Bert	ESP	Spain	Albacete, provincia		Ab	38.1	-2.1	
00:00.0	FUND. BIODIVER	1348607	Unknown	Aegilops neglecta Req. ex Bertol	Req. ex Bert	ESP	Spain	Piedrabuena, finca El GargantÃ³n		CR	38.1	-4.1	
	FUND. BIODIVER	1946419	Unknown	Aegilops neglecta Req. ex Bertol	Req. ex Bert	ESP	Spain	Provincia de Ciudad Real		CR			
	SIVIM	S-P04373:Aegi	Observation	Aegilops neglecta Req. ex Bertol.	Req. ex Bert	ES	Spain	La Retuerta de Pina			41.43	-0.36	300
	SIVIM	S-P04712:Aegi	Observation	Aegilops neglecta Req. ex Bertol.	Req. ex Bert	ES	Spain	Fuentes hacia Mediana			41.43	-0.72	270
	SIVIM	S-P07890:Aegi	Observation	Aegilops neglecta Req. ex Bertol.	Req. ex Bert	ES	Spain	carretera de Roses a CadaquÃ©s			42.27	3.12	0
00:00.0	MGC	62199-1	Unknown	Aegilops neglecta Req.	Req.	ES	Spain	AlmadÃ©n de la Plata; Entre Almad		Se	37.817	-6.075	350
00:00.0	FUND. BIODIVER	1715694	Unknown	Aegilops neglecta Req. Ex Berto	Req. Ex Bert	ESP	Spain	Roses,al puig de les Garrigues		Ge	42.1	2.1	
	SIVIM	P-P04336:Aegi	Observation	Aegilops neglecta Req. ex Bertol.	Req. ex Bert	ES	Spain	entrada oriental de MÃ¡laga			36.67	-4.45	25
	SIVIM	Q-P02012:Aegi	Observation	Aegilops neglecta Req. ex Bertol.	Req. ex Bert	ES	Spain	Vers el Mas de la Torre			40.26	-0.41	1500
00:00.0	REDIAM-CMA	38333	Observation	Aegilops neglecta		ESP	Spain		Villanueva del Du	Co	38.2795	-4.98854	693
00:00.0	REDIAM-CMA	47504	Observation	Aegilops neglecta		ESP	Spain		Torres	J	37.7455	-3.53902	1393
	SIVIM	T-P01104:Aegi	Observation	Aegilops neglecta Req. ex Bertol.	Req. ex Bert	ES	Spain	Illa Grossa, near the town of Eivissa			38.83	1.38	0
	SIVIM	T-P06094:Aegi	Observation	Aegilops neglecta Req. ex Bertol.	Req. ex Bert	ES	Spain	de Venta el CastaÃ±o, en las Yeseras			37.04	-2.21	280
	SIVIM	T-P09176:Aegi	Observation	Aegilops neglecta Req. ex Bertol.	Req. ex Bert	ES	Spain	CabezÃ³n			41.71	-4.68	0
00:00.0	SEV	49875-1	Specimen	Aegilops neglecta Req. ex Bertol.	Req. ex Bert	ES	Spain	CastaÃ±o del Robledo		H			1
00:00.0	REDIAM-CMA	199339	Observation	Aegilops neglecta		ESP	Spain		Constantina	Se	37.8467	-5.62952	499
00:00.0	COFC	52517-1	Specimen	Aegilops neglecta Req. ex Bertol.	Req. ex Bert	ES	Spain	Sierra de la Oliva		Ba	38	-6	1
	SIVIM	T-P18883:Aegi	Observation	Aegilops neglecta Req. ex Bertol.	Req. ex Bert	ES	Spain	Proximidad inmediata de Mas Rablault			43.44	3.74	20
	SIVIM	T-P26919:Aegi	Observation	Aegilops neglecta Req. ex Bertol.	Req. ex Bert	ES	Spain	*			42.17	-1.66	320
00:00.0	FUND. BIODIVER	1835111	Unknown	Aegilops neglecta Req. ex Bertol	Req. ex Bert	ESP	Spain	bajada Puerto Caracollera		CR	38.1	-4.1	
00:00.0	REDIAM-CMA	390252	Observation	Aegilops neglecta		ESP	Spain		AlmadÃ©n de la P	Se	37.872	-5.94686	200
	SIVIM	Q-P05505:Aegi	Observation	Aegilops neglecta Req. ex Bertol.	Req. ex Bert	ES	Spain	Palau-saverdera, a la carretera de Roses a			42.27	3.12	0
	SIVIM	U-P13962:Aegi	Observation	Aegilops neglecta Req. ex Bertol.	Req. ex Bert	ES	Spain	El Bonillo			38.93	-2.65	0
	FUND. BIODIVER	1093229	Unknown	Aegilops neglecta		ESP	Spain	Dehesa de los Caballos, Plasencia		Cc	39.1	-5.1	
	FUND. BIODIVER	1109625	Unknown	Aegilops neglecta Req. ex Bertl.	Req. ex Bert	ESP	Spain	Aldeanueva de Figueroa		Sa	40.1	-5.1	
00:00.0	FUND. BIODIVER	1946414	Unknown	Aegilops neglecta Req. ex Bertol	Req. ex Bert	ESP	Spain	Fuencaliente, Sierra Madrona, umb		CR	38.1	-4.1	780
00:00.0	REDIAM-CMA	23583	Observation	Aegilops neglecta		ESP	Spain		Mijas	Ma	36.53	-4.70779	100
	SIVIM	S-P04692:Aegi	Observation	Aegilops neglecta Req. ex Bertol.	Req. ex Bert	ES	Spain	Bujaraloz, en la Salada de la Playa, hacia l			41.33	-0.25	0
	FUND. BIODIVER	1125957	Unknown	Aegilops neglecta Req. ex Bertol	Req. ex Bert	ESP	Spain	Pedraja de Portillo		Va	41.1	-4.1	
00:00.0	REDIAM-CMA	39592	Observation	Aegilops neglecta		ESP	Spain		Hornachuelos	Co	37.9282	-5.26424	500
00:00.0	REDIAM-CMA	39898	Observation	Aegilops neglecta		ESP	Spain		Alcaracejos	Co	38.2339	-4.90931	600
00:00.0	REDIAM-CMA	64070	Observation	Aegilops neglecta		ESP	Spain		Villanueva de la R	J	38.1616	-3.88605	464
	IPK	32069	Living	Aegilops neglecta Req. ex Bertol. subsp. recta		ESP	Spain	Finca La Cigue?ela, ostlich Aracena, Huelva					

00:00.0	SALA	102067-1	Specimen	Aegilops neglecta Req.	Req.	ES	Spain	_; Villamayor		Sa	41.01	-5.69	
00:00.0	GDAC	GDAC32510-2	Specimen	Aegilops neglecta Req. ex Bertol	Req. ex Bert	ES	Spain	MÃ¡laga, Antequera, El Torcal.		MA			0
	IPK	77874	Living	Aegilops neglecta Req. ex Bertol. subsp. recta		ESP	Spain	Stra?e Piedralaves - S. Martin de Viera, Avila					
00:00.0	REDIAM-CMA	81754	Observation	Aegilops neglecta		ESP	Spain		Ronda	Ma	36.6914	-5.05086	1300
00:00.0	REDIAM-CMA	106129	Observation	Aegilops neglecta		ESP	Spain		Villanueva de la R	J	38.1259	-3.92243	343
00:00.0	REDIAM-CMA	113838	Observation	Aegilops neglecta		ESP	Spain		Cazalla de la Sier	Se	37.9569	-5.76792	662
00:00.0	REDIAM-CMA	126619	Observation	Aegilops neglecta		ESP	Spain		Cala	H	37.9454	-6.27646	605
00:00.0	FUND. BIODIVER	1835101	Unknown	Aegilops neglecta Req. ex Bertol	Req. ex Bert	ESP	Spain	Puebla del Principe, cercanias del c		CR	38.1	-2.1	940
	SIVIM	T-P15735:Aegi	Observation	Aegilops neglecta Req. ex Bertol.	Req. ex Bert	ES	Spain	Sierra de Aguas			36.85	-4.79	0
00:00.0	REDIAM-CMA	392047	Observation	Aegilops neglecta		ESP	Spain		Siles	J	38.4848	-2.78925	780
	CZE122	01C2108004	Specimen	Aegilops neglecta subsp. recta (ZHUK.) HAMM		ESP	Spain	bei Monasterio de Yuste, Sierra de Gredos, Caceres					
	DEU146	AE 585	Specimen	Aegilops neglecta Req. ex Bertol. subsp. recta		ESP	Spain	bei Molinillo nahe Bejar, Salamanca					
	DEU146	AE 1346	Specimen	Aegilops neglecta REQ. ex BERTOL.		ESP	Spain	Sdspanien: Andalusien, Sierranevada, ca. 30 km sdstlich von Granada, Lanjaron					
00:00.0	S	S07-11003	Specimen	Aegilops neglecta Req. ex Bertol.	L.	Spain	Spain	Spain, CÃ¡diz. Near Atlanterra betw		CÃ¡diz			
	FUND. BIODIVER	1062081	Unknown	Aegilops neglecta Req. & Bertol	Req. & Berto	ESP	Spain	Sierra de Aracena		H	37.1	-6.1	
	FUND. BIODIVER	1835105	Unknown	Aegilops neglecta Req. ex Bertol	Req. ex Bert	ESP	Spain	Real Valle de Alcudia		CR			
00:00.0	REDIAM-CMA	384707	Observation	Aegilops neglecta		ESP	Spain		Espiel	Co	38.1366	-5.11313	689
00:00.0	REDIAM-CMA	387615	Observation	Aegilops neglecta		ESP	Spain		Cambil	J	37.7325	-3.50589	1631
	SIVIM	Q-P02280:Aegi	Observation	Aegilops neglecta Req. ex Bertol.	Req. ex Bert	ES	Spain	Ciutadella: Cala En Blanes			39.92	3.7	10
00:00.0	REDIAM-CMA	144317	Observation	Aegilops neglecta		ESP	Spain		Santa Olalla del C	H	37.9024	-6.19228	510
00:00.0	REDIAM-CMA	161599	Observation	Aegilops neglecta		ESP	Spain		Villanueva de la R	J	38.3269	-3.8719	698
	SIVIM	T-P17445:Aegi	Observation	Aegilops neglecta Req. ex Bertol.	Req. ex Bert	ES	Spain	ChinchÃ³n			40.1	-3.46	0
00:00.0	GDAC	GDAC38246-1	Specimen	Aegilops neglecta Req. ex Bertol	Req. ex Bert	ES	Spain	AlmerÃ-a, SÂª de GÃ¡dor, sobre Ca		AL			1200
	FUND. BIODIVER	1093237	Unknown	Aegilops neglecta		ESP	Spain	Finca de Araya, Arroyo de la Luz		Cc	39.1	-6.1	
	FUND. BIODIVER	1093259	Unknown	Aegilops neglecta		ESP	Spain	Tejeda de TiÃ©tar, Castillejo		Cc	39.1	-5.1	
00:00.0	FUND. BIODIVER	1946423	Unknown	Aegilops neglecta Req. ex Bertol	Req. ex Bert	ESP	Spain	Solana del Pino, Alhorin		CR	38.1	-3.1	660
	SIVIM	S-P00111:Aegi	Observation	Aegilops neglecta Req. ex Bertol.	Req. ex Bert	ES	Spain	*			41.36	2.04	200
	SIVIM	S-P02877:Aegi	Observation	Aegilops neglecta Req. ex Bertol.	Req. ex Bert	ES	Spain	massif du Tibidabo: Ã l'ouest de Moncada,			41.45	2.16	0
	SIVIM	S-P04719:Aegi	Observation	Aegilops neglecta Req. ex Bertol.	Req. ex Bert	ES	Spain	El Burgo de Ebro, cerca del cementerio, bc			41.53	-0.84	190
	SIVIM	S-P06251:Aegi	Observation	Aegilops neglecta Req. ex Bertol.	Req. ex Bert	ES	Spain	de Linares de Mora y Cerro Brun			40.26	-0.64	0
	SIVIM	T-P27666:Aegi	Observation	Aegilops neglecta Req. ex Bertol.	Req. ex Bert	ES	Spain	PrÃ³x. Cjo. Rosa Alta, SÂª de Rute			37.3	-4.35	0
00:00.0	MGC	33847-1	Unknown	Aegilops neglecta Req.	Req.	ES	Spain	Grazalema; Casa del BÃºho		Ca	0	0	1
00:00.0	ABH	44528-1	Specimen	Aegilops neglecta Req ex Bertol.	Req ex Berto	ES	Spain	Alicante; Cala cantalares		A	38.35	-0.43	
00:00.0	SEV	101366-1	Specimen	Aegilops neglecta Req. ex Bertol.	Req. ex Bert	ES	Spain	RÃ-o NÃ©valo. Paredones de Las A		Co			1
00:00.0	GDAC	GDAC41207-1	Specimen	Aegilops neglecta Req. ex Bertol	Req. ex Bert	ES	Spain	CÃ³rdoba, SÂª Morena, prox. Arroyo		CO			740
00:00.0	REDIAM-CMA	246383	Observation	Aegilops neglecta		ESP	Spain		Cortegana	H	37.9477	-6.78153	420
00:00.0	REDIAM-CMA	268868	Observation	Aegilops neglecta		ESP	Spain		Villanueva de CÃ	Co	38.2822	-4.58957	663
	SIVIM	T-P27002:Aegi	Observation	Aegilops neglecta Req. ex Bertol.	Req. ex Bert	ES	Spain	*			41.34	-0.49	226
	SIVIM	T-P27677:Aegi	Observation	Aegilops neglecta Req. ex Bertol.	Req. ex Bert	ES	Spain	Sierra de Rute			37.3	-4.35	0
00:00.0	REDIAM-CMA	43929	Observation	Aegilops neglecta		ESP	Spain		Huelma	J	37.7126	-3.46661	1674
00:00.0	REDIAM-CMA	67727	Observation	Aegilops neglecta		ESP	Spain		Hornachuelos	Co	38.0055	-5.45579	421

00:00.0	REDIAM-CMA	75593	Observation	Aegilops neglecta		ESP	Spain		Tolox	Ma	36.6795	-4.93562	762
	SIVIM	T-P00901:Aegi	Observation	Aegilops neglecta Req. ex Bertol.	Req. ex Bert	ES	Spain	PeÃ±a de Jijona			38.46	-0.59	0
	FUND. BIODIVER	1093260	Unknown	Aegilops neglecta		ESP	Spain	Valverde de la Vera, La Vega		Cc	39.1	-5.1	
00:00.0	FUND. BIODIVER	1946424	Unknown	Aegilops neglecta Req. ex Bertol	Req. ex Bert	ESP	Spain	sierra de Alhambra		CR	38.1	-2.1	
00:00.0	REDIAM-CMA	403117	Observation	Aegilops neglecta		ESP	Spain		El Real de la Jara	Se	37.9887	-6.05924	500
00:00.0	REDIAM-CMA	413751	Observation	Aegilops neglecta		ESP	Spain		Villanueva de CÃ¡	Co	38.2941	-4.55091	732
	SIVIM	S-P00112:Aegi	Observation	Aegilops neglecta Req. ex Bertol.	Req. ex Bert	ES	Spain	*			41.36	2.04	200
	SIVIM	S-P02878:Aegi	Observation	Aegilops neglecta Req. ex Bertol.	Req. ex Bert	ES	Spain	massif du Levant: au-dessus de Sta. Colon			41.36	2.16	170
	SIVIM	S-P04738:Aegi	Observation	Aegilops neglecta Req. ex Bertol.	Req. ex Bert	ES	Spain	Retuerta de Pina, barranco al sur de la car			41.43	-0.36	280
	SIVIM	S-P06252:Aegi	Observation	Aegilops neglecta Req. ex Bertol.	Req. ex Bert	ES	Spain	de Linares de Mora y Cerro Brun			40.26	-0.64	0
00:00.0	FCO	24526-1	Specimen	Aegilops neglecta Req. ex Bertol.	Req. ex Bert	ES	Spain	Cazorla; Nava de San Pablo		J			
	FUND. BIODIVER	1093245	Unknown	Aegilops neglecta		ESP	Spain	Cerro de Aldeamoret		Cc	39.1	-6.1	
	FUND. BIODIVER	1946409	Unknown	Aegilops neglecta Req. ex Bertol	Req. ex Bert	ESP	Spain	Despenaperros		CR	38.1	-3.1	
00:00.0	FUND. BIODIVER	1946431	Unknown	Aegilops neglecta Req. ex Bertol	Req. ex Bert	ESP	Spain	Alcubillas, alrededores		CR	38.1	-2.1	830
00:00.0	REDIAM-CMA	21579	Observation	Aegilops neglecta		ESP	Spain		Torres	J	37.7376	-3.50336	1624
00:00.0	REDIAM-CMA	395335	Observation	Aegilops neglecta		ESP	Spain		Hornachuelos	Co	37.9281	-5.26431	500
00:00.0	REDIAM-CMA	404814	Observation	Aegilops neglecta		ESP	Spain		Aracena	H	37.9457	-6.57449	451
	SIVIM	S-P03041:Aegi	Observation	Aegilops neglecta Req. ex Bertol.	Req. ex Bert	ES	Spain	massif de Sant LlorenÃ§: entre le Coll d'Es			41.63	1.91	0
	SIVIM	S-P04964:Aegi	Observation	Aegilops neglecta Req. ex Bertol.	Req. ex Bert	ES	Spain	Bujaraloz, cerca de la Laguna de la Playa			41.42	-0.24	340
	SIVIM	S-P06278:Aegi	Observation	Aegilops neglecta Req. ex Bertol.	Req. ex Bert	ES	Spain	de la comarca de Linares-Valdelinares			40.26	-0.64	0
	SANT	48206	Specimen	Aegilops neglecta Req. ex Bertol.		ES	Spain	SabiÃ±Ã¡nigo. Nocito. Barranco de		Hu			
00:00.0	ABH	42146-1	Specimen	Aegilops neglecta Req ex Bertol.	Req ex Berto	ES	Spain	Alicante; Urbanova		A	38.27	-0.52	
00:00.0	BC	862086	Specimen	Aegilops neglecta Req. ex Bertol.	Req. ex Bert	ES	Spain	AlmadÃ©n; Almaden		CR	38.7443	-4.8409	
00:00.0	SALA	115073-1	Specimen	Aegilops neglecta Req. ex Bertol.	Req. ex Bert	ES	Spain	ArbancÃ³n, Barranco de la Hoz		Gu	41	-3.16	
	FUND. BIODIVER	1043768	Unknown	Aegilops neglecta Req. ex Bertol	Req. ex Bert	ESP	Spain	MÃ¡laga		Ma	36.1	-4.1	
00:00.0	FUND. BIODIVER	1835098	Unknown	Aegilops neglecta Req. ex Bertol	Req. ex Bert	ESP	Spain	Fuencaliente, Sierra Madrona, umb		CR	38.1	-4.1	780
	SIVIM	Q-P02156:Aegi	Observation	Aegilops neglecta Req. ex Bertol.	Req. ex Bert	ES	Spain	Vistabella			40.25	-0.29	1250
	SIVIM	T-P18881:Aegi	Observation	Aegilops neglecta Req. ex Bertol.	Req. ex Bert	ES	Spain	Junto a FabrÃ©gues			43.53	3.74	0
	SIVIM	T-P19983:Aegi	Observation	Aegilops neglecta Req. ex Bertol.	Req. ex Bert	ES	Spain	Almaraz			39.8	-5.68	320
00:00.0	SEV	108386-1	Specimen	Aegilops neglecta Req. ex Bertol.	Req. ex Bert	ES	Spain	Medina Sidonia. Puerto de los Reye		Ca			20
	SIVIM	T-P27048:Aegi	Observation	Aegilops neglecta Req. ex Bertol.	Req. ex Bert	ES	Spain	*			41.52	-0.6	220
	CZE122	01C2108029	Specimen	Aegilops neglecta REQ. ex BERTOL.		ESP	Spain	Rio Argon, Villanua					
00:00.0	SALA	84424-1	Specimen	Aegilops neglecta Req. ex Bertol	Req. ex Bert	ES	Spain	_; CastronuÃ±o		Va	41.39	-5.27	
00:00.0	ABH	47795-1	Specimen	Aegilops neglecta Req ex Bertol.	Req ex Berto	ES	Spain	Alcoba; RaÃ±a del Peral		CR	39.32	-4.39	
00:00.0	SALA	52037-1	Specimen	Aegilops neglecta Req. ex Bertol	Req. ex Bert	ES	Spain	_; Fresno de la Ribera		Za			
00:00.0	REDIAM-CMA	365591	Observation	Aegilops neglecta		ESP	Spain		Cuevas del Almar	Al	37.2996	-1.79985	50
	SIVIM	P-P02632:Aegi	Observation	Aegilops neglecta Req. ex Bertol.	Req. ex Bert	ES	Spain	solana del Tibidabo, sobre la Bonanova			41.36	2.04	270
	SIVIM	Q-P01995:Aegi	Observation	Aegilops neglecta Req. ex Bertol.	Req. ex Bert	ES	Spain	Pla de Baix			40.25	-0.29	1100
	SIVIM	U-P08107:Aegi	Observation	Aegilops neglecta Req. ex Bertol.	Req. ex Bert	ES	Spain	Los Cercones, LogrosÃ¡n			39.27	-5.55	0
	SIVIM	U-P10311:Aegi	Observation	Aegilops neglecta Req. ex Bertol.	Req. ex Bert	ES	Spain	entre Pueblo de Alfambra y el de Cuevas L			40.36	-1.11	0
00:00.0	UNEX	05897-1	Observation	Aegilops neglecta Req. ex Bertol.	_	ESP	Spain	Segura de LeÃ³n: Entre Segura de		Ba	38.1	-6.6	

00:00.0	GDA	GDA24226-1-2	Specimen	Aegilops neglecta Req. ex Bertol	Req. ex Bert	ES	Spain	Ciudad Real, SÂª Alhambra, en la b		CR			0
00:00.0	SEV	101361-1	Specimen	Aegilops neglecta Req. ex Bertol.	Req. ex Bert	ES	Spain	Entre Ojuelos Altos y La Cardencho		Co			1
00:00.0	FCO	27632-1	Specimen	Aegilops neglecta Req. ex Bertol.	Req. ex Bert	ES	Spain	ArbancÃ³n, Barranco de la Hoz		Gu			
	SIVIM	P-P04511:Aegi	Observation	Aegilops neglecta Req. ex Bertol.	Req. ex Bert	ES	Spain	Figuerola d'Orcau			42.07	0.94	0
00:00.0	HSS	13181	Specimen	Aegilops neglecta Req. ex Bertol	Req. ex Bert	ES	Spain	BaÃ±os de Montemayor		Cc	40.2996	-5.88265	
00:00.0	SALA	68750-1	Specimen	Aegilops neglecta Req. ex Bertol	Req. ex Bert	ES	Spain	_; Navalmoral de la Mata		Cc	39.88	-5.55	
	IPK	AE 590	Living	Aegilops neglecta Req. ex Bertol. subsp. recta (Zhuk.			Spain	Finca La Cigue?ela, ostlich Aracena, Huelv			37.8919	-6.52083	
	FUND. BIODIVER	1093246	Unknown	Aegilops neglecta		ESP	Spain	Dehesa de Valdeobispo, Almaraz		Cc	39.1	-6.1	
	FUND. BIODIVER	1946410	Unknown	Aegilops neglecta Req. ex Bertol	Req. ex Bert	ESP	Spain	Intercalacion calizo-devonica de Aln		CR	38.1	-4.1	
00:00.0	FUND. BIODIVER	1946432	Unknown	Aegilops neglecta Req. ex Bertol	Req. ex Bert	ESP	Spain	Alcoba, Cabaneros, Guarreras		CR	39.1	-4.1	
	SIVIM	S-P05024:Aegi	Observation	Aegilops neglecta Req. ex Bertol.	Req. ex Bert	ES	Spain	Flix (La Ribera, CataluÃ±a occidental), orill			41.16	0.49	0
	SIVIM	S-P05048:Aegi	Observation	Aegilops neglecta Req. ex Bertol.	Req. ex Bert	ES	Spain	Zuera, llanura del GÃ¡llego			41.8	-0.83	270
	SIVIM	S-P06279:Aegi	Observation	Aegilops neglecta Req. ex Bertol.	Req. ex Bert	ES	Spain	de la comarca de Linares-Valdelinares			40.26	-0.64	0
00:00.0	MA	729772-1	Specimen	Aegilops neglecta Req. ex Bertol	Req. ex Bert	ES	Spain	Aldea del Rey, Cerro de la Higuera.		CR	38	-3	
	ESP004	NC043472	Specimen	Aegilops neglecta REQ. ex BERTOL.		ESP	Spain	Minas de Aldeamoret, Caceres, province of			39.45	-6.38333	451
	SIVIM	T-P15717:Aegi	Observation	Aegilops neglecta Req. ex Bertol.	Req. ex Bert	ES	Spain	Base suroriental de Sierra de Aguas			36.76	-4.79	0
00:00.0	FUND. BIODIVER	1835110	Unknown	Aegilops neglecta Req. ex Bertol	Req. ex Bert	ESP	Spain	Viso del Marques, Camino Real de		CR	38.1	-3.1	840
00:00.0	REDIAM-CMA	386567	Observation	Aegilops neglecta		ESP	Spain		Torres	J	37.7807	-3.49427	1146
00:00.0	REDIAM-CMA	393960	Observation	Aegilops neglecta		ESP	Spain		La Puerta de Seg	J	38.3683	-2.85369	597
	SIVIM	Q-P05359:Aegi	Observation	Aegilops neglecta Req. ex Bertol.	Req. ex Bert	ES	Spain	Roses, al Puig de la Garriga, prop del parc			42.27	3.12	0
	SIVIM	U-P13958:Aegi	Observation	Aegilops neglecta Req. ex Bertol.	Req. ex Bert	ES	Spain	Robledo			38.75	-2.53	1010
00:00.0	SEV	101362-1	Specimen	Aegilops neglecta Req. ex Bertol.	Req. ex Bert	ES	Spain	Entre PeÃ±arroya y El Hoyo, a la al		Co			1
00:00.0	REDIAM-CMA	192061	Observation	Aegilops neglecta		ESP	Spain		El Pedroso	Se	37.869	-5.77772	418
	SIVIM	T-P19968:Aegi	Observation	Aegilops neglecta Req. ex Bertol.	Req. ex Bert	ES	Spain	Finca de Valdelasyeguas (Aliseda)			39.36	-6.79	0
	SIVIM	T-P20218:Aegi	Observation	Aegilops neglecta Req. ex Bertol.	Req. ex Bert	ES	Spain	Entre Martos y Alcaudete			37.58	-4.13	0
	DEU146	AE 1194	Specimen	Aegilops neglecta REQ. ex BERTOL.		ESP	Spain	Pablo Romaso, Strae Sevilla - Huelva, 4 km von Sanlcar la Mayo					40
00:00.0	REDIAM-CMA	329001	Observation	Aegilops neglecta		ESP	Spain		Espiel	Co	38.1365	-5.04188	615
00:00.0	REDIAM-CMA	379078	Observation	Aegilops neglecta		ESP	Spain		Vilches	J	38.121	-3.46067	352
	SIVIM	P-P04926:Aegi	Observation	Aegilops neglecta Req. ex Bertol.	Req. ex Bert	ES	Spain	Castiliscar, talud junto a la carretera de So			42.34	-1.3	0
	FUND. BIODIVER	76096	Unknown	Aegilops neglecta Req. Ex Berto	Req. Ex Bert	ESP	Spain	El Pego		Za	41.1	-5.1	
	FUND. BIODIVER	78795	Unknown	Aegilops neglecta Req. ex Portol	Req. ex Port	ESP	Spain	Portillo		Va	41.1	-4.1	
00:00.0	REDIAM-CMA	82277	Observation	Aegilops neglecta		ESP	Spain		Ronda	Ma	36.6878	-5.05694	1300
00:00.0	REDIAM-CMA	90925	Observation	Aegilops neglecta		ESP	Spain		BailÃ©n	J	38.1314	-3.8234	341
	FUND. BIODIVER	1674410	Unknown	Aegilops triuncialis		ESP	Spain	Talavera, carretera a Segurilla		To	39.1	-4.1	440
00:00.0	REDIAM-CMA	268828	Observation	Aegilops triuncialis		ESP	Spain		Montoro	Co	38.2302	-4.54388	599
00:00.0	REDIAM-CMA	277435	Observation	Aegilops triuncialis		ESP	Spain		Dos Torres	Co	38.4976	-4.89309	563
00:00.0	REDIAM-CMA	282022	Observation	Aegilops triuncialis		ESP	Spain		Hinojosa del Duq	Co	38.4674	-5.10081	575
	SIVIM	T-P27673:Aegi	Observation	Aegilops triuncialis L.	L.	ES	Spain	Prados de la Nava, SÂª de Cabra			37.48	-4.47	1020
	SIVIM	T-P28807:Aegi	Observation	Aegilops triuncialis L.	L.	ES	Spain	Riberos del Tajo, Serradilla			39.71	-6.2	260
	SIVIM	T-P29372:Aegi	Observation	Aegilops triuncialis L.	L.	ES	Spain	Sierra de GÃ¡dor			36.86	-2.55	1800
	SIVIM	T-P30060:Aegi	Observation	Aegilops triuncialis L.	L.	ES	Spain	Los Callejones, Cofrentes, Valencia			39.19	-1.26	0

	SIVIM	U-P03853:Aegi	Observation	Aegilops triuncialis L.	L.	ES	Spain	Ãvila: Navamorales			40.44	-5.47	1010
	RUS001	VIR100602145	Specimen	Aegilops triuncialis L.		ESP	Spain						
	RUS001	VIR100602311	Specimen	Aegilops triuncialis L.		ESP	Spain						
	FUND. BIODIVER	1074126	Unknown	Aegilops triuncialis		ESP	Spain	DoÃ±a Mencia		Co	37.1	-4.1	
	FUND. BIODIVER	1085278	Unknown	Aegilops triuncialis		ESP	Spain	Villaviciosa de OdÃ³n		M	40.1	-3.1	
	FUND. BIODIVER	1946447	Unknown	Aegilops triuncialis L.	L.	ESP	Spain	Provincia de Ciudad Real		CR			
00:00.0	REDIAM-CMA	403182	Observation	Aegilops triuncialis		ESP	Spain		Villanueva de la R	J	38.3366	-3.86171	694
00:00.0	REDIAM-CMA	414209	Observation	Aegilops triuncialis		ESP	Spain		CardeÃ±a	Co	38.2393	-4.54289	614
00:00.0	REDIAM-CMA	421466	Observation	Aegilops triuncialis		ESP	Spain		Felix	Al	36.9051	-2.72243	1399
	SIVIM	R-P08446:Aegi	Observation	Aegilops triuncialis L.	L.	ES	Spain	El Calabrial			36.86	-2.77	1400
00:00.0	HSS	13386	Specimen	Aegilops triuncialis L.	L.	ES	Spain	Zafra, Matanegra		Ba	38.4149	-6.42321	
00:00.0	MA	718177-1	Specimen	Aegilops triuncialis L.	L.	ES	Spain	SangÃ¼esa, RÃo AragÃ³n.		Na	42	-1	
	IPK	32152	Living	Aegilops triuncialis L. subsp. triuncialis var. flav		ESP	Spain	Huelma Andalusien					
	RUS001	VIR100602175	Specimen	Aegilops triuncialis L.		ESP	Spain						
	RUS001	VIR100602149	Specimen	Aegilops triuncialis L.		ESP	Spain						
	FUND. BIODIVER	1035682	Unknown	Aegilops triuncialis		ESP	Spain	Sierra de Aguas, Carratraca		Ma	36.1	-4.1	500
	FUND. BIODIVER	1043774	Unknown	Aegilops triuncialis L.	L.	ESP	Spain	CoÃn		Ma	36.1	-4.1	
	FUND. BIODIVER	1062080	Unknown	Aegilops triuncialis L.	L.	ESP	Spain	Sierra de Aracena		H	37.1	-6.1	
00:00.0	FUND. BIODIVER	1835125	Unknown	Aegilops triuncialis L.	L.	ESP	Spain	Malagon-Los Quiles, olivos		CR	39.1	-3.1	
00:00.0	FUND. BIODIVER	1835146	Unknown	Aegilops triuncialis L.	L.	ESP	Spain	Alhambra, saladar en arroyo de Los		CR	38.1	-2.1	
00:00.0	REDIAM-CMA	384672	Observation	Aegilops triuncialis		ESP	Spain		Cambil	J	37.6923	-3.51138	1052
	SIVIM	U-P13539:Aegi	Observation	Aegilops triuncialis L.	L.	ES	Spain	BraganÃ§a, Sta Maria, Ã¡ saÃda para Fra			41.8	-6.83	0
00:00.0	COFC	36336-1	Specimen	Aegilops triuncialis L.	L.	ES	Spain	Puente Genil; Sierra Gorda		Co			1
00:00.0	SALA	30600-1	Specimen	Aegilops triuncialis L.	L.	ES	Spain	_; CaÃ±izal		Za			
00:00.0	W	42841	Unknown	Aegilops triuncialis L.		ESP	Spain	Prov. Mursia: Sierra de la Gresta des Gallo: ca. 1 km S von der F					420
00:00.0	UNEX	10352-1	Observation	Aegilops triuncialis L.	_	ESP	Spain	Olivenza: Embalse de Piedra Aguda		Ba	38.7	-7.2	
00:00.0	GDA	GDA13281-1	Specimen	Aegilops triuncialis L.	L.	ES	Spain	MÃ¡laga, CoÃn, La AlbuquerÃa.		MA			0
00:00.0	SALA	42164-1	Specimen	Aegilops triuncialis L.	L.	ES	Spain	_; Entre Torrecampo y San Benito		Co			
00:00.0	SALA	52030-1	Specimen	Aegilops triuncialis L.	L.	ES	Spain	_; Granja de la Moreruela		Za			
00:00.0	SEV	27370-1	Specimen	Aegilops triuncialis L.	L.	ES	Spain	El Garrobo		Se			1
00:00.0	MA	627503-1	Specimen	Aegilops triuncialis L.	L.	ES	Spain	Piedrabuena, cunetas de la carreter		CR	39	-4	
00:00.0	HSS	14720	Specimen	Aegilops triuncialis L.	L.	ES	Spain	La Parra		Ba	38.5098	-6.64918	
00:00.0	COFC	41303-1	Specimen	Aegilops triuncialis L.	L.	ES	Spain	Lucena; Km-63 de la carretera entre		Co	37	-4	1
	FUND. BIODIVER	1370914	Unknown	Aegilops triuncialis L.	L.	ESP	Spain	El Molar		M	40.1	-3.1	
	FUND. BIODIVER	1377177	Unknown	Aegilops triuncialis L.	L.	ESP	Spain	Vilardesilva		Or	42.1	-6.1	450
	FUND. BIODIVER	1463957	Unknown	Aegilops triuncialis L.	L.	ESP	Spain	Gerb		L	41.1	0.1	260
	FUND. BIODIVER	913849	Unknown	Aegilops triuncialis L.	L.	ESP	Spain	Massif de St. LlorenÃ§, Valles		B	41.1	1.1	
00:00.0	REDIAM-CMA	162842	Observation	Aegilops triuncialis		ESP	Spain		AndÃºjar	J	38.2947	-3.89919	500
	SIVIM	T-P16904:Aegi	Observation	Aegilops triuncialis L.	L.	ES	Spain	Tres Cantos, Universidad AutÃ³noma			40.55	-3.82	0
00:00.0	SALA	36021-1	Specimen	Aegilops triuncialis L.	L.	ES	Spain	_; Montemayor del RÃo		Sa			
	SIVIM	P-P08867:Aegi	Observation	Aegilops triuncialis L.	L.	ES	Spain	Mas de la Caramassa; H. St. Joan			40.88	0.26	0

	SIVIM	U-P09949:Aegi	Observation	Aegilops triuncialis L.	L.	ES	Spain	Cuneta camino Villahermosa			38.66	-2.88	0
	BDBCV-General	276488	Observation	Aegilops triuncialis		ESPA	Spain	Bolbaite	La Canal de Nava	Valenc	39.0517	-0.74664	
00:00.0	MGC	55147-1	Unknown	Aegilops triuncialis L.	L.	ES	Spain	Sierra Palmitera. Carretera San Ped		Ma	0	0	1
	FUND. BIODIVER	99838	Unknown	Aegilops triuncialis L.	L.	ESP	Spain	Perilla de Castro, Puente de la Estre		Za	41.1	-5.1	
00:00.0	SEV	49881-1	Specimen	Aegilops triuncialis L.	L.	ES	Spain	Entre Valdezufre y Aracena		H			1
00:00.0	REDIAM-CMA	235648	Observation	Aegilops triuncialis		ESP	Spain		El Real de la Jara	Se	37.9218	-5.97063	402
	SIVIM	T-P20229:Aegi	Observation	Aegilops triuncialis L.	L.	ES	Spain	Alcaudete, lÃmite con la provincia de CÃ³r			37.58	-4.13	0
	SIVIM	T-P26221:Aegi	Observation	Aegilops triuncialis L.	L.	ES	Spain	Arenales de Las Virtudes, Villena			38.56	-0.93	0
	ESP004	NC027459	Specimen	Aegilops triuncialis L.		ESP	Spain	Riodeva, province of Teruel			40.1167	-1.13333	967
	ESP004	NC022340	Specimen	Aegilops triuncialis L.		ESP	Spain	Fuente-Agria, Espiel, province of Cordoba					
	W	43303	Unknown	Aegilops triuncialis L.		ESP	Spain	An dÃ¼rren, trockenen Stellen der Sierra de la Sagra bei der kle					1500
	FUND. BIODIVER	74649	Unknown	Aegilops triuncialis L.	L.	ESP	Spain	Montemayor del rÃo		Sa	40.1	-5.1	
00:00.0	REDIAM-CMA	80344	Observation	Aegilops triuncialis		ESP	Spain		El Castillo de las	Se	37.6553	-6.22918	207
00:00.0	REDIAM-CMA	94330	Observation	Aegilops triuncialis		ESP	Spain		BaÃ±os de la Enc	J	38.28	-3.80298	600
	SIVIM	T-P13210:Aegi	Observation	Aegilops triuncialis L.	L.	ES	Spain	UjuÃ©, hacia Gallipienzo			43.25	-1.52	0
	SIVIM	T-P15731:Aegi	Observation	Aegilops triuncialis L.	L.	ES	Spain	zonas elevadas de la Sierra de Aguas			36.85	-4.79	0
	FUND. BIODIVER	1551179	Unknown	Aegilops triuncialis		ESP	Spain	boreali rara (Gallec. pr. el Ferrol)					
	FUND. BIODIVER	983878	Unknown	Aegilops triuncialis L.	L.	ESP	Spain	MaÃ±eru		Na	42.1	-1.1	
00:00.0	REDIAM-CMA	194389	Observation	Aegilops triuncialis		ESP	Spain		Quesada	J	37.8165	-3.09819	716
00:00.0	REDIAM-CMA	216358	Observation	Aegilops triuncialis		ESP	Spain		Gor	Gr	37.3964	-2.94044	1283
00:00.0	REDIAM-CMA	239916	Observation	Aegilops triuncialis		ESP	Spain		El Real de la Jara	Se	37.9406	-6.04852	685
	SIVIM	T-P17783:Aegi	Observation	Aegilops triuncialis L.	L.	ES	Spain	Vaciamadrid			40.28	-3.58	0
00:00.0	SALA	46237-1	Specimen	Aegilops triuncialis L.	L.	ES	Spain	_; Cantalapiedra, La Carolina		Sa			
00:00.0	SALA	52029-1	Specimen	Aegilops triuncialis L.	L.	ES	Spain	_; Abezames		Za			
	SIVIM	T-P18897:Aegi	Observation	Aegilops triuncialis L.	L.	ES	Spain	AlcalÃ¡ de Henares, finca La Clota			40.46	-3.47	0
	SIVIM	T-P21028:Aegi	Observation	Aegilops triuncialis L.	L.	ES	Spain	El Peralejo, Los Villares			37.67	-3.9	1200
00:00.0	MA	636845-1	Specimen	Aegilops triuncialis L.	L.	ES	Spain	Santiuste de Pedraza, de Torre Val		Sg	41	-3	
	JBS	460-1	Specimen	Aegilops triuncialis L.	L.	ES	Spain	SÃ³ller; Ctra. a DeiÃ		Mll	39.77	2.69	1
00:00.0	COFC	46910-1	Specimen	Aegilops triuncialis L.	L.	ES	Spain	Posadas; ctra de Villaviciosa de CÃ		Co			1
	FUND. BIODIVER	1012905	Unknown	Aegilops triuncialis L.	L.	ESP	Spain	Sorbas		Al	36.1	-1.1	
	FUND. BIODIVER	102487	Unknown	Aegilops triuncialis L.	L.	ESP	Spain	Salobral		Av	40.1	-4.1	
00:00.0	FUND. BIODIVER	1714167	Unknown	Aegilops triuncialis L.	L.	ESP	Spain	Eivissa, Sant Carles, prop de la font		PM	38.1	1.1	90
	FUND. BIODIVER	1762692	Unknown	Aegilops triuncialis		ESP	Spain	Morron de Villamayor		CR	38.1	-3.1	
00:00.0	BC	70830	Specimen	Aegilops triuncialis L.	L.	ES	Spain	De VÃ©lez a Canillas de Aceituno		Ma			
00:00.0	BC	70837	Specimen	Triticum triunciale (L.) Raspail	(L.) Raspail	ES	Spain	Lanteira; in Sierra Nevada: Lanteria		Gr	37.18	-3.17	1400
	BC	91137	Specimen	Aegilops triuncialis L.	L.	ES	Spain	Velez Rubio; Velez-Rubio		Al	37.63	-2.04	500
00:00.0	MGC	62203-1	Unknown	Aegilops triuncialis L.	L.	ES	Spain	Lora del RÃo; Al Norte de Lora del		Se	37.681	-5.557	120
00:00.0	ABH	52804-1	Specimen	Aegilops triuncialis L.	L.	ES	Spain	Estepona; carretera general de Ger		Ma	36.47	-5.18	
00:00.0	REDIAM-CMA	246731	Observation	Aegilops triuncialis		ESP	Spain		La Nava	H	37.9601	-6.79343	600
00:00.0	REDIAM-CMA	277979	Observation	Aegilops triuncialis		ESP	Spain		AlanÃs	Se	38.1061	-5.59518	385
00:00.0	REDIAM-CMA	273259	Observation	Aegilops triuncialis		ESP	Spain		Villanueva de CÃ	Co	38.3174	-4.53847	693

	SIVIM	T-P27677:Aegi	Observation	Aegilops triuncialis L.	L.	ES	Spain	Sierra de Rute			37.3	-4.35	0
	SIVIM	T-P28810:Aegi	Observation	Aegilops triuncialis L.	L.	ES	Spain	Castillo de Mirabel			39.8	-6.31	0
	SIVIM	T-P29379:Aegi	Observation	Aegilops triuncialis L.	L.	ES	Spain	Sierra de GÃ¡dor			36.86	-2.55	1580
	SIVIM	T-P30063:Aegi	Observation	Aegilops triuncialis L.	L.	ES	Spain	Pr. Las Salinas, Casas de Ves, Albacete			39.28	-1.37	0
	SIVIM	U-P06644:Aegi	Observation	Aegilops triuncialis L.	L.	ES	Spain	Zimbreira			37.22	-8.32	180
	SIVIM	T-P27682:Aegi	Observation	Aegilops triuncialis L.	L.	ES	Spain	La Nava, SÂª de Cabra			37.48	-4.47	1020
	SIVIM	T-P28872:Aegi	Observation	Aegilops triuncialis L.	L.	ES	Spain	El Ejido, Saucedilla			39.8	-5.68	280
	SIVIM	T-P29476:Aegi	Observation	Aegilops triuncialis L.	L.	ES	Spain	Diferentes localidades de la Sierra del Agu			36.85	-4.79	0
	SIVIM	T-P30066:Aegi	Observation	Aegilops triuncialis L.	L.	ES	Spain	Pr. La Zorrera, Jalance, Valencia			39.1	-1.14	0
	SIVIM	U-P06647:Aegi	Observation	Aegilops triuncialis L.	L.	ES	Spain	Rocha da Pena			37.22	-8.21	250
00:00.0	SEV	98919-1	Specimen	Aegilops triuncialis L.	L.	ES	Spain	Entre MorÃ³n y VillamartÃn		Se			1
00:00.0	SEV	98944-1	Specimen	Aegilops triuncialis L.	L.	ES	Spain	Entre Castilblanco y El Pedroso de		Se			280
1876-06-0	W	43351	Unknown	Aegilops triuncialis L.		ESP	Spain	Hispania: In collibus prope El Escorial. In itinere hispanico-lusitanico.					
	FUND. BIODIVER	1376738	Unknown	Aegilops triuncialis L.	L.	ESP	Spain	Sierra de Baza, Parque Natural		Gr	37.1	-2.1	
	FUND. BIODIVER	1475537	Unknown	Aegilops triuncialis L.	L.	ESP	Spain	Sierra del Ricote		Mu	37.1	-1.1	
00:00.0	REDIAM-CMA	161598	Observation	Aegilops triuncialis		ESP	Spain		Villanueva de la F	J	38.3268	-3.87197	698
00:00.0	REDIAM-CMA	173080	Observation	Aegilops triuncialis		ESP	Spain		AlanÃs	Se	38.0138	-5.58576	668
00:00.0	HSS	10178	Specimen	Aegilops triuncialis L.	L.	ES	Spain	Bohonal de Ibor, alrededores		Cc	39.7678	-5.51012	
	SIVIM	T-P16898:Aegi	Observation	Aegilops triuncialis L.	L.	ES	Spain	Alcobendas			40.46	-3.7	0
	SIVIM	T-P16925:Aegi	Observation	Aegilops triuncialis L.	L.	ES	Spain	CeclavÃn			39.81	-6.78	0
00:00.0	SALA	52031-1	Specimen	Aegilops triuncialis L.	L.	ES	Spain	_; Algodre		Za			
00:00.0	SALA	85590-1	Specimen	Aegilops triuncialis L.	L.	ES	Spain	_; Aldeanueva de Figueroa		Sa			
	FCO	4045-1	Specimen	Aegilops triuncialis L.	L.	ES	Spain	Valdemoro		M			
00:00.0	COA	41144-1	Specimen	Aegilops triuncialis L.	L.	ES	Spain	Cruce carretera Montoro, CardeÃ±a		Co	38.21	-4.37	
00:00.0	ABH	1038-1	Specimen	Aegilops triuncialis L.	L.	ES	Spain	Alcoleja; SÂª Aitana, prox. base mili		A	38.66	-0.32	
00:00.0	BC	646876	Specimen	Aegilops triuncialis L.	L.	ES	Spain	Argentona; Argentona, riera de Cire		B	41.5	2.34	
	CZE122	01C2107126	Specimen	Aegilops triuncialis subsp. triuncialis var. flaves		ESP	Spain	Huebma (Andalousie), Spanien					
	DEU146	AE 631	Specimen	Aegilops triuncialis subsp. triuncialis var. flaves		ESP	Spain	Huebma (Andalousie)					
00:00.0	SALA	10064-1	Specimen	Aegilops triuncialis L.	L.	ES	Spain	_; La Bouza		Sa			
00:00.0	SEV	53883-1	Specimen	Aegilops triuncialis L.	L.	ES	Spain	El Escorial. En talud del ferrocarril, (		M			1
00:00.0	SEV	5991-1	Specimen	Aegilops triuncialis L.	L.	ES	Spain	Madrid, Ciudad Universitaria		M			1
00:00.0	SEV	67812-1	Specimen	Aegilops triuncialis L.	L.	ES	Spain	Sierra Nevada. Subida al Veleta		Gr			2000
00:00.0	SEV	30540-1	Specimen	Aegilops triuncialis L.	L.	ES	Spain	Entre MorÃ³n y El Saucejo, arroyo S		Se			1
00:00.0	GDA	GDA23166-1-1	Specimen	Aegilops triuncialis L.	L.	ES	Spain	Madrid, S. MartÃn de la Vega, cerr		M			0
00:00.0	SALA	8161-1	Specimen	Aegilops triuncialis L.	L.	ES	Spain	_; Cabrerizos		Sa			
	BDBCV-General	74119	Observation	Aegilops triuncialis		ESPA	Spain	Villamalur	El Alto Mijares	Castel	39.9446	-0.36651	
00:00.0	COFC	12023-1	Specimen	Aegilops triuncialis L.	L.	ES	Spain	IznÃƒÂ¡jar; loma del SantÃƒÂsimo		Co	37	-4	1
00:00.0	COFC	16304-1	Specimen	Aegilops triuncialis L.	L.	ES	Spain	Santa Eufemia; carretera a la estac		Co	38	-4	1
00:00.0	MA	569168-1	Specimen	Aegilops triuncialis L.	L.	ES	Spain	Valle del Tabladillo		Sg			
	RUS001	VIR100602133	Specimen	Aegilops triuncialis L.		ESP	Spain						
	FUND. BIODIVER	80853	Unknown	Aegilops triuncialis L.	L.	ESP	Spain	SepÃºlveda, Villar de SobrepeÃ±a		Sg	41.1	-3.1	

00:00.0	UNEX	10357-1	Observation	Aegilops triuncialis L.	_	ESP	Spain	Los Santos de Maimona: Cerro San		Ba	38.5	-6.5	
00:00.0	COFC	41141-1	Specimen	Aegilops triuncialis L.	L.	ES	Spain	Puente Genil; rÃƒÂ-o Genil; presa C		Co	37	-4	1
00:00.0	BC	601435	Specimen	Aegilops triuncialis L.	L.	ES	Spain	Prades; Muntanyes de Prades: Plan		T	41.31	1.03	950
00:00.0	BC	601439	Specimen	Aegilops triuncialis L.	L.	ES	Spain	la FebrÃ³; Muntanyes de Prades: Ve		T	41.31	1.03	825
00:00.0	BC	646879	Specimen	Aegilops triuncialis L.	L.	ES	Spain	Argentona; Argentona, final del torre		B	41.5	2.34	
	FUND. BIODIVER	1109627	Unknown	Aegilops triuncialis L.	L.	ESP	Spain	Aldeanueva de Figueroa		Sa	40.1	-5.1	
00:00.0	FUND. BIODIVER	1946438	Unknown	Aegilops triuncialis L.	L.	ESP	Spain	Herencia, de Herencia a Villarta de		CR	39.1	-3.1	700
00:00.0	REDIAM-CMA	79740	Observation	Aegilops triuncialis		ESP	Spain		Villalba del Alcor	H	37.4538	-6.50644	108
00:00.0	REDIAM-CMA	99453	Observation	Aegilops triuncialis		ESP	Spain		AlmadÃ©n de la F	Se	37.8524	-6.0056	278
00:00.0	REDIAM-CMA	23879	Observation	Aegilops triuncialis		ESP	Spain		Cambil	J	37.6922	-3.51118	1052
00:00.0	REDIAM-CMA	415549	Observation	Aegilops triuncialis		ESP	Spain		Cazorla	J	37.8221	-2.85846	1400
	SIVIM	T-P11869:Aegi	Observation	Aegilops triuncialis L.	L.	ES	Spain	Valverde Enrique			42.24	-5.3	0
00:00.0	GDA	GDA13281-1-1	Specimen	Aegilops triuncialis L.	L.	ES	Spain	MÃ¡laga, CoÃ-n, La AlbuquerÃ-a.		MA			0
	FUND. BIODIVER	76094	Unknown	Aegilops triuncialis L.	L.	ESP	Spain	CaÃ±izal		Za	41.1	-5.1	
00:00.0	BC	601437	Specimen	Aegilops triuncialis L.	L.	ES	Spain	VimbodÃ-; Muntanyes de Prades: V		T	41.4	1.03	525
	SIVIM	R-P10341:Aegi	Observation	Aegilops triuncialis L.	L.	ES	Spain	Casas del Corchadillo , Jerez de la Fronter			36.47	-5.67	0
	SIVIM	S-P04337:Aegi	Observation	Aegilops triuncialis L.	L.	ES	Spain	pedregales del Congost, cerca de Les Fran			41.54	2.28	190
00:00.0	COFC	41146-1	Specimen	Aegilops triuncialis L.	L.	ES	Spain	Cabra; arroyo Galindo		Co	37	-4	1
00:00.0	REDIAM-CMA	261706	Observation	Aegilops triuncialis		ESP	Spain		Villanueva de CÃ	Co	38.3027	-4.64304	686
00:00.0	REDIAM-CMA	273465	Observation	Aegilops triuncialis		ESP	Spain		Villanueva de CÃ	Co	38.3388	-4.67068	687
00:00.0	REDIAM-CMA	278414	Observation	Aegilops triuncialis		ESP	Spain		AlanÃ-s	Se	38.0833	-5.59706	500
00:00.0	REDIAM-CMA	95163	Observation	Aegilops triuncialis		ESP	Spain		Hornachuelos	Co	37.9694	-5.40714	547
	SIVIM	T-P28874:Aegi	Observation	Aegilops triuncialis L.	L.	ES	Spain	Puente de Corrinches, Romangordo			39.71	-5.79	200
	SIVIM	T-P29478:Aegi	Observation	Aegilops triuncialis L.	L.	ES	Spain	Diferentes localidades de la Sierra del Agu			36.85	-4.79	350
	SIVIM	T-P30079:Aegi	Observation	Aegilops triuncialis L.	L.	ES	Spain	Barrio del Santuario, Villa de Ves, Albacete			39.19	-1.26	0
	SIVIM	U-P06649:Aegi	Observation	Aegilops triuncialis L.	L.	ES	Spain	Rib. da Quinta do Freixo			37.22	-8.21	2400
	SIVIM	T-P15738:Aegi	Observation	Aegilops triuncialis L.	L.	ES	Spain	Sierra de Aguas			36.85	-4.79	0
	ESP004	NC043489	Specimen	Aegilops triuncialis L.		ESP	Spain	Duraton, Sepulveda, province of Segovia			41.2833	-3.68333	943
00:00.0	SALA	40759-1	Specimen	Aegilops triuncialis L.	L.	ES	Spain	_; Sacramenia		Sg			
00:00.0	SALA	42452-1	Specimen	Aegilops triuncialis L.	L.	ES	Spain	_; Encinas de Esgueva, Otero		Va			
	FUND. BIODIVER	78798	Unknown	Aegilops triuncialis L.	L.	ESP	Spain	Cogeces de Ã•scar		Va	41.1	-4.1	
00:00.0	BC	627484	Specimen	Aegilops triuncialis L.	L.	ES	Spain	els Guiamets; Barranc de les Maleie		T	41.13	0.8	120
00:00.0	REDIAM-CMA	91372	Observation	Aegilops triuncialis		ESP	Spain		Ronda	Ma	36.6813	-5.07368	1100
00:00.0	REDIAM-CMA	117922	Observation	Aegilops triuncialis		ESP	Spain		Zufre	H	37.8212	-6.32032	301
	RUS001	VIR100602173	Specimen	Aegilops triuncialis L.		ESP	Spain						
00:00.0	FCO	6559-1	Specimen	Aegilops triuncialis L.	L.	ES	Spain	Aranjuez; OntÃ-gola		M			
	SIVIM	T-P10393:Aegi	Observation	Aegilops triuncialis L.	L.	ES	Spain	Entre Los Pilones y Cerro Alto. Trocha de l			36.66	-5.01	1720
	SIVIM	T-P11407:Aegi	Observation	Aegilops triuncialis L.	L.	ES	Spain	Tragacete			40.28	-1.82	1240
	FUND. BIODIVER	1043776	Unknown	Aegilops triuncialis L.	L.	ESP	Spain	MÃ¡laga		Ma	36.1	-4.1	
	FUND. BIODIVER	1065853	Unknown	Aegilops triuncialis L.	L.	ESP	Spain	Sierra de Aracena		H	37.1	-6.1	
	FUND. BIODIVER	1072126	Unknown	Aegilops triuncialis L.	L.	ESP	Spain	Arroyo del Salado, cerca de MorÃ³n		Se	36.1	-5.1	

00:00.0	FUND. BIODIVER	1835127	Unknown	Aegilops triuncialis L.	L.	ESP	Spain	Navalpino, rio Valdehornos		CR	39.1	-4.1	540
	FUND. BIODIVER	1835148	Unknown	Aegilops triuncialis L.	L.	ESP	Spain	Almuradiel-Venta de Cardenas		CR	38.1	-3.1	
00:00.0	REDIAM-CMA	386469	Observation	Aegilops triuncialis		ESP	Spain		Torres	J	37.7435	-3.51164	1400
	SIVIM	Q-P09214:Aegi	Observation	Aegilops triuncialis L.	L.	ES	Spain	LÃmites Evora			38.57	-7.96	280
00:00.0	HUAL	5974-1	Specimen	Aegilops triuncialis L.	L.	ES	Spain	SÂª de GÃ¡dor		Al	36.934	-2.905	
	SANT	2110	Specimen	Aegilops triuncialis L.		ES	Spain	Cerro Negro		M			
00:00.0	SEV	98924-1	Specimen	Aegilops triuncialis L.	L.	ES	Spain	PeÃ±Ã³n de AlgÃ¡mitas		Se			1
00:00.0	SEV	99064-1	Specimen	Aegilops triuncialis L.	L.	ES	Spain	Above Los Barrios		Ca			170
00:00.0	COA	41189-1	Specimen	Aegilops triuncialis L.	L.	ES	Spain	Km 4 de BelalcÃ¡zar a Hinojosa		Co	38.47	-5.18	
	COA	41222-1	Specimen	Aegilops triuncialis L.	L.	ES	Spain	Navalquejigo		M	40.55	-4.18	
00:00.0	COFC	46902-1	Specimen	Aegilops triuncialis L.	L.	ES	Spain	arroyo Benajarate		Co			1
00:00.0	FUND. BIODIVER	1774963	Unknown	Aegilops triuncialis L.	L.	ESP	Spain	Almunia de San Juan, Ariestolas, rib		Hu	41.1	-0.9	265
	RUS001	VIR100602161	Specimen	Aegilops triuncialis L.		ESP	Spain						
00:00.0	MA	772818-1	Specimen	Aegilops triuncialis L.	L.	ES	Spain	Puente Genil; Sierra Gorda. Margen		Co			
	BC	92064	Specimen	Aegilops triuncialis L.	L.	ES	Spain	Zamora; Zamora		Za	41.48	-5.69	
	FUND. BIODIVER	904087	Unknown	Aegilops triuncialis L.	L.	ESP	Spain	Entre Matadepera y Tarrasa		B	41.1	1.1	
	FUND. BIODIVER	918141	Unknown	Aegilops triuncialis L.	L.	ESP	Spain	Plana de Vich		B	41.1	2.1	
	IPK	32168	Living	Aegilops triuncialis L. subsp. triuncialis		ESP	Spain	Guara					
	IPK	32260	Living	Aegilops triuncialis L. subsp. triuncialis var. co		ESP	Spain	Fuencaliente (Soria)					
00:00.0	FUND. BIODIVER	1835138	Unknown	Aegilops triuncialis L.	L.	ESP	Spain	San Lorenzo de Calatrava, finca Pe		CR	38.1	-3.1	800
00:00.0	COFC	28803-1	Specimen	Aegilops triuncialis L.	L.	ES	Spain	Andujar; parque natural: coto 'Sellad		J			1
	SIVIM	U-P09957:Aegi	Observation	Aegilops triuncialis L.	L.	ES	Spain	Cuneta cerca de Casas Blancas			38.84	-3	0
00:00.0	REDIAM-CMA	144123	Observation	Aegilops triuncialis		ESP	Spain		Santa Olalla del C	H	37.9018	-6.19082	538
00:00.0	REDIAM-CMA	185111	Observation	Aegilops triuncialis		ESP	Spain		Cala	H	37.973	-6.3392	675
00:00.0	REDIAM-CMA	391039	Observation	Aegilops triuncialis		ESP	Spain		Zufre	H	37.803	-6.47089	517
00:00.0	REDIAM-CMA	394625	Observation	Aegilops triuncialis		ESP	Spain		CardeÃ±a	Co	38.2463	-4.28951	740
00:00.0	COFC	50174-1	Specimen	Aegilops triuncialis L.	L.	ES	Spain	Valle del Guadiato; desembocadura		Co			1
	RUS001	VIR100602136	Specimen	Aegilops triuncialis L.		ESP	Spain						
	SIVIM	T-P16896:Aegi	Observation	Aegilops triuncialis L.	L.	ES	Spain	Majadahonda			40.46	-3.94	0
	SIVIM	T-P16920:Aegi	Observation	Aegilops triuncialis L.	L.	ES	Spain	Ciudad Universitaria			40.37	-3.7	0
00:00.0	SEV	98911-1	Specimen	Aegilops triuncialis L.	L.	ES	Spain	Carretera de Posadas a Villaviciosa		Co			1
00:00.0	GDA	GDA23166-1	Specimen	Aegilops triuncialis L.	L.	ES	Spain	Madrid, S. MartÃn de la Vega, cerro		M			0
	FUND. BIODIVER	1085539	Unknown	Aegilops triuncialis		ESP	Spain	Ciudad Universitaria		M	40.1	-3.1	
	FUND. BIODIVER	1085540	Unknown	Aegilops triuncialis		ESP	Spain	Ajalvir		M	40.1	-3.1	
	FUND. BIODIVER	1093250	Unknown	Aegilops triuncialis		ESP	Spain	Almaraz		Cc	39.1	-5.1	
	FUND. BIODIVER	1946435	Unknown	Aegilops triuncialis L.	L.	ESP	Spain	Ciudad Real		CR			
00:00.0	MA	657096-1	Specimen	Aegilops triuncialis L.	L.	ES	Spain	Segovia		Sg	40	-4	
00:00.0	REDIAM-CMA	405067	Observation	Aegilops triuncialis		ESP	Spain		Cortelazor	H	37.913	-6.61759	699
00:00.0	REDIAM-CMA	415475	Observation	Aegilops triuncialis		ESP	Spain		Cortes de Baza	Gr	37.748	-2.8552	931
00:00.0	UNEX	10348-1	Observation	Aegilops triuncialis L.	_	ESP	Spain	Badajoz: Campus Universitario. 29		Ba	38.8	-7.00001	
00:00.0	HSS	12652	Specimen	Aegilops triuncialis L.	L.	ES	Spain	CÃ¡ceres, proximidades de la ciuda		Cc	39.4952	-6.38377	

00:00.0	MA	557154-1	Specimen	Aegilops triuncialis L.	L.	ES	Spain	Alhambra		CR	38	-3	
00:00.0	HSS	21525	Specimen	Aegilops triuncialis L.	L.	ES	Spain	Valdebotoa		Ba	38.9645	-6.86504	
	SIVIM	S-P01252:Aegi	Observation	Aegilops triuncialis L.	L.	ES	Spain	Belianes, cap al Puig			41.53	0.96	0
	SIVIM	S-P03967:Aegi	Observation	Aegilops triuncialis L.	L.	ES	Spain	Belianes, cap al Puig			41.53	0.96	0
	FUND. BIODIVER	1115794	Unknown	Aegilops triuncialis L.	L.	ESP	Spain	Faramontanos de TÃ¡bara		Za	41.1	-5.1	
	FUND. BIODIVER	51168	Unknown	Aegilops triuncialis L.	L.	ESP	Spain	La Fregeneda		Sa	40.1	-6.1	
	FUND. BIODIVER	56043	Unknown	Aegilops triuncialis L.	L.	ESP	Spain	Castrillo de Don Juan		P	41.1	-3.1	
00:00.0	SEV	101360-1	Specimen	Aegilops triuncialis L.	L.	ES	Spain	Near Arenillas, San Roque		Ca			170
00:00.0	REDIAM-CMA	45156	Observation	Aegilops triuncialis		ESP	Spain		Rute	Co	37.3271	-4.34502	860
	IPK	32098	Living	Aegilops triuncialis L. subsp. triuncialis var. fla		ESP	Spain	Guara					
	SIVIM	T-P06435:Aegi	Observation	Aegilops triuncialis L.	L.	ES	Spain	Cheste			39.45	-0.79	250
	FUND. BIODIVER	1557995	Unknown	Aegilops triuncialis		ESP	Spain	Soto de Cerrato		P	41.1	-4.1	850
	FUND. BIODIVER	83631	Unknown	Aegilops triuncialis L.	L.	ESP	Spain	Navamorales		Sa	40.1	-5.1	
	RUS001	VIR100602166	Specimen	Aegilops triuncialis L.		ESP	Spain						
00:00.0	REDIAM-CMA	190567	Observation	Aegilops triuncialis		ESP	Spain		Constantina	Se	37.9481	-5.62298	699
	FUND. BIODIVER	1050668	Unknown	Aegilops triuncialis L.	L.	ESP	Spain	RegiÃ³n inferior		Ca			
	FUND. BIODIVER	1835133	Unknown	Aegilops triuncialis L.	L.	ESP	Spain	Provincia de Ciudad Real		CR			
00:00.0	COFC	52520-1	Specimen	Aegilops triuncialis L.	L.	ES	Spain	Castilblanco; casa de 'Rompealbarc		Ba	39	-5	1
00:00.0	REDIAM-CMA	80608	Observation	Aegilops triuncialis		ESP	Spain		Ronda	Ma	36.7571	-5.00571	700
00:00.0	UNEX	05900-1	Observation	Aegilops triuncialis L.	_	ESP	Spain	Zarza la Mayor: Zarza la Mayor. 29		Cc	39.8	-6.9	
00:00.0	HSS	4021	Specimen	Aegilops triuncialis L.	L.	ES	Spain	Zafra, sierras calcÃ¡reas entre El Ra		Ba	38.4124	-6.30879	
	SIVIM	T-P10781:Aegi	Observation	Aegilops triuncialis L.	L.	ES	Spain	Faramontanos de TÃ¡bara			41.78	-5.88	0
00:00.0	REDIAM-CMA	390484	Observation	Aegilops triuncialis		ESP	Spain		Huelma	J	37.6797	-3.47971	1199
	IPK	AE 631	Living	Aegilops triuncialis L. subsp. triuncialis var. flavescen			Spain	Huebma (Andalousie)			37.6497	-3.55	
	SIVIM	Q-P03611:Aegi	Observation	Aegilops triuncialis L.	L.	ES	Spain	Villamalur			39.99	-0.42	0
	SIVIM	R-P04799:Aegi	Observation	Aegilops triuncialis L.	L.	ES	Spain	Navalgrande-Guimorcondo (provincia de A			40.64	-4.3	1400
	IDBD-GN	42518	Observation	Aegilops triuncialis L.	L.	ES	Spain	RÃo AragÃ³n	SangÃ¼esa	Na	42.5681	-1.27733	400
00:00.0	GDA	GDA30025-2	Specimen	Aegilops triuncialis L.	L.	ES	Spain	Granada, Iznalloz, Cueva del Agua		GR			1700
	ESP004	NC010108	Specimen	Aegilops triuncialis L.		ESP	Spain	Palacios de La Sierra/Salas de Los Infante			41.9833	-3.16667	1110
00:00.0	FUND. BIODIVER	1369183	Unknown	Aegilops triuncialis L.	L.	ESP	Spain	Berlanga de Duero, hacia Morales		So	41.1	-2.1	880
	FUND. BIODIVER	1463960	Unknown	Aegilops triuncialis L.	L.	ESP	Spain	Les Avellanes		L	41.1	0.1	600
	FUND. BIODIVER	92445	Unknown	Aegilops triuncialis L.	L.	ESP	Spain	RÃ¡bano		Va	41.1	-3.1	
	FUND. BIODIVER	1554593	Unknown	Aegilops triuncialis L.	L.	ESP	Spain	pte. de Castro		Le	42.1	-5.1	
00:00.0	REDIAM-CMA	147108	Observation	Aegilops triuncialis		ESP	Spain		CardeÃ±a	Co	38.3048	-4.22889	490
00:00.0	REDIAM-CMA	152909	Observation	Aegilops triuncialis		ESP	Spain		Ronda	Ma	36.6941	-5.03905	1346
00:00.0	REDIAM-CMA	164995	Observation	Aegilops triuncialis		ESP	Spain		Espiel	Co	38.2124	-4.99144	643
00:00.0	HUAL	5846-1	Specimen	Aegilops triuncialis L.	L.	ES	Spain	SÂª de Los Filabres, EscÃºllar, prÃ³		Al	37.231	-2.746	
00:00.0	MA	718178-1	Specimen	Aegilops triuncialis L.	L.	ES	Spain	Petilla de AragÃ³n.Selva.		Na	42	-1	
00:00.0	REDIAM-CMA	189998	Observation	Aegilops triuncialis		ESP	Spain		Aracena	H	37.8754	-6.52836	697
	SIVIM	T-P16907:Aegi	Observation	Aegilops triuncialis L.	L.	ES	Spain	Loeches			40.37	-3.47	0
00:00.0	COFC	52518-1	Specimen	Aegilops triuncialis L.	L.	ES	Spain	Campus Universitario		Ba	38	-6	1

	ADIMAN	40	Observation	Aegilops triuncialis		ESP	Spain	EnguÃ-danos		CU	39.6671	-1.71038	
	FUND. BIODIVER	1030247	Unknown	Aegilops triuncialis L.	L.	ESP	Spain	Campillo de Arenas, Cerro ALberqu		J	37.1	-3.1	1300
	GJO	GJO-0015441	Unknown	Aegilops triuncialis L.		Spain	Spain	Spanien, RegiÃ³n de Murcia; Murcia, loc. herbidis ad Puerta de la Cadena in Sie					
00:00.0	REDIAM-CMA	299501	Observation	Aegilops triuncialis		ESP	Spain		Cazorla	J	37.8332	-2.88311	1378
	IPK	AE 1146	Living	Aegilops triuncialis L.			Spain	Portugal, Prov. Tras-os-Montes(Alto Douro			41.9292	-6.27083	
	SIVIM	U-P09881:Aegi	Observation	Aegilops triuncialis L.	L.	ES	Spain	Cuneta a 7 Km. de Ruidera			38.93	-2.88	0
00:00.0	BC	805570	Specimen	Aegilops triuncialis L.	L.	ES	Spain	Orihuela del Tremedal; Orihuela del		Te	40.4635	-1.70236	
	RUS001	VIR100602129	Specimen	Aegilops triuncialis L.		ESP	Spain						
	RUS001	VIR100602065	Specimen	Aegilops triuncialis L.		ESP	Spain						
00:00.0	BC	70835	Specimen	Aegilops triuncialis L.	L.	ES	Spain	Barcelona; Sables granitiques prÃ¨s		B	41.41	2.1	
	FUND. BIODIVER	1085273	Unknown	Aegilops triuncialis		ESP	Spain	Tres Cantos		M	40.1	-3.1	
	FUND. BIODIVER	1090319	Unknown	Aegilops triuncialis		ESP	Spain	Majadahonda		M	40.1	-3.1	
	FUND. BIODIVER	1093235	Unknown	Aegilops triuncialis		ESP	Spain	Finca de Valdelasyeguas, Aliseda		Cc	39.1	-6.1	
00:00.0	FUND. BIODIVER	1946442	Unknown	Aegilops triuncialis L.	L.	ESP	Spain	Manzanares, de Almagro a Calzada		CR	38.1	-3.1	700
	GDA	GDA43469-1-3	Specimen	Aegilops triuncialis L.	L.	ES	Spain	Granada, Guadix, Rambla Becerra.		GR			990
00:00.0	REDIAM-CMA	408707	Observation	Aegilops triuncialis		ESP	Spain		GÃ©nave	J	38.43	-2.75189	794
00:00.0	REDIAM-CMA	412682	Observation	Aegilops triuncialis		ESP	Spain		El Castillo de las	Se	37.6554	-6.22919	207
00:00.0	REDIAM-CMA	415838	Observation	Aegilops triuncialis		ESP	Spain		Cazorla	J	37.8826	-2.89784	1521
00:00.0	SALA	60049-1	Specimen	Aegilops triuncialis L.	L.	ES	Spain	_; Villadepera		Za			
	ESP004	NC050482	Specimen	Aegilops triuncialis L.		ESP	Spain	Cuenca/Villalba de la Sierra 8km N, Cuenc			40.15	-2.13333	940
	FUND. BIODIVER	1115802	Unknown	Aegilops triuncialis L.	L.	ESP	Spain	Faramontanos de TÃ¡bara		Za	41.1	-5.1	
	FUND. BIODIVER	117679	Unknown	Aegilops triuncialis L.	L.	ESP	Spain	Burgos, CastaÃ±ares		Bu	42.1	-3.1	
00:00.0	FUND. BIODIVER	1946455	Unknown	Aegilops triuncialis L.	L.	ESP	Spain	San Lorenzo de Calatrava, Los Cas		CR	38.1	-3.1	800
	FUND. BIODIVER	56194	Unknown	Aegilops triuncialis L.	L.	ESP	Spain	Pedrajas de San Esteban		Va	41.1	-4.1	
	FUND. BIODIVER	1115795	Unknown	Aegilops triuncialis L.	L.	ESP	Spain	Dehesa de Misleo		Za	41.1	-5.1	
	FUND. BIODIVER	1135163	Unknown	Aegilops triuncialis L.	L.	ESP	Spain	Castrofuerte		Le	42.1	-5.1	743
	FUND. BIODIVER	51169	Unknown	Aegilops triuncialis L.	L.	ESP	Spain	San Felices		Sa	40.1	-6.1	
	FUND. BIODIVER	59598	Unknown	Aegilops triuncialis L.	L.	ESP	Spain	Monterrubio de ArmuÃ±a		Sa	40.1	-5.1	
00:00.0	REDIAM-CMA	27045	Observation	Aegilops triuncialis		ESP	Spain		La Puebla de los	Se	37.8384	-5.4244	399
00:00.0	SEV	35044-1	Specimen	Aegilops triuncialis L.	L.	ES	Spain	CardeÃ±a, finca de Campasolo		Co			1
00:00.0	SEV	49885-1	Specimen	Aegilops triuncialis L.	L.	ES	Spain	Aracena. Castillo		H			1
00:00.0	ABH	6475-1	Specimen	Aegilops triuncialis L.	L.	ES	Spain	Villena; Sierra de Salinas		A	38.47	-1.05	
	SIVIM	S-P14055:Aegi	Observation	Aegilops triuncialis L.	L.	ES	Spain	Serrinha			38.39	-8.54	0
	SIVIM	T-P07219:Aegi	Observation	Aegilops triuncialis L.	L.	ES	Spain	Capella			42.15	0.33	0
	SIVIM	T-P06456:Aegi	Observation	Aegilops triuncialis L.	L.	ES	Spain	Sinarcas			39.73	-1.24	0
00:00.0	COFC	11963-1	Specimen	Aegilops triuncialis L.	L.	ES	Spain	Priego de CÃ³rdoba; entre el munici		Co	37	-4	1
	BDBCV-General	276668	Observation	Aegilops triuncialis		ESPA	Spain	NavarrÃ©s	La Canal de Nava	Valenc	39.0517	-0.74664	
00:00.0	MGC	8142-1	Unknown	Aegilops triuncialis L.	L.	ES	Spain	RÃ-o Aguas		Al	0	0	1
00:00.0	S	S05-10796	Specimen	Aegilops triuncialis L.	L.	Spain	Spain	Habitat in Monspela, Massiliae , Smyrnae aridis. lectam in Hispania misit a mioris					
	RUS001	VIR100602142	Specimen	Aegilops triuncialis L.		ESP	Spain						
	RUS001	VIR100602222	Specimen	Aegilops triuncialis L.		ESP	Spain						

	RUS001	VIR100602157	Specimen	Aegilops triuncialis L.		ESP	Spain						
	FUND. BIODIVER	1085281	Unknown	Aegilops triuncialis		ESP	Spain	Villanueva del Pardillo		M	40.51	-4	
	FUND. BIODIVER	1091709	Unknown	Aegilops triuncialis L.	L.	ESP	Spain	RÃo Guadarranque		Cc	39.1	-4.1	
	FUND. BIODIVER	1093222	Unknown	Aegilops triuncialis L.	L.	ESP	Spain	Alrededores de CÃ¡ceres		Cc	39.1	-4.1	
	FUND. BIODIVER	1895399	Unknown	Aegilops triuncialis L.	L.	ESP	Spain	Vitoria, Gasteiz, poligono industrial		Vi	42.1	-2.1	500
00:00.0	FCO	24488-1	Specimen	Aegilops triuncialis L.	L.	ES	Spain	Olmedo; Olmedo, proximidades		Va			
	FUND. BIODIVER	1835121	Unknown	Aegilops triuncialis L.	L.	ESP	Spain	Despenaperros		CR	38.1	-3.1	
00:00.0	FUND. BIODIVER	1835142	Unknown	Aegilops triuncialis L.	L.	ESP	Spain	Villamayor de Calatrava, volcan del		CR	38.1	-3.1	842
00:00.0	REDIAM-CMA	14220	Observation	Aegilops triuncialis		ESP	Spain		Torres	J	37.7783	-3.50598	923
00:00.0	REDIAM-CMA	21094	Observation	Aegilops triuncialis		ESP	Spain		Zufre	H	37.7727	-6.42309	400
00:00.0	REDIAM-CMA	423301	Observation	Aegilops triuncialis		ESP	Spain		Dos Torres	Co	38.5469	-4.77484	474
00:00.0	REDIAM-CMA	274393	Observation	Aegilops triuncialis		ESP	Spain		BelalcÃ¡zar	Co	38.5762	-5.0614	500
	SIVIM	R-P09412:Aegi	Observation	Aegilops triuncialis L.	L.	ES	Spain	Cirueches			41.1	-2.76	0
	SIVIM	S-P02911:Aegi	Observation	Aegilops triuncialis L.	L.	ES	Spain	massif du St. LlorenÃ§: Riera de les Arene			41.54	1.92	0
	SIVIM	Q-P07460:Aegi	Observation	Aegilops triuncialis L.	L.	ES	Spain	Torrebuceit			39.92	-2.53	0
	SIVIM	U-P13533:Aegi	Observation	Aegilops triuncialis L.	L.	ES	Spain	Vinhais, OuzilhÃ£o			41.71	-6.95	0
	SIVIM	T-P28878:Aegi	Observation	Aegilops triuncialis L.	L.	ES	Spain	Llanos de la SeÃ±oria, Romangordo			39.71	-5.79	260
	SIVIM	T-P29814:Aegi	Observation	Aegilops triuncialis L.	L.	ES	Spain	Pr Sierra Monterilla, Requena, Valencia			39.28	-1.37	0
	SIVIM	U-P06651:Aegi	Observation	Aegilops triuncialis L.	L.	ES	Spain	CabeÃ§o da CÃ¢mara			37.04	-8.1	0
00:00.0	UNEX	11783-1	Observation	Aegilops triuncialis L.	_	ESP	Spain	Alconera: En pastizales higrocalizos		Ba	38.4	-6.6	
00:00.0	UNEX	10353-1	Observation	Aegilops triuncialis L.	_	ESP	Spain	Castilblanco: Casa de Rompealbard		Ba	39.3	-5.2	
00:00.0	SEV	112615-1	Specimen	Aegilops triuncialis L.	L.	ES	Spain	Near Estepona, Sierra Bermeja		Ma			1200
00:00.0	SEV	124286-1	Specimen	Aegilops triuncialis L.	L.	ES	Spain	Puerto Cabopino, Marbella		Ma			10
00:00.0	MGC	56762-1	Unknown	Aegilops triuncialis L.	L.	ES	Spain	MÃ¡laga; La AraÃ±a. FÃ¡brica de ce		Ma	36.719	-4.316	50
	SANT	18149	Specimen	Aegilops triuncialis L.		ES	Spain	RubiÃ¡,Cobas,hacia Vilardesilva		Or			
00:00.0	HSS	2592	Specimen	Aegilops triuncialis L.	L.	ES	Spain	Peloche		Ba	39.2346	-5.14346	
00:00.0	BC	70838	Specimen	Triticum triunciale (L.) Raspail	(L.) Raspail	ES	Spain	Urbe; in Castella Nova: La Calderina		To	39.34	-3.75	
	RUS001	VIR100602171	Specimen	Aegilops triuncialis L.		ESP	Spain						
00:00.0	SEV	85160-1	Specimen	Aegilops triuncialis L.	L.	ES	Spain	Lora del RÃo. Cortijo Mazueco		Se			100
00:00.0	SEV	99068-1	Specimen	Aegilops triuncialis L.	L.	ES	Spain	Obejo. Ermita de San Benito		Co			1
00:00.0	COA	41184-1	Specimen	Aegilops triuncialis L.	L.	ES	Spain	4 Km al norte de Santa Eufemia		Co	38.56	-4.95	
	FUND. BIODIVER	1043778	Unknown	Aegilops triuncialis L.	L.	ESP	Spain	OjÃ©n, Sierra Blanca		Ma	36.1	-4.1	
	FUND. BIODIVER	1066941	Unknown	Aegilops triuncialis L.	L.	ESP	Spain	Paradas		Se	37.1	-5.1	
00:00.0	FUND. BIODIVER	1835129	Unknown	Aegilops triuncialis L.	L.	ESP	Spain	Navas de Estena, arroyo de Ciguinu		CR	39.1	-4.1	780
00:00.0	FUND. BIODIVER	1835150	Unknown	Aegilops triuncialis L.	L.	ESP	Spain	Alcubillas, alrededores		CR	38.1	-2.1	830
00:00.0	BC	804983	Specimen	Aegilops triuncialis L.	L.	ES	Spain	Monistrol de Montserrat; Bages: Mo		B	41.605	1.842	275
00:00.0	REDIAM-CMA	384850	Observation	Aegilops triuncialis		ESP	Spain		Jimena	J	37.8179	-3.46976	829
00:00.0	REDIAM-CMA	389411	Observation	Aegilops triuncialis		ESP	Spain		Hornachuelos	Co	37.8767	-5.3235	305
	SANT	2112	Specimen	Aegilops triuncialis L.		ES	Spain	Sierra Nevada,Sierra de Guejar		Gr			
00:00.0	GDAC	GDAC42937-1	Specimen	Aegilops triuncialis L.	L.	ES	Spain	AlmerÃa, LÃºcar, SÂª de LÃºcar.		AL			0
00:00.0	REDIAM-CMA	234585	Observation	Aegilops triuncialis		ESP	Spain		El Real de la Jara	Se	37.9511	-5.96866	587

00:00.0	REDIAM-CMA	238640	Observation	Aegilops triuncialis		ESP	Spain		Cumbres Mayores	H	38.0089	-6.66486	503
00:00.0	SEV	98923-1	Specimen	Aegilops triuncialis L.	L.	ES	Spain	Sierra Margarita		Ca			1
00:00.0	SEV	99063-1	Specimen	Aegilops triuncialis L.	L.	ES	Spain	Hills above Los Barrios		Ca			300
	SIVIM	T-P21024:Aegi	Observation	Aegilops triuncialis L.	L.	ES	Spain	PeÃ±a El Altar, Los Villares			37.67	-3.9	1150
00:00.0	GDA	GDA15837-1-2	Specimen	Aegilops triuncialis L.	L.	ES	Spain	Granada, SÂª Sagra, HuÃ©scar, co		GR			0
	RUS001	VIR100602172	Specimen	Aegilops triuncialis L.		ESP	Spain						
00:00.0	FCO	6560-1	Specimen	Aegilops triuncialis L.	L.	ES	Spain	CastejÃ³n de la PeÃ±a					
	FUND. BIODIVER	1035842	Unknown	Aegilops triuncialis		ESP	Spain	Sierra de Aguas, Arroyo de los Hue		Ma	36.1	-4.1	
	FUND. BIODIVER	1043777	Unknown	Aegilops triuncialis L.	L.	ESP	Spain	Marbella		Ma	36.1	-4.1	
	FUND. BIODIVER	1072127	Unknown	Aegilops triuncialis L.	L.	ESP	Spain	MorÃ³n de la Frontera, alrededores		Se	36.1	-5.1	
00:00.0	FUND. BIODIVER	1835128	Unknown	Aegilops triuncialis L.	L.	ESP	Spain	Moral de Calatrava, sierra de Moral		CR	38.1	-3.1	
00:00.0	FUND. BIODIVER	1835149	Unknown	Aegilops triuncialis L.	L.	ESP	Spain	Aldea del Rey, cerro de la Vaqueriz		CR	38.1	-3.1	750
00:00.0	REDIAM-CMA	393065	Observation	Aegilops triuncialis		ESP	Spain		Arroyomolinos de	H	38.0098	-6.43185	700
00:00.0	MGC	11818-1	Unknown	Aegilops triuncialis L.	L.	ES	Spain	Sierra Blanca. Carretera de OjÃ©n		Ma	0	0	1
	GDA	GDA42723-1	Specimen	Aegilops triuncialis L.	L.	ES	Spain	Granada, Deifontes, base de la Sier		GR			800
00:00.0	GDAC	GDAC26136-2	Specimen	Aegilops triuncialis L.	L.	ES	Spain	Granada, SÂª de Baza, entre la esta		GR			0
00:00.0	BC	655691	Specimen	Aegilops triuncialis L.	L.	ES	Spain	el Bruc; Anoia: el Bruc, el Bruc de D		B	41.586	1.77	550
00:00.0	REDIAM-CMA	247339	Observation	Aegilops triuncialis		ESP	Spain		Cortegana	H	37.9327	-6.82174	600
00:00.0	REDIAM-CMA	278064	Observation	Aegilops triuncialis		ESP	Spain		AlanÃ-s	Se	38.0633	-5.65362	555
00:00.0	REDIAM-CMA	282189	Observation	Aegilops triuncialis		ESP	Spain		Hinojosa del Duq	Co	38.4155	-5.06206	555
	ESP004	NC043498	Specimen	Aegilops triuncialis L.		ESP	Spain	Hontoba, province of Guadalajara			40.45	-3.03333	730
	RUS001	VIR100602162	Specimen	Aegilops triuncialis L.		ESP	Spain						
	SIVIM	T-P27678:Aegi	Observation	Aegilops triuncialis L.	L.	ES	Spain	PrÃ³x. Cj. MolejÃ³n, SÂª Gallinera			37.39	-4.35	0
	SIVIM	T-P28870:Aegi	Observation	Aegilops triuncialis L.	L.	ES	Spain	El Moralo, SerrejÃ³n			39.8	-5.92	0
	SIVIM	T-P30065:Aegi	Observation	Aegilops triuncialis L.	L.	ES	Spain	Camino al Regajo, Jalance, Valencia			39.1	-1.14	0
	SIVIM	U-P06646:Aegi	Observation	Aegilops triuncialis L.	L.	ES	Spain	CabeÃ§a Aguda			37.13	-8.21	170
00:00.0	FUND. BIODIVER	1946459	Unknown	Aegilops triuncialis L.	L.	ESP	Spain	Villamanrique, arroyo de la Fuente d		CR	38.1	-2.1	840
	FUND. BIODIVER	54855	Unknown	Aegilops triuncialis L.	L.	ESP	Spain	BÃ³veda del rÃ-o Almar		Sa	40.1	-4.1	
	RUS001	VIR100602138	Specimen	Aegilops triuncialis L.		ESP	Spain						
00:00.0	FUND. BIODIVER	1835137	Unknown	Aegilops triuncialis L.	L.	ESP	Spain	San Lorenzo de Calatrava, umbria d		CR	38.1	-3.1	620
	FUND. BIODIVER	1093226	Unknown	Aegilops triuncialis		ESP	Spain	Toril		Cc			
00:00.0	REDIAM-CMA	41398	Observation	Aegilops triuncialis		ESP	Spain		PeÃ±aflor	Se	37.7457	-5.37158	137
00:00.0	REDIAM-CMA	48192	Observation	Aegilops triuncialis		ESP	Spain		Rute	Co	37.3279	-4.33222	800
00:00.0	REDIAM-CMA	73030	Observation	Aegilops triuncialis		ESP	Spain		Villanueva de la R	J	38.2839	-3.86098	599
00:00.0	REDIAM-CMA	384090	Observation	Aegilops triuncialis		ESP	Spain				37.6694	-3.41945	
00:00.0	REDIAM-CMA	390944	Observation	Aegilops triuncialis		ESP	Spain		Ronda	Ma	36.6941	-5.03914	1346
	RUS001	VIR100602223	Specimen	Aegilops triuncialis L.		ESP	Spain						
	RUS001	VIR100602158	Specimen	Aegilops triuncialis L.		ESP	Spain						
00:00.0	REDIAM-CMA	414660	Observation	Aegilops triuncialis		ESP	Spain		Baza	Gr	37.4581	-2.87803	1146
	SIVIM	S-P14066:Aegi	Observation	Aegilops triuncialis L.	L.	ES	Spain	Freixo do Meio			39.38	-8.88	145
	SIVIM	T-P06417:Aegi	Observation	Aegilops triuncialis L.	L.	ES	Spain	Aldea de La Torre			39.64	-1.36	0

	SIVIM	T-P09347:Aegi	Observation	Aegilops triuncialis L.	L.	ES	Spain	PontÃ³n de la Oliva			40.83	-3.47	0
	FUND. BIODIVER	1369185	Unknown	Aegilops triuncialis L.	L.	ESP	Spain	Medinaceli		So	40.1	-2.1	
	FUND. BIODIVER	915851	Unknown	Aegilops triuncialis L.	L.	ESP	Spain	Guardiola de BerguedÃ		B	42.1	1.1	
	FUND. BIODIVER	971339	Unknown	Aegilops triuncialis L.	L.	ESP	Spain	Calatayud		Z	41.1	-1.1	
	FUND. BIODIVER	1038227	Unknown	Aegilops triuncialis L.	L.	ESP	Spain	Puerto de las Pedrizas		Ma	36.1	-4.1	
	FUND. BIODIVER	1067628	Unknown	Aegilops triuncialis L.	L.	ESP	Spain	Sevilla		Se	37.1	-5.1	
00:00.0	FUND. BIODIVER	1835120	Unknown	Aegilops triuncialis L.	L.	ESP	Spain	Herencia, cerro Navajo, lad. SE reta		CR	39.1	-3.1	660
00:00.0	FUND. BIODIVER	1835141	Unknown	Aegilops triuncialis L.	L.	ESP	Spain	Retuerta del Bullaque, Cabaneros		CR	39.1	-4.1	
	SIVIM	R-P09669:Aegi	Observation	Aegilops triuncialis L.	L.	ES	Spain	entre Cirueches y Cambias			41.1	-2.76	0
	SIVIM	S-P01245:Aegi	Observation	Aegilops triuncialis L.	L.	ES	Spain	els Omells de Na Gaia, afores del poble			41.44	0.96	0
	SIVIM	S-P03960:Aegi	Observation	Aegilops triuncialis L.	L.	ES	Spain	els Omells de Na Gaia, afores del poble			41.44	0.96	0
00:00.0	REDIAM-CMA	128462	Observation	Aegilops triuncialis		ESP	Spain		Cazalla de la Sier	Se	37.9301	-5.71162	443
00:00.0	REDIAM-CMA	141024	Observation	Aegilops triuncialis		ESP	Spain		Monda	Ma	36.647	-4.90733	782
00:00.0	REDIAM-CMA	386963	Observation	Aegilops triuncialis		ESP	Spain		Aroche	H	37.878	-6.97682	315
	IPK	AE 672	Living	Aegilops triuncialis L. subsp. triuncialis var. flavescen			Spain	Huelma Andalusien			37.6497	-3.55	
	SIVIM	T-P16908:Aegi	Observation	Aegilops triuncialis L.	L.	ES	Spain	TamajÃ³n			40.92	-3.35	0
00:00.0	GDAC	GDAC11570-1	Specimen	Aegilops triuncialis L.	L.	ES	Spain	Granada, Puerto de la Mora.		GR			0
	SIVIM	Q-P07458:Aegi	Observation	Aegilops triuncialis L.	L.	ES	Spain	Olmedilla de AlarcÃ³n			39.56	-2.18	0
	SIVIM	U-P13532:Aegi	Observation	Aegilops triuncialis L.	L.	ES	Spain	BraganÃ§a, Babe			41.79	-6.71	0
00:00.0	BC	92747	Specimen	Aegilops triuncialis L.	L.	ES	Spain	Benaocaz; Benaocaz		Ca	36.71	-5.41	1000
00:00.0	COA	41191-1	Specimen	Aegilops triuncialis L.	L.	ES	Spain	Km 10 de BelalcÃ¡zar hacia el oeste		Co	38.56	-5.3	
00:00.0	HSS	12600	Specimen	Aegilops triuncialis L.	L.	ES	Spain	Villafranca de los Barros		Ba	38.5924	-6.30208	
00:00.0	HUAL	1141-1	Specimen	Aegilops triuncialis L.	L.	ES	Spain	SÂª de Cazorla, Mirador de Las Pal		J	37.952	-2.937	
	FUND. BIODIVER	1369189	Unknown	Aegilops triuncialis L.	L.	ESP	Spain	CaÃ±amaque		So	41.1	-2.1	
00:00.0	REDIAM-CMA	141296	Observation	Aegilops triuncialis		ESP	Spain		Monda	Ma	36.6468	-4.90728	782
00:00.0	REDIAM-CMA	177590	Observation	Aegilops triuncialis		ESP	Spain		AlanÃ-s	Se	38.0277	-5.70091	676
00:00.0	COFC	41304-1	Specimen	Aegilops triuncialis L.	L.	ES	Spain	Lucena; carretera entre el municipio		Co	37	-4	1
00:00.0	COFC	50170-1	Specimen	Aegilops triuncialis L.	L.	ES	Spain	Valle del Guadiato; casas 'Lagar de		Co			1
00:00.0	MA	569169-1	Specimen	Aegilops triuncialis L.	L.	ES	Spain	SebÃºlcor (carretera de Cantalejo)		Sg			
00:00.0	HUAL	1138-1	Specimen	Aegilops triuncialis L.	L.	ES	Spain	SÂª de Cazorla, de La Fuente del C		J	37.907	-2.949	
	SANT	18366	Specimen	Aegilops triuncialis L.		ES	Spain	RubiÃ¡,Cobas,en la estaciÃ³n de fer		Or			
00:00.0	UNEX	10355-1	Observation	Aegilops triuncialis L.	_	ESP	Spain	Zafra: Rivera de Zafra. 29SQC25		Ba	38.4	-6.5	
00:00.0	SEV	98915-1	Specimen	Aegilops triuncialis L.	L.	ES	Spain	Entre San Clixto y Hornachuelos		Co			1
00:00.0	SEV	98940-1	Specimen	Aegilops triuncialis L.	L.	ES	Spain	Entre MorÃ³n y Pruna, a 6 Km de P		Se			1
	SIVIM	T-P16880:Aegi	Observation	Aegilops triuncialis L.	L.	ES	Spain	CobeÃ±a			40.55	-3.59	0
	SIVIM	T-P16910:Aegi	Observation	Aegilops triuncialis L.	L.	ES	Spain	Ajalvir			40.46	-3.58	0
00:00.0	BC	92743	Specimen	Aegilops triuncialis L.	L.	ES	Spain	Alcala de Guadaira; AlcalÃ¡ de Gua		Se	37.33	-5.88	
	SANT	2111	Specimen	Aegilops triuncialis L.		ES	Spain	Corbera de Alcira		V			
00:00.0	UNEX	10349-1	Observation	Aegilops triuncialis L.	_	ESP	Spain	AlÃ-a: La Calera.Hornos de la cal. :		Cc	39.5	-5.3	
00:00.0	MGC	29641-1	Unknown	Aegilops triuncialis L.	L.	ES	Spain	Pasado el Puerto de la Mora		Gr	0	0	1
00:00.0	MGC	10436-1	Unknown	Aegilops triuncialis L.	L.	ES	Spain	Archidona; Entre Salinas y Archidon		Ma	0	0	1

	RUS001	VIR100602077	Specimen	Aegilops triuncialis L.		ESP	Spain						
00:00.0	BC	646878	Specimen	Aegilops triuncialis L.	L.	ES	Spain	Pineda de Mar; Pineda		B	41.6	2.7	
	FUND. BIODIVER	1074433	Unknown	Aegilops triuncialis L.	L.	ESP	Spain	Venta de CÃ¡rdenas		CR	38.1	-3.1	
	FUND. BIODIVER	1085282	Unknown	Aegilops triuncialis		ESP	Spain	Hoyo de Manzanares		M	40.1	-3.1	
	FUND. BIODIVER	1093075	Unknown	Aegilops triuncialis		ESP	Spain	CeclavÃ-n		Cc	39.1	-6.1	
	FUND. BIODIVER	1093223	Unknown	Aegilops triuncialis L.	L.	ESP	Spain	Coria		Cc	39.1	-6.1	
	FUND. BIODIVER	1093244	Unknown	Aegilops triuncialis		ESP	Spain	Coria		Cc	39.1	-6.1	
00:00.0	COFC	46906-1	Specimen	Aegilops triuncialis L.	L.	ES	Spain	Hornachuelos; camino vecinal de V		Co			1
	FUND. BIODIVER	1000500	Unknown	Aegilops triuncialis		ESP	Spain	Fresneda de Allarejos		Cu	39.1	-2.1	
	FUND. BIODIVER	1027220	Unknown	Aegilops triuncialis L.	L.	ESP	Spain	Marmolejo		J	38.1	-3.1	650
00:00.0	GDA	GDA10270-1-1	Specimen	Aegilops triuncialis L.	L.	ES	Spain	Granada, AlmegÃ-jar, prÃ³x. barran		GR			900
00:00.0	ABH	5010-1	Specimen	Aegilops triuncialis L.	L.	ES	Spain	Quatretondeta; Riu Vall de Ceta		A	38.72	-0.33	
1879-06-1	BC	824679	Specimen	Aegilops triuncialis L.	L.	ES	Spain	Terrassa		B			
00:00.0	REDIAM-CMA	395260	Observation	Aegilops triuncialis		ESP	Spain		Cabra	Co	37.4861	-4.3549	1041
00:00.0	REDIAM-CMA	382749	Observation	Aegilops triuncialis		ESP	Spain		Torres	J	37.7783	-3.50615	923
	SIVIM	R-P09469:Aegi	Observation	Aegilops triuncialis L.	L.	ES	Spain	Riba de Santiuste			41.19	-2.76	1000
	SIVIM	U-P08132:Aegi	Observation	Aegilops triuncialis L.	L.	ES	Spain	La Nava, Berzocana			39.36	-5.55	0
00:00.0	GDA	GDA30026-1-2	Specimen	Aegilops triuncialis L.	L.	ES	Spain	Granada, Alhambra, Cementerio.		GR			0
00:00.0	SEV	101902-1	Specimen	Aegilops triuncialis L.	L.	ES	Spain	Arroyo de Montuerga, entre Los Bla		Co			1
00:00.0	BC	646877	Specimen	Aegilops triuncialis L.	L.	ES	Spain	Llinars del VallÃ¨s; Dosrius, vers Ca		B	41.6	2.46	
	FUND. BIODIVER	1085280	Unknown	Aegilops triuncialis		ESP	Spain	Alcobendas		M	40.1	-3.1	
	FUND. BIODIVER	1091708	Unknown	Aegilops triuncialis L.	L.	ESP	Spain	AlÃ-a		Cc	39.1	-5.1	
	FUND. BIODIVER	1093221	Unknown	Aegilops triuncialis L.	L.	ESP	Spain	Bazagona		Cc	39.1	-5.1	
	FUND. BIODIVER	1093264	Unknown	Aegilops triuncialis L.	L.	ESP	Spain	Comarca de la Vera		Cc	39.1	-5.1	
	FUND. BIODIVER	1946449	Unknown	Aegilops triuncialis L.	L.	ESP	Spain	Provincia de Ciudad Real		CR			
00:00.0	COFC	38999-1	Specimen	Aegilops triuncialis L.	L.	ES	Spain	IznÃƒÂ¡jar; loma del SantÃƒÂ-simo		Co			1
00:00.0	REDIAM-CMA	398194	Observation	Aegilops triuncialis		ESP	Spain		Hornachuelos	Co	37.9339	-5.40363	494
00:00.0	REDIAM-CMA	422614	Observation	Aegilops triuncialis		ESP	Spain		Villanueva de CÃ	Co	38.3224	-4.52456	675
	SIVIM	R-P09387:Aegi	Observation	Aegilops triuncialis L.	L.	ES	Spain	Entre FuencemillÃ¡n y Cogolludo			40.92	-3.11	0
00:00.0	COFC	4631-1	Specimen	Aegilops triuncialis L.	L.	ES	Spain	CardeÃƒÂ±a; finca 'YegÃƒÂ¼erizo'		Co			1
00:00.0	MGC	2539-1	Unknown	Aegilops triuncialis L.	L.	ES	Spain	Bezas; _		Te	40.32	-1.29	1
00:00.0	REDIAM-CMA	196463	Observation	Aegilops triuncialis		ESP	Spain		Cazorla	J	37.8685	-2.92651	1426
00:00.0	REDIAM-CMA	235743	Observation	Aegilops triuncialis		ESP	Spain		El Real de la Jara	Se	37.9223	-5.97136	412
	SIVIM	T-P19059:Aegi	Observation	Aegilops triuncialis L.	L.	ES	Spain	Proximidades a NarvÃ¡ez			37.31	-2.88	1200
	SIVIM	T-P20230:Aegi	Observation	Aegilops triuncialis L.	L.	ES	Spain	Proximidades de Baena			37.57	-4.35	0
	SIVIM	T-P26222:Aegi	Observation	Aegilops triuncialis L.	L.	ES	Spain	CaÃ±ada de Biar, Biar			38.55	-0.81	0
00:00.0	SEV	101355-1	Specimen	Aegilops triuncialis L.	L.	ES	Spain	Conil de la Frontera. Pinares de Las		Ca			30
	RUS001	VIR100602137	Specimen	Aegilops triuncialis L.		ESP	Spain						
00:00.0	UNEX	29028-1	Observation	Aegilops triuncialis L.	_	ESP	Spain	Monterrubio de la Serena: Carretera		Ba	38.6	-5.5	
00:00.0	UNEX	29041-1	Observation	Aegilops triuncialis L.	_	ESP	Spain	Monterrubio de la Serena: Carretera		Ba	38.6	-5.5	
00:00.0	W	42571	Unknown	Aegilops triuncialis L.		ESP	Spain	Barcelone: sables granitiques prÃ¨s la Bonanova.					

	FUND. BIODIVER	1085538	Unknown	Aegilops triuncialis		ESP	Spain	Loeches		M	40.1	-3.1	
00:00.0	FUND. BIODIVER	1946434	Unknown	Aegilops triuncialis L.	L.	ESP	Spain	Fuencaliente, confluencia de los rios		CR	38.1	-2.1	1000
00:00.0	MGC	44022-1	Unknown	Aegilops triuncialis L.	L.	ES	Spain	Estepona; Sierra Bermeja. Tinao de		Ma	0	0	160
00:00.0	REDIAM-CMA	395697	Observation	Aegilops triuncialis		ESP	Spain		Luque	Co	37.522	-4.25821	979
00:00.0	REDIAM-CMA	399794	Observation	Aegilops triuncialis		ESP	Spain		Hornachuelos	Co	37.9958	-5.45278	623
00:00.0	REDIAM-CMA	405003	Observation	Aegilops triuncialis		ESP	Spain		Santiago-Pontone	J	38.1535	-2.7953	899
00:00.0	COA	41152-1	Specimen	Aegilops triuncialis L.	L.	ES	Spain	Sierra del Castillo, cerca de Espiel,		Co	38.11	-5.05	
	SIVIM	R-P09690:Aegi	Observation	Aegilops triuncialis L.	L.	ES	Spain	Riba de Santiuste			41.19	-2.76	1000
	SIVIM	S-P01247:Aegi	Observation	Aegilops triuncialis L.	L.	ES	Spain	l'Espluga Calba, els Bassals			41.44	0.96	0
	SIVIM	S-P03962:Aegi	Observation	Aegilops triuncialis L.	L.	ES	Spain	l'Espluga Calba, els Bassals			41.44	0.96	0
	BDBCV-General	76829	Observation	Aegilops triuncialis		ESPA	Spain	El Toro	El Alto Palancia	Castel	39.9542	-0.83434	
	GDAC	GDAC11569-1	Specimen	Aegilops triuncialis L.	L.	ES	Spain	Granada, SÂª de HuÃ©tor, Chorrillo		GR			0
00:00.0	ABH	9815-1	Specimen	Aegilops triuncialis L.	L.	ES	Spain	HondÃ³n de las Nieves; Cerro de Sa		A	38.27	-0.9	
00:00.0	SEV	30539-1	Specimen	Aegilops triuncialis L.	L.	ES	Spain	Entre MorÃ³n y El Saucejo, finca La		Se			1
	IDBD-GN	42512	Observation	Aegilops triuncialis L.	L.	ES	Spain		MaÃ±eru	Na	42.6697	-1.84197	
00:00.0	BC	601440	Specimen	Aegilops triuncialis L.	L.	ES	Spain	VimbodÃ-; Muntanyes de Prades: V		T	41.31	1.03	525
00:00.0	ABH	5351-1	Specimen	Aegilops triuncialis L.	L.	ES	Spain	SÂªEspuÃ±a, Valle del Rio EspuÃ±		Mu	37.86	-1.56	
00:00.0	GDA	GDA12184-1-2	Specimen	Aegilops triuncialis L.	L.	ES	Spain	Granada, Lobras, falda del cerro Ve		GR			800
	BDBCV-General	74118	Observation	Aegilops triuncialis		ESPA	Spain	Castillo de Villam	El Alto Mijares	Castel	40.1246	-0.35957	
00:00.0	COFC	12021-1	Specimen	Aegilops triuncialis L.	L.	ES	Spain	Cabra; entre el municipio y Carcabu		Co	37	-4	1
00:00.0	COFC	12022-1	Specimen	Aegilops triuncialis L.	L.	ES	Spain	Almedinilla; Sierra de Albayate		Co	37	-4	1
00:00.0	COFC	16303-1	Specimen	Aegilops triuncialis L.	L.	ES	Spain	Los BlÃƒÂ¡zquez; carretera a La Gr		Co	38	-5	1
00:00.0	COFC	46905-1	Specimen	Aegilops triuncialis L.	L.	ES	Spain	ctra hacia Sevilla; finca 'Los Cabezo		Co			1
	FUND. BIODIVER	1000499	Unknown	Aegilops triuncialis		ESP	Spain	JÃ¡bala, Boniches		Cu	39.1	-1.1	
	FUND. BIODIVER	1027219	Unknown	Aegilops triuncialis L.	L.	ESP	Spain	Marmolejo		J	38.1	-4.1	500
00:00.0	FUND. BIODIVER	1332457	Unknown	Aegilops triuncialis L.	L.	ESP	Spain	RÃ-o de la Cabrera		J	38.1	-3.1	
	FUND. BIODIVER	76092	Unknown	Aegilops triuncialis L.	L.	ESP	Spain	Castrillo de la GuareÃ±a		Za	41.1	-5.1	
00:00.0	REDIAM-CMA	106042	Observation	Aegilops triuncialis		ESP	Spain		AndÃºjar	J	38.2569	-4.03636	597
00:00.0	REDIAM-CMA	112904	Observation	Aegilops triuncialis		ESP	Spain		Carcabuey	Co	37.4414	-4.2918	700
	ESP004	NC050500	Specimen	Aegilops triuncialis L.		ESP	Spain	CaÃƒÂ±averas/Priego 5km NE, Villaconejo			40.4	-2.33333	880
00:00.0	FUND. BIODIVER	1946458	Unknown	Aegilops triuncialis L.	L.	ESP	Spain	Villamayor de Calatrava, volcan del		CR	38.1	-3.1	842
	FUND. BIODIVER	62022	Unknown	Aegilops triuncialis L.	L.	ESP	Spain	La Bouza		Sa	40.1	-6.1	
00:00.0	REDIAM-CMA	31202	Observation	Aegilops triuncialis		ESP	Spain		La Puebla de los	Se	37.7847	-5.35286	220
00:00.0	REDIAM-CMA	72872	Observation	Aegilops triuncialis		ESP	Spain		La Puebla del RÃ	Se	36.9613	-6.1829	2
	SIVIM	S-P14065:Aegi	Observation	Aegilops triuncialis L.	L.	ES	Spain	Freixo do Meio			39.38	-8.88	146
	SIVIM	T-P06408:Aegi	Observation	Aegilops triuncialis L.	L.	ES	Spain	Andilla			39.82	-0.89	0
	SIVIM	T-P09203:Aegi	Observation	Aegilops triuncialis L.	L.	ES	Spain	Soto de Cerrato			41	-4.42	0
	FUND. BIODIVER	1587956	Unknown	Aegilops triuncialis		ESP	Spain	Tierra de Campos		P			
	FUND. BIODIVER	993577	Unknown	Aegilops triuncialis		ESP	Spain	TamajÃ³n		Gu	40.1	-3.1	
	MA	700960-1	Specimen	Aegilops triuncialis L.	L.	ES	Spain	Ciudad Universitaria		M			
	SIVIM	T-P19061:Aegi	Observation	Aegilops triuncialis L.	L.	ES	Spain	Cerro de Calabozo			37.22	-3.45	1680

	SIVIM	T-P20231:Aegi	Observation	Aegilops triuncialis L.	L.	ES	Spain	DoÃ±a MencÃ-a			37.48	-4.35	0
	SIVIM	T-P26224:Aegi	Observation	Aegilops triuncialis L.	L.	ES	Spain	Proximidades de Biar, Biar			38.55	-0.81	0
00:00.0	FCO	10586-1	Specimen	Aegilops triuncialis L.	L.	ES	Spain	Santillana, embalse		M			
	FUND. BIODIVER	1115797	Unknown	Aegilops triuncialis L.	L.	ESP	Spain	Moreruela de TÃ¡bara		Za	41.1	-5.1	
00:00.0	FUND. BIODIVER	1946450	Unknown	Aegilops triuncialis L.	L.	ESP	Spain	Puebla del Principe, cercanias del c		CR	38.1	-2.1	940
00:00.0	FUND. BIODIVER	1369199	Unknown	Aegilops triuncialis L.	L.	ESP	Spain	La Rubia		So	41.1	-2.1	1100
	FUND. BIODIVER	930417	Unknown	Aegilops triuncialis L.	L.	ESP	Spain	VimbodÃ-, Vallclara, La FebrÃ³		T	41.1	0.1	
	SANT	20974	Specimen	Aegilops triuncialis L.		ES	Spain	La Moncloa		M			
00:00.0	REDIAM-CMA	148402	Observation	Aegilops triuncialis		ESP	Spain		Santa Olalla del C	H	37.8977	-6.21487	500
00:00.0	REDIAM-CMA	181778	Observation	Aegilops triuncialis		ESP	Spain		Cortelazor	H	37.9124	-6.61769	699
	SIVIM	T-P06091:Aegi	Observation	Aegilops triuncialis L.	L.	ES	Spain	de la comarca del RÃ-o Aguas			37.13	-1.98	200
	SIVIM	T-P06468:Aegi	Observation	Aegilops triuncialis L.	L.	ES	Spain	Onteniente			38.82	-0.69	0
	SIVIM	T-P09156:Aegi	Observation	Aegilops triuncialis L.	L.	ES	Spain	CabezÃ³n			41.71	-4.68	0
	GDA	GDA43469-1	Specimen	Aegilops triuncialis L.	L.	ES	Spain	Granada, Guadix, Rambla Becerra.		GR			990
00:00.0	GDAC	GDAC26140-1	Specimen	Aegilops triuncialis L.	L.	ES	Spain	Granada, SÂª de Baza, Rambla del		GR			0
	BC	92745	Specimen	Aegilops triuncialis L.	L.	ES	Spain	Vallfogona de Riucorb; Vallfogona d		T	41.58	1.26	
	SIVIM	T-P16889:Aegi	Observation	Aegilops triuncialis L.	L.	ES	Spain	ValdepiÃ©lagos			40.73	-3.47	0
	SIVIM	T-P16916:Aegi	Observation	Aegilops triuncialis L.	L.	ES	Spain	Ciudad Universitaria			40.37	-3.7	0
00:00.0	COFC	7351-1	Specimen	Aegilops triuncialis L.	L.	ES	Spain	Valle del Guadalmellato; puente Mo		Co			1
	FUND. BIODIVER	120463	Unknown	Aegilops triuncialis L.	L.	ESP	Spain	Valdevacas de Montejo, Valdevacas		Sg	41.1	-3.1	
	FUND. BIODIVER	72902	Unknown	Aegilops triuncialis L.	L.	ESP	Spain	DoÃ±inos de Salamanca		Sa	40.1	-5.1	
	FUND. BIODIVER	86604	Unknown	Aegilops triuncialis L.	L.	ESP	Spain	Aldehuela de PeriÃ¡Ã±ez, hacia Alm		So	41.1	-2.1	
00:00.0	UNEX	10359-1	Observation	Aegilops triuncialis L.	_	ESP	Spain	Sierra de Oliva. 29SQC49		Ba	38.7	-6.2	
	IDBD-GN	42520	Observation	Aegilops triuncialis L.	L.	ES	Spain	Balsa del Juncal	Tafalla	Na	42.5107	-1.70494	420
00:00.0	REDIAM-CMA	80027	Observation	Aegilops triuncialis		ESP	Spain		Yunquera	Ma	36.7086	-4.96757	1101
00:00.0	REDIAM-CMA	99714	Observation	Aegilops triuncialis		ESP	Spain		AndÃºjar	J	38.3204	-4.00726	691
00:00.0	BC	70833	Specimen	Aegilops triuncialis L.	L.	ES	Spain	Burgos; CastaÃ±ares, pr. Burgos		Bu	42.32	-3.67	
	SIVIM	T-P10780:Aegi	Observation	Aegilops triuncialis L.	L.	ES	Spain	Moreruela de TÃ¡bara			41.78	-5.88	0
	SIVIM	T-P12097:Aegi	Observation	Aegilops triuncialis L.	L.	ES	Spain	Camposagrado			42.68	-5.8	1170
	SANT	48205	Specimen	Aegilops triuncialis L.		ES	Spain	SabiÃ±Ã¡nigo. Entre Lasaosa y Noc		Hu			
	FUND. BIODIVER	1115798	Unknown	Aegilops triuncialis L.	L.	ESP	Spain	Pozuelo de TÃ¡bara		Za	41.1	-5.1	
	FUND. BIODIVER	1120226	Unknown	Aegilops triuncialis L.	L.	ESP	Spain	Abezames, Gafos		Za	41.1	-5.1	
00:00.0	FUND. BIODIVER	1946451	Unknown	Aegilops triuncialis L.	L.	ESP	Spain	Piedrabuena, volcan de Piedrabuen		CR	38.1	-3.1	600
00:00.0	COA	41146-1	Specimen	Aegilops triuncialis L.	L.	ES	Spain	Km 7 de Villa del RÃ-o a Bujalance		Co	37.94	-4.37	
	CZE122	01C2107133	Specimen	Aegilops triuncialis subsp. triuncialis var. flaves		ESP	Spain	Huelma Andalusien, Spanien					
00:00.0	SEV	99217-1	Specimen	Aegilops triuncialis L.	L.	ES	Spain	Los Barrios		Ca			160
00:00.0	REDIAM-CMA	423634	Observation	Aegilops triuncialis		ESP	Spain		El Guijo	Co	38.5254	-4.81537	504
00:00.0	BC	641201	Specimen	Aegilops triuncialis L.	L.	ES	Spain	Loscos; Calamocha: roquedos junto		Te	41.073	-1.125	1070
00:00.0	BC	866349	Specimen	Aegilops triuncialis L.	L.	ES	Spain	Fuente Alamo; Al peu de Carrascoy		Mu	37.8453	-1.29499	
00:00.0	BC	601441	Specimen	Aegilops triuncialis L.	L.	ES	Spain	Prades; Muntanyes de Prades: Plan		T	41.31	1.03	1100
	SIVIM	T-P06961:Aegi	Observation	Aegilops triuncialis L.	L.	ES	Spain	ArdÃ³n			42.42	-5.67	0

	SIVIM	T-P09157:Aegi	Observation	Aegilops triuncialis L.	L.	ES	Spain	Portillo			41.44	-4.67	0
00:00.0	ABH	10692-1	Specimen	Aegilops triuncialis L.	L.	ES	Spain	Villena; Arenal de la Virgen		A	38.61	-0.94	
	FUND. BIODIVER	904085	Unknown	Aegilops triuncialis L.	L.	ESP	Spain	Barcelona		B	41.1	2.1	
00:00.0	BC	839370	Specimen	Aegilops triuncialis L.	L.	ES	Spain	Salvatierra de Esca; Mte Salvatierra		Z	42.735	-0.917	900
00:00.0	SEV	108995-1	Specimen	Aegilops triuncialis L.	L.	ES	Spain	Near Coin		Ma			360
00:00.0	SEV	55682-1	Specimen	Aegilops triuncialis L.	L.	ES	Spain	Laujar de Andarex, nacimiento, num		Al			900
00:00.0	SALA	50161-1	Specimen	Aegilops triuncialis L.	L.	ES	Spain	_; La Aliseda, DespeÃ±aperros		J			
00:00.0	REDIAM-CMA	132854	Observation	Aegilops triuncialis		ESP	Spain		Constantina	Se	37.905	-5.72682	387
00:00.0	REDIAM-CMA	149094	Observation	Aegilops triuncialis		ESP	Spain		Santiago-Pontone	J	38.0491	-2.64878	1595
00:00.0	REDIAM-CMA	183844	Observation	Aegilops triuncialis		ESP	Spain		Montoro	Co	38.1276	-4.37502	300
	SIVIM	T-P16895:Aegi	Observation	Aegilops triuncialis L.	L.	ES	Spain	Las Rozas			40.46	-3.94	0
	SIVIM	T-P16919:Aegi	Observation	Aegilops triuncialis L.	L.	ES	Spain	Ciudad Universitaria			40.37	-3.7	0
00:00.0	COA	41150-1	Specimen	Aegilops triuncialis L.	L.	ES	Spain	4 Km al norte de Hornachuelos		Co	37.84	-5.27	
00:00.0	MGC	60701-1	Unknown	Aegilops triuncialis L.	L.	ES	Spain	Genalguacil; Los Zaharames		Ma	36.525	-5.262	150
00:00.0	COA	41147-1	Specimen	Aegilops triuncialis L.	L.	ES	Spain	San Calixto		Co	37.92	-5.39	
00:00.0	SEV	101356-1	Specimen	Aegilops triuncialis L.	L.	ES	Spain	Ente Torrecampo y San Benito. RÃ-		Co			1
00:00.0	SEV	106435-1	Specimen	Aegilops triuncialis L.	L.	ES	Spain	Coripe, Sierra		Se			1
00:00.0	REDIAM-CMA	280193	Observation	Aegilops triuncialis		ESP	Spain		Adamuz	Co	38.0204	-4.52593	211
00:00.0	GDA	GDA12184-1-1	Specimen	Aegilops triuncialis L.	L.	ES	Spain	Granada, Lobras, falda del cerro Ve		GR			800
00:00.0	GDA	GDA15569-1-2	Specimen	Aegilops triuncialis L.	L.	ES	Spain	Granada, SÂª de Madrid, Lagunazo		GR			1200
	SIVIM	T-P28626:Aegi	Observation	Aegilops triuncialis L.	L.	ES	Spain	Arroyo de la Vid, TorrejÃ³n el Rubio			39.71	-6.03	300
	SIVIM	T-P30049:Aegi	Observation	Aegilops triuncialis L.	L.	ES	Spain	Pr. Arenal de Las Salinas, Casas de Ves, A			39.28	-1.37	0
00:00.0	COFC	46903-1	Specimen	Aegilops triuncialis L.	L.	ES	Spain	Palma del RÃƒÂ-o; ctra de Hornach		Co			1
	FUND. BIODIVER	101227	Unknown	Aegilops triuncialis L.	L.	ESP	Spain	Quintanas de Gormaz, Hacia Valde		So	41.1	-2.1	950
00:00.0	FUND. BIODIVER	1774964	Unknown	Aegilops triuncialis L.	L.	ESP	Spain	Almunia de San Juan, Ariestolas, rit		Hu	41.1	-0.9	270
00:00.0	SEV	59042-1	Specimen	Aegilops triuncialis L.	L.	ES	Spain	Algodonales, Sierra de LÃ-jar		Ca			500
00:00.0	MA	680840-1	Specimen	Aegilops triuncialis L.	L.	ES	Spain	SerrejÃ³n. 'El Pizarral'		Cc			
00:00.0	MGC	44023-1	Unknown	Aegilops triuncialis L.	L.	ES	Spain	Estepona; Sierra Bermeja. Cara sur		Ma	0	0	250
00:00.0	SEV	49882-1	Specimen	Aegilops triuncialis L.	L.	ES	Spain	Higuera de la Sierra		H			1
00:00.0	FUND. BIODIVER	1835118	Unknown	Aegilops triuncialis L.	L.	ESP	Spain	Fuencaliente, confluencia de los rio		CR	38.1	-2.1	1000
00:00.0	FUND. BIODIVER	1835139	Unknown	Aegilops triuncialis L.	L.	ESP	Spain	San Lorenzo de Calatrava, Los Cas		CR	38.1	-3.1	800
00:00.0	COFC	46900-1	Specimen	Aegilops triuncialis L.	L.	ES	Spain	arroyo Guadalbaida; ctra hacia Sevi		Co			1
	IPK	AE 1057	Living	Aegilops triuncialis L.			Spain	Beira, Alta, Barca de Alva			41.0264	-6.05889	
	IPK	AE 1316	Living	Aegilops triuncialis L.			Spain	Baira Alta, Barca de Alva			41.0264	-6.05889	
00:00.0	REDIAM-CMA	388625	Observation	Aegilops triuncialis		ESP	Spain		Torres	J	37.7471	-3.52847	1500
00:00.0	REDIAM-CMA	394672	Observation	Aegilops triuncialis		ESP	Spain		Chiclana de Segu	J	38.3708	-2.94946	780
00:00.0	REDIAM-CMA	364593	Observation	Aegilops triuncialis		ESP	Spain		Turre	Al	37.1351	-1.94038	100
	IPK	AE 1051	Living	Aegilops triuncialis L.			Spain	Puerto Real (Cadiz)			36.5331	-6.81667	
	SIVIM	U-P13530:Aegi	Observation	Aegilops triuncialis L.	L.	ES	Spain	Macedo, Morais, junto ao estradÃ£o para F			41.44	-6.84	0
	SIVIM	P-P08869:Aegi	Observation	Aegilops triuncialis L.	L.	ES	Spain	Els Garrigons; Beseit			40.79	0.15	0
	SIVIM	U-P09954:Aegi	Observation	Aegilops triuncialis L.	L.	ES	Spain	Carretera de Ossa a Ruidera			38.93	-2.76	0

	RUS001	VIR100602176	Specimen	Aegilops triuncialis L.		ESP	Spain						
	RUS001	VIR100602154	Specimen	Aegilops triuncialis L.		ESP	Spain						
00:00.0	MA	756580-1	Specimen	Aegilops triuncialis L.	L.	ES	Spain	Aguilafuente, alrededores del arene		Sg	41	-4	
	FUND. BIODIVER	1043773	Unknown	Aegilops triuncialis L.	L.	ESP	Spain	Carratraca, Sierra de Aguas		Ma	36.1	-4.1	
00:00.0	FUND. BIODIVER	1835124	Unknown	Aegilops triuncialis L.	L.	ESP	Spain	Piedrabuena, cunetas de la carreter		CR	39.1	-4.1	700
00:00.0	FUND. BIODIVER	1835145	Unknown	Aegilops triuncialis L.	L.	ESP	Spain	Almagro, volcan de Yezosa, sobre e		CR	38.1	-3.1	853
	GDA	GDA42723-1-1	Specimen	Aegilops triuncialis L.	L.	ES	Spain	Granada, Deifontes, base de la Sier		GR			800
	SIVIM	U-P13537:Aegi	Observation	Aegilops triuncialis L.	L.	ES	Spain	Braganga, SÃ©, junto ao campo da aviaÃ§			41.8	-6.83	0
00:00.0	FUND. BIODIVER	1369187	Unknown	Aegilops triuncialis L.	L.	ESP	Spain	Lodares de Medinaceli		So	41.1	-2.1	900
	ESP003	NC061596	Specimen	Aegilops triuncialis L.		ESP	Spain	Puerto Real, province of Cadiz			36.5167	-6.18333	10
	ESP004	NC043495	Specimen	Aegilops triuncialis L.		ESP	Spain	Encinas de Esgueva, province of Valladolid			41.75	-4.1	832
00:00.0	COFC	36337-1	Specimen	Aegilops triuncialis L.	L.	ES	Spain	Puente Genil; embalse de la Cordob		Co			1
	FUND. BIODIVER	1135156	Unknown	Aegilops triuncialis L.	L.	ESP	Spain	Castrovega de Valmadrigal		Le	42.1	-5.1	
00:00.0	FUND. BIODIVER	1946462	Unknown	Aegilops triuncialis L.	L.	ESP	Spain	Alhambra, saladar en arroyo de Los		CR	38.1	-2.1	
00:00.0	REDIAM-CMA	167757	Observation	Aegilops triuncialis		ESP	Spain		Villanueva del Re	Co	38.1813	-5.24509	715
00:00.0	BC	96444	Specimen	Aegilops triuncialis L.	L.	ES	Spain	Barcelona; pr. St Medi. Barcelona		B	41.41	2.1	
	SIVIM	T-P16879:Aegi	Observation	Aegilops triuncialis L.	L.	ES	Spain	HÃºmera			40.37	-3.82	0
	SIVIM	T-P16909:Aegi	Observation	Aegilops triuncialis L.	L.	ES	Spain	Fresneda de Allarejos			39.92	-2.41	0
00:00.0	REDIAM-CMA	48643	Observation	Aegilops triuncialis		ESP	Spain		El Castillo de las	Se	37.6378	-6.31084	300
	ESP004	NC043486	Specimen	Aegilops triuncialis L.		ESP	Spain	Retiendas, province of Guadalajara			40.9667	-3.26667	895
	SIVIM	S-P14069:Aegi	Observation	Aegilops triuncialis L.	L.	ES	Spain	Almendres			42.99	-3.49	295
	SIVIM	T-P06421:Aegi	Observation	Aegilops triuncialis L.	L.	ES	Spain	Siete Aguas			39.46	-1.02	0
	SIVIM	T-P09350:Aegi	Observation	Aegilops triuncialis L.	L.	ES	Spain	TorrebeleÃ±a			40.83	-3.23	0
	FUND. BIODIVER	1339637	Unknown	Aegilops triuncialis L.	L.	ESP	Spain	Albacete, provincia		Ab	38.1	-2.1	
00:00.0	FUND. BIODIVER	1348608	Unknown	Aegilops triuncialis L.	L.	ESP	Spain	Piedrabuena		CR	39.1	-4.1	550
	FUND. BIODIVER	70872	Unknown	Aegilops triuncialis L.	L.	ESP	Spain	Monterrubio de ArmuÃ±a		Sa	40.1	-5.1	
	FUND. BIODIVER	80277	Unknown	Aegilops triuncialis L.	L.	ESP	Spain	Cantalejo, La MuÃ±a		Sg	41.1	-3.1	
00:00.0	GDA	GDA16061-1-1	Specimen	Aegilops triuncialis L.	L.	ES	Spain	Granada, SÂª de Madrid, antes del		GR			1200
00:00.0	REDIAM-CMA	83204	Observation	Aegilops triuncialis		ESP	Spain		Arroyo del Ojanco	J	38.3019	-2.85046	949
	SIVIM	T-P10773:Aegi	Observation	Aegilops triuncialis L.	L.	ES	Spain	Dehesa de Misleo (Moreruela de TÃ¡bara)			41.78	-5.88	0
1862-07-0	W	43488	Unknown	Aegilops triuncialis L.		ESP	Spain	Paturages arides de Casa del Campo pres de Madrid.					
00:00.0	BC	857424	Specimen	Aegilops triuncialis L.	L.	ES	Spain	Torija; bajo Torija		Gu	40.728	-3.041	900
00:00.0	BC	144252	Specimen	Aegilops triuncialis L.	L.	ES	Spain	Sant Mateu de Bages; ad ripas fl. C		B	41.77	1.62	
	RUS001	VIR100602130	Specimen	Aegilops triuncialis L.		ESP	Spain						
	RUS001	VIR100602087	Specimen	Aegilops triuncialis L.		ESP	Spain						
	FUND. BIODIVER	1084636	Unknown	Aegilops triuncialis L.	L.	ESP	Spain	Vaciamadrid		M	40.1	-3.1	
	FUND. BIODIVER	1090314	Unknown	Aegilops triuncialis		ESP	Spain	CobeÃ±a		M	40.1	-3.1	
	FUND. BIODIVER	1925491	Unknown	Aegilops triuncialis L.	L.	ESP	Spain	Sigues, Venta Carrica		Z	42.1	-0.1	550
00:00.0	FUND. BIODIVER	1946441	Unknown	Aegilops triuncialis L.	L.	ESP	Spain	Malagon-Los Quiles, olivos		CR	39.1	-3.1	
	BC	608752	Specimen	Aegilops triuncialis L.	L.	ES	Spain	Barcelona; prope Barcinonem		B	41.41	2.1	
00:00.0	REDIAM-CMA	25357	Observation	Aegilops triuncialis		ESP	Spain		AlbÃ¡nchez de M	J	37.7873	-3.48675	1200

00:00.0	REDIAM-CMA	412049	Observation	Aegilops triuncialis		ESP	Spain		La Granada de R	H	37.7567	-6.48868	507
	SIVIM	S-P02157:Aegi	Observation	Aegilops triuncialis L.	L.	ES	Spain	Les PLanes de CastellÃ³ de Farfanya			41.79	0.71	0
00:00.0	SALA	97516-1	Specimen	Aegilops triuncialis L.	L.	ES	Spain	_; Hecho, EmbÃºn, convento del Pil		Hu	42.61	-0.73	
	UNEX	10358-1	Observation	Aegilops triuncialis L.	_	ESP	Spain	Badajoz: La Orden. 29SQD00		Ba	38.8	-6.7	
00:00.0	MA	753632-1	Specimen	Aegilops triuncialis L.	L.	ES	Spain	Barranco de las Gayombas		Gr			
	RUS001	VIR100602220	Specimen	Aegilops triuncialis L.		ESP	Spain						
	RUS001	VIR100602177	Specimen	Aegilops triuncialis L.		ESP	Spain						
	RUS001	VIR100602155	Specimen	Aegilops triuncialis L.		ESP	Spain						
	FUND. BIODIVER	1035582	Unknown	Aegilops triuncialis		ESP	Spain	Zonas elevadas de la Sierra de Agu		Ma	36.1	-4.1	
	FUND. BIODIVER	104260	Unknown	Aegilops triuncialis L.	L.	ESP	Spain	CastrejÃ³n de la PeÃ±a, entre Castr		P	42.1	-4.1	
	FUND. BIODIVER	1043772	Unknown	Aegilops triuncialis L.	L.	ESP	Spain	Canillas de Albaida		Ma	36.1	-3.1	
	FUND. BIODIVER	1835123	Unknown	Aegilops triuncialis L.	L.	ESP	Spain	intercalacion basaltica del Alamillo		CR	38.1	-4.1	
00:00.0	FUND. BIODIVER	1835144	Unknown	Aegilops triuncialis L.	L.	ESP	Spain	Villanueva de los Infantes, rio Jabal		CR	38.1	-2.1	820
00:00.0	REDIAM-CMA	392064	Observation	Aegilops triuncialis		ESP	Spain		Cabra	Co	37.4929	-4.37575	1000
	BC	92727	Specimen	Aegilops triuncialis L.	L.	ES	Spain	Chiva; Chiva		V	39.5	-0.73	
	SIVIM	U-P13536:Aegi	Observation	Aegilops triuncialis L.	L.	ES	Spain	Vinhais, Vila Verde, estradÃ£o para o Mon			41.8	-6.95	0
00:00.0	HUAL	1139-1	Specimen	Aegilops triuncialis L.	L.	ES	Spain	SÂª del Pozo, prÃ³x. Nava de San P		J	37.889	-2.881	
00:00.0	UNEX	10356-1	Observation	Aegilops triuncialis L.	_	ESP	Spain	Robledillo de la Vera: Garganta de		Cc	40	-5.6	
00:00.0	HSS	2630	Specimen	Aegilops triuncialis L.	L.	ES	Spain	Almendral, El Calerizos		Ba	38.6021	-6.76099	
	ESP004	NC043485	Specimen	Aegilops triuncialis L.		ESP	Spain	isla Mallorca, Soller, province of Baleares			39.75	2.7	41
00:00.0	FUND. BIODIVER	1348609	Unknown	Aegilops triuncialis L.	L.	ESP	Spain	Piedrabuena, carretera entre Arroba		CR	38.1	-4.1	700
	FUND. BIODIVER	70873	Unknown	Aegilops triuncialis L.	L.	ESP	Spain	San CristÃ³bal de la Cuesta		Sa	40.1	-5.1	
00:00.0	GDAC	GDAC41208-1	Specimen	Aegilops triuncialis L.	L.	ES	Spain	CÃ³rdoba, SÂª Morena, CardeÃ±a.		CO			740
00:00.0	BC	70839	Specimen	Triticum triunciale (L.) Raspail	(L.) Raspail	ES	Spain	San Pablo de los Montes; in Castell		To	39.61	-4.34	
	SIVIM	T-P11862:Aegi	Observation	Aegilops triuncialis L.	L.	ES	Spain	El Portillo			42.51	-5.55	0
	SIVIM	T-P15403:Aegi	Observation	Aegilops triuncialis L.	L.	ES	Spain	Castrovega del Valmadrigal			42.24	-5.3	0
	ESP004	NC050478	Specimen	Aegilops triuncialis L.		ESP	Spain	Valdemorillo Sierra, province of Cuenca			40.0333	-1.76667	1211
	FUND. BIODIVER	1120227	Unknown	Aegilops triuncialis L.	L.	ESP	Spain	Granja de Moreruela, Los Coloraos		Za	41.1	-5.1	
00:00.0	FUND. BIODIVER	1946452	Unknown	Aegilops triuncialis L.	L.	ESP	Spain	Solana del Pino, Alhorin		CR	38.1	-3.1	660
	FUND. BIODIVER	36853	Unknown	Aegilops triuncialis L.	L.	ESP	Spain	Elciego		Vi	42.1	-2.1	450
	FUND. BIODIVER	51242	Unknown	Aegilops triuncialis L.	L.	ESP	Spain	Villarino		Sa	41.1	-6.1	
00:00.0	FUND. BIODIVER	1743189	Unknown	Aegilops triuncialis L.	L.	ESP	Spain	Ciruelos de Cervera, pie del alto de		Bu	41.1	-3.1	1100
	FUND. BIODIVER	992692	Unknown	Aegilops triuncialis L.	L.	ESP	Spain	Turmiel		Gu	40.1	-1.1	
	FUND. BIODIVER	998074	Unknown	Aegilops triuncialis		ESP	Spain	Cuenca		Cu	39.1	-1.1	
	SIVIM	T-P06093:Aegi	Observation	Aegilops triuncialis L.	L.	ES	Spain	de Venta el CastaÃ±o, en las Yeseras			37.04	-2.21	260
	SIVIM	T-P06962:Aegi	Observation	Aegilops triuncialis L.	L.	ES	Spain	Ardoncino			42.42	-5.67	0
	SIVIM	P-P08852:Aegi	Observation	Aegilops triuncialis L.	L.	ES	Spain	Riu Matarranya; Beseit			40.79	0.15	0
	SIVIM	U-P09884:Aegi	Observation	Aegilops triuncialis L.	L.	ES	Spain	Erial entre Ossa y Villahermosa			38.66	-2.88	0
00:00.0	COFC	46909-1	Specimen	Aegilops triuncialis L.	L.	ES	Spain	arroyo anterior al rÃo NÃ©valo		Co			1
	SIVIM	T-P19968:Aegi	Observation	Aegilops triuncialis L.	L.	ES	Spain	Finca de Valdelasyeguas (Aliseda)			39.36	-6.79	0
	FUND. BIODIVER	1001948	Unknown	Aegilops triuncialis		ESP	Spain	Sierra de San Felipe		Cu	40.1	-1.1	

	FUND. BIODIVER	1012904	Unknown	Aegilops triuncialis L.	L.	ESP	Spain	Turre		Al	36.1	-1.1	
	FUND. BIODIVER	1024008	Unknown	Aegilops triuncialis L.	L.	ESP	Spain	DespeÃ±aperros		J	38.1	-3.1	
00:00.0	MA	596985-1	Specimen	Aegilops triuncialis L.	L.	ES	Spain	Solana del Pino, AlharÃ-n		CR	38	-4	
00:00.0	GDA	GDA30026-1-1	Specimen	Aegilops triuncialis L.	L.	ES	Spain	Granada, Alhambra, Cementerio.		GR			0
	SIVIM	P-P12756:Aegi	Observation	Aegilops triuncialis L.	L.	ES	Spain	Carretera de Santa MarÃ-a de Nieva			37.49	-2.09	1090
	ESP004	NC024050	Specimen	Aegilops triuncialis L.		ESP	Spain	Alcornoquillo, Alhama de Granada, provinc			36.9333	-4.01667	1078
00:00.0	COA	41145-1	Specimen	Aegilops triuncialis L.	L.	ES	Spain	Cruce carretera Montoro, CardeÃ±a		Co	38.21	-4.37	
	FUND. BIODIVER	1340022	Unknown	Aegilops triuncialis L.	L.	ESP	Spain	Madrid, Campus Universitario Monc		M	39.1	-3.1	
	FUND. BIODIVER	77473	Unknown	Aegilops triuncialis L.	L.	ESP	Spain	Puebla de Sanabria		Za	41.1	-6.1	
1872-06-0	BC	627486	Specimen	Aegilops triuncialis L.	L.	ES	Spain	Bellmunt del Priorat; Llera eixuta de		T	41.13	0.8	100
	IPK	32374	Living	Aegilops triuncialis L. subsp. triuncialis var. triu		ESP	Spain	Finca La Cigue?ela, ostlich Aracena, Huelva					
00:00.0	HSS	13774	Specimen	Aegilops triuncialis L.	L.	ES	Spain	Alconera, Puerto de Calatrava		Ba	38.4149	-6.42321	500
00:00.0	REDIAM-CMA	93162	Observation	Aegilops triuncialis		ESP	Spain		Cabra	Co	37.4916	-4.37523	1000
00:00.0	COA	41188-1	Specimen	Aegilops triuncialis L.	L.	ES	Spain	Km 4 de BelalcÃ¡zar a Hinojosa		Co	38.47	-5.18	
	SIVIM	T-P10774:Aegi	Observation	Aegilops triuncialis L.	L.	ES	Spain	Faramontanos de TÃ¡bara			41.78	-5.88	0
	SIVIM	T-P11863:Aegi	Observation	Aegilops triuncialis L.	L.	ES	Spain	MatadeÃ³n de los Oteros			42.33	-5.42	0
	SIVIM	T-P15430:Aegi	Observation	Aegilops triuncialis L.	L.	ES	Spain	Chozas de Abajo			42.5	-5.79	0
00:00.0	SEV	5993-1	Specimen	Aegilops triuncialis L.	L.	ES	Spain	Aldeaquemada		J			1
00:00.0	BC	124056	Specimen	Aegilops triuncialis L.	L.	ES	Spain	San Fernando; Baetica: La Carraca		Ca	36.52	-6.15	
00:00.0	SEV	98909-1	Specimen	Aegilops triuncialis L.	L.	ES	Spain	El Vacar. Barranco del RÃ-o Guada		Co			1
00:00.0	SEV	99071-1	Specimen	Aegilops triuncialis L.	L.	ES	Spain	SerranÃ-a de Ronda. Sierra de las I		Ma			900
00:00.0	MGC	67085-1	Unknown	Aegilops triuncialis L.	L.	ES	Spain	Ciudad Universitaria		M	0	0	1
	SALA	1563-1	Specimen	Aegilops triuncialis L.	L.	ES	Spain	_; Madrid, campos de la Moncloa		M			
00:00.0	GDA	GDA10270-1-2	Specimen	Aegilops triuncialis L.	L.	ES	Spain	Granada, AlmegÃ-jar, prÃ³x. barran		GR			900
00:00.0	FCO	17836-1	Specimen	Aegilops triuncialis L.	L.	ES	Spain	Pinar de Selas		Gu	40.94	-2.13	
00:00.0	FUND. BIODIVER	1369193	Unknown	Aegilops triuncialis L.	L.	ESP	Spain	Los RÃ¡banos, La Carrascosa		So	41.1	-2.1	1000
00:00.0	COFC	41256-1	Specimen	Aegilops triuncialis L.	L.	ES	Spain	entre Cabra y Nueva Carteya		Co	37	-4	1
	SIVIM	T-P16882:Aegi	Observation	Aegilops triuncialis L.	L.	ES	Spain	Ribatejada			40.64	-3.47	0
	SIVIM	T-P16912:Aegi	Observation	Aegilops triuncialis L.	L.	ES	Spain	JÃ¡baga			40.01	-2.29	0
00:00.0	ABH	10172-1	Specimen	Aegilops triuncialis L.	L.	ES	Spain	Petrer; prox. los Castellerets		A	38.49	-0.73	
	DEU146	AE 784	Specimen	Aegilops triuncialis subsp. triuncialis var. const		ESP	Spain	Fuencaliente (Soria)					
	BC	96440	Specimen	Aegilops triuncialis L.	L.	ES	Spain	Barcelona; St Geroni de la Vall d'He		B	41.41	2.1	
00:00.0	UNEX	30123-1	Observation	Aegilops triuncialis L.	_	ESP	Spain	Magacela: Pastizal ruderalizado sob		Ba	38.8	-5.8	
	SANT	16351	Specimen	Aegilops triuncialis L.		ES	Spain	RubiÃ¡. Cobas		Or			
00:00.0	ABH	31246-1	Specimen	Aegilops triuncialis L.	L.	ES	Spain	Petrel; El Arenal		A	38.51	-0.77	
00:00.0	BC	857419	Specimen	Aegilops triuncialis L.	L.	ES	Spain	Algora; Algora		Gu	40.961	-2.638	1050
00:00.0	FUND. BIODIVER	1369191	Unknown	Aegilops triuncialis L.	L.	ESP	Spain	Valdenarros		So	41.1	-2.1	950
00:00.0	BC	100851	Specimen	Aegilops triuncialis L.	L.	ES	Spain	Montcada i Reixac; Montcada pr de		B	41.5	2.22	
00:00.0	REDIAM-CMA	141831	Observation	Aegilops triuncialis		ESP	Spain		Zufre	H	37.8787	-6.39073	323
00:00.0	REDIAM-CMA	147853	Observation	Aegilops triuncialis		ESP	Spain		Santa Olalla del C	H	37.8981	-6.21523	500
	GDA	GDA43469-1-1	Specimen	Aegilops triuncialis L.	L.	ES	Spain	Granada, Guadix, Rambla Becerra.		GR			990

	SIVIM	T-P16881:Aegi	Observation	Aegilops triuncialis L.	L.	ES	Spain	Colmenar Viejo			40.64	-3.82	0
	SIVIM	T-P16911:Aegi	Observation	Aegilops triuncialis L.	L.	ES	Spain	TamajÃ³n			40.92	-3.35	0
00:00.0	COFC	41144-1	Specimen	Aegilops triuncialis L.	L.	ES	Spain	Lucena; rÃƒÂ-o Anzur; entre el mun		Co	37	-4	1
	ESP004	NC050486	Specimen	Aegilops triuncialis L.		ESP	Spain	Las Majadas/Beteta 8km NE, Cuenca, prov			40.35	-2	1210
	MA	573625-1	Specimen	Aegilops triuncialis L.	L.	ES	Spain	Moncloa		M			
00:00.0	FUND. BIODIVER	1946466	Unknown	Aegilops triuncialis L.	L.	ESP	Spain	Alcubillas, alrededores		CR	38.1	-2.1	830
00:00.0	BC	835718	Specimen	Aegilops triuncialis L.	L.	ES	Spain	Hecho; Hecho, EmbÃºn, convento d		Hu	42.615	-0.726	680
00:00.0	MA	719813-1	Specimen	Aegilops triuncialis L.	L.	ES	Spain	En los contornos de Madrid		M			
	SIVIM	S-P09958:Aegi	Observation	Aegilops triuncialis L.	L.	ES	Spain	font de l'Espinal, serra Aitana (Alcoleja)			38.63	-0.35	1150
	SIVIM	T-P05776:Aegi	Observation	Aegilops triuncialis L.	L.	ES	Spain	HondÃ³n de las Nieves			38.19	-0.94	0
	SIVIM	T-P06427:Aegi	Observation	Aegilops triuncialis L.	L.	ES	Spain	Casas de Madrona			38.92	-1.15	0
00:00.0	COA	41156-1	Specimen	Aegilops triuncialis L.	L.	ES	Spain	Km 5 al sur de CardeÃ±a		Co	38.21	-4.37	
	BC	92753	Specimen	Aegilops triuncialis L.	L.	ES	Spain	Puerto Real; Calerones. S. Fernand		Ca	36.51	-6.04	
	BC	140329	Specimen	Aegilops triuncialis L.	L.	ES	Spain	BenicÃ ssim; Regno valentino: VilÂ		Cs	40.03	0.01	
	IDBD-GN	42516	Observation	Aegilops triuncialis L.	L.	ES	Spain		Petilla de AragÃ³r	Na	42.4474	-1.04961	1000
	ESP004	NC043494	Specimen	Aegilops triuncialis L.		ESP	Spain	Molino de la Villa, Cantalapiedra, province			41.15	-5.16667	785
	SANT	20746	Specimen	Aegilops triuncialis L.		ES	Spain	RubiÃ¡,Cobas;en la estaciÃ³n de fer		Or			
	FUND. BIODIVER	1141568	Unknown	Aegilops triuncialis L.	L.	ESP	Spain	VillamaÃ±Ã¡n		Le	42.1	-5.1	760
00:00.0	FUND. BIODIVER	1946463	Unknown	Aegilops triuncialis L.	L.	ESP	Spain	Albadalejo		CR	38.1	-2.1	900
	FUND. BIODIVER	47603	Unknown	Aegilops triuncialis L.	L.	ESP	Spain	CastronuÃ±o, Las Cocineras, Valde		Va	41.1	-5.1	
00:00.0	ABH	11698-1	Specimen	Aegilops triuncialis L.	L.	ES	Spain	San Juan;		A	38.37	-0.48	
00:00.0	REDIAM-CMA	266897	Observation	Aegilops triuncialis		ESP	Spain		Villaviciosa de CÃ	Co	38.0859	-4.97367	549
00:00.0	REDIAM-CMA	281766	Observation	Aegilops triuncialis		ESP	Spain		BelalcÃ¡zar	Co	38.5204	-5.05934	526
00:00.0	REDIAM-CMA	43483	Observation	Aegilops triuncialis		ESP	Spain		El Castillo de las	Se	37.6565	-6.22937	215
00:00.0	GDAC	GDAC21286-1	Specimen	Aegilops triuncialis L.	L.	ES	Spain	Madrid, Embalse de Santillana.		M			0
	SIVIM	T-P27383:Aegi	Observation	Aegilops triuncialis L.	L.	ES	Spain	Almoguera			40.29	-3	0
	SIVIM	T-P28630:Aegi	Observation	Aegilops triuncialis L.	L.	ES	Spain	Castillo de Mirabel			39.8	-6.31	0
	SIVIM	T-P28915:Aegi	Observation	Aegilops triuncialis L.	L.	ES	Spain	Saltos de TorrejÃ³n			39.71	-6.2	0
	SIVIM	T-P30051:Aegi	Observation	Aegilops triuncialis L.	L.	ES	Spain	Pr. Hazas del Calvario, Casas IbÃ¡Ã±ez, A			39.19	-1.49	0
	SIVIM	S-P14070:Aegi	Observation	Aegilops triuncialis L.	L.	ES	Spain	Almendres			42.99	-3.49	300
	SIVIM	T-P06424:Aegi	Observation	Aegilops triuncialis L.	L.	ES	Spain	Cortes de PallÃ¡s			39.19	-1.03	340
	SIVIM	T-P09351:Aegi	Observation	Aegilops triuncialis L.	L.	ES	Spain	TamajÃ³n			40.92	-3.35	1000
	MA	719927-1	Specimen	Aegilops triuncialis L.	L.	ES	Spain	Se cria en la pradera del canal y otr		M			
	FUND. BIODIVER	120462	Unknown	Aegilops triuncialis L.	L.	ESP	Spain	Valdevacas de Montejo, Valdevacas		Sg	41.1	-3.1	
	FUND. BIODIVER	72901	Unknown	Aegilops triuncialis L.	L.	ESP	Spain	Salamanca, Tejares		Sa	40.1	-5.1	
00:00.0	REDIAM-CMA	125287	Observation	Aegilops triuncialis		ESP	Spain		Aracena	H	37.8673	-6.46354	643
	SIVIM	T-P10779:Aegi	Observation	Aegilops triuncialis L.	L.	ES	Spain	Moreruela de TÃ¡bara			41.78	-5.88	0
	SIVIM	T-P12079:Aegi	Observation	Aegilops triuncialis L.	L.	ES	Spain	Lorenzana			42.6	-5.68	0
1876-06-0	W	43349	Unknown	Aegilops triuncialis L.		ESP	Spain	Hispania: Prope El Escorial. In itinere hispanico-lusitanico.					
00:00.0	GDA	GDA15569-1	Specimen	Aegilops triuncialis L.	L.	ES	Spain	Granada, SÂª de Madrid, Lagunazo		GR			1200
00:00.0	ABH	23286-1	Specimen	Aegilops triuncialis L.	L.	ES	Spain	Monforte del Cid; Sierra del Cid		A	38.38	-0.82	

	FUND. BIODIVER	1135164	Unknown	Aegilops triuncialis L.	L.	ESP	Spain	Puente Castro		Le	42.1	-5.1	910
	FUND. BIODIVER	51170	Unknown	Aegilops triuncialis L.	L.	ESP	Spain	Masueco		Sa	41.1	-6.1	
00:00.0	COFC	46907-1	Specimen	Aegilops triuncialis L.	L.	ES	Spain	El Cabril; cementerio nuclear; 'La R		Co			1
	FUND. BIODIVER	1019469	Unknown	Aegilops triuncialis L.	L.	ESP	Spain	Pinos Genil		Gr	36.1	-3.1	
00:00.0	REDIAM-CMA	45858	Observation	Aegilops triuncialis		ESP	Spain		Posadas	Co	37.8962	-5.13781	299
00:00.0	REDIAM-CMA	365763	Observation	Aegilops triuncialis		ESP	Spain		PulpÃ-	Al	37.4243	-1.79014	216
	SIVIM	T-P06467:Aegi	Observation	Aegilops triuncialis L.	L.	ES	Spain	Benisoda			38.82	-0.58	350
	SIVIM	P-P11202:Aegi	Observation	Aegilops triuncialis L.	L.	ES	Spain	Font del Fangar, rodalies, Xerta			40.89	0.38	130
	SIVIM	U-P08150:Aegi	Observation	Aegilops triuncialis L.	L.	ES	Spain	La Nava, Berzocana			39.36	-5.55	0
00:00.0	FCO	6560-1	Specimen	Aegilops triuncialis L.	L.	ES	Spain	CastejÃƒÂ³n de la PeÃƒÂ±a					
	FUND. BIODIVER	1551192	Unknown	Aegilops triuncialis		ESP	Spain	Cantabr. (pr. Durango)		Bi			
00:00.0	REDIAM-CMA	195077	Observation	Aegilops triuncialis		ESP	Spain		Constantina	Se	37.8842	-5.48997	507
00:00.0	REDIAM-CMA	203204	Observation	Aegilops triuncialis		ESP	Spain		AlanÃ-s	Se	38.0683	-5.6413	582
00:00.0	REDIAM-CMA	239986	Observation	Aegilops triuncialis		ESP	Spain		El Real de la Jara	Se	37.9407	-6.04858	685
00:00.0	W	42843	Unknown	Aegilops triuncialis L.		ESP	Spain	Prov. Granada: S von Dilar: von der Eremita Nieves gegen die C					900
00:00.0	REDIAM-CMA	232279	Observation	Aegilops triuncialis		ESP	Spain		AlmadÃ©n de la F	Se	37.8837	-5.99816	417
	SANT	27301	Specimen	Aegilops triuncialis L.		ES	Spain	RubiÃ¡, entre Vilardesilva y PardollÃ		Or			
	SIVIM	T-P19982:Aegi	Observation	Aegilops triuncialis L.	L.	ES	Spain	Almaraz			39.8	-5.68	320
00:00.0	SALA	16060-1	Specimen	Aegilops triuncialis L.	L.	ES	Spain	_; Masueco		Sa			
00:00.0	SALA	14393-1	Specimen	Aegilops triuncialis L.	L.	ES	Spain	_; Pastores		Sa			
	FUND. BIODIVER	1369181	Unknown	Aegilops triuncialis L.	L.	ESP	Spain	Casarejos		So	41.1	-2.1	
	FUND. BIODIVER	1463959	Unknown	Aegilops triuncialis L.	L.	ESP	Spain	Gros, tossal		L	41.1	0.1	420
	FUND. BIODIVER	92444	Unknown	Aegilops triuncialis L.	L.	ESP	Spain	Sacramenia, Convento de San Bern		Sg	41.1	-3.1	
	FUND. BIODIVER	970926	Unknown	Aegilops triuncialis L.	L.	ESP	Spain	Las Cuerlas		Z	40.1	-1.1	
00:00.0	SALA	87728-1	Specimen	Aegilops triuncialis L.	L.	ES	Spain	_; Puertollano, sierra de Puertollano		CR	38.63	-4.14	
00:00.0	REDIAM-CMA	188285	Observation	Aegilops triuncialis		ESP	Spain		AlanÃ-s	Se	38.0456	-5.65185	623
00:00.0	COFC	50178-1	Specimen	Aegilops triuncialis L.	L.	ES	Spain	Valle del Guadiato; arroyo Trigachos		Co			1
00:00.0	MA	718179-1	Specimen	Aegilops triuncialis L.	L.	ES	Spain	Petilla de AragÃ³n.		Na	42	-1	
	SIVIM	T-P16906:Aegi	Observation	Aegilops triuncialis L.	L.	ES	Spain	Loeches			40.37	-3.47	0
00:00.0	COFC	41263-1	Specimen	Aegilops triuncialis L.	L.	ES	Spain	Puente Genil; Km-7,5 entre el munic		Co	37	-4	1
00:00.0	COFC	41301-1	Specimen	Aegilops triuncialis L.	L.	ES	Spain	Cabra; 'La Chacona'		Co	37	-5	1
	FUND. BIODIVER	1369195	Unknown	Aegilops triuncialis L.	L.	ESP	Spain	Soria		So	41.1	-2.1	
	FUND. BIODIVER	930415	Unknown	Aegilops triuncialis L.	L.	ESP	Spain	L'Aleixar, Vilaplana del Camp		T	41.1	0.1	
	FUND. BIODIVER	96898	Unknown	Aegilops triuncialis L.	L.	ESP	Spain	Villadepera		Za			
00:00.0	REDIAM-CMA	148155	Observation	Aegilops triuncialis		ESP	Spain		AlanÃ-s	Se	37.9979	-5.55611	694
00:00.0	REDIAM-CMA	170660	Observation	Aegilops triuncialis		ESP	Spain		Espiel	Co	38.1695	-5.02437	596
	SIVIM	T-P16884:Aegi	Observation	Aegilops triuncialis L.	L.	ES	Spain	Majadahonda			40.46	-3.94	0
	SIVIM	T-P16885:Aegi	Observation	Aegilops triuncialis L.	L.	ES	Spain	Villaviciosa de OdÃ³n			40.28	-3.94	0
	SIVIM	T-P16913:Aegi	Observation	Aegilops triuncialis L.	L.	ES	Spain	Boniches			39.92	-1.71	0
00:00.0	MA	627753-1	Specimen	Aegilops triuncialis L.	L.	ES	Spain	Piedrabuena		CR	39	-4	
00:00.0	MGC	20264-1	Unknown	Aegilops triuncialis L.	L.	ES	Spain	Marbella; Puerto de Cabo Pino. Km		Ma	36.54	-4.73	10

00:00.0	COFC	41261-1	Specimen	Aegilops triuncialis L.	L.	ES	Spain	Cabra; arroyo Pozas		Co	37	-4	1
00:00.0	COFC	41299-1	Specimen	Aegilops triuncialis L.	L.	ES	Spain	Baena; Torre Morena		Co	37	-4	1
	RUS001	VIR100602164	Specimen	Aegilops triuncialis L.		ESP	Spain						
	GDA	GDA43469-1-2	Specimen	Aegilops triuncialis L.	L.	ES	Spain	Granada, Guadix, Rambla Becerra.		GR			990
00:00.0	MA	772200-1	Specimen	Aegilops triuncialis L.	L.	ES	Spain	pr. El RocÃ-o		H	37.1532	-6.4908	
	FUND. BIODIVER	1036795	Unknown	Aegilops triuncialis L.	L.	ESP	Spain	Sierra Almijara		Ma	36.1	-3.1	
	FUND. BIODIVER	1067622	Unknown	Aegilops triuncialis		ESP	Spain	Puebla del RÃ-o		Se	37.1	-5.1	
	FUND. BIODIVER	1073154	Unknown	Aegilops triuncialis		ESP	Spain	Baena		Co	37.1	-4.1	
00:00.0	FUND. BIODIVER	1835135	Unknown	Aegilops triuncialis L.	L.	ESP	Spain	Piedrabuena, volcan de Piedrabuen		CR	38.1	-3.1	600
	SEV	10311-1	Specimen	Aegilops triuncialis L.	L.	ES	Spain	H. R. Matritensis		M			1
00:00.0	REDIAM-CMA	394390	Observation	Aegilops triuncialis		ESP	Spain		Hornos	J	38.18	-2.64657	1430
00:00.0	COA	41153-1	Specimen	Aegilops triuncialis L.	L.	ES	Spain	Km 30 de CÃ³rdoba al Carpio		Co	37.67	-4.59	
00:00.0	FCO	10586-1	Specimen	Aegilops triuncialis L.	L.	ES	Spain	Santillana, embalse		M			
00:00.0	BC	646874	Specimen	Aegilops triuncialis L.	L.	ES	Spain	Santa Susanna; Pineda, platja de S		B	41.6	2.7	
00:00.0	BC	70820	Specimen	Aegilops triuncialis L.	L.	ES	Spain	Torres; Prado del Hoyalino (AlmadÃ		J	37.72	-3.51	1500
	BC	70823	Specimen	Aegilops triuncialis L.	L.	ES	Spain	Esteba		Se			
00:00.0	SEV	30541-1	Specimen	Aegilops triuncialis L.	L.	ES	Spain	Entre MorÃ³n y Pruna, arroyo Salad		Se			1
00:00.0	MGC	72820-1	Unknown	Aegilops triuncialis L.	L.	ES	Spain	Casares; Camino de Los Pobres		Ma	36.442	-5.315	190
	BDBCV-General	74120	Observation	Aegilops triuncialis		ESPA	Spain	Villanueva de Viv	El Alto Mijares	Castel	40.0397	-0.59724	
00:00.0	COFC	12024-1	Specimen	Aegilops triuncialis L.	L.	ES	Spain	Lucena; cortijo 'El Duque'		Co	37	-4	1
00:00.0	COFC	21305-1	Specimen	Aegilops triuncialis L.	L.	ES	Spain	Sierra de Hornachuelos; 'Minas de l		Co			1
	FUND. BIODIVER	1141592	Unknown	Aegilops triuncialis L.	L.	ESP	Spain	Puente Villarente		Le	42.1	-5.1	754
00:00.0	FUND. BIODIVER	1946465	Unknown	Aegilops triuncialis L.	L.	ESP	Spain	Aldea del Rey, cerro de la Vaqueriz		CR	38.1	-3.1	750
00:00.0	GDAC	GDAC39914-1	Specimen	Aegilops triuncialis L.	L.	ES	Spain	Granada, SÂª de Castril, rÃ-o Castr		GR			910
00:00.0	MA	562406-1	Specimen	Aegilops triuncialis L.	L.	ES	Spain	Vall d'AlcalÃ¡, cerca de Adsubia		A	38	0	
	SIVIM	T-P05761:Aegi	Observation	Aegilops triuncialis L.	L.	ES	Spain	HondÃ³n de las Nieves			38.19	-0.94	0
	SIVIM	T-P06426:Aegi	Observation	Aegilops triuncialis L.	L.	ES	Spain	Los Herreros			39.28	-1.02	0
00:00.0	BC	597977	Specimen	Aegilops triuncialis L.	L.	ES	Spain	la Granja d'Escarp; SegriÃ : Vers la		L	41.39	0.31	
	SANT	2109	Specimen	Aegilops triuncialis L.		ES	Spain	'Chozas de la Sierra'		M			
00:00.0	GDA	GDA12184-1	Specimen	Aegilops triuncialis L.	L.	ES	Spain	Granada, Lobras, falda del cerro Ve		GR			800
00:00.0	BC	70821	Specimen	Aegilops triuncialis L.	L.	ES	Spain	Jodar; Golondrina		J	37.81	-3.28	800
00:00.0	SEV	122196-1	Specimen	Aegilops triuncialis L.	L.	ES	Spain	San MartÃ-n de la Vega, cerro Buta		M			1
	IPK	32134	Living	Aegilops triuncialis L.		ESP	Spain	Guara					
00:00.0	REDIAM-CMA	243391	Observation	Aegilops triuncialis		ESP	Spain		Cortegana	H	37.9188	-6.83211	612
00:00.0	REDIAM-CMA	251288	Observation	Aegilops triuncialis		ESP	Spain		AlÃ¡jar	H	37.8746	-6.64956	701
00:00.0	REDIAM-CMA	266848	Observation	Aegilops triuncialis		ESP	Spain		Espiel	Co	38.0944	-4.99147	500
	SIVIM	T-P28629:Aegi	Observation	Aegilops triuncialis L.	L.	ES	Spain	Riberos del Tajo, Serradilla			39.71	-6.2	260
	SIVIM	T-P28912:Aegi	Observation	Aegilops triuncialis L.	L.	ES	Spain	Riberos del Tajo, Serradilla			39.8	-6.19	240
	SIVIM	T-P30050:Aegi	Observation	Aegilops triuncialis L.	L.	ES	Spain	Pr. Corral de la Rada, Villamalea, Albacete			39.29	-1.6	0
	SIVIM	T-P31987:Aegi	Observation	Aegilops triuncialis L.	L.	ES	Spain	de AlcalÃ¡ de Henares, finca La Clota , Pro			40.46	-3.47	0
	SIVIM	U-P06716:Aegi	Observation	Aegilops triuncialis L.	L.	ES	Spain	Cerro de S. Miguel			37.04	-7.87	100

00:00.0	MA	554950-1	Specimen	Aegilops triuncialis L.	L.	ES	Spain	Estepona, Sierra Bermeja de Estep		Ma	36	-4	
00:00.0	MA	651058-1	Specimen	Aegilops triuncialis L.	L.	ES	Spain	AndÃºjar, Arroyo Membrillejo		J	38	-4	
00:00.0	BC	124222	Specimen	Aegilops triuncialis L.	L.	ES	Spain	Barcelona; Barcelona, Pedralbes		B	41.41	2.1	
00:00.0	COA	41195-1	Specimen	Aegilops triuncialis L.	L.	ES	Spain	El Porvenir de la Industria, PeÃ±arr		Co	38.28	-5.4	
00:00.0	COFC	46879-1	Specimen	Aegilops triuncialis L.	L.	ES	Spain	ctra de Santa MarÃƒÂ-a de Trasierr		Co			1
00:00.0	COFC	46901-1	Specimen	Aegilops triuncialis L.	L.	ES	Spain	Posadas; ctra de Hornachuelos CO		Co			1
00:00.0	COFC	46911-1	Specimen	Aegilops triuncialis L.	L.	ES	Spain	Posadas; carretera hacia Villavicios		Co	37	-5	1
	FUND. BIODIVER	1762693	Unknown	Aegilops triuncialis		ESP	Spain	Volcan de Yezosa		CR	38.1	-3.1	
00:00.0	SEV	98921-1	Specimen	Aegilops triuncialis L.	L.	ES	Spain	Constantina, Finca El Retortillo		Se			1
00:00.0	SEV	98922-1	Specimen	Aegilops triuncialis L.	L.	ES	Spain	BenaojÃ¡n. Sierra de San Diego		Ma			1000
	SEV	98946-1	Specimen	Aegilops triuncialis L.	L.	ES	Spain	Puebla del RÃ-o		Se			1
00:00.0	REDIAM-CMA	382893	Observation	Aegilops triuncialis		ESP	Spain		Torres	J	37.7783	-3.50637	923
	SIVIM	P-P08892:Aegi	Observation	Aegilops triuncialis L.	L.	ES	Spain	Barranc de la Canaleta; Horta de Sant Joa			40.88	0.26	0
	SIVIM	U-P09955:Aegi	Observation	Aegilops triuncialis L.	L.	ES	Spain	Cuneta cerca de Casas Blancas			38.84	-3	0
00:00.0	MA	562505-1	Specimen	Aegilops triuncialis L.	L.	ES	Spain	Castell de Castells, rincÃ³n de Fora		A	38	0	
	IPK	AE 1169	Living	Aegilops triuncialis L.			Spain	Tras-os-Montes (Alto Douro), Portelo - Brag			41.9292	-6.27083	
00:00.0	MGC	13224-1	Unknown	Aegilops triuncialis L.	L.	ES	Spain	RÃ-o Aguas		Al	37.09	-2.04	1
00:00.0	BC	91859	Specimen	Aegilops triuncialis L.	L.	ES	Spain	Zamora; Zamora. Alto de las burras		Za	41.48	-5.69	
00:00.0	FUND. BIODIVER	1684422	Unknown	Aegilops triuncialis L.	L.	ESP	Spain	Sierra del Pinar, ladera N de El Torr		Ca	36.1	-5.1	
00:00.0	GDA	GDA30029-1-1	Specimen	Aegilops triuncialis L.	L.	ES	Spain	JaÃ©n, SÂª MÃ¡gina, SÂª de la Cru		J			1000
00:00.0	BC	70840	Specimen	Triticum triunciale (L.) Raspail	(L.) Raspail	ES	Spain	Chinchilla; Chinchilla		Ab	38.89	-1.67	
00:00.0	HUAL	5783-1	Specimen	Aegilops triuncialis L.	L.	ES	Spain	Velefique; SÂª de Los Filabres, La C		Al	37.203	-2.397	
00:00.0	REDIAM-CMA	264725	Observation	Aegilops triuncialis		ESP	Spain		Cazorla	J	37.9096	-2.92014	1175
00:00.0	REDIAM-CMA	274949	Observation	Aegilops triuncialis		ESP	Spain		Villaviciosa de CÃ	Co	37.9674	-4.99164	300
00:00.0	REDIAM-CMA	282355	Observation	Aegilops triuncialis		ESP	Spain		Hinojosa del Duq	Co	38.4139	-5.13987	611
	SIVIM	T-P30045:Aegi	Observation	Aegilops triuncialis L.	L.	ES	Spain	El Campichuelo, gravera, Jalance, Valencia			39.19	-1.26	0
	SIVIM	T-P31985:Aegi	Observation	Aegilops triuncialis L.	L.	ES	Spain	de AlcalÃ¡ de Henares, finca La Clota , Pro			40.46	-3.47	0
	SIVIM	U-P06653:Aegi	Observation	Aegilops triuncialis L.	L.	ES	Spain	Rocha da Pena			37.22	-8.21	0
00:00.0	COFC	46908-1	Specimen	Aegilops triuncialis L.	L.	ES	Spain	Hornachuelos; tÃƒÂ©rmino municip		Co			1
	FUND. BIODIVER	1001681	Unknown	Aegilops triuncialis		ESP	Spain	Puerto de Cabrejas		Cu	39.1	-2.1	
	FUND. BIODIVER	1012903	Unknown	Aegilops triuncialis L.	L.	ESP	Spain	RÃ-o Aguas		Al	36.1	-2.1	
00:00.0	SEV	99218-1	Specimen	Aegilops triuncialis L.	L.	ES	Spain	Sierra Bermeja		Ma			800
	BDBCV-General	279918	Observation	Aegilops triuncialis		ESPA	Spain	Teresa de Cofren	El Valle de Ayora	Valenc	39.146	-0.97503	
00:00.0	REDIAM-CMA	293908	Observation	Aegilops triuncialis		ESP	Spain		La Puerta de Seg	J	38.3845	-2.80499	687
00:00.0	REDIAM-CMA	382828	Observation	Aegilops triuncialis		ESP	Spain				37.6367	-3.13218	
	DEU146	AE 902	Specimen	Aegilops triuncialis subsp. triuncialis var. flaves		ESP	Spain	Penarroya-Pueblonuevo, Cordoba					
	SIVIM	P-P12754:Aegi	Observation	Aegilops triuncialis L.	L.	ES	Spain	Carretera de Santa MarÃ-a de Nieva			37.49	-2.09	1090
	SIVIM	U-P08202:Aegi	Observation	Aegilops triuncialis L.	L.	ES	Spain	Alcorneo, Valencia de AlcÃ¡ntara			39.28	-7.26	0
00:00.0	GDAC	GDAC24839-1	Specimen	Aegilops triuncialis L.	L.	ES	Spain	Zamora, CaÃ±izal.		ZA			0
00:00.0	MA	755445-1	Specimen	Aegilops triuncialis L.	L.	ES	Spain	Lastras de Cuellar, La Poza		Sg	41	-4	
00:00.0	UNEX	28636-1	Observation	Aegilops triuncialis L.	_	ESP	Spain	Cabeza del Buey: Pastizal llano de		Ba	38.6	-5.3	

	W	43562	Unknown	Aegilops triuncialis L.		ESP	Spain	Granada.					
	W	43585	Unknown	Aegilops triuncialis L.		ESP	Spain	Granada. ubique in collibus.					
00:00.0	BC	92748	Specimen	Aegilops triuncialis L.	L.	ES	Spain	Sant Guim de Freixenet; Sant Guim		L	41.68	1.38	
00:00.0	COA	41180-1	Specimen	Aegilops triuncialis L.	L.	ES	Spain	Cruce carretera MÃ¡laga		Co	37.58	-4.7	
00:00.0	COA	41202-1	Specimen	Aegilops triuncialis L.	L.	ES	Spain	Posadas		Co	37.75	-5.16	
00:00.0	SALA	71779-1	Specimen	Aegilops triuncialis L.	L.	ES	Spain	_; SerrejÃ³n		Cc	39.82	-5.8	
00:00.0	BC	92752	Specimen	Aegilops triuncialis L.	L.	ES	Spain	Vallfogona de Riucorb; Vallfogona d		T	41.58	1.26	
00:00.0	COFC	41145-1	Specimen	Aegilops triuncialis L.	L.	ES	Spain	Luque; cerro Juan MartÃƒÂ-n		Co	37	-4	1
	ESP004	NC043490	Specimen	Aegilops triuncialis L.		ESP	Spain	Casas de Belvis, Belvis de Monroy, provinc			39.8167	-5.58333	296
00:00.0	SEV	98914-1	Specimen	Aegilops triuncialis L.	L.	ES	Spain	Carretera de Posadas a Villaviciosa		Co			1
00:00.0	SEV	98939-1	Specimen	Aegilops triuncialis L.	L.	ES	Spain	Jerez de la Frontera		Ca			1
00:00.0	SALA	82104-1	Specimen	Aegilops triuncialis L.	L.	ES	Spain	_; Moraleja		Cc	40.1	-6.81	
	FUND. BIODIVER	78797	Unknown	Aegilops triuncialis L.	L.	ESP	Spain	Quintanilla de Arriba		Va	41.1	-4.1	
00:00.0	REDIAM-CMA	91262	Observation	Aegilops triuncialis		ESP	Spain		Hornachuelos	Co	37.8487	-5.33218	200
00:00.0	REDIAM-CMA	96601	Observation	Aegilops triuncialis		ESP	Spain		El Pedroso	Se	37.7995	-5.78411	350
00:00.0	REDIAM-CMA	107155	Observation	Aegilops triuncialis		ESP	Spain		El Castillo de las	Se	37.7133	-6.32993	300
00:00.0	REDIAM-CMA	117690	Observation	Aegilops triuncialis		ESP	Spain		Cazalla de la Sier	Se	37.9731	-5.76512	608
00:00.0	REDIAM-CMA	128096	Observation	Aegilops triuncialis		ESP	Spain		Cazalla de la Sier	Se	37.9295	-5.71219	439
00:00.0	COFC	25776-1	Specimen	Aegilops triuncialis L.	L.	ES	Spain	Santa Olalla del Cala; Sierra de Ara		H	37	-6	1
1880-06-1	W	55335	Unknown	Aegilops triuncialis L.		ESP	Spain	en los Clanos de paller Ã¡ 3 kilometres Sur de La Carolina terreno arenoso, (In d					
	SIVIM	T-P10275:Aegi	Observation	Aegilops triuncialis L.	L.	ES	Spain	Puerto Viejo (Los Villares)			37.58	-3.9	1300
	SIVIM	T-P11406:Aegi	Observation	Aegilops triuncialis L.	L.	ES	Spain	El Ventorro a Villalba			40.19	-2.17	0
	SIVIM	T-P15744:Aegi	Observation	Aegilops triuncialis L.	L.	ES	Spain	Arroyo Paredones, Sierra de Aguas			36.85	-4.79	300
00:00.0	GDA	GDA30009-1-2	Specimen	Aegilops triuncialis L.	L.	ES	Spain	MÃ¡laga, AxarquÃ-a.		MA			0
00:00.0	MGC	11346-1	Unknown	Aegilops triuncialis L.	L.	ES	Spain	Algodonales; Sierra de LÃ-jar		Ca	0	0	500
00:00.0	COFC	4630-1	Specimen	Aegilops triuncialis L.	L.	ES	Spain	CardeÃƒÂ±a; finca 'El TelÃƒÂ©graf		Co			1
00:00.0	SALA	18838-1	Specimen	Aegilops triuncialis L.	L.	ES	Spain	_; Villarino de los Aires		Sa			
00:00.0	SALA	16077-1	Specimen	Aegilops triuncialis L.	L.	ES	Spain	_; La Fregeneda		Sa			
00:00.0	FUND. BIODIVER	1946456	Unknown	Aegilops triuncialis L.	L.	ESP	Spain	San Lorenzo de Calatrava, cerro de		CR	38.1	-3.1	820
	FUND. BIODIVER	52932	Unknown	Aegilops triuncialis L.	L.	ESP	Spain	Arapiles, borde de la carretera, a la		Sa	40.1	-5.1	
	FUND. BIODIVER	56195	Unknown	Aegilops triuncialis L.	L.	ESP	Spain	Olmedo		Va	41.1	-4.1	
00:00.0	GDAC	GDAC26139-1	Specimen	Aegilops triuncialis L.	L.	ES	Spain	Granada, SÂª de Baza, de la estaci		GR			0
00:00.0	REDIAM-CMA	72415	Observation	Aegilops triuncialis		ESP	Spain		Villanueva de la R	J	38.2976	-3.84733	699
00:00.0	SALA	12481-1	Specimen	Aegilops triuncialis L.	L.	ES	Spain	_; San Esteban de la Sierra		Sa			
	SIVIM	S-P14057:Aegi	Observation	Aegilops triuncialis L.	L.	ES	Spain	Serrinha			38.39	-8.54	350
00:00.0	GDA	GDA23166-1-2	Specimen	Aegilops triuncialis L.	L.	ES	Spain	Madrid, S. MartÃ-n de la Vega, cerr		M			0
00:00.0	GDA	GDA30026-1	Specimen	Aegilops triuncialis L.	L.	ES	Spain	Granada, Alhambra, Cementerio.		GR			0
	GDAC	GDAC29637-1	Specimen	Aegilops triuncialis L.	L.	ES	Spain	Granada, SÂª de Parapanda, carret		GR			1000
	FUND. BIODIVER	1714999	Unknown	Aegylops triuncialis L.	L.	ESP	Spain	Sierra las Corchuelas, Parque Natu		Cc	39.1	-5.1	
00:00.0	COA	41179-1	Specimen	Aegilops triuncialis L.	L.	ES	Spain	Club de Golf		Co	37.93	-4.82	
00:00.0	REDIAM-CMA	190414	Observation	Aegilops triuncialis		ESP	Spain		San NicolÃ¡s del	Se	37.9566	-5.62413	650

00:00.0	REDIAM-CMA	200943	Observation	Aegilops triuncialis		ESP	Spain		Constantina	Se	37.8637	-5.6697	538
00:00.0	REDIAM-CMA	210905	Observation	Aegilops triuncialis		ESP	Spain		Hornachuelos	Co	37.9112	-5.29736	393
00:00.0	REDIAM-CMA	233372	Observation	Aegilops triuncialis		ESP	Spain		Fuente Obejuna	Co	38.1377	-5.3485	599
00:00.0	COFC	52519-1	Specimen	Aegilops triuncialis L.	L.	ES	Spain	Alconera; _		Ba	38	-6	1
00:00.0	COFC	41305-1	Specimen	Aegilops triuncialis L.	L.	ES	Spain	Lucena; venta 'La Camila'		Co	37	-4	1
00:00.0	MGC	43914-1	Unknown	Aegilops triuncialis L.	L.	ES	Spain	BenahavÃs; Sierra Palmitera. Carre		Ma	36.6	-5.063	1100
	SEV	98912-1	Specimen	Aegilops triuncialis L.	L.	ES	Spain	Constantina. Sierra Norte		Se			1
00:00.0	SEV	98937-1	Specimen	Aegilops triuncialis L.	L.	ES	Spain	BÃ©lmez. Arroyo de la Juliana		Co			1
00:00.0	COFC	36340-1	Specimen	Aegilops triuncialis L.	L.	ES	Spain	IznÃƒÂ¡jar; cerro de la Camorrilla y		Co			1
00:00.0	UNEX	05901-1	Observation	Aegilops triuncialis L.	_	ESP	Spain	Badajoz: Cantuesal, camino cemen		Ba	38.8	-7.00001	
00:00.0	ABH	53011-1	Specimen	Aegilops triuncialis L.	L.	ES	Spain	Villena; PeÃ±a Rubia		A	38.59	-0.81	
00:00.0	MGC	13918-1	Unknown	Aegilops triuncialis L.	L.	ES	Spain	Castrillo de la GuareÃ±a; _		Za	0	0	1
	FUND. BIODIVER	1762694	Unknown	Aegilops triuncialis		ESP	Spain	Volcan de Piedrabuena		CR	38.1	-4.1	
	IDBD-GN	42515	Observation	Aegilops triuncialis L.	L.	ES	Spain	Bambial	Foz de ArbayÃºn-	Na	42.6619	-1.23204	
00:00.0	REDIAM-CMA	370098	Observation	Aegilops triuncialis		ESP	Spain		Villanueva de CÃ	Co	38.3127	-4.55277	727
	IPK	AE 1170	Living	Aegilops triuncialis L.			Spain	Puerto Real (Cadiz)			36.5331	-6.81667	
00:00.0	SALA	47671-1	Specimen	Aegilops triuncialis L.	L.	ES	Spain	_; Fuentes de BÃ©jar		Sa			
	SIVIM	U-P08620:Aegi	Observation	Aegilops triuncialis L.	L.	ES	Spain	Sierra del Carche			38.38	-1.16	1060
00:00.0	BC	70825	Specimen	Aegilops triuncialis L.	L.	ES	Spain	Alcaraz; La Molata pr. Alcaraz (Reg		Ab	38.62	-2.48	
00:00.0	SALA	40758-1	Specimen	Aegilops triuncialis L.	L.	ES	Spain	_; Santo TomÃ© del Puerto, Siguer		Sg			
00:00.0	SEV	101357-1	Specimen	Aegilops triuncialis L.	L.	ES	Spain	San NicolÃ¡s del Puerto. Cerro del I		Se			670
00:00.0	SEV	101358-1	Specimen	Aegilops triuncialis L.	L.	ES	Spain	Priego de CÃ³rdoba. Pala de Malom		Co			1
00:00.0	BC	70831	Specimen	Aegilops triuncialis L.	L.	ES	Spain	la Cellera de Ter; Sorrals del Ter, s		Ge	41.96	2.58	
00:00.0	BC	70836	Specimen	Triticum triunciale (L.) Raspail	(L.) Raspail	ES	Spain	Ateca; in Aragonia australe: Ateca		Z	41.32	-1.75	650
00:00.0	ABH	52936-1	Specimen	Aegilops triuncialis L.	L.	ES	Spain	Petrel; Arenal de Petrel		A	38.5	-0.79	
00:00.0	SALA	84427-1	Specimen	Aegilops triuncialis L.	L.	ES	Spain	_; CastronuÃ±o		Va	41.39	-5.27	
00:00.0	MA	722804-1	Specimen	Aegilops triuncialis L.	L.	ES	Spain	San Lorenzo de Calatrava, cerro de		CR	38	-3	
	CZE122	01C2107102	Specimen	Aegilops triuncialis subsp. triuncialis var. flaves		ESP	Spain	Guara, Spanien					
	FUND. BIODIVER	1674405	Unknown	Aegilops triuncialis		ESP	Spain	Lucillos, Los Nogales		To	39.1	-4.1	350
	BDBCV-General	274437	Observation	Aegilops triuncialis		ESPA	Spain	Bolbaite	La Canal de Nava	Valenc	39.0517	-0.74664	
00:00.0	HUAL	2952-1	Specimen	Aegilops triuncilis L.	L.	ES	Spain	SÂª de GÃ¡dor, Balsa de CaparidÃ¡		Al	36.934	-2.893	
00:00.0	REDIAM-CMA	266938	Observation	Aegilops triuncialis		ESP	Spain		Fuente Obejuna	Co	38.3252	-5.31475	528
00:00.0	REDIAM-CMA	281796	Observation	Aegilops triuncialis		ESP	Spain		Hinojosa del Duq	Co	38.4915	-5.04803	542
00:00.0	REDIAM-CMA	284957	Observation	Aegilops triuncialis		ESP	Spain		Torres	J	37.7456	-3.53898	1393
00:00.0	GDAC	GDAC4835-1	Specimen	Aegilops triuncialis L.	L.	ES	Spain	MÃ¡laga, Torcal de Antequera.		MA			1200
	SIVIM	T-P27623:Aegi	Observation	Aegilops triuncialis L.	L.	ES	Spain	Prados de la Nava, Sierra de Cabra			37.48	-4.47	1010
	SIVIM	T-P28633:Aegi	Observation	Aegilops triuncialis L.	L.	ES	Spain	Castillo de Mirabel			39.8	-6.31	0
	SIVIM	T-P28917:Aegi	Observation	Aegilops triuncialis L.	L.	ES	Spain	Puente de El Cardenal, TorrejÃ³n el Rubio			39.8	-5.92	280
	SIVIM	T-P30055:Aegi	Observation	Aegilops triuncialis L.	L.	ES	Spain	El Campichuelo, Cofrentes, Valencia			39.19	-1.14	0
	SIVIM	U-P02907:Aegi	Observation	Aegilops triuncialis L.	L.	ES	Spain	Vall d'AlcalÃ , Beniaia			38.72	-0.35	0
	SIVIM	U-P07712:Aegi	Observation	Aegilops triuncialis L.	L.	ES	Spain	Valencia: BÃ©tera			39.54	-0.55	100

00:00.0	SEV	98908-1	Specimen	Aegilops triuncialis L.	L.	ES	Spain	Villaverde del RÃ-o		Se			1
00:00.0	SEV	99070-1	Specimen	Aegilops triuncialis L.	L.	ES	Spain	Entre ValdepeÃ±as de JaÃ©n y Los		J			1200
00:00.0	REDIAM-CMA	282311	Observation	Aegilops triuncialis		ESP	Spain		Hinojosa del Duq	Co	38.4053	-5.16508	600
	SIVIM	T-P28614:Aegi	Observation	Aegilops triuncialis L.	L.	ES	Spain	Finca de los Cuartos, ValdecaÃ±as de Taj			39.71	-5.68	320
	SIVIM	T-P28877:Aegi	Observation	Aegilops triuncialis L.	L.	ES	Spain	Casatejada			39.8	-5.68	280
	SIVIM	T-P29485:Aegi	Observation	Aegilops triuncialis L.	L.	ES	Spain	Diferentes localidades de la Sierra del Agu			36.85	-4.79	0
00:00.0	GDA	GDA15837-1	Specimen	Aegilops triuncialis L.	L.	ES	Spain	Granada, SÂª Sagra, HuÃ©scar, co		GR			0
00:00.0	MGC	1107-1	Unknown	Aegilops triuncialis L.	L.	ES	Spain	MÃ¡laga; Puerto de la Torre		Ma	0	0	1
	RUS001	VIR100602144	Specimen	Aegilops triuncialis L.		ESP	Spain						
	RUS001	VIR100602310	Specimen	Aegilops triuncialis L.		ESP	Spain						
	FUND. BIODIVER	1085279	Unknown	Aegilops triuncialis		ESP	Spain	Paracuellos del Jarama		M	40.1	-3.1	
	FUND. BIODIVER	1946448	Unknown	Aegilops triuncialis L.	L.	ESP	Spain	Provincia de Ciudad Real		CR			
	FUND. BIODIVER	1674407	Unknown	Aegilops triuncialis		ESP	Spain	San RomÃ¡n		To	39.1	-4.1	480
00:00.0	COFC	41260-1	Specimen	Aegilops triuncialis L.	L.	ES	Spain	Cabra; cerro los Cerrajones		Co	37	-4	1
00:00.0	COFC	41297-1	Specimen	Aegilops triuncialis L.	L.	ES	Spain	Cabra; arroyo Galindo		Co	37	-4	1
00:00.0	COFC	41298-1	Specimen	Aegilops triuncialis L.	L.	ES	Spain	Baena; cerro Valladolid		Co	37	-4	1
00:00.0	REDIAM-CMA	422506	Observation	Aegilops triuncialis		ESP	Spain		Villanueva de CÃ	Co	38.3611	-4.57028	700
00:00.0	REDIAM-CMA	267617	Observation	Aegilops triuncialis		ESP	Spain		Santa Eufemia	Co	38.6086	-4.87497	463
	SIVIM	R-P08597:Aegi	Observation	Aegilops triuncialis L.	L.	ES	Spain	30 S WF 3082			36.86	-2.66	1050
	SIVIM	S-P02910:Aegi	Observation	Aegilops triuncialis L.	L.	ES	Spain	VallÃ¨s: entre Terrassa et Matadepera, vers			41.54	1.92	0
	SIVIM	T-P27666:Aegi	Observation	Aegilops triuncialis L.	L.	ES	Spain	PrÃ³x. Cjo. Rosa Alta, SÂª de Rute			37.3	-4.35	0
	SIVIM	T-P28635:Aegi	Observation	Aegilops triuncialis L.	L.	ES	Spain	BaldÃ-os de TorrejÃ³n el Rubio			39.71	-6.08	330
	SIVIM	T-P28921:Aegi	Observation	Aegilops triuncialis L.	L.	ES	Spain	Riberos del Tajo, Serradilla			39.8	-6.19	260
	SIVIM	T-P30057:Aegi	Observation	Aegilops triuncialis L.	L.	ES	Spain	Los Callejones, Cofrentes, Valencia			39.19	-1.26	0
00:00.0	MA	644448-1	Specimen	Aegilops triuncialis L.	L.	ES	Spain	Cullar Baza, El MÃ¡rgen, arroyo El M		Gr	37	-2	
00:00.0	COFC	29895-1	Specimen	Aegilops triuncialis L.	L.	ES	Spain	Guadalcanal; Charco de la Sal		Se			1
00:00.0	BC	92751	Specimen	Aegilops triuncialis L.	L.	ES	Spain	Sant Just Desvern; S. Pere Martir B		B	41.41	2.1	
00:00.0	BC	805385	Specimen	Aegilops triuncialis L.	L.	ES	Spain	Orihuela del Tremedal; Orihuela		Te	40.4635	-1.70236	
	MA	654049-1	Specimen	Aegilops triuncialis L.	L.	ES	Spain	BÃ©jar		Sa			
00:00.0	SEV	9949-1	Specimen	Aegilops triuncialis L.	L.	ES	Spain	Ronda. Sierra de las Nieves		Ma			1300
	SANT	17437	Specimen	Aegilops triuncialis L.		ES	Spain	RubiÃ¡,Cobas,en la estaciÃ³n del fe		Or			
00:00.0	BC	601433	Specimen	Aegilops triuncialis L.	L.	ES	Spain	Vallclara; Muntanyes de Prades: Ve		T	41.4	1.03	625
00:00.0	COA	41182-1	Specimen	Aegilops triuncialis L.	L.	ES	Spain	De Hinojosa a BelalcÃ¡zar		Co	38.47	-5.18	
00:00.0	COA	41204-1	Specimen	Aegilops triuncialis L.	L.	ES	Spain	Sierra Montoro, entre los rÃ-os Aren		Co	38.03	-4.48	
00:00.0	MGC	20415-1	Unknown	Aegilops triuncialis L.	L.	ES	Spain	Sierra de Tejeda. Entre el cruce y C		Ma	0	0	1
	SANT	20739	Specimen	Aegilops triuncialis L.		ES	Spain	RubiÃ¡,Cobas;en la estaciÃ³n de fer		Or			
00:00.0	SALA	101526-1	Specimen	Aegilops triuncialis L.	L.	ES	Spain	_; HervÃ¡s, Pista Heidi		Cc	40.26	-5.87	
00:00.0	MA	753631-1	Specimen	Aegilops triuncialis L.	L.	ES	Spain	Parque de Invierno		Gr			
00:00.0	SEV	98918-1	Specimen	Aegilops triuncialis L.	L.	ES	Spain	Entre Azuel y el rÃ-o Yeguas		Co			1
00:00.0	SEV	98943-1	Specimen	Aegilops triuncialis L.	L.	ES	Spain	Guadalupe, bajada de la Sierra del		Cc			820
	RUS001	VIR100602128	Specimen	Aegilops triuncialis L.		ESP	Spain						

	RUS001	VIR100602085	Specimen	Aegilops triuncialis L.		ESP	Spain						
00:00.0	SALA	124862-1	Specimen	Aegilops triuncialis L.	L.	ES	Spain	Babilafuente, El Llano		Sa	40.98	-5.46	
	FUND. BIODIVER	1085274	Unknown	Aegilops triuncialis		ESP	Spain	HÃºmera		M	40.1	-3.1	
00:00.0	FUND. BIODIVER	1946443	Unknown	Aegilops triuncialis L.	L.	ESP	Spain	Navalpino, rio Valdehornos		CR	39.1	-4.1	540
00:00.0	REDIAM-CMA	12906	Observation	Aegilops triuncialis		ESP	Spain		Lora del RÃo	Se	37.6757	-5.55406	124
00:00.0	COA	28712-1	Specimen	Aegilops triuncialis L.	L.	ES	Spain	Antequera, Monte Hacho, Partido de		Ma	36.95	-4.57	
00:00.0	COA	41151-1	Specimen	Aegilops triuncialis L.	L.	ES	Spain	4 Km al N de Villaharta		Co	38.11	-4.94	
00:00.0	COA	41237-1	Specimen	Aegilops triuncialis L.	L.	ES	Spain	Villanueva del RÃo		Co	38.2	-5.17	
	CZE122	01C2107117	Specimen	Aegilops triuncialis subsp. triuncialis L.		ESP	Spain	Guara					
00:00.0	SALA	85587-1	Specimen	Aegilops triuncialis L.	L.	ES	Spain	_; DoÃ±inos		Sa			
00:00.0	COA	41282-1	Specimen	Aegilops triuncialis L.	L.	ES	Spain	Km 5 al Sur de CardeÃ±a		Co	38.21	-4.37	
00:00.0	SALA	80653-1	Specimen	Aegilops triuncialis L.	L.	ES	Spain	_; San MartÃn de la Vega		M			
00:00.0	SEV	30535-1	Specimen	Aegilops triuncialis L.	L.	ES	Spain	Entre MorÃ³n y Puebla de Cazalla		Se			1
00:00.0	SEV	59331-1	Specimen	Aegilops triuncialis L.	L.	ES	Spain	Entre Coripe y Montellano		Se			1
00:00.0	ABH	47705-1	Specimen	Aegilops triuncialis L.	L.	ES	Spain	Jarafuel; Tejares Abarca		V	39.14	-1.06	
	BDBCV-General	74116	Observation	Aegilops triuncialis		ESPA	Spain	Argelita	El Alto Mijares	Castel	40.0346	-0.36305	
00:00.0	REDIAM-CMA	306582	Observation	Aegilops triuncialis		ESP	Spain		Guillena	Se	37.6692	-6.17041	171
	SIVIM	P-P08868:Aegi	Observation	Aegilops triuncialis L.	L.	ES	Spain	Mas de la Caramassa; H. St. Joan			40.88	0.26	0
	SIVIM	U-P09953:Aegi	Observation	Aegilops triuncialis L.	L.	ES	Spain	Carretera de Ossa a Ruidera			38.93	-2.76	0
00:00.0	COA	41183-1	Specimen	Aegilops triuncialis L.	L.	ES	Spain	Km 6 de Alcaracejos a El Viso		Co	38.38	-5.06	
00:00.0	COA	41216-1	Specimen	Aegilops triuncialis L.	L.	ES	Spain	JardÃn BotÃ¡nico de CÃ³rdoba, cul		Co	37.84	-4.82	
00:00.0	MGC	54823-1	Unknown	Aegilops triuncialis L.	L.	ES	Spain	Estepona; Sierra Bermeja. Tinao de		Ma	0	0	190
	ESP004	NC050474	Specimen	Aegilops triuncialis L.		ESP	Spain	Cuenca/Carrascosa del Campo 5km W, Fu			40.0833	-2.21667	960
00:00.0	COFC	41514-1	Specimen	Aegilops triuncialis L.	L.	ES	Spain	Priego de CÃƒÂ³rdoba; la TiÃƒÂ±os		Co			750
00:00.0	SALA	7590-1	Specimen	Aegilops triuncialis L.	L.	ES	Spain	_; Cogeces de Iscar		Va			
	FUND. BIODIVER	117647	Unknown	Aegilops triuncialis L.	L.	ESP	Spain	Burgos		Bu	42.1	-3.1	
00:00.0	FUND. BIODIVER	1946454	Unknown	Aegilops triuncialis L.	L.	ESP	Spain	San Lorenzo de Calatrava, finca Pe		CR	38.1	-3.1	800
	FUND. BIODIVER	56193	Unknown	Aegilops triuncialis L.	L.	ESP	Spain	Bocigas		Va	41.1	-4.1	
	FUND. BIODIVER	61255	Unknown	Aegilops triuncialis L.	L.	ESP	Spain	BÃ©jar		Sa	40.1	-5.1	
00:00.0	REDIAM-CMA	47505	Observation	Aegilops triuncialis		ESP	Spain		Torres	J	37.7455	-3.53918	1393
00:00.0	REDIAM-CMA	63142	Observation	Aegilops triuncialis		ESP	Spain		Carcabuey	Co	37.4067	-4.30885	753
	W	43793	Unknown	Aegilops triuncialis L.		ESP	Spain	Plantas EspaÃ±olas. Granada; xxx					
	SIVIM	T-P06094:Aegi	Observation	Aegilops triuncialis L.	L.	ES	Spain	de Venta el CastaÃ±o, en las Yeseras			37.04	-2.21	280
	SIVIM	T-P06967:Aegi	Observation	Aegilops triuncialis L.	L.	ES	Spain	Puente Castro			42.51	-5.55	0
00:00.0	SEV	98920-1	Specimen	Aegilops triuncialis L.	L.	ES	Spain	Arroyo anterior al rÃo NÃ©valo		Co			1
00:00.0	SEV	98945-1	Specimen	Aegilops triuncialis L.	L.	ES	Spain	Coria del RÃo. Finca La Jampa		Se			1
00:00.0	ABH	54390-1	Specimen	Aegilops triuncialis L.	L.	ES	Spain	Villena; Arenal de la Virgen		A	38.61	-0.92	
00:00.0	ABH	10287-1	Specimen	Aegilops triuncialis L.	L.	ES	Spain	Alcoi; ctra. Alcoi-Benifallim		A	38.67	-0.45	
	FUND. BIODIVER	94086	Unknown	Aegilops triuncialis L.	L.	ESP	Spain	Aranda de Duero, hacia La Aguilera		Bu	41.1	-3.1	
00:00.0	ABH	2315-1	Specimen	Aegilops triuncialis L.	L.	ES	Spain	XÃ bia; Fontanelles		A	38.77	0.12	
00:00.0	COFC	50176-1	Specimen	Aegilops triuncialis L.	L.	ES	Spain	Valle del Guadiato; puente de la Ca		Co			1

	SIVIM	T-P16900:Aegi	Observation	Aegilops triuncialis L.	L.	ES	Spain	EstaciÃ³n de Tres Cantos			40.55	-3.82	0
	DEU146	AE 903	Specimen	Aegilops triuncialis L. subsp. triuncialis var. triu		ESP	Spain	Finca La Cigueela, stlich Aracena, Huelva					
00:00.0	ABH	55380-1	Specimen	Aegilops triuncialis L.	L.	ES	Spain	Villena; estrecho Pipa-Moratillas		A	38.62	-0.97	
00:00.0	ABH	8661-1	Specimen	Aegilops triuncialis L.	L.	ES	Spain	Confrides; SÂªAitana, prox.Font de		A	38.66	-0.29	
00:00.0	COA	28271-1	Specimen	Aegilops squarrosa L.	L.	ES	Spain	CÃ³rdoba, JardÃn BotÃ¡nico de CÃ		Co	37.84	-4.82	
00:00.0	COA	41149-1	Specimen	Aegilops triuncialis L.	L.	ES	Spain	A 2 Km de IznÃ¡jar desde Priego		Co	38.12	-4.37	
	DEU146	AE 907	Specimen	Aegilops triuncialis L. subsp. triuncialis var. triu		ESP	Spain	Strae Piedralaves - S. Martin de Viera, Avila					
	DEU146	AE 615	Specimen	Aegilops triuncialis subsp. triuncialis var. flaves		ESP	Spain	Guara					
	DEU146	AE 672	Specimen	Aegilops triuncialis subsp. triuncialis var. flaves		ESP	Spain	Huelma Andalusien					
	FUND. BIODIVER	1674409	Unknown	Aegilops triuncialis		ESP	Spain	Lucillos, hacia Cardiel de los Monte		To	39.1	-4.1	350
00:00.0	REDIAM-CMA	281967	Observation	Aegilops triuncialis		ESP	Spain		Hinojosa del Duq	Co	38.4647	-5.15766	550
00:00.0	COFC	11964-1	Specimen	Aegilops triuncialis L.	L.	ES	Spain	Almedinilla; La Carrasca; rÃo Alme		Co	37	-4	1
	SIVIM	T-P27672:Aegi	Observation	Aegilops triuncialis L.	L.	ES	Spain	Prados de la Nava, SÂª de Cabra			37.48	-4.47	1020
	SIVIM	T-P28637:Aegi	Observation	Aegilops triuncialis L.	L.	ES	Spain	Casatejada			39.8	-5.68	280
	SIVIM	T-P29360:Aegi	Observation	Aegilops triuncialis L.	L.	ES	Spain	Cerros de Vianos, Sierra de Alcaraz, Albac			38.57	-2.54	1250
	SIVIM	T-P30059:Aegi	Observation	Aegilops triuncialis L.	L.	ES	Spain	El Campichuelo, Cofrentes, Valencia			39.19	-1.14	0
	SIVIM	U-P03850:Aegi	Observation	Aegilops triuncialis L.	L.	ES	Spain	Salamanca: Puente del Congosto			40.44	-5.59	1000
00:00.0	COA	41181-1	Specimen	Aegilops triuncialis L.	L.	ES	Spain	A 8 Km de Santa Eufemia desde El		Co	38.47	-4.95	
00:00.0	MGC	28904-1	Unknown	Aegilops triuncialis L.	L.	ES	Spain	Marbella; San Pedro de AlcÃ¡ntara.		Ma	0	0	1
	ESP004	NC043488	Specimen	Aegilops triuncialis L.		ESP	Spain	Duruelo, province of Segovia			41.2333	-3.63333	1114
	ESP004	NC027417	Specimen	Aegilops triuncialis L.		ESP	Spain	Puebla de Don Fadrique, province of Grana			37.95	-2.43333	1164
	FUND. BIODIVER	118439	Unknown	Aegilops triuncialis L.	L.	ESP	Spain	La Pola de GordÃ³n, Santa LucÃa		Le	42.1	-5.1	
	FUND. BIODIVER	85961	Unknown	Aegilops triuncialis L.	L.	ESP	Spain	Casarejos		So	41.1	-2.1	
00:00.0	HUAL	5969-1	Specimen	Aegilops triuncialis L.	L.	ES	Spain	SÂª de GÃ¡dor, CaparidÃ¡n		Al	36.934	-2.905	
00:00.0	REDIAM-CMA	78017	Observation	Aegilops triuncialis		ESP	Spain		Villalba del Alcor	H	37.4537	-6.50635	108
00:00.0	REDIAM-CMA	107359	Observation	Aegilops triuncialis		ESP	Spain		Hornos	J	38.18	-2.64664	1430
00:00.0	SEV	98916-1	Specimen	Aegilops triuncialis L.	L.	ES	Spain	Entre Torrecampo y San Benito, RÃ		Co			1
00:00.0	SEV	98941-1	Specimen	Aegilops triuncialis L.	L.	ES	Spain	Bornos		Ca			1
	SIVIM	T-P10395:Aegi	Observation	Aegilops triuncialis L.	L.	ES	Spain	Proximidades del Pto. La Sardina. Ronda			36.66	-5.12	1150
	SIVIM	T-P11408:Aegi	Observation	Aegilops triuncialis L.	L.	ES	Spain	Tragacete			40.28	-1.82	1240
00:00.0	REDIAM-CMA	364148	Observation	Aegilops triuncialis		ESP	Spain		BÃ©dar	Al	37.2001	-1.99805	578
	FUND. BIODIVER	1369197	Unknown	Aegilops triuncialis L.	L.	ESP	Spain	Aldehuela de PeriÃ¡Ã±ez, hacia Alm		So	41.1	-2.1	1050
	FUND. BIODIVER	930416	Unknown	Aegilops triuncialis L.	L.	ESP	Spain	L'Espluga de FrancolÃ		T	41.1	0.1	
	FUND. BIODIVER	96899	Unknown	Aegilops triuncialis L.	L.	ESP	Spain	Fermoselle		Za			
	SIVIM	P-P08860:Aegi	Observation	Aegilops triuncialis L.	L.	ES	Spain	Font del Fangar, rodalies; Xerta			40.89	0.38	130
	SIVIM	U-P09311:Aegi	Observation	Aegilops triuncialis L.	L.	ES	Spain	Torres de Albarracin			40.37	-1.58	1200
	SIVIM	U-P09890:Aegi	Observation	Aegilops triuncialis L.	L.	ES	Spain	Cuneta a 7 Km de Ruidera			38.93	-2.88	0
00:00.0	REDIAM-CMA	142693	Observation	Aegilops triuncialis		ESP	Spain		Santa Olalla del C	H	37.9049	-6.21665	499
00:00.0	COFC	50172-1	Specimen	Aegilops triuncialis L.	L.	ES	Spain	Valle del Guadiato; puente de la Ca		Co			1
	SIVIM	T-P16886:Aegi	Observation	Aegilops triuncialis L.	L.	ES	Spain	Paracuellos de Jarama			40.46	-3.58	0
	SIVIM	T-P16914:Aegi	Observation	Aegilops triuncialis L.	L.	ES	Spain	Venta de Cabrejas			40.01	-2.41	0

00:00.0	FCO	20764-1	Specimen	Aegilops triuncialis L.	L.	ES	Spain	Madrid; Madrid, Ciudad Universitaria		M			
	BG-UPM	3054	Unknown	Aegilops triuncialis L.	L.	ESP	Spain	Ciudad Universitaria		M			
	SIVIM	T-P28879:Aegi	Observation	Aegilops triuncialis L.	L.	ES	Spain	Cerro el Cesto, Tomangordo			39.71	-5.79	270
	SIVIM	T-P30042:Aegi	Observation	Aegilops triuncialis L.	L.	ES	Spain	AlterÃ³n de Bucar, Jalance, Valencia			39.19	-1.14	330
00:00.0	SALA	78446-1	Specimen	Aegilops triuncialis L.	L.	ES	Spain	_; Castilblanco		Se			
00:00.0	MA	732357-1	Specimen	Aegilops triuncialis L.	L.	ES	Spain	Pinoso, Encebras.		A	38	0	
00:00.0	UNEX	10354-1	Observation	Aegilops triuncialis L.	_	ESP	Spain	Burguillos del Cerro: Finca 'Los Toc		Ba	38.4	-6.6	
00:00.0	COA	41238-1	Specimen	Aegilops triuncialis L.	L.	ES	Spain	Villanueva del RÃ-o		Co	38.2	-5.17	
	RUS001	VIR100602170	Specimen	Aegilops triuncialis L.		ESP	Spain						
	FUND. BIODIVER	1036001	Unknown	Aegilops triuncialis L.	L.	ESP	Spain	Sierra de Aguas, Carratraca		Ma	36.1	-4.1	
	FUND. BIODIVER	1043779	Unknown	Aegilops triuncialis L.	L.	ESP	Spain	Ronda, Sierra de las Nieves		Ma	36.1	-4.1	
	FUND. BIODIVER	1835130	Unknown	Aegilops triuncialis L.	L.	ESP	Spain	Lagunas de Ruidera, Ortigosa		CR	38.1	-2.1	
00:00.0	UNEX	10351-1	Observation	Aegilops triuncialis L.	_	ESP	Spain	Olivenza: Pantano de Piedra Aguda		Ba	38.7	-7.2	
00:00.0	REDIAM-CMA	383888	Observation	Aegilops triuncialis		ESP	Spain				37.6699	-3.42665	
00:00.0	REDIAM-CMA	386566	Observation	Aegilops triuncialis		ESP	Spain		Torres	J	37.7806	-3.49425	1146
00:00.0	SEV	99207-1	Specimen	Aegilops triuncialis L.	L.	ES	Spain	Above Grazalema		Ca			1400
	SIVIM	Q-P03038:Aegi	Observation	Aegilops triuncialis L.	L.	ES	Spain	St. MagÃ- de Brufaganya, prop del santuar			41.44	1.32	0
1876-06-0	W	43611	Unknown	Aegilops triuncialis L.		ESP	Spain	Hispania: In locis sterilibus prope Guadarrama.					
	SANT	38067	Specimen	Aegilops triuncialis L.		ES	Spain	Graus; entre Juseu y Aguinaliu		Hu			
00:00.0	MA	562394-1	Specimen	Aegilops triuncialis L.	L.	ES	Spain	Castell de Castells, S. d'Aixorta, Ce		A	38	0	
00:00.0	UNEX	01249-1	Observation	Aegilops triuncialis L.	_	ESP	Spain	Estepona: Sierra Bermeja de Estep		Ma	36.4762	-5.11215	
00:00.0	COA	41178-1	Specimen	Aegilops triuncialis L.	L.	ES	Spain	Km 7 de Montoro a Adamuz		Co	38.03	-4.48	
00:00.0	COA	41211-1	Specimen	Aegilops triuncialis L.	L.	ES	Spain	Puertollano		CR	38.66	-4.15	
00:00.0	SALA	77071-1	Specimen	Aegilops triuncialis L.	L.	ES	Spain	_; CÃ¡ceres		Cc	0	0	
00:00.0	GDA	GDA18850-1	Specimen	Aegilops triuncialis L.	L.	ES	Spain	Toledo, Calzada de Oropesa, Cerro		TO			0
00:00.0	GDAC	GDAC26138-1	Specimen	Aegilops triuncialis L.	L.	ES	Spain	Granada, SÂª de Baza, La Benajara		GR			1750
00:00.0	COA	41185-1	Specimen	Aegilops triuncialis L.	L.	ES	Spain	Cerca de EstaciÃ³n de BelalcÃ¡zar		Co	38.65	-5.18	
00:00.0	COA	41207-1	Specimen	Aegilops triuncialis L.	L.	ES	Spain	Sierra Madrona		CR	38.39	-4.26	
00:00.0	SEV	98910-1	Specimen	Aegilops triuncialis L.	L.	ES	Spain	Almodovar. Camino vecinal de los T		Co			1
00:00.0	SEV	99072-1	Specimen	Aegilops triuncialis L.	L.	ES	Spain	A 8 Km de El Pedroso de la Sierra h		Se			1
00:00.0	SALA	47670-1	Specimen	Aegilops triuncialis L.	L.	ES	Spain	_; Guijuelo		Sa			
00:00.0	MA	712089-1	Specimen	Aegilops triuncialis L.	L.	ES	Spain	Cabezarrubias del Puerto, Valle de		CR	38	-4	
00:00.0	SEV	98925-1	Specimen	Aegilops triuncialis L.	L.	ES	Spain	Grazalema. CercanÃ-as al CaserÃ-		Ca			800
00:00.0	SEV	99065-1	Specimen	Aegilops triuncialis L.	L.	ES	Spain	Entre PeÃ±arroya y El Hoyo, a la al		Co			1
	RUS001	VIR100602146	Specimen	Aegilops triuncialis L.		ESP	Spain						
	FUND. BIODIVER	1085277	Unknown	Aegilops triuncialis		ESP	Spain	RÃ-o Guadarrama		M	40.1	-3.1	
	FUND. BIODIVER	1093261	Unknown	Aegilops triuncialis		ESP	Spain	Valverde de la Vera, La Vega		Cc	39.1	-5.1	
	FUND. BIODIVER	1946446	Unknown	Aegilops triuncialis L.	L.	ESP	Spain	Lagunas de Ruidera, Ortigosa		CR	38.1	-2.1	
00:00.0	MGC	60318-1	Unknown	Aegilops triuncialis L.	L.	ES	Spain	Nerja; P. N. de las Sierras de Tejed		Ma	36.787	-3.79	500
00:00.0	MGC	63366-1	Unknown	Aegilops triuncialis L.	L.	ES	Spain	Jimera de LÃ-bar; El Chaparral		Ma	36.659	-5.288	420
	RUS001	VIR100602132	Specimen	Aegilops triuncialis L.		ESP	Spain						

	RUS001	VIR100602339	Specimen	Aegilops triuncialis L.		ESP	Spain						
	FUND. BIODIVER	1946439	Unknown	Aegilops triuncialis L.	L.	ESP	Spain	intercalacion basaltica del Alamillo		CR	38.1	-4.1	
	SIVIM	R-P10419:Aegi	Observation	Aegilops triuncialis L.	L.	ES	Spain	K 11 de la carretera del Picacho , AG			36.47	-5.67	0
00:00.0	FUND. BIODIVER	1374902	Unknown	Aegilops triuncialis L.	L.	ESP	Spain	Montes de Toledo, Parque Natural d		CR	39.1	-4.1	
00:00.0	FUND. BIODIVER	1473158	Unknown	Aegilops triuncialis L.	L.	ESP	Spain	Valle de Losa, Lastras de la Torre		Bu	42.1	-3.1	675
	FUND. BIODIVER	930418	Unknown	Aegilops triuncialis L.	L.	ESP	Spain	Valle de Castellfollit		T	41.1	0.1	
00:00.0	REDIAM-CMA	132648	Observation	Aegilops triuncialis		ESP	Spain		AznalcÃ³llar	Se	37.5319	-6.28601	100
00:00.0	COFC	50173-1	Specimen	Aegilops triuncialis L.	L.	ES	Spain	Valle del Guadiato; puente de la Ca		Co			1
	FUND. BIODIVER	56042	Unknown	Aegilops triuncialis L.	L.	ESP	Spain	Encinas de Esgueva, San Juna, El I		Va	41.1	-3.1	
00:00.0	COFC	29492-1	Specimen	Aegilops triuncialis L.	L.	ES	Spain	El Hopillo		Co			1
	SIVIM	T-P16891:Aegi	Observation	Aegilops triuncialis L.	L.	ES	Spain	Alcobendas			40.46	-3.7	0
	SIVIM	T-P16917:Aegi	Observation	Aegilops triuncialis L.	L.	ES	Spain	CobeÃ±a			40.55	-3.59	0
00:00.0	REDIAM-CMA	50817	Observation	Aegilops triuncialis		ESP	Spain		El Castillo de las	Se	37.6767	-6.20587	207
00:00.0	REDIAM-CMA	59121	Observation	Aegilops triuncialis		ESP	Spain		Tolox	Ma	36.6772	-4.91067	376
	SIVIM	S-P09959:Aegi	Observation	Aegilops triuncialis L.	L.	ES	Spain	prop de la font de l'Arbre, serra Aitana (Cor			38.63	-0.35	1200
	SIVIM	T-P05777:Aegi	Observation	Aegilops triuncialis L.	L.	ES	Spain	Embalse, HondÃ³n de las Nieves			38.19	-0.94	0
	SIVIM	T-P06428:Aegi	Observation	Aegilops triuncialis L.	L.	ES	Spain	Villargordo del Cabriel			39.46	-1.48	0
	SIVIM	T-P09143:Aegi	Observation	Aegilops triuncialis L.	L.	ES	Spain	PeÃ±aflor de Hornija			41.71	-5.04	0
	FUND. BIODIVER	1482316	Unknown	Aegilops triuncialis L.	L.	ESP	Spain	P.N. CabaÃ±eros		CR			
00:00.0	REDIAM-CMA	232681	Observation	Aegilops triuncialis		ESP	Spain		AlmadÃ©n de la P	Se	37.8842	-5.99772	423
00:00.0	REDIAM-CMA	236719	Observation	Aegilops triuncialis		ESP	Spain		Fuente Obejuna	Co	38.2289	-5.35487	589
00:00.0	COA	41186-1	Specimen	Aegilops triuncialis L.	L.	ES	Spain	A 8 Km de Santa Eufemia desde El		Co	38.47	-4.95	
	COA	41208-1	Specimen	Aegilops triuncialis L.	L.	ES	Spain	Ciudad Universitaria		M	40.38	-3.82	
00:00.0	SALA	66719-1	Specimen	Aegilops triuncialis L.	L.	ES	Spain	_; Calzada de Oropesa		To			
00:00.0	SEV	99067-1	Specimen	Aegilops triuncialis L.	L.	ES	Spain	Obejo. IntersecciÃ³n de los rÃ-os C		Co			1
00:00.0	BC	96441	Specimen	Aegilops triuncialis L.	L.	ES	Spain	Santa Coloma de Gramenet; Sta Co		B	41.5	2.22	
00:00.0	COFC	28813-1	Specimen	Aegilops triuncialis L.	L.	ES	Spain	Andujar; parque natural; coto 'Fonta		J			1
	FUND. BIODIVER	1551207	Unknown	Aegilops triuncialis		ESP	Spain	regno Legion. (Villafranca del Vierzo		Le			
	FUND. BIODIVER	993535	Unknown	Aegilops triuncialis		ESP	Spain	ValdepiÃ©lagos		Gu	40.1	-3.1	
	FUND. BIODIVER	99837	Unknown	Aegilops triuncialis L.	L.	ESP	Spain	Pozuelo de TÃ¡bara		Za	41.1	-5.1	
	SIVIM	T-P17787:Aegi	Observation	Aegilops triuncialis L.	L.	ES	Spain	Vaciamadrid			40.28	-3.58	0
	SIVIM	T-P19972:Aegi	Observation	Aegilops triuncialis L.	L.	ES	Spain	Coria			39.9	-6.54	263
	SIVIM	T-P20228:Aegi	Observation	Aegilops triuncialis L.	L.	ES	Spain	Entre Alcaudete y Martos			37.58	-4.13	0
	SIVIM	T-P26217:Aegi	Observation	Aegilops triuncialis L.	L.	ES	Spain	RincÃ³n de Benizar, Moratalla			38.21	-1.97	1000
	IDBD-GN	42517	Observation	Aegilops triuncialis L.	L.	ES	Spain	Selva	Petilla de AragÃ³r	Na	42.4486	-1.12254	950
00:00.0	W	42829	Unknown	Aegilops triuncialis L.		ESP	Spain	Catalogne: Barcelone, sables granitiques du Tibidabo.					
00:00.0	FCO	6559-1	Specimen	Aegilops triuncialis L.	L.	ES	Spain	Aranjuez; OntÃƒÂ-gola		M			
00:00.0	I.E.L.	139	Specimen	Aegilops triuncialis	L.	ES	Spain	Arenal de la Virge	Villena	ALICANTE			510
	ESP003	NC061689	Specimen	Aegilops triuncialis L.		ESP	Spain	Ciudad Universitaria, Madrid, province of Madrid					
	ESP004	NC050497	Specimen	Aegilops triuncialis L.		ESP	Spain	Beteta/Las Majadas 17km SE, Poyatos, pro			40.4167	-2.03333	1520
	ESP004	NC043497	Specimen	Aegilops triuncialis L.		ESP	Spain	Guadalajara, province of Guadalajara			40.6333	-3.15	685

00:00.0	FUND. BIODIVER	1946460	Unknown	Aegilops triuncialis L.	L.	ESP	Spain	Villanueva de los Infantes, rio Jabal		CR	38.1	-2.1	820
	FUND. BIODIVER	58085	Unknown	Aegilops triuncialis L.	L.	ESP	Spain	Cantalapiedra		Sa	40.1	-5.1	
	FUND. BIODIVER	62024	Unknown	Aegilops triuncialis L.	L.	ESP	Spain	Pastores, Cabezal Viejo		Sa	40.1	-6.1	
00:00.0	REDIAM-CMA	32664	Observation	Aegilops triuncialis		ESP	Spain		Chiclana de Segu	J	38.3857	-2.9159	644
	IPK	70863	Living	Aegilops triuncialis L.		ESP	Spain	Puerto Real (Cadiz)					
00:00.0	SEV	101359-1	Specimen	Aegilops triuncialis L.	L.	ES	Spain	Sierra de Rute. Barranco de las CÃ		Co			1
	SIVIM	S-P14067:Aegi	Observation	Aegilops triuncialis L.	L.	ES	Spain	Freixo do Meio			39.38	-8.88	144
	SIVIM	T-P06418:Aegi	Observation	Aegilops triuncialis L.	L.	ES	Spain	Alcotas			39.82	-1.01	0
	SIVIM	T-P09348:Aegi	Observation	Aegilops triuncialis L.	L.	ES	Spain	Torrelaguna			40.73	-3.59	0
00:00.0	MGC	45951-1	Unknown	Aegilops triuncialis L.	L.	ES	Spain	Ronda; P. N. Sierra de las Nieves. E		Ma	36.6	-5.063	1150
00:00.0	HUAL	13242-1	Specimen	Aegilops triuncialis L.	L.	ES	Spain	SÂª de los Filabres		Al	37.221	-2.42	1600
00:00.0	FCO	20764-1	Specimen	Aegilops triuncialis L.	L.	ES	Spain	Madrid; Madrid, Ciudad Universitaria		M			
	FUND. BIODIVER	1487852	Unknown	Aegilops triuncialis L.	L.	ESP	Spain	P.N. Sierra Nevada		Gr	36.1	-3.1	
	FUND. BIODIVER	987988	Unknown	Aegilops triuncialis L.	L.	ESP	Spain	CaÃ±amaque		So	41.1	-2.1	
00:00.0	COA	41155-1	Specimen	Aegilops triuncialis L.	L.	ES	Spain	Junto Venta de Azuel		Co	38.3	-4.37	
00:00.0	COA	41148-1	Specimen	Aegilops triuncialis L.	L.	ES	Spain	Km 4 de Bujalance a CaÃ±ete		Co	37.85	-4.36	
00:00.0	REDIAM-CMA	211777	Observation	Aegilops triuncialis		ESP	Spain		Hornachuelos	Co	37.9187	-5.32128	374
00:00.0	REDIAM-CMA	237158	Observation	Aegilops triuncialis		ESP	Spain		Fuente Obejuna	Co	38.2146	-5.43745	596
00:00.0	ABH	14378-1	Specimen	Aegilops triuncialis L.	L.	ES	Spain	Corduente; pr. Monte Coronado		Gu	40.82	-1.98	
00:00.0	SEV	49884-1	Specimen	Aegilops triuncialis L.	L.	ES	Spain	Entre Aracena y Los Marines		H			1
00:00.0	HUAL	1142-1	Specimen	Aegilops triuncialis L.	L.	ES	Spain	SÂª del Pozo, Nava del Espino		J	37.907	-2.892	
00:00.0	HSS	2688	Specimen	Aegilops triuncialis L.	L.	ES	Spain	Malcocinado		Ba	38.1423	-5.68158	
00:00.0	MGC	7596-1	Unknown	Aegilops triuncialis L.	L.	ES	Spain	RÃo PadrÃ³n		Ma	36.44	-5.18	1
	RUS001	VIR100602165	Specimen	Aegilops triuncialis L.		ESP	Spain						
00:00.0	FUND. BIODIVER	1835134	Unknown	Aegilops triuncialis L.	L.	ESP	Spain	Puebla del Principe, cercanias del c		CR	38.1	-2.1	940
00:00.0	ABH	53040-1	Specimen	Aegilops triuncialis L.	L.	ES	Spain	Villena; Arenal de la Virgen, Casa d		A	38.61	-0.93	
00:00.0	REDIAM-CMA	386669	Observation	Aegilops triuncialis		ESP	Spain		Villanueva del Re	Co	38.133	-5.17966	601
	IPK	AE 903	Living	Aegilops triuncialis L. subsp. triuncialis var. triuncialis			Spain	Finca La Cigue?ela, ostlich Aracena, Huelv			37.8919	-6.52083	
	SIVIM	Q-P03616:Aegi	Observation	Aegilops triuncialis L.	L.	ES	Spain	Villamalur			39.99	-0.42	0
	RUS001	VIR100602163	Specimen	Aegilops triuncialis L.		ESP	Spain						
	FUND. BIODIVER	1036796	Unknown	Aegilops triuncialis L.	L.	ESP	Spain	Sierra Tejeda		Ma	36.1	-3.1	
	FUND. BIODIVER	1067623	Unknown	Aegilops triuncialis		ESP	Spain	Coria del RÃo		Se	37.1	-5.1	
	FUND. BIODIVER	1073155	Unknown	Aegilops triuncialis		ESP	Spain	Puente Genil		Co	37.1	-4.1	
00:00.0	FUND. BIODIVER	1835136	Unknown	Aegilops triuncialis L.	L.	ESP	Spain	Solana del Pino, Alhorin		CR	38.1	-3.1	660
00:00.0	SALA	30599-1	Specimen	Aegilops triuncialis L.	L.	ES	Spain	_; Castrillo de la GuareÃ±a		Za			
00:00.0	FUND. BIODIVER	1369175	Unknown	Aegilops triuncialis L.	L.	ESP	Spain	Olmillos		So	41.1	-2.1	860
00:00.0	MA	528493-1	Specimen	Aegilops triuncialis L.	L.	ES	Spain	Alcaraz, Srra. de Alcaraz, Los Batan		Ab	38	-2	
	SIVIM	T-P09352:Aegi	Observation	Aegilops triuncialis L.	L.	ES	Spain	Alpedrete de la Sierra			40.83	-3.47	0
	SIVIM	T-P11350:Aegi	Observation	Aegilops triuncialis L.	L.	ES	Spain	Puerto de Cabrejas			40.01	-2.29	1000
	SANT	14572	Specimen	Aegilops triuncialis L.		ES	Spain	San MartÃn de la Vega,Cerro Buta		M			
00:00.0	GDAC	GDAC38658-1	Specimen	Aegilops triuncialis L.	L.	ES	Spain	AlmerÃa, OcaÃ±a, Rambla prox. al		AL			0

00:00.0	REDIAM-CMA	205950	Observation	Aegilops triuncialis		ESP	Spain		Cazalla de la Sier	Se	37.8846	-5.74826	472
00:00.0	REDIAM-CMA	236030	Observation	Aegilops triuncialis		ESP	Spain		Almonaster la Rea	H	37.8696	-6.75321	592
	SIVIM	T-P19062:Aegi	Observation	Aegilops triuncialis L.	L.	ES	Spain	Blanquizares de Gor			37.31	-3	1300
	SIVIM	T-P20232:Aegi	Observation	Aegilops triuncialis L.	L.	ES	Spain	Inmediaciones de Puente Genil			37.3	-4.8	150
	SIVIM	T-P26236:Aegi	Observation	Aegilops triuncialis L.	L.	ES	Spain	Sierra de la Zarza, Caravaca			38.03	-1.86	1300
00:00.0	MGC	59933-1	Unknown	Aegilops triuncialis L. subsp. triun	L.	ES	Spain	Arenas del Rey; P. N. de las Sierras		Gr	36.903	-3.87	1000
00:00.0	MGC	61818-1	Unknown	Aegilops triuncialis L.	L.	ES	Spain	Torcal de Antequera		Ma	36.96	-4.556	1200
00:00.0	FCO	24488-1	Specimen	Aegilops triuncialis L.	L.	ES	Spain	Olmedo; Olmedo, proximidades		Va			
00:00.0	REDIAM-CMA	149391	Observation	Aegilops triuncialis		ESP	Spain		AlanÃs	Se	38.0068	-5.57581	690
00:00.0	REDIAM-CMA	161387	Observation	Aegilops triuncialis		ESP	Spain		Zufre	H	37.9228	-6.40132	412
	SIVIM	T-P16897:Aegi	Observation	Aegilops triuncialis L.	L.	ES	Spain	Villaviciosa de OdÃ³n			40.28	-3.94	0
	SIVIM	T-P16924:Aegi	Observation	Aegilops triuncialis L.	L.	ES	Spain	CeclavÃn			39.81	-6.78	0
	FUND. BIODIVER	1674408	Unknown	Aegilops triuncialis		ESP	Spain	Lucillos, Arroyo de Ventalana		To	39.1	-4.1	450
00:00.0	COFC	36339-1	Specimen	Aegilops triuncialis L.	L.	ES	Spain	Rute; alrededores del municipio		Co			1
00:00.0	REDIAM-CMA	244041	Observation	Aegilops triuncialis		ESP	Spain		Aroche	H	37.9473	-6.89503	399
	SIVIM	T-P27668:Aegi	Observation	Aegilops triuncialis L.	L.	ES	Spain	La Nava, SÂª de Cabra			37.48	-4.47	1020
	SIVIM	T-P28636:Aegi	Observation	Aegilops triuncialis L.	L.	ES	Spain	El Ejido, Saucedilla			39.8	-5.68	280
	SIVIM	T-P30058:Aegi	Observation	Aegilops triuncialis L.	L.	ES	Spain	El Campichuelo, Cofrentes, Valencia			39.19	-1.14	0
	SIVIM	U-P02915:Aegi	Observation	Aegilops triuncialis L.	L.	ES	Spain	La Xara XÃ bia			38.72	0	0
00:00.0	MGC	35481-1	Unknown	Aegilops triuncialis L.	L.	ES	Spain	Tolox; Sierra de Tolox		Ma	0	0	1
	FUND. BIODIVER	998072	Unknown	Aegilops triuncialis		ESP	Spain	El Ventorro a Villalba		Cu	40.1	-1.1	
	ESP004	NC043491	Specimen	Aegilops triuncialis L.		ESP	Spain	Minas de Aldeamoret, Caceres, province of			39.45	-6.38333	451
00:00.0	COA	41187-1	Specimen	Aegilops triuncialis L.	L.	ES	Spain	Km 10 de Adamuz a Montoro		Co	38.03	-4.48	
00:00.0	SEV	58996-1	Specimen	Aegilops triuncialis L.	L.	ES	Spain	Algodonales, Sierra de LÃjar		Ca			500
00:00.0	FUND. BIODIVER	1369179	Unknown	Aegilops triuncialis L.	L.	ESP	Spain	Ucero		So	41.1	-2.1	1000
	FUND. BIODIVER	78796	Unknown	Aegilops triuncialis L.	L.	ESP	Spain	PeÃ±aflor de Hornija		Va	41.1	-4.1	
00:00.0	REDIAM-CMA	90987	Observation	Aegilops triuncialis		ESP	Spain		Almonte	H	37.2363	-6.44773	50
	BDBCV-General	76808	Observation	Aegilops triuncialis		ESPA	Spain	El Toro	El Alto Palancia	Castel	39.952	-0.71736	
	SIVIM	T-P09354:Aegi	Observation	Aegilops triuncialis L.	L.	ES	Spain	ValdepeÃ±as de la Sierra			40.83	-3.47	0
	SIVIM	T-P11405:Aegi	Observation	Aegilops triuncialis L.	L.	ES	Spain	Cuenca			40.01	-2.17	0
	ESP004	NC024039	Specimen	Aegilops triuncialis L.		ESP	Spain	El Molinillo, Huetor-Santillan, province of G			37.3	-3.43333	786
00:00.0	MGC	6977-1	Unknown	Aegilops triuncialis L.	L.	ES	Spain	Madrid; Ciudad Universitaria		M	0	0	1
	FUND. BIODIVER	80852	Unknown	Aegilops triuncialis L.	L.	ESP	Spain	SepÃºlveda, SepÃºlveda		Sg	41.1	-3.1	
	SEV	85162-1	Specimen	Aegilops triuncialis L.	L.	ES	Spain	El Garrobo		Se			1
00:00.0	SEV	98907-1	Specimen	Aegilops triuncialis L.	L.	ES	Spain	Galaroza. Los Batanes		H			1
00:00.0	SEV	99069-1	Specimen	Aegilops triuncialis L.	L.	ES	Spain	Priego de CÃ³rdoba, carretera de P		Co			1
00:00.0	REDIAM-CMA	78936	Observation	Aegilops triuncialis		ESP	Spain		Ronda	Ma	36.7041	-5.02604	1316
00:00.0	REDIAM-CMA	93225	Observation	Aegilops triuncialis		ESP	Spain		BaÃ±os de la Enc	J	38.3238	-3.81441	593
	SIVIM	T-P10778:Aegi	Observation	Aegilops triuncialis L.	L.	ES	Spain	Pozuelo de TÃ¡bara			41.78	-6	0
	SIVIM	T-P11867:Aegi	Observation	Aegilops triuncialis L.	L.	ES	Spain	Ardoncino			42.42	-5.67	0
00:00.0	COFC	26934-1	Specimen	Aegilops triuncialis L.	L.	ES	Spain	CardeÃƒÂ±a; coto 'Los Atalayones'		Co			1

	FUND. BIODIVER	1370916	Unknown	Aegilops triuncialis L.	L.	ESP	Spain	RedueÃ±a		M	40.1	-3.1	
	FUND. BIODIVER	1463958	Unknown	Aegilops triuncialis L.	L.	ESP	Spain	Les Planes de CastellÃ³ de Farfany		L	41.1	0.1	360
	FUND. BIODIVER	931851	Unknown	Aegilops triuncialis L.	L.	ESP	Spain	Tarragona		T	40.1	1.1	
00:00.0	REDIAM-CMA	146460	Observation	Aegilops triuncialis		ESP	Spain		Hornachuelos	Co	37.8892	-5.42192	300
	SIVIM	T-P16905:Aegi	Observation	Aegilops triuncialis L.	L.	ES	Spain	Colmenar Viejo, rÃ-o Manzanares			40.64	-3.82	0
00:00.0	SALA	12856-1	Specimen	Aegilops triuncialis L.	L.	ES	Spain	_; Esquivias		To			
00:00.0	SALA	16061-1	Specimen	Aegilops triuncialis L.	L.	ES	Spain	_; San Felices de los Gallegos		Sa			
	FUND. BIODIVER	1554592	Unknown	Aegilops triuncialis L.	L.	ESP	Spain	LeÃ³n		Le	42.1	-5.1	
00:00.0	MGC	59991-1	Unknown	Aegilops triuncialis L.	L.	ES	Spain	Sedella; P. N. de las Sierras de Teje		Ma	36.875	-4.049	960
00:00.0	REDIAM-CMA	208511	Observation	Aegilops triuncialis		ESP	Spain		Constantina	Se	37.9391	-5.55352	669
00:00.0	REDIAM-CMA	232648	Observation	Aegilops triuncialis		ESP	Spain		Aroche	H	38.0747	-6.97116	382
	SIVIM	T-P19655:Aegi	Observation	Aegilops triuncialis L.	L.	ES	Spain	Camino de Los Toros (AlmodÃ³var)			37.83	-5.04	130
	SALA	58051-1	Specimen	Aegilops triuncialis L.	L.	ES	Spain	_;		??			
00:00.0	SEV	49883-1	Specimen	Aegilops triuncialis L.	L.	ES	Spain	Entre Valdezufre e Higuera de la Si		H			1
00:00.0	FUND. BIODIVER	1684423	Unknown	Aegilops triuncialis L.	L.	ESP	Spain	Sierra de Zafalgar, La Camilla		Ca	36.1	-5.1	
	BDBCV-General	74117	Observation	Aegilops triuncialis		ESPA	Spain	Torrechiva	El Alto Mijares	Castel	40.0346	-0.36305	
00:00.0	COFC	12020-1	Specimen	Aegilops triuncialis L.	L.	ES	Spain	Priego de CÃƒÂ³rdoba; Sierra de la		Co	37	-4	1
00:00.0	REDIAM-CMA	251091	Observation	Aegilops triuncialis		ESP	Spain		AlÃ¡jar	H	37.8286	-6.63232	457
	SIVIM	T-P28624:Aegi	Observation	Aegilops triuncialis L.	L.	ES	Spain	Majadas de la Cruz, TorrejÃ³n el Rubio			39.71	-6.2	340
	SIVIM	T-P30046:Aegi	Observation	Aegilops triuncialis L.	L.	ES	Spain	Pr. Casa de AntÃ³n, Jalance, Valencia			39.19	-1.14	0
00:00.0	COA	41190-1	Specimen	Aegilops triuncialis L.	L.	ES	Spain	Km 10 de Espiel a Alcaracejos		Co	38.2	-5.06	
00:00.0	SALA	77295-1	Specimen	Aegilops triuncialis L.	L.	ES	Spain	_; JaraÃ-z de la Vera		Cc	40.06	-5.77	
00:00.0	SEV	98913-1	Specimen	Aegilops triuncialis L.	L.	ES	Spain	SeseÃ±a		M			1
00:00.0	SEV	98938-1	Specimen	Aegilops triuncialis L.	L.	ES	Spain	Villa del RÃ-o		Co			1
	RUS001	VIR100602134	Specimen	Aegilops triuncialis L.		ESP	Spain						
	FUND. BIODIVER	1077310	Unknown	Aegilops triuncialis L.	L.	ESP	Spain	Polan, Ventosilla		To	39.1	-3.1	
	FUND. BIODIVER	1109626	Unknown	Aegilops triuncialis L.	L.	ESP	Spain	DoÃ±inos de Salamanca		Sa	40.1	-5.1	
	FUND. BIODIVER	1946437	Unknown	Aegilops triuncialis L.	L.	ESP	Spain	Despenaperros		CR	38.1	-3.1	
	BG-UPM	2961	Unknown	Aegilops triuncialis L.	L.	ESP	Spain	Puerto Real		Ca			
00:00.0	REDIAM-CMA	405187	Observation	Aegilops triuncialis		ESP	Spain		Villanueva del Arz	J	38.1642	-2.89877	797
00:00.0	REDIAM-CMA	411235	Observation	Aegilops triuncialis		ESP	Spain		FondÃ³n	Al	37.05	-2.83473	1804
	SIVIM	R-P10311:Aegi	Observation	Aegilops triuncialis L.	L.	ES	Spain	Del Picacho a AlcalÃ¡ de los Gazules			36.47	-5.79	0
	SIVIM	S-P01265:Aegi	Observation	Aegilops triuncialis L.	L.	ES	Spain	l'Espluga Calba, els Graus			41.44	0.96	0
00:00.0	FUND. BIODIVER	1473159	Unknown	Aegilops triuncialis L.	L.	ESP	Spain	Sotresgudo, CaserÃ-o de Monte Re		Bu	42.1	-4.1	910
	FUND. BIODIVER	918139	Unknown	Aegilops triuncialis L.	L.	ESP	Spain	Garriga		B	41.1	2.1	
	FUND. BIODIVER	930419	Unknown	Aegilops triuncialis L.	L.	ESP	Spain	Plans de Prades, Plans Pages		T	41.1	1.1	
00:00.0	REDIAM-CMA	143538	Observation	Aegilops triuncialis		ESP	Spain		Santa Olalla del C	H	37.9043	-6.20472	500
	SIVIM	T-P16894:Aegi	Observation	Aegilops triuncialis L.	L.	ES	Spain	Villanueva del Pardillo			40.46	-4.06	0
	SIVIM	T-P16918:Aegi	Observation	Aegilops triuncialis L.	L.	ES	Spain	Ciudad Universitaria			40.37	-3.7	0
00:00.0	MA	753630-1	Specimen	Aegilops triuncialis L.	L.	ES	Spain	Cortijo de la Fuente del Lobo		Gr			
00:00.0	GDA	GDA16061-1	Specimen	Aegilops triuncialis L.	L.	ES	Spain	Granada, SÂª de Madrid, antes del		GR			1200

00:00.0	REDIAM-CMA	266907	Observation	Aegilops triuncialis		ESP	Spain		Espiel	Co	38.0882	-4.96259	483
00:00.0	REDIAM-CMA	276243	Observation	Aegilops triuncialis		ESP	Spain		Pedroche	Co	38.4826	-4.76265	525
00:00.0	REDIAM-CMA	281775	Observation	Aegilops triuncialis		ESP	Spain		Hinojosa del Duqu	Co	38.5094	-5.07529	538
	SIVIM	T-P27401:Aegi	Observation	Aegilops triuncialis L.	L.	ES	Spain	Albares			40.29	-3.11	0
	SIVIM	T-P28632:Aegi	Observation	Aegilops triuncialis L.	L.	ES	Spain	Cerro del Cesto, Romangordo			39.71	-5.79	290
	SIVIM	T-P28916:Aegi	Observation	Aegilops triuncialis L.	L.	ES	Spain	Colmenar del Negrete, Serradilla			39.8	-6.19	200
	SIVIM	T-P30054:Aegi	Observation	Aegilops triuncialis L.	L.	ES	Spain	Pr. Corral Confite, Villamalea, Albacete			39.29	-1.6	0
	SIVIM	U-P06777:Aegi	Observation	Aegilops triuncialis L.	L.	ES	Spain	Lagos e Relvas			37.04	-7.98	220
	ESP004	NC043487	Specimen	Aegilops triuncialis L.		ESP	Spain	Fuencaliente, province of Ciudad Real			38.4	-4.3	700
	BC	92742	Specimen	Aegilops triuncialis L.	L.	ES	Spain	Lleida; Raimat Lleida		L	41.66	0.54	
	FUND. BIODIVER	78965	Unknown	Aegilops triuncialis L.	L.	ESP	Spain	Cabrerizos		Sa	40.1	-5.1	
00:00.0	REDIAM-CMA	78359	Observation	Aegilops triuncialis		ESP	Spain		Tolox	Ma	36.691	-5.01562	1699
00:00.0	REDIAM-CMA	97639	Observation	Aegilops triuncialis		ESP	Spain		Castilblanco de lo	Se	37.7047	-5.96785	295
00:00.0	REDIAM-CMA	108006	Observation	Aegilops triuncialis		ESP	Spain		La Granada de R.	H	37.7568	-6.48866	507
	SIVIM	T-P10772:Aegi	Observation	Aegilops triuncialis L.	L.	ES	Spain	Dehesa de Misleo (Moreruela de TÃ¡bara)			41.78	-5.88	0
	SIVIM	T-P11410:Aegi	Observation	Aegilops triuncialis L.	L.	ES	Spain	Sierra de San Felipe			40.37	-1.93	1370
00:00.0	HUAL	9706-1	Specimen	Aegilops triuncialis L.	L.	ES	Spain	LubrÃn; Sierra de BÃ©dar. El Cam		Al	37.2	-2.03	580
	RUS001	VIR100602131	Specimen	Aegilops triuncialis L.		ESP	Spain						
	RUS001	VIR100602067	Specimen	Aegilops triuncialis L.		ESP	Spain						
00:00.0	SALA	122459-1	Specimen	Aegilops triuncialis L.	L.	ES	Spain	Monterrubio de la ArmuÃ±a, arenal		Sa			
	FUND. BIODIVER	1111151	Unknown	Aegilops triuncialis L.	L.	ESP	Spain	Fuentesclaras de Arriba		Av	40.1	-4.1	
00:00.0	FUND. BIODIVER	1946440	Unknown	Aegilops triuncialis L.	L.	ESP	Spain	Piedrabuena, cunetas de la carreter		CR	39.1	-4.1	700
00:00.0	REDIAM-CMA	24611	Observation	Aegilops triuncialis		ESP	Spain		Jimena	J	37.8178	-3.46975	829
00:00.0	REDIAM-CMA	401578	Observation	Aegilops triuncialis		ESP	Spain		El Castillo de las	Se	37.6824	-6.39084	346
00:00.0	REDIAM-CMA	415582	Observation	Aegilops triuncialis		ESP	Spain		Pozo AlcÃ³n	J	37.771	-2.90483	950
00:00.0	REDIAM-CMA	420115	Observation	Aegilops triuncialis		ESP	Spain		La Granada de R.	H	37.7688	-6.51262	453
	SIVIM	R-P11631:Aegi	Observation	Aegilops triuncialis L.	L.	ES	Spain	Jerez de la Frontera, Casas del Corchadill			36.65	-6.2	0
	FUND. BIODIVER	1001974	Unknown	Aegilops triuncialis		ESP	Spain	Tragacete		Cu	40.1	-1.1	
	FUND. BIODIVER	1030246	Unknown	Aegilops triuncialis L.	L.	ESP	Spain	Noalejo, Santa MercÃ©		J	37.1	-3.1	1100
00:00.0	REDIAM-CMA	370152	Observation	Aegilops triuncialis		ESP	Spain		Villanueva de CÃ	Co	38.3466	-4.53049	668
00:00.0	REDIAM-CMA	382039	Observation	Aegilops triuncialis		ESP	Spain				37.8829	-3.41005	
	SIVIM	U-P08621:Aegi	Observation	Aegilops triuncialis L.	L.	ES	Spain	La Cella (Jumilla)			38.38	-1.51	0
	SIVIM	U-P09878:Aegi	Observation	Aegilops triuncialis L.	L.	ES	Spain	Cuneta en'Casas Blancas'			38.84	-3	0
	FUND. BIODIVER	1120228	Unknown	Aegilops triuncialis L.	L.	ESP	Spain	Algodre, Lagunas de Algodre		Za	41.1	-5.1	
00:00.0	FUND. BIODIVER	1946453	Unknown	Aegilops triuncialis L.	L.	ESP	Spain	San Lorenzo de Calatrava, umbria d		CR	38.1	-3.1	620
	FUND. BIODIVER	51243	Unknown	Aegilops triuncialis L.	L.	ESP	Spain	Almenara de Tormes, tesos		Sa	40.1	-5.1	
	FUND. BIODIVER	56192	Unknown	Aegilops triuncialis L.	L.	ESP	Spain	Aguasal		Va	41.1	-4.1	
	FUND. BIODIVER	60945	Unknown	Aegilops triuncialis L.	L.	ESP	Spain	San Esteban de la Sierra		Sa	40.1	-5.1	
	SIVIM	T-P06963:Aegi	Observation	Aegilops triuncialis L.	L.	ES	Spain	VillamaÃ±Ã¡n			42.24	-5.66	0
	SIVIM	T-P06964:Aegi	Observation	Aegilops triuncialis L.	L.	ES	Spain	Castrofuerte			42.15	-5.54	0
00:00.0	GDA	GDA15837-1-1	Specimen	Aegilops triuncialis L.	L.	ES	Spain	Granada, SÂª Sagra, HuÃ©scar, co		GR			0

00:00.0	ABH	10096-1	Specimen	Aegilops triuncialis L.	L.	ES	Spain	Petrer; camino Petrer-RincÃ³n Bello		A	38.48	-0.72	
00:00.0	ABH	10814-1	Specimen	Aegilops triuncialis L.	L.	ES	Spain	Villena; Arenal de la Virgen		A	38.61	-0.93	
00:00.0	GDAC	GDAC30982-1	Specimen	Aegilops triuncialis L.	L.	ES	Spain	JaÃ©n, EStaciÃ³n de ferrocarril Lina		J			330
00:00.0	BC	839395	Specimen	Aegilops triuncialis L.	L.	ES	Spain	Casarabonela; Entre Casarramonel		Ma	36.748	-4.843	400
00:00.0	SALA	103274-1	Specimen	Aegilops triuncialis L.	L.	ES	Spain	Fermoselle		Za			
	IPK	32350	Living	Aegilops triuncialis L.		ESP	Spain	Penarroya-Pueblonuevo, Cordoba					
00:00.0	COFC	39804-1	Specimen	Aegilops triuncialis L.	L.	ES	Spain	Baena; cerro AlbendÃƒÂ-n		Co	37	-4	1
00:00.0	MGC	34649-1	Unknown	Aegilops triuncialis L.	L.	ES	Spain	Grazalema; Puerto de Las Cumbres		Ca	0	0	1
00:00.0	COA	41154-1	Specimen	Aegilops triuncialis L.	L.	ES	Spain	PeÃ±arroya-Pueblonuevo		Co	38.29	-5.29	
	FUND. BIODIVER	1554591	Unknown	Aegilops triuncialis L.	L.	ESP	Spain	Aranda		Bu	41.1	-3.1	
00:00.0	REDIAM-CMA	236585	Observation	Aegilops triuncialis		ESP	Spain		Fuente Obejuna	Co	38.2291	-5.35605	582
00:00.0	COFC	12026-1	Specimen	Aegilops triuncialis L.	L.	ES	Spain	Priego de CÃƒÂ³rdoba; entre Fuent		Co	37	-4	1
00:00.0	COFC	21307-1	Specimen	Aegilops triuncialis L.	L.	ES	Spain	Sierra de Hornachuelos; 'CanÃƒÂ³n		Co			1
	SIVIM	T-P19983:Aegi	Observation	Aegilops triuncialis L.	L.	ES	Spain	Almaraz			39.8	-5.68	320
	RUS001	VIR100602167	Specimen	Aegilops triuncialis L.		ESP	Spain						
	FUND. BIODIVER	1043781	Unknown	Aegilops triuncialis L.	L.	ESP	Spain	Sierra Tejeda		Ma	36.1	-3.1	
	FUND. BIODIVER	1835132	Unknown	Aegilops triuncialis L.	L.	ESP	Spain	Provincia de Ciudad Real		CR			
00:00.0	REDIAM-CMA	384974	Observation	Aegilops triuncialis		ESP	Spain		AlbÃ¡nchez de MÃ	J	37.7873	-3.48673	1200
00:00.0	REDIAM-CMA	386609	Observation	Aegilops triuncialis		ESP	Spain		Lora del RÃ-o	Se	37.6817	-5.56858	180
00:00.0	REDIAM-CMA	388256	Observation	Aegilops triuncialis		ESP	Spain		Torres	J	37.7454	-3.53909	1393
	SIVIM	Q-P03606:Aegi	Observation	Aegilops triuncialis L.	L.	ES	Spain	Villamalur			39.99	-0.42	0
	RUS001	VIR100602135	Specimen	Aegilops triuncialis L.		ESP	Spain						
	FUND. BIODIVER	1093251	Unknown	Aegilops triuncialis		ESP	Spain	Almaraz		Cc	39.1	-5.1	
00:00.0	FUND. BIODIVER	1946436	Unknown	Aegilops triuncialis L.	L.	ESP	Spain	Herencia, cerro Navajo, lad. SE reta		CR	39.1	-3.1	660
00:00.0	REDIAM-CMA	16033	Observation	Aegilops triuncialis		ESP	Spain		PeÃ±aflor	Se	37.7364	-5.36871	100
00:00.0	REDIAM-CMA	400665	Observation	Aegilops triuncialis		ESP	Spain		Santiago-Pontone	J	38.1233	-2.54809	1544
00:00.0	REDIAM-CMA	415525	Observation	Aegilops triuncialis		ESP	Spain		Cazorla	J	37.8459	-2.89269	1251
	SIVIM	S-P01255:Aegi	Observation	Aegilops triuncialis L.	L.	ES	Spain	Vinaixa, la Solana			41.44	0.84	0
	SIVIM	S-P03970:Aegi	Observation	Aegilops triuncialis L.	L.	ES	Spain	Vinaixa, la Solana			41.44	0.84	0
	SIVIM	S-P05566:Aegi	Observation	Aegilops triuncialis L.	L.	ES	Spain	Serreta Negra, Ã¡rea de Els Boixos			41.42	0	300
	ESP004	NC050471	Specimen	Aegilops triuncialis L.		ESP	Spain	Tarancon/Carrascosa del Campo 14km E,			40.0667	-2.86667	840
	FUND. BIODIVER	116425	Unknown	Aegilops triuncialis L.	L.	ESP	Spain	Almenara de Adaja		Va	41.1	-4.1	
00:00.0	FUND. BIODIVER	1946457	Unknown	Aegilops triuncialis L.	L.	ESP	Spain	Retuerta del Bullaque, Cabaneros		CR	39.1	-4.1	
	FUND. BIODIVER	57499	Unknown	Aegilops triuncialis L.	L.	ESP	Spain	Matilla de los CaÃ±os del rÃ-o		Sa	40.1	-5.1	
00:00.0	REDIAM-CMA	47931	Observation	Aegilops triuncialis		ESP	Spain		El Castillo de las	Se	37.7263	-6.25802	252
00:00.0	GDA	GDA30025-2-1	Specimen	Aegilops triuncialis L.	L.	ES	Spain	Granada, Iznalloz, Cueva del Agua		GR			1700
	SIVIM	S-P14062:Aegi	Observation	Aegilops triuncialis L.	L.	ES	Spain	Serrinha			38.39	-8.54	0
	SIVIM	T-P06371:Aegi	Observation	Aegilops triuncialis L.	L.	ES	Spain	Camporrobles			39.55	-1.48	0
	SIVIM	T-P09198:Aegi	Observation	Aegilops triuncialis L.	L.	ES	Spain	Mojados			41.35	-4.67	0
00:00.0	BC	627485	Specimen	Aegilops triuncialis L.	L.	ES	Spain	Bellmunt del Priorat; Vorada camÃ-		T	41.13	0.8	300
00:00.0	FCO	17836-1	Specimen	Aegilops triuncialis L.	L.	ES	Spain	Pinar de Selas		Gu	40.94	-2.13	

00:00.0	BC	96442	Specimen	Aegilops triuncialis L.	L.	ES	Spain	Terrassa; Les fonts de Terrassa		B	41.5	2.1	
00:00.0	SALA	93785-1	Specimen	Aegilops triuncialis L.	L.	ES	Spain	_; Villa de Ves		Ab	39.21	-1.29	
00:00.0	MA	589846-1	Specimen	Aegilops triuncialis L.	L.	ES	Spain	Hecho, EmbÃºn, convento del Pilar		Hu	42	0	
	ESP004	NC027447	Specimen	Aegilops triuncialis L.		ESP	Spain	Alcolea del Pinar, province of Guadalajara			41.0333	-2.45	1205
	ESP004	NC022311	Specimen	Aegilops triuncialis L.		ESP	Spain	Olvera, province of Cadiz			36.9333	-5.26667	623
	FUND. BIODIVER	76093	Unknown	Aegilops triuncialis L.	L.	ESP	Spain	Venialbo		Za	41.1	-5.1	
	FUND. BIODIVER	847028	Unknown	Aegilops triuncialis L.	L.	ESP	Spain	Valle d'Aguies Vives		V	38.1	-0.1	
	RUS001	VIR100602221	Specimen	Aegilops triuncialis L.		ESP	Spain						
	RUS001	VIR100602156	Specimen	Aegilops triuncialis L.		ESP	Spain						
	FUND. BIODIVER	1043771	Unknown	Aegilops triuncialis L.	L.	ESP	Spain	Archidona		Ma	36.1	-4.1	
	FUND. BIODIVER	1067630	Unknown	Aegilops triuncialis L.	L.	ESP	Spain	Pruna, AlgÃ¡mitas, Sierra del TablÃ		Se	36.1	-4.1	
00:00.0	FUND. BIODIVER	1835122	Unknown	Aegilops triuncialis L.	L.	ESP	Spain	Herencia, de Herencia a Villarta de		CR	39.1	-3.1	700
00:00.0	FUND. BIODIVER	1835143	Unknown	Aegilops triuncialis L.	L.	ESP	Spain	Villamanrique, arroyo de la Fuente d		CR	38.1	-2.1	840
	SIVIM	T-P10903:Aegi	Observation	Aegilops triuncialis L.	L.	ES	Spain	Valverde de la Vega, 'La Vega'			40.08	-5.46	300
	SIVIM	U-P13535:Aegi	Observation	Aegilops triuncialis L.	L.	ES	Spain	Braganga, Samil, Alto das Cantarias			41.71	-6.83	0
00:00.0	SEV	30542-1	Specimen	Aegilops triuncialis L.	L.	ES	Spain	Entre MorÃ³n y Pruna, alrededores		Se			1
00:00.0	COFC	12025-1	Specimen	Aegilops triuncialis L.	L.	ES	Spain	Priego de CÃƒÂ³rdoba; pico Leones		Co	37	-4	1
00:00.0	COFC	21306-1	Specimen	Aegilops triuncialis L.	L.	ES	Spain	Sierra de Hornachuelos; 'Mata Rom		Co			1
	CZE122	01C2107104	Specimen	Aegilops triuncialis L.		ESP	Spain	Guara, Spanien					
00:00.0	SEV	99066-1	Specimen	Aegilops triuncialis L.	L.	ES	Spain	Almedinilla, rÃo Almedinilla, en las		Co			1
	RUS001	VIR100602174	Specimen	Aegilops triuncialis L.		ESP	Spain						
	ESP004	NC043496	Specimen	Aegilops triuncialis L.		ESP	Spain	Adrada de Piron, province of Segovia			41.05	-4.03333	1019
	FUND. BIODIVER	1043775	Unknown	Aegilops triuncialis L.	L.	ESP	Spain	Estepona, Sierra Bermeja		Ma	36.1	-4.1	
	FUND. BIODIVER	1065852	Unknown	Aegilops triuncialis L.	L.	ESP	Spain	Sierra de Aracena		H	37.1	-6.1	
	FUND. BIODIVER	1072125	Unknown	Aegilops triuncialis L.	L.	ESP	Spain	Las Rozas, Encarnaciones, alreded		Se	36.1	-5.1	
00:00.0	FUND. BIODIVER	1835126	Unknown	Aegilops triuncialis L.	L.	ESP	Spain	Manzanares, de Almagro a Calzada		CR	38.1	-3.1	700
00:00.0	FUND. BIODIVER	1835147	Unknown	Aegilops triuncialis L.	L.	ESP	Spain	Albadalejo		CR	38.1	-2.1	900
	FUND. BIODIVER	1135155	Unknown	Aegilops triuncialis L.	L.	ESP	Spain	Chozas de Abajo		Le	42.1	-5.1	
	FUND. BIODIVER	1141562	Unknown	Aegilops triuncialis L.	L.	ESP	Spain	Ardoncino		Le	42.1	-5.1	830
00:00.0	FUND. BIODIVER	1946461	Unknown	Aegilops triuncialis L.	L.	ESP	Spain	Almagro, volcan de Yezosa, sobre e		CR	38.1	-3.1	853
00:00.0	REDIAM-CMA	386381	Observation	Aegilops triuncialis		ESP	Spain		Villanueva del Re	Co	38.2055	-5.11748	503
00:00.0	REDIAM-CMA	392443	Observation	Aegilops triuncialis		ESP	Spain		Cabra	Co	37.4996	-4.36915	1000
00:00.0	REDIAM-CMA	34339	Observation	Aegilops triuncialis		ESP	Spain		Torres	J	37.7434	-3.51158	1400
00:00.0	REDIAM-CMA	74736	Observation	Aegilops triuncialis		ESP	Spain		Huelma	J	37.6798	-3.47973	1199
	SIVIM	U-P13540:Aegi	Observation	Aegilops triuncialis L.	L.	ES	Spain	BraganÃ§a, Rabal, junto a um caminho			41.8	-6.83	0
	SIVIM	S-P14068:Aegi	Observation	Aegilops triuncialis L.	L.	ES	Spain	Almendres			42.99	-3.49	290
	SIVIM	T-P06420:Aegi	Observation	Aegilops triuncialis L.	L.	ES	Spain	Siete Aguas			39.46	-0.9	0
	SIVIM	T-P09349:Aegi	Observation	Aegilops triuncialis L.	L.	ES	Spain	ValdepeÃ±as de la Sierra			40.83	-3.47	0
00:00.0	GDA	GDA30029-1	Specimen	Aegilops triuncialis L.	L.	ES	Spain	JaÃ©n, SÂª MÃ¡gina, SÂª de la Cru		J			1000
00:00.0	COFC	41262-1	Specimen	Aegilops triuncialis L.	L.	ES	Spain	Puente Genil; rÃƒÂo Genil; cerro H		Co	37	-4	1
00:00.0	COFC	41300-1	Specimen	Aegilops triuncialis L.	L.	ES	Spain	Cabra; Atalayas		Co	37	-4	1

00:00.0	MGC	40316-1	Unknown	Aegilops triuncialis L.	L.	ES	Spain	Igualeja; Carretera C-339, Ronda-S		Ma	36.627	-5.063	1100
	FUND. BIODIVER	918145	Unknown	Aegilops triuncialis L.	L.	ESP	Spain	Montserrat		B	41.1	1.1	
00:00.0	REDIAM-CMA	135127	Observation	Aegilops triuncialis		ESP	Spain		Zufre	H	37.891	-6.39075	350
00:00.0	REDIAM-CMA	186567	Observation	Aegilops triuncialis		ESP	Spain		Cala	H	37.9759	-6.35518	594
	SIVIM	T-P16899:Aegi	Observation	Aegilops triuncialis L.	L.	ES	Spain	Tres Cantos			40.55	-3.82	0
	RUS001	VIR100602148	Specimen	Aegilops triuncialis L.		ESP	Spain						
	RUS001	VIR100602127	Specimen	Aegilops triuncialis L.		ESP	Spain						
	FUND. BIODIVER	1085275	Unknown	Aegilops triuncialis		ESP	Spain	Colmenar Viejo		M	40.1	-3.1	
	FUND. BIODIVER	1090390	Unknown	Aegilops triuncialis		ESP	Spain	CobeÃ±a		M	40.1	-3.1	
00:00.0	FUND. BIODIVER	1946444	Unknown	Aegilops triuncialis L.	L.	ESP	Spain	Moral de Calatrava, sierra de Moral		CR	38.1	-3.1	
00:00.0	REDIAM-CMA	26754	Observation	Aegilops triuncialis		ESP	Spain		Torres	J	37.7456	-3.53877	1393
00:00.0	REDIAM-CMA	403114	Observation	Aegilops triuncialis		ESP	Spain		El Real de la Jara	Se	37.9886	-6.05926	500
00:00.0	REDIAM-CMA	413749	Observation	Aegilops triuncialis		ESP	Spain		Villanueva de CÃ	Co	38.2941	-4.55084	732
00:00.0	REDIAM-CMA	416006	Observation	Aegilops triuncialis		ESP	Spain		Quesada	J	37.7659	-2.97587	1500
00:00.0	REDIAM-CMA	420721	Observation	Aegilops triuncialis		ESP	Spain		Hinojosa del Duq	Co	38.4795	-5.31862	528
00:00.0	S	S09-18310	Specimen	Aegilops triuncialis L.	L.	Spain	Spain	Hispania [Solander scripsit]					
	SIVIM	R-P06817:Aegi	Observation	Aegilops triuncialis L.	L.	ES	Spain	AlcalÃ¡ del JÃºcar, Albacete			39.1	-1.49	0
00:00.0	FCO	25921-1	Specimen	Aegilops triuncialis L.	L.	ES	Spain	Campos de Ledesma		Sa			
	FUND. BIODIVER	1674406	Unknown	Aegilops triuncialis		ESP	Spain	Cazalegas		To	39.1	-4.1	440
00:00.0	REDIAM-CMA	243894	Observation	Aegilops triuncialis		ESP	Spain		Aroche	H	37.9438	-6.88229	472
00:00.0	REDIAM-CMA	267591	Observation	Aegilops triuncialis		ESP	Spain		Santa Eufemia	Co	38.6112	-4.88565	484
00:00.0	REDIAM-CMA	276711	Observation	Aegilops triuncialis		ESP	Spain		Dos Torres	Co	38.5499	-4.82424	491
	SIVIM	T-P28634:Aegi	Observation	Aegilops triuncialis L.	L.	ES	Spain	La CaÃ±ada, Romangordo			39.71	-5.79	290
	SIVIM	T-P28919:Aegi	Observation	Aegilops triuncialis L.	L.	ES	Spain	Solana de la Parrilla, SerrejÃ³n			39.71	-5.91	240
	SIVIM	T-P30056:Aegi	Observation	Aegilops triuncialis L.	L.	ES	Spain	Los Callejones, Cofrentes, Valencia			39.19	-1.26	0
	SIVIM	U-P02908:Aegi	Observation	Aegilops triuncialis L.	L.	ES	Spain	Vall d'AlcalÃ , Beniaia			38.72	-0.35	0
00:00.0	COFC	41143-1	Specimen	Aegilops triuncialis L.	L.	ES	Spain	Cabra; Mojonera W; Torre del Puert		Co	37	-4	1
	IDBD-GN	42514	Observation	Aegilops triuncialis L.	L.	ES	Spain		MaÃ±eru	Na	42.6697	-1.84197	
00:00.0	BC	70834	Specimen	Aegilops triuncialis L.	L.	ES	Spain	Burgos; Orillas del ArlanzÃ³n en Bu		Bu	42.32	-3.67	
00:00.0	SALA	30603-1	Specimen	Aegilops triuncialis L.	L.	ES	Spain	_; Venialbo		Za			
00:00.0	BC	601436	Specimen	Aegilops triuncialis L.	L.	ES	Spain	l'Aleixar; Baix Camp: Conreus de l'A		T	41.22	1.03	265
00:00.0	REDIAM-CMA	277743	Observation	Aegilops triuncialis		ESP	Spain		Guadalcanal	Se	38.1607	-5.76724	542
00:00.0	REDIAM-CMA	282123	Observation	Aegilops triuncialis		ESP	Spain		Hinojosa del Duq	Co	38.4173	-5.09999	573
00:00.0	REDIAM-CMA	289015	Observation	Aegilops triuncialis		ESP	Spain		Chiclana de Segu	J	38.3905	-2.94034	699
	SIVIM	T-P28808:Aegi	Observation	Aegilops triuncialis L.	L.	ES	Spain	Arroyo de la Vid, TorrejÃ³n el Rubio			44.1	-14.74	260
	SIVIM	T-P29378:Aegi	Observation	Aegilops triuncialis L.	L.	ES	Spain	Sierra de GÃ¡dor			36.86	-2.55	1600
	SIVIM	T-P30062:Aegi	Observation	Aegilops triuncialis L.	L.	ES	Spain	Pr. Las Salinas, Casas de Ves, Albacete			39.28	-1.37	0
	ESP004	NC050489	Specimen	Aegilops triuncialis L.		ESP	Spain	casa forestal de Tejadillos, Cuenca, provin			40.4	-1.98333	1080
	ESP004	NC043493	Specimen	Aegilops triuncialis L.		ESP	Spain	Pedrosillo el Ralo, province of Salamanca			41.05	-5.53333	818
	FUND. BIODIVER	1135158	Unknown	Aegilops triuncialis L.	L.	ESP	Spain	ArdÃ³n		Le	42.1	-5.1	820
	FUND. BIODIVER	116787	Unknown	Aegilops triuncialis L.	L.	ESP	Spain	Ciruelos de Cervera, pie del Alto de		Bu	41.1	-3.1	

	FUND. BIODIVER	1946464	Unknown	Aegilops triuncialis L.	L.	ESP	Spain	Almuradiel-Venta de Cardenas		CR	38.1	-3.1	
	FUND. BIODIVER	58156	Unknown	Aegilops triuncialis L.	L.	ESP	Spain	Babilafuente		Sa	40.1	-5.1	
	FUND. BIODIVER	65366	Unknown	Aegilops triuncialis L.	L.	ESP	Spain	Guijuelo		Sa	40.1	-5.1	
00:00.0	REDIAM-CMA	35039	Observation	Aegilops triuncialis		ESP	Spain		Torres	J	37.7806	-3.49427	1146
00:00.0	REDIAM-CMA	49724	Observation	Aegilops triuncialis		ESP	Spain		Torres	J	37.7471	-3.52842	1500
00:00.0	REDIAM-CMA	58198	Observation	Aegilops triuncialis		ESP	Spain		Orcera	J	38.4633	-2.81491	753
	SIVIM	S-P14071:Aegi	Observation	Aegilops triuncialis L.	L.	ES	Spain	Almendres			42.99	-3.49	310
	SIVIM	T-P06425:Aegi	Observation	Aegilops triuncialis L.	L.	ES	Spain	Sot de Chera			39.55	-1.02	0
	RUS001	VIR100602147	Specimen	Aegilops triuncialis L.		ESP	Spain						
	RUS001	VIR100602126	Specimen	Aegilops triuncialis L.		ESP	Spain						
	FUND. BIODIVER	1085276	Unknown	Aegilops triuncialis		ESP	Spain	Ribatejada		M	40.1	-3.1	
00:00.0	FUND. BIODIVER	1946445	Unknown	Aegilops triuncialis L.	L.	ESP	Spain	Navas de Estena, arroyo de Ciguinu		CR	39.1	-4.1	780
00:00.0	REDIAM-CMA	409646	Observation	Aegilops triuncialis		ESP	Spain		Aldeaquemada	J	38.3999	-3.36747	689
00:00.0	SALA	91901-1	Specimen	Aegilops triuncialis L.	L.	ES	Spain	_; Petrer, prox. los Castellerets		A	38.49	-0.73	
00:00.0	COFC	41142-1	Specimen	Aegilops triuncialis L.	L.	ES	Spain	Luque; Laguna del Salobral		Co	37	-4	1
00:00.0	BC	92759	Specimen	Aegilops triuncialis L.	L.	ES	Spain	El Arahal; Emp. MorÃ³n CÃ¡diz		Se	37.15	-5.65	
	RUS001	VIR100602140	Specimen	Aegilops triuncialis L.		ESP	Spain						
00:00.0	REDIAM-CMA	15000	Observation	Aegilops triuncialis		ESP	Spain		Torres	J	37.7784	-3.50645	923
00:00.0	REDIAM-CMA	398408	Observation	Aegilops triuncialis		ESP	Spain		El Pedroso	Se	37.8208	-5.7107	396
	SIVIM	R-P09640:Aegi	Observation	Aegilops triuncialis L.	L.	ES	Spain	Atienza			41.19	-2.88	1160
	SIVIM	S-P00322:Aegi	Observation	Aegilops triuncialis L.	L.	ES	Spain	*			41.36	2.04	130
00:00.0	COA	41236-1	Specimen	Aegilops triuncialis L.	L.	ES	Spain	3 Km al norte de BelalcÃ¡zar		Co	38.56	-5.18	
00:00.0	COFC	41302-1	Specimen	Aegilops triuncialis L.	L.	ES	Spain	Cabra; los Cerrajones		Co	37	-4	1
00:00.0	GDA	GDA10270-1	Specimen	Aegilops triuncialis L.	L.	ES	Spain	Granada, AlmegÃ-jar, prÃ³x. barran		GR			900
00:00.0	MA	729745-1	Specimen	Aegilops triuncialis L.	L.	ES	Spain	Aldea del Rey, cerro de la Vaqueriz		CR	38	-3	
00:00.0	BC	70824	Specimen	Aegilops triuncialis L.	L.	ES	Spain	Madrid; El Prado		M	40.51	-3.77	
	FUND. BIODIVER	1370912	Unknown	Aegilops triuncialis L.	L.	ESP	Spain	Guadalix de la Sierra		M	40.1	-3.1	
	FUND. BIODIVER	1463956	Unknown	Aegilops triuncialis L.	L.	ESP	Spain	Congost de Santa Anna		L	41.1	0.1	320
00:00.0	SALA	40757-1	Specimen	Aegilops triuncialis L.	L.	ES	Spain	_; RÃ¡bano		Va			
00:00.0	SALA	46424-1	Specimen	Aegilops triuncialis L.	L.	ES	Spain	_; Cantalapiedra		Sa			
00:00.0	REDIAM-CMA	136699	Observation	Aegilops triuncialis		ESP	Spain		AlanÃ-s	Se	38.0121	-5.74397	600
	SIVIM	T-P16901:Aegi	Observation	Aegilops triuncialis L.	L.	ES	Spain	Hoyo de Manzanares			40.55	-3.94	0
00:00.0	GDA	GDA15569-1-1	Specimen	Aegilops triuncialis L.	L.	ES	Spain	Granada, SÂª de Madrid, Lagunazo		GR			1200
	IDBD-GN	42513	Observation	Aegilops triuncialis L.	L.	ES	Spain		SangÃ¼esa	Na	42.5719	-1.23458	
	SEV	11063-1	Specimen	Aegilops triuncialis L.	L.	ES	Spain	Inmediaciones de Sevilla		Se			1
	IPK	32114	Living	Aegilops triuncialis L. subsp. triuncialis var. fla		ESP	Spain	Huebma (Andalousie)					
	SANT	55882	Specimen	Aegilops triuncialis L.		ES	Spain	Sevilla, ctra. Utrera Km 1, Campus		Se			
	RUS001	VIR100602224	Specimen	Aegilops triuncialis L.		ESP	Spain						
	RUS001	VIR100602181	Specimen	Aegilops triuncialis L.		ESP	Spain						
	RUS001	VIR100602159	Specimen	Aegilops triuncialis L.		ESP	Spain						
	FCO	4045-1	Specimen	Aegilops triuncialis L.	L.	ES	Spain	Valdemoro		M			

	FUND. BIODIVER	1038226	Unknown	Aegilops triuncialis L.	L.	ESP	Spain	Canillas de Albaidia		Ma	36.1	-3.1	
	FUND. BIODIVER	1835119	Unknown	Aegilops triuncialis L.	L.	ESP	Spain	Ciudad Real		CR			
00:00.0	FUND. BIODIVER	1835140	Unknown	Aegilops triuncialis L.	L.	ESP	Spain	San Lorenzo de Calatrava, cerro de		CR	38.1	-3.1	820
	FUND. BIODIVER	1869151	Unknown	Aegilops triuncialis L.	L.	ESP	Spain	Ciudad Real, La Atalaya		CR	38.1	-3.1	670
00:00.0	GDAC	GDAC37347-1	Specimen	Aegilops triuncialis L.	L.	ES	Spain	AlmerÃ-a, SÂª de GÃ¡dor, La Parra		AL			1300
	IPK	32373	Living	Aegilops triuncialis L. subsp. triuncialis var. fla		ESP	Spain	Penarroya-Pueblonuevo, Cordoba					
	SIVIM	U-P13531:Aegi	Observation	Aegilops triuncialis L.	L.	ES	Spain	BraganÃ§a, Samil			41.71	-6.83	0
	FUND. BIODIVER	1630735	Unknown	Aegilops triuncialis L.	L.	ESP	Spain	RÃ-o MiÃ±o frente a Barbantes		Or	42.1	-7.1	
	FUND. BIODIVER	995301	Unknown	Aegilops triuncialis L.	L.	ESP	Spain	Almonacid del Marquesado y Villare		Cu	39.1	-2.1	
00:00.0	REDIAM-CMA	189480	Observation	Aegilops triuncialis		ESP	Spain		Abrucena	Al	37.1347	-2.8443	1300
00:00.0	REDIAM-CMA	208265	Observation	Aegilops triuncialis		ESP	Spain		Hornachuelos	Co	37.8895	-5.42266	300
00:00.0	REDIAM-CMA	232386	Observation	Aegilops triuncialis		ESP	Spain		AlmadÃ©n de la P	Se	37.8833	-5.99783	417
00:00.0	GDAC	GDAC26137-1	Specimen	Aegilops triuncialis L.	L.	ES	Spain	Granada, SÂª de Baza, Cerro de la		GR			0
00:00.0	BC	631616	Specimen	Aegilops triuncialis L.	L.	ES	Spain	l'Espluga Calba; Entre l'Espluga Ca		L	41.49	1.02	
	BC	70832	Specimen	Aegilops triuncialis L.	L.	ES	Spain	la Cellera de Ter; La Sellera		Ge	41.96	2.58	
00:00.0	REDIAM-CMA	241719	Observation	Aegilops triuncialis		ESP	Spain		El Real de la Jara	Se	37.9218	-6.13177	495
00:00.0	REDIAM-CMA	261984	Observation	Aegilops triuncialis		ESP	Spain		Pozoblanco	Co	38.3056	-4.7398	689
00:00.0	REDIAM-CMA	274353	Observation	Aegilops triuncialis		ESP	Spain		BelalcÃ¡zar	Co	38.5378	-5.09995	530
00:00.0	REDIAM-CMA	282229	Observation	Aegilops triuncialis		ESP	Spain		Villanueva del Du	Co	38.3783	-5.02697	606
	SIVIM	T-P28611:Aegi	Observation	Aegilops triuncialis L.	L.	ES	Spain	Saltos de TorrejÃ³n			39.8	-6.07	0
	SIVIM	T-P28876:Aegi	Observation	Aegilops triuncialis L.	L.	ES	Spain	La CaÃ±ada, Romangordo			39.71	-5.68	300
	SIVIM	T-P29483:Aegi	Observation	Aegilops triuncialis L.	L.	ES	Spain	Diferentes localidades de la Sierra del Agu			36.85	-4.79	0
	SIVIM	T-P30120:Aegi	Observation	Aegilops triuncialis L.	L.	ES	Spain	Cerro Cuchillo, Alborea, Albacete			39.19	-1.37	0
	SIVIM	U-P06650:Aegi	Observation	Aegilops triuncialis L.	L.	ES	Spain	Quinta da Figueirinha			37.13	-8.43	0
00:00.0	W	43792	Unknown	Aegilops triuncialis L.		ESP	Spain	Calatayud (In arenosis et incultis ad vias et agrorum marines)					
00:00.0	MA	722793-1	Specimen	Aegilops triuncialis L.	L.	ES	Spain	San Lorenzo de Calatrava, umbrÃ-a		CR	38	-3	
00:00.0	ABH	4010-1	Specimen	Aegilops triuncialis L.	L.	ES	Spain	Confrides; SÂª Aitana, prop Font de		A	38.66	-0.29	
	IDBD-GN	42519	Observation	Aegilops triuncialis L.	L.	ES	Spain	RÃ-o AragÃ³n	SangÃ¼esa	Na	42.5681	-1.27733	400
00:00.0	COFC	46904-1	Specimen	Aegilops triuncialis L.	L.	ES	Spain	ctra al pantano del Retortillo, CO-14		Co			1
	FUND. BIODIVER	1000498	Unknown	Aegilops triuncialis		ESP	Spain	Venta de Cabrejas		Cu	39.1	-2.1	
	FUND. BIODIVER	1027218	Unknown	Aegilops triuncialis L.	L.	ESP	Spain	Marmolejo		J	38.1	-4.1	650
00:00.0	FUND. BIODIVER	1774965	Unknown	Aegilops triuncialis L.	L.	ESP	Spain	Monzon, El Regal, Torre Carrasque		Hu	41.1	0.1	310
00:00.0	REDIAM-CMA	291673	Observation	Aegilops triuncialis		ESP	Spain		Cortes de Baza	Gr	37.751	-2.84779	901
	SIVIM	P-P09051:Aegi	Observation	Aegilops triuncialis L.	L.	ES	Spain	Barranc de les Canals; Arnes			40.79	0.15	0
	SIVIM	P-P09119:Aegi	Observation	Aegilops triuncialis L.	L.	ES	Spain	Serra de les Albardes; CoratxÃ			40.61	0.04	1180
	IPK	32378	Living	Aegilops triuncialis L. subsp. triuncialis var. triu		ESP	Spain	Stra?e Piedralaves - S. Martin de Viera, Avila					
00:00.0	SALA	18873-1	Specimen	Aegilops triuncialis L.	L.	ES	Spain	_; Almenara de Tormes		Sa			
00:00.0	GDA	GDA18850-1-1	Specimen	Aegilops triuncialis L.	L.	ES	Spain	Toledo, Calzada de Oropesa, Cerro		TO			0
00:00.0	MA	636844-1	Specimen	Aegilops triuncialis L.	L.	ES	Spain	Santiuste de Pedraza, Requijada, a		Sg	41	-3	
00:00.0	BC	646875	Specimen	Aegilops triuncialis L.	L.	ES	Spain	MatarÃ³; MatarÃ³, a Vistalegre		B	41.6	2.46	
	W	56432	Unknown	Aegilops triuncialis L.		ESP	Spain	[#####] en los Clanos de paller Ã¡ 3 kilometres Sur de La Carolina, ####. Terre					

	FUND. BIODIVER	1002570	Unknown	Aegilops triuncialis		ESP	Spain	Bosque de Alpera		Ab	38.1	-1.1	
	FUND. BIODIVER	1025030	Unknown	Aegilops triuncialis		ESP	Spain	Alcaudete		J	37.1	-3.1	
00:00.0	REDIAM-CMA	377836	Observation	Aegilops triuncialis		ESP	Spain		Hinojosa del Duq	Co	38.3867	-5.13172	589
	SIVIM	U-P09882:Aegi	Observation	Aegilops triuncialis L.	L.	ES	Spain	Cuneta cercana a 'El Sabinar'			38.12	-2.2	0
00:00.0	GDA	GDA16061-1-2	Specimen	Aegilops triuncialis L.	L.	ES	Spain	Granada, SÂª de Madrid, antes del		GR			1200
	IPK	69300	Living	Aegilops triuncialis L.		ESP	Spain	Puerto Real (Cadiz)					
	BC	70863	Specimen	Aegilops triuncialis L.	L.	ES	Spain	San CristÃ³bal		Te			
00:00.0	BC	92746	Specimen	Aegilops triuncialis L.	L.	ES	Spain	Dos Hermanas; Dos Hermanas Sev		Se	37.33	-5.88	
00:00.0	MA	651304-1	Specimen	Aegilops triuncialis L.	L.	ES	Spain	BaÃ±os de la Encina, Charca de la		J	38	-3	
00:00.0	SEV	98917-1	Specimen	Aegilops triuncialis L.	L.	ES	Spain	AznalcÃ¡zar		Se			1
00:00.0	SEV	98942-1	Specimen	Aegilops triuncialis L.	L.	ES	Spain	Sierra de Baza, entrando a Santa B		Gr			1500
	RUS001	VIR100602139	Specimen	Aegilops triuncialis L.		ESP	Spain						
00:00.0	REDIAM-CMA	395592	Observation	Aegilops triuncialis		ESP	Spain		CardeÃ±a	Co	38.2141	-4.30552	737
	SIVIM	R-P09664:Aegi	Observation	Aegilops triuncialis L.	L.	ES	Spain	Aleas			40.83	-3.23	0
	SIVIM	S-P01240:Aegi	Observation	Aegilops triuncialis L.	L.	ES	Spain	l'Albi			41.35	0.84	0
	SIVIM	S-P03955:Aegi	Observation	Aegilops triuncialis L.	L.	ES	Spain	l'Albi			41.35	0.84	0
	GDA	GDA42723-1-2	Specimen	Aegilops triuncialis L.	L.	ES	Spain	Granada, Deifontes, base de la Sier		GR			800
00:00.0	MGC	21971-1	Unknown	Aegilops triuncialis L.	L.	ES	Spain	San NicolÃ¡s del Puerto; Cerro del H		Se	0	0	670
	FUND. BIODIVER	70819	Unknown	Aegilops triuncialis L.	L.	ESP	Spain	Santiuste de Pedraza		Sg	40.1	-3.1	
00:00.0	REDIAM-CMA	78902	Observation	Aegilops triuncialis		ESP	Spain		Santiago-Pontone	J	38.1163	-2.67859	1400
00:00.0	REDIAM-CMA	84381	Observation	Aegilops triuncialis		ESP	Spain		Santiago-Pontone	J	38.0662	-2.59994	1409
00:00.0	REDIAM-CMA	108596	Observation	Aegilops triuncialis		ESP	Spain		Cabra	Co	37.5017	-4.36981	1000
00:00.0	REDIAM-CMA	124641	Observation	Aegilops triuncialis		ESP	Spain		Villacarrillo	J	38.0761	-3.01345	532
	SIVIM	T-P10777:Aegi	Observation	Aegilops triuncialis L.	L.	ES	Spain	Pozuelo de TÃ¡bara			41.78	-6	0
	SIVIM	T-P11864:Aegi	Observation	Aegilops triuncialis L.	L.	ES	Spain	Castrofuerte			42.15	-5.54	0
	SIVIM	T-P11865:Aegi	Observation	Aegilops triuncialis L.	L.	ES	Spain	Bracas			43.14	-5.58	0
00:00.0	BC	70819	Specimen	Aegilops triuncialis L.	L.	ES	Spain	Fonollosa; Fals (Bages)		B	41.77	1.74	
	W	42815	Unknown	Aegilops triuncialis L.		ESP	Spain	Catalogne: Barcelone, pentes du Tibidabo.					
00:00.0	GDA	GDA13281-1-2	Specimen	Aegilops triuncialis L.	L.	ES	Spain	MÃ¡laga, CoÃ-n, La AlbuquerÃ-a.		MA			0
00:00.0	BC	601434	Specimen	Aegilops triuncialis L.	L.	ES	Spain	Vilaplana; Muntanyes de Prades: Ve		T	41.22	1.03	365
00:00.0	BC	601438	Specimen	Aegilops triuncialis L.	L.	ES	Spain	Prades; Muntanyes de Prades: Plan		T	41.31	1.03	950
	RUS001	VIR100602169	Specimen	Aegilops triuncialis L.		ESP	Spain						
	FUND. BIODIVER	1043780	Unknown	Aegilops triuncialis L.	L.	ESP	Spain	San Pedro de AlcÃ¡ntara		Ma	36.1	-4.1	
	FUND. BIODIVER	1835131	Unknown	Aegilops triuncialis L.	L.	ESP	Spain	Provincia de Ciudad Real		CR			
00:00.0	REDIAM-CMA	389733	Observation	Aegilops triuncialis		ESP	Spain		Ronda	Ma	36.6927	-5.06103	1299
	SIVIM	Q-P03602:Aegi	Observation	Aegilops triuncialis L.	L.	ES	Spain	Villahermosa			40.17	-0.41	0
00:00.0	FCO	25921-1	Specimen	Aegilops triuncialis L.	L.	ES	Spain	Campos de Ledesma		Sa			
	FUND. BIODIVER	992691	Unknown	Aegilops triuncialis L.	L.	ESP	Spain	Mazarete		Gu	40.1	-2.1	
	FUND. BIODIVER	998073	Unknown	Aegilops triuncialis		ESP	Spain	Puerto de Cabrejas a Cuenca		Cu	39.1	-2.1	
00:00.0	REDIAM-CMA	202182	Observation	Aegilops triuncialis		ESP	Spain		Villaviciosa de CÃ	Co	37.98	-5.16753	302
00:00.0	REDIAM-CMA	228678	Observation	Aegilops triuncialis		ESP	Spain		Felix	Al	36.9051	-2.72226	1399

00:00.0	REDIAM-CMA	233870	Observation	Aegilops triuncialis		ESP	Spain		Espiel	Co	38.1588	-4.9069	700
00:00.0	REDIAM-CMA	237817	Observation	Aegilops triuncialis		ESP	Spain		AlmadÃ©n de la F	Se	37.8728	-5.94915	200
00:00.0	COFC	52521-1	Specimen	Aegilops triuncialis L.	L.	ES	Spain	Robledillo de la Vera; garganta de C		Cc	40	-5	1
	SIVIM	T-P18895:Aegi	Observation	Aegilops triuncialis L.	L.	ES	Spain	AlcalÃ¡ de Henares, finca La Clota			40.46	-3.47	0
00:00.0	MGC	62202-1	Unknown	Aegilops triuncialis L.	L.	ES	Spain	Lora del RÃo; Al Norte de Lora del		Se	37.681	-5.568	120
	FUND. BIODIVER	1025031	Unknown	Aegilops triuncialis		ESP	Spain	E. Martos y Alcaudete		J	37.1	-3.1	
00:00.0	REDIAM-CMA	363908	Observation	Aegilops triuncialis		ESP	Spain		LubrÃn	Al	37.1957	-2.0313	600
00:00.0	REDIAM-CMA	383104	Observation	Aegilops triuncialis		ESP	Spain				37.7445	-3.2906	
	SIVIM	P-P08710:Aegi	Observation	Aegilops triuncialis L.	L.	ES	Spain	SolÃ de les Codolles; Beseit			40.79	0.15	0
	SIVIM	U-P09883:Aegi	Observation	Aegilops triuncialis L.	L.	ES	Spain	Borde de cultivo de cebada en Ossa de Mo			38.93	-2.76	0
00:00.0	GDA	GDA18850-1-2	Specimen	Aegilops triuncialis L.	L.	ES	Spain	Toledo, Calzada de Oropesa, Cerro		TO			0
	ESP004	NC043492	Specimen	Aegilops triuncialis L.		ESP	Spain	Babilafuente, province of Salamanca			40.9667	-5.41667	801
	FUND. BIODIVER	1369177	Unknown	Aegilops triuncialis L.	L.	ESP	Spain	Osma		So	41.1	-2.1	
	FUND. BIODIVER	89108	Unknown	Aegilops triuncialis L.	L.	ESP	Spain	El Tiemblo, Valle de Iruelas, subida		Av	40.1	-4.1	1500
00:00.0	REDIAM-CMA	95180	Observation	Aegilops triuncialis		ESP	Spain		Cabra	Co	37.4921	-4.37281	1000
	SIVIM	T-P09353:Aegi	Observation	Aegilops triuncialis L.	L.	ES	Spain	PontÃ³n de la Oliva			40.83	-3.47	0
	SIVIM	T-P11404:Aegi	Observation	Aegilops triuncialis L.	L.	ES	Spain	Pto. de Cabrejas a Cuenca			40.01	-2.29	1000
	SIVIM	T-P15741:Aegi	Observation	Aegilops triuncialis L.	L.	ES	Spain	Sierra de Aguas			36.85	-4.79	0
00:00.0	UNEX	26866-1	Observation	Aegilops triuncialis L.	_	ESP	Spain	Magacela: Ermita de Los Remedios		Ba	38.8	-5.8	
00:00.0	UNEX	30099-1	Observation	Aegilops triuncialis L.	_	ESP	Spain	Magacela: Vallicar sobre suelos silÃ		Ba	38.8	-5.8	
	FUND. BIODIVER	1696531	Unknown	Aegilops ventricosa Tausch.	Tausch.	ESP	Spain	Cantavieja, mas de Porcar		Te	40.1	-0.1	1500
	FUND. BIODIVER	1700457	Unknown	Aegilops ventricosa		ESP	Spain	Cantavieja, Masia de Porcar		Te	40.1	-0.1	1400
	RUS001	VIR100602059	Specimen	Aegilops ventricosa Tausch		ESP	Spain						
00:00.0	BC	830716	Specimen	Aegilops ventricosa Tausch	Tausch	ES	Spain	Palma de Mallorca; A sota de la Vic		PM	39.16	2.94	
00:00.0	SALA	33263-1	Specimen	Aegilops ventricosa Tausch	Tausch	ES	Spain	_; Vallirana, Baix Llobregat		B			
1891-06-1	W	43622	Unknown	Aegilops ventricosa Tausch		ESP	Spain	Albacete, in aggeribus viarum ad Alcaraz. sol. caliar. 5-600 m.s.r					500
	FUND. BIODIVER	931082	Unknown	Aegilops ventricosa Tausch	Tausch	ESP	Spain	Port Beseit		T	40.1	0.1	
00:00.0	BC	485756	Specimen	Aegilops ventricosa Tausch	Tausch	ES	Spain	Esporlas; Menorca: Ferreries, pr. Sc		PM	39.97	4	
	NORDGEN	NGB90331	Living	Aegilops ventricosa		ESP	Spain	Spain: Mallorca, Baleares, Bellver			39.0833	3	
	SWE054	NGB90331	Specimen	Aegilops ventricosa TAUSCH		ESP	Spain	Spain: Mallorca, Baleares, Bellver			39.0833	3	
00:00.0	FUND. BIODIVER	1580842	Unknown	Aegilops ventricosa		ESP	Spain	Guardo		P	42.1	-4.1	1120
	MA	630039-1	Specimen	Aegilops venticosa Taush	Taush	ES	Spain	Cillorigo de LiÃ©bana, el Allende de		S	43	-4	
	SIVIM	T-P10780:Aegi	Observation	Aegilops ventricosa Tausch	Tausch	ES	Spain	Moreruela de TÃ¡bara			41.78	-5.88	0
	FUND. BIODIVER	99835	Unknown	Aegilops ventricosa Tausch	Tausch	ESP	Spain	Moreruela de TÃ¡bara		Za	41.1	-5.1	
	SIVIM	T-P25269:Aegi	Observation	Aegilops ventricosa Tausch	Tausch	ES	Spain	carretera a Cap Salines, marge d'una cune			39.29	3	0
00:00.0	SALA	48371-1	Specimen	Aegilops ventricosa Tausch	Tausch	ES	Spain	_; Castellanos de Villiquera		Sa			
00:00.0	BC	646575	Specimen	Aegilops ventricosa Tausch	Tausch	ES	Spain	Roquetes; Casetes Velles PORT DE		T	40.76	0.33	
	IPK	32135	Living	Aegilops ventricosa Tausch		ESP	Spain	La Alcarria, Spanien					
	FUND. BIODIVER	1643505	Unknown	Aegilops ventricosa Tausch	Tausch	ESP	Spain	El Castellar, Val de Zaragoza		Z	41.1	-0.1	640
	FUND. BIODIVER	1677768	Unknown	Aegilops ventricosa Tausch.	Tausch.	ESP	Spain	Archipielago de Cabrera, isla de Ca		PM	38.1	2.1	
	GDA	GDA43470-1-2	Specimen	Aegilops ventricosa Tausch.	Tausch.	ES	Spain	Granada, Guadix, Rambla Becerra.		GR			950

	ADIMAN	41	Observation	Aegilops ventricosa		ESP	Spain	EnguÃ-danos		CU	39.6715	-1.63478	
	GDA	GDA43470-1	Specimen	Aegilops ventricosa Tausch.	Tausch.	ES	Spain	Granada, Guadix, Rambla Becerra.		GR			950
00:00.0	BC	70864	Specimen	Aegilops ventricosa Tausch	Tausch	ES	Spain	Esporlas; Ferrerias, Menorca		PM	39.97	4	
00:00.0	FUND. BIODIVER	1369211	Unknown	Aegilops ventricosa Tausch.	Tausch.	ESP	Spain	Tejado, hacia Paridera Carralmonte		So	41.1	-2.1	1010
00:00.0	MA	636842-1	Specimen	Aegilops ventricosa		ES	Spain	Gallegos, 'Reollo', alrededores de a		Sg	41	-3	
00:00.0	REDIAM-CMA	384592	Observation	Aegilops ventricosa		ESP	Spain				37.6352	-3.15605	
00:00.0	SALA	40755-1	Specimen	Aegilops ventricosa Tausch	Tausch	ES	Spain	_; Santo TomÃ© del Puerto, Siguer		Sg			
00:00.0	BC	109565	Specimen	Aegilops ventricosa Tausch	Tausch	ES	Spain	Palma de Mallorca; A sota de sa Vi		PM	39.16	2.94	
	CZE122	01C2100523	Specimen	Aegilops ventricosa TAUSCH		ESP	Spain	La Alcarria, Spanien					
00:00.0	BDBCV	123	Observation	Aegilops ventricosa		ESP	Spain	Parque Natural de Penyagolosa		Cs			
00:00.0	BC	868565	Specimen	Aegilops ventricosa Tausch	Tausch	ES	Spain	Palma de Mallorca; Garrigues de so		PM	39.16	2.94	
00:00.0	SALA	85594-1	Specimen	Aegilops ventricosa Tausch	Tausch	ES	Spain	_; Castellanos de Villiquera		Sa	41.06	-5.64	
	ESP004	NC024038	Specimen	Aegilops ventricosa TAUSCH		ESP	Spain	Darro, province of Granada			37.35	-3.28333	1120
00:00.0	BDBCV	122	Observation	Aegilops ventricosa		ESP	Spain	Parque Natural de Penyagolosa		Cs	0	0	
00:00.0	SALA	122457-1	Specimen	Aegilops ventricosa Tausch	Tausch	ES	Spain	Pedrosillo el Ralo, Borde de prado c		Sa			
00:00.0	GDA	GDA18053-1	Specimen	Aegilops ventricosa Tausch.	Tausch.	ES	Spain	Granada, SÂª Nevada, Monachil, ba		GR			1600
	IPK	AE 653	Living	Aegilops ventricosa Tausch			Spain	La Alcarria, Spanien			40.5164	-2.25	
00:00.0	ABH	14362-1	Specimen	Aegilops ventricosa Tausch	Tausch	ES	Spain	Torremocha del Pinar; pr. Cerro Go		Gu	40.88	-2.04	
	SIVIM	T-P13702:Aegi	Observation	Aegilops ventricosa Tausch	Tausch	ES	Spain	Cantavieja			40.44	-0.52	1400
	FUND. BIODIVER	118438	Unknown	Aegilops ventricosa Tausch	Tausch	ESP	Spain	La Pola de GordÃ³n, Santa LucÃ-a		Le	42.1	-5.1	
	FUND. BIODIVER	1189437	Unknown	Aegilops ventricosa Tausch	Tausch	ESP	Spain	Santa Ponsa en Alaior		PM	39.1	3.1	
00:00.0	FCO	7227-1	Specimen	Aegilops ventricosa Tausch.	Tausch.	ES	Spain	Cervera del Pisuerga		P			
00:00.0	SALA	10060-1	Specimen	Aegilops ventricosa Tausch	Tausch	ES	Spain	_; La Fuente de San Esteban, MuÃ		Sa			
00:00.0	FUND. BIODIVER	1369207	Unknown	Aegilops ventricosa Tausch.	Tausch.	ESP	Spain	Renieblas		So	41.1	-2.1	1050
00:00.0	FUND. BIODIVER	1473161	Unknown	Aegilops ventricosa Tausch	Tausch	ESP	Spain	Sotresgudo, CaserÃ-o de Monte Re		Bu	42.1	-4.1	910
	GDA	GDA43470-1-3	Specimen	Aegilops ventricosa Tausch.	Tausch.	ES	Spain	Granada, Guadix, Rambla Becerra.		GR			950
00:00.0	BC	103568	Specimen	Aegilops ventricosa Tausch	Tausch	ES	Spain	Palma de Mallorca; a sota sa Vicari		PM	39.16	2.94	
	IPK	AE 1054	Living	Aegilops ventricosa Tausch			Spain	Bellver (Baleares)			39.5625	2.62	
00:00.0	FCO	7227-1	Specimen	Aegilops ventricosa Tausch.	Tausch.	ES	Spain	Cervera del Pisuerga		P			
00:00.0	SEV	8649-1	Specimen	Aegilops ventricosa Tausch	Tausch	ES	Spain	Entre Villaquejida y Villamandos		Le			1
	DEU146	AE 653	Specimen	Aegilops ventricosa TAUSCH		ESP	Spain	La Alcarria, Spanien					
	BDBCV-General	88471	Observation	Aegilops ventricosa		ESPA	Spain	Ares del Maestre	L'Alt Maestrat	Castel	40.3889	-0.11368	
	GDA	GDA43471-1-1	Specimen	Aegilops ventricosa Tausch.	Tausch.	ES	Spain	Granada, Guadix, Rambla Becerra.		GR			980
	W	43548	Unknown	Aegilops ventricosa Tausch		ESP	Spain	Sierra de Cabrera del Cuarto in Spanien auf Roggenfeldern.					
00:00.0	FUND. BIODIVER	1946468	Unknown	Aegilops ventricosa Tausch	Tausch	ESP	Spain	Villahermosa, Santa Maria		CR	38.1	-2.1	760
	FUND. BIODIVER	59597	Unknown	Aegilops ventricosa Tausch	Tausch	ESP	Spain	Monterrubio de ArmuÃ±a		Sa	40.1	-5.1	
00:00.0	GDA	GDA30031-1	Specimen	Aegilops ventricosa Tausch.	Tausch.	ES	Spain	Mallorca, Bellver.		PM			0
00:00.0	BC	103567	Specimen	Aegilops ventricosa Tausch	Tausch	ES	Spain	Palma de Mallorca; a sota sa Vicari		PM	39.16	2.94	
	GDA	GDA30030-1-2	Specimen	Aegilops ventricosa Tausch.	Tausch.	ES	Spain	Granada, SÂª Nevada.		GR			0
	FUND. BIODIVER	1043782	Unknown	Aegilops ventricosa Tausch	Tausch	ESP	Spain	Sierra de las Nieves		Ma	36.1	-4.1	
00:00.0	BC	92757	Specimen	Aegilops ventricosa Tausch	Tausch	ES	Spain	Bacares; De Becares a la Venta de		Al	37.27	-2.49	

	SIVIM	T-P13665:Aegi	Observation	Aegilops ventricosa Tausch	Tausch	ES	Spain	Mosqueruela			40.35	-0.52	1480
	W	43641	Unknown	Aegilops ventricosa Tausch		ESP	Spain	In arvis Sierra Nevada. Alt: 5000'					1524
	BC	92754	Specimen	Aegilops ventricosa Tausch	Tausch	ES	Spain	Almansa; Almansa		Ab	38.88	-1.1	
00:00.0	BC	830206	Specimen	Aegilops ventricosa Tausch	Tausch	ES	Spain	Garrigues de sota	PALMA	PM	39.1191	2.884322	
00:00.0	MA	643239-1	Specimen	Aegilops ventricosa Tausch	Tausch	ES	Spain	Juseu		Hu	42	0	
	IPK	32169	Living	Aegilops ventricosa Tausch var. comosa (Coss		ESP	Spain	La Alcarria					
	ESP004	NC043502	Specimen	Aegilops ventricosa TAUSCH		ESP	Spain	Pedrosillo el Ralo, province of Salamanca			41.05	-5.53333	818
	FUND. BIODIVER	1131637	Unknown	Aegilops ventricosa Tausch	Tausch	ESP	Spain	Alar del Rey		P	42.1	-4.1	
	FUND. BIODIVER	56044	Unknown	Aegilops ventricosa Tausch.	Tausch.	ESP	Spain	Encinas de Esgueva, Fuenteblanca		Va	41.1	-3.1	
	SIVIM	T-P01738:Aegi	Observation	Aegilops ventricosa Tausch	Tausch	ES	Spain	Mallorca: Lluc, prop de Son Llovera (fons d			39.74	2.76	0
	FUND. BIODIVER	1648490	Unknown	Aegilops ventricosa Tausch.	Tausch.	ESP	Spain	Puertomingalvo, entre Mosqueruela		Te	40.1	-0.1	
00:00.0	SEV	108391-1	Specimen	Aegilops ventricosa Tausch	Tausch	ES	Spain	SerranÃ-a de Ronda. Sierra de las I		Ma			900
00:00.0	MA	643238-1	Specimen	Aegilops ventricosa Tausch	Tausch	ES	Spain	Roda de IsÃ¡bena		Hu	42	0	
00:00.0	HUAL	1140-1	Specimen	Aegilops ventricosa Tansch	Tansch	ES	Spain	SÂª del Pozo, prÃ³x. Nava de San P		J	37.889	-2.881	
00:00.0	GDA	GDA17351-1	Specimen	Aegilops ventricosa Tausch.	Tausch.	ES	Spain	Granada, SÂª Parapanda, prÃ³ximo		GR			1100
	FUND. BIODIVER	998075	Unknown	Aegilops ventricosa		ESP	Spain	Sierra de San Felipe		Cu	40.1	-1.1	
1894-06-1	W	43607	Unknown	Aegilops ventricosa Tausch		ESP	Spain	Espagne, province de Granada, Puebla de Don Fadrique					
00:00.0	COFC	52522-1	Specimen	Aegilops ventricosa Tausch	Tausch	ES	Spain	Ribera del Fresno; _		Ba	38	-6	1
00:00.0	GDA	GDA17351-1-1	Specimen	Aegilops ventricosa Tausch.	Tausch.	ES	Spain	Granada, SÂª Parapanda, prÃ³ximo		GR			1100
	FUND. BIODIVER	62023	Unknown	Aegilops ventricosa Tausch	Tausch	ESP	Spain	La Fuente de San Esteban, Boadilla		Sa	40.1	-6.1	
00:00.0	SALA	124639-1	Specimen	Aegilops ventricosa Tausch	Tausch	ES	Spain	Calvia, Ball Negre		Mll			
	FUND. BIODIVER	1106550	Unknown	Aegilops ventricosa Tausch	Tausch	ESP	Spain	Molino de la Villa		Sa	40.1	-5.1	760
	RUS001	VIR100602307	Specimen	Aegilops ventricosa Tausch		ESP	Spain						
	IPK	31850	Living	Aegilops ventricosa Tausch var. ventricosa		ESP	Spain	Lluchmajor, Majorque, Baleares					
00:00.0	FUND. BIODIVER	1897919	Unknown	Aegilops ventricosa Tausch	Tausch	ESP	Spain	Junta de Traslaloma, Villalacre		Bu	42.1	-3.1	720
	IDBD-GN	42521	Observation	Aegilops ventricosa Tausch	Tausch	ES	Spain		Valle de Lana	Na	42.7629	-2.20689	
00:00.0	GDAC	GDAC15898-1	Specimen	Aegilops ventricosa Tausch	Tausch	ES	Spain	CÃ¡ceres, Minas de Aldeamoret.		CC			0
	FUND. BIODIVER	1106551	Unknown	Aegilops ventricosa Tausch	Tausch	ESP	Spain	Cantalapiedra		Sa	40.1	-5.1	760
	GDA	GDA43471-1	Specimen	Aegilops ventricosa Tausch.	Tausch.	ES	Spain	Granada, Guadix, Rambla Becerra.		GR			980
	SANT	2114	Specimen	Aegilops ventricosa Tausch.		ES	Spain	Mallorca, Bellver		PM			
	IDBD-GN	42522	Observation	Aegilops ventricosa Tausch	Tausch	ES	Spain	Gastiain	Lana	Na	42.6736	-2.33007	
	IPK	32100	Living	Aegilops ventricosa Tausch		ESP	Spain	La Alcarria, Spanien					
00:00.0	FUND. BIODIVER	1774966	Unknown	Aegilops ventricosa Tausch	Tausch	ESP	Spain	Monzon, pie del cerro del castillo		Hu	41.1	-0.9	300
	SIVIM	T-P13691:Aegi	Observation	Aegilops ventricosa Tausch	Tausch	ES	Spain	MasÃ-a de la Tosquilla, Iglesuela del Cid			40.44	-0.4	1300
00:00.0	GDA	GDA18053-1-1	Specimen	Aegilops ventricosa Tausch.	Tausch.	ES	Spain	Granada, SÂª Nevada, Monachil, ba		GR			1600
	ESP004	NC043499	Specimen	Aegilops ventricosa TAUSCH		ESP	Spain	Palma de Mallorca, province of Baleares			39.5667	2.65	15
00:00.0	MA	618657-1	Specimen	Aegilops ventricosa Tausch.	Tausch.	ES	Spain	Randa, carretera de Randa al Santu		Mll	39	2	
00:00.0	BC	830717	Specimen	Aegilops ventricosa Tausch	Tausch	ES	Spain	Palma de Mallorca; A sota de sa Vi		PM	39.16	2.94	
00:00.0	GDA	GDA48364-1-3	Specimen	Aegilops ventricosa Tausch.	Tausch.	ES	Spain	Guadalajara, ArbancÃ³n, Barranco d		GU			950
	FUND. BIODIVER	1135165	Unknown	Aegilops ventricosa Tausch	Tausch	ESP	Spain	Santa LucÃ-a de GordÃ³n		Le	42.1	-5.1	
	FUND. BIODIVER	117643	Unknown	Aegilops ventricosa Tausch	Tausch	ESP	Spain	Burgos		Bu	42.1	-3.1	

	FUND. BIODIVER	1374649	Unknown	Aegilops ventricosa Tausch	Tausch	ESP	Spain	OntiÃ±ena		Hu	41.1	0.9	230
00:00.0	FUND. BIODIVER	1469278	Unknown	Aegilops ventricosa Tausch	Tausch	ESP	Spain	Beteta, valle del Tajo, Hoyo Redon		Cu	40.1	-1.1	1350
00:00.0	UNEX	08472-1	Observation	Aegilops ventricosa Tausch	_	ESP	Spain	Ribera del Fresno: Suelos bÃ¡sicos		Ba	38.5	-6.2	
	ESP004	NC050477	Specimen	Aegilops ventricosa TAUSCH		ESP	Spain	Valdemorillo Sierra, province of Cuenca			40.0333	-1.76667	1211
00:00.0	BC	112848	Specimen	Aegilops ventricosa Tausch	Tausch	ES	Spain	Palma de Mallorca; Bellver. Mallorc		PM	39.52	2.59	
	FUND. BIODIVER	1135166	Unknown	Aegilops ventricosa Tausch	Tausch	ESP	Spain	Bercianos del Real Camino		Le	42.1	-4.1	
00:00.0	SALA	64487-1	Specimen	Aegilops ventricosa Tausch	Tausch	ES	Spain	_; CÃ¡ceres, Aldea Moret, Minas Al		Cc			
	IPK	AE 617	Living	Aegilops ventricosa Tausch			Spain	La Alcarria, Spanien			40.5164	-2.25	
00:00.0	SALA	40756-1	Specimen	Aegilops ventricosa Tausch	Tausch	ES	Spain	_; SepÃºlveda, Rabo de la Lastra		Sg			
00:00.0	FUND. BIODIVER	1369205	Unknown	Aegilops ventricosa Tausch.	Tausch.	ESP	Spain	Hinojosa de la Sierra, hacia Langos		So	41.1	-2.1	1100
00:00.0	FUND. BIODIVER	1473160	Unknown	Aegilops ventricosa Tausch	Tausch	ESP	Spain	Merindad de Sotoscueva, Cornejo		Bu	42.1	-3.1	640
	FUND. BIODIVER	1684424	Unknown	Aegilops ventricosa Tausch.	Tausch.	ESP	Spain	Cerro de San Cristobal sobre Graza		Ca	36.1	-5.1	
	FUND. BIODIVER	1700272	Unknown	Aegilops ventricosa		ESP	Spain	Iglesuela del Cid, Masia de la Tosq		Te	40.1	-0.1	1300
00:00.0	GDA	GDA17351-1-2	Specimen	Aegilops ventricosa Tausch.	Tausch.	ES	Spain	Granada, SÂª Parapanda, prÃ³ximo		GR			1100
00:00.0	HUAL	5972-1	Specimen	Aegilops ventricosa Tansch	Tansch	ES	Spain	SÂª de GÃ¡dor, El Calabrial		Al	36.906	-2.725	
	GDA	GDA43470-1-1	Specimen	Aegilops ventricosa Tausch.	Tausch.	ES	Spain	Granada, Guadix, Rambla Becerra.		GR			950
	SIVIM	Q-P07407:Aegi	Observation	Aegilops ventricosa Tausch	Tausch	ES	Spain	Finca Navodres			39.65	-2.06	0
00:00.0	BC	867056	Specimen	Aegilops ventricosa Tausch	Tausch	ES	Spain	AlcalÃ¡ de la Selva; AlcalÃ de la S.		Te	40.3591	-0.76228	
	FUND. BIODIVER	1189440	Unknown	Aegilops ventricosa Tausch.	Tausch.	ESP	Spain	RambÃ s		PM	39.1	3.1	
	FUND. BIODIVER	70816	Unknown	Aegilops ventricosa Tausch	Tausch	ESP	Spain	Santiuste de Pedraza		Sg	40.1	-3.1	
	MGC	37002-1	Specimen	Aegilops ventricosa Tausch	Tausch	ES	Spain	Antequera; Torcal de Antequera		Ma	36.99	-4.52	1175
	SIVIM	T-P11410:Aegi	Observation	Aegilops ventricosa Tausch	Tausch	ES	Spain	Sierra de San Felipe			40.37	-1.93	1370
	DEU146	AE 954	Specimen	Aegilops ventricosa TAUSCH		ESP	Spain	Mallorca, Balearen					
	SIVIM	R-P11646:Aegi	Observation	Aegilops ventricosa Tausch	Tausch	ES	Spain	Tarifa			44.93	-6.04	0
00:00.0	GDA	GDA30031-1-2	Specimen	Aegilops ventricosa Tausch.	Tausch.	ES	Spain	Mallorca, Bellver.		PM			0
	DEU146	AE 357	Specimen	Aegilops ventricosa Tausch var. ventricosa (C		ESP	Spain	Lluchmajor, Majorque, Baleares					
	SIVIM	Q-P07462:Aegi	Observation	Aegilops ventricosa Tausch	Tausch	ES	Spain	Cervera del Llano			39.74	-2.53	0
	FUND. BIODIVER	70817	Unknown	Aegilops ventricosa Tausch	Tausch	ESP	Spain	Gallegos		Sg	40.1	-3.1	
	SIVIM	T-P10773:Aegi	Observation	Aegilops ventricosa Tausch	Tausch	ES	Spain	Dehesa de Misleo (Moreruela de TÃ¡bara)			41.78	-5.88	0
	ESP004	NC043505	Specimen	Aegilops ventricosa TAUSCH		ESP	Spain	Adrada de Piron, province of Segovia			41.05	-4.03333	1019
	FUND. BIODIVER	70818	Unknown	Aegilops ventricosa Tausch	Tausch	ESP	Spain	Pedraza		Sg	40.1	-3.1	
	FUND. BIODIVER	70874	Unknown	Aegilops ventricosa Tsch.	Tsch.	ESP	Spain	Gomecello		Sa	40.1	-5.1	
00:00.0	BC	866347	Specimen	Aegilops ventricosa Tausch	Tausch	ES	Spain	Valdelinares; Valdecerezo		Te	40.3567	-0.6446	
	FUND. BIODIVER	1385217	Unknown	Aegilops ventricosa Tausch	Tausch	ESP	Spain	Cabra		Co	37.1	-4.1	
	FUND. BIODIVER	96897	Unknown	Aegilops ventricosa Tausch	Tausch	ESP	Spain	Pereruela, San RomÃ¡n de los Infa		Za	41.1	-5.1	
00:00.0	GDA	GDA52403-1	Specimen	Aegilops ventricosa Tausch	Tausch	ES	Spain	AlmerÃ-a, FondÃ³n, cabecera del B		AL			1720
00:00.0	JBS	457-1	Specimen	Aegilops ventricosa Tausch	Tausch	ES	Spain	Cabrera (Palma); Cabrera		Mll	39.14	2.95	1
	GDA	GDA43471-1-3	Specimen	Aegilops ventricosa Tausch.	Tausch.	ES	Spain	Granada, Guadix, Rambla Becerra.		GR			980
00:00.0	BC	92756	Specimen	Aegilops ventricosa Tausch	Tausch	ES	Spain	CalviÃ ; CalviÃ (Mallorca) Ball Neg		PM	39.52	2.48	
	ESP004	NC050488	Specimen	Aegilops ventricosa TAUSCH		ESP	Spain	casa forestal de Tejadillos, Cuenca, provin			40.4	-1.98333	1080
	IPK	AE 954	Living	Aegilops ventricosa Tausch			Spain	Mallorca, Balearen			39.5	3	

	ESP004	NC027462	Specimen	Aegilops ventricosa TAUSCH		ESP	Spain	Ports de Beceite, Roquetes, province of Tarragona					
00:00.0	MA	636843-1	Specimen	Aegilops ventricosa Tausch.	Tausch.	ES	Spain	Santiuste de Pedraza, de Torre Val		Sg	41	-3	
00:00.0	REDIAM-CMA	87352	Observation	Aegilops ventricosa		ESP	Spain		Cambil	J	37.7288	-3.48885	1599
	ESP004	NC050481	Specimen	Aegilops ventricosa TAUSCH		ESP	Spain	Cuenca/Villalba de la Sierra 8km N, Cuenc			40.15	-2.13333	940
	FUND. BIODIVER	1115796	Unknown	Aegilops ventricosa Tausch	Tausch	ESP	Spain	Dehesa de Misleo		Za	41.1	-5.1	
	FUND. BIODIVER	115633	Unknown	Aegilops ventricosa Tausch	Tausch	ESP	Spain	Guardo, Guardo		P	42.1	-4.1	
	FUND. BIODIVER	99836	Unknown	Aegilops ventricosa Tausch	Tausch	ESP	Spain	Carbajales de Alba		Za	41.1	-5.1	
	FUND. BIODIVER	984113	Unknown	Aegilops ventricosa var. comosa	Cosson	ESP	Spain	Valle de Lana		Na	42.1	-2.1	
	SIVIM	T-P26238:Aegi	Observation	Aegilops ventricosa Tausch	Tausch	ES	Spain	RincÃ³n de los Huertos, Moratlla			38.12	-1.97	1300
00:00.0	SALA	18782-1	Specimen	Aegilops ventricosa Tausch	Tausch	ES	Spain	_; PelarrodrÃ-guez, Peramato		Sa			
00:00.0	BC	151093	Specimen	Aegilops ventricosa Tausch	Tausch	ES	Spain	Vistabella del Maestrazgo; St. Joan		Cs	40.22	-0.36	1275
	FUND. BIODIVER	1380640	Unknown	Aegilops ventricosa Tausch	Tausch	ESP	Spain	Moratalla, proximidades de La Roga		Mu	37.1	-2.1	1200
00:00.0	SEV	99223-1	Specimen	Aegilops ventricosa Tausch	Tausch	ES	Spain	Entre Ronda y Sierra de las Nieves.		Ma			1150
	FUND. BIODIVER	1374645	Unknown	Aegilops ventricosa Tausch	Tausch	ESP	Spain	Roda de IsÃ¡bena		Hu	42.1	0.1	820
	FUND. BIODIVER	1385294	Unknown	Aegilops ventricosa Tausch	Tausch	ESP	Spain	CaÃ±ada del RÃ-o PirÃ³n, Adrada d		Sg	40.1	-3.1	960
1879-07-0	W	43620	Unknown	Aegilops ventricosa Tausch		ESP	Spain	Regnum Granatense, Sierra da Alfaran, ad margine lotorum cult					1300
00:00.0	GDAC	GDAC37577-1	Specimen	Aegilops ventricosa Tausch	Tausch	ES	Spain	Granada, Parque Natural de la SÂª		GR			1700
	IDBD-GN	42523	Observation	Aegilops ventricosa Tausch	Tausch	ES	Spain	Gastiain	Lana	Na	42.6736	-2.33007	
00:00.0	JBS	458-1	Specimen	Aegilops ventricosa Tausch	Tausch	ES	Spain	SÃ³ller; Coll de Ses Puntes		Mll	39.81	2.7	1
00:00.0	MA	569241-1	Specimen	Aegilops ventricosa Tausch	Tausch	ES	Spain	Siguero, AldealapeÃ±a		Sg			
00:00.0	BC	109560	Specimen	Aegilops ventricosa Tausch	Tausch	ES	Spain	Palma de Mallorca; Al costat del Ca		PM	39.16	2.94	
00:00.0	SALA	30602-1	Specimen	Aegilops ventricosa Tausch	Tausch	ES	Spain	_; La BÃ³veda de Toro		Za			
00:00.0	COA	41232-1	Specimen	Aegilops ventricosa Tausch	Tausch	ES	Spain	JardÃ-n BotÃ¡nico de CÃ³rdoba, cul		Co	37.84	-4.82	
00:00.0	BC	70860	Specimen	Aegilops ventricosa Tausch	Tausch	ES	Spain	Mancha Real; Ad Cortijo de Los Pra		J	37.81	-3.62	1400
	CZE122	01C2100507	Specimen	Aegilops ventricosa var. ventricosa TAUSCH		ESP	Spain	Lluchmajor, Majorque, Baleares					
00:00.0	BC	605380	Specimen	Aegilops ventricosa Tausch	Tausch	ES	Spain	Escorca; Mallorca: Lluc, Son Macip		PM	39.8	2.94	
	GDA	GDA30030-1	Specimen	Aegilops ventricosa Tausch.	Tausch.	ES	Spain	Granada, SÂª Nevada.		GR			0
00:00.0	BC	92755	Specimen	Aegilops ventricosa Tausch	Tausch	ES	Spain	Mataporquera; Mataporquera Santa		S	42.85	-4.16	950
	IPK	AE 357	Living	Aegilops ventricosa Tausch var. ventricosa			Spain	Lluchmajor, Majorque, Baleares			39.4858	2.894722	
	ESP004	NC027422	Specimen	Aegilops ventricosa TAUSCH		ESP	Spain	Carrascosa del Campo, Campos del Parais			40.0333	-2.75	898
	FUND. BIODIVER	1189436	Unknown	Aegilops ventricosa Tausch	Tausch	ESP	Spain	Palafanguer		PM			
	GDA	GDA30030-1-1	Specimen	Aegilops ventricosa Tausch.	Tausch.	ES	Spain	Granada, SÂª Nevada.		GR			0
	ESP004	NC043501	Specimen	Aegilops ventricosa TAUSCH		ESP	Spain	Ciutatdella de Menorca, province of Balear			40	3.83333	17
00:00.0	BDBCV	124	Observation	Aegilops ventricosa		ESP	Spain	Parque Natural de Penyagolosa		Cs			
	DEU146	AE 689	Specimen	Aegilops ventricosa Tausch var. comosa (Coss		ESP	Spain	La Alcarria					
	MA	584625-1	Specimen	Aegilops ventricosa Tausch.	Tausch.	ES	Spain			Ca			
	FUND. BIODIVER	1699514	Unknown	Aegilops ventricosa		ESP	Spain	Mosqueruela		Te	40.1	-0.1	1480
00:00.0	SALA	66978-1	Specimen	Aegilops ventricosa Tausch	Tausch	ES	Spain	_; Llanos de Matamoros		Cc			
	IPK	32425	Living	Aegilops ventricosa Tausch		ESP	Spain	Mallorca, Balearen					
00:00.0	BC	70859	Specimen	Aegilops ventricosa Tausch	Tausch	ES	Spain	Marratxi; Pont d'Inca		PM	39.61	2.71	
	FUND. BIODIVER	1648489	Unknown	Aegilops ventricosa Tausch.	Tausch.	ESP	Spain	Iglesuela del Cid, Masico MarÃ-n		Te	40.1	-0.1	

00:00.0	BC	109289	Specimen	Aegilops ventricosa Tausch	Tausch	ES	Spain	Palma de Mallorca; Al costat del Ca		PM	39.16	2.94	
	RUS001	VIR100602058	Specimen	Aegilops ventricosa Tausch		ESP	Spain						
	SIVIM	U-P02914:Aegi	Observation	Aegilops ventricosa Tausch	Tausch	ES	Spain	Castells de Serrella			38.72	-0.23	0
00:00.0	GDAC	GDAC31052-1	Specimen	Aegilops ventricosa Tausch	Tausch	ES	Spain	JaÃ©n, Santiago de la Espada.		J			0
00:00.0	BC	605800	Specimen	Aegilops ventricosa Tausch	Tausch	ES	Spain	Campos del Puerto; Mallorca: S. Alc		PM	39.34	3.06	
00:00.0	SALA	83694-1	Specimen	Aegilops ventricosa Tausch	Tausch	ES	Spain	_; La Pola de GordÃ³n, Santa Lucia		Le			
00:00.0	Marimurtra	4475-1	Specimen	Aegilops ventricosa Tausch.	Tausch.	ES	Spain	Palma de Mallorca; Bellver		PM			
00:00.0	GDAC	GDAC26133-1	Specimen	Aegilops ventricosa Tausch	Tausch	ES	Spain	Granada, SÂª de Baza, Camino de		GR			1450
00:00.0	GDA	GDA22406-1-2	Specimen	Aegilops ventricosa Tausch.	Tausch.	ES	Spain	Granada, Cogollos Vega, prÃ³ximo		GR			1440
00:00.0	MA	730044-1	Specimen	Aegilops ventricosa Tausch	Tausch	ES	Spain	Castromonte		Va	41	-5	
00:00.0	ABH	49084-1	Specimen	Aegilops ventricosa Tausch	Tausch	ES	Spain	ArbancÃ³n; Barranco de la Hoz		Gu	0.41	-7.08	
	ESP004	NC050473	Specimen	Aegilops ventricosa TAUSCH		ESP	Spain	Cuenca/Carrascosa del Campo 5km W, Fu			40.0833	-2.21667	960
	ESP004	NC043503	Specimen	Aegilops ventricosa TAUSCH		ESP	Spain	Molino de la Villa, Cantalapiedra, province			41.15	-5.16667	785
	FUND. BIODIVER	1120229	Unknown	Aegilops ventricosa Tausch	Tausch	ESP	Spain	Abezames, Gafos		Za	41.1	-5.1	
1876-06-2	W	43523	Unknown	Aegilops ventricosa Tausch		ESP	Spain	Hispania: In valle fluv. Darro supra Granatam. In itinere hispanico-lusitanico.					
1879-07-0	W	43534	Unknown	Aegilops ventricosa Tausch		ESP	Spain	Regnum Granatense, Sierra de Alfaran. ad margines lotorum cu					1300
	FUND. BIODIVER	1370910	Unknown	Aegilops ventricosa Tausch	Tausch	ESP	Spain	RedueÃ±a		M	40.1	-3.1	
	FUND. BIODIVER	1696530	Unknown	Aegilops ventricosa Tausch.	Tausch.	ESP	Spain	Puertomingalvo, entre Mosqueruela		Te	40.1	-0.1	1500
00:00.0	COA	41170-1	Specimen	Aegilops ventricosa Tausch	Tausch	ES	Spain	Cabra, pr. final		Co	37.4	-4.47	
00:00.0	HSS	20954	Specimen	Aegilops ventricosa	Taush	ES	Spain	Villafranca de los Barros, Cerro de S		Ba	38.505	-6.42001	
	FUND. BIODIVER	1189438	Unknown	Aegilops ventricosa Tausch	Tausch	ESP	Spain	Camino de Santa Eulalia a Toro		PM	39.1	3.1	
00:00.0	SEV	97579-1	Specimen	Aegilops ventricosa Tausch	Tausch	ES	Spain	Minas de Aldeamoret		Cc			1
	FUND. BIODIVER	1374647	Unknown	Aegilops ventricosa Tausch	Tausch	ESP	Spain	Olvena		Hu	41.1	0.1	460
	FUND. BIODIVER	1648491	Unknown	Aegilops ventricosa Tausch.	Tausch.	ESP	Spain	Cantavieja, Mas de Porcar		Te	40.1	-0.1	
00:00.0	FUND. BIODIVER	1835151	Unknown	Aegilops ventricosa Tausch	Tausch	ESP	Spain	Puebla del Principe, ermita de la Ma		CR	38.1	-2.1	860
00:00.0	FUND. BIODIVER	1835152	Unknown	Aegilops ventricosa Tausch	Tausch	ESP	Spain	Villahermosa, Santa Maria		CR	38.1	-2.1	760
00:00.0	REDIAM-CMA	236254	Observation	Aegilops ventricosa		ESP	Spain		Castril	Gr	37.8533	-2.78051	1244
00:00.0	BC	622803	Specimen	Aegilops ventricosa Tausch	Tausch	ES	Spain	La Fuente de San Esteban; MuÃ±oz		Sa	40.84	-6.21	
00:00.0	BC	70862	Specimen	Aegilops ventricosa Tausch	Tausch	ES	Spain	Castromonte; Castille: Castromonte		Va	41.76	-4.98	
00:00.0	SEV	83896-1	Specimen	Aegilops ventricosa Tausch	Tausch	ES	Spain	Sierra Nevada. Fuente de Don Man		Gr			1750
	RUS001	VIR100602061	Specimen	Aegilops ventricosa Tausch		ESP	Spain						
	RUS001	VIR100602060	Specimen	Aegilops ventricosa Tausch		ESP	Spain						
	SIVIM	R-P08277:Aegi	Observation	Aegilops ventricosa Tausch	Tausch	ES	Spain	Coll dels Brucs (CastellolÃ-)			41.54	1.68	0
00:00.0	SALA	122458-1	Specimen	Aegilops ventricosa Tausch	Tausch	ES	Spain	Prado a la derecha de la carretera C		Sa			
	FUND. BIODIVER	1931161	Unknown	Aegilops ventricosa		ESP	Spain	Carretera a Cap Salines		PM	39.1	2.1	
00:00.0	HSS	13399	Specimen	Aegilops ventricosa Taush	Taush	ES	Spain	Zafra, Matanegra		Ba	38.4149	-6.42321	
00:00.0	FUND. BIODIVER	1369201	Unknown	Aegilops ventricosa Tausch.	Tausch.	ESP	Spain	Beltejar, hacia Blocona		So	41.1	-2.1	1150
	ESP004	NC050485	Specimen	Aegilops ventricosa TAUSCH		ESP	Spain	Las Majadas/Beteta 8km NE, Cuenca, prov			40.35	-2	1210
	FUND. BIODIVER	114935	Unknown	Aegilops ventricosa Tausch	Tausch	ESP	Spain	Guardo, Guardo		P	42.1	-4.1	
00:00.0	FUND. BIODIVER	1946467	Unknown	Aegilops ventricosa Tausch	Tausch	ESP	Spain	Puebla del Principe, ermita de la Ma		CR	38.1	-2.1	860
00:00.0	BC	830731	Specimen	Aegilops ventricosa Tausch	Tausch	ES	Spain	Palma de Mallorca; Casernes, al co		PM	39.16	2.94	

00:00.0	FUND. BIODIVER	1593457	Unknown	Aegilops ventricosa		ESP	Spain	MuÃ±eca		P	42.1	-4.1	1180
	GDA	GDA43471-1-2	Specimen	Aegilops ventricosa Tausch.	Tausch.	ES	Spain	Granada, Guadix, Rambla Becerra.		GR			980
00:00.0	SALA	30601-1	Specimen	Aegilops ventricosa Tausch	Tausch	ES	Spain	_; FuentelapeÃ±a		Za			
1879-07-0	GJO	GJO-0015464	Unknown	Aegilops ventricosa Tausch		Spain	Spain	Regnum Prenaterse, Sierra de la Alfacar, ad margines locarum c					100
	FUND. BIODIVER	58084	Unknown	Aegilops ventricosa Tausch	Tausch	ESP	Spain	Cantalapiedra		Sa	40.1	-5.1	
	FUND. BIODIVER	85049	Unknown	Aegilops ventricosa Tausch	Tausch	ESP	Spain	Sierra del Almuerzo		So	41.1	-2.1	
00:00.0	MA	623260-1	Specimen	Aegilops ventricosa		ES	Spain	Torices, CabezÃ³n de LiÃ©bana		S	43	-4	
00:00.0	GDA	GDA30031-1-1	Specimen	Aegilops ventricosa Tausch.	Tausch.	ES	Spain	Mallorca, Bellver.		PM			0
00:00.0	MA	715545-1	Specimen	Aegilops ventricosa Tausch	Tausch	ES	Spain	Sierra de Villafuerte, fuente del Can		Mu	38.1412	-2.1598	
	ESP004	NC027450	Specimen	Aegilops ventricosa TAUSCH		ESP	Spain	Turmiel, Maranchon, province of Guadalaja			41.0167	-2.06667	1122
	FUND. BIODIVER	76090	Unknown	Aegilops ventricosa Tausch	Tausch	ESP	Spain	FuentelapeÃ±a		Za	41.1	-5.1	
	FUND. BIODIVER	76091	Unknown	Aegilops ventricosa Tausch	Tausch	ESP	Spain	La BÃ³veda de Toro		Za	41.1	-5.1	
	SIVIM	T-P13686:Aegi	Observation	Aegilops ventricosa Tausch	Tausch	ES	Spain	Cantavieja			40.44	-0.52	1340
00:00.0	FUND. BIODIVER	1369209	Unknown	Aegilops ventricosa Tausch.	Tausch.	ESP	Spain	La Rubia		So	41.1	-2.1	1100
	FUND. BIODIVER	1696529	Unknown	Aegilops ventricosa Tausch.	Tausch.	ESP	Spain	Iglesuela del Cid, masico Marin		Te	40.1	-0.1	1500
00:00.0	GDAC	GDAC24840-1	Specimen	Aegilops ventricosa Tausch	Tausch	ES	Spain	Zamora, Boveda de Toro.		ZA			0
00:00.0	BC	70856	Specimen	Aegilops ventricosa Tausch	Tausch	ES	Spain	Burgos; Burgos		Bu	42.32	-3.67	
	FUND. BIODIVER	1487854	Unknown	Aegilops ventricosa Tausch	Tausch	ESP	Spain	P.N. Sierra Nevada		Gr	36.1	-3.1	
00:00.0	GDA	GDA52403-1-1	Specimen	Aegilops ventricosa Tausch	Tausch	ES	Spain	AlmerÃ-a, FondÃ³n, cabecera del B		AL			1720
1889-06-2	GJO	GJO-0015463	Unknown	Aegilops ventricosa Tausch		Spain	Spain	Spanien, AndalucÃ-a; VallÃ©e de la Sierra de la Nieva, moissons sur le calcaire					
	FUND. BIODIVER	1340285	Unknown	Aegilops ventricosa Tausch	Tausch	ESP	Spain	FrÃ-as		Bu	42.1	-3.1	550
	FUND. BIODIVER	1359463	Unknown	Aegilops ventricosa Tausch	Tausch	ESP	Spain	RincÃ³n de los Huertos, Moratalla		Mu	38.1	-1.1	1300
00:00.0	BC	830730	Specimen	Aegilops ventricosa Tausch	Tausch	ES	Spain	Palma; Al costat del Campament. C		PM	39.1192	2.884495	
	FUND. BIODIVER	1379222	Unknown	Aegilops ventricosa Tausch	Tausch	ESP	Spain	Colio, Cillorigo-Castro		S	43.1	-4.1	
	FUND. BIODIVER	92443	Unknown	Aegilops ventricosa Tausch	Tausch	ESP	Spain	SepÃºlveda, RendilÃ³n		Sg	41.1	-3.1	
	ESP004	NC010109	Specimen	Aegilops ventricosa TAUSCH		ESP	Spain	Sedano/Pesadas de Burgos 4km, Sedano,			42.7333	-3.68333	930
00:00.0	MGC	55146-1	Unknown	Aegilops ventricosa Tausch	Tausch	ES	Spain	Venta del Molinillo, orillas del RÃ-o		Gr	0	0	1130
00:00.0	SEV	5984-1	Specimen	Aegilops ventricosa Tausch var. c	(Cosson & D	ES	Spain	Near BaÃ±albufar		Mll			1
	FUND. BIODIVER	1700271	Unknown	Aegilops ventricosa		ESP	Spain	Cantavieja		Te	40.1	-0.1	1340
00:00.0	COFC	12401-1	Specimen	Aegilops ventricosa Tausch	Tausch	ES	Spain	Aldea Moret; minas		Cc			1
00:00.0	FUND. BIODIVER	1369203	Unknown	Aegilops ventricosa Tausch.	Tausch.	ESP	Spain	Los Llamosos		So	41.1	-2.1	1000
00:00.0	MA	636841-1	Specimen	Aegilops ventricosa		ES	Spain	Pedraza, orilla derecha del rÃ-o Ceg		Sg	41	-3	
	JBS	456-1	Specimen	Aegilops ventricosa Tausch var. v	Eig	ES	Spain	Escorca; Embassament des Gorg B		Mll	39.81	2.82	1
	IPK	AE 689	Living	Aegilops ventricosa Tausch var. comosa (Coss. & Du			Spain	La Alcarria			40.5164	-2.25	
	SIVIM	U-P02803:Aegi	Observation	Aegilops ventricosa Tausch	Tausch	ES	Spain	Castells de Serrella			38.63	-0.24	0
00:00.0	GDA	GDA22406-1	Specimen	Aegilops ventricosa Tausch.	Tausch.	ES	Spain	Granada, Cogollos Vega, prÃ³ximo		GR			1440
00:00.0	BC	70858	Specimen	Aegilops ventricosa Tausch	Tausch	ES	Spain	Mahon; S. Juan, c. MahÃ³n		PM	39.88	4.23	
1889-06-2	W	43540	Unknown	Aegilops ventricosa Tausch		ESP	Spain	VallÃ©e de la Sierra de la Nieva, moissons sur le calcaire.					
00:00.0	GDAC	GDAC26132-1	Specimen	Aegilops ventricosa Tausch	Tausch	ES	Spain	Granada, SÂª de Baza, Canaleja Ba		GR			0
00:00.0	SALA	43784-1	Specimen	Aegilops ventricosa Tausch	Tausch	ES	Spain	_; Cantalapiedra, La Carolina		Sa			
00:00.0	BC	70865	Specimen	Aegilops ventricosa Tausch	Tausch	ES	Spain	CalviÃ ; Sta. Ponsa Menorca		PM	39.52	2.48	

00:00.0	HUAL	1137-1	Specimen	Aegilops ventricosa Tansch	Tansch	ES	Spain	SÂª del Pozo, Nava del Espino		J	37.907	-2.892	
	ESP004	NC043504	Specimen	Aegilops ventricosa TAUSCH		ESP	Spain	Encinas de Esgueva, province of Valladolid			41.75	-4.1	832
	FUND. BIODIVER	1115800	Unknown	Aegilops ventricosa Tausch	Tausch	ESP	Spain	Moreruela de TÃ¡bara		Za	41.1	-5.1	
	FUND. BIODIVER	1533642	Unknown	Aegilops ventricosa Tsch.	Tsch.	ESP	Spain	Ãlava		Vi			
	FUND. BIODIVER	1593209	Unknown	Aegilops ventricosa Tausch	Tausch	ESP	Spain	Guardo		P	42.1	-4.1	
	ESP004	NC043500	Specimen	Aegilops ventricosa TAUSCH		ESP	Spain	Llucmajor, province of Baleares			39.4833	2.88333	43
	CZE122	01C2100534	Specimen	Aegilops ventricosa var. comosa (COSS. et DU		ESP	Spain	La Alcarria					
00:00.0	BC	70855	Specimen	Aegilops ventricosa Tausch	Tausch	ES	Spain	Lapuebla de Labarca; La Puebla		Vi	42.5	-2.57	
00:00.0	W	43516	Unknown	Aegilops ventricosa Tausch		ESP	Spain	Spanien: Castille: Castromonte Ã la S.ta Espania, lieux herbeux.					
00:00.0	BC	840351	Specimen	Aegilops ventricosa Tausch	Tausch	ES	Spain	Almanza; 4 kms. al O. de Almanza		Le	42.66	-5.01	950
00:00.0	MGC	70411-1	Unknown	Aegilops ventricosa Tausch	Tausch	ES	Spain	Sierra de las Nieves. Quejigales. Ca		Ma	36.691	-5.054	1326
	FUND. BIODIVER	1336388	Unknown	Aegilops ventricosa Tausch	Tausch	ESP	Spain	Tubilla del Agua		Bu	42.1	-3.1	
	SIVIM	T-P13701:Aegi	Observation	Aegilops ventricosa Tausch	Tausch	ES	Spain	MasÃ-a de Porcar, Cantavieja			40.44	-0.4	1400
	SIVIM	Q-P02164:Aegi	Observation	Aegilops ventricosa Tausch	Tausch	ES	Spain	Sant Joan			40.17	-0.41	1275
	SIVIM	Q-P07460:Aegi	Observation	Aegilops ventricosa Tausch	Tausch	ES	Spain	Torrebuceit			39.92	-2.53	0
00:00.0	W	43519	Unknown	Aegilops ventricosa Tausch		ESP	Spain	Spanien: Alava: La Puebla, paturages.					
	RUS001	VIR100602217	Specimen	Aegilops ventricosa Tausch		ESP	Spain						
	FUND. BIODIVER	62078	Unknown	Aegilops ventricosa Tausch	Tausch	ESP	Spain	La Fuente de San Esteban, MuÃ±oz		Sa	40.1	-6.1	
	SIVIM	Q-P08745:Aegi	Observation	Aegilops ventricosa Tausch	Tausch	ES	Spain	Guardo ,			42.7	-4.95	1120
00:00.0	SALA	14387-1	Specimen	Aegilops ventricosa Tausch	Tausch	ES	Spain	_; La Fuente de San Esteban, Boad		Sa			
	FUND. BIODIVER	1369213	Unknown	Aegilops ventricosa Tausch.	Tausch.	ESP	Spain	Sierra del Almuerzo		So	41.1	-2.1	
	FUND. BIODIVER	1376740	Unknown	Aegilops ventricosa Tausch.	Tausch.	ESP	Spain	Sierra de Baza, Parque Natural		Gr	37.1	-2.1	
	CZE122	01C2100522	Specimen	Aegilops ventricosa TAUSCH		ESP	Spain	La Alcarria, Spanien					
	DEU146	AE 617	Specimen	Aegilops ventricosa TAUSCH		ESP	Spain	La Alcarria, Spanien					
	DEU146	AE 1054	Specimen	Aegilops ventricosa TAUSCH		ESP	Spain	Bellver (Baleares)					
	MA	640319-1	Specimen	Aegilops ventricosa Tausch	Tausch	ES	Spain	Tubilla de Agua		Bu	42	-3	
	BDBCV-General	279919	Observation	Aegilops ventricosa		ESPA	Spain	Teresa de Cofren	El Valle de Ayora	Valenc	39.146	-0.97503	
	FUND. BIODIVER	1700458	Unknown	Aegilops ventricosa		ESP	Spain	Cantavieja		Te	40.1	-0.1	1400
00:00.0	BC	800084	Specimen	Aegilops ventricosa Tausch	Tausch	ES	Spain	Orihuela del Tremedal; Orihuela		Te	40.4635	-1.70236	1400
	SIVIM	U-P05225:Aegi	Observation	Aegilops ventricosa Tausch	Tausch	ES	Spain	LogroÃ±o, ZorraquÃ-n: Turgaiza			42.27	-3.12	0
	SIVIM	R-P08189:Aegi	Observation	Aegilops ventricosa Tausch	Tausch	ES	Spain	Plana de can Soteres, sobre la boca E del			41.54	1.68	0
00:00.0	SALA	100726-1	Specimen	Aegilops ventricosa Tausch	Tausch	ES	Spain	_; ZorraquÃ-n, monte Turgaiza.		Lo	42.33	-3.05	
1876-06-2	W	43522	Unknown	Aegilops ventricosa Tausch		ESP	Spain	Hispania: In valle fluv. Darro supra Granatam. In itinere hispanico-lusitanico.					
	FUND. BIODIVER	1476397	Unknown	Aegilops ventricosa Tausch	Tausch	ESP	Spain	Vallanca, hacia SalvacaÃ±ete, por l		V	39.1	-1.1	1050
00:00.0	UNEX	10380-1	Observation	Aegilops ventricosa Tausch	_	ESP	Spain	Ribera del Fresno: Suelos bÃ¡sicos		Ba	38.5	-6.2	
	IPK	70861	Living	Aegilops ventricosa Tausch		ESP	Spain	Bellver (Baleares)					
	SIVIM	Q-P07408:Aegi	Observation	Aegilops ventricosa Tausch	Tausch	ES	Spain	Finca Navodres			39.65	-2.06	0
00:00.0	SALA	24141-1	Specimen	Aegilops ventricosa Tausch	Tausch	ES	Spain	_; Valoria la Buena		Va			
	FUND. BIODIVER	1481199	Unknown	Aegilops ventricosa Tausch	Tausch	ESP	Spain	P.N. ArchipiÃ©lago de Cabrera		PM	38.1	2.1	
	FUND. BIODIVER	1125912	Unknown	Aegylops ventricosa Tausch	Tausch	ESP	Spain	Encinas de Esgueva		Va	41.1	-3.1	
00:00.0	BC	109335	Specimen	Aegilops ventricosa Tausch	Tausch	ES	Spain	Palma de Mallorca; Al costat del Ca		PM	39.16	2.94	

00:00.0	MGC	60824-1	Unknown	Aegilops ventricosa Tausch	Tausch	ES	Spain	Venta del Molinillo. RÃo Fardes		Gr	0	0	1
00:00.0	REDIAM-CMA	198480	Observation	Aegilops ventricosa		ESP	Spain		Cazorla	J	37.882	-2.96269	1339
	MA	630046-1	Specimen	Aegilops ventrocosa Taush	Taush	ES	Spain	Aguliar de Campoo, pr. Renedo de		P	42	-4	
	JBS	459-1	Specimen	Aegilops ventricosa Tausch	Tausch	ES	Spain	Escorca; Embassament des Gorg B		Mll	39.81	2.82	1
	FUND. BIODIVER	1030248	Unknown	Aegilops ventricosa Tausch	Tausch	ESP	Spain	Noalejo Marceral		J	37.1	-3.1	1300
00:00.0	GDAC	GDAC11572-1	Specimen	Aegilops ventricosa Tausch	Tausch	ES	Spain	Granada, SÂª de Alfacar, Cerro de l		GR			0
	FUND. BIODIVER	70875	Unknown	Aegilops ventricosa Tsch.	Tsch.	ESP	Spain	Pedrosillo el Ralo		Sa	40.1	-5.1	
00:00.0	GDA	GDA18053-1-2	Specimen	Aegilops ventricosa Tausch.	Tausch.	ES	Spain	Granada, SÂª Nevada, Monachil, ba		GR			1600
	BC	115532	Specimen	Aegilops ventricosa Tausch	Tausch	ES	Spain	Puerto Real; Calerones. S. Fernand		Ca	36.51	-6.04	
1876-07-0	W	44027	Unknown	Aegilops ventricosa Tausch		ESP	Spain	Hispania: In valle fluv. Darro supra Granatam. In itinere hispanico-lusitanico.					
00:00.0	SALA	52028-1	Specimen	Aegilops ventricosa Tausch	Tausch	ES	Spain	_; Abezames		Za			
	FUND. BIODIVER	1030249	Unknown	Aegilops ventricosa Tausch	Tausch	ESP	Spain	Noalejo Marceral		J	37.1	-3.1	1100
00:00.0	GDA	GDA22406-1-1	Specimen	Aegilops ventricosa Tausch.	Tausch.	ES	Spain	Granada, Cogollos Vega, prÃ³ximo		GR			1440
	FUND. BIODIVER	1340284	Unknown	Aegilops ventricosa Tausch	Tausch	ESP	Spain	Pancorbo		Bu	42.1	-2.1	680

ו					

aron					

s apud Hispanos longe meritissimus.					

aron					

[illegible]					

rra de la Fuensantas, sol. cretaceo (iter II. Hispanicum 1890);					
ssidum misit, Dnus petr. Loefling, Sae Cathol: Majtis olim botanicus indefessus.					

ler Sierra Morena des ......)					

[illegible]o arenosio. El purito Agremenses. ### Rubera.					

;					
