# Supplementary material for: Identification of ecogeographical gaps in the Spanish Aegilops collections with potential tolerance to drought and salinity
Source: PeerJ. 2017 Jul 27;5:e3494. doi: 10.7717/peerj.3494 (PMC5534164; doi:10.7717/peerj.3494)
Supplement: Supplemental Information 3 [file peerj-05-3494-s017.pdf]

Genero	Especie	Autabre	IDCita	Pais	Provincia	Localidad	UTM	UTM_Aprox	Altitud	Registro	Fecha
Aegilops	macrochaeta	Schutt y Huet	1013199	España	Almería	El Alquián	30SWF57	1			1975
Aegilops	macrochaeta	Schultz. et Huet.	861710	España	Gerona	Prados y márgenes secos	31TDG89	1			1880
Aegilops	lorentii	Hochst	2442819	España	Guadalajara	Córcoles	30TWK28	0			1984
Aegilops	lorentii	Hochst	2442818	España	Guadalajara	El Recuenco	30TWK59	0			1984
Aegilops	biuncialis	Vis.	1027212	España	Jaén	Marmolejo	30SVH0314	0	200		1994
Aegilops	lorentii	Hoschst.	1013131	España	Almería	Llano de la Charca del Sa	30SWF18	1		DUDOSO	1975
Aegilops	lorentii	Hochst	1835092	España	Ciudad Real	Lagunas de Ruidera, lagu	30SWJ1515	0		DUDOSO	2005
Aegilops	lorentii	Hochst.	1487851	España	Granada	P.N. Sierra Nevada	30SVG70	1		DUDOSO	1987
Aegilops	lorentii	Hochst.	1376734	España	Granada	Sierra de Baza, Parque Na	30SWG13	1		DUDOSO	1991
Aegilops	lorentii	Hochst.	1029115	España	Jaén	Andújar, desembocadura	30SUH9440	0	400	DUDOSO	1995
Aegilops	lorentii	Hochst.	1029114	España	Jaén	Andújar, Río Valmayor, V	30SUH9444	0	500	DUDOSO	1995
Aegilops	lorentii	Hochst.	1029113	España	Jaén	Andújar, desembocadura	30SVH0314	0	200	DUDOSO	1995
Aegilops	lorentii	Hochst.	1029112	España	Jaén	Villanueva del Arzobispo	30SVH9926	0	620	DUDOSO	1995
Aegilops	lorentii	Hochst.	1086009	España	Madrid	Arganda del Rey	30TVK66	1		DUDOSO	1986
Aegilops	lorentii	Hochst	58086	España	Salamanca	Cantalapiedra	30TUL15	1		DUDOSO	1987
Aegilops	geniculata	Roth	36854	España	Álava	Berrícano	30TWN2154	0	600		1985
Aegilops	geniculata	Roth	36855	España	Álava	Martioda	30TWN1847	0	500		1985
Aegilops	geniculata	Roth	36856	España	Álava	Araia	30TWN5548	0	600		1985
Aegilops	geniculata	Roth	36857	España	Álava	Arreo	30TWN0136	0	700		1985
Aegilops	geniculata	Roth	36858	España	Álava	Ocio	30TWN1323	0	600		1985
Aegilops	geniculata	Roth	36859	España	Álava	Corres	30TWN4727	0	700		1985
Aegilops	geniculata	Roth	36860	España	Álava	Labastida	30TWN1516	0	500		1985
Aegilops	geniculata	Roth	36861	España	Álava	Oyón	30TWN4505	0	450		1985
Aegilops	ovata	L.	54282	España	Salamanca	Alba de Tormes, cercanía	30TTL82	1			1977
Aegilops	geniculata	Roth	54856	España	Salamanca	Bóveda del río Almar	30TUL12	1			1983
Aegilops	geniculata	Roth	54857	España	Salamanca	Macotera	30TUL02	1			1983
Aegilops	geniculata	Roth.	56040	España	Valladolid	Encinas de Esgueva, La Ro	30TVM02	1			1985
Aegilops	geniculata	Roth.	56041	España	Burgos	Villaescusa de Roa, junto	30TVM12	1			1985
Aegilops	ovata	L.	56188	España	Valladolid	Aguasal	30TUL67	1			1908
Aegilops	ovata	L.	56189	España	Valladolid	Bocigas	30TUL56	1			1908
Aegilops	ovata	L.	56190	España	Valladolid	Pedrajas de San Esteban	30TUL67	1			1908
Aegilops	ovata	L.	56191	España	Valladolid	Olmedo	30TUL57	1			1908
Aegilops	geniculata	Roth	57500	España	Salamanca	Matilla de los Caños del r	30TTL52	1			1984
Aegilops	geniculata	Roth	58087	España	Salamanca	Cantalapiedra	30TUL15	1			1987
Aegilops	ovata	L.	58155	España	Salamanca	Babilafuente	30TTL93	1			1977
Aegilops	geniculata	Roth	59183	España	Salamanca	Pelabravo	30TTL83	1			1991
Aegilops	geniculata	Roth	59599	España	Salamanca	Monterrubio de Armuña	30TTL74	1			1991

Aegilops	geniculata	Roth.	65367	España	Salamanca	Guijuelo	30TTK79	1			1989
Aegilops	geniculata	Roth	70820	España	Segovia	San Ildefonso o la Granja	30TVL1429	0			1995
Aegilops	geniculata	Roth	70821	España	Segovia	Torre Val de San Pedro	30TVL2849	0			1995
Aegilops	geniculata	Roth	70822	España	Segovia	Cubillo	30TVL2353	0			1995
Aegilops	ovata	L.	70860	España	Salamanca	Valdunciel	30TTL75	1			1976
Aegilops	ovata	L.	70861	España	Salamanca	Arcediano, camino a La V	30TTL85	1			1976
Aegilops	ovata	L.	70862	España	Salamanca	Moriscos, camino hacia G	30TTL84	1			1976
Aegilops	ovata	L.	70863	España	Salamanca	La Orbada	30TTL95	1			1976
Aegilops	ovata	L.	70864	España	Salamanca	Parada de Rubiales, cami	30TTL95	1			1976
Aegilops	ovata	L.	70865	España	Salamanca	Castellanos de Villiquera,	30TTL74	1			1976
Aegilops	ovata	L.	70866	España	Salamanca	Aldeanueva de Figueroa	30TTL85	1			1976
Aegilops	ovata	L.	70867	España	Salamanca	La Velles	30TTL85	1			1976
Aegilops	ovata	L.	70868	España	Salamanca	Valverdón	30TTL64	1			1976
Aegilops	ovata	L.	70869	España	Salamanca	Espino de la Orbada	30TTL95	1			1976
Aegilops	ovata	L.	70870	España	Salamanca	Villaverde de Guareña	30TTL84	1			1976
Aegilops	geniculata	Rotch.	72899	España	Salamanca	Doñinos de Salamanca	30TTL63	1			1986
Aegilops	geniculata	Rotch.	72900	España	Salamanca	Salamanca, Tejares	30TTL73	0			1986
Aegilops	geniculata	Roth.	76095	España	Zamora	Castrillo de la Guareña	30TUL06	1			1984
Aegilops	geniculata	Roth.	78788	España	Valladolid	Mojados	30TUL68	1			1983
Aegilops	geniculata	Roth.	78789	España	Valladolid	Portillo	30TUL69	1			1983
Aegilops	geniculata	Roth.	78790	España	Valladolid	Quintanilla de Arriba	30TUM90	1			1983
Aegilops	geniculata	Roth.	78791	España	Valladolid	Olmos de Esgueva	30TUM71	1			1983
Aegilops	geniculata	Roth.	78792	España	Palencia	Soto de Cerrato	30TUM84	1			1983
Aegilops	ovata	L.	78964	España	Salamanca	Aldealengua	30TTL84	1			1977
Aegilops	geniculata	Roth	80849	España	Segovia	Sepúlveda, Sepúlveda, er	30TVL3771	0			1985
Aegilops	geniculata	Roth	80850	España	Segovia	Sepúlveda, Sepúlveda, er	30TVL3671	0			1985
Aegilops	geniculata	Roth	80851	España	Segovia	Carrascal del Río, Carrasc	30TVL2674	0			1985
Aegilops	geniculata	Roth.	82305	España	Segovia	Cedillo de la Torre	30TVL48	1			1985
Aegilops	geniculata	Roth.	82306	España	Segovia	Fresno de la Fuente	30TVL48	1			1985
Aegilops	geniculata	Roth	83531	España	Zamora	Tábara, La Breñica	30TTM53	1			1997
Aegilops	geniculata		85990	España	Soria	Herrera de Soria	30TVM92	0			1987
Aegilops	geniculata		86617	España	Soria	Aldehuela de Periáñez, h	30TWM52	0			1982
Aegilops	geniculata		86868	España	Soria	Arguijo	30TWM44	0			1983
Aegilops	geniculata	Roth	92446	España	Segovia	Fuentidueña (El Rebollo)	30TVL1890	0			1989
Aegilops	geniculata	Roth	94006	España	Zamora	Tábara, La Breñica	30TTM5336	0			1997
Aegilops	geniculata	Roth.	96900	España	Zamora	Fermoselle	29TQF17	1			1983
Aegilops	geniculata	Roth.	96901	España	Zamora	Pereruela, San Román de	30TTL69	1			1983
Aegilops	geniculata	Roth.	96902	España	Zamora	Pereruela	30TTL58	1			1983
Aegilops	geniculata	Roth	99839	España	Zamora	Tábara	30TTM53	1			1982

Aegilops	geniculata	Roth	99840	España	Zamora	Pozuelo de Tábara	30TTM53	1			1982
Aegilops	geniculata	Roth	101226	España	Soria	Recuerda, Hacia Morales	30TWL09	0	900		1996
Aegilops	geniculata	Roth	102488	España	Ávila	Padiernos	30TUK49	1			1989
Aegilops	geniculata	Roth	102489	España	Ávila	Muñogalindo	30TUK39	1			1989
Aegilops	geniculata	Roth	103038	España	León	Boñar, Rucayo	30TUN15	1			1984
Aegilops	ovata	L.	104259	España	Palencia	Castrejón de la Peña, ent	30TUN64	1			1957
Aegilops	geniculata	Roth.	109362	España	León	Los Barrios de Luna, Miñ	30TTN64	1			1983
Aegilops	geniculata	Roth	110577	España	Palencia	Cervera de Pisuerga, Cela	30TUN85	1			1989
Aegilops	geniculata	Roth	113680	España	Valladolid	Renedo	30TUM61	1			2001
Aegilops	geniculata	Roth	115631	España	Palencia	Guardo, Guardo	30TUN4939	0			1990
Aegilops	geniculata	Roth	115632	España	Palencia	Guardo, Muñeca	30TUN5239	0			1990
Aegilops	geniculata	Roth.	116427	España	Valladolid	Íscar	30TUL7281	0			1987
Aegilops	geniculata	Roth.	116428	España	Valladolid	Almenara de Adaja	30TUL6163	0			1987
Aegilops	geniculata	Roth.	116429	España	Valladolid	Aldea de San Miguel	30TUL6689	0			1987
Aegilops	geniculata	Roth.	116430	España	Valladolid	Aguasal	30TUL6275	0			1987
Aegilops	ovata	L.	117644	España	Burgos	Castrillo de la Reina	30TVM84	1			1924
Aegilops	geniculata	Roth.	118440	España	León	La Pola de Gordón, Santa	30TTN85	0			1985
Aegilops	geniculata	Roth.	118441	España	León	La Pola de Gordón, Bebe	30TTN85	0			1985
Aegilops	geniculata	Roth	120458	España	Segovia	Montejo de la Vega de la	30TVM4800	0			1996
Aegilops	geniculata	Roth	120459	España	Segovia	Valdevacas de Montejo,	30TVL4699	0			1996
Aegilops	geniculata	Roth	120460	España	Segovia	Valdevacas de Montejo,	30TVL4898	0			1996
Aegilops	ovata		135668	España	Castellón	Fredes, La Senia, Tossal R	31TBF51	1			1956
Aegilops	ovata	L.	261210	España	Tenerife	La Palma	28RBS27	0			1983
Aegilops	ovata	L.	261211	España	Tenerife	La Palma, Mirca, carreter	28RBS27	0	150		1983
Aegilops	ovata	L.	847027	España	Valencia	Sierra de la Murta	30SYJ33	1			1951
Aegilops	ovata		856220	España	Alicante	Alicante, Talaia-Rambla C	30SYH14	1			1957
Aegilops	ovata		856281	España	Alicante	Alicante, hacia Talaia	30SYH14	1			1957
Aegilops	ovata	L.	860300	España	Alicante	Alrededores de Polop	30SYH57	1			1934
Aegilops	ovata	L.	860301	España	Alicante	Sierra de San Julián	30SYH24	1			1934
Aegilops	ovata	L.	861709	España	Gerona	Besalú	31TDG77	1			1880
Aegilops	ovata	L.	902845	España	Gerona	Prats de Llusanés, sitios e	31TDG25	1			1880
Aegilops	ovata	L.	902846	España	Barcelona	Borredá	31TDG16	1			1880
Aegilops	ovata	L.	913680	España	Barcelona	Massif de:Tibidabo,Levar	31TDF28	1			1974
Aegilops	ovata	L.	913740	España	Barcelona	Massif de Ordal	31TDF08	1			1974
Aegilops	ovata	L.	913754	España	Barcelona	Massif de St. Llorenç	31TDG11	1			1974
Aegilops	ovata	L.	913768	España	Barcelona	Serra Superior del Valles	31TDF39	1			1974
Aegilops	ovata	L.	918138	España	Barcelona	Garriga	31TDG41	1			1883
Aegilops	ovata	L.	918140	España	Barcelona	Plana de Vich	31TDG34	1			1877
Aegilops	ovata	L.	918144	España	Barcelona	Montserrat	31TDG00	1			1953

Aegilops	ovata	L.	925115	España	Tarragona	Cuenca del Gaiá	31TCF66	1			1950
Aegilops	ovata	L.	930420	España	Tarragona	L'Aleixar-Vilaplana del Ca	31TCF36	1			1972
Aegilops	ovata	L.	930421	España	Tarragona	Puig de Marc, Valle El Tit	31TCF38	1			1972
Aegilops	ovata	L.	930422	España	Tarragona	Siurana de Prades, Capaf	31TCF26	1			1972
Aegilops	ovata	L.	930423	España	Tarragona	Plans de Prades, Plans de	31TCF57	1			1972
Aegilops	ovata	L.	930424	España	Tarragona	Macizo de La Guardia	31TCF48	1			1972
Aegilops	ovata	L.	939739	España	Lérida	Balaguer	31TCG12	1			1925
Aegilops	ovata		960553	España	Huesca	Jaca	30TYN01	1			1906
Aegilops	ovata	L.	974822	España	Zaragoza	Calatayud	30TXL17	1			1894
Aegilops	ovata	L.	976282	España	Teruel	Torrecilla de Alcañiz	30TYL43	1			1903
Aegilops	ovata	L.	979128	España	Teruel	Valdealgorfa	30TYL44	1			1901
Aegilops	ovata	L.	979129	España	Teruel	Cercanías de Teruel	30TXK66	1			1896
Aegilops	ovata	L.	979130	España	Teruel	Blancas	30TXL21	1			1896
Aegilops	ovata	L.	979131	España	Teruel	Tramacastilla	30TXK27	1			1896
Aegilops	ovata	L.	992693	España	Guadalajara	Tartanedo	30TWL93	1			1948
Aegilops	ovata	L.	992694	España	Guadalajara	Turmiel	30TWL74	1			1948
Aegilops	ovata	L.	992695	España	Guadalajara	Embid	30TXL03	1			1948
Aegilops	geniculata		993534	España	Guadalajara	Casar de Talamanca	30TVL60	1			1977
Aegilops	geniculata		993536	España	Madrid	Valdepiélagos	30TVL61	1			1977
Aegilops	geniculata		993576	España	Guadalajara	Tamajón	30TVL73	0			1977
Aegilops	ovata	L.	995300	España	Cuenca	Almonacid del Marquesa	30SWK10	1			1899
Triticum	ovatum	G.G.	996412	España	Cuenca	Puente Vadillos	30TWK68	1			1948
Aegilops	geniculata		998068	España	Cuenca	Altos de Cabrejas	30TWK5736	0			1978
Aegilops	geniculata		998069	España	Cuenca	Cuenca	30TWK7036	0			1978
Aegilops	geniculata		998070	España	Cuenca	Tragacete	30TXK0066	0			1978
Aegilops	geniculata		998071	España	Cuenca	Cañete	30TXK1337	0			1978
Aegilops	geniculata		1000495	España	Cuenca	Fresneda de Allarejos	30SWK51	1			1977
Aegilops	geniculata		1000496	España	Cuenca	Jábala, Boniches	30SXK12	0			1977
Aegilops	geniculata		1000497	España	Cuenca	Venta de Cabrejas	30TWK53	0			1977
Aegilops	geniculata		1001949	España	Cuenca	Sierra de San Felipe	30TWK9970	0			1978
Aegilops	geniculata		1001955	España	Cuenca	Puerto de Cabrejas a Cue	30TWK6236	0			1978
Aegilops	geniculata		1001970	España	Cuenca	Valle de Valdemeca	30TXK0851	0			1978
Aegilops	geniculata		1001978	España	Cuenca	Cañete a Boniches	30SXK1323	0			1978
Aegilops	geniculata		1002566	España	Albacete	Bosque de Alpera	30SXJ51	1			1985
Aegilops	ovata	L.	1010455	España	Murcia	Mazarrón	30SXG46	1			1971
Aegilops	ovata	L.	1010574	España	Murcia	Casa de las Coronadas, M	30SXG46	0			1975
Aegilops	ovata	L.	1018236	España	Granada	La Gabia	30SVG30	1			1981
Aegilops	ovata	L.	1024006	España	Jaén	Valdeazores	30SVH54	1			1948
Aegilops	ovata	L.	1024007	España	Jaén	Valle de las Correderas	30SVH54	1			1948

Aegilops	geniculata		1025028	España	Jaén	E. Martos y Alcaudete	30SVG17	1			1986
Aegilops	geniculata		1025029	España	Jaén	Alcaudete	30SVG06	0			1986
Aegilops	ovata		1025032	España	Jaén	E. Martos y Alcaudete	30SVG17	1			1986
Aegilops	ovata		1025033	España	Jaén	Mengíbar, Bailén	30SVH2901	0			1986
Aegilops	geniculata	Roth.	1028170	España	Jaén	Valdepeñas de Jaén, carr	30SVG26	0			1994
Aegilops	geniculata	Roth.	1028171	España	Jaén	Valdepeñas de Jaén	30SVG26	0			1994
Aegilops	ovata		1035355	España	Málaga	Sierra de Aguas, Arroyo d	30SUF48	0			1975
Aegilops	ovata		1035513	España	Málaga	Base suroriental de la Sie	30SUF48	0			1975
Aegilops	ovata		1035554	España	Málaga	Zonas elevadas de la Sier	30SUF38	0			1975
Aegilops	ovata		1035623	España	Málaga	Laderas del Arroyo de Pa	30SUF48	0			1975
Aegilops	ovata		1035699	España	Málaga	Sierra de Aguas, Carratra	30SUF38	0	500		1975
Aegilops	ovata		1035830	España	Málaga	Sierra de Aguas, Arroyo d	30SUF38	0			1975
Aegilops	ovata	L.	1036000	España	Málaga	Sierra de Aguas, Carratra	30SUF38	0			1975
Aegilops	ovata		1036558	España	Málaga	Málaga	30SUF76	1			1956
Aegilops	ovata		1036574	España	Málaga	Málaga	30SUF76	1			1956
Aegilops	ovata	L.	1036793	España	Málaga	Sierra Tejeda	30SVF08	1			1948
Aegilops	ovata	L.	1036794	España	Málaga	Sierra Almijara	30SVF27	1			1948
Aegilops	geniculata	Roth	1038228	España	Málaga	Sierra de Mijas	30SUF55	0			1987
Aegilops	geniculata	Roth	1043751	España	Málaga	Antequera	30SUF69	0			1990
Aegilops	geniculata	Roth	1043752	España	Málaga	Antequera, Torcal	30SUF69	0			1990
Aegilops	geniculata	Roth	1043753	España	Málaga	Canillas de Aceituno	30SVF08	0			1990
Aegilops	geniculata	Roth	1043754	España	Málaga	Carratraca, Sierra de Agu	30SUF37	0			1990
Aegilops	geniculata	Roth	1043755	España	Málaga	Istán, Bohornoque	30SUF24	0			1990
Aegilops	geniculata	Roth	1043756	España	Málaga	Málaga	30SUF76	0			1990
Aegilops	geniculata	Roth	1043757	España	Málaga	Sierra de Mijas	30SUF55	0			1990
Aegilops	geniculata	Roth	1043758	España	Málaga	Mollina	30SUG50	1			1990
Aegilops	geniculata	Roth	1043759	España	Málaga	Nerja	30SVF2268	0			1990
Aegilops	geniculata	Roth	1043760	España	Málaga	Ojén, Sierra Blanca	30SUF34	0			1990
Aegilops	geniculata	Roth	1043761	España	Málaga	Rincón de la Victoria	30SUF86	0			1990
Aegilops	geniculata	Roth	1043762	España	Málaga	San Pedro de Alcántara	30SUF24	0			1990
Aegilops	geniculata	Roth	1043763	España	Málaga	Sierra Tejeda	30SVF08	0			1990
Aegilops	geniculata	Roth	1050669	España	Cádiz	Sierras de Algeciras	30STE79	1			1985
Aegilops	ovata		1050671	España	Cádiz	Sierra de Ubrique	30STF86	1			1986
Aegilops	ovata		1050672	España	Cádiz	Ubrique	30STF86	1			1986
Aegilops	ovata		1050673	España	Cádiz	Villaluenga del Rosario	30STF86	0			1986
Aegilops	geniculata	Roth	1052572	España	Cádiz	Montes de Propios de Je	29SQA56	1			1991
Aegilops	ovata		1060398	España	Cádiz	Los Barrios	30STF70	0			1986
Aegilops	geniculata	Roth	1062079	España	Huelva	Sierra de Aracena	29SQB19	0			1985
Aegilops	ovata	L.	1066943	España	Sevilla	Morón	30STG81	1			1948

Aegilops	ovata		1067620	España	Sevilla	Coria del Río	29SQB63	1			1955
Aegilops	ovata		1067621	España	Sevilla	Puebla del Río	29SQB62	1			1955
Aegilops	ovata	L.	1067624	España	Sevilla	Alcalá de Guadaira	30STG43	1			1888
Aegilops	ovata	L.	1067625	España	Sevilla	Sevilla	30STG44	1			1897
Aegilops	ovata	L.	1067626	España	Sevilla	Alcalá de Guadaira	30STG43	1			1897
Aegilops	ovata	L.	1067627	España	Sevilla	Pedroso	30STG59	1			1897
Aegilops	ovata	L.	1067631	España	Sevilla	Las Rozas, Encarnaciones	30STG90	0			1984
Aegilops	ovata	L.	1070040	España	Sevilla	Carmona	30STG65	1			1891
Aegilops	ovata	L.	1072128	España	Sevilla	Arroyo del Salado, cerca	30STG80	0			1984
Aegilops	ovata	L.	1072129	España	Sevilla	Morón de la Frontera, alr	30STG81	0			1984
Aegilops	ovata	L.	1072130	España	Sevilla	Pruna, Algámitas, Sierra d	30SUF09	0			1984
Aegilops	geniculata	L.	1072714	España	Badajóz	Antigua Finca Las Rocillas	29SPD70	1			1989
Aegilops	geniculata		1073152	España	Córdoba	Doña Mencía	30SUG75	1			1986
Aegilops	geniculata		1073153	España	Córdoba	Puente Genil	30SUG44	1			1986
Aegilops	ovata		1073156	España	Córdoba	Baena	30SUG8066	0			1986
Aegilops	ovata		1074140	España	Córdoba	Puente Genil	30SUG44	1			1986
Aegilops	geniculata		1074145	España	Córdoba	Baena	30SUG8066	0			1986
Aegilops	ovata	L.	1077194	España	Toledo	Ontígola	30SVK52	1			1972
Aegilops	ovata	L.	1077308	España	Toledo	Polán	30SVK00	1			1882
Aegilops	ovata	L.	1077309	España	Toledo	Toledo, término municipa	30SVK10	1			1882
Aegilops	ovata		1082854	España	Madrid	Chinchón	30TVK64	1			1974
Aegilops	ovata	L.	1084632	España	Madrid	Tielmes	30TVK75	1			1972
Aegilops	geniculata		1085535	España	Madrid	Loeches	30TVK67	1			1977
Aegilops	geniculata		1085536	España	Madrid	Ciudad Universitaria	30TVK47	0			1977
Aegilops	geniculata		1085537	España	Madrid	Ajalvir	30TVK58	0			1977
Aegilops	geniculata	Roth.	1086010	España	Madrid	Arganda del Rey	30TVK66	1			1986
Aegilops	geniculata		1090389	España	Madrid	Cobeña	30TVK59	0			1977
Aegilops	ovata	L.	1090545	España	Madrid	Villarejo de Salvanés	30TVK74	1			1972
Aegilops	ovata		1093225	España	Cáceres	Peraleda de la Mata, Tori	30STK81	1			1987
Aegilops	geniculata		1093227	España	Cáceres	Dehesa de los Caballos, P	29TQE43	0			1989
Aegilops	geniculata		1093228	España	Cáceres	Dehesa de los Caballos, P	29TQE43	0			1989
Aegilops	geniculata		1093231	España	Cáceres	El Arco, Cañaveral	29SQE20	0			1989
Aegilops	geniculata		1093233	España	Cáceres	Finca de Valdelasyeguas,	29SPD96	0			1989
Aegilops	geniculata		1093236	España	Cáceres	Finca de Araya, Arroyo de	29SQD07	1			1989
Aegilops	geniculata		1093238	España	Cáceres	Cerro de Aldeamoret	29SQD26	1			1989
Aegilops	geniculata		1093239	España	Cáceres	Cerro de Aldeamoret	29SQD26	1			1989
Aegilops	geniculata		1093242	España	Cáceres	Coria	29SQE12	1			1989
Aegilops	geniculata		1093252	España	Cáceres	Almaraz	30STK70	0			1989
Aegilops	geniculata		1093253	España	Cáceres	Almaraz	30STK70	0			1989

Aegilops	geniculata		1093255	España	Cáceres	Cuesta de Araya, Garrovi	29SQD08	0			1989
Aegilops	geniculata		1093257	España	Cáceres	El Arco, Cañaveral	29SQE20	0			1989
Aegilops	geniculata	Rothm.	1115791	España	Zamora	Dehesa de Misleo	30TTM63	1			1984
Aegilops	geniculata	Rothm.	1115792	España	Zamora	Puente Quintos	30TTM63	0			1984
Aegilops	geniculata	Rothm.	1115793	España	Zamora	Faramontanos de Tábara	30TTM63	0			1984
Aegilops	geniculata	Rothm.	1115801	España	Zamora	Pozuelo de Tábara	30TTM53	1			1984
Aegilops	geniculata	Rothm.	1115803	España	Zamora	Moreruela de Tábara	30TTM63	1			1984
Aegilops	geniculata	Roth	1120219	España	Zamora	Abezames, Gafos	30TTM9911	0			1994
Aegilops	geniculata	Roth	1120220	España	Zamora	Fresno de la Ribera, La Ce	30TTM80	1			1994
Aegilops	geniculata	Roth	1120221	España	Zamora	Aspariegos, Las Cabecina	30TTM81	0			1994
Aegilops	geniculata	Roth	1120222	España	Zamora	Algodre, Las Gavias	30TTM80	1			1994
Aegilops	geniculata	Roth	1120223	España	Zamora	Santa María de la Vega	30TTM66	1			1994
Aegilops	ovata	L.	1125485	España	Valladolid	Laguna de Duero	30TUM50	1			1900
Aegilops	geniculata	Roth	1135157	España	León	Castrovega de Valmadrig	30TUM18	1			1986
Aegilops	geniculata	Roth	1135159	España	León	Ardón	30TTN90	1	820		1984
Aegilops	geniculata	Roth	1135160	España	León	Castrofuerte	30TTM97	1	743		1984
Aegilops	geniculata	Roth	1135161	España	León	Puente Villarente	30TTN91	1	754		1984
Aegilops	geniculata	Roth	1135162	España	León	Matadeón	30TUM09	1	910		1984
Aegilops	geniculata	Roth	1141561	España	León	Ardoncino	30TTN80	1	830		1984
Aegilops	geniculata	Roth	1141567	España	León	Villamañán	30TTM89	1	760		1984
Aegilops	geniculata	Roth	1141586	España	León	Puente Castro	30TTN91	1	910		1984
Aegilops	ovata	L.	1186718	España	Islas Baleares	Mallorca, Son Coll-Banya	31SDD59	1			1958
Aegilops	ovata	L.	1188175	España	Islas Baleares	Mallorca, Valldemossa	31SDD69	1			1958
Aegilops	ovata	L.	1189434	España	Islas Baleares	Cabrera, Camí Ses Cases (	31SDD93	1			1936
Aegilops	ovata	L.	1189439	España	Islas Baleares	Menorca, Rafal Fort	31SEE92	1			1901
Aegilops	geniculata	Roth.	1332455	España	Jaén	Río Yeguas	30SUH9440	0			1996
Aegilops	geniculata	Roth	1335996	España	Navarra	Aibar, Valladana	30TXN3713	0	450		1991
Aegilops	geniculata	Roth	1335997	España	Navarra	Castillonuevo, Sierra de L	30TXN5825	0	910		1991
Aegilops	geniculata	Roth	1335998	España	Navarra	Lumbier, foz del río Irati	30TXN3921	0	450		1991
Aegilops	geniculata	Roth	1335999	España	Navarra	Monreal, Higa	30TXN2028	0	1280		1991
Aegilops	geniculata	Roth	1336000	España	Navarra	Orísoain, Valdorba, camii	30TXN1417	0	700		1991
Aegilops	geniculata	Roth	1336001	España	Navarra	Rocaforte, el Romeral	30TXN3719	0	560		1991
Aegilops	geniculata	Roth	1336002	España	Navarra	Ujué, camino de Chucho	30TXN2111	0	650		1991
Aegilops	geniculata	Roth	1336387	España	Burgos	Montorio	30TVN3715	0			1990
Aegilops	geniculata	Roth.	1339635	España	Albacete	Albacete, provincia	30SWH6760	0			1996
Aegilops	geniculata	Roth	1340021	España	Madrid	Madrid, Campus Universi	30TVK37	0			1993
Aegilops	geniculata	Roth	1340278	España	Burgos	Frías	30TVN7534	0	600		1997
Aegilops	geniculata	Roth	1340279	España	Burgos	Oña, hacia Pino de Bureb	30TVN6429	0	600		1997
Aegilops	geniculata	Roth	1340280	España	Burgos	Frías	30TVN7534	0	600		1997

Aegilops	geniculata	Roth	1340281	España	Burgos	Ranera	30TVN7331	0	750		1997
Aegilops	geniculata	Roth	1340282	España	Burgos	Frías	30TVN7534	0	550		1997
Aegilops	geniculata	Roth	1340283	España	Burgos	Frías	30TVN7534	0	530		1997
Aegilops	geniculata	Roth	1351576	España	Zaragoza	Chodes, Cantera de yeso	30TXL29	0	430		1997
Aegilops	geniculata	Roth	1354405	España	La Rioja	Viguera	30TWM4081	0	1100		1988
Aegilops	geniculata	Roth	1354558	España	León	Campohermoso	30TUN04	0			1988
Aegilops	geniculata	Roth	1354560	España	León	Aviados	30TUN04	0			1988
Aegilops	geniculata	Roth	1366002	España	Navarra	Castillo Nuevo, Barranco	30TXN5924	0	1000		1992
Aegilops	geniculata	Roth	1366004	España	Navarra	Lumbier, Biezcas	30TXN3820	0	440		1992
Aegilops	geniculata	Roth	1366006	España	Navarra	Lumbier, La Oquía	30TXN4021	0	600		1992
Aegilops	geniculata	Roth	1366008	España	Zaragoza	Salvatierra, Barranco de l	30TXN6224	0	600		1992
Aegilops	geniculata	Roth	1370918	España	Madrid	Soto del Real	30TVL3410	0			1997
Aegilops	geniculata	Roth	1370920	España	Madrid	San Agustín de Guadalix	30TVL4806	0			1997
Aegilops	geniculata	Roth	1370922	España	Madrid	Guadalix de la Sierra	30TVL4114	0			1997
Aegilops	geniculata	Roth	1370924	España	Madrid	Guadalix de la Sierra	30TVL4214	0			1997
Aegilops	geniculata	Roth	1370926	España	Madrid	El Molar	30TVL5008	0			1997
Aegilops	geniculata	Roth	1370928	España	Madrid	Redueña	30TVL5016	0			1997
Aegilops	geniculata	Roth	1373118	España	Albacete	Villapalacios, barranco de	30SWH2472	0	900		1994
Aegilops	geniculata	Roth.	1376732	España	Granada	Sierra de Baza, Parque Na	30SWG13	1			1991
Aegilops	geniculata	Roth	1377175	España	Orense	Vilardesilva	29TPH7903	0	450		1996
Aegilops	geniculata	Roth	1463961	España	Lérida	Pla d'Albelda	31TBG93	0	440		2001
Aegilops	geniculata	Roth	1463962	España	Lérida	Pla de les Gesses	31TBG93	0	320		2001
Aegilops	geniculata	Roth	1463963	España	Lérida	Montferrús de Camporre	31TBG94	0	680		2001
Aegilops	geniculata	Roth	1463964	España	Lérida	Penya Roja de Valldellou	31TCG04	0	420		2001
Aegilops	geniculata	Roth	1463965	España	Lérida	Gerb	31TCG13	0	230		2001
Aegilops	geniculata	Roth	1463966	España	Lérida	La Garriga de Gerb	31TCG13	0	350		2001
Aegilops	geniculata	Roth	1463967	España	Lérida	Les Avellanes	31TCG14	0	600		2001
Aegilops	geniculata	Roth	1463968	España	Lérida	Les Avellanes, monestir	31TCG14	0	580		2001
Aegilops	geniculata	Roth	1463969	España	Lérida	Vilanova de la Sal, salines	31TCG14	0	550		2001
Aegilops	geniculata	Roth	1463970	España	Lérida	Tartareu	31TCG14	0	600		2001
Aegilops	geniculata	Roth	1463971	España	Lérida	Àger, riu Fred	31TCG15	0	650		2001
Aegilops	geniculata	Roth	1463972	España	Lérida	Montroig	31TCG23	0	400		2001
Aegilops	geniculata	Roth	1463973	España	Lérida	Serra de Monteró	31TCG23	0	360		2001
Aegilops	geniculata	Roth	1463974	España	Lérida	Valldarnàs, entre Cubells	31TCG23	0	340		2001
Aegilops	geniculata	Roth	1463975	España	Lérida	Fontllonga	31TCG24	0	650		2001
Aegilops	geniculata	Roth	1463976	España	Lérida	Sant Linya, vers l'estació	31TCG24	0	560		2001
Aegilops	geniculata	Roth	1463977	España	Lérida	Peralba	31TCG25	0	780		2001
Aegilops	geniculata	Roth	1463978	España	Lérida	Serra d'Arquells	31TCG33	0	500		2001
Aegilops	geniculata	Roth	1463979	España	Lérida	Alòs de Balaguer	31TCG34	0	300		2001

Aegilops	geniculata	Roth	1463980	España	Lérida	Gàrzola	31TCG34	0	540		2001
Aegilops	geniculata	Roth	1463981	España	Lérida	Puig de Sant Ermengol	31TCG34	0	460		2001
Aegilops	geniculata	Roth	1463982	España	Lérida	Santa Maria de Meià	31TCG35	0	630		2001
Aegilops	geniculata	Roth.	1475536	España	Murcia	Sierra del Ricote	30SXH32	0			1985
Aegilops	geniculata	Roth.	1476046	España	Alicante	Sistemas de dunas litoral	30SYH00	0			1985
Aegilops	geniculata	Roth.	1476047	España	Murcia	Sistemas de dunas litoral	30SXG86	0			1985
Aegilops	geniculata	Roth	1481198	España	Islas Baleares	P.N. Archipiélago de Cab	31SDD93	1			1997
Aegilops	geniculata	Roth	1487853	España	Granada	P.N. Sierra Nevada	30SVG70	1			1987
Aegilops	geniculata	Roth	1489076	España	Ciudad Real	P.N. Tablas de Daimiel	30SVJ43	1			1994
Aegilops	ovata		1531728	España	Cantabria	camino de regreso de Qu	30TVN04	1			1953
Aegilops	ovata	L.	1531939	España	Cantabria	Mataporquera	30TVN04	1			1953
Aegilops	ovata	L.	1531949	España	Burgos	Mena	30TVN77	1			1953
Aegilops	ovata		1535538	España	Cantabria	parte occidental de Mata	30TVN04	1			1953
Aegilops	ovata	L.	1539227	España	León	pte. de Castro	30TTN91	1			1917
Aegilops	ovata		1549353	España	La Coruña	Gallec. (comarca del Ferr	29TNJ61	1			1861
Aegilops	ovata		1549487	España	Burgos	utraque Cast. (Burgos)	30TVM48	1			1861
Aegilops	ovata		1549491	España	León	regno Legion. (Villafranca	29TPH71	1			1861
Aegilops	ovata		1549511	España	Vizcaya	Cantabr. (Durango)	30TWN37	1			1861
Aegilops	ovata	L.	1551967	España	Burgos	Aranda	30TVM41	1			1917
Aegilops	ovata	L.	1554537	España	Burgos	Pancorbo	30TVN92	1			1917
Aegilops	ovata	L.	1554580	España	Palencia	Cervera	30TUN74	1			1917
Aegilops	ovata	L.	1554585	España	Burgos	Briviesca	30TVM71	1			1917
Aegilops	ovata	L.	1554599	España	León	Peña Corada	30TUN34	1			1917
Aegilops	ovata	L.	1554636	España	León	León	30TTN81	1			1917
Aegilops	geniculata		1558107	España	Palencia	Soto de Cerrato	30TUM7549	0	840		1983
Aegilops	geniculata		1558123	España	Palencia	Reinoso de Cerrato	30TUM8943	0	850		1983
Aegilops	geniculata		1564672	España	Palencia	Hornillo de Cerrato	30TUM9052	0	830		1983
Aegilops	geniculata	Roth.	1564722	España	Palencia	Soto de Cerrato	30TUM7549	0			1983
Aegilops	ovata		1564847	España	Palencia	Soto de Cerrato	30TUM7549	0	830		1983
Aegilops	geniculata		1564979	España	Palencia	Soto de Cerrato	30TUM7549	0	810		1983
Aegilops	ovata		1565147	España	Palencia	Hornillo de Cerrato	30TUM9052	0	850		1983
Aegilops	geniculata	Roth	1573681	España	Palencia	proximidades de Celada	30TUN85	0			1989
Aegilops	geniculata		1578713	España	Palencia	Ruesga	30TUN75	0			1990
Aegilops	geniculata	Roth.	1593210	España	Palencia	Muñeca	30TUN5239	0			1990
Aegilops	geniculata	Roth.	1593211	España	Palencia	Guardo	30TUN4939	0			1990
Aegilops	geniculata		1593497	España	Palencia	Muñeca	30TUN53	0	1180		1990
Aegilops	geniculata		1593500	España	Palencia	Guardo	30TUN43	0	1120		1990
Aegilops	geniculata	Roth	1597203	España	León	Monte del Duque	30TTM96	0			1988
Aegilops	geniculata		1600822	España	León	Valderas, Monte del Duq	30TTM96	0	760		1988

Aegilops	ovata	L.	1618204	España	León	Mora de Luna	30TTN64	0			1983
Aegilops	geniculata	Roth	1620398	España	León	Miñera de Luna	30TTN64	1			1983
Aegilops	ovata	L.	1625896	España	Palencia	Partido Farmacéutico de	30TUM78	1			1913
Aegilops	geniculata	Roth	1643496	España	Zaragoza	El Castellar	30TXM7041	0	540		2003
Aegilops	geniculata	Roth	1643497	España	Zaragoza	El Castellar	30TXM7043	0	660		2003
Aegilops	geniculata	Roth	1643498	España	Zaragoza	Juslibol	30TXM7518	0	230		2003
Aegilops	geniculata	Roth	1643499	España	Zaragoza	Peñaflor, Los Rasos	30TXM9127	0	360		2003
Aegilops	geniculata	Roth	1643500	España	Zaragoza	Peñaflor, San Cristóbal	30TXM8626	0	300		2003
Aegilops	geniculata	Roth	1643501	España	Zaragoza	Alfocea	30TXM6921	0	280		2003
Aegilops	geniculata	Roth	1643502	España	Zaragoza	Villamayor, Cuesta de Sa	30TXM8718	0	300		2003
Aegilops	geniculata	Roth	1643503	España	Zaragoza	Mediana, Llanos de la Sa	30TXL8898	0	370		2003
Aegilops	geniculata	Roth	1643504	España	Zaragoza	Plana de Zaragoza, Acam	30TXL7298	0	600		2003
Aegilops	geniculata		1653412	España	Albacete	Fuentealbilla, hacia Aben	30SXJ24	1			2003
Aegilops	geniculata		1674401	España	Toledo	Cazalegas	30TUK5630	0	440		2004
Aegilops	ovata	L.	1676972	España	Islas Baleares	Archipiélago de Cabrera,	31SDD93	1		CB	1975
Aegilops	ovata	L.	1676973	España	Islas Baleares	Archipiélago de Cabrera,	31SDD93	1		CB	1975
Aegilops	ovata	L.	1684419	España	Cádiz	Márgenes Arroyo El Desc	30STF8071	0			1978
Aegilops	ovata	L.	1684420	España	Cádiz	Los Espartales, junto a ch	30STF8975	0			1978
Aegilops	ovata	L.	1684421	España	Cádiz	El Bosque, junto campo d	30STF7671	0			1978
Aegilops	ovata	L.	1684447	España	Cádiz	Cuneta carretera de Ubri	30STF7864	0			1978
Aegilops	geniculata	Roth	1696519	España	Teruel	Iglesuela del Cid, pista ar	30TYK2582	0	1500		2002
Aegilops	geniculata	Roth	1696520	España	Teruel	Cantavieja, pista a Tarayu	30TYK1988	0	1500		2002
Aegilops	geniculata	Roth	1696521	España	Teruel	Cantavieja, mas de Porca	30TYK2385	0	1500		2002
Aegilops	geniculata	Roth	1696522	España	Teruel	Puertomingalvo, El Letra	30TYK1368	0	1500		2002
Aegilops	geniculata	Roth	1696523	España	Teruel	Iglesuela del Cid, mas de	30TYK2786	0	1500		2002
Aegilops	geniculata	Roth	1696524	España	Teruel	Iglesuela del Cid, torre Ni	30TYK3084	0	1500		2002
Aegilops	geniculata	Roth	1696525	España	Teruel	Iglesuela del Cid, la Pobla	30TYK2883	0	1500		2002
Aegilops	geniculata	Roth	1696526	España	Teruel	Puertomingalvo, pista de	30TYK1468	0	1500		2002
Aegilops	geniculata	Roth	1696527	España	Teruel	Cantavieja	30TYK1987	0	1500		2002
Aegilops	geniculata	Roth	1696528	España	Teruel	Iglesuela del Cid, pista ar	30TYK2581	0	1500		2002
Aegilops	geniculata		1699512	España	Teruel	Mosqueruela	30TYK1570	0	1480		2002
Aegilops	geniculata		1699513	España	Teruel	Mosqueruela, La Barraca	30TYK2974	0	1100		2002
Aegilops	geniculata		1700269	España	Teruel	Vilafranca, Masía de la Ra	30TYK3084	0	1140		2002
Aegilops	geniculata		1700270	España	Teruel	Iglesuela del Cid, Masía d	30TYK2383	0	1300		2002
Aegilops	geniculata		1700449	España	Teruel	Cantavieja, Masía de Por	30TYK2385	0	1400		2002
Aegilops	geniculata		1700450	España	Teruel	Puertomingalvo, El Letra	30TYK1368	0	1525		2002
Aegilops	geniculata		1700451	España	Teruel	Cantavieja	30TYK1987	0	1400		2002
Aegilops	geniculata		1700452	España	Teruel	Puertomingalvo, inicio pi	30TYK1468	0	1500		2002
Aegilops	geniculata		1700453	España	Teruel	Iglesuela del Cid, Masía d	30TYK2786	0	1320		2002

Aegilops	geniculata		1700454	España	Teruel	Cantavieja, pista a Tarayu	30TYK1988	0	1380		2002
Aegilops	geniculata		1700455	España	Teruel	Iglesuela del Cid,Torre de	30TYK3084	0	1140		2002
Aegilops	geniculata		1700456	España	Teruel	Iglesuela del Cid, la Pobla	30TYK2883	0	1115		2002
Aegilops	geniculata		1701104	España	Teruel	Mosqueruela, Masico Bie	30TYK1673	0	1560		2002
Aegilops	geniculata		1701105	España	Teruel	Iglesuela del Cid, Pobla d	30TYK2883	0	1120		2002
Aegilops	geniculata		1701106	España	Teruel	Mosqueruela, Cuartel-Ba	30TYK1670	0	1420		2002
Aegilops	geniculata		1701107	España	Teruel	Vilafranca, Masía de la Ra	30TYK3084	0	1140		2002
Aegilops	geniculata		1713416	España	Islas Baleares	Mallorca, Calvià, finca Se	31SDD58	1	110		2002
Aegilops	geniculata		1713441	España	Islas Baleares	Mallorca, Calvià, finca Se	31SDD58	1	120		2002
Aegilops	geniculata	Roth.	1715001	España	Cáceres	Sierra las Corchuelas, Par	30STK51	1			1986
Aegilops	geniculata		1715682	España	Gerona	Siurana, entre Brava i Bas	31TEG0072	0			2006
Aegilops	geniculata		1715683	España	Gerona	Vilanova de la Muga, a to	31TEG0281	0			2006
Aegilops	geniculata		1715684	España	Gerona	Peraleda, marge del cam	31TEG0581	0			2006
Aegilops	geniculata		1715685	España	Gerona	Peraleda, regall de la pist	31TEG0382	0			2006
Aegilops	geniculata	Roth	1715686	España	Gerona	Aiguamolls de l'Empurdá	31TEG06	0			2006
Aegilops	geniculata		1715687	España	Gerona	Ventalló, entre el puig d'l	31TEG0466	0			2006
Aegilops	geniculata	Roth	1715688	España	Gerona	Aiguamolls de l'Empurdá	31TEG07	0			2006
Aegilops	geniculata	Roth	1715689	España	Gerona	Peraleda, prop a Mas Bar	31TEG0582	0			2006
Aegilops	geniculata	Roth	1715690	España	Gerona	Peraleda, Mas Barrera	31TEG08	0			2006
Aegilops	geniculata	Roth	1715691	España	Gerona	Aiguamolls de l'Empurdá	31TEG16	0			2006
Aegilops	geniculata		1762687	España	Ciudad Real	Morrón de Villamayor	30SVH09	0			2004
Aegilops	geniculata		1762688	España	Ciudad Real	Volcán de Yezosa	30SVJ40	0			2004
Aegilops	geniculata		1762689	España	Ciudad Real	Volcán de Piedrabuena	30SUJ92	0			2004
Aegilops	geniculata	Roth	1774958	España	Huesca	Monzón	31TBG6844	0	320		2004
Aegilops	geniculata	Roth	1774959	España	Huesca	Monzón, cerro de Santa (	31TBG6743	0	365		2004
Aegilops	geniculata	Roth	1774960	España	Huesca	Almunia de San Juan	31TBG7146	0	370		2004
Aegilops	geniculata	Roth	1774961	España	Huesca	Monzón, camino del Vall	31TBG6843	0	290		2004
Aegilops	geniculata		1811863	España	Navarra	Arguedas, carretera a Tu	30TXM16	0			1986
Aegilops	geniculata		1811864	España	Navarra	Tudela, carretera a Ejea d	30TXM15	0			1986
Aegilops	geniculata		1811865	España	Navarra	Vedado de Eguaras	30TXM28	0	480		1986
Aegilops	geniculata		1811866	España	Navarra	Fitero	30TWM95	0	300		1986
Aegilops	geniculata		1811867	España	Navarra	Milagro, carretera a Cadr	30TXM07	0			1986
Aegilops	geniculata		1811868	España	Navarra	Valtierra	30TXM1371	0			1986
Aegilops	geniculata		1811869	España	Navarra	Sierra de San Pedro, a Ga	30TXN30	1			1989
Aegilops	geniculata		1811870	España	Navarra	Bardena Negra	30TXM27	1	400		1989
Aegilops	geniculata	Roth	1811871	España	Navarra	Monte Peña, Sierra de Sa	30TXN4007	0	900		1993
Aegilops	geniculata	Roth	1811872	España	Navarra	Bardena Blanca	30TXM2474	0			1986
Aegilops	geniculata	Roth	1811873	España	Navarra	Tudela	30TXM1658	0			1986
Aegilops	geniculata	Roth	1811874	España	Navarra	Loma Negra	30TXM3858	0			1986

Aegilops	geniculata	Roth	1811875	España	Navarra	Milagro	30TXM0278	0			1986
Aegilops	geniculata	Roth	1811876	España	Navarra	Loma Negra	30TXM3464	0			1986
Aegilops	geniculata	Roth.	1811877	España	Navarra	Castillonuevo, Barranco d	30TXN5924	0	1000		1992
Aegilops	geniculata	Roth.	1811878	España	Navarra	Lumbier, Biezcas	30TXN3820	0	440		1992
Aegilops	geniculata	Roth.	1811879	España	Navarra	Lumbier, La Oquía	30TXN4021	0	600		1992
Aegilops	geniculata	Roth.	1811880	España	Navarra	Salvatierra, Barranco de l	30TXN6224	0	600		1992
Aegilops	ovata		1811881	España	Huesca	Peña Forca Oriental, Río S	30TXN85	1	1000		1982
Aegilops	ovata	L.	1834167	España	Melilla	Melilla, pinares de Rostro	30SWE00	0			2003
Aegilops	geniculata	Roth	1835070	España	Ciudad Real	Despeñaperros	30SVH55	1		CB	2005
Aegilops	geniculata	Roth	1835071	España	Ciudad Real	Herencia, cerro Navajo, la	30SVJ6551	0	660	CB	2005
Aegilops	geniculata	Roth	1835072	España	Ciudad Real	Herencia, de Herencia a V	30SVJ6853	0	700	CB	2005
Aegilops	geniculata	Roth	1835073	España	Ciudad Real	La Solana, sierra de Alhar	30SVJ90	1		CB	2005
Aegilops	geniculata	Roth	1835074	España	Ciudad Real	Moral de Calatrava, sierra	30SVH5100	0		CB	2005
Aegilops	geniculata	Roth	1835075	España	Ciudad Real	Malagón-Los Quiles, olivo	30SVJ2340	0		CB	2005
Aegilops	geniculata	Roth	1835076	España	Ciudad Real	Lagunas de Ruidera, bord	30SWJ01	1		CB	2005
Aegilops	geniculata	Roth	1835077	España	Ciudad Real	Piedrabuena, volcán de P	30SUJ9723	0	600	CB	2005
Aegilops	geniculata	Roth	1835080	España	Ciudad Real	Tablas de Daimiel, isla de	30SVJ43	1		CB	2005
Aegilops	geniculata	Roth	1835081	España	Ciudad Real	Torralba de Calatrava, Ca	30SVJ3031	0	610	CB	2005
Aegilops	geniculata	Roth	1835082	España	Ciudad Real	Puerto de la Caracollera	30SUH78	1		CB	2005
Aegilops	geniculata	Roth	1835083	España	Ciudad Real	Solana del Pino, alrededo	30SVH0459	0	800	CB	2005
Aegilops	geniculata	Roth	1835084	España	Ciudad Real	Villamayor de Calatrava,	30SVH0297	0	842	CB	2005
Aegilops	geniculata	Roth	1835085	España	Ciudad Real	Villanueva de San Carlos,	30SVH2672	0	600	CB	2005
Aegilops	geniculata	Roth	1835086	España	Ciudad Real	Villanueva de la Fuente	30SWH2582	0	1000	CB	2005
Aegilops	geniculata	Roth	1835088	España	Ciudad Real	Albadalejo	30SWH1674	0	900	CB	2005
Aegilops	geniculata	Roth	1835089	España	Ciudad Real	Alamillo, laderas del cerr	30SUH4678	0	600	CB	2005
Aegilops	geniculata	Roth	1835090	España	Ciudad Real	Alhambra, sierra de Alha	30SVJ9307	0		CB	2005
Aegilops	geniculata	Roth	1835091	España	Ciudad Real	Almagro, volcán de Yezos	30SVJ4200	0	853	CB	2005
Aegilops	geniculata	Roth	1869149	España	Ciudad Real	Ciudad Real, La Atalaya	30SVJ22	1	670		2004
Aegilops	geniculata		1886757	España	Valladolid	Renedo de Esgueva, zona	30TUM61	0			2006
Aegilops	geniculata	Roth	1888170	España	Valencia	Sierra del Caballón	30SYJ0052	0			2005
Aegilops	geniculata	Roth	1888171	España	Valencia	Sierra del Caballón	30SXJ9952	0			2005
Aegilops	geniculata	Roth	1889261	España	Alicante	Sierra de Orihuela	30SXH71	1			2001
Aegilops	geniculata	Roth	1891036	España	Islas Baleares	Formentera	31SCC78	0			2001
Aegilops	geniculata	Roth	1891037	España	Islas Baleares	Formentera	31SCC57	0			2001
Aegilops	geniculata	Roth	1891038	España	Islas Baleares	Formentera	31SCC58	0			2001
Aegilops	geniculata	Roth	1891039	España	Islas Baleares	Formentera	31SCC67	0			2001
Aegilops	geniculata	Roth	1891040	España	Islas Baleares	Formentera	31SCC77	0			2001
Aegilops	geniculata	Roth	1891041	España	Islas Baleares	Formentera	31SCC68	0			2001
Aegilops	ovata	L.	1893638	España	Tenerife	Tenerife, Igueste de Cand	28RCS63	1	500		1973

Aegilops	geniculata	Roth.	1895398	España	Burgos	Valle de Mena, Irus	30TVN6871	0	540		1993
Aegilops	geniculata	Roth	1909855	España	Navarra	Artajona, hacia Larraga, b	30TWN9715	0	330		1999
Aegilops	geniculata		1918074	España	Navarra	Artajona, hacia Larraga, b	30TWN9715	0	330		1999
Aegilops	geniculata	Roth	1925485	España	Navarra	Izal	30TXN5242	0	1105		2001
Aegilops	geniculata	Roth	1925486	España	Navarra	Lumbier, Foz de Lumbier	30TXN3822	0	420		2001
Aegilops	geniculata	Roth	1925487	España	Navarra	Yesa	30TXN4920	0	540		2001
Aegilops	geniculata	Roth	1925488	España	Navarra	Elcoaz, Collado Borrokosk	30TXN4546	0	1000		2001
Aegilops	geniculata	Roth	1925489	España	Navarra	Itoiz	30TXN3442	0	510		2001
Aegilops	geniculata	Roth	1925490	España	Navarra	Imízcoz	30TXN3352	0	820		2001
Aegilops	geniculata		1930605	España	Islas Baleares	Menorca, Macarella, calv	31SEE72	1			1988
Aegilops	geniculata		1930606	España	Islas Baleares	Menorca, Llucalari	31SEE91	1			1988
Aegilops	geniculata		1931162	España	Islas Baleares	Mallorca, Garrigas de Sor	31SED09	0			1988
Aegilops	geniculata		1931466	España	Islas Baleares	Menorca, Cerca de Maca	31SEE72	1			1988
Aegilops	geniculata		1972307	España	Huesca	Valle de Escuaín, Puértola	31TBH6023	0	1980		2008
Aegilops	geniculata		1972308	España	Huesca	Valle de Escuaín, Puértola	31TBH6023	0	2020		2008
Aegilops	geniculata		1972309	España	Huesca	Valle de Escuaín, Puértola	31TBH6023	0	2010		2008
Aegilops	geniculata		2064108	España	Alicante	Ibi, término municipal	30SYH17	0			2005
Aegilops	geniculata	Roth	2074534	España	Cuenca	Barajas de Melo	30TWK04	0			2001
Aegilops	ovata		2076447	España	Zaragoza	Sierra de Vicort, alrededo	30TXL18	1			1953
Aegilops	ovata	G. G.	2078524	España	Islas Baleares	Cabrera	31SDD93	1			1955
Aegilops	ovata	L.	2079076	España	Palencia	Castejón, a Villanueva de	30TUN64	1			1956
Aegilops	ovata	L.	2081930	España	Granada	Puebla de Don Fadrique,	30SWH40	1	1200		1956
Aegilops	ovata		2084586	España	Teruel	Alfambra a Cuevas Labra	30TXK69	1	1050		1957
Aegilops	ovata	L.	2086274	España	Toledo	Añover de Tajo, cerca car	30SVK32	1			1959
Aegilops	ovata	L.	2086275	España	Madrid	Cerro Negro	30TVK47	1			1959
Aegilops	ovata	L.	2086276	España	Madrid	Alcalá de Henares	30TVK68	1			1959
Aegilops	ovata	L.	2088035	España	Zaragoza	Castilíscar, a 4 km, Novel	30TXM49	1			1961
Aegilops	ovata		2088567	España	Zaragoza	Castilíscar, junto carreter	30TXN40	1	500		1961
Aegilops	ovata		2089201	España	Lérida	Figuerola d'Orcau	31TCG36	1	560		1961
Aegilops	ovata	L.	2092337	España	Teruel	Sierra de Corbalán	30TXK77	1			1961
Aegilops	ovata	L.	2092338	España	Teruel	Valdelinares	30TXK97	1			1961
Aegilops	ovata	L.	2092339	España	Teruel	Linares de Mora, Cerro B	30TYK06	1			1961
Aegilops	ovata	L.	2092512	España	Teruel	Linares, a Valdelinares	30TYK07	1			1961
Aegilops	ovata	L.	2092513	España	Teruel	Linares de Mora	30TYK06	1			1961
Aegilops	ovata	L.	2092514	España	Teruel	Albentosa, altiplano de S	30TXK84	1			1961
Aegilops	ovata		2102100	España	Guadalajara	Atienza	30TWL16	1	1100		1969
Aegilops	geniculata	Roth	2109955	España	Tarragona	Delta de l'Ebre	31TBF90	0			2007
Aegilops	geniculata	Roth	2144252	España	Valladolid	Renedo de Esgueva	30TUM6310	0	780		2006
Aegilops	geniculata	Roth	2144263	España	Valladolid	Renedo de Esgueva	30TUM6511	0	790		2006

Aegilops	geniculata	Roth	2144265	España	Valladolid	Renedo de Esgueva	30TUM6611	0	850		2006
Aegilops	geniculata	Roth	2144273	España	Valladolid	Renedo de Esgueva	30TUM6612	0	850		2006
Aegilops	geniculata	Roth.	2147307	España	Alicante	Petrer, Loma Badá	30SXH9458	0	450		2003
Aegilops	geniculata	Roth.	2147308	España	Alicante	Petrer, Batech	30SXH9560	0	550		2003
Aegilops	geniculata	Roth.	2147309	España	Alicante	Petrer, Batech	30SXH9560	0	520		2003
Aegilops	geniculata	Roth.	2147310	España	Alicante	Petrer, Batech	30SXH9560	0	560		2003
Aegilops	geniculata	Roth.	2147311	España	Alicante	Petrer, subida a la Silla de	30SXH9561	0	530		2003
Aegilops	geniculata	Roth.	2147312	España	Alicante	Sierra del Cid	30SXH9561	0			2003
Aegilops	geniculata	Roth.	2147313	España	Alicante	Petrer, Rambla de los Mo	30SXH9562	0	520		2003
Aegilops	geniculata	Roth.	2147315	España	Alicante	Sierra del Cid	30SXH9863	0			2003
Aegilops	geniculata	Roth.	2147317	España	Alicante	Petrer, Sierra del Cid, La	30SXH9863	0	640		2003
Aegilops	geniculata		2156933	España	Albacete	Villa de Ves, Sierra del Bo	30SXJ5138	0	810		2008
Aegilops	geniculata		2157153	España	Albacete	La Roda, de Fuensanta al	30SWJ8338	0	720		2008
Aegilops	geniculata		2160829	España	Albacete	Jorquera, hacia Alcalá de	30SXJ3137	0	550		2008
Aegilops	geniculata		2161239	España	Albacete	Casas de Juan Núñez, hac	30SXJ2635	0	690		2008
Aegilops	geniculata		2161291	España	Albacete	Bormate, hacia Mahora	30SXJ1838	0	650		2008
Aegilops	geniculata		2161982	España	Albacete	Alcalá del Júcar, Rambla	30SXJ3437	0	730		2008
Aegilops	geniculata		2162011	España	Albacete	Alcalá del Júcar, hacia Vil	30SXJ3332	0	740		2008
Aegilops	geniculata		2162022	España	Albacete	Albacete, cantera de Los	30SXJ0932	0	630		2008
Aegilops	geniculata		2162064	España	Albacete	Villa de Ves, Casa Sandur	30SXJ5140	0	570		2008
Aegilops	geniculata		2162080	España	Albacete	Alcalá del Júcar	30SXJ3638	0	720		2008
Aegilops	geniculata		2162086	España	Albacete	Casas de Ves, Barranco d	30SXJ4639	0	650		2008
Aegilops	geniculata		2162191	España	Albacete	Albacete, hacia El Encina	30SXJ0428	0	670		2008
Aegilops	geniculata		2162213	España	Albacete	Albacete, Los Yesares	30SXJ0732	0	700		2008
Aegilops	geniculata		2162246	España	Albacete	Fuensanta, Cerro del Gat	30SWJ8341	0	720		2008
Aegilops	geniculata		2162413	España	Albacete	Fuentealbilla, hacia Alcal	30SXJ2546	0	690		2008
Aegilops	geniculata		2164544	España	Albacete	Fuensanta, Cerro del Gat	30SWJ8340	0	730		2008
Aegilops	geniculata		2200249	España	Alicante	Provincia de Alicante	30SYH57	0			2007
Aegilops	geniculata		2200250	España	Alicante	Provincia de Alicante	30SYH28	0			2007
Aegilops	geniculata		2200251	España	Alicante	Provincia de Alicante	30SYH15	0			2007
Aegilops	geniculata		2200252	España	Alicante	Provincia de Alicante	30SYH17	0			2007
Aegilops	geniculata		2200253	España	Alicante	Provincia de Alicante	30SYH13	0			2007
Aegilops	geniculata		2200254	España	Alicante	Provincia de Alicante	30SYH18	0			2007
Aegilops	geniculata		2200255	España	Alicante	Provincia de Alicante	30SYH08	0			2007
Aegilops	geniculata		2200256	España	Alicante	Provincia de Alicante	30SYH19	0			2007
Aegilops	geniculata		2200257	España	Alicante	Provincia de Alicante	30SYH24	0			2007
Aegilops	geniculata		2200258	España	Alicante	Provincia de Alicante	30SYH25	0			2007
Aegilops	geniculata		2200259	España	Alicante	Provincia de Alicante	30SYH27	0			2007
Aegilops	geniculata		2200260	España	Alicante	Provincia de Alicante	30SYH14	0			2007

Aegilops	geniculata		2200261	España	Alicante	Provincia de Alicante	30SYH29	0			2007
Aegilops	geniculata		2200262	España	Alicante	Provincia de Alicante	30SYH37	0			2007
Aegilops	geniculata		2200263	España	Alicante	Provincia de Alicante	30SYH38	0			2007
Aegilops	geniculata		2200264	España	Alicante	Provincia de Alicante	30SYH39	0			2007
Aegilops	geniculata		2200265	España	Alicante	Provincia de Alicante	30SYH47	0			2007
Aegilops	geniculata		2200266	España	Alicante	Provincia de Alicante	30SYH49	0			2007
Aegilops	geniculata		2200267	España	Alicante	Provincia de Alicante	30SYJ50	0			2007
Aegilops	geniculata		2200268	España	Alicante	Provincia de Alicante	30SYH59	0			2007
Aegilops	geniculata		2200269	España	Alicante	Provincia de Alicante	30SYJ40	0			2007
Aegilops	geniculata		2200270	España	Alicante	Provincia de Alicante	30SYH58	0			2007
Aegilops	geniculata		2200271	España	Alicante	Provincia de Alicante	30SYH07	0			2007
Aegilops	geniculata		2200272	España	Alicante	Provincia de Alicante	30SYH48	0			2007
Aegilops	geniculata		2200273	España	Alicante	Provincia de Alicante	30SXH79	0			2007
Aegilops	geniculata		2200274	España	Alicante	Provincia de Alicante	30SYH06	0			2007
Aegilops	geniculata		2200275	España	Alicante	Provincia de Alicante	30SYJ30	0			2007
Aegilops	geniculata		2200276	España	Alicante	Provincia de Alicante	31SBC48	0			2007
Aegilops	geniculata		2200277	España	Alicante	Provincia de Alicante	31SBC49	0			2007
Aegilops	geniculata		2200278	España	Alicante	Provincia de Alicante	31SBC59	0			2007
Aegilops	geniculata		2200279	España	Alicante	Provincia de Alicante	31SBD50	0			2007
Aegilops	geniculata		2200280	España	Alicante	Provincia de Alicante	30SXH75	0			2007
Aegilops	geniculata		2200281	España	Alicante	Provincia de Alicante	30SXH81	0			2007
Aegilops	geniculata		2200282	España	Alicante	Provincia de Alicante	30SXH83	0			2007
Aegilops	geniculata		2200283	España	Alicante	Provincia de Alicante	30SXH84	0			2007
Aegilops	geniculata		2200284	España	Alicante	Provincia de Alicante	30SXH85	0			2007
Aegilops	geniculata		2200285	España	Alicante	Provincia de Alicante	30SYH02	0			2007
Aegilops	geniculata		2200286	España	Alicante	Provincia de Alicante	30SXH71	0			2007
Aegilops	geniculata		2200287	España	Alicante	Provincia de Alicante	30SYH03	0			2007
Aegilops	geniculata		2200288	España	Alicante	Provincia de Alicante	30SXH87	0			2007
Aegilops	geniculata		2200289	España	Alicante	Provincia de Alicante	30SYH00	0			2007
Aegilops	geniculata		2200290	España	Alicante	Provincia de Alicante	30SXH98	0			2007
Aegilops	geniculata		2200291	España	Alicante	Provincia de Alicante	30SXH97	0			2007
Aegilops	geniculata		2200292	España	Alicante	Provincia de Alicante	30SXH96	0			2007
Aegilops	geniculata		2200293	España	Alicante	Provincia de Alicante	30SXH95	0			2007
Aegilops	geniculata		2200294	España	Alicante	Provincia de Alicante	30SXH94	0			2007
Aegilops	geniculata		2200295	España	Alicante	Provincia de Alicante	30SXH89	0			2007
Aegilops	geniculata		2200296	España	Alicante	Provincia de Alicante	30SXH88	0			2007
Aegilops	geniculata		2200297	España	Alicante	Provincia de Alicante	30SYH05	0			2007
Aegilops	geniculata		2242469	España	Málaga	Ronda, proximidades de	30SUF15	1	1150		1998
Aegilops	geniculata		2242470	España	Málaga	Ronda, camino del Puert	30SUF06	1	1120		1998

Aegilops	geniculata		2242471	España	Cáceres	La Nava, Berzocana, Colla	30STJ8464	0	750		2008
Aegilops	geniculata		2242472	España	Cáceres	Berzocana	30STJ8968	0	650		2008
Aegilops	geniculata		2242473	España	Cáceres	La Nava, Berzocana	30STJ8664	0	645-783		2008
Aegilops	geniculata		2242474	España	Cáceres	Logrosán, Minas de Sierra	30STJ8556	0	410-650		2008
Aegilops	geniculata		2242475	España	Cáceres	La Nava, Berzocana, Colla	30STJ8464	0	700-890		2008
Aegilops	geniculata		2242476	España	Cáceres	Los Lotes, Cañamero	30STJ9259	0	500		2008
Aegilops	geniculata		2242477	España	Cáceres	La Nava, Berzocana	30STJ8664	0	760-780		2008
Aegilops	geniculata		2242478	España	Cáceres	Logrosán, Dehesa Boyal	30STJ8655	0	490		2008
Aegilops	geniculata		2242479	España	Cáceres	La Nava, Berzocana	30STJ8664	0	640		2008
Aegilops	geniculata		2242480	España	Cádiz	Alcalá de los Gazules, Los	30STF64	1			1997
Aegilops	geniculata		2242481	España	Cádiz	Alcalá de los Gazules, km	30STF64	1	100		1999
Aegilops	geniculata		2242482	España	Jaén	Torreperogil	30SVH70	1	790		2006
Aegilops	geniculata		2242483	España	Cádiz	Alcalá de los Gazules, La	30STF62	1	100		1999
Aegilops	geniculata		2242484	España	Jaén	Los Villares, urbanización	30SVG26	1	1240		2000
Aegilops	geniculata		2242485	España	Cádiz	Alcalá de los Gazules	30STF53	1			1997
Aegilops	geniculata		2242486	España	Jaén	Jaén, Polígono Llanos del	30SVG38	1	520		2006
Aegilops	geniculata		2242487	España	Cádiz	Los Barrios, Cortijo de Oj	30STF60	1			1997
Aegilops	geniculata		2242488	España	Cádiz	Alcalá de los Gazules, La J	30STF55	1			1997
Aegilops	geniculata		2242489	España	Jaén	Jimena	30SVG58	1	640		2006
Aegilops	geniculata		2242490	España	Jaén	Los Villares, carretera sub	30SVG26	1	1050-1180		2000
Aegilops	geniculata		2242491	España	Jaén	Los Villares, Puerto La Ol	30SVG27	1	1360		2000
Aegilops	geniculata		2242492	España	Cádiz	Jerez de la Frontera, Casa	29SQA56	1			1997
Aegilops	geniculata		2242493	España	Cádiz	Tarifa	30STE69	1			1997
Aegilops	geniculata		2242494	España	Cádiz	Alcalá de los Gazules, Veg	30STF53	1			1997
Aegilops	geniculata	Roth	2242495	España	Málaga	Alcaucín	30SVF0185	0			2005
Aegilops	geniculata	Roth	2242496	España	Málaga	Canillas de Aceituno	30SVF0383	0			2005
Aegilops	geniculata	Roth	2242497	España	Málaga	Nerja	30SVF26	0			2005
Aegilops	geniculata	Roth	2242498	España	Granada	Arenas del Rey	30SVF2375	0			2005
Aegilops	geniculata	Roth	2242499	España	Málaga	Nerja	30SVF2971	0			2005
Aegilops	geniculata	Roth	2242500	España	Granada	Alhama de Granada	30SVF0890	0			2005
Aegilops	geniculata	Roth	2242501	España	Málaga	Ronda	30SUF07	1			2007
Aegilops	ovata		2242505	España	Cáceres	Los Cercones, Logrosán	30STJ8658	0	430-440		2008
Aegilops	ovata		2242506	España	Cáceres	Logrosán, Estación Ferro	30STJ8657	0	450		2008
Aegilops	geniculata	Roth	2274104	España	Sevilla	Montellano, Sierra de Mo	30STG70	0			1981
Aegilops	geniculata		2276471	España	Valladolid	Quintanilla de Trigueros	30TUM6237	0	850		2009
Aegilops	geniculata		2276472	España	Valladolid	Cigales	30TUM5528	0	850		2009
Aegilops	geniculata		2276473	España	Valladolid	Quintanilla de Trigueros	30TUM6238	0	850		2009
Aegilops	geniculata		2276474	España	Valladolid	Cigales	30TUM5528	0	850		2009
Aegilops	geniculata		2287376	España	Cuenca	Enguídanos, laderas de La	30SXJ2195	0	685		2009

Aegilops	geniculata		2287377	España	Cuenca	Enguídanos, laderas de L	30SXJ2194	0	680		2009
Aegilops	geniculata		2287378	España	Cuenca	Enguídanos, Las Quebrad	30SXJ2291	0	670-680		2009
Aegilops	geniculata	Roth	2309217	España	Córdoba	Fuenteovejuna, compren	30STH83	1			1986
Aegilops	geniculata	Roth	2309218	España	Córdoba	Valle del Guadalquivir, zo	30SUG08	1			1986
Aegilops	geniculata	Roth	2309219	España	Córdoba	Valle del Guadalquivir, pa	30SUG18	1			1986
Aegilops	geniculata	Roth	2309220	España	Córdoba	La Carlota, poblaciones d	30SUG27	1			1986
Aegilops	geniculata	Roth	2309221	España	Córdoba	Puente Genil	30SUG43	1			1986
Aegilops	geniculata	Roth	2309222	España	Córdoba	Valle del Guadalquivir, de	30SUG59	1			1986
Aegilops	geniculata	Roth	2309223	España	Córdoba	Campiña de Moriles Luce	30SUG64	1			1986
Aegilops	geniculata	Roth	2309224	España	Córdoba	Campiña de Baena, abarc	30SUG86	1			1986
Aegilops	geniculata	Roth	2309225	España	Córdoba	Subbético, comprende C	30SUG95	1			1986
Aegilops	geniculata	Roth	2309226	España	Córdoba	Hinojosa del Duque, part	30SUH16	1			1986
Aegilops	ovata	L.	2364326	España	La Coruña	Gallec., comarca del Ferr	29TNJ61	1			1861-1862
Aegilops	ovata	L.	2364329	España	Vizcaya	Cantabr., Durango	30TWN27	1			1861-1862
Aegilops	ovata	L.	2364330	España	Zaragoza	Aragon., Saragossae	30TXM71	1			1861-1862
Aegilops	ovata	L.	2364331	España	Teruel	Aragon., Calamocha	30TXL43	1			1861-1862
Aegilops	ovata	L.	2364332	España	Navarra	Navarra, Olave	30TXN14	1			1861-1862
Aegilops	ovata	L.	2364333	España	Valladolid	utraque Cast., Valladolid	30TUM51	1			1861-1862
Aegilops	ovata	L.	2364334	España	Burgos	utraque Cast., Burgos	30TVM48	1			1861-1862
Aegilops	ovata	L.	2364335	España	Madrid	utraque Cast., Guadarran	30TVL00	1			1861-1862
Aegilops	ovata	L.	2364336	España	Madrid	utraque Cast., Aranjuez	30TVK43	1			1861-1862
Aegilops	ovata	L.	2364337	España	Madrid	utraque Cast., Madrid	30TVK47	1			1861-1862
Aegilops	ovata	L.	2364403	España	León	regno Legion., Villafranca	29TPH71	1			1861-1862
Aegilops	ovata		2406816	España	Cádiz	Benaocaz	30STF86	1			1941
Aegilops	ovata		2406817	España	Sevilla	Morón	30STG81	1			1941
Aegilops	ovata		2406818	España	Cádiz	Puerto de Santa María	29SQA45	1			1941
Aegilops	ovata		2406819	España	Málaga	Sierra Blanquilla-Junquer	30SUF26	1			1941
Aegilops	ovata		2406820	España	Madrid	El Pardo	30TVK38	1			1941
Aegilops	ovata		2406936	España	Cuenca	Hoz de Beteta	30TWK79	1			1942
Aegilops	ovata	L.	2425008	España	Orense	Rubiana, por encima de V	29TPH70	1	750		1967
Aegilops	geniculata	Roth.	2442796	España	Guadalajara	Córcoles	30TWK28	0			1984
Aegilops	geniculata		2442798	España	Guadalajara	Pareja	30TWK28	0	800		1984
Aegilops	geniculata		2442799	España	Guadalajara	Pareja	30TWK28	0	780		1984
Aegilops	geniculata		2442800	España	Guadalajara	Pareja	30TWK28	0	780		1984
Aegilops	geniculata		2442801	España	Guadalajara	Alcocer	30TWK38	0	800		1984
Aegilops	geniculata		2442802	España	Guadalajara	Alique	30TWK39	0	920		1984
Aegilops	geniculata		2442803	España	Guadalajara	Pareja	30TWK28	0	750		1984
Aegilops	geniculata		2442804	España	Guadalajara	Pareja	30TWK28	0	750		1984
Aegilops	geniculata		2442805	España	Guadalajara	Villanueva de Alcorón	30TWL60	0	1200		1984

Aegilops	geniculata		2442806	España	Guadalajara	Chillarón del Rey	30TWK29	1	780		1984
Aegilops	geniculata	Roth.	2442807	España	Guadalajara	Alcocer	30TWK38	0			1984
Aegilops	geniculata	Roth.	2442808	España	Guadalajara	Millana	30TWK38	0			1984
Aegilops	geniculata		2442809	España	Guadalajara	Alcocer	30TWK38	0	820		1984
Aegilops	geniculata		2442810	España	Guadalajara	La Puerta	30TWK39	0	800		1984
Aegilops	geniculata	Roth.	2442811	España	Guadalajara	Peralveche	30TWK49	0			1984
Aegilops	geniculata	Roth.	2442812	España	Guadalajara	El Recuenco	30TWK59	0			1984
Aegilops	geniculata		2442813	España	Guadalajara	Villanueva de Alcorón	30TWL60	0	1260		1984
Aegilops	geniculata		2442815	España	Guadalajara	Alcocer	30TWK38	0	810		1984
Aegilops	geniculata		2442816	España	Guadalajara	Alique	30TWK39	0	930		1984
Aegilops	geniculata	Roth.	2442817	España	Guadalajara	Salmerón	30TWK48	0			1984
Aegilops	ovata	L.	2466925	España	Tenerife	Tenerife. Tégueste	28RCS65	1			1908
Aegilops	ovata	L.	2474587	España	Tenerife	Tenerife. In valle Teguest	28RCS65	1			1844-1850
Aegilops	geniculata		2488825	España	Jaén	Sierra de Segura, Los Are	30SWG1496	0	1400		2006
Aegilops	geniculata	Roth	2513818	España	Burgos	Quintanilla del Coco	30TVM5449	0	1007		2006
Aegilops	geniculata	Roth	2513819	España	Burgos	Santibáñez del Val	30TVM5846	0	986		2006
Aegilops	geniculata	Roth	2513820	España	Burgos	Santo Domingo de Silos	30TVM6741	0	1240		2006
Aegilops	geniculata	Roth	2513821	España	Burgos	Ciruelos de Cervera	30TVM5840	0	1077		2006
Aegilops	geniculata	Roth	2513822	España	Burgos	Hontoria del Pinar	30TVM8430	0	1242		2006
Aegilops	geniculata	Roth	2513823	España	Burgos	Hontoria del Pinar	30TVM8728	0	1248		2006
Aegilops	geniculata	Roth	2513824	España	Burgos	Hontoria del Pinar	30TVM8431	0	1185		2006
Aegilops	geniculata	Roth	2513825	España	Burgos	Hontoria del Pinar	30TVM8331	0	1258		2006
Aegilops	geniculata	Roth	2513826	España	Burgos	Sotresgudo, Caserío de M	30TUN9716	0	910		2006
Aegilops	geniculata	Roth	2513827	España	Burgos	Valle de Manzanedo, Pue	30TVN3254	0	1000		2006
Aegilops	geniculata	Roth	2513828	España	Burgos	Junta de Traslaloma, Coli	30TVN6465	0	700		2006
Aegilops	geniculata	Roth	2513829	España	Burgos	Valle de Losa, Lastras de	30TVN7762	0	675		2006
Aegilops	geniculata	Roth	2513830	España	Burgos	Merindad de Sotoscueva	30TVN4764	0	740		2006
Aegilops	geniculata	Roth	2513831	España	Burgos	Merindad de Montija, Po	30TVN6163	0	740		2006
Aegilops	geniculata	Roth	2513832	España	Burgos	Los Altos, Carretera hacia	30TVN4334	0	1120		2006
Aegilops	geniculata	Roth	2513833	España	Burgos	Oña, Cornudilla, c, Monte	30TVN6325	0	650		2006
Aegilops	geniculata	Roth	2513834	España	Burgos	Fuentenebro, Piedemont	30TVL3890	0	1190		2006
Aegilops	geniculata	Roth	2513835	España	Burgos	Entre Cabañes de Esguev	30TVM3331	0	865		2006
Aegilops	geniculata	Roth	2513836	España	Burgos	Vadocondes, Márgenes d	30TVM5210	0	730		2006
Aegilops	geniculata	Roth	2513837	España	Burgos	Condado de Treviño, Arri	30TWN2335	0	700	CB	2006
Aegilops	geniculata	Roth	2513838	España	Burgos	Contreras	30TVM6749	0	1315		2006
Aegilops	geniculata	Roth	2513839	España	Burgos	Contreras	30TVM6749	0	1372		2006
Aegilops	geniculata	Roth	2513840	España	Burgos	Contreras	30TVM6353	0	1030		2006
Aegilops	geniculata	Roth	2513841	España	Burgos	Valle de Mena, Irús	30TVN6871	0	540	CB	2006
Aegilops	geniculata	Roth	2513842	España	Burgos	Villaescusa de Roa, Junto	30TVM12	1		CB	2006

Aegilops	geniculata	Roth	2513843	España	Burgos	Tubilla del Agua	30TVN3523	0	1020		2006
Aegilops	geniculata	Roth	2513844	España	Burgos	Arauzo de Miel	30TVM6938	0	1139		2006
Aegilops	geniculata	Roth	2513845	España	Burgos	Burgos	30TVM4390	0	880		2006
Aegilops	geniculata	Roth	2513846	España	Burgos	Entre Belorado y Pradolu	30TVM89	1		CB	2006
Aegilops	geniculata	Roth	2513847	España	Burgos	Montorio	30TVN3715	0		CB	2006
Aegilops	geniculata	Roth	2513848	España	Burgos	Valle de Losa, Quincoces	30TVN8059	0	650		2006
Aegilops	geniculata	Roth	2513849	España	Burgos	Valle de Losa, Relloso	30TVN8062	0	700		2006
Aegilops	geniculata	Roth	2513850	España	Burgos	Burgos capital, inmediaci	30TVM3890	0	850		2006
Aegilops	geniculata	Roth	2513851	España	Burgos	Oña, Hacia Pino de Bureb	30TVN6429	0	600	CB	2006
Aegilops	geniculata	Roth	2513852	España	Burgos	Frías	30TVN7534	0	530	CB	2006
Aegilops	geniculata	Roth	2513853	España	Burgos	Frías	30TVN7534	0	550	CB	2006
Aegilops	geniculata	Roth	2513854	España	Burgos	Frías	30TVN7534	0	600	CB	2006
Aegilops	geniculata	Roth	2513855	España	Burgos	Frías	30TVN7534	0	600	CB	2006
Aegilops	geniculata	Roth	2513856	España	Burgos	Partido de la Sierra en To	30TVN7331	0	750	CB	2006
Aegilops	geniculata		2604138	España	Alicante	Parque Natural de la Fon	30SYH1784				2011
Aegilops	geniculata		2604139	España	Alicante	Parque Natural de la Fon	30SYH1984				2011
Aegilops	geniculata		2604140	España	Alicante	Parque Natural de la Fon	30SYH1583				2011
Aegilops	geniculata		2604141	España	Alicante	Parque Natural de la Fon	30SYH1584				2011
Aegilops	geniculata		2604142	España	Alicante	Parque Natural de la Fon	30SYH1585				2011
Aegilops	geniculata		2604143	España	Alicante	Parque Natural de la Fon	30SYH1679				2011
Aegilops	geniculata		2604144	España	Alicante	Parque Natural de la Fon	30SYH1780				2011
Aegilops	geniculata		2604145	España	Alicante	Parque Natural de la Fon	30SYH1880				2011
Aegilops	geniculata		2604146	España	Alicante	Parque Natural de la Fon	30SYH1883				2011
Aegilops	geniculata		2604147	España	Alicante	Parque Natural de la Fon	30SYH1884				2011
Aegilops	geniculata		2604148	España	Alicante	Parque Natural de la Fon	30SYH1980				2011
Aegilops	geniculata		2604149	España	Alicante	Parque Natural de la Fon	30SYH1983				2011
Aegilops	geniculata		2604150	España	Alicante	Parque Natural de la Fon	30SYH0983				2011
Aegilops	geniculata		2604151	España	Alicante	Parque Natural de la Fon	30SYH2183				2011
Aegilops	geniculata		2604152	España	Alicante	Parque Natural de la Fon	30SYH1582				2011
Aegilops	geniculata		2604153	España	Alicante	Parque Natural de la Fon	30SYH1982				2011
Aegilops	geniculata		2604154	España	Alicante	Parque Natural de la Fon	30SYH1080				2011
Aegilops	geniculata		2604155	España	Alicante	Parque Natural de la Fon	30SYH1180				2011
Aegilops	geniculata		2604156	España	Alicante	Parque Natural de la Fon	30SYH0981				2011
Aegilops	geniculata		2604157	España	Alicante	Parque Natural de la Fon	30SYH1484				2011
Aegilops	geniculata		2604158	España	Alicante	Parque Natural de la Fon	30SYH1182				2011
Aegilops	geniculata		2604159	España	Alicante	Parque Natural de la Fon	30SYH1183				2011
Aegilops	geniculata		2604160	España	Alicante	Parque Natural de la Fon	30SYH1281				2011
Aegilops	geniculata		2604161	España	Alicante	Parque Natural de la Fon	30SYH1378				2011
Aegilops	geniculata		2604162	España	Alicante	Parque Natural de la Fon	30SYH1382				2011

Aegilops	geniculata		2604163	España	Alicante	Parque Natural de la Fon	30SYH1383				2011
Aegilops	geniculata		2604164	España	Alicante	Parque Natural de la Fon	30SYH1478				2011
Aegilops	geniculata		2604165	España	Alicante	Parque Natural de la Fon	30SYH1479				2011
Aegilops	geniculata		2604166	España	Alicante	Parque Natural de la Fon	30SYH1482				2011
Aegilops	geniculata		2604167	España	Alicante	Parque Natural de la Fon	30SYH1781				2011
Aegilops	geniculata		2604168	España	Alicante	Parque Natural de la Fon	30SYH1379				2011
Aegilops	ovata		2614413	España	Zaragoza	Chiprana	30TYL47	1			1997
Aegilops	geniculata		2617757	España	Teruel	Valle de Escriche	30TXK77		1260-1500		2003
Aegilops	geniculata		2617758	España	Teruel	Valle de Escriche	30TXK77		1260-1500		2003
Aegilops	geniculata		2637929	España	Toledo	Los Yébenes	30SVJ28	1			1982
Aegilops	geniculata		2648878	España	Guadalajara	Puebla de Vallés	30TVL73	1	1000		1985
Aegilops	geniculata		2648879	España	Guadalajara	Tamajón	30TVL73	1	1000		1985
Aegilops	geniculata		2648880	España	Guadalajara	Valdepeñas de la Sierra	30TVL62	1	730		1985
Aegilops	geniculata		2648881	España	Guadalajara	Pontón de la Oliva	30TVL62	1	800		1985
Aegilops	geniculata		2648882	España	Guadalajara	Pontón de la Oliva	30TVL62	1	830		1985
Aegilops	geniculata		2648883	España	Guadalajara	Torrebeleña	30TVL82	1	830		1985
Aegilops	geniculata		2648884	España	Madrid	Torrelaguna	30TVL52	1	830		1985
Aegilops	geniculata		2648885	España	Guadalajara	Valdepeñas de la Sierra	30TVL62	1	870		1985
Aegilops	geniculata		2648886	España	Guadalajara	Alpedrete de la Sierra	30TVL62	1	900		1985
Aegilops	geniculata		2648887	España	Guadalajara	Puebla de Baleña	30TVL82	1	950		1985
Aegilops	geniculata		2656317	España	Lérida	l'Espluga Calba	31TCF39	1			1987-88
Aegilops	geniculata		2677374	España	Salamanca	Monterrubio de la Armuí	30TTL74	1	810		1996
Aegilops	geniculata		2678181	España	Almería	La Loma	30SWF47	1	60		1999
Aegilops	geniculata		2678483	España	Jaén	Los Villares, Puerto Viejo	30SVG26	1	1200		2000
Aegilops	geniculata		2678484	España	Jaén	Valdepeñas de Jaén, próx	30SVG26	1	1200		2000
Aegilops	geniculata	Roth.	2690713	España	Valladolid	Cabezón de Pisuerga	30TUM6722		840-850		2011
Aegilops	geniculata	Roth.	2690714	España	Valladolid	Cigales	30TUM5729		840		2011
Aegilops	geniculata	Roth.	2690715	España	Valladolid	Corcos del Valle	30TUM5931		850		2011
Aegilops	geniculata	Roth.	2690716	España	Valladolid	Peñaflor de Hornija	30TUM3520		840		2011
Aegilops	geniculata	Roth.	2690717	España	Valladolid	Zaratán	30TUM5113		760		2011
Aegilops	geniculata		2692395	España	Cuenca	Serranía de Cuenca, Caña	30TWK9276		1473		2012
Aegilops	geniculata		2692396	España	Cuenca	Serranía de Cuenca, Caña	30TWK9277		1471		2012
Aegilops	geniculata		2692397	España	Cuenca	Serranía de Cuenca, Punt	30TWK9177		1462		2012
Aegilops	geniculata		2712149	España	Málaga	El Burgo. Sierra Blanquilla	30SUF2972		850		2012
Aegilops	geniculata		2713464	España	Jaén	Cortijos Nuevos, El Conta	30SWH23	1			2012
Aegilops	geniculata	Roth	2715962	España	Islas Baleares	Menorca, Arenal de Sant	31SEE91	1			2009
Aegilops	geniculata	Roth	2716316	España	Islas Baleares	Eivissa, es Codolar	31SCD50	1			2011
Aegilops	ovata	Linn.	2722390	España	Islas Baleares	In collibus petrosis circa P	31SDD68	1			1827
Aegilops	geniculata		2728708	España	Guadalajara	Entre Sigüenza y Pelegrin	30TWL34	1	1100		1998

Aegilops	geniculata		2728709	España	Guadalajara	Riba de Santiuste	30TWL26	1	1100		1998
Aegilops	geniculata		2728710	España	Guadalajara	Riba de Santiuste	30TWL26	1	1000		1998
Aegilops	geniculata		2728711	España	Guadalajara	Muduex	30TWL02	1	1010		1998
Aegilops	geniculata		2728712	España	Guadalajara	Atienza	30TWL16	1	1130		1998
Aegilops	geniculata		2728713	España	Guadalajara	Entre Valdelcubo y Barah	30TWL26	1	1140		1998
Aegilops	geniculata	Roth	2729743	España	Badajóz	Badajoz. Proximidades de	29SPD70				2013
Aegilops	geniculata	Roth	2745525	España	ESPAÑA	Sierra de Mariola	30SYH19	1		CB	2012
Aegilops	geniculata	Roth	2746667	España	Teruel	Ladruñán (Castellote, Ma	30TYL10				2012
Aegilops	geniculata	Roth	2746668	España	Teruel	Ladruñán (Castellote, Ma	30TYL11				2012
Aegilops	geniculata	Roth	2746669	España	Teruel	Ladruñán (Castellote, Ma	30TYL20				2012
Aegilops	geniculata	Roth	2746670	España	Teruel	Ladruñán (Castellote, Ma	30TYL21				2012
Aegilops	geniculata		2749724	España	Madrid	Oteruelo del Valle	30TVL2629		1140		1988
Aegilops	geniculata		2749725	España	Madrid	Rascafría - Oteruelo del V	30TVL2729		1140		1988
Aegilops	geniculata		2749726	España	Madrid	Oteruelo del Valle	30TVL2729		1140		1988
Aegilops	geniculata		2749727	España	Madrid	Oteruelo del Valle	30TVL2729		1140		1988
Aegilops	geniculata		2749728	España	Madrid	Rascafría - Oteruelo del V	30TVL2729		1140		1988
Aegilops	geniculata		2749729	España	Madrid	Rascafría - Oteruelo del V	30TVL2729		1140		1988
Aegilops	geniculata		2749730	España	Madrid	Alameda del Valle	30TVL2830		1130		1988
Aegilops	geniculata		2749731	España	Madrid	Alameda del Valle	30TVL2830		1130		1988
Aegilops	geniculata		2749732	España	Madrid	Alameda del Valle	30TVL2830		1130		1988
Aegilops	geniculata		2749733	España	Madrid	Cruce de Pinilla del Valle	30TVL3031		1100		1988
Aegilops	geniculata		2749734	España	Madrid	Pinilla del Valle	30TVL3031		1100		1988
Aegilops	geniculata		2749735	España	Madrid	Pinilla del Valle, cerro cal	30TVL3231		1110		1988
Aegilops	geniculata		2749736	España	Madrid	Cerros calizos de Pinilla d	30TVL3231		1110		1988
Aegilops	geniculata		2749737	España	Madrid	Buitrago - Villavieja de Lo	30TVL4544		1000		1988
Aegilops	geniculata		2749738	España	Madrid	Solanas de Lozoyuela	30TVL4731		1060		1988
Aegilops	geniculata		2749739	España	Madrid	Solanas de Lozoyuela	30TVL4731		1060		1988
Aegilops	ovata	L.	2786976	España	Madrid	Cerca de Madrid	30TVK47	1			1861
Aegilops	geniculata	Roth	2791167	España	Tarragona	Baix Ebre, Alfara de Carle	31TBF7722		1030		2006
Aegilops	geniculata	Roth	2791168	España	Tarragona	Montsià, Masdenverge-G	31TBF90		60		2006
Aegilops	neglecta	Reg. ex Bertol.	1339636	España	Albacete	Albacete, provincia	30SWH6250	0			1996
Aegilops	triaristata		2100023	España	Almería	Enix, cerros	30SWF38	1	750		1969
Aegilops	triaristata	Willd.	1012899	España	Almería	Río Aguas	30SWG01	1			1968
Aegilops	triaristata	Willd.	1012992	España	Almería	Río Aguas	30SWG01	1			1971
Aegilops	neglecta	Req. & Bertol	1013432	España	Almería	Los Yesos, Tabernas, alre	30SWG60	0			1987
Aegilops	triaristata	Willd.	1012901	España	Almería	Venta de los Yesos	30SWG60	1			1968
Aegilops	triaristata	Willd.	1012902	España	Almería	Venta de los Yesos-Taber	30SWG60	1			1968
Aegilops	triaristata	Willd.	1012900	España	Almería	Sorbas	30SWG70	1			1968
Aegilops	triaristata	Willd.	918142	España	Barcelona	Tibidabo	31TDF28	1			1911

Aegilops	triaristata	Willd.	918143	España	Barcelona	Tarrasa	31TDG10	1			1911
Aegilops	neglecta	Req. ex Bertol.	2513861	España	Burgos	Burgos	30TVM48	0		CB	2006
Aegilops	neglecta	Req. ex Bertol.	2513862	España	Burgos	Burgos, Gamonal	30TVM48	0		CB	2006
Aegilops	neglecta	Req. ex Bertol.	2513859	España	Burgos	Santo Domingo de Silos	30TVM6246	0	983		2006
Aegilops	neglecta	Req. ex Bertol.	2513858	España	Burgos	Hortigüela	30TVM6357	0	1004		2006
Aegilops	neglecta	Req. ex Bertol.	2513857	España	Burgos	Hortigüela	30TVM6457	0	983		2006
Aegilops	neglecta	Req. ex Bertol.	2749375	España	Burgos	Fresneda de la Sierra Tiró	30TVM8885		1040		2012
Aegilops	neglecta	Req. ex Bertol.	2513860	España	Burgos	Villarcayo, Encinillas	30TVN54	1		CB	2006
Aegilops	triaristata	W.	2364339	España	Burgos	c. Encinillas [Incinillas] pr	30TVN54	1			1861-1862
Aegilops	triaristata		1549516	España	Burgos	c. Encinillas [Incinillas] pr	30TVN54	1			1861
Aegilops	neglecta	Req. ex BertoL.	1835096	España	Ciudad Real	intercalación basáltica de	30SUH48	1		CB	2005
Aegilops	neglecta	Req. ex BertoL.	1835094	España	Ciudad Real	Intercalación calizo-devó	30SUH49	1		CB	2005
Aegilops	neglecta	Req. ex BertoL.	1835111	España	Ciudad Real	bajada Puerto Caracoller	30SUH78	1		CB	2005
Aegilops	neglecta	Req. ex BertoL.	1835117	España	Ciudad Real	Brazatortas, valle del arro	30SUH7962	0	740	CB	2005
Aegilops	neglecta	Req. ex BertoL.	1835098	España	Ciudad Real	Fuencaliente, Sierra Mad	30SUH8560	0	780	CB	2005
Aegilops	neglecta	Req. ex BertoL.	1835097	España	Ciudad Real	Fuencaliente, valle del río	30SUH8856	0	860	CB	2005
Aegilops	neglecta	Req. ex BertoL.	1835095	España	Ciudad Real	Fuencaliente, sierra del C	30SUH9353	0	820	CB	2005
Aegilops	neglecta	Req. ex Bertol.	1348605	España	Ciudad Real	Puebla de Don Rodrigo, F	30SUJ7019	0	490		1998
Aegilops	neglecta	Req. ex BertoL.	1835102	España	Ciudad Real	Puebla de Don Rodrigo, p	30SUJ7019	0	490	CB	2005
Aegilops	neglecta	Req. ex Bertol.	1482315	España	Ciudad Real	P.N. Cabañeros	30SUJ76	1			1993
Aegilops	neglecta	Req. ex BertoL.	1835114	España	Ciudad Real	Abenójar, entre Casa de l	30SUJ8112	0	680	CB	2005
Aegilops	neglecta	Req. ex BertoL.	1835099	España	Ciudad Real	Piedrabuena, chopera pr	30SUJ8129	0	700	CB	2005
Aegilops	neglecta	Req. ex Bertol.	1348607	España	Ciudad Real	Piedrabuena, finca El Gar	30SUJ8129	0			1998
Aegilops	neglecta	Req. ex BertoL.	1835116	España	Ciudad Real	Alcoba, Cabañeros, Guar	30SUJ8553	0		CB	2005
Aegilops	neglecta	Req. ex Bertol.	1374900	España	Ciudad Real	Montes de Toledo, Parqu	30SUJ8553	0			1993
Aegilops	neglecta		1762691	España	Ciudad Real	Volcán de Piedrabuena	30SUJ92	0			2004
Aegilops	neglecta		1762690	España	Ciudad Real	Cerro de la Higuera	30SUJ95	0			2004
Aegilops	neglecta	Req. ex Bertol.	1348606	España	Ciudad Real	Piedrabuena	30SUJ9821	1	700		1998
Aegilops	neglecta	Req. ex BertoL.	1835107	España	Ciudad Real	Solana del Pino, Alhorín	30SVH0365	0	660	CB	2005
Aegilops	neglecta	Req. ex BertoL.	1835100	España	Ciudad Real	Mestanza, valle del río Ro	30SVH1452	0	440	CB	2005
Aegilops	neglecta	Req. ex BertoL.	1835112	España	Ciudad Real	Aldea del Rey, cerro de la	30SVH2087	0	750	CB	2005
Aegilops	neglecta	Req. ex BertoL.	1835106	España	Ciudad Real	San Lorenzo de Calatrava	30SVH2359	0	620	CB	2005
Aegilops	neglecta	Req. ex BertoL.	1835113	España	Ciudad Real	Aldea del Rey, cerro de la	30SVH2389	0	790	CB	2005
Aegilops	neglecta	Req. ex BertoL.	1835109	España	Ciudad Real	Viso del Marqués, sierra d	30SVH3654	0	900	CB	2005
Aegilops	neglecta	Req. ex BertoL.	1835110	España	Ciudad Real	Viso del Marqués, Camin	30SVH5058	0	840	CB	2005
Aegilops	neglecta	Req. ex BertoL.	1835093	España	Ciudad Real	Despeñaperros	30SVH55	1		CB	2005
Aegilops	neglecta	Req. ex BertoL.	1835115	España	Ciudad Real	Alcubillas, alrededores	30SVH9089	0	830	CB	2005
Aegilops	neglecta	Req. ex Bertol.	1869150	España	Ciudad Real	Ciudad Real, La Atalaya	30SVJ22	1	670		2004
Aegilops	neglecta	Req. ex BertoL.	1835108	España	Ciudad Real	sierra de Alhambra	30SVJ90	1		CB	2005

Aegilops	neglecta	Req. ex BertoL.	1835101	España	Ciudad Real	Puebla del Príncipe, cerca	30SWH0864	0	940	CB	2005
Aegilops	neglecta		1093234	España	Cáceres	Finca de Valdelasyeguas,	29SPD96	0			1989
Aegilops	neglecta		1093237	España	Cáceres	Finca de Araya, Arroyo de	29SQD07	1			1989
Aegilops	neglecta		1093245	España	Cáceres	Cerro de Aldeamoret	29SQD26	1			1989
Aegilops	neglecta		1093240	España	Cáceres	Cerro de Aldeamoret	29SQD26	1			1989
Aegilops	neglecta		1093241	España	Cáceres	Cerro de Aldeamoret	29SQD26	1			1989
Aegilops	neglecta		1093254	España	Cáceres	El Portanchito, casas de l	29SQD27	1			1989
Aegilops	neglecta		1093243	España	Cáceres	Coria	29SQE12	1			1989
Aegilops	neglecta		1093232	España	Cáceres	El Arco, Cañaveral	29SQE20	0			1989
Aegilops	neglecta		1093256	España	Cáceres	El Arco, Cañaveral	29SQE20	0			1989
Aegilops	neglecta		1093258	España	Cáceres	Dehesa de Valdeobispo, A	29TQE34	1			1989
Aegilops	neglecta		1093246	España	Cáceres	Dehesa de Valdeobispo, A	29TQE34	1			1989
Aegilops	neglecta		1093229	España	Cáceres	Dehesa de los Caballos, P	29TQE43	0			1989
Aegilops	neglecta		1093230	España	Cáceres	Dehesa de los Caballos, P	29TQE43	0			1989
Aegilops	neglecta		1093247	España	Cáceres	Cerro Calero, Campillo de	30STJ79	0			1989
Aegilops	triaristata	Willd.	1091705	España	Cáceres	Guadalupe	30STJ97	1			1952
Aegilops	neglecta	Req. Ex Bertol	1715000	España	Cáceres	Sierra las Corchuelas, Par	30STK51	1			1986
Aegilops	neglecta		1093249	España	Cáceres	Almaraz	30STK70	0			1989
Aegilops	neglecta		1093248	España	Cáceres	Almaraz	30STK70	0			1989
Aegilops	triaristata	Willd.	1091707	España	Cáceres	Alía	30SUJ06	1			1952
Aegilops	triaristata	Willd.	1091706	España	Cáceres	Río Guadarranque	30SUJ16	1			1952
Aegilops	neglecta		1093259	España	Cáceres	Tejeda de Tiétar, Castillej	30TTK5332	0			1993
Aegilops	neglecta	Req.	1093263	España	Cáceres	Comarca de la Vera	30TTK63	0			1993
Aegilops	neglecta	Req.	1331445	España	Cáceres	Comarca de la Vera	30TTK63	0			1993
Aegilops	neglecta		1093260	España	Cáceres	Valverde de la Vera, La V	30TTK9241	0			1993
Aegilops	neglecta		1093262	España	Cáceres	Villanueva de la Vera	30TTK9445	0			1993
Aegilops	neglecta	Req ex Bertol	1048664	España	Cádiz	Montes de Propios de Jer	29SQA56	1			1991
Aegilops	neglecta	Req. & Bertol	1050670	España	Cádiz	Sierra de Algarrobo	30STE79	1			1985
Aegilops	neglecta	Req. ex Bertol.	1451608	España	Cádiz	Villaluenga del Rosario, B	30STF8764	0	700		1987
Aegilops	neglecta	Req. ex Bertol.	2309227	España	Córdoba	Sierra Morena, NE, N de	30STH94	1			1986
Aegilops	neglecta	Req. ex Bertol.	2309228	España	Córdoba	Sierra Morena, SW, desd	30STH94	1			1986
Aegilops	neglecta	Req. ex Bertol.	2309229	España	Córdoba	Puente Genil	30SUG43	1			1986
Aegilops	neglecta	Req. ex Bertol.	2309230	España	Córdoba	Valle del Guadalquivir, de	30SUG59	1			1986
Aegilops	neglecta	Req. ex Bertol.	2309231	España	Córdoba	Belalcázar, estación, entr	30SUH28	1			1986
Aegilops	neglecta	Req. ex Bertol.	2309232	España	Córdoba	Villanueva de Córdoba, p	30SUH54	1			1986
Aegilops	neglecta	Req. ex Bertol.	2309233	España	Córdoba	Cardeña, Valle de los Pec	30SUH83	1			1986
Aegilops	neglecta		1715692	España	Gerona	Roses, marge de camí al	31TEG1280	0			2006
Aegilops	neglecta	Req. Ex Bertol.	1715694	España	Gerona	Roses,al puig de les Garri	31TEG1280	0			2006
Aegilops	neglecta		1715693	España	Gerona	Palau-saverdera, prat pas	31TEG1282	0			2006

Aegilops	neglecta		1755605	España	Gerona	Cap de Creus, Camp de c	31TEG17	0	40		1995
Aegilops	neglecta		1755642	España	Gerona	Cap de Creus, Marge de l	31TEG18	1	30		1995
Aegilops	neglecta		1753299	España	Gerona	Cap de Creus, Camí sota (	31TEG28	1	50		1995
Aegilops	triaristata	Willd.	1180911	España	Gerona	Port-Vendres	31TEH00	1			1911
Aegilops	neglecta	Req. ex Bertol.	2242502	España	Granada	Arenas del Rey	30SVF2285	0			2005
Aegilops	triaristata	W.	2366923	España	Granada	prope Granatam	30SVG41	1			1861-1862
Aegilops	neglecta	Req. ex Bertol.	1376736	España	Granada	Sierra de Baza, Parque Na	30SWG13	1			1991
Aegilops	neglecta	Req. ex Bertol.	2442822	España	Guadalajara	Pareja	30TWK28	0			1984
Aegilops	neglecta	Req. ex Bertol.	2442821	España	Guadalajara	Alcocer	30TWK38	0			1984
Aegilops	neglecta	Req. ex Bertol.	2442820	España	Guadalajara	Millana	30TWK38	0			1984
Aegilops	triaristata	Willd.	1060680	España	Huelva	Cartaya	29SPB62	1			1948
Aegilops	triaristata	Willd.	1060679	España	Huelva	Almonaster	29SPB98	1			1948
Aegilops	neglecta	Req. & Bertol	1062081	España	Huelva	Sierra de Aracena	29SQB09	0			1985
Aegilops	neglecta	Req. & Bertol	1065854	España	Huelva	Sierra de Aracena	29SQB19	0			1985
Aegilops	triaristata	Willd.	1060678	España	Huelva	Santa Olalla	29SQB49	1			1948
Aegilops	neglecta	Req. ex Bertol.	1465441	España	Huesca	Aniés	30TYM0187	0	1000		2002
Aegilops	neglecta	Req. ex Bertol.	1465439	España	Huesca	Villanueva de Sigena, Río	30TYM4621	0	200		2002
Aegilops	neglecta	Req. ex Bertol.	1465440	España	Huesca	Jánovas	30TYN4604	0	670		2002
Aegilops	neglecta	Req. ex Bertol.	1774962	España	Huesca	Almunia de San Juan, Ari	31TBG6650	0	270		2004
Aegilops	triaristata	W.	1185295	España	Islas Baleares	Ibiza	31SCD60	1			1900
Aegilops	ovata	L.	1481133	España	Islas Baleares	P.N. Archipiélago de Cabr	31SDD93	1			1976
Aegilops	neglecta	Req ex. Bertol.	1712651	España	Islas Baleares	Menorca, Ciutadella, Sta.	31SEE7921	0			1998
Aegilops	neglecta	Req. ex Bertol	1712962	España	Islas Baleares	Menorca, Es Berrecks de	31SEE82	1		CB	2000
Aegilops	neglecta	Req. ex Bertol	1712963	España	Islas Baleares	Menorca, Alaior, Son Làd	31SEE9927	0	100		2000
Aegilops	neglecta	Req. ex Bertol.	1027214	España	Jaén	Marmolejo	30SUH9345	0	500		1994
Aegilops	neglecta	Req. ex Bertol.	1027213	España	Jaén	Marmolejo	30SUH9748	0	800		1994
Aegilops	neglecta	Req. ex Bertol.	1027215	España	Jaén	Marmolejo	30SVH0048	0	600		1994
Aegilops	neglecta	Req. ex Bertol.	1332456	España	Jaén	Enjambradero	30SVH0141	0			1996
Aegilops	neglecta	Req. ex Bertol.	1027216	España	Jaén	Marmolejo	30SVH0240	0	700		1994
Aegilops	neglecta	Req. ex Bertol.	1027217	España	Jaén	Marmolejo	30SVH0434	0	600		1994
Aegilops	triaristata	Willd.	2786977	España	Madrid	Aranjuez	30TVK43	1		CB	1861
Aegilops	triaristata	W.	2364340	España	Madrid	Aranjuez	30TVK43	1			1861-1862
Aegilops	triaristata	W.	2364328	España	Madrid	Madrid	30TVK47	1			1861-1862
Aegilops	triaristata	Willd.	2786978	España	Madrid	Madrid	30TVK47	1		CB	1861
Aegilops	neglecta	Req. ex Bertol	1011319	España	Murcia	Sierra de Espuña	30SXG2993	0	1200		1988
Aegilops	neglecta	Req. ex Bertol.	2451769	España	Murcia	Sierra Espuña	30SXG39	1		CB	2011
Aegilops	neglecta	Req. ex Bertol.	2451770	España	Murcia	Sierras de Cartagena	30SXG66	1		CB	2011
Aegilops	neglecta	Req. ex Bertol.	2242504	España	Málaga	Sierra de Líbar	30STF95	1			2007
Aegilops	neglecta	Req. ex Bertol.	2242503	España	Málaga	Benaoján	30STF96	1			2007

Aegilops	neglecta	Req. ex Bertol	1043767	España	Málaga	Estepona	30SUF03	0			1990
Aegilops	neglecta	Req. ex Bertol	1043770	España	Málaga	Ronda, Sierra de las Niev	30SUF17	1			1990
Aegilops	neglecta	Req. ex Bertol	1043766	España	Málaga	Coín	30SUF45	0			1990
Aegilops	neglecta	Req. ex Bertol	1043765	España	Málaga	Alora	30SUF47	0			1990
Aegilops	neglecta	Req. ex Bertol	1043764	España	Málaga	Alhaurín el Grande, Sierra	30SUF55	0			1990
Aegilops	neglecta	Req. ex Bertol	1043769	España	Málaga	Mijas	30SUF55	0			1990
Aegilops	neglecta	Req. ex Bertol	1043768	España	Málaga	Málaga	30SUF76	0			1990
Aegilops	triaristata	Willd.	1868447	España	Málaga	Circà Malaga	30SUF77	1			1839
Aegilops	neglecta	Req. ex Bertol.	2713094	España	Palencia	Monzón de Campos	30TUM76	1		CB	2012
Aegilops	neglecta	Req. ex Bertol.	2713093	España	Palencia	Cordovilla la Real	30TUM95	1		CB	2012
Aegilops	neglecta	Req. ex Bertol.	2713095	España	Palencia	Rabanal de las Llantas [3(	30TUN6750		1000		2012
Aegilops	neglecta	Req. ex Bertol	74650	España	Salamanca	Montemayor del río	30TTK57	1			1985
Aegilops	neglecta	Req. ex Bertol	65368	España	Salamanca	Guijuelo	30TTK79	1			1989
Aegilops	neglecta	Reg. ex Bertol	57501	España	Salamanca	Matilla de los Caños del r	30TTL52	1			1984
Aegilops	neglecta	Req. ex Bertol	52931	España	Salamanca	Arapiles	30TTL73	1			1984
Aegilops	neglecta		1810918	España	Salamanca	Monterrubio de la Armui	30TTL7747	0	800		2006
Aegilops	neglecta	Req. ex Bertl.	1109625	España	Salamanca	Aldeanueva de Figueroa	30TTL85	1			1996
Aegilops	neglecta	Req. ex Bertol.	54858	España	Salamanca	Bóveda del río Almar	30TUL12	1			1983
Aegilops	neglecta	Req. ex Bertol.	120461	España	Segovia	Montejo de la Vega de la	30TVM40	0			1996
Aegilops	neglecta	Req. ex Bertol.	2418176	España	Sevilla	Castilblanco de los Arroy	30STG37	1	300		1987
Aegilops	triaristata	Wk.	1067629	España	Sevilla	Alcalá de Guadaira	30STG43	1			1897
Aegilops	triaristata	Willd.	1066942	España	Sevilla	Paradas	30STG72	1			1948
Aegilops	neglecta		86017	España	Soria	Santa María de las Hoyas	30TVM92	0			1987
Aegilops	triaristata	Willd.	987987	España	Soria	Berlanga	30TWL19	1			1942
Aegilops	triaristata	Willd.	987985	España	Soria	Cañamaque	30TWL68	1			1942
Aegilops	triaristata	Willd.	987986	España	Soria	Serón	30TWL69	1			1942
Aegilops	triaristata	Willd.	987984	España	Soria	Nódalo	30TWM11	1			1942
Aegilops	neglecta		86871	España	Soria	Arguijo	30TWM44	0			1983
Aegilops	neglecta	Req.	2064065	España	Teruel	Comunidad de Albarracír	30TXK0880	0			2008
Aegilops	neglecta		1674402	España	Toledo	Talavera, carretera a Seg	30TUK4431	0	440		2004
Aegilops	neglecta		1674404	España	Toledo	San Román	30TUK5237	0	480		2004
Aegilops	neglecta		1674403	España	Toledo	Lucillos, hacia Cardiel de	30TUK6231	0	350		2004
Aegilops	neglecta	Req. ex Bertol	47602	España	Valladolid	Castronuño, Valdecierva,	30TUL18	1			1988
Aegilops	neglecta	Req. ex Bertol	1125956	España	Valladolid	Dehesa Raso de Portillo	30TUL59	1			1984
Aegilops	neglecta	Req. ex Bertol.	116426	España	Valladolid	Almenara de Adaja	30TUL6163	0			1987
Aegilops	neglecta	Req. ex Portol.	78794	España	Valladolid	Mojados	30TUL68	1			1983
Aegilops	neglecta	Req. ex Bertol	1125957	España	Valladolid	Pedraja de Portillo	30TUL69	1			1984
Aegilops	neglecta	Req. ex Portol.	78795	España	Valladolid	Portillo	30TUL69	1			1983
Aegilops	neglecta	Req. ex Portol.	78793	España	Valladolid	Cogeces de Íscar	30TUL78	1			1983

Aegilops	neglecta	Reg. ex Bertol	96904	España	Zamora	Fermoselle	29TQF17	1			1983
Aegilops	neglecta	Reg. ex Bertol	96903	España	Zamora	Pereruela	30TTL58	1			1983
Aegilops	neglecta	Req. Ex Bertol.	76098	España	Zamora	Cuelgamures	30TTL77	1			1984
Aegilops	neglecta	Req. Ex Bertol.	76096	España	Zamora	El Pego	30TTL97	1			1984
Aegilops	neglecta	Req. ex Bertol.	99841	España	Zamora	Moreruela de Tábara	30TTM63	1			1982
Aegilops	neglecta	Req. ex Bertol	1115799	España	Zamora	Moreruela de Tábara	30TTM63	1			1984
Aegilops	neglecta	Req. ex Bertol	1120224	España	Zamora	Fresno de la Ribera, La Ce	30TTM80	1			1994
Aegilops	neglecta	Req. Ex Bertol.	76097	España	Zamora	Cañizal	30TUL06	1			1984
Aegilops	neglecta	Req. ex Bertol	1120225	España	Zamora	Pinilla de Toro, El Ama	30TUM01	1			1994
Aegilops	neglecta	Req. ex Bertol	89107	España	Ávila	Barraco, Valle de Iruelas	30TUK67	1			1992
Aegilops	triuncialis		2103312	España	Albacete	Sierra de Alcaraz, Cerros	30SWH47	1	1250		1969
Aegilops	triuncialis	L.	1339637	España	Albacete	Albacete, provincia	30SWH5249	0			1996
Aegilops	triuncialis	L.	2158666	España	Albacete	Fuentealbilla	30SXJ2547	0			2008
Aegilops	triuncialis		2162071	España	Albacete	Alcalá del Júcar	30SXJ3638	0	720		2008
Aegilops	triuncialis	L.	2158667	España	Albacete	Casas de Ves	30SXJ44	1			2008
Aegilops	triuncialis	L.	2158668	España	Albacete	Villa de Ves	30SXJ5041	0			2008
Aegilops	triuncialis		1002570	España	Albacete	Bosque de Alpera	30SXJ51	1			1985
Aegilops	triuncialis		2200313	España	Alicante	Provincia de Alicante	30SXH75	0			2007
Aegilops	triuncialis		2200312	España	Alicante	Provincia de Alicante	30SXH76	0			2007
Aegilops	triuncialis		2200310	España	Alicante	Provincia de Alicante	30SXH77	0			2007
Aegilops	triuncialis		2200315	España	Alicante	Provincia de Alicante	30SXH79	0			2007
Aegilops	triuncialis		2200316	España	Alicante	Provincia de Alicante	30SXH83	0			2007
Aegilops	triuncialis		2200317	España	Alicante	Provincia de Alicante	30SXH84	0			2007
Aegilops	triuncialis		2200318	España	Alicante	Provincia de Alicante	30SXH87	0			2007
Aegilops	triuncialis		2200319	España	Alicante	Provincia de Alicante	30SXH89	0			2007
Aegilops	triuncialis		2708258	España	Alicante	Arenal de Biar, Peñarrubi	30SXH9174		680		2010
Aegilops	triuncialis		2708259	España	Alicante	Arenal de Petrel	30SXH9364		520		2010
Aegilops	triuncialis		2708261	España	Alicante	Arenal de Petrel	30SXH9364		500		2010
Aegilops	triuncialis		2708260	España	Alicante	Arenal de Petrel	30SXH9364		505		2010
Aegilops	triuncialis		2200320	España	Alicante	Provincia de Alicante	30SXH95	0			2007
Aegilops	triuncialis		2200321	España	Alicante	Provincia de Alicante	30SXH96	0			2007
Aegilops	triuncialis	L.	2147314	España	Alicante	Sierra del Cid	30SXH96	1		CB	2003
Aegilops	triuncialis		2200314	España	Alicante	Provincia de Alicante	30SXH97	0			2007
Aegilops	triuncialis		2200306	España	Alicante	Provincia de Alicante	30SXH98	0			2007
Aegilops	triuncialis	L.	2147316	España	Alicante	Sierra del Cid	30SXH9863	0			2003
Aegilops	triuncialis	L.	2147318	España	Alicante	Sierra del Cid	30SXH9962	0			2003
Aegilops	triuncialis		2200299	España	Alicante	Provincia de Alicante	30SYH05	0			2007
Aegilops	triuncialis		2200300	España	Alicante	Provincia de Alicante	30SYH06	0			2007
Aegilops	triuncialis		2200301	España	Alicante	Provincia de Alicante	30SYH08	0			2007

Aegilops	triuncialis		2604171	España	Alicante	Parque Natural de la Fon	30SYH1182				2011
Aegilops	triuncialis		2604169	España	Alicante	Parque Natural de la Fon	30SYH1482				2011
Aegilops	triuncialis		2604170	España	Alicante	Parque Natural de la Fon	30SYH1585				2011
Aegilops	triuncialis		2200302	España	Alicante	Provincia de Alicante	30SYH19	0			2007
Aegilops	triuncialis		2200303	España	Alicante	Provincia de Alicante	30SYH25	0			2007
Aegilops	triuncialis		2200304	España	Alicante	Provincia de Alicante	30SYH27	0			2007
Aegilops	triuncialis		2200309	España	Alicante	Provincia de Alicante	30SYH28	0			2007
Aegilops	triuncialis		2200305	España	Alicante	Provincia de Alicante	30SYH38	0			2007
Aegilops	triuncialis		2200298	España	Alicante	Provincia de Alicante	30SYH39	0			2007
Aegilops	triuncialis		2200307	España	Alicante	Provincia de Alicante	30SYJ50	0			2007
Aegilops	triuncialis		2200311	España	Alicante	Provincia de Alicante	31SBC49	0			2007
Aegilops	triuncialis		2200308	España	Alicante	Provincia de Alicante	31SBC59	0			2007
Aegilops	triuncialis		2103677	España	Almería	Sierra de Gádor	30SWF28	1	1350-1950		1969
Aegilops	triuncialis	L.	1012903	España	Almería	Río Aguas	30SWG01	1			1968
Aegilops	triuncialis	L.	1012905	España	Almería	Sorbas	30SWG70	1			1968
Aegilops	triuncialis	L.	1012904	España	Almería	Turre	30SWG91	1			1968
Aegilops	triuncialis	L.	904085	España	Barcelona	Barcelona	31TDF38	1			1953
Aegilops	triuncialis	L.	918145	España	Barcelona	Montserrat	31TDG00	1			1953
Aegilops	triuncialis	L.	915851	España	Barcelona	Guardiola de Berguedà	31TDG07	0			1985
Aegilops	triuncialis	L.	904087	España	Barcelona	Entre Matadepera y Tarr	31TDG10	1			1953
Aegilops	triuncialis	L.	913849	España	Barcelona	Massif de St. Llorenç, Val	31TDG11	1			1974
Aegilops	triuncialis	L.	918141	España	Barcelona	Plana de Vich	31TDG34	1			1877
Aegilops	triuncialis	L.	918139	España	Barcelona	Garriga	31TDG41	1			1883
Aegilops	triuncialis	L.	2513871	España	Burgos	Sotresgudo, Caserío de M	30TUN9716	0	910	CB	2006
Aegilops	triuncialis	L.	1473159	España	Burgos	Sotresgudo, Caserío de M	30TUN9716	0	910		2003
Aegilops	triuncialis	L.	2513864	España	Burgos	Sotresgudo, Caserío de M	30TUN9716	0	910		2006
Aegilops	triuncialis	L.	2513865	España	Burgos	Rebolledo de la Torre, va	30TUN9925	0	930		2006
Aegilops	triuncialis	L.	2513876	España	Burgos	Fuentenebro	30TVL3890	0	1180		2006
Aegilops	triuncialis	L.	2513866	España	Burgos	Fuentenebro, Piedemont	30TVL3890	0	1190		2006
Aegilops	triuncialis	L.	2513868	España	Burgos	Fuentenebro, Serrezuela	30TVL3890	0	1180		2006
Aegilops	triuncialis	L.	2513869	España	Burgos	Aranda de Duero, Hacia L	30TVM31	0		CB	2006
Aegilops	triuncialis	L.	94086	España	Burgos	Aranda de Duero, hacia L	30TVM31	0			1997
Aegilops	triuncialis	L.	1554591	España	Burgos	Aranda	30TVM41	1			1917
Aegilops	triuncialis	L.	2513873	España	Burgos	Burgos	30TVM48	0		CB	2006
Aegilops	triuncialis	L.	117647	España	Burgos	Burgos	30TVM48	1			1924
Aegilops	triuncialis	L.	2513875	España	Burgos	Burgos, Castañares	30TVM48	0		CB	2006
Aegilops	triuncialis	L.	117679	España	Burgos	Burgos, Castañares	30TVM48	1			1924
Aegilops	triuncialis	L.	2513874	España	Burgos	Ciruelos de Cervera, Pie d	30TVM5743	0		CB	2006
Aegilops	triuncialis	L.	1743189	España	Burgos	Ciruelos de Cervera, pie d	30TVM5743	0	1100		1980

Aegilops	triuncialis	L.	2513872	España	Burgos	Contreras	30TVM6355	0	898		2006
Aegilops	triuncialis	L.	2513863	España	Burgos	Mamolar	30TVM7243	0	1174		2006
Aegilops	triuncialis	L.	2513867	España	Burgos	Hacinas, Cunetas de la ca	30TVM7548	0	980		2006
Aegilops	triuncialis	L.	1473158	España	Burgos	Valle de Losa, Lastras de	30TVN7862	0	675		2003
Aegilops	triuncialis	L.	2513870	España	Burgos	Valle de Losa, Lastras de	30TVN7862	0	675	CB	2006
Aegilops	triuncialis	L.	2791169	España	Castellón	Baix Maestrat, Santa Mag	31TBE77		100		2006
Aegilops	triuncialis	L.	2791173	España	Castellón	Baix Maestrat, La Pobla d	31TBF6009		1080		2006
Aegilops	triuncialis	L.	1835123	España	Ciudad Real	intercalación basáltica de	30SUH48	1		CB	2005
Aegilops	triuncialis	L.	1835124	España	Ciudad Real	Piedrabuena, cunetas de	30SUJ2983	0	700	CB	2005
Aegilops	triuncialis	L.	1835127	España	Ciudad Real	Navalpino, río Valdehorn	30SUJ6147	0	540	CB	2005
Aegilops	triuncialis	L.	1835129	España	Ciudad Real	Navas de Estena, arroyo	30SUJ6172	0	780	CB	2005
Aegilops	triuncialis	L.	1348609	España	Ciudad Real	Piedrabuena, carretera e	30SUJ6737	0	700		1998
Aegilops	triuncialis	L.	1482316	España	Ciudad Real	P.N. Cabañeros	30SUJ76	1			1993
Aegilops	triuncialis	L.	1374902	España	Ciudad Real	Montes de Toledo, Parqu	30SUJ8954	0			1993
Aegilops	triuncialis	L.	1835141	España	Ciudad Real	Retuerta del Bullaque, Ca	30SUJ8954	0		CB	2005
Aegilops	triuncialis		1762694	España	Ciudad Real	Volcán de Piedrabuena	30SUJ92	0			2004
Aegilops	triuncialis	L.	1835135	España	Ciudad Real	Piedrabuena, volcán de P	30SUJ9723	0	600	CB	2005
Aegilops	triuncialis	L.	1348608	España	Ciudad Real	Piedrabuena	30SUJ9821	1	550		1998
Aegilops	triuncialis	L.	1835142	España	Ciudad Real	Villamayor de Calatrava,	30SVH0297	0	842	CB	2005
Aegilops	triuncialis	L.	1835136	España	Ciudad Real	Solana del Pino, Alhorín	30SVH0365	0	660	CB	2005
Aegilops	triuncialis		1762692	España	Ciudad Real	Morrón de Villamayor	30SVH09	0			2004
Aegilops	triuncialis	L.	1835149	España	Ciudad Real	Aldea del Rey, cerro de la	30SVH2087	0	750	CB	2005
Aegilops	triuncialis	L.	1835137	España	Ciudad Real	San Lorenzo de Calatrava	30SVH2359	0	620	CB	2005
Aegilops	triuncialis	L.	1835140	España	Ciudad Real	San Lorenzo de Calatrava	30SVH2459	0	820	CB	2005
Aegilops	triuncialis	L.	1835139	España	Ciudad Real	San Lorenzo de Calatrava	30SVH2954	0	800	CB	2005
Aegilops	triuncialis	L.	1835138	España	Ciudad Real	San Lorenzo de Calatrava	30SVH3055	0	800	CB	2005
Aegilops	triuncialis	L.	1835126	España	Ciudad Real	Manzanares, de Almagro	30SVH3799	0	700	CB	2005
Aegilops	triuncialis	L.	1835148	España	Ciudad Real	Almuradiel-Venta de Cár	30SVH55	1		CB	2005
Aegilops	triuncialis	L.	1835121	España	Ciudad Real	Despeñaperros	30SVH55	1		CB	2005
Aegilops	triuncialis	L.	1074433	España	Ciudad Real	Venta de Cárdenas	30SVH55	1			1948
Aegilops	triuncialis	L.	1835150	España	Ciudad Real	Alcubillas, alrededores	30SVH9089	0	830	CB	2005
Aegilops	triuncialis	L.	1835144	España	Ciudad Real	Villanueva de los Infantes	30SVH9583	0	820	CB	2005
Aegilops	triuncialis	L.	1835119	España	Ciudad Real	Ciudad Real	30SVJ11	1		CB	2005
Aegilops	triuncialis	L.	1869151	España	Ciudad Real	Ciudad Real, La Atalaya	30SVJ22	1	670		2004
Aegilops	triuncialis	L.	1835125	España	Ciudad Real	Malagón-Los Quiles, oliv	30SVJ2340	0		CB	2005
Aegilops	triuncialis		1762693	España	Ciudad Real	Volcán de Yezosa	30SVJ40	0			2004
Aegilops	triuncialis	L.	1835145	España	Ciudad Real	Almagro, volcán de Yezos	30SVJ4200	0	853	CB	2005
Aegilops	triuncialis	L.	1835128	España	Ciudad Real	Moral de Calatrava, sierr	30SVJ50	1		CB	2005
Aegilops	triuncialis	L.	1835120	España	Ciudad Real	Herencia, cerro Navajo, l	30SVJ6551	0	660	CB	2005

Aegilops	triuncialis	L.	1835122	España	Ciudad Real	Herencia, de Herencia a V	30SVJ6853	0	700	CB	2005
Aegilops	triuncialis	L.	1835146	España	Ciudad Real	Alhambra, saladar en arr	30SVJ9307	0		CB	2005
Aegilops	triuncialis	L.	1835143	España	Ciudad Real	Villamanrique, arroyo de	30SWH0267	0	840	CB	2005
Aegilops	triuncialis	L.	1835134	España	Ciudad Real	Puebla del Príncipe, cerca	30SWH0864	0	940	CB	2005
Aegilops	triuncialis	L.	1835147	España	Ciudad Real	Albadalejo	30SWH1674	0	900	CB	2005
Aegilops	triuncialis	L.	1835118	España	Ciudad Real	Fuencaliente, confluencia	30SWH2582	0	1000	CB	2005
Aegilops	triuncialis	L.	1835130	España	Ciudad Real	Lagunas de Ruidera, Orti	30SWJ11	1		CB	2005
Aegilops	triuncialis	L.	995301	España	Cuenca	Almonacid del Marquesa	30SWK10	1			1899
Aegilops	triuncialis		1000500	España	Cuenca	Fresneda de Allarejos	30SWK51	1			1977
Aegilops	triuncialis		1000499	España	Cuenca	Jábala, Boniches	30SXK12	0			1977
Aegilops	triuncialis	L.	2074533	España	Cuenca	Barajas de Melo	30TWK04	0			2001
Aegilops	triuncialis		1000498	España	Cuenca	Venta de Cabrejas	30TWK53	0			1977
Aegilops	triuncialis		1001681	España	Cuenca	Puerto de Cabrejas	30TWK6236	0			1978
Aegilops	triuncialis		998073	España	Cuenca	Puerto de Cabrejas a Cue	30TWK6236	0			1978
Aegilops	triuncialis		998074	España	Cuenca	Cuenca	30TWK7036	0			1978
Aegilops	triuncialis		998072	España	Cuenca	El Ventorro a Villalba	30TWK7651	0			1978
Aegilops	triuncialis		2406937	España	Cuenca	Puente Vadillos, orillas d	30TWK78	1			1942
Aegilops	triuncialis		1001948	España	Cuenca	Sierra de San Felipe	30TWK9970	0			1978
Aegilops	triuncialis		1001974	España	Cuenca	Tragacete	30TXK0066	0			1978
Aegilops	triuncialis		2242509	España	Cáceres	Valencia de Alcántara, Al	29SPD5253	0	460-862		2008
Aegilops	triuncialis		1093235	España	Cáceres	Finca de Valdelasyeguas,	29SPD96	0			1989
Aegilops	triuncialis		1093075	España	Cáceres	Ceclavín	29SPE91	1			1977
Aegilops	triuncialis		1093244	España	Cáceres	Coria	29SQE12	1			1989
Aegilops	triuncialis		2242510	España	Cáceres	La Nava, Berzocana	30STJ8664	0	645-783		2008
Aegilops	triuncialis		2242511	España	Cáceres	La Nava, Berzocana	30STJ8664	0	640		2008
Aegilops	triuncialis	L.	1714999	España	Cáceres	Sierra las Corchuelas, Par	30STK51	1			1986
Aegilops	triuncialis		1093226	España	Cáceres	Toril	30STK6220	0			1987
Aegilops	triuncialis		1093251	España	Cáceres	Almaraz	30STK70	0			1989
Aegilops	triuncialis		1093250	España	Cáceres	Almaraz	30STK70	0			1989
Aegilops	triuncialis	L.	1091708	España	Cáceres	Alía	30SUJ06	1			1952
Aegilops	triuncialis	L.	1091709	España	Cáceres	Río Guadarranque	30SUJ16	1			1952
Aegilops	triuncialis	L.	1093264	España	Cáceres	Comarca de la Vera	30TTK63	0			1993
Aegilops	triuncialis		1093261	España	Cáceres	Valverde de la Vera, La V	30TTK9241	0			1993
Aegilops	triuncialis		2242512	España	Cádiz	Jerez de la Frontera, Casa	29SQA56	1			1997
Aegilops	triuncialis	L.	1684422	España	Cádiz	Sierra del Pinar, ladera N	30STF8371	0			1978
Aegilops	triuncialis	L.	1684423	España	Cádiz	Sierra de Zafalgar, La Car	30STF8575	0			1978
Aegilops	triuncialis	L.	2309234	España	Córdoba	Fuenteovejuna, compren	30STH83	1			1986
Aegilops	triuncialis	L.	2309237	España	Córdoba	Sierra Morena, NE, N de	30STH94	1			1986
Aegilops	triuncialis	L.	2309238	España	Córdoba	Sierra Morena, NW, pobl	30STH94	1			1986

Aegilops	triuncialis	L.	2309236	España	Córdoba	Sierra Morena, SE, límite	30STH94	1			1986
Aegilops	triuncialis	L.	2309235	España	Córdoba	Sierra Morena, SW, desd	30STH94	1			1986
Aegilops	triuncialis	L.	2309239	España	Córdoba	Mármol, estación del Má	30STH96	1			1986
Aegilops	triuncialis		1073155	España	Córdoba	Puente Genil	30SUG44	1			1986
Aegilops	triuncialis	L.	2309240	España	Córdoba	Campiña Cordobesa, S de	30SUG49	1			1986
Aegilops	triuncialis		1074126	España	Córdoba	Doña Mencia	30SUG75	1			1986
Aegilops	triuncialis	L.	2309241	España	Córdoba	Campiña de Bujalance, té	30SUG79	1			1986
Aegilops	triuncialis		1073154	España	Córdoba	Baena	30SUG8066	0			1986
Aegilops	triuncialis	L.	2309242	España	Córdoba	Iznájar, S de Priego de Có	30SUG82	1			1986
Aegilops	triuncialis	L.	2309243	España	Córdoba	Subbético, comprende Co	30SUG95	1			1986
Aegilops	triuncialis	L.	2309244	España	Córdoba	Hinojosa del Duque, part	30SUH16	1			1986
Aegilops	triuncialis	L.	2309245	España	Córdoba	Belalcázar, estación, entr	30SUH28	1			1986
Aegilops	triuncialis	L.	2326225	España	Córdoba	Batolito de los Pedroches	30SUH73	0			1978
Aegilops	triuncialis	L.	2326223	España	Córdoba	Batolito de los Pedroches	30SUH74	0			1978
Aegilops	triuncialis	L.	2326224	España	Córdoba	Batolito de los Pedroches	30SUH83	0			1978
Aegilops	triuncialis	L.	2309246	España	Córdoba	Cardeña, Valle de los Ped	30SUH83	1			1986
Aegilops	triuncialis	L.	2745526	España	ESPAÑA	Sierra de Mariola	30SYH19	1		CB	2012
Aegilops	triuncialis	L.	2242515	España	Granada	Arenas del Rey	30SVF2284	0			2005
Aegilops	triuncialis	L.	2242516	España	Granada	Almuñécar	30SVF3173	0			2005
Aegilops	triuncialis	L.	1019469	España	Granada	Pinos Genil	30SVG51	1			1975
Aegilops	triuncialis	L.	1487852	España	Granada	P.N. Sierra Nevada	30SVG70	1			1987
Aegilops	triuncialis	L.	1376738	España	Granada	Sierra de Baza, Parque Na	30SWG13	1			1991
Aegilops	triuncialis		2648898	España	Guadalajara	Alpedrete de la Sierra	30TVL62	1	900		1985
Aegilops	triuncialis		2648894	España	Guadalajara	Pontón de la Oliva	30TVL62	1	830		1985
Aegilops	triuncialis		2648893	España	Guadalajara	Pontón de la Oliva	30TVL62	1	800		1985
Aegilops	triuncialis		2648892	España	Guadalajara	Valdepeñas de la Sierra	30TVL62	1	730		1985
Aegilops	triuncialis		2648897	España	Guadalajara	Valdepeñas de la Sierra	30TVL62	1	870		1985
Aegilops	triuncialis		993577	España	Guadalajara	Tamajón	30TVL73	0			1977
Aegilops	triuncialis		2648891	España	Guadalajara	Tamajón	30TVL73	1	1000		1985
Aegilops	triuncialis		2648895	España	Guadalajara	Torrebeleña	30TVL82	1	830		1985
Aegilops	triuncialis		2728717	España	Guadalajara	Aleas	30TVL83	1	860		1998
Aegilops	triuncialis		2442833	España	Guadalajara	Pareja	30TWK28	0	800		1984
Aegilops	triuncialis		2442830	España	Guadalajara	Pareja	30TWK28	0	750		1984
Aegilops	triuncialis		2442834	España	Guadalajara	Chillarón del Rey	30TWK29	1	780		1984
Aegilops	triuncialis		2442827	España	Guadalajara	Alcocer	30TWK38	0	820		1984
Aegilops	triuncialis	L.	2442823	España	Guadalajara	Alcocer	30TWK38	0			1984
Aegilops	triuncialis		2442828	España	Guadalajara	Alcocer	30TWK38	0	800		1984
Aegilops	triuncialis		2442824	España	Guadalajara	Alique	30TWK39	0	930		1984
Aegilops	triuncialis		2442829	España	Guadalajara	Alique	30TWK39	0	850		1984

Aegilops	triuncialis	L.	2442831	España	Guadalajara	Hontanilla	30TWK39	1			1984
Aegilops	triuncialis		2442825	España	Guadalajara	Salmerón	30TWK48	0	800		1984
Aegilops	triuncialis	L.	2442836	España	Guadalajara	El Recuenco	30TWK59	0			1984
Aegilops	triuncialis		2728715	España	Guadalajara	Atienza	30TWL16	1	1160		1998
Aegilops	triuncialis		2728716	España	Guadalajara	Entre Cirueches y Carabia	30TWL24	1	960		1998
Aegilops	triuncialis		2728714	España	Guadalajara	Riba de Santiuste	30TWL26	1	1000		1998
Aegilops	triuncialis		2442835	España	Guadalajara	Azañón	30TWL30	0	840		1984
Aegilops	triuncialis		2442826	España	Guadalajara	Villanueva de Alcorón	30TWL60	0	1260		1984
Aegilops	triuncialis	L.	992691	España	Guadalajara	Mazarete	30TWL63	1			1948
Aegilops	triuncialis	L.	992692	España	Guadalajara	Turmiel	30TWL74	1			1948
Aegilops	triuncialis	L.	1065853	España	Huelva	Sierra de Aracena	29SQB09	0			1985
Aegilops	triuncialis	L.	1065852	España	Huelva	Sierra de Aracena	29SQB19	0			1985
Aegilops	triuncialis	L.	1062080	España	Huelva	Sierra de Aracena	29SQB29	0			1985
Aegilops	triuncialis	L.	1774963	España	Huesca	Almunia de San Juan, Ari	31TBG6649	0	265		2004
Aegilops	triuncialis	L.	1774964	España	Huesca	Almunia de San Juan, Ari	31TBG6650	0	270		2004
Aegilops	triuncialis	L.	1774965	España	Huesca	Monzón, El Regal, Torre (	31TBG6845	0	310		2004
Aegilops	triuncialis	L.	1714167	España	Islas Baleares	Eivissa, Sant Carles, prop	31SCD7220	0	90		2003
Aegilops	triuncialis	L.	1027218	España	Jaén	Marmolejo	30SUH8850	0	650		1994
Aegilops	triuncialis	L.	1027219	España	Jaén	Marmolejo	30SUH9444	0	500		1994
Aegilops	triuncialis		1025030	España	Jaén	Alcaudete	30SVG06	0			1986
Aegilops	triuncialis		1025031	España	Jaén	E. Martos y Alcaudete	30SVG17	1			1986
Aegilops	triuncialis		2678485	España	Jaén	Los Villares, El Peralejo	30SVG27	1	1200		2000
Aegilops	triuncialis		2242513	España	Jaén	Los Villares, Puerto Viejo	30SVG27	1	1300		2000
Aegilops	triuncialis		2678486	España	Jaén	Los Villares, Peña El Altar	30SVG36	1	1550		2000
Aegilops	triuncialis	L.	1030246	España	Jaén	Noalejo, Santa Mercé	30SVG4053	0	1100		1996
Aegilops	triuncialis	L.	1030247	España	Jaén	Campillo de Arenas, Cerr	30SVG4160	0	1300		1996
Aegilops	triuncialis	L.	1027220	España	Jaén	Marmolejo	30SVH0049	0	650		1994
Aegilops	triuncialis	L.	1332457	España	Jaén	Río de la Cabrera	30SVH0437	0			1996
Aegilops	triuncialis	L.	1024008	España	Jaén	Despeñaperros	30SVH54	1			1948
Aegilops	triuncialis		2488826	España	Jaén	Sierra de Segura, Los Are	30SWG1496	0	1400		2006
Aegilops	triuncialis		2713465	España	Jaén	Cortijos Nuevos, El Conta	30SWH23	1			2012
Aegilops	triuncialis	L.	2396169	España	La Coruña	Ferrol	29TNJ61	1		CB	2006
Aegilops	triuncialis	L.	2364345	España	La Coruña	Gallec. pr el Ferrol	29TNJ61	1			1861-1862
Aegilops	triuncialis		1551179	España	La Coruña	boreali rara (Gallec. pr. e	29TNJ61	1			1861
Aegilops	triuncialis		1551207	España	León	regno Legion. (Villafranca	29TPH71	1			1861
Aegilops	triuncialis	L.	2364341	España	León	regno Legion., Villafranca	29TPH71	1			1861-1862
Aegilops	triuncialis	L.	1141568	España	León	Villamañán	30TTM89	1	760		1984
Aegilops	triuncialis	L.	1135163	España	León	Castrofuerte	30TTM97	1	743		1984
Aegilops	triuncialis	L.	1135155	España	León	Chozas de Abajo	30TTN71	1			1986

Aegilops	triuncialis	L.	1141562	España	León	Ardoncino	30TTN80	1	830		1984
Aegilops	triuncialis	L.	1554592	España	León	León	30TTN81	1			1917
Aegilops	triuncialis	L.	118439	España	León	La Pola de Gordón, Santa	30TTN85	0			1985
Aegilops	triuncialis	L.	1135158	España	León	Ardón	30TTN90	1	820		1984
Aegilops	triuncialis	L.	1135164	España	León	Puente Castro	30TTN91	1	910		1984
Aegilops	triuncialis	L.	1141592	España	León	Puente Villarente	30TTN91	1	754		1984
Aegilops	triuncialis	L.	1554593	España	León	pte. de Castro	30TTN91	1			1917
Aegilops	triuncialis	L.	1135156	España	León	Castrovega de Valmadrig	30TUM18	1			1986
Aegilops	triuncialis	L.	2396168	España	Lugo	Quiroga, Montefurado, p	29TPG4795	0	300		2006
Aegilops	triuncialis	L.	1463956	España	Lérida	Congost de Santa Anna	31TBG93	0	320		2001
Aegilops	triuncialis		2656318	España	Lérida	l'Espluga Calba	31TCF39	1			1987-88
Aegilops	triuncialis	L.	1463957	España	Lérida	Gerb	31TCG13	0	260		2001
Aegilops	triuncialis	L.	1463959	España	Lérida	Gros, tossal	31TCG13	0	420		2001
Aegilops	triuncialis	L.	1463958	España	Lérida	Les Planes de Castelló de	31TCG13	0	360		2001
Aegilops	triuncialis	L.	1463960	España	Lérida	Les Avellanes	31TCG14	0	600		2001
Aegilops	triuncialis		1085281	España	Madrid	Villanueva del Pardillo	30TVK18	1			1977
Aegilops	triuncialis		1085278	España	Madrid	Villaviciosa de Odón	30TVK26	1			1977
Aegilops	triuncialis		1085277	España	Madrid	Río Guadarrama	30TVK27	1			1977
Aegilops	triuncialis		1090319	España	Madrid	Majadahonda	30TVK28	0			1977
Aegilops	triuncialis		1085282	España	Madrid	Hoyo de Manzanares	30TVK29	1			1977
Aegilops	triuncialis		1085274	España	Madrid	Húmera	30TVK37	0			1977
Aegilops	triuncialis	L.	1340022	España	Madrid	Madrid, Campus Universi	30TVK37	0			1993
Aegilops	triuncialis	L.	2786979	España	Madrid	Cerca de Madrid	30TVK47	1			1861
Aegilops	triuncialis		1085539	España	Madrid	Ciudad Universitaria	30TVK47	0			1977
Aegilops	triuncialis	L.	2364346	España	Madrid	utraque Castella, Madriti	30TVK47	1			1861-1862
Aegilops	triuncialis	L.	2364347	España	Madrid	utraque Castella, el Retir	30TVK47	1			1861-1862
Aegilops	triuncialis		1085280	España	Madrid	Alcobendas	30TVK48	1			1977
Aegilops	triuncialis		1085273	España	Madrid	Tres Cantos	30TVK49	1			1977
Aegilops	triuncialis	L.	1084636	España	Madrid	Vaciamadrid	30TVK57	1			1972
Aegilops	triuncialis		1085540	España	Madrid	Ajalvir	30TVK58	0			1977
Aegilops	triuncialis		1085279	España	Madrid	Paracuellos del Jarama	30TVK58	0			1977
Aegilops	triuncialis		1090390	España	Madrid	Cobeña	30TVK59	0			1977
Aegilops	triuncialis		1090314	España	Madrid	Cobeña	30TVK59	0			1977
Aegilops	triuncialis		1085538	España	Madrid	Loeches	30TVK67	1			1977
Aegilops	triuncialis	L.	2086277	España	Madrid	Alcalá de Henares	30TVK68	1			1959
Aegilops	triuncialis	L.	2364344	España	Madrid	utraque Castella, Guadar	30TVL00	1			1861-1862
Aegilops	triuncialis		2749740	España	Madrid	Oteruelo del Valle	30TVL2629		1140		1988
Aegilops	triuncialis		2749741	España	Madrid	Rascafría - Oteruelo del V	30TVL2630		1160		1988
Aegilops	triuncialis		2749743	España	Madrid	Oteruelo del Valle	30TVL2729		1140		1988

Aegilops	triuncialis		2749744	España	Madrid	Oteruelo del Valle	30TVL2729		1140		1988
Aegilops	triuncialis		2749745	España	Madrid	Rascafría - Oteruelo del V	30TVL2729		1140		1988
Aegilops	triuncialis		2749746	España	Madrid	Rascafría - Oteruelo del V	30TVL2729		1140		1988
Aegilops	triuncialis		2749742	España	Madrid	Rascafría - Oteruelo del V	30TVL2729		1140		1988
Aegilops	triuncialis		2749748	España	Madrid	Alameda del Valle	30TVL2830		1130		1988
Aegilops	triuncialis		2749749	España	Madrid	Alameda del Valle	30TVL2830		1130		1988
Aegilops	triuncialis		2749747	España	Madrid	Alameda del Valle	30TVL2830		1130		1988
Aegilops	triuncialis		1085275	España	Madrid	Colmenar Viejo	30TVL30	1			1977
Aegilops	triuncialis		2749750	España	Madrid	Cerros calizos de Pinilla d	30TVL3030		1110		1988
Aegilops	triuncialis		2749751	España	Madrid	Cruce de Pinilla del Valle	30TVL3031		1100		1988
Aegilops	triuncialis		2749753	España	Madrid	Cerros calizos de Pinilla d	30TVL3231		1110		1988
Aegilops	triuncialis		2749752	España	Madrid	Pinilla del Valle, cerro cal	30TVL3231		1110		1988
Aegilops	triuncialis	L.	1370912	España	Madrid	Guadalix de la Sierra	30TVL3912	0			1997
Aegilops	triuncialis		2749754	España	Madrid	Buitrago - Villavieja de Lo	30TVL3934		1120		1988
Aegilops	triuncialis		2749755	España	Madrid	Buitrago de Lozoya	30TVL4638		980		1988
Aegilops	triuncialis		2749757	España	Madrid	Solanas de Lozoyuela	30TVL4731		1060		1988
Aegilops	triuncialis		2749756	España	Madrid	Solanas de Lozoyuela	30TVL4731		1060		1988
Aegilops	triuncialis	L.	1370916	España	Madrid	Redueña	30TVL5016	0			1997
Aegilops	triuncialis	L.	1370914	España	Madrid	El Molar	30TVL5109	0			1997
Aegilops	triuncialis		2648896	España	Madrid	Torrelaguna	30TVL52	1	830		1985
Aegilops	triuncialis		1085276	España	Madrid	Ribatejada	30TVL60	1			1977
Aegilops	triuncialis		993535	España	Madrid	Valdepiélagos	30TVL61	1			1977
Aegilops	triuncialis	L.	1475537	España	Murcia	Sierra del Ricote	30SXH32	0			1985
Aegilops	triuncialis	L.	2242517	España	Málaga	Jimera de Líbar	30STF95	1			2007
Aegilops	triuncialis	L.	1043775	España	Málaga	Estepona, Sierra Bermeja	30SUF03	0			1990
Aegilops	triuncialis		2242507	España	Málaga	Ronda, proximidades del	30SUF16	1	1150		1998
Aegilops	triuncialis		2242508	España	Málaga	Tolox, entre Los Pilones y	30SUF16	1	1720		1998
Aegilops	triuncialis	L.	1043779	España	Málaga	Ronda, Sierra de las Niev	30SUF17	1			1990
Aegilops	triuncialis	L.	1043780	España	Málaga	San Pedro de Alcántara	30SUF24	0			1990
Aegilops	triuncialis	L.	1043777	España	Málaga	Marbella	30SUF34	0			1990
Aegilops	triuncialis	L.	1043778	España	Málaga	Ojén, Sierra Blanca	30SUF34	0			1990
Aegilops	triuncialis	L.	1043773	España	Málaga	Carratraca, Sierra de Agu	30SUF37	0			1990
Aegilops	triuncialis		1035842	España	Málaga	Sierra de Aguas, Arroyo d	30SUF38	0			1975
Aegilops	triuncialis	L.	1036001	España	Málaga	Sierra de Aguas, Carratra	30SUF38	0			1975
Aegilops	triuncialis		1035682	España	Málaga	Sierra de Aguas, Carratra	30SUF38	0	500		1975
Aegilops	triuncialis		2101954	España	Málaga	Sierra del Agua de Carrat	30SUF38	1	500-750		1969
Aegilops	triuncialis		1035582	España	Málaga	Zonas elevadas de la Sier	30SUF38	0			1975
Aegilops	triuncialis	L.	1043774	España	Málaga	Coín	30SUF45	0			1990
Aegilops	triuncialis	L.	1043776	España	Málaga	Málaga	30SUF76	0			1990

Aegilops	triuncialis	L.	1038227	España	Málaga	Puerto de las Pedrizas	30SUF79	1			1936
Aegilops	triuncialis	L.	1043771	España	Málaga	Archidona	30SUG70	0			1990
Aegilops	triuncialis	L.	2242514	España	Málaga	Sedella	30SVF0681	0			2005
Aegilops	triuncialis	L.	1036796	España	Málaga	Sierra Tejeda	30SVF08	1			1948
Aegilops	triuncialis	L.	1043781	España	Málaga	Sierra Tejeda	30SVF08	0			1990
Aegilops	triuncialis	L.	1038226	España	Málaga	Canillas de Albaidia	30SVF17	1			1933
Aegilops	triuncialis	L.	1043772	España	Málaga	Canillas de Albaida	30SVF18	0			1990
Aegilops	triuncialis	L.	1036795	España	Málaga	Sierra Almijara	30SVF27	1			1948
Aegilops	triuncialis	L.	983878	España	Navarra	Mañeru	30TWN92	1			1973
Aegilops	triuncialis	L.	1630735	España	Orense	Río Miño frente a Barban	29TNG78	1			1909
Aegilops	triuncialis	L.	2425009	España	Orense	Rubiana, por encima de V	29TPH70	1	750		1967
Aegilops	triuncialis	L.	1377177	España	Orense	Vilardesilva	29TPH7903	0	450		1996
Aegilops	triuncialis		1557995	España	Palencia	Soto de Cerrato	30TUM7549	0	850		1983
Aegilops	triuncialis	L.	104260	España	Palencia	Castrejón de la Peña, ent	30TUN64	1			1957
Aegilops	triuncialis		2079077	España	Palencia	Villanueva de la Peña	30TUN64	1			1956
Aegilops	triuncialis	L.	56043	España	Palencia	Castrillo de Don Juan	30TVM12	1			1985
Aegilops	triuncialis	L.	62022	España	Salamanca	La Bouza	29TPF82	1			1978
Aegilops	triuncialis	L.	51168	España	Salamanca	La Fregeneda	29TPF83	1			1979
Aegilops	triuncialis	L.	51169	España	Salamanca	San Felices	29TPF92	0			1979
Aegilops	triuncialis	L.	62024	España	Salamanca	Pastores, Cabezal Viejo	29TQE18	1			1978
Aegilops	triuncialis	L.	51170	España	Salamanca	Masueco	29TQF06	0			1979
Aegilops	triuncialis	L.	51242	España	Salamanca	Villarino	29TQF17	1			1979
Aegilops	triuncialis	L.	1109626	España	Salamanca	Doñinos de Salamanca	29TQF44	0			1996
Aegilops	triuncialis	L.	74649	España	Salamanca	Montemayor del río	30TTK57	1			1985
Aegilops	triuncialis	L.	60945	España	Salamanca	San Esteban de la Sierra	30TTK58	1			1979
Aegilops	triuncialis	L.	61255	España	Salamanca	Béjar	30TTK67	1			1975
Aegilops	triuncialis	L.	65366	España	Salamanca	Guijuelo	30TTK79	1			1989
Aegilops	triuncialis		2117645	España	Salamanca	Puente del Congosto	30TTK8483	0	1000		2004
Aegilops	triuncialis	L.	2130749	España	Salamanca	Navamorales	30TTK9084	0	1010		2004
Aegilops	triuncialis	L.	2130750	España	Salamanca	Navamorales	30TTK9084	0	1010		2004
Aegilops	triuncialis	L.	83631	España	Salamanca	Navamorales	30TTK98	1			1994
Aegilops	triuncialis	L.	57499	España	Salamanca	Matilla de los Caños del r	30TTL52	1			1984
Aegilops	triuncialis	L.	72902	España	Salamanca	Doñinos de Salamanca	30TTL63	1			1986
Aegilops	triuncialis	L.	51243	España	Salamanca	Almenara de Tormes, tes	30TTL65	1			1979
Aegilops	triuncialis	L.	52932	España	Salamanca	Arapiles, borde de la carr	30TTL73	1			1984
Aegilops	triuncialis	L.	72901	España	Salamanca	Salamanca, Tejares	30TTL73	0			1986
Aegilops	triuncialis	L.	70872	España	Salamanca	Monterrubio de Armuña	30TTL74	1			1976
Aegilops	triuncialis	L.	59598	España	Salamanca	Monterrubio de Armuña	30TTL74	1			1991
Aegilops	triuncialis	L.	70873	España	Salamanca	San Cristóbal de la Cuesta	30TTL74	1			1976

Aegilops	triuncialis	L.	78965	España	Salamanca	Cabrerizos	30TTL83	1			1977
Aegilops	triuncialis	L.	1109627	España	Salamanca	Aldeanueva de Figueroa	30TTL85	1			1996
Aegilops	triuncialis	L.	58156	España	Salamanca	Babilafuente	30TTL93	1			1977
Aegilops	triuncialis	L.	54855	España	Salamanca	Bóveda del río Almar	30TUL12	1			1983
Aegilops	triuncialis	L.	58085	España	Salamanca	Cantalapiedra	30TUL15	1			1987
Aegilops	triuncialis	L.	80277	España	Segovia	Cantalejo, La Muña	30TVL17	1			1985
Aegilops	triuncialis	L.	92444	España	Segovia	Sacramenia, Convento de	30TVL2294	0			1989
Aegilops	triuncialis	L.	70819	España	Segovia	Santiuste de Pedraza	30TVL2850	0			1995
Aegilops	triuncialis	L.	80853	España	Segovia	Sepúlveda, Villar de Sobr	30TVL3571	0			1985
Aegilops	triuncialis	L.	80852	España	Segovia	Sepúlveda, Sepúlveda	30TVL3671	0			1985
Aegilops	triuncialis	L.	120462	España	Segovia	Valdevacas de Montejo, V	30TVL4699	0			1996
Aegilops	triuncialis	L.	120463	España	Segovia	Valdevacas de Montejo, V	30TVL4898	0			1996
Aegilops	triuncialis	L.	2325587	España	Sevilla	El Garrobo	29SQB46	1			1978
Aegilops	triuncialis	L.	2342899	España	Sevilla	El Garrobo	29SQB46	1		CB	1999
Aegilops	triuncialis		1067622	España	Sevilla	Puebla del Río	29SQB62	1			1955
Aegilops	triuncialis		1067623	España	Sevilla	Coria del Río	29SQB63	1			1955
Aegilops	triuncialis	L.	1067628	España	Sevilla	Sevilla	30STG44	1			1897
Aegilops	triuncialis	L.	1066941	España	Sevilla	Paradas	30STG72	1			1948
Aegilops	triuncialis	L.	1072126	España	Sevilla	Arroyo del Salado, cerca	30STG80	0			1984
Aegilops	triuncialis	L.	1072127	España	Sevilla	Morón de la Frontera, alr	30STG81	0			1984
Aegilops	triuncialis	L.	1072125	España	Sevilla	Las Rozas, Encarnaciones	30STG90	0			1984
Aegilops	triuncialis	L.	1067630	España	Sevilla	Pruna, Algámitas, Sierra (	30SUF09	0			1984
Aegilops	triuncialis	L.	1369175	España	Soria	Olmillos	30TVL89	0	860		1998
Aegilops	triuncialis	L.	1369177	España	Soria	Osma	30TVM90	0			1998
Aegilops	triuncialis	L.	1369179	España	Soria	Ucero	30TVM91	0	1000		1998
Aegilops	triuncialis	L.	1369181	España	Soria	Casarejos	30TVM92	0			1998
Aegilops	triuncialis	L.	85961	España	Soria	Casarejos	30TVM92	0			1987
Aegilops	triuncialis	L.	1369183	España	Soria	Berlanga de Duero, hacia	30TWL09	0	880		1998
Aegilops	triuncialis	L.	101227	España	Soria	Quintanas de Gormaz, Ha	30TWL09	0	950		1996
Aegilops	triuncialis	L.	1369185	España	Soria	Medinaceli	30TWL45	0			1998
Aegilops	triuncialis	L.	1369187	España	Soria	Lodares de Medinaceli	30TWL56	0	900		1998
Aegilops	triuncialis	L.	987988	España	Soria	Cañamaque	30TWL68	1			1942
Aegilops	triuncialis	L.	1369189	España	Soria	Cañamaque	30TWL68	0			1998
Aegilops	triuncialis	L.	1369191	España	Soria	Valdenarros	30TWM00	0	950		1998
Aegilops	triuncialis	L.	1369193	España	Soria	Los Rábanos, La Carrasco	30TWM41	0	1000		1998
Aegilops	triuncialis	L.	1369195	España	Soria	Soria	30TWM42	0			1998
Aegilops	triuncialis	L.	1369197	España	Soria	Aldehuela de Periáñez, h	30TWM52	0	1050		1998
Aegilops	triuncialis	L.	86604	España	Soria	Aldehuela de Periáñez, h	30TWM52	0			1982
Aegilops	triuncialis	L.	1369199	España	Soria	La Rubia	30TWM53	0	1100		1998

Aegilops	triuncialis	L.	2791170	España	Tarragona	Montsià, Ulldecona, Cam	31TBE89		240		2006
Aegilops	triuncialis	L.	2791172	España	Tarragona	Montsià, Alcanar, Platja d	31TBE99		3		2006
Aegilops	triuncialis	L.	2791171	España	Tarragona	Montsià, Alcanar, Platja d	31TBE99		3		2006
Aegilops	triuncialis	L.	2791174	España	Tarragona	Montsià, Santa Bàrbara,	31TBF81		70		2006
Aegilops	triuncialis	L.	930415	España	Tarragona	L'Aleixar, Vilaplana del Ca	31TCF36	1			1972
Aegilops	triuncialis	L.	930418	España	Tarragona	Valle de Castellfollit	31TCF38	1			1972
Aegilops	triuncialis	L.	930417	España	Tarragona	Vimbodí, Vallclara, La Fel	31TCF38	1			1972
Aegilops	triuncialis	L.	930416	España	Tarragona	L'Espluga de Francolí	31TCF48	1			1972
Aegilops	triuncialis	L.	931851	España	Tarragona	Tarragona	31TCF55	1			1908
Aegilops	triuncialis	L.	930419	España	Tarragona	Plans de Prades, Plans Pa	31TCF57	1			1972
Aegilops	triuncialis	L.	1077310	España	Toledo	Polan, Ventosilla	30SVK00	1			1882
Aegilops	triuncialis		1674410	España	Toledo	Talavera, carretera a Seg	30TUK4431	0	440		2004
Aegilops	triuncialis		1674407	España	Toledo	San Román	30TUK5237	0	480		2004
Aegilops	triuncialis		1674406	España	Toledo	Cazalegas	30TUK5630	0	440		2004
Aegilops	triuncialis		1674405	España	Toledo	Lucillos, Los Nogales	30TUK6131	0	350		2004
Aegilops	triuncialis		1674409	España	Toledo	Lucillos, hacia Cardiel de	30TUK6231	0	350		2004
Aegilops	triuncialis		1674408	España	Toledo	Lucillos, Arroyo de Venta	30TUK6331	0	450		2004
Aegilops	triuncialis	L.	847028	España	Valencia	Valle d'Aguies Vives	30SYJ23	1			1951
Aegilops	triuncialis	L.	47603	España	Valladolid	Castronuño, Las Cocinera	30TUL18	1			1988
Aegilops	triuncialis	L.	56193	España	Valladolid	Bocigas	30TUL56	1			1908
Aegilops	triuncialis	L.	56195	España	Valladolid	Olmedo	30TUL57	1			1908
Aegilops	triuncialis	L.	116425	España	Valladolid	Almenara de Adaja	30TUL6163	0			1987
Aegilops	triuncialis	L.	56192	España	Valladolid	Aguasal	30TUL67	1			1908
Aegilops	triuncialis	L.	56194	España	Valladolid	Pedrajas de San Esteban	30TUL67	1			1908
Aegilops	triuncialis	L.	78798	España	Valladolid	Cogeces de Íscar	30TUL78	1			1983
Aegilops	triuncialis	L.	78796	España	Valladolid	Peñaflor de Hornija	30TUM31	1			1983
Aegilops	triuncialis	L.	2364343	España	Valladolid	utraque Castella, Vallado	30TUM51	1			1861-1862
Aegilops	triuncialis	L.	2144247	España	Valladolid	Renedo de Esgueva	30TUM61	0			2006
Aegilops	triuncialis	L.	78797	España	Valladolid	Quintanilla de Arriba	30TUM90	1			1983
Aegilops	triuncialis	L.	92445	España	Valladolid	Rábano	30TVL1297	0			1989
Aegilops	triuncialis	L.	56042	España	Valladolid	Encinas de Esgueva, San .	30TVM02	1			1985
Aegilops	triuncialis		1551192	España	Vizcaya	Cantabr. (pr. Durango)	30TWN27	1			1861
Aegilops	triuncialis	L.	2364342	España	Vizcaya	Cantabr., pr. Durango	30TWN27	1			1861-1862
Aegilops	triuncialis	L.	77473	España	Zamora	Puebla de Sanabria	29TPG95	1			1986
Aegilops	triuncialis	L.	96899	España	Zamora	Fermoselle	29TQF17	1			1983
Aegilops	triuncialis	L.	96898	España	Zamora	Villadepera	29TQG30	1			1983
Aegilops	triuncialis	L.	76093	España	Zamora	Venialbo	30TTL88	1			1984
Aegilops	triuncialis	L.	99837	España	Zamora	Pozuelo de Tábara	30TTM53	1			1982
Aegilops	triuncialis	L.	1115798	España	Zamora	Pozuelo de Tábara	30TTM53	1			1984

Aegilops	triuncialis	L.	99838	España	Zamora	Perilla de Castro, Puente	30TTM62	1			1982
Aegilops	triuncialis	L.	1115795	España	Zamora	Dehesa de Misleo	30TTM63	1			1984
Aegilops	triuncialis	L.	1115802	España	Zamora	Faramontanos de Tábara	30TTM63	0			1984
Aegilops	triuncialis	L.	1115794	España	Zamora	Faramontanos de Tábara	30TTM63	0			1984
Aegilops	triuncialis	L.	1120227	España	Zamora	Granja de Moreruela, Los	30TTM63	1			1994
Aegilops	triuncialis	L.	1115797	España	Zamora	Moreruela de Tábara	30TTM63	1			1984
Aegilops	triuncialis	L.	1120228	España	Zamora	Algodre, Lagunas de Algo	30TTM80	1			1994
Aegilops	triuncialis	L.	1120226	España	Zamora	Abezames, Gafos	30TTM9911	0			1994
Aegilops	triuncialis	L.	76092	España	Zamora	Castrillo de la Guareña	30TUL06	1			1984
Aegilops	triuncialis	L.	76094	España	Zamora	Cañizal	30TUL06	1			1984
Aegilops	triuncialis	L.	971339	España	Zaragoza	Calatayud	30TXL17	1			1911
Aegilops	triuncialis	L.	2784452	España	Zaragoza	Cuneta de la carretera en	30TXL1938	0			1983
Aegilops	triuncialis	L.	1925491	España	Zaragoza	Sigüés, Venta Carrica	30TXN6320	0	550		2001
Aegilops	triuncialis		2615128	España	Zaragoza	Chiprana	30TYL47	1			1997
Aegilops	triuncialis	L.	1895399	España	Álava	Vitoria, Gasteiz, polígono	30TWN2846	0	500		1993
Aegilops	triuncialis	L.	36853	España	Álava	Elciego	30TWN30	0	450		1985
Aegilops	triuncialis		2117782	España	Ávila	Navamorales	30TTK9084	0	1010		2004
Aegilops	triuncialis	L.	102487	España	Ávila	Salobral	30TUK49	0			1989
Aegilops	triuncialis	L.	89108	España	Ávila	El Tiemblo, Valle de Iruel	30TUK77	1	1500		1992
Aegilops	triuncialis		2081589	España	Ávila	Sierra de Malagón, falda	30TUK89	1	1400		1956
Aegilops	triuncialis	L.	1111151	España	Ávila	Fuentesclaras de Arriba	30TUL50	1			1935
Aegilops	ventricosa	Tsch.	2364348	España	Albacete	Cast. nova, c. Alcaraz	30SWH48	1			1861-1862
Aegilops	ventricosa		2200322	España	Alicante	Provincia de Alicante [sin	30SYH4689	0		CB	2007
Aegilops	ventricosa		2200323	España	Alicante	Provincia de Alicante	30SYH48	0			2007
Aegilops	ventricosa		2200324	España	Alicante	Provincia de Alicante	30SYH49	0			2007
Aegilops	ventricosa	Tausch	2513877	España	Burgos	Sotresgudo, Caserío de M	30TUN9716	0	910		2006
Aegilops	ventricosa	Tausch	2513881	España	Burgos	Sotresgudo, Caserío de M	30TUN9716	0	910	CB	2006
Aegilops	ventricosa	Tausch	1473161	España	Burgos	Sotresgudo, Caserío de M	30TUN9716	0	910		2003
Aegilops	ventricosa	Tausch	2513879	España	Burgos	Tórtoles de Esgueva, entr	30TVM1329	0	860		2006
Aegilops	ventricosa	Tausch	2513886	España	Burgos	Burgos capital, inmediaci	30TVM3890	0	850		2006
Aegilops	ventricosa	Tausch	2513882	España	Burgos	Burgos	30TVM48	0		CB	2006
Aegilops	ventricosa	Tausch	117643	España	Burgos	Burgos	30TVM48	1			1924
Aegilops	ventricosa	Tausch	2513885	España	Burgos	Neila, Hacia Huerta de Ar	30TVM9956	0	1220		2006
Aegilops	ventricosa	Tausch	2513878	España	Burgos	Basconcillos del Tozo, Ho	30TVN2328	0	900		2006
Aegilops	ventricosa	Tausch	1336388	España	Burgos	Tubilla del Agua	30TVN3528	0			1990
Aegilops	ventricosa	Tausch	2513883	España	Burgos	Tubilla del Agua	30TVN3528	0		CB	2006
Aegilops	ventricosa	Tausch	1473160	España	Burgos	Merindad de Sotoscueva	30TVN5062	0	640		2003
Aegilops	ventricosa	Tausch	2513880	España	Burgos	Merindad de Sotoscueva	30TVN5064	0	640	CB	2006
Aegilops	ventricosa	Tausch	1897919	España	Burgos	Junta de Traslaloma, Villa	30TVN6763	0	720		1997

Aegilops	ventricosa	Tausch	2513887	España	Burgos	Junta de Traslaloma, Villa	30TVN6763	0	720	CB	2006
Aegilops	ventricosa	Tausch	1340285	España	Burgos	Frías	30TVN7534	0	550		1997
Aegilops	ventricosa	Tausch	2513888	España	Burgos	Frías	30TVN7534	0	550	CB	2006
Aegilops	ventricosa	Tausch	1340284	España	Burgos	Pancorbo	30TVN8920	0	680		1997
Aegilops	ventricosa	Tausch	2513889	España	Burgos	Pancorvo	30TVN8920	0	680	CB	2006
Aegilops	ventricosa	Tausch	2513884	España	Burgos	Neila, Cerca de arroyo de	30TWM0056	0	1220		2006
Aegilops	ventricosa	Tausch	1379222	España	Cantabria	Colio, Cillorigo-Castro	30TUN68	0			1990
Aegilops	ventricosa	Tausch	2743404	España	Castellón	Morella, camino dels Lliv	30TYK4894		1000		2011
Aegilops	ventricosa	Tausch.	2791175	España	Castellón	Baix Maestrat, La Pobla d	31TBF5711		1200		2006
Aegilops	ventricosa	Tausch	1835151	España	Ciudad Real	Puebla del Príncipe, ermi	30SWH0666	0	860	CB	2005
Aegilops	ventricosa	Tausch	1835152	España	Ciudad Real	Villahermosa, Santa Mari	30SWH2288	0	760	CB	2005
Aegilops	ventricosa	Tausch	2074532	España	Cuenca	Barajas de Melo	30TWK04	0			2001
Triticum	ventricosum	Ces.	996413	España	Cuenca	Puente Vadillos	30TWK68	1			1948
Triticum	ventricosum	Ces.	996414	España	Cuenca	Beteta	30TWK79	1			1948
Triticum	ventricosum		2407179	España	Cuenca	Beteta	30TWK79	1			1942
Aegilops	ventricosa	Tausch	1469278	España	Cuenca	Beteta, valle del Tajo, Ho	30TWK9191	0	1350		1999
Aegilops	ventricosa		998075	España	Cuenca	Sierra de San Felipe	30TWK9970	0			1978
Aegilops	ventricosa		2242518	España	Cádiz	Tarifa	30STE69	1			1997
Aegilops	ventricosa	Tausch.	1684424	España	Cádiz	Cerro de San Cristóbal so	30STF87	0		CB	1978
Aegilops	ventricosa	Tausch	2333875	España	Córdoba	Cabra	30SUG7248	0			1986
Aegilops	ventricosa	Tausch	1385217	España	Córdoba	Cabra	30SUG7248	0			1987
Aegilops	ventricosa	Tausch	2309247	España	Córdoba	Cabra, alrededores	30SUG7248	0			1986
Aegilops	ventricosa	Tausch	2309248	España	Córdoba	Subbético, comprende C	30SUG95	1			1986
Aegilops	ventricosa	Tsch.	2364338	España	Granada	Granat., Granatae	30SVG41	1	833		1861-1862
Aegilops	ventricosa	Tausch.	1868449	España	Granada	Sierra Nevada circà San G	30SVG60	1	1525		1839
Aegilops	ventricosa	Tausch	1487854	España	Granada	P.N. Sierra Nevada	30SVG70	1			1987
Aegilops	ventricosa	Tausch.	1376740	España	Granada	Sierra de Baza, Parque Na	30SWG13	1			1991
Aegilops	ventricosa	Tsch.	2510552	España	Granada	Sierra de Castril	30SWG18	1			1905
Aegilops	ventricosa	Tsch.	2510554	España	Granada	Sierra del Cuarto	30SWH30	1			1905
Aegilops	ventricosa	Tausch.	2442839	España	Guadalajara	Millana	30TWK38	0			1984
Aegilops	ventricosa	Tausch.	2442838	España	Guadalajara	Salmerón	30TWK48	0			1984
Aegilops	ventricosa	Tausch	1374649	España	Huesca	Ontiñena	31TBG5716	0	230		1997
Aegilops	ventricosa	Tausch	1774966	España	Huesca	Monzón, pie del cerro de	31TBG6743	0	300		2004
Aegilops	ventricosa	Tausch	1374647	España	Huesca	Olvena	31TBG7365	0	460		1997
Aegilops	ventricosa	Tausch	1374645	España	Huesca	Roda de Isábena	31TBG9685	0	820		1997
Aegilops	ventricosa	Tausch.	1677768	España	Islas Baleares	Archipiélago de Cabrera,	31SDD93	1		CB	1975
Aegilops	ventricosa	Tausch	1481199	España	Islas Baleares	P.N. Archipiélago de Cabr	31SDD93	1			1997
Aegilops	ventricosa		1931161	España	Islas Baleares	Mallorca, Carretera a Cap	31SED06	1			1988
Aegilops	ventricosa	Tausch	1189438	España	Islas Baleares	Menorca, Camino de San	31SEE92	1			1874

Aegilops	ventricosa	Tausch.	1189440	España	Islas Baleares	Menorca, Rambàs	31SEE92	1			1901	
Aegilops	ventricosa	Tausch	1189437	España	Islas Baleares	Menorca, Santa Ponsa er	31SEE92	1			1874	
Aegilops	ventricosa	Tausch	1030249	España	Jaén	Noalejo Marceral	30SVG4053	0	1100		1996	
Aegilops	ventricosa	Tausch	1030248	España	Jaén	Noalejo Marceral	30SVG4053	0	1300		1996	
Aegilops	ventricosa	Tsch.	2511930	España	Jaén	(Massif de la Sagra, 1904	30SWG08	1			1906	
Aegilops	ventricosa	Tsch.	2510553	España	Jaén	Sierra de Cazorla	30SWH01	1			1905	
Aegilops	ventricosa		2068402	España	La Rioja	Zorraquín, Turgaiza	30TVM9786	0			2005	
Aegilops	ventricosa	Tausch	118438	España	León	La Pola de Gordón, Santa	30TTN85	0			1985	
Aegilops	ventricosa	Tausch	1135165	España	León	Santa Lucía de Gordón	30TTN85	0			1986	
Aegilops	ventricosa	Tausch	1135166	España	León	Bercianos del Real Camin	30TUM29	1			1986	
Aegilops	ventricosa		2749758	España	Madrid	Oteruelo del Valle	30TVL2629		1140		1988	
Aegilops	ventricosa		2749760	España	Madrid	Oteruelo del Valle	30TVL2729		1140		1988	
Aegilops	ventricosa		2749761	España	Madrid	Oteruelo del Valle	30TVL2729		1140		1988	
Aegilops	ventricosa		2749762	España	Madrid	Rascafría - Oteruelo del V	30TVL2729		1140		1988	
Aegilops	ventricosa		2749759	España	Madrid	Rascafría - Oteruelo del V	30TVL2729		1140		1988	
Aegilops	ventricosa		2749763	España	Madrid	Alameda del Valle	30TVL2830		1130		1988	
Aegilops	ventricosa		2749764	España	Madrid	Alameda del Valle	30TVL2830		1130		1988	
Aegilops	ventricosa		2749765	España	Madrid	Alameda del Valle	30TVL2830		1130		1988	
Aegilops	ventricosa		2749766	España	Madrid	Cruce de Pinilla del Valle	30TVL3031		1100		1988	
Aegilops	ventricosa		2749767	España	Madrid	Pinilla del Valle, plaza de	30TVL3131		1090		1988	
Aegilops	ventricosa	Tausch	1370910	España	Madrid	Redueña	30TVL5015	0			1997	
Aegilops	ventricosa	Tausch	1380640	España	Murcia	Moratalla, proximidades	30SWH6220	0	1200		1988	
Aegilops	ventricosa	Tausch	1359463	España	Murcia	Rincón de los Huertos, M	30SWH8333	0	1300		1993	
Aegilops	ventricosa	Tausch.	2383818	España	Málaga	Sierra de la Nieve Granat	30SUF26	1			1893	
Aegilops	ventricosa	Tausch	1043782	España	Málaga	Sierra de las Nieves	30SUF26	0			1990	
Aegilops	ventricosa		1580842	España	Palencia	Guardo	30TUN43	0	1120		1990	
Aegilops	ventricosa	Tausch	1593209	España	Palencia	Guardo	30TUN4939	0			1990	
Aegilops	ventricosa	Tausch	114935	España	Palencia	Guardo, Guardo	30TUN4939	0			1990	
Aegilops	ventricosa	Tausch	115633	España	Palencia	Guardo, Guardo	30TUN4939	0			1990	
Aegilops	ventricosa		1593457	España	Palencia	Muñeca	30TUN53	0	1180		1990	
Aegilops	ventricosa	Taussch.	2079078	España	Palencia	Alar del Rey	30TUN92	1			1956	
Aegilops	ventricosa	Tausch	1131637	España	Palencia	Alar del Rey	30TUN92	0			1986	
Aegilops	ventricosa	Tausch	62023	España	Salamanca	La Fuente de San Estebar	29TQF32	1			1978	
Aegilops	ventricosa	Tausch	62078	España	Salamanca	La Fuente de San Estebar	29TQF32	1			1978	
Aegilops	ventricosa	Tausch	59597	España	Salamanca	Monterrubio de Armuña	30TTL74	1			1991	
Aegilops	ventricosa	Tsch.	70874	España	Salamanca	Gomecello	30TTL84	1			1976	
Aegilops	ventricosa	Tsch.	70875	España	Salamanca	Pedrosillo el Ralo	30TTL84	1			1976	
Aegilops	ventricosa	Tausch	1106551	España	Salamanca	Cantalapiedra	30TUL0957	0	760		1988	
Aegilops	ventricosa	Tausch	1106550	España	Salamanca	Molino de la Villa	30TUL0957	0	760		1988	

Aegilops	ventricosa	Tausch	58084	España	Salamanca	Cantalapiedra	30TUL15	1			1987
Aegilops	ventricosa	Tausch	1385294	España	Segovia	Cañada del Río Pirón, Ad	30TVL1246	0	960		1987
Aegilops	ventricosa	Tausch	70816	España	Segovia	Santiuste de Pedraza	30TVL2850	0			1995
Aegilops	ventricosa	Tausch	70818	España	Segovia	Pedraza	30TVL2952	0			1995
Aegilops	ventricosa	Tausch	70817	España	Segovia	Gallegos	30TVL3448	0			1995
Aegilops	ventricosa	Tausch	92443	España	Segovia	Sepúlveda, Rendilón	30TVL3572	0			1989
Aegilops	ventricosa	Tausch.	1369201	España	Soria	Beltejar, hacia Blocona	30TWL46	0	1150		1998
Aegilops	ventricosa	Tausch.	1369203	España	Soria	Los Llamosos	30TWM31	0	1000		1998
Aegilops	ventricosa	Tausch.	1369205	España	Soria	Hinojosa de la Sierra, hac	30TWM33	0	1100		1998
Aegilops	ventricosa	Tausch.	1369207	España	Soria	Renieblas	30TWM52	0	1050		1998
Aegilops	ventricosa	Tausch.	1369209	España	Soria	La Rubia	30TWM53	0	1100		1998
Aegilops	ventricosa	Tausch.	1369211	España	Soria	Tejado, hacia Paridera Ca	30TWM60	0	1010		1998
Aegilops	ventricosa	Tausch.	1369213	España	Soria	Sierra del Almuerzo	30TWM63	0			1998
Aegilops	ventricosa	Tausch	85049	España	Soria	Sierra del Almuerzo	30TWM63	0			1985
Aegilops	ventricosa	Tausch	931082	España	Tarragona	Port Beseit	31TBF71	0			1983
Aegilops	ventricosa	Tausch.	2791176	España	Tarragona	Baix Ebre, Roquetes, Vall	31TBF7314		540		2006
Aegilops	ventricosa	L.	2155558	España	Teruel	Cedrillas, barranco de las	30TXK8379	0	1380		2009
Aegilops	ventricosa		1699514	España	Teruel	Mosqueruela	30TYK1570	0	1480		2002
Aegilops	ventricosa	Tausch.	1696530	España	Teruel	Puertomingalvo, entre M	30TYK1570	0	1500		2002
Aegilops	ventricosa		1700458	España	Teruel	Cantavieja	30TYK1987	0	1400		2002
Aegilops	ventricosa		1700271	España	Teruel	Cantavieja	30TYK1988	0	1340		2002
Aegilops	ventricosa		1700272	España	Teruel	Iglesuela del Cid, Masía d	30TYK2383	0	1300		2002
Aegilops	ventricosa		1700457	España	Teruel	Cantavieja, Masía de Por	30TYK2385	0	1400		2002
Aegilops	ventricosa	Tausch.	1696531	España	Teruel	Cantavieja, mas de Porca	30TYK2385	0	1500		2002
Aegilops	ventricosa	Tausch.	1696529	España	Teruel	Iglesuela del Cid, masico	30TYK3085	0	1500		2002
Aegilops	ventricosa	Tsch.	2364319	España	Valencia	regno Valent., mont. Eng	30SYJ01	1			1861-1862
Aegilops	ventricosa	Tausch	1476397	España	Valencia	Vallanca, hacia Salvacañe	30TXK3836	0	1050		1989
Aegilops	ventricosa	Tausch	2720848	España	Valladolid	Robladillo	30TUM4007		850		2007
Aegilops	ventricosa	Tausch	2720849	España	Valladolid	Encinas de Esgueva	30TVM02	1		CB	2007
Aegilops	ventricosa	Tausch.	56044	España	Valladolid	Encinas de Esgueva, Fuer	30TVM02	1			1985
Aegilops	ventricosa	Tausch	1125912	España	Valladolid	Encinas de Esgueva	30TVM0822	0			1986
Aegilops	ventricosa	Tausch	96897	España	Zamora	Pereruela, San Román de	30TTL69	1			1983
Aegilops	ventricosa	Tausch	76091	España	Zamora	La Bóveda de Toro	30TTL97	1			1984
Aegilops	ventricosa	Tausch	99836	España	Zamora	Carbajales de Alba	30TTM51	1			1982
Aegilops	ventricosa	Tausch	1115796	España	Zamora	Dehesa de Misleo	30TTM63	1			1984
Aegilops	ventricosa	Tausch	1115800	España	Zamora	Moreruela de Tábara	30TTM63	1			1984
Aegilops	ventricosa	Tausch	99835	España	Zamora	Moreruela de Tábara	30TTM63	1			1982
Aegilops	ventricosa	Tausch	1120229	España	Zamora	Abezames, Gafos	30TTM9911	0			1994
Aegilops	ventricosa	Tausch	76090	España	Zamora	Fuentelapeña	30TUL06	1			1984

Aegilops	ventricosa	Tausch	1643505	España	Zaragoza	El Castellar, Val de Zarag	30TXM7043	0	640		2003
Aegilops	ventricosa	Tsch.	1533642	España	Álava	Álava	30TWN23	1			1953
Aegilops	ventricosa	L.	2155557	España	Teruel	Sierra del Pobo	30TXK79	1		DUDOSO	2009
